# Abstracts from the 9th Biennial Scientific Meeting of the Asia Pacific Paediatric Endocrine Society (APPES) and the 50th Annual Meeting of the Japanese Society for Pediatric Endocrinology (JSPE): Tokyo, Japan. 17-20 November 2016

**DOI:** 10.1186/s13633-017-0054-x

**Published:** 2017-12-28

## PS1 Fat fate and disease - from science to global policy

### Peter Gluckman

#### Office of Chief Science Advsor to the Prime Minister

Attempts to deal with the obesity epidemic based solely on adult behavioural change have been rather disappointing. Indeed the evidence that biological, developmental and contextual factors are operating from the earliest stages in development and indeed across generations is compelling. The marked individual differences in the sensitivity to the obesogenic environment need to be understood at both the individual and population level. There is considerable evidence showing that the fetus and neonate are affected by the environment created by its mother and these have longer-term metabolic consequences including obesity and the implications for non-communicable disease (NCD). But while this evidence emerged over 30 years ago, it is only in the past 5 years it has gained traction in the policy domain. A number of barriers, both scientific and societal, existed to the incorporation of this knowledge into public policy. These barriers included an over-simplistic understanding of the phenomenon, a failure to appreciate its normative nature and multiple mechanisms that are best understood in a evolutionary medicine context, a lack of compelling biological mechanistic explanations and estimates of the size of the developmental component. These issues have largely been addressed to the extent needed for policy recommendations although there are areas of research that need further promotion – in particular those related to the development of eating behaviours and satiety control. However in part because of the Millennium Development Goals that brought an emphasis to maternal and girls’ health and because of scientific progress as reflected in the “ first 1000 days” movement, the first recognition of the developmental and epigenetic component to obesity was noted in the political declaration of the UN General Assembly in 2011. This led the WHO and an increasing number of governments to start to formulate developmental strategies to confront obesity. The World Health Assembly in May 2016 adopted the report of the Commission on Ending Childhood Obesity and this has major implications for national and global health policy. I will discuss these developments from both a scientific and public policy perspective.

## PS2 Bone Fragility in Children - Genes and Treatments

### Frank Rauch

#### Shriners Hospitals for Children

Heritable forms of primary bone fragility in children typically lead to a clinical diagnosis of either osteogenesis imperfecta (OI) or juvenile osteoporosis (JO). OI is usually caused by dominant mutations affecting one of the two genes that code for two collagen type I, but a recessive form of OI is present in 5-10% of individuals with a clinical diagnosis of OI. Most of the involved genes code for proteins that play a role in the processing of collagen type I protein (BMP1, CREB3L1, CRTAP, LEPRE1, P4HB, PPIB, FKBP10, PLOD2, SERPINF1, SERPINH1, SEC24D, SPARC,

TMEM38B), or interfere with osteoblast function (SP7, WNT1). Specific phenotypes are caused by mutations in SERPINF1 (recessive OI type VI), P4HB (Cole-Carpenter syndrome) and SEC24D (‘Cole-Carpenter like’). Patients with heritable bone fragility who do not have extraskeletal manifestations of OI are often diagnosed with JO. The JO phenotype can be caused by mutations in LRP5 and PLS3, but also by mutations in some of the genes that cause OI. For most of these gene defects the mechanisms linking mutation to phenotype remain to be elucidated. Regarding specific medical therapy of bone fragility in children, bisphosphonates are currently the main treatment option. Recent data indicate that children with moderate to severe OI have the ability to reshape most compressed vertebra, if treatment is started early enough and is continued throughout the growing period. However, bisphosphonate therapy does not have a major effect on the development of scoliosis and the incidence of long- bone fractures remains elevated. Even though children with moderate OI (OI type IV) typically achieve independent ambulation, this is rarely the case for children with severe OI (OI type III). Newer medications are being evaluated in an attempt to improve on the therapeutic efficacy of bisphosphonates but the available information about their action in OI is very limited at present.

## PS3 Unique Newly Discovered Muse Cells May Lead to the Paradigm Shift of Stem Cell Therapy

### Mari Dezawa

#### Department of Stem Cell Biology and History & Department of Anatomy and Anthropology, Tohoku University Graduate School of Medicine

Multilineage-differentiating stress enduring (Muse) cells are naturally existing unique stem cells that are non- tumorigenic and are pluripotent-like because they can generate cells representative of all three germ layers from a single cell, express pluripotency markers, and are able to self-renew and to spontaneously differentiate into cells compatible to the tissue they homed in vivo after engraftment. Whenever they are injected intravenously, they can escape from being trapped in the lung and spleen, unlike mesenchymal stem cells (MSCs), and efficiently home into damaged tissue, suggesting that robust repair can be delivered by intravenous injection of naïve Muse cells. Such unique functions of Muse cells were demonstrated in animal models of stroke, partial hepatectomy, skin ulcer of diabetes mellitus and muscle degeneration. They do not have to be “induced,” or genetically manipulated, to be pluripotent or be purposive cells before transplantation as required with some other cell varieties - they already display inherent pluripotent-like properties after isolation and, with their acquired properties of purposive cells, Muse cells spontaneously repair damaged sites based on their unique mechanisms.

They can be collected as cells positive for SSEA-3, a surface marker for pluripotent stem cells, from readily accessible sources such as the bone marrow (~0.03% of the total mononucleated cell population), and from cultured fibroblasts (several %), as well as from the dermis and adipose tissue. Thus, they are expected to be practical cells for clinical application. Recently, Muse cells are shown to circulate in peripheral blood in healthy donors, and the number increases in stroke patients in an acute phase, suggesting that endogenous Muse cells are mobilized into peripheral blood to repair tissues while their number is not sufficient to recover, and that supply of exogenous Muse cells is expected to deliver clinically relevant functional recovery. Overall, results suggest that Muse cells are a feasible and promising source for cell-based approaches.

## PS4 RASopathy - from molecular mechanism to clinical practice

### Yoichi Matsubara

#### National Center for Child Health and Development

Studies in the past decade revealed that a group of genetic disorders results from dysregulation of the Ras/ MAPK (mitogen-activated protein kinase) signaling pathway. These syndromes are comprehensively termed “Ras/ MAPK pathway syndromes” or “RASopathies”. These disorders include Noonan syndrome, Noonan syndrome with multiple lentigines (formerly called LEOPARD syndrome), Costello syndrome, cardiofaciocutaneous (CFC) syndrome, Noonan-like syndrome, neurofibromatosis type I, NF-llike syndrome, hereditary gingival fibromatosis, Legius syndrome and capillary malformation-arteriovenous malformation. Patients with these syndromes share many clinical features such as distinct facial appearances, developmental delays, cardiac defects, growth delays, and neurological disturbances. Molecular analyses identified mutations in genes related to the Ras/MAPK signaling pathway, namely PTPN11, SOS1, RAF1, KRAS, BRAF, NRAS, HRAS, MAP2K1/2, SHOC2, CBL, NF1, SPRED1 and RASA1.

More recently, novel gene variants, including RIT1, RRAS, RASA2, A2ML1, SOS2 and LZTR1, have been shown to be associated with RASopathies, further expanding the disease entity. Molecular characterization of these syndromes help to better elucidate an understanding of the pathogenesis of these disorders and aid in the development of potential therapeutic approaches. Model organisms, such as zebrafish and mice, harboring disease-causing mutations have been produced and they exhibit phenotypes resembling clinical pictures of patients. A series of compounds acting on the Ras/MAPK pathway seem to ameliorate various symptoms, holding promise for treating patients with these syndromes.

## SY1-1 Growth: New era of growth disorders

### How to approach growth disorders: genetics for better clinical care

#### Andrew Dauber

##### Cincinnati Center for Growth Disorders, Division of Endocrinology, Cincinnati Children fs Hospital Medical Center

Human growth is a complex process governed by a multitude of biological process. Traditionally, genetic analysis has played a limited role in the pediatric endocrinologist’s evaluation of patients with growth disorders. Focused candidate gene testing is employed in cases of distinct syndromes (i.e. Leri-Weill Dyschondrosteosis, Noonan Syndrome, Russell Silver Syndrome) or clear defects in the growth hormone/IGF-I axis. We will first explore the role of candidate gene testing in the evaluation of short stature and growth hormone deficiency. We will then turn to the use of genomic technologies including whole exome sequencing and chromosomal microarrays for the identification of rare genetic sequence and copy number variants underlying individual patient’s short stature. We will review a proposed algorithm for the diagnosis of growth disorders.

## SY1-2 Growth: New era of growth disorders

### IGF2 mutation in Silver-Russell syndrome

#### Thomas Eggermann

##### Institute of Human Genetics, University Hospital, Aachen Technical

With the identification of 11p15.5 disturbances in Silver-Russell syndrome (SRS) ten years ago, the central role of at least the telomeric imprinted region ICR1 in 11p15.5 for the etiology of the disease became obvious. Two of the genes regulated by the ICR1 are IGF2 and H19. Due to its physiological function and its imprinting status, IGF2 had been suggested as the causative gene for SRS, but the final clue was still missing. We recently identified a paternally inherited IGF2 mutation in a family with growth retardation and SRS, we could confirm the hypothesis that IGF2 is the key factor for the growth retardation and other features in SRS. However, molecular studies in further SRS families as well as in sporadic patients did not identify further cases, thus IGF2 mutations do not significantly contribute to the mutation spectrum in this disorder. However, in the last decade it has emerged that IGF2 and other imprinted genes belong to common functional networks, either because of their physiological function or because they belong to the imprinted genes network (IGN), and molecular alterations of genes of IGF2 interaction partners also contribute to growth retardation. Comprehensive studies in our patient cohort (n>500) referred for SRS diagnostic testing helped us to identify further molecular alterations in the 11p15.5 region itself, but also in other genes and chromosomes (e.g. GRB10 in 7p13, MEST in 7q31, IGF1R in 15q26). Our data and those from animal and cellular models indicate that IGF2 and the coregulated H19 gene as well as further imprinted and/or functionally related factors belong to the IGN and the IGFII-IGF1R signaling cascade by cis and trans acting mechanisms. These findings contribute to a better understanding of human growth and its disturbances. The translational use of this knowledge has significantly improved molecular diagnostics of SRS and will have an impact on personalized patient management.

## SY1-3 Growth: New era of growth disorders

### Treatment Strategies for Short Stature in Achondroplasia

#### Hiroshi Kitoh

##### Department of Orthopaedic Surgery, Nagoya University Graduate School of Medicine

Achodnroplasia (ACH) is one of the most common short-limbed skeletal dysplasias characterized by rhizomelic shortening of the extremities, frontal bossing with midface hypoplasia, and trident hands. ACH is caused by gain- of-function mutations in the fibroblast growth factor receptor 3 (FGFR3) gene, which is a negative regulator of longitudinal bone growth. No effective treatments for short stature in ACH are currently available. The limb lengthening is a therapeutic option to gain bone length, but the treatment period for bone maturation is very long which results in higher rates of complications.

We have established a novel cell therapy using culture-expanded bone marrow cells (BMC) and platelet rich plasma (PRP) during the limb lengthening to accelerate callus formation. We have performed this cell therapy in more than 130 bones since 2002. The clinical outcome of the limb lengthening in ACH was significantly improved by BMC and PRP transplantation. The effectiveness of this cell therapy, however, was limited for skeletally mature patients due to their limited osteoblastic differentiation of BMC.

For further improvement of our cell therapy, we tried to search clinically applicable drugs that promote the activity of Runx2, which plays a pivotal role in the differentiation of mesenchymal cells to the osteoblastic lineages. By drug repositioning (DR) strategy, we found that a proton pump inhibitor, lansoprazole, enhanced nuclear accumulation of Runx2, promoted expressions of various osteoblastic genes, and induced osteoblastogenesis of human BMC. Systemic administration of lansoprazole to a rat femoral fracture model increased osteoblastogenesis. Lansoprazole could be applicable for ex vivo culture to obtain better quality of BMC.

We next tried to search drugs that suppress abnormally activated FGFR3 signaling in ACH. We found that meclozine, an antihistamine drug that has long been used for motion sickness, facilitated chondrocyte proliferation and mitigated loss of extracellular matrix in ACH model cells. Meclozine also suppressed abnormally activated FGFR3 signaling in various chondrocytic cells that were infected with human mutants. In bone explant culture, meclozine increased the longitudinal growth of embryonic tibia from ACH model mice. After 3 weeks administration of meclozine, the body and tail length, and the individual bone length was significantly longer in meclozine-treated ACH mice than in untreated ACH mice. The plasma concentration of meclozine during treatment was within the range that has been used in clinical settings for motion sickness. These results indicated potential clinical feasibility of meclozine for the improvement of short stature in ACH.

## SY2-1 Puberty: Mechanisms and disorders of puberty

### Neuroendocrine mechanism regulating puberty onset

#### Hiroko Tsukamura

##### Nagoya University

Puberty, which is associated with a maturation of the gonadal axis, is timed by an increase in tonic gonadotropin release from the anterior pituitary, where synthesis and release of gonadotropins are stimulated by gonadotropin- releasing hormone (GnRH) released from the hypothalamus. Kisspeptin, encoded by Kiss1, is a potent stimulator of gonadotropin release via inducing GnRH release, and plays a critical role in puberty onset, because inactivating mutation of Kiss1 or GPR54, a kisspeptin receptor gene, causes pubertal failure in rodents and humans. Accumulating evidence suggests that KNDy neurons, which express kisspeptin, neurokinin B and dynorphin A, in the hypothalamic arcuate nucleus (ARC) are a key regulator of tonic GnRH/gonadotropin release, which governs gonadal activity, i.e., gametogenesis and steroidogenesis in both sexes. Most probably, pubertal maturation of gonadal axis is triggered by an increase in Kiss1/kisspeptin expression in the ARC KNDy neurons in rodents. It is likely that estrogen derived from the immature ovary plays a critical role in prepubertal restraint of ARC Kiss1/ kisspeptin expression, and that a decrease in sensitivity to the estrogen negative feedback during peripubertal period is a key event for pubertal maturation of the hypothalamic mechanism regulating GnRH and then gonadal activity in rodents.

## SY2-2 Puberty: Mechanisms and disorders of puberty

### Central precocious puberty caused by mutations in the imprinted gene MKRN3

#### Ana Paula Abreu

##### Universidade de Sao Paulo/ Brigham and Women's Hospital

Puberty and reproduction are controlled by the hypothalamic-pituitary-gonadal axis. This axis is active during the embryonic and neonatal stages of human life but then suppressed during childhood, then ultimately re-activated, first detected as an increase in amplitude and frequency of gonadotropin-releasing hormone (GnRH) pulses, to initiate puberty. The precise mechanisms that regulate GnRH secretion to constrain the HPG axis during infancy and childhood and subsequently trigger the initiation of puberty remain elusive. Complex interactions among genetic, nutritional, and environmental factors influence pubertal initiation. Pubertal timing is associated with risks of subsequent disease. Early activation of the hypothalamic–pituitary–gonadal axis results in gonadotropin- dependent or central precocious puberty, which is clinically evidenced by pubertal initiation before 8 years old in girls and 9 years old in boys. The genetic determinants of the timing of human pubertal development and in particular CPP are largely unknown. In 2013, we identified loss-of-function mutations in a single gene, MKRN3, encoding makorin ring finger protein 3, in five of 15 families with central precocious puberty, using an exome sequencing approach. MKRN3 is located on chromosome 15q, in the Prader-Willi Syndrome critical region, and is maternally imprinted, expressed only from the paternally inherited allele. Following this inheritance pattern, all MKRN3 mutations identified in patients with central precocious puberty were inherited from their fathers. To date, MKRN3 is the most common genetic defect associated with central precocious puberty in families from several different ethnic groups. MKRN3 belongs to the Makorin family, a family of E3 ubiquitin ligases. Its protein structure has a ubiquitin ligase domain. The mutations identified in patients with central precocious puberty are expected to disrupt protein function. MKRN3 expression in the arcuate nucleus of mice is high prepubertally and decreases before puberty initiation, reaching very low levels in adult life. Taken together, these findings suggest that the loss of function of MKRN3 results in the release of a restraint mechanism on the GnRH pulse generator, which in turn releases the brake on puberty. MKRN3 is the first gene with an inhibitory role on GnRH secretion with mutations identified in humans.

## SY2-3 Puberty: Mechanisms and disorders of puberty

### Mechanisms of pubertal growth spurt and growth plate closure

#### Kye Shik Shim

##### Kyung Hee University Hospital at Gangdong

Growth of height is increased in early or mid-puberty and is known as pubertal growth spurt, but decreased and ceased after growth plate closure in late puberty. Therefore, the pubertal growth spurt has a growth-promoting effect and the growth plate closure reveals a limiting effect on bone elongation. However, the mechanisms behind these contrary phenomena during puberty are still not exactly known.

The elongation of long bones results from chondrocyte differentiation, proliferation and hypertrophy, and extracellular matrix secretion in proliferative and hypertrophic zone of epiphyseal plates.

The growth plate closure is thought as the result of epiphyseal senescence due to the loss of differentiation potential of chondrocytes in resting zone of epiphyseal plates.

The complex networks of genetic, nutritional, cellular, paracrine, and endocrine factors are considered closely related with pubertal growth spurt and growth plate closure.

The genetic factors include the genes linked with intracellular, extracellular, paracrine and endocrine factors. The intracellular factors contain chondrocyte transcription factors, Ras-MAPK signaling, and centrosomal proteins, etc. The extracellular factors are the cartilage extracellular matrixes that contain specific collagen, non-collagenous proteins, and proteoglycans, etc.

The important paracrine factors that regulate the growth plate chondrocytes are parathyroid hormone-related protein/Indian hedgehog pathway, fibroblast growth factor and C-type natriuretic peptide signaling.

The major endocrine systems that control pubertal growth are GH-IGF-I and estrogen signaling.

Therefore, a lot of factors include the cellular factors involved with chondrocyte differentiation, proliferation and hypertrophy, and multiple molecular pathways related with chondrocyte differentiation, vascularization, and ossification in the growth plate are considered influencing each other. However, the complex interactions of these factors are still unclear. Elucidation of the detailed mechanisms of the pubertal growth spurt and growth plate closure will allow a better understanding of the molecular mechanisms responsible for idiopathic short stature, precocious puberty, and skeletal disorders. It will also contribute to the development of new therapeutic modalities for those disorders with better efficacy and. fewer side effects.

## SY3 Iodine deficiency disorders

### Somchit Jaruratanasirikul

#### Songkla University

Iodine is a trace element that is essential for thyroid hormone synthesis, and the adequate production of thyroid hormone is essential for brain development, physical growth and control of metabolic processes in the body throughout the lifespan. In pregnant women, thyroid hormone is essential for neurological development of the fetus. During the infancy period, iodine is also essential for neurological development, being involved in neuronal cell migration, myelination and various other functions.

The major source of iodine is from exogenous or dietary intake. However, dietary sources provide only a small amount of iodine which is usually insufficient to meet the daily requirement. Long duration of iodine insufficiency leads to iodine deficiency disorder which is a significant public health problem in many countries. Iodine deficiency- related problems such as goiter, hypothyroidism, growth stunting, and cognitive impairment are relatively common. Various national policies have been enacted to eliminate conditions related to iodine insufficiency. Iodine supplementation through salt iodization is an effective and sustainable strategy for preventing iodine deficiency. Biomarkers used to monitor iodine status at the population level include urinary iodine excretion (UIE), goiter rates, and serum thyroid stimulating hormone (TSH) levels. In recent decades the more extreme manifestations of iodine deficiency such as cretinism or multinodular goiter have significantly decreased, however the various less severe manifestations noted above are still prevalent. Hence, national surveillance strategies towards establishing the iodine status in a country’s population should be regularly carried out. In common clinical practice, spot urinary iodine levels for measuring the median iodine excretion are generally used to assess iodine status in population studies.

The most susceptible group for iodine deficiency disorder is women of reproductive age, whose offspring, if iodine deficient in utero, are at high risk of irreversible mental impairment. The other susceptible group is women providing breast milk to their children, as this may be the only source of iodine during the first 6 months of life. Low concentrations of thyroid hormone during the fetal stage and early infancy are associated with irreversible brain damage, including mental retardation and neurological abnormalities. The main factors influencing the extent and magnitude of neurological complications arising from iodine deficiency are the timing and severity of the deficiency and consequent thyroid hormone deficits. Subtle impairment of cognitive function is likely to occur even in offspring of pregnant women with mild or asymptomatic hypothyroidism.

## SY3-2 Thyroid: Management of thyroid disorders

### The long term outcome of subclinical congenital hypothyroidism

#### Sarah Mathai

##### Department of Pediatrics, Christian Medical College

The biochemical diagnosis of congenital primary hypothyroidism(CH) is made when serum TSH level is elevated and Free T4(FT4) level is low/low normal. Newborn babies have a physiological TSH surge soon after birth (70- 100 mIU/ml) which declines by 72 hours of age. TSH level remains rather stable (< 6 mIU/ml) between one month of age and adult life. Elevated TSH level (>6mIU/ml) with normal FT4 levels beyond the neonatal period may be defined as subclinical congenital hypothyroidism(SCCH).

Hypothyroidism adversely affects growth & cause irreversible developmental delay especially in young children, therefore urgent initiation of levothyroxine(LT4) supplements is recommended in CH. However overtreatment may cause hyperactivity, bone age advancement and craniosynostosis. In children with SCCH, decision to treat or not during early infancy is very difficult. The American Academy of Pediatrics recommend treatment if TSH is persistently > 10 mIU/ml beyond 2 weeks of age irrespective of T4 levels. There are no definite guidelines regarding initiation of treatment in infants with TSH levels 6-10 mIU/ml.

Among the factors implicated in subclinical hypothyroidism, the most important is maternal iodine deficiency. In fact maternal iodine status rather than the maternal thyroid status has been reported to adversely affect the cognitive development in children according to a recent study and this group has demonstrated improvement in neurodevelopmental outcome with maternal iodine supplementation.

There is limited data on the long term outcome of SCCH in children. The outcomes reported are ongoing subclinical hypothyroidism even up to late adolescence, adverse neurodevelopmental outcome especially verbal skills and development of overt hypothyroidism. Children who had persistent TSH elevation have been reported to have abnormal thyroid scintiscans. While better cognitive outcome has been reported with initiation of low dose LT4 supplements during infancy, some studies have also reported bone age advancement. In our institution we have screened 150,000 newborns for CH over the last 15 years. In our practice we often do not commence LT4 supplements for children with SCCH but periodic monitoring is done. We have assessed their growth and development and the results are awaited.

In conclusion, CH should be diagnosed based on S.TSH and T4/FT4 levels to prevent overdiagnosis and overtreatment. Demonstration of abnormal thyroid scintiscan in children with SCCH may predict persistence of TSH elevation. All children with SCCH whether treated or not need long term monitoring. Optimising maternal iodine and thyroid status may be simple and effective in reducing SCCH prevalence and its adverse long term impact on children.

## SY3-3 Thyroid: Management of thyroid disorders

### Management of autoimmune thyroiditis

#### Andrew Bauer

##### The Children's Hospital of Philadelphia; Perelman School of Medicine, The University of Pennsylvania

Autoimmune thyroid disease (AITD) is comprised of two distinct but related disorders, Graves’ disease (GD) and Hashimoto’s thyroiditis (HT). In both disorders T-cells infiltrate the thyroid resulting in tissue disruption as well as activating B-cells to produce anti-thyroid antibodies. GD is associated with the production of a TSH-receptor antibody that stimulates dysregulated overproduction of thyroid hormone. In contrast, HT is associated with thyrocyte apoptosis and production of anti-thyroglobulin (TgAb) and anti-thyroid peroxidase (TPOAb), with TPOAb further reducing thyroid hormone production via decreased organification. Iodine excess may increase the risk for developing AITD, in particular HT. Early identification and treatment of autoimmune thyroid disease in children and adolescents is critical to optimize growth and development. In addition, there is evidence that both GD and HT may increase the risk for developing thyroid nodules and thyroid cancer. We will review the evaluation and management of these two entities with a focus on subclinical hypothyroidism and the definitive treatment of GD.

## SY4-1 New Strategy

### Next-generation sequencing and array-based comparative genomic hybridization for patients with pediatric endocrine disorders

#### Maki Fukami

##### National Research Institute for Child Health and Development

Recent advances in molecular technology including next-generation sequencing (NGS) and array-based comparative genomic hybridization (aCGH) enabled researchers to perform high-throughput mutation screening and genome-wide copy number analysis for multiple samples. To date, we have performed NGS and aCGH analyses for more than 500 patients with pediatric endocrine disorders. Representative results include the flowing. [1] Molecular diagnosis of patients with hypogonadism or hypospadias: We performed NGS-based mutation screening of all known causative genes and aCGH-based genome-wide copy-number analysis. We analyzed 58 patients with hypogonadotropic hypogonadism and identified pathogenic molecular defects in 14 patients. Oligogenicity was not evident in our patient group. As rare abnormalities, we identified a submicroscopic deletion involving FGFR1, an SOX3 polyalanine deletion, and a WDR11 splice site mutation. Then, we also analyzed 62 patients with non-syndromic hypospadias. We found that monogenic and digenic mutations in known causative genes and cryptic copy-number alterations account for more than 10% of cases. [2] Identification of genomic rearrangements underlying aromatase excess syndrome: We performed aCGH for male patients with hereditary gynecomastia. The results suggest that microdeletions, microduplications, and complex rearrangements around CYP19A1 encoding aromatase lead to estrogen overproduction and resultant gynecomastia. These data provides novel models of human disorders caused by cryptic genomic rearrangements. [3] Whole exome sequencing of patients with disorders of sex development (DSD): We performed whole exome sequencing for individuals with various types of disorders of sex development. As a result, we detected intragenic mutations and upstream deletion of SOX9 in 46,XY DSD patients without campomelic dysplasia. These data expand the phenotypic spectrum of known monogenic disorders. Furthermore, we identified novel gene mutations that may be associated with DSD.

This session aims to introduce the usefulness of NGS and aCGH in the field of pediatric endocrinology.

## SY4-2 New Strategy

### Generating Functional Miniature Organs (Organoids) in a Dish

#### Hidenori Akutsu

##### Department of Reproductive Medicine, National Research Institute for Child Health and Development

Pluripotent stem cells can generate virtually any cell type and, as such, can be used to model development and disease and even hold the promise of providing cell-replacement therapies. Structures resembling whole organs, termed organoids, have been generated from stem cells through the development of three dimensional culture systems. Organoids are derived from pluripotent stem cells that differentiate to form an organlike tissue exhibiting multiple cell types. However, no one reported that organoids in vitro possessed similar functions of “real” organs. Intestines are generated through a highly orchestrated, serial developmental process and are composed of cell types from all three primary germ layers. Directed differentiation of human pluripotent stem cells (hPSCs) can yield gut-specific cell types; however, these structures do not replicate some of the critical functional interactions between cell types of different germ layers. Recently, we have developed a simple protocol based on a tissue self- organization concept for generation of mature functional intestinal organoids from hPSCs under xenogeneic- free conditions. The organoids showed the gut tube-like architecture consisted of mucosa and submucosa by histological, immunofluorescence and electron micrograph examinations. Stem cell-derived miniature guts (mini-guts) were derived from all three germ layers and contained distinct types of intestinal cells, including enterocytes, goblet cells, Paneth cells, and enteroendocrine cells. They demonstrated intestinal functions including peptide absorption and innervated bowel movements responsive in response to stimulation with histamine and anticholinergic drugs. Here, I present our recent achievements of mini-guts and discuss about future applications with 3D organoids.

## SY4-3 New Strategy

### Clinical steroid mass spectrometry and metabolomics

#### Cedric HL Shackleton

##### Institute of Metabolism and systems research (IMSR), University of Birmingham / UCSF Benioff Children fs Hospital Oakland Research Institute

This talk will summarise the 80 year history of steroid metabolomics in diagnosis, ending with latest developments in tandem MS and machine interpretation of GC/MS metabolomic data.

The characterization and measurement of steroids in health and disease has been fundamental to endocrinology since the 1930s. The first example of a diagnostic steroid for an endocrine disorder was the discovery of pregnanetriol in the urine of patients with the adrenogenital syndrome (CAH) by Guy Marrian in 1937. For decades, paper and TLC dominated metabolomic (urinary steroid) approaches to steroid analysis, while from the 1960s RIA of circulating hormones and precursors was pre-eminent and remains useful.

Mass spectrometry now dominates the steroid analytical field but it has taken a long time to reach this position. The breakthrough came in the mid-1960s, when a Swedish company introduced the LKB 9000 GC/MS instrument combining the separation powers of gas chromatography with the structural information of mass spectrometry. In 1967 this presenter had the privilege to use the first British LKB in Professor Charles Brooks’ lab in Glasgow to document the structures of steroids excreted by the human newborn.

In the 50 years of steroid mass spectrometry there have been continuous advances, notably for GC/MS the introduction of capillary columns, chemical derivatisation methods and advanced data systems.

A most important advance was combining HPLC and MS and the introduction of MS/MS to provides high specificity and sensitivity. Automated HPLC MS/MS systems now allow quick separations and accurate measurement of vanishingly small amounts of hormonal steroids. The high throughput has rendered HPLC/tandem MS the desired method for routine clinical serum steroid measurements. In contrast to serum steroids, HPLC/MS of urinary steroids was challenging because of the metabolite structures involved but in the last two years quality comprehensive urinary steroid profiles have been produced and the method will be central to metabolomics.

In spite of being labour intensive GC/MS still holds its own for metabolomic studies, although an ongoing drawback of complex metabolomic data production is interpretation by the operator and explaining diagnostic findings to the interested clinician. To that end my colleagues in Birmingham (Prof Wiebke Arlt. Elizabeth Baranowski, Angela Taylor, Tulay Gulan, Michael Biehl, Kerstin Bunte, Peter Tino and others) have developed a novel interpretable machine learning technique, Angle Learning Vector Quantisation , designed to distinguish multiple biosynthetic error conditions in adults and children using the urinary steroid metabolome. Predictions with close to 100% specificity and sensitivity. Interestingly, pregnanetriol, the mother of steroid metabolites, remains a central analyte with our metabolic profile studies almost 80 years after Marrian’s painstaking identification.

## SY5-1 Obesity: Increasing risk of morbidity in children and adolescents

### The role of MC4R in pediatric obesity: above and beyond the central nervous system

#### Feihong Luo

##### Deparment of Pediatric Endocrinology and Inborn Metabolic Diseases, Shanghai, China

Obesity is a worldwide health problem. Approximately 21-24% children and adolescents are overweight, and 16-18% of individuals have abdominal obesity. Obese 10- to 13-year-old children are 6 to 7 times more likely to become obese adults, when compared with their nonobese peers 50% to 65% of adults with severe obesity (>180% of ideal weight) were obese as children. Obesity is a complex, multifactorial condition with high heritability. Increasing evidence that environmental and nutritional influences during infant and early childhood’s development can have lasting effects on adult’s predisposition to obesity and metabolic syndrome. We analyzed the successive anthropometric data from birth to 24 months and found parental pre-pregnant BMI played the most important role in their offspring’s body size after 6 months age; the total gestational weight gaining during pregnancy of the mother steadily explains nearly a quarter (25 ± 5%) of the relative importance of children’s weight; the weight of the parents is the major factor influencing the children’s weight after 6 months of age, this complex feature of human early growth highlights the underlying association with epigenetic and genetic regulation. While, among the known obese-causing single gene defects which result in human obesity, MC4R deficiency is probably the most frequent. The prevalence of loss-of-function MC4R mutations ranges from 0.5% to 5.8% in childhood onset obesity. A generational effect was observed, with a penetrance of 40% in MC4R-deficient adults aged>52 years, 60% in 18-52 year-old adults and 79% in children. MC4R is expressed by distinct neurons in the CNS that synapse with neurons that express the pomc gene, and produce a α -MSH-related end-product. The role of MC4R as a regulator of feeding behavior in mice was established not long after the characterization of the mc4r gene. The binding of α -MSH to neurons in the hypothalamus that express mc4r will decrease feeding behavior. Conversely, point mutations of the mc4r gene that render the receptor nonfunctional result in the onset of obesity. The patients with MC4R mutations were obese from an early age, but with increase in both fat and lean masses, and were excessively hungry from 6–8 months of age with hyperphagia and persistent food-seeking behavior. Besides of the well- established central MC4R pathways, recent research revealed that MC4Rs expressed on intestinal enteroendocrine L cells regulate PYY and GLP-1 secretion, two gut hormones implicated in the regulation of energy and glucose homeostasis. Increasing evidences support the notion that MC4R may have a variety of physiological roles in the pathogenesis of pediatric early onset obesity.

## SY5-2 Obesity: Increasing risk of morbidity in children and adolescents

### Management of hypothalamic obesity

#### Mitchell Eugene Geffner

##### Keck School of Medicine of USC

Hypothalamic obesity is typically an intractable form of obesity that was initially described in patients with craniopharyngiomas usually following surgical treatment and unintentional damage to hypothalamic nuclei, which results in disruption of energy regulation. However, this definition has now expanded to include obesity developing after a variety of hypothalamic insults, both acquired and congenital (such as other hypothalamic tumors, pituitary macroadenomas with suprasellar extension, gliomas, meningiomas, teratomas, and germ cell tumors; radiotherapy, Prader-Willi syndrome, and mutations in the leptin, leptin receptor, POMC , MC4R , and CART genes). The pathogenic mechanisms underlying hypothalamic obesity are complex and multifactorial, including damage to the ventromedial hypothalamus, which leads to hyperphagia; a low-resting metabolic rate; autonomic imbalance; hypomobility; and insomnia. Additional mechanisms by which the brain regulates adipose tissue and β -cells of the pancreas include the sympathetic nervous system, vagally-mediated hyperinsulinemia, and the endocrine system, namely the growth hormone, thyroid, and hypothalamo-pituitary-adrenal axes. Medical therapy to date (methamphetamine derivatives, somatostatin analogs, GLP-1 and its analogs, oxytocin, and MetAP2 inhibitors have been tried with varying degrees of success), with bariatric surgery now coming under study. Understanding the central role of the hypothalamus in the regulation of feeding and energy metabolism may also be critical to help gain insight into the pathogenesis and management of common obesity.

## SY5-3 Obesity: Increasing risk of morbidity in children and adolescents

### Determinants, consequences and prevention of childhood overweight and obesity

#### Muhammad Yazid Jalaludin

##### Department of Paediatrics, Faculty of Medicine, University Malaya

Over the recent years, the prevalence of childhood obesity has increased rapidly worldwide, likely as a result of complex interactions between genes, dietary intake, physical activity, and the environment. The determinants of childhood obesity is multifactorial and involves a complex relationship among various non-modifiable and modifiable factors. Apart from genetic factors, maternal obesity, gestational diabetes and adiposity rebound are some of the important non-modifiable factors. Evaluating modifiable factors contributing to obesity (weight gain during infancy, physical activity, diet, sleep, parental feeding practices, screentime, parental obesity and families from lower socioeconomic status) is an important step towards effective intervention.

Childhood obesity is associated with significant short and long-term health and social consequences. Childhood obesity affects nearly every organ of the body. Short term health impacts such as hypertension, dyslipidaemia and insulin resistance (metabolic syndrome) which were earlier considered markers of adult cardiovascular disease, are now common among obese children. Long term health impacts of childhood obesity although less commonly seen by paediatrician, include premature cardiovascular diseases, type 2 diabetes, non-alcoholic fatty liver disease (NAFLD), obstructive sleep apnoea syndrome (OSAS), infertility and orthopaedic pain.

Prevention is more effective than treatment in dealing with the rising prevalence of childhood obesity. Roots of obesity can be traced to very early in life and appropriate prenatal and postnatal nutrition can help to reduce the risk of overweight/obesity in adulthood. Establishing healthy dietary and lifestyle habits after birth and throughout the childhood and adolescent period can help reduce the risk of childhood obesity. Child health clinics, school health screening programmes, as well as general paediatricians/family physicians are at the first line of defense even before the BMI crosses the acceptable range and hence play a crucial role in prevention and treatment of childhood obesity. Routine anthropometric measurement are essential during clinic visits to detect overweight/ obesity at an early stage, thus permitting necessary actions to be taken.

Once the child’s BMI crosses the overweight percentile, individualise interventions are critical for successful weight management. The Staged Approach recommended by the American Academy of Paediatrics (AAP) involves multi- disciplinary team as early as Stage 2. The need for multi-disciplinary team involvement increases as the problem escalate.

## SY6-1 Which is most dangerous for future cognition in young children with diabetes: hypo- or hyperglycemia?

### Diabetes dysglycaemia, cognition and the developing brain

#### Fergus Cameron

##### Royal Children fs Hospital

Glucose is the preferred metabolite of the brain with 25% of circulating blood glucose in adults destined for cerebral metabolism. It is intuitive then that type 1 diabetes mellitus (T1DM), a disorder characterised by perturbations in blood glucose (“dysglycaemia”), should cause acute and chronic brain dysfunction. These cognitive and affective impacts appear to be greatest in the developing brain of children and adolescents with T1DM. Aspects of diabetic dysglycaemia that appear to be most significant are hypoglycaemia, hyperglycaemia and diabetic ketoacidosis (DKA). Some early in vitro work suggest that glycaemic variability may also play a significant role in neural cell injury. Prospective observational data from the point of diagnosis to neuromaturation in the Royal Children’s Hospital Diabetes Cohort Study revealed a rather lamentable “rule of thirds”- one third of subjects developed a DSM IV threshold mental health disorder, one third did not complete secondary schooling and one third did not continue in adult care after transition. This was coincident with a 0.3 SD loss of full scale IQ and changes in regional brain volumes. More recent functional imaging studies have provided insight into some of the mechanistic aspects of dysglycaemia-induced brain injury. DKA at the point of diagnosis is associated with acute grey and white matter volume and spectroscopic changes that are associated with neurocognitive outcome in the medium term. Clamp studies combined with MRI have shown that hypo- and hyperglycaemia result in distinct regional changes in brain perfusion and metabolic activity. Potential synergies of chronic and additive dysglycaemic insults are difficult to quantitate largely due to an inability to fully record all aspects of glycaemic perturbation over a life course. In addition to this, pre-conditioning and programming may also play significant mediating roles. However, developmental age at the time of diabetes onset appears to have a critical influence upon outcome. A nascent understanding of mechanism of neural injury is providing some insights as to potential non-glycaemic interventions that might be used to protect the developing brain.

## SY6-2 Which is most dangerous for future cognition in young children with diabetes: hypo- or hyperglycemia?

### Improving metabolic control without increasing severe hypoglycemia

#### Karin Åkesson

##### National Swedish Padiatric Diabetes Registry

Over the last years the metabolic control in children with type 1 diabetes in Sweden has improved and the mean HbA1c in year 2015 is 57.3 mmol/mol, IFCC (7.4 %, DCCT). Data on almost all (>97 %) children, 0-18 years of age, with type 1 diabetes in Sweden is registered in Swediabkids, the Swedish Pediatric Diabetes Quality Registry. Swediabkids includes data on HbA1c, BMI, insulin dose and frequency of insulin pumps as well as on acute and chronic complications. This allows us to continuously follow changes in the frequency of severe hypoglycemia when HbA1c is decreasing. The new technology, with Continuous Glucose Monitoring and advanced insulin pumps, has made it possible to have a tight control of the children’s glucose level. The children are taught to correct their glucose level if it is below 4 or above 8 mmol/mol and to always give dextrose to normalize a low glucose level. These recommendations make the children and their families highly involved in their own treatment. The families as well as the personnel in schools and day care centers are taught to count carbohydrates and to give insulin correction doses during the days. Since July 2009 a new law is implemented in Sweden to secure the rights of children with diabetes. The pediatric diabetologist or nurse has to make a detailed agreement with the parents or other caregivers to what extent the child needs help with the diabetes self-care during school day. This agreement should be written down in a national uniform action plan form. By giving the child and its close environment the possibility to take command over the treatment and by extra support from the pediatric diabetes team when this is needed, it is possible to improve metabolic control without increasing severe hypoglycemia.

## SY6-3 Which is most dangerous for future cognition in young children with diabetes: hypo- or hyperglycemia?

### Closed loop systems to avoid extreme hypo- and hyperglycemia

#### Martin de Bock

##### Princess Margaret Hospital

Closed loop insulin delivery systems aim to closely regulate blood glucose levels by using algorithms that interpret real-time sensor glucose values and adjust insulin delivery in real time, without increasing the risk of hypoglycaemia. These systems have been extensively tested in closely regulated scenarios including hotels and diabetes camps – all of which have shown favourable glycaemic outcomes compared to standard pump therapy. The first long term, unsupervised outpatient trials have now been conducted, confirming that these results can be translated into real life settings. Closed loop insulin delivery systems are expected to be commercially available within the next 2 years, heralding a new era for patients with type 1 diabetes, where tight glycaemic control can be achieved, without the burden of hypoglycaemia or requirement for frequent patient initiated management strategies.

**Fig. 1 (abstract SY6-3).**
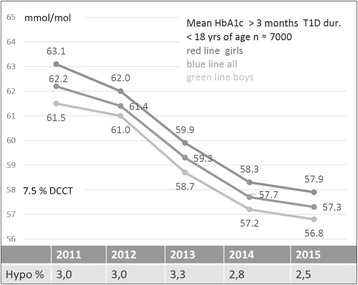
See text for description

## SY7-1 DSD: Variations in Sex Development and in Gender Identity

### Initial approach to Disorders of Sexual Differentiation

#### Kah Yin Loke

##### National University of Singapore

The disorders of sexual differentiation (DSD) comprise many conditions of diverse pathophysiology which present in the newborn with atypical external genitalia or in the adolescent with atypical sexual development during the pubertal years. These clinical situations are difficult to manage and an initial comprehensive evaluation is necessary to (1) assign the appropriate gender, (2) develop a logical, pragmatic plan for investigation and (3) organize the necessary medical, surgical and psychological intervention.

The initial diagnostic clinical evaluation requires a thorough prenatal and family history. Dysmorphic features and midline facial defects may provide clues to the underlying cause. External genitalia should be examined for the presence and symmetry of gonads in the labioscrotal folds, fusion of the folds, phallic size and site of the urinary meatus on the phallus, from which the external masculinization score can be derived.

For infants with ambiguous genitalia, investigations can be ordered based on:

Bilateral non-palpable gonads: first line tests include sex chromosome karyotyping, serum electrolytes, serum 17-hydroxyprogesterone [17OHP], testosterone, LH, FSH, and a pelvic ultrasound for female internal organs.

The normal 46 XX chromosomal female with elevated 17OHP levels and the presence of a uterus suggests congenital adrenal hyperplasia (CAH). (ii) For infants with chromosomal karyotype other than 46 XX, the second line tests aimed to determine the presence of a testes and the adequacy of androgen production include measuring the anti-Mullerian hormone, HCG stimulation test, with further imaging and laparoscopy. Confirmation of a specific diagnosis may require genetic analysis.

Palpable gonads (likely to be testis / ovotestis): sex chromosomal karyotyping should be performed, followed by second line tests indicated above.

Adolescents with DSD may present as:

Girls with primary amenorrhoea, which may be caused by DSD with 46 XY sex reversal (gonadal dysgenesis, testosterone biosynthetic defects, androgen insensitivity syndrome [AIS], LH receptor mutations). Initial relevant investigations will include a chromosomal karyotype, LH, FSH, androgen levels and pelvic ultrasound, followed by an HCG stimulation test.

Girls who virilise at puberty, which can be caused by DSDs with undetectable Mullerian structures (17 β

-hydroxysteroid dehydrogenase deficiency, 5 α -reductase deficiency, partial AIS), and DSDs with detectable Mullerian structures (partial gonadal dysgenesis, ovotesticular DSD, CAH, androgen secreting tumours). Relevant investigations include a pelvic ultrasound, LH, FSH, dehydroepiandrosterone, serum androgen with their precursor levels and 17OHP.

## SY7-2 DSD: Variations in Sex Development and in Gender Identity

### Long-term outcome of early surgical intervention for hypospadias; What happens at Puberty?

#### Kimihiko Moriya

##### Department of Renal and Genitourinary Surgery, Hokkaido University

Hypospadias (HS) is one of the most common congenital anomalies in male children. The goal of HS surgery is reconstructing the urethra to the tip of the glans and straightening the penis. Successful HS surgery ensures a cosmetic penile appearance, voiding in the standing position and unhampered sexual function in adulthood. Despite great surgical interest in short-term results, there are only a few studies regarding the long-term outcome. In this lecture, I will present a long term outcome of HS surgery, focusing on the cosmetic and endocrinological outcome at puberty from our long-term outcome study.

As a first study, to clarify the cosmetic and sexual outcome, a questionnaire was mailed to HS patients and control subjects at 18 years old or older. This study demonstrated that although their sexual behavior was not different from that in control subjects, HS patients had a higher rate of dissatisfaction with smaller penile size. Following the outcome of the questionnaire study, actual penile length of HS patients was evaluated. As a result, while the severity of HS and endocrinological abnormality at post-pubertal evaluation were factors affecting penile size, penile length in patients with severe HS was shorter even in cases without endocrinological abnormality. These results suggest that severe HS is not only a disorder of urethral development, but also a disorder of penile development.

A multifactorial etiology is considered to be involved in the genesis of HS. Among them, androgen actions have an important role for urethral development during fetal period. Several studies have shown a prepubertal hormonal abnormality in patients with HS from the endocrinological viewpoint. However, reports of hormonal status in HS patients at puberty are scarce. Therefore, we retrospectively reviewed endocrinological status at puberty. Endocrinological abnormality was identified in patients with mild as well as severe HS. While severity of HS did not affected the incidence of high serum FSH, history of the undescended testis was a risk factor for high serum FSH at post-pubertal evaluation.

In conclusion, penile size, which is most dissatisfied issue from the patients’ view point at puberty, is affected by severity of HS. In addition, history of undescended testis is an important issue from the endocrinological view point, especially on reproductive function. Since both testicular descent and urethral formation are influenced by androgen action, coexistence of these pathology would be a severe form in the spectrum of male genital anomaly form the reproductive view point.

## SY7-3 DSD: Variations in Sex Development and in Gender Identity

### Transgender Youth: Current Concepts, Management, and Priorities for Research

#### Stephen Rosenthal

##### Division of Pediatric Endocrinology, Child and Adolescent Gender Center, University of California, San Francisco

An increasing number of preadolescents and adolescents identifying as “gender nonconforming”, “gender expansive”, or “transgender”, are seeking medical services to enable the development of physical characteristics consistent with their experienced gender. The onset of puberty in transgender youth is often accompanied by increased gender dysphoria, the persistent distress associated with incongruence between assigned sex at birth and experienced gender identity. While recent reports from the Netherlands have shown that gender dysphoria may be ameliorated by a gender-affirming model of care, including puberty suppression, cross sex hormones, and gender confirming surgery, there are relatively limited outcomes data, and many questions remain unanswered. This presentation will highlight current concepts of the biology of gender identity development, management of gender dysphoria, gaps in knowledge, and priorities for research. It is hoped that such research will inform optimal care of transgender youth and promote further understanding and acceptance of these individuals.

## SY8-1 Workshop on Developing countries

### A report on the first year of the APPES-CLAN Equity Working Group. Where to from here? What can you do to help?

#### Kate Armstrong

##### President & Founder, CLAN (Caring & Living As Neighbours)

The APPES-CLAN Equity (ACE) Working Group was established in 2015 as a formal collaboration between the Asia Pacific Pediatric Endocrinology Society (APPES) and CLAN (Caring & Living As Neighbours) to drive long-term, sustainable change that redresses inequity and improves quality of life for children living with endocrine conditions in the Asia Pacific region.

The ACE Working Group agreed to adopt a rights-based, community development approach to achieving equity for children living with endocrine conditions, and committed to the principles of: participation; accountability; non- discrimination and equality; empowerment; and legal, policy and ethical frameworks. CLAN’s strategic framework for action and five pillars further informed the ACE Working Group partnership as early priorities were considered:

- Affordable access to medicines and equipment
- Education, research and advocacy
- Optimal medical management
- Encouragement of family support groups and
- Reducing financial burdens on families.

Deliverables for the first six months of the ACE Working Group included: a mapping exercise to determine the most vulnerable paediatric endocrine communities in the Asia Pacific region; development of equity markers to track achievements and improvements in health outcomes for children affected by endocrine conditions; -collaborative action with CAH communities and APPES members to address barriers to accessing essential medicines and equipment identification of existing educational resources for paediatric endocrine communities, with information made publicly available online; development of a clear strategic plan to inform action improving access to hydrocortisone and fludrocortisone for the CAH Community in the region; support of fellows training, informed by community priorities; utilisation of communication platforms to promote activities of the partnership; advocacy and action relating to universal access to newborn screening for congenital hypothyroidism in the region; strengthening networks between paediatric endocrine communities and associated professional societies clarification of barriers to financial independence for paediatric endocrine communities, with particular focus on school attendance and health insurance.

This presentation will report on the achievements of the ACE Working Group in its first year, including practical examples of collaborative action already transforming the lives of children in the Asia Pacific region. The session will be an opportunity for APPES members and multisectoral partners to engage with ACE Working Group members and collaboratively explore opportunities for involvement in future activities.

## SY8-2 Workshop on Developing countries

### Challenges for Pediatric Endocrinology in a resource constrained environment

#### Syed Jamal Raza

##### National Institute of Child Health

Development of Pediatric subspecialties is a relatively new frontier for certain countries where overall healthcare scenario is still far behind the developed world. Lack of adequate finances is a major but not the only constraint that obstructs growth of specialties.

There are many other barriers and challenges in developing, establishing and flourishing of the subspecialty. Access to health care, which is a general problem, also affects disorders of endocrinology especially in situations where you need urgent care like Diabetic Ketoacidosis, salt wasting adrenal crises etc.

However, the most pressing and difficult problem is the lack of trained personal. In Pakistan, a country of population over 180 million have only a handful of trained Pediatric Endocrinologist. Lack of trained personal not only makes it difficult to establish the discipline but also results in a dearth of training opportunities for local doctors. This situation can be helped greatly by other countries by providing funded training opportunities for doctors from resource-constrained countries. Once enough trained personal become available, local training programs can be developed, subsequently reducing the need for people to go abroad from training.

Lack of availability of affordable endocrine investigations have also been and will remain a barrier in developing services. With more sophisticated (and hence expensive) tests becoming available which on one hand improve the diagnostic acumen but remain difficult to perform even in large towns and centers and even more in peripheral or small towns with limited health facility. Molecular work in diagnosis has now become a norm, and most of the conditions can only be diagnosed with certainty with molecular testing (especially DSD). This not only has prognostic implications for patients but also imperative for prenatal testing. Facilities for molecular testing are not only expensive, but also require technical manpower which is difficult to obtain and retain in public sectors which is only place the majority can access.

Drug availability has also unfortunately been a problem in many such countries and simple, essential life saving drugs like hydrocortisone and fludrocortisone are not registered and regularly available in country like Pakistan. This brings a disastrous situation for the patients when despite the diagnosis they cannot be adequately treated.

So, in conclusion, there are many barriers and challenges still withholding development in resource poor countries. Most can be overcome with advocacy, planning and some help from the developed world.

## SY8-3 Workshop on Developing countries

### Building Newborn Screening Systems

#### Dianne Webster

##### Newborn Metabolic Screening Programme / Antenatal Screening for Down Syndrome & Other Conditions

Newborn baby screening for congenital hypothyroidism (CH) is a proven, well accepted public health initiative but implementation generally is problematic in developing economies. The first consideration is that of prioritisation of public health initiatives within the jurisdiction and newborn screening may be perceived as of lower benefit and priority than eg immunisation or elimination of disease carrying vectors or provision of clean water. Following a decision to screen items for consideration include who (start with private payers? With hospitals?); what condition/s? what cutoff/s? who will collect samples, test, organise followup and treatment? Payment for treatment? Audit and monitoring? Of high importance is definition of the goals of screening in terms of health outcomes eg for CH growth and intellectual development optimal for the individual. Achievement of health outcomes requires definition of a newborn screening system incorporating not only the sampling and laboratory testing (relatively easy to establish) but also the maternity services and facilities, diagnostic and treatment pathways including paediatrics, pharmacy, followup monitoring testing, family information and support that are essential to ensure that an infant with a high TSH level achieves their full potential. Collection and transport of samples is generally difficult especially outside urban areas and consideration should be given in the initial stages of a programme of testing for conditions where the baby will benefit from detection of the condition even if this is delayed beyond what many programmes would consider optimal (receipt in laboratory by 4-5 days of age) such as CH; and to starting in an urban setting, with families able to pay if this is the only way to begin screening. Followup of positive tests has been an issue in some programmes and an argument can be made for setting the screening cutoffs at values which give high positive predictive values until the followup system is established. Conditions such as CAH where the testing and notification is time critical can be added, the population screened broadened and cutoffs lowered when the infrastructure is in place. The gradual approach to implementation enables building of the multiple relationships and processes which ensure the best outcomes for affected infants.

## SY9-1 Hot topics

### Epigenetics as the mediator of gene:environment interactions and programming of non-communicable disease

#### Richard Saffery

##### Murdoch Childrens Research Insitute and Department of Pediatrics, University of Melbourne

The Developmental Origins of Health and Disease (DOHaD) hypothesis is based largely on observational studies in humans and direct experimentation in animal models. Several mechanisms have been proposed to account for the latency between an “exposure” in utero and ‘programming’ of later disease risk, including epigenetic variation induced early in development. Environmental sensitvity is a hallmark of epigenetic variation and exogenous exposures have the potential to influence epigenetic profile, modifying the developmental trajectory and risk of disease.

Animal studies have demonstrated conclusively that the in utero environment can modify the epigenetic profile in progeny in association with long term (even intergenerational) phenotypic consequences, though replication in such models remains fragmented. Equivalent data in humans are also beginning to emerge. However, to establish epigenetics as the mediator of ‘programming’ in humans several criteria must be met. First, interindividual differences in epigenetic profile must be present in early life. Second, environmental exposures implicated in later life phenotypic outcomes, must be linked to specific epigenetic variants. Third, such epigenetic variants should be reproducibly associated with later life phenotypic outcomes. Fourth, specific epigenetic variants must have demonstrated functional consequences on gene activity or cellular function.

It is now clear that genetic variation ‘shapes’ the human epigenetic profile throughout life and may play a role in mediating the effects of environmental exposures on epigenetic variation. Specific in utero exposures (such as maternal smoking) induce defined epigenetic changes in newborns, some of which persists into adulthood.

Mounting evidence also links early epigenetic variation to later onset phenotypes and non-communicable diseases. The field is advancing at a rapid pace but remains in its infancy. The recent adoption of standardised platforms for epigentic analysis, the advent of several very large prospective birth cohorts, and the formation of large consortia to build sample sizes with sufficient power to detect small magnitude effects, represent major advances. Ultimately, a complementary approach encompassing (i), well controlled and reproducible animal studies, (ii) ongoing human longitudinal observational cohorts, and (iii) well-informed interventional studies, represents the best way forward to firmly establish epigentic variation as a mediator of DOHaD-related health outcomes in humans.

**Author details**: Professor Richard Saffery is National Health & Medical Research Council (Australia) Senior Research Fellow with 20 years experience in Molecular and Cellular Biology, including 15 in the field of Epigenetics. He heads a team investigating the determinants of the human epigenetics profile in early life and its role in later health outcomes. This involves state-of-the-art multidisciplinary approaches, encompassing genetic, environmental and epigenetic analysis. Central to our work has been the establishment of several longitudinal birth cohorts with detailed environmental, anthropometric, clinical and other data, with associated biorepository.

Prof Saffery has published over 170 papers, including key discoveries demonstrating the interaction of genetic, environmental, temporal and tissue-specific factors in regulating the early life epigenetic profile in humans. His team has received over $15M in funding and has developed an international reputation in early life human epigenetics research. Prof Saffery currently leads the Epigenetics Work Package for a large multinational European Commission (FP7) funded project examining the early life determinants of metabolic health in children.

## SY9-2 Hot topics

### Obesity and Brain Insulin Resistance: Cause of Consequence

#### Hans-Ulrich Häring

##### Department of Internal Medicine IV, University Hospital Tübingen

The Tübingen family study collects people with increased risk to develop type 2 diabetes and comprises by now approx. 3.000 deeply phenotyped individuals. We used wholebody MRI imaging to assess bodyfat distribution patterns. We could identify subphenotypes of bodyfat distribution, a metabolically healthy phenotype of obesity (MHO) versus a metabolically unhealthy phenotype (MUHO) as well as distinct patterns of perivascular adiopose tissue as well as neckfat.

In a subgroup of approx. 250 individuals we studied brain insulin effects by MEG and fMRI and observed brain insulin resistance in obese people. We used nasal insulin as a tool to study brain effects in the fMRI.

Stimulation of brain insulin signalling by nasal insulin caused brain responses related to homeostatic regulation, eating behaviour but also caused changes of peripheral insulin sensitivity as determined by glucose clamp. Based on stable isotope techniques this brain derived signals affect both glucose appearance from the liver as well as glucose disappearance. Low hypothalamic insulin sensitivity correlated with increased visceral fat and decreased subcutaneous fat. We hypothesize that the brain insulin resistance causes unfavourable fat distribution patterns as a consequence of impaired signalling from the brain to the periphery favouring accumulation of visceral fat. This mechanism might also favour the develoment of NAFLD.

## SY9-3 Hot topics

### MIRAGE Syndrome: A New Adrenal hypoplasia Syndrome Caused by Heterozygous SAMD9 Mutations

#### Naoko Amano

##### Department of Pediatrics, Keio University School of Medicine Department of Pediatrics, Saiseikai Central Hospital

Most patients with primary adrenal insufficiency (PAI), such as 21-hydroxylase deficiency, can be diagnosed biochemically by measuring urine or serum steroid metabolites, while a minor subset of PAI patients lacks diagnostic biochemical features. We analyzed known causative genes in biochemically uncharacterized PAI patients and diagnosed genetically about 70% of them. Nonetheless, etiologies of the remaining 30% were not elucidated. By exome sequencing and follow-up genetic analyses in 24 early-onset PAI patients with biochemically and genetically unknown etiologies, we identified heterozygous SAMD9 mutations in 11 patients. According to the common clinical features among these 11 patients, we named this disorder ‘MIRAGE’ syndrome. 1) Myelodysplasia; All patients showed transient thrombocytopenia and/or anemia in early infancy. Two patients subsequently developed myelodysplastic syndrome and died in early childhood. 2) Infection; All patients experienced severe invasive infections, and six patients died before age two years. 3) Restriction of growth; All patients were born preterm (gestational age, 25 to 35 weeks), small-for-gestational age (birth weight, -4.0 to -2.2 SD), and also had severe growth restriction after birth. 4) Adrenal hypoplasia; All seven evaluated patients showed adrenal hypoplasia by ultrasonography. Postmortem analysis of two female patients revealed that their adrenal glands were extremely small and highly disorganized. The adrenal medulla was only partially surrounded by the adrenal cortex, and the cytoplasm of dysgenetic adrenocortical cells were foamy in appearance. 5) Genital phenotypes; All seven patients with 46,XY karyotype had underdeveloped external genitalia. Postmortem analysis of the two female patients revealed that their ovaries were extremely small and had very few primordial cells. 6) Enteropathy; All nine evaluated patients had chronic diarrhea with colonic dilation.

In this symposium, I will talk about identification of a new causative gene for PAI, SAMD9 , clinical features of MIRAGE syndrome and some functional studies of the identified SAMD9 mutations.

## MTE1 Bone

### Pediatric Osteoporosis

#### Craig Munns

##### The Children's Hospital at Westmead

Up to 50% of children fracture before age 18 years, with a peak incidence during early adolescents. Of these who fracture, approximately 20% will sustain a second fracture. Although childhood fracture is usually associated with trauma there are increasing data to indicate that fracture incidence in children is related to reduced bone mineral density and relative skeletal fragility.

It is impractical and unnecessary for the bone health to be assessed in every child who presents with fracture. The ISCD 2013 Pediatric Official Position on Fracture Prediction and the Definition of Osteoporosis in Children and Adolescents (1) has recommended:

Evaluation of bone health should identify children and adolescents who may benefit from intervention to decrease their elevated risk of a clinically significant fracture

A clinically significant fracture history is one or more of the following:

- Two or more long bone fractures by age 10 yr
- Three or more long bone fractures at any age up to 16 yr.

Vertebral compression fracture (loss of 20% height at any point).

One or more vertebral compression fractures are indicative of osteoporosis. In such children and adolescents, measuring BMD adds to the overall assessment of bone health. Children who fulfil the above criteria should undergo a bone health evaluation.

So, what investigations should be performed in a child with recurrent fracture? This can be guided by the clinical features of the child but there are ‘routine screening’ investigations that may be considered in the majority of children. It is important to assess mineral homeostasis and vitamin D levels. Coeliac and thyroid disease should be screened. Dependent on the age of the child, gonadal status may be evaluated. All children with recurrent fracture should have a lateral spine xray to assess for vertebral compression fracture. This is especially the case in children with low trauma fracture, osteogenesis imperfecta or if there is a history of glucocorticoid therapy. Duel energy xray absorptiometry (DXA) remains the gold standard for assessment of reduced bone density and bone mineral content for age. The caveats of DXA in children will be discussed.

Management of recurrent fracture in children centres around reduction of risk factors and improvement in muscle and bone mass. Optimising vitamin D, calcium and pubertal progression are also important as is the judicious use of bisphosphonates and other bone specific therapy.

**Reference**

Bishop et al. 2014 Journal of Clinical Densitometry: Assessment & Management of Musculoskeletal Health Volume 17.

## MTE2 Endocrine Imaging

### The state-of-the-art applications of PET in endocrinology

#### Tohru Yorifuji

##### Endocrinology and Metabolism, Children's Medical Center, Osaka City General Hospital

## MTE3 Growth & Genetics

### Genetic Approaches to make a Diagnosis in Short Stature

#### Peter Clayton

##### Faculty of Biology, Medicine & Health, University of Manchester

Over the last 30 years many monogenic causes of growth disorders, including hormonal, primordial, skeletal and syndrome-associated, have been identified by examining candidate genes or hunting for the causative gene in regions of homozygosity or gene rearrangement. For instance mutations in most of the components of the GH-IGF axis have been identified, as have the causes of major skeletal dysplasias.

In more recent years, genome wide association studies (GWAS) have found variants in a number of these genes contribute to adult stature, and revealed that many other genes, as yet not implicated in growth disorders, also contribute. In addition array studies have shown that copy number variants (CNVs) are more commonly found in short stature. Alongside these observations, advances in genetic technologies have led to the increasing use of targeted gene panels (e.g. for rasopathies) and whole exome (or genome) sequencing (WES) in growth disorders. This is leading to diagnoses of a particular condition that had not been anticipated based on phenotype.

We therefore have powerful tools to achieve a diagnosis, but how should they be used in the approach to a child presenting with significant short stature (height SDS <-2). Attention to defining auxology (including limb to axial skeleton proportions) and growth pattern in relation to stature and development in the family, obtaining a comprehensive history of potential risk factors and/or occult system disease, and carefully documenting overall phenotype remains the cornerstone of initial assessment. This clinical evaluation is likely to trigger a request for investigations that may include a screen for system disease, a skeletal survey, hormonal evaluation and an array study. In some cases, the clinical features may immediately point to a specific genetic condition, and if so, then a traditional search for a mutation in a single gene could be undertaken. If there are suggestive features, a targeted gene panel would be appropriate. In those whose short stature appears to be of undefined aetiology (so called idiopathic short stature [ISS]), then WES can provide an opportunity to make a diagnosis.

These testing strategies have led to the recognition that genes known to cause short stature syndromes with well-defined clinical features may be mutated in ISS children. This extends the phenotype, and can lead to the recognition of more subtle features associated with that condition. These strategies may also identify genes implicated in growth physiology but not previously recognized to cause human disease. The CNV and whole exome/genome approaches may lead to genes never previously recognized as related to growth being implicated as causes of short stature. In this situation it is important that defects in these genes are found in multiple families to confirm causality.

The use of genetic technologies will vary considerably dependent on the health-care resource setting. Studies evaluating large cohorts of unselected ISS children are required to evaluate the cost-effectiveness of using next generation sequencing to improve molecular diagnosis rates.

## MTE4 Growth

### New data on long-term safety of GH treatment in SGA

#### AnitaHokken-Koelega

##### Erasmus University Medical Center

**Background**: Children born SGA have a higher risk to develop metabolic diseases in adulthood. GH treatment increases adult height but also causes insulin resistance, which have raised concerns about the long-term consequences of GH treatment in children born SGA. Published data of the SAGhE project suggested an increased cardiovascular mortality in adults born SGA who were treated with GH during childhood. The main limitation of the SAGhE project is that data of ex-GH-treated adult patients are compared with national reference values and not with an age-matched untreated adult patients. To study the long-term effects of GH treatment on the risk of diabetes mellitus type 2 and cardiovascular diseases, it is important to compare data of previously GH-treated adults born SGA with those of untreated adults born SGA.

**Outline**: This session will give an overview of published GH safety data and will present recently published data of the largest follow-up study in 199 previously GH-treated SGA young adults who were longitudinally studied for five years after attainment of adult height, at GH-cessation, six months, two and five years thereafter. Body composition, insulin sensitivity and β -cell function were determined and data at five years after GH-cessation were compared with untreated age-matched controls: 51 untreated short SGA adults, 92 SGA adults with spontaneous catch-up growth, and 142 adults born appropriate for gestational age.

**Main findings**: Data show a steady increase in fat mass during five years after GH treatment, indicating the loss of pharmacologic effects of GH. Reassuringly, the GH-induced reduction in insulin sensitivity was fully reversed within six months after cessation of GH treatment and remained similar thereafter, despite the increase in fat mass. At 5 years after GH-cessation, fat mass, insulin sensitivity and β -cell function of previously GH-treated SGA adults were similar to untreated short SGA adults, indicating that long-term GH treatment in children born SGA has no unfavourable effect on metabolic health in early adulthood.

## MTE5 Water and electrolytes

### Management of water and electrolytes imbalance

#### Joseph Majzoub

##### Boston Children's Hospital, Harvard Medical School

Maintenance of the tonicity of extracellular fluids within a very narrow range is crucial for proper cell function. Extracellular osmolality regulates cell shape as well as intracellular concentrations of ions and other osmolytes. Furthermore, proper extracellular ionic concentrations are necessary for the correct function of ion channels, action potentials, and other modes of intercellular communication. Extracellular fluid tonicity is regulated almost exclusively by the amount of water intake and excretion, whereas extracellular volume is regulated by the level of sodium chloride intake and excretion. Normal blood tonicity is maintained over a 10-fold variation in water intake by a coordinated interaction among thirst, arginine vasopressin (AVP)/anti-diuretic hormone (ADH), and renal systems. Dysfunction in any of these systems can result in abnormal regulation of blood osmolality, which if not properly recognized and treated may cause dysfunction in neuronal and other cellular activities. When the relationships among water intake, vasopressin status, and renal responsiveness are not understood, life-threatening hyponatremia or hypernatremia may occur, whereas knowledge of these systems almost always allows for proper management of sodium and water balance without these complications.

**Learning objectives:** As a result of participating in this session, learners should be able:

- To understand how to treat a patient with diabetes insipidus or SIADH with combinations of fluid and AVP or DDAVP, without causing hyper- or hypo-natremia. When AVP/DDAVP is high (anti-diuretic state), a patient can only tolerate 1/10th as much fluid as when vasopressin/DDAVP is low (diuretic state).
- To diagnose cause of polyuria: central diabetes insipidus versus primary increased fluid intake
- To use measurement of blood copeptin instead of AVP
- To diagnose cause of hyponatremia: dehydration/reduced intravascular volume versus SIAD
- To treat diabetes insipidus in neonates without causing hyponatremia

## MTE6 Adrenals

### Diagnosis and Management of CAH

#### Toshihiro Tajima

##### Jichi Children's Medical Center Tochigi

Congenital adrenal hyperplasia (CAH) refers to a range of autosomal recessive diseases resulting from deficiency of cortisol secretion. The incidence is 1/10,000-20,000 globally and 21-OHD is the most frequent disease among CAH cases. Many patients with 21-OHD have pigmentation, virilization of the external genitalia in females, failure to thrive and poor weight gain, but others have 21-OHD with only very mild clinical symptoms. Elevated blood 17-hydroxyprogesterone is the best biochemical diagnostic marker fro diagnosis. Furthermore, tests for diagnosis and understanding of the pathological condition include plasma ACTH, serum electrolytes, plasma glucose, plasma aldosterone, plasma renin activity or concentration, and blood gas. If 21-OHD is suspected due to clinical symptoms and abnormal test results, and a manifestation of adrenal insufficiency is found, treatment must be the priority.

The principles of treatment of 21-OHD are to supplement insufficient glucocorticoid and mineralocorticoid levels, inhibit enhanced adrenal androgen production, and maintain growth and maturation similar to those of healthy children. Treatment continues for life. Insufficient treatment causes adrenal crisis (acute and severe adrenal insufficiency) due to decreased tolerance to physical stress and short stature due to bone age advancement. Excessive treatment causes iatrogenic Cushing's syndrome, including short stature, obesity and hypertension.

In the neonatal stage, since adrenal androgen production is markedly enhanced, high dose of hydrocortisone (HC) (25-100 mg/m2) is needed to suppress adrenal androgen. HC is also used as maintenance therapy and the dose of glucocorticoids during maintenance therapy must be carefully determined to prevent overdose and underdose. Salt wasting 21-OHD is not sufficiently treated with HC alone and requires fludrocortisone (FC). Sodium intake from breast milk and bottle formula is insufficient for treatment in the infant stage, and sodium chloride replacement is also necessary. A dose of FC in maintenance therapy is 0.025 to 0.2 mg/day. The FC dose should be adjusted based on the plasma renin activity or concentration, electrolyte levels; and body weight gain. Adverse reactions including increased blood pressure and edema should be monitored.

It is also very important to avoid adrenal insufficiency. If a patient has febrile illness, gastroenteritis with dehydration, surgery or trauma, it is necessary to increase the dose of glucocorticoids transiently. A child at an early age has a risk for hypoglycemia and electrolyte imbalance; therefore, long-term fasting conditions should be avoided, and intravenous administration of glucose and sodium should be performed as required.

## MTE7 Thyroid

### Medical and Surgical Management of Pediatric Thyroid Nodules and Cancer

#### Andrew Bauer^1,2^, Ken Kazahaya^3,4^

##### ^1^The Children's Hospital of Philadelphia; ^2^Perelman School of Medicine, The University of Pennsylvania; ^3^Division of Pediatric Otolaryngology, The Children's Hospital of Philadelphia; ^4^Department of Otorhinolaryngology, Head and Neck Surgery, University of Pennsylvania

Evaluation and management of nodular thyroid disease in the pediatric population is different from the adult population. Thyroid nodules and cancer in the pediatric population have distinct biology, pathophysiology, clinical findings, and prognosis. For example, thyroid nodules are less frequent in childhood than in adulthood, but are more often malignant. Previous guidelines for the management of thyroid nodules and cancers were geared toward adults. Diagnostic work up or treatment that may be recommended for an adult may not be appropriate for a child. In 2015, the American Thyroid Association (ATA) formulated and published guidelines for evaluation and management of children and adolescents with thyroid tumors. The intent of the guideline was to provide recommendations to ensure stratification of care based on complete and accurate pre-operative assessment with thyroid/neck ultrasound and fine needle aspiration. Pediatric specific post-operative risk levels were also created to identify patients at risk of persistent disease that would benefit from radioiodine therapy. The evaluation and management of patients with persistent and/or recurrent disease were also reviewed with recommendations directed at reducing surgical complications as well potential complications associated with repeat radioiodine therapy.

We will address the initial work up, operative management and postop care in the pediatric population. In addition to review of the key points outlined in the ATA pediatric guidelines, we will also discuss additional strategies from our practice to reduce complications of therapy as well as more recent data from the literature.

**Table 1 (abstract MTE7).** See text for description

ATA Pediatric Risk Level	Definition	Initial Post-operative Staging
Low	Disease confined to the thyroid with minimal level VI lymph node metastasis	TSH suppressed Tg at 3 month intervals with neck ultrasound at 6 months
Intermediate	Extrathyroidal extension with extensive level VI or lateral neck lymph node metastasis	TSH-stimulated Tg and diagnostic 123I whole body scan
High	Regionally extensive disease with or without distant metastasis	TSH-stimulated Tg and diagnostic 123I whole body scan

## LS9 New Genetic Causes of Short Stature: A Primer for Endocrinologists

### Andrew Dauber

#### Cincinnati Center for Growth Disorders, Division of Endocrinology, Cincinnati Children's Hospital Medical Center

In this talk, we will review recent advances in the field of growth genetics. These defects include mutations in genes within the growth hormone/IGF-I axis, including the recently discovered mutations in the PAPPA2 and IGF2 genes, as well as mutations in genes outside this axis. We will review a few genes with direct effects at the growth plates (ACAN and NPR2). We will also briefly discuss new findings in primordial dwarfism including a network of DNA damage repair genes in which mutations cause severe short stature. Finally, there will be a brief overview of the lessons learned from genomewide association studies about the genetics of height in the general population.

## LS10 Current Status of the long-term Safety of GH Treatment

### Peter Clayton

#### Faculty of Biology, Medicine & Health, University of Manchester

Pituitary-derived growth hormone (GH) was first used in the 1950s and withdrawn in the mid-1980s because of its link with Creutzfeld-Jacob disease, with recombinant human GH (r-hGH) now being in use for >30 years. Over that time its safety and efficacy in children and adults has been subject to considerable scrutiny. R-hGH is used under licence in a wide range of disorders, including growth failure associated with a number of diverse aetiologies in children, GH deficiency in adults with onset in childhood or adulthood, and HIV/AIDS-wasting in adults. It has also been used in clinical trials to assess its impact in skeletal dysplasias, steroid-induced growth failure, poor growth associated with inflammatory disorders and fracture healing, as well as off-label use.

There are therefore significant challenges in evaluating safety in all these contexts. These include: lack of an untreated comparator population, relatively small sample sizes, inadequate long-term data on GH exposure, in particular dosages, inconsistent definitions of diagnoses and outcomes, and reporting bias of adverse events. Nevertheless there is a large literature primarily from observational studies while on r-hGH that indicates an overall good safety record, with no increase in de-novo cancer or tumour recurrence in children. There are a number of well recognised but infrequent side-effects (intracranial hypertension, musculoskeletal symptoms, obstructive sleep apnoea, bone problems [usually related to the underlying condition e.g. scoliosis], and alterations in thyroid and cortisol metabolism).

Despite r-hGH’s safety record, there have been underlying concerns related to the potential impact of the physiological actions of r-hGH on cell growth and metabolism, that could associate with cancer risk and cardiometabolic effects, and hence an overall effect on mortality.

To address these concerns, large cohorts of patients with well-characterised diagnoses and treatment exposures need to be assembled and followed long-term, both while on r-hGH and in the years after treatment is completed. The ‘Safety and Appropriateness of GH treatments in Europe’ study was established to assemble a large group of patients, who were at least 18 years of age and had been treated in childhood only with r-hGH. This cohort was first assembled in France, followed by 7 other European countries. This has resulted in a metacohort of ~25,000 individuals, most commonly treated for growth failure due to isolated GH deficiency, idiopathic short stature or short stature in children born small for gestational age (53%), Turner syndrome (13%) and growth hormone deficiency linked to neoplasia (12%), with >400,000 person years of follow-up and an average duration of 17 years per patient.

To date results on cancer incidence, mortality and vascular complications have been reported from the French cohort (n=6298), and mortality has been reported from a combined Swedish, Belgium and Dutch (S/B/D) cohort (n=2543). In the French cohort, there was an increased standardised mortality ratio (SMR) for ‘ill-defined conditions’ (SMR 3.35), bone neoplasms (SMR 5) and cerebrovascular disease (SMR 5.29), while in S/B/D cohort there were no deaths from cancer or vascular disease. In the French study, high GH doses (>50μg/kg/day) were associated with an increased SMR (3.41). These different results illustrate the importance of assembling very large datasets, and this will be addressed in the full SAGhE cohort.

A recent position statement, generated by an international panel of experts, which had evaluated published safety data and current pharma databases, concluded that ‘GH continues to have a good safety record when used for approved indications and at recommended doses’. Importantly the group recommended that long-term pharmacovigilance of all exposed to r-hGH should occur over the years after treatment.

## LS11 How GH treatment changes the phenotype of children with Prader Willi Syndrome

### Anita Hokken-Koelega

#### Erasmus University Medical Center

The most important reason for treating children with Prader-Willi Syndrome (PWS) with growth hormone (GH) is to optimize their body composition.

Setting Longitudinal study to evaluate efficacy and safety data during 8 years of GH treatment, in a Dutch cohort of 60 prepubertal children with genetically proven Prader Willi syndrome, treated with 1 mg GH/m2/day (0.035 mg GH/kg/day). Annual measurements of lean body mass (LBM) and fat mass percentage (%) by the same dual-energy x-ray absorptiometry (DXA) machine in all children.

**Results**: After a significant increase during the first year of GH treatment (P < 0.0001), LBM remained stable for 7 years at a level above baseline (P < 0.0001). After a significant decrease in the first year, fat mass % SDS and body mass index (BMI) SDS remained stable at a level not significantly higher than at baseline (P = 0.06, P = 0.14, resp.). BMI SDS based on PWS references was, however, significantly lower after 8 years of GH treatment than at baseline (P < 0.0001). After 8 years of treatment, height SDS and head circumference SDS had completely normalized. Mean IGF-I SDS increased to + 2.36 SDS during the first year of treatment (P < 0.0001) and remained stable since then. GH treatment did not adversely affect glucose homeostasis, serum lipids, blood pressure and bone maturation.

**Conclusion**: This 8-year study demonstrates that long-term GH treatment is able to change the Prader Willi phenotype, by counteracting the natural course of increasing obesity and abnormal body composition in PWS.

## LS12 Safety and Efficacy of PEG-rhGH in Growth Hormone Deficiency Children: Results of Multicenter Clinical Studies in China

### Pinchas Cohen^1^, Xiaoping Luo^2^

#### ^1^SC Leonard Davis School of Gerontology; ^2^Chinese Society of Pediatric Endocrinology and Metabolism / Department of Pediatrics, Tongji Hospital, Tongji Medical College of Huazhong University of Sciense & Technology

**Introduction**: Jintrolong® (PEG-rhGH) is a long-acting rhGH prepared by conjugating a branched polyethylene glycol molecule to rhGH. A phase-I pharmacokinetics study showed it to be suitable for weekly dosing regimen in children with GHD. A phase-II study established the efficacious dose of weekly PEG-rhGH to be 0.2-mg/kg/wk. Phase III and IV studies were designed to evaluate short-term and long-term efficacy and safety of PEG-rhGH in large scale of children with GHD.

**Methods**: A phase-III, multicenter, randomized, open-label, controlled trial enrolled treatment-naïve, prepubertal GHD children >3 years. 108 patients were randomized in a 2:1 ratio to s.c. injection weekly PEG-rhGH at a dose of 0.2-mg/ kg/wk or to daily rhGH at a dose of 0.25-mg/kg/wk for 25-weeks. The primary endpoint was height velocity (HV) increase at 25 weeks. The phase IV study included 4 sub-studies, that enrolled prepubertal GHD children ≥3 years from 80 hospitals, that were treated for 26-weeks. The study design is showed in figure-1. The primary endpoint was height standard deviation score increase (ΔHtSDS) at 26 weeks. All patients were enrolled to extension studies after 26-weeks with optimal PEG-rhGH dosage until final height.

**Results**: Significantly greater HV increase was associated with PEG-rhGH treatment vs. daily rhGH (P<0.05) at 25 weeks in the phase-III study. Adverse events (AEs) were comparable between two groups. In the phase-IV study, 944 patients from 4 sub-studies completed the 26-week treatment. TheΔHtSDS were comparable between the different dosage PEG-rhGH groups in sub-study-1, and sub-study-2. Although the ΔHtSDS were comparable within bi-weekly PEG- rhGH, weekly PEG-rhGH and daily rhGH in sub-study-3, a borderline significance trend was observed after 39-weeks follow-up between the bi-weekly and weekly PEG-rhGH. In the observational, open-label sub-study-4, the most frequent treatment dosage was weekly PEG-rhGH at 0.15-mg/kg/wk. AEs were comparable between PEG-rhGH and daily rhGH in all 4 sub-studies.

**Conclusions**: PEG-rhGH showed excellent safety and efficacy in GHD children treated with doses from 0.1 to 0.2-mg/kg/wk. Preliminary results from phase IV studies need to be confirmed with additional patients and long-term follow-up.

**Fig. 1 (abstract LS12).**
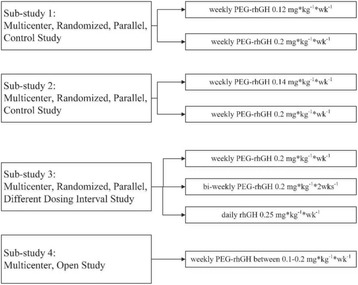
Phase IV study design

## LS13 Moving Towards an International Consensus on the Future of GH therapy

### Pinchas Cohen

#### USC Leonard Davis School of Gerontology

As growth hormone (GH) therapy completes its sixth decade, it has become a staple of endocrine therapy for both children and adults. Thirty years ago, recombinant daily GH replaced pituitary GH therapy that was associated with risk of Jakob-Creutzfeldt Disease. However, as daily rhGH treatment continued to be refined, concerns about its safety (primarily related to cancer risk) lingered, even as large observational phase-IV studies did not demonstrate specific risks. Over the last several years, preliminary results of a retrospective analysis of a large cohort with long term outcomes of GH recipients in Europe (SAGhE) raised serious concerns about the possibility of increased mortality in a French sub-cohort that were related mainly to cardiovascular causes. Analysis of other sub-cohorts including Scandinavian data did not confirm this finding. The international pediatric endocrine community responded to this concern with serious reconsideration of the issues that culminated in a consensus workshop co-sponsored by all of the major relevant societies that recognized some inherent concerns related to GH use, particularly as they relate to second malignancies among cancer survivors, but did not find evidence for elevated cancer risk in other populations and concluded that overall mortality did not appear to be increased when the entire SAGhE population was taken into account (1). These important lessons will be critical in the clinical integration of a new generation of long-acting growth hormone products, with over six different distinct preparations currently being tested in phase-II and –III studies. Another consensus workshop recently held and published, examined the various issues involved in developing this new product class (2). While challenges in optimizing safety and efficacy of these new drugs clearly exist, early data, and a rationale clinical development strategy, make it very likely that several long-acting products will soon be available for the use of the endocrine community, which will enhance physician-patient choices, improve convenience, and potentially overcome adherence and compliance issues, without compromising the excellent safety record of GH.

**References**

1. Allen DB, Backeljauw P, Bidlingmaier M, Biller B, Boguszewski M, Burman P, Butler G, Chihara K, Christiansen JS, Cianfarani S, Clayton PE, Clemmons D, Cohen P, Darendeliler F, Deal C, Dunger DP, Erfurth EM, Fuqua J, Grimberg A, Haymond M, Higham C, Ho KK, Hoffman AR, Hokken-Koelega AC, Johannsson G, Juul A, Kopchick JJ, Lee P, Pollak M, Radovick S, Robison L, Rosenfeld R, Ross RJ, Savendahl L, Saenger P, Toft Sorensen H, Stochholm K, Strasburger CJ, Swerdlow A, Thorner MO. Growth Hormone Safety Workshop Position Paper: a critical appraisal of recombinant human growth hormone therapy in children and adults. Eur J Endocrinol. 2015; 174:1–9.

2. Christiansen JS, Backeljauw P, Bidlingmaier M, Biller B, Boguszewski M, Casanueva FF, Chanson P, Chatelain P, Choong CS, Clemmons DR, Cohen L, Cohen P, Frystyk J, Grimberg A, Hasegawa Y, Haymond M, Ho K, Hoffman AR, Holly JM, Horikawa R, Hoybye C, Jorgensen JO, Johannsson G, Juul A, Katznelson L, Kopchick JJ, Lee KO, Lee KW, Luo XP, Melmed S, Miller B, Misra M, Popović V, Rosenfeld RG, Ross J, Ross RJ, Saenger P, Strasburger CJ, Thorner MO, Werner H, Yuen KC. Growth Hormone Research Society Perspective on the Development of Long- Acting Growth Hormone Preparations. Eur J Endocrinol. 2016; 174:1–9.

## LS14 When one gene does it all: monogenic forms of autoimmune diabetes

### Elisa De Franco

#### Naomi Berrie Fellow in Diabetes Research, Molecular Genetics, University of Exeter Medical School

Neonatal diabetes is diagnosed before 6 months of age and is generally caused by a mutation in a single gene. Traditionally it has been considered as a separate entity from the more common type 1 diabetes, which is generally diagnosed after 6 months and is caused by a combination of genetic predisposition and environmental triggers. Last generation genetic approaches have allowed great advances in the knowledge of the genetic basis of both type 1 and neonatal diabetes, challenging the conventional view of neonatal diabetes and type 1 diabetes as two completely distinct diseases.

Mutations in 23 genes have been identified to date to cause neonatal diabetes, providing a genetic diagnosis for 82% of patients diagnosed before 6 months of age. The majority of these genes are involved in insulin sensing/ secretion and beta cell function/development. A notable exception are mutations in three genes (FOXP3, STAT3, and IL2RA) which cause neonatal diabetes with additional autoimmune features. These three genes are not directly involved in beta cell development or function, in fact they encode for proteins that are important for regulation of the immune system and prevention of autoimmunity. Patients with mutations in these genes have diabetes soon after birth as a result of early autoimmune destruction of their beta cells. Furthermore, these patients generally have low birth weight, suggesting that the autoimmune reaction starts in utero. These patients generally develop additional autoimmune features early in infancy and allogenic stem cell transplantion is currently the only therapeutic option. The Exeter team has been specifically looking for the genetic causes of early onset diabetes and autoimmunity and has currently identified 2 further genes causing neonatal diabetes through immune dysregulation.

But how accurate is the cut-off of 6 months for a differential diagnosis of neonatal diabetes versus type 1 diabetes? We have assessed this using a T1D genetic risk score generated from type 1 diabetes-associated common genetic variants in patients diagnosed before 6 months of age in whom a genetic cause could not be identified. This approach has identified patients who are likely to have early onset type 1 diabetes who were indeed diagnosed before the age of 6 months.

Uncovering the genetic basis of neonatal and type 1 diabetes broadens our understanding of the mechanisms involved in the the pathogenesis of both diseases. Emerging evidence points towards a continuum model for different diabetes subtypes: from monogenic to more common forms of diabetes.

## LS15 African Efe Pygmies: From Anthropology to Endocrinology

### Mitchell E. Geffner

#### Keck School of Medicine of USC, Center for Endocrinology, Diabetes & Metabolism, Children's Hospital Los Angeles

Pygmy populations occupy a vast territory extending west-to-east along the central equatorial African belt from the Congo Basin to Lake Victoria, but are also found in other parts of the world, e.g. , Papua, New Guinea. Their short stature is thought to be adaptive (as opposed to a defect) in terms of ease of locomotion through dense forest understory, minimization of food requirements during cyclical periods of undernutrition, and selection for survival in hot, humid forest environment where greater surface area:body mass ratio is necessary for sufficient thermoregulation. The evolutionary history of the human pygmy phenotype (small body size), a characteristic of African rainforest hunter-gatherers, is largely unknown. Certain genomic regions are significantly associated with the pygmy phenotype in some rainforest hunter-gatherer populations from east central Africa and have multiple attributes that provide supporting evidence of genuine association with the pygmy phenotype, including enrichments for SNPs previously associated with stature variation in Europeans and for genes with growth hormone receptor and regulatory functions consistent with early suggestions that the short stature of pygmies results from GH resistance and an absent adolescent growth spurt. Efe pygmies of northeast Zaire have the shortest mean adult stature of any population on earth. We previously described GH and IGF-I unresponsiveness in T-lymphoblast cell lines derived from Efe pygmies. Using this model, we also found markedly decreased cell surface expression of type 1 IGF receptors (IGF-I receptors) with normal ligand binding affinity. The pygmy IGF-I receptors were not autophosphorylated and did not transmit a signal in response to physiological concentrations of IGF-I. There was a substantially decreased level of IGF-I receptor mRNA in the pygmy cells with a normal mRNA half-life. No consistent mutation was found in the exons encoding the IGF-I receptor. These results indicate decreased IGF-I receptor gene transcription and IGF-I receptor signaling as the primary variation in the pygmy cell lines, with GH resistance a secondary back-up phenomenon. Our findings point to the IGF-I receptor as the locus governing short stature in the African Efe pygmy and suggest that human stature may be genetically controlled by expression of the IGF-I receptor.

## LS16 Skeletal, Growth, and Functional Improvements in Children with Hypophosphatasia Treated with Asfotase Alfa for 5 years

### Jill H Simmons

#### Vanderbilt University School of Medicine

Hypophosphatasia (HPP) is an ultra-rare orphan disease caused by mutation(s) in the ALPL gene resulting in deficiency of tissue-nonspecific alkaline phosphatase (ALP). Disease presentation is variable, ranging from frequently fatal in the perinatal or infantile forms to primarily causing musculoskeletal abnormalities and pain in juveniles and adults. Infantile HPP features poor bone mineralization leading to rickets and is sometimes complicated by seizures, respiratory compromise, poor growth, and delayed motor function. Juvenile HPP features include delayed/ impaired motor function, generalized muscle pain, poor bone mineralization, abnormal gait, and early tooth loss. Historically, HPP patients were treated with surgical management, dental hygiene, respiratory support, and dietary modifications when necessary, but until recently, there was no disease-specific therapy available. Recombinant bone-targeted ALP is available for patients with HPP, and studies have demonstrated benefits upon the perinatal, infantile, and juvenile forms. Asfotase Alfa (AA) is given subcutaneously at a dose of 2 mg/ kg/ day 3 days per week or 1 mg/ kg/ day 6 days per week in Japan.

Five- year survival in perinatal/ infantile HPP patients treated with AA was 84%(n=37) , which was higher than that observed in a historical control group (27%). Changes in HPP-related skeletal disease have been demonstrated, with the Radiographic Global Impression of Change (RGI-C) scale (-3=severe worsening; +3=near/complete healing) improving to +2.7 (+1.3, +3.0) by year 1 (n=9) and continuing at +2.0 (+2.0, +3.0) at year 5 (n=9) Infants treated with AA also have significant improvement in respiratory status, growth and motor skills development. Thirteen 6-12 year-old childhood-onset HPP patients treated with AA for 5 years were compared with 16 untreated pediatric historical controls with HPP. Improvements were seen in RGI-C scale of treated patients by 6 weeks [+1 (0.0, +2.0), p=0.001] and persisted through 5 years [+2.2 (+1.7, +2.7), p=0.0005]; no change was seen in the RGI-C scale of historical controls. In the treated group, improvements occurred in weight and height z-scores and developmental, pain, and disability assessments.

In all patient populations studied, serious adverse events have been rare but injection site reactions (ISR) are common and include erythema, warmth, and lipohypertrophy at injection sites. ISRs and AES have not led to medication discontinuation.

In conclusion, AA enzyme replacement therapy has been demonstrated to improve survival, skeletal mineralization, growth, and motor skills in children with HPP. Side effects have been minimal and have not led to drug discontinuation. Follow-up for long-term effects needs to continue.

## ES1-1 Total management of Rickets: causes, prevention and treatment

### Global Consensus Recommendations on Prevention and Management of Nutritional Rickets: Definition and Diagnosis

#### Lorna Ramos-Abad

##### University of the Philippines College of Medicine, Philippine General Hospital Metro Manila

Nutritional Rickets (NR), a disorder of defective chondrocyte differentia- tion and mineralization of the growth plate and defective osteoid mineralization, is caused by vitamin D deficiency and/or low calcium intake in children. Osteo- malacia is abnormal matrix mineralization in established bone, and although present in children with rickets, it is used to describe bone mineralization defects after com- pletion of growth.

Diagnosis of NR is made on the basis of history, physical examination, and biochemical testing and is confirmed by radiographs. Osseous signs and symptoms include: swelling wrists and ankles Delayed fontanelle closure (normally closed by the age of 2 years) Delayed tooth eruption (no incisors by the age of 10 months, no molars by 18 months), Leg deformity (genu varum, genu valgum, windswept deformity) Rachitic rosary (enlarged costochondral joints – felt anteriorly, lateral to the nipple line) Frontal bossing Craniotabes (softening of skull bones, usually evident on palpation of cranial sutures in the first 3 months) Bone pain, restlessness, and irritability. Radiographic features are: splaying, fraying, cupping, and coarse trabecular pattern of metaphyses, widening of the growth plate, Osteopenia. There could be nonosseous features such as Hypocalcemic seizure and tetany, Hypocalcemic dilated cardiomyopathy (heart failure, arrhythmia, cardiac arrest, death) Failure to thrive and poor linear growth, delayed gross motor development with muscle weakness, raised intracranial pressure.

Biochemical testing alone is not sufficient to diagnose NR and may not differentiate whether the primary cause of NR is vitamin D or dietary calcium deficiency because combined deficiencies are common. Vitamin D status is assessed by measuring blood levels of total 25OHD. Classification of vitamin D status is based on serum 25-hydroxyvitamin as: sufficiency, >50 nmol/l , Insufficiency, 30–50 nmol/l Deficiency, <30 nmol/l .

## ES1-2 Total management of Rickets: causes, prevention and treatment

### Global Consensus Recommendations on Prevention and Management of Nutritional Rickets

#### Navoda Atapattu

##### Consultant Paediatric Endocrinologist

Nutritional rickets (NR) has been increasingly reported in high and low income countries. Resurgence of NR has been observed in the high income countries due to increase in immigrants and refugee groups. There is a considerable variation in the definition, diagnosis and management of NR. European Society for Pediatric Endocrinology decided to formulate evidence-based recommendations together with the PES, JSPE, SLEP, APEG, ISPAE, ASPAE, CSPEM, ESPGHAN. The consensus paper includes evidence up to the end of 2014.

Nutritional rickets is defined as defective chondrocyte differentiation and mineralization of growth plate due to vitamin D deficiency and or low calcium intake. Defective mineralization results in growth plate widening, metaphyseal cupping and fraying seen on the radiography. Vitamin D status is assessed by measuring blood levels of total 25 OH vitamin D. Dietary calcium deficiency is diagnosed by obtaining a calcium intake history with the help of dietary calcium intake questionnaire specific to their country/region. Vitamin D level of >50 nmol/ l is considered sufficient, 30-50 nmol/l is considered as insufficient and < 30 nmol/L is deficient and >250nmol/ l with hypercalciuria is considered as vitamin D toxicity. It is recommended to provide 400IU of vitamin D daily for all infants from birth irrespective of mode of feeding and 600IU is recommended for children and adults through diet or supplementation. In countries where food fortification is not a routine practice, vitamin D supplementation is needed for children with a history of vitamin D deficiency, children and adult at risk of vitamin D deficiency and pregnant women. Minimal dose of vitamin D to treat rickets is 2000IU daily for 3 months. Oral route of vitamin D is recommended. Calcium 500mg as dietary intake or supplement is routinely needed together with vitamin D in addition to vitamin D supplement. Complementary feeding rich in calcium should be started no later than 26 weeks to prevent rickets. Population based studies should determine the burden of rickets and screening should be done based on clinical features. NR can have a major impact on the health of infants, children, and adolescents. It is important to recognize NR as a preventable global public health problem and implement rickets preventing programs by adhering recommended vitamin D and calcium intakes, monitoring for adherence and promoting staple food fortification programs.

## ES1-3 Total management of Rickets: causes, prevention and treatment

### Hypophosphatemic Rickets: Current Status and Future Perspectives

#### Toshimi Michigami

##### Department of Bone and Mineral Metabolism, Osaka Medical Center and Research Institute for Maternal and Child Health, 840 Murodo-cho, Izumi, Osaka Japan

Phosphate is a component of hydroxyapatite and is indispensable for skeletal mineralization. Prolonged deficiency of phosphate can be caused by impaired intestinal absorption and/or renal phosphate wasting, resulting in rickets in children and osteomalacia in adults. Although phosphate is abundant in food, patients with malnutrition or malabsorption may have phosphate deficiency, leading to hypophosphatemic rickets. Renal phosphate wasting can be observed in the conditions with impaired function of renal proximal tubules, such as Fanconi syndrome and some forms of renal tubular acidosis. Renal reabsorption of phosphate is mostly mediated by type 2a and 2c sodium-phosphate co-transporters (NPT2a and NPT2c) in the proximal tubules. Loss-of-function mutations in SLC34A3 encoding NPT2c cause hereditary hypophosphatemic rickets with hypercalciuria (HHRH).

Several kinds of hypophosphatemic rickets and osteomalacia are caused by excessive actions of fibroblast growth factor 23 (FGF23). FGF23 is produced mainly by osteocytes in the bone and exerts its effects on the distant organs. In the kidney, FGF23 increases renal phosphate excretion by suppressing the expression of NPT2a and NPT2c. In addition, FGF23 reduces the level of 1,25-dihydroxyvitamin D. FGF23-related hypophosphatemic rickets/ osteomalacia is characterized by renal phosphate wasting, hypophosphatemia, and inappropriately low level of serum 1,25(OH)2D. Mutations in FGF23 that make the protein resistant to proteolytic cleavage cause autosomal dominant hypophosphatemic rickets (ADHR). X-linked hypophosphatemic rickets (XLH), the most common form of FGF23-related hereditary hypophosphatemic rickets, is caused by inactivating mutations in the phosphate- regulating gene homologous to endopeptidase on X chromosome (PHEX). Genetic evidence has revealed that loss- of-function mutations in dentin matrix protein 1 (DMP1), ectonucleotide pyrophosphatase phosphodiesterase-1 (ENPP1) , and family with sequence similarity 20, member C (FAM20C) lead to FGF23-related hypophosphatemic rickets/osteomalacia of autosomal recessive inheritance. Other conditions such as McCune-Albright syndrome, linear nevus sebaceous syndrome, and tumor-induced osteomalacia, are also associated with FGF23-related hypophosphatemia. Measurement of serum FGF23 levels is useful to distinguish FGF23-related hypophosphatemia from rickets or osteomalacia of other causes, because they are rather low in vitamin D deficiency and intrinsic defects in renal proximal tubules. Patients with XLH are currently treated with active vitamin D and phosphorus. However, the administration of active vitamin D and phosphorus further increases the level of FGF23, which might worsen the disease. A neutralizing antibody against FGF23 has been developed recently as a new therapy for XLH, and clinical trials are currently undergoing.

## ES2 Adrenarche

### Adrenarche

#### Joseph Majzoub

##### Royal Children's Hospital

Adrenarche is the initiation of dehydroepiandrosterone (DHEA) and DHEA-sulfate (DHEAS) secretion from the zona reticularis of the adrenal gland during childhood. Adrenarche is a gradual process beginning during the first five years of life, whereas pubarche, the appearance of pubic hair and an end result of adrenarche, normally occurs when children are eight or more years of age. Premature adrenarche is among the most common pediatric endocrine disorders, usually considered benign but occasionally the first sign of an adrenal tumor or enzyme deficiency. In some children, premature adrenarche may be a forerunner of the polycystic ovary syndrome or the metabolic syndrome. The trigger for adrenarche remains unknown, and initiating factors leading to production of adrenal androgens are poorly defined.

**Learning objectives**: As a result of participating in this session, learners should be able:

- To understand the changes that occur in 17, 20 lyase and 3 β HSD2 enzyme activities during adrenarche
- To understand changes in the zona reticularis during adrenarche
- To understand how to identify true, genetic 3 β HSD2 deficiency
- To understand whether mild, nonclassical 3 β HSD2 exists
- To discuss the possible role of intracrine, intra-adrenal cortisol in the onset of adrenarche
- To speculate on why benign premature adrenarche is associated with obesity

## Kaichi-Kida 1 Thymic deletion of ICA69 induces autoimmune diabetes and other endocrine diseases

### Asako Tajima^1,2^, Ichiro Miyata^1^, Hiroyuki Ida^1^, Massimo Trucco^2^, Yong Fan^2^

#### ^1^Department of Pediatrics, Jikei University School of Medicine; ^2^Allegheny Health Network, Institute of Cellular Therapeutics

**Aim**: Islet autoantigen 69 (ICA69), encoded by the Ica1 gene, is one of the known autoantigens in type 1 diabetes (T1D). In addition to pancreatic beta cells, ICA69 is also present in extrapancreatic endocrine and exocrine tissues, such as the thyroid and salivary gland. Notably, ICA69 is ectopically expressed in the thymus, and its level of expression is significantly decreased in T1D prone NOD mice, compared to that of T1D resistant C57BL/6 mice. Immunization with ICA69 peptides in NOD mice not only accelerates diabetes progression, but also induces autoimmune response in the salivary glands. These results highlight the antigenic role of ICA69 in the progression of these diseases and suggest that thymic ICA69 expression is essential to establish immune tolerance of ICA69-expressing organs. In this study we attempted to elucidate the underlying mechanism of ICA69 and its role in the induction of autoimmunity in the pancreas and other endocrine tissues.

**Materials and Methods**: C57BL/6 mice with systemic Ica1 gene deficiency (B6.ICAdel/wt for heterozygous and B6.ICAdel/del for homozygous ICA69 deletion) were first generated to observe the spontaneous effect of ICA69 in the endocrine tissues. Next, ICA69 was eliminated specifically in the thymus using the Cre-lox system (Aire-ICA69). Endocrine functions were assessed by intraperitoneal glucose tolerance tests and saliva flow rate. Flow cytometry, Enzyme-Linked ImmunoSpot assay, and immunohistochemistry were performed to investigate the potential link between the level of thymic ICA69 expression and peripheral anti-ICA69 autoimmunity.

**Results**: Significant decrease of thymic ICA69 expression was observed in B6.ICAdel/wt mice. When immunized with ICA69 peptides, B6.ICAdel/wt mice develop both insulitis and thyroiditis. Consistently, Aire-ICA69 mice displayed an impaired glucose tolerance as early as 12 weeks of age. In addition to insulitis, immunohistochemical analyses revealed the spontaneous development of autoimmunity in multiple extrapancreatic organs, including sialadenitis, thyroiditis, and gastritis.

**Conclusion**: Our findings establish a direct link between compromised thymic ICA69 expression and autoimmunity against multiple ICA69- expressing organs, and identify a potential mechanism for the development of T1D-associated autoimmune disorders, as well as other multi- organ diseases, such as autoimmune polyglandular syndromes 2 and 3.

## Kaichi-Kida 2 DNA methylation defects in short children born small for gestational age

### Akie Nakamura^1^, Takanobu Inoue^1^, Keiko Matsubara^1^, Shinichiro Sano^2^, Yasuhiro Naiki^3^, Shuichi Yatsuga^4^, Junko Nishioka^4^, Keisuke Nagasaki^5^, Koji Muroya^6^, Sachiko Kitanaka^7^, Toshihiro Tajima^8^, Reiko Horikawa^3^, Tsutomu Ogata^2^, Maki Fukami^1^, Masayo Kagami^1^

#### ^1^Department of Moledular Endocrinology, National Research Institute of Child Health and Development; ^2^Department of Pediatrics, Hamamatsu University School of Medicine; ^3^Division of Endocrinology and Metabolism, National Medical Center for Children and Mothers; ^4^Department of Pediatrics and Child Health, Kurume University School of Medicine; ^5^Department of Homeostatic Regulation and Development, Niigata University Graduate School of Medical and Dental Sciences; ^6^Division of Pediatrics; ^7^Department of Endocrinology and Metabolism, Kanagawa Children's Medical Center; ^8^Department of Pediatrics, The University of Tokyo; ^9^Department of Pediatrics, Jichi Children's Medical Center Tochigi

**Background**: DNA methylation defects at differentially methylated regions (DMRs) of imprinted loci are one of the cause of intrauterine growth restriction (IUGR) and postnatal growth failure. Among eight known imprinting disorders, Silver-Russell syndrome (SRS), Temple syndrome and Prader-Willi syndrome patients show pre- and postnatal growth retardation. Furthermore, maternal uniparental disomy of chromosome 6 (UPD(6)mat) and 20 (UPD(20)mat) have also been associated with growth failure. However, there is no single report of systematic methylation analysis of a large cohort of patients with IUGR and postnatal growth failure. In this study, we aimed to clarify the frequency of methylation defects in short children born small for gestational age (SGA).

**Participants**: A total of 183 short children (height < -2.0 SD after the age of 2 years or before growth hormone treatment) born SGA were recruited in this study. Inclusion criteria of SGA were defined as birth weight and birth length SDS less than 10th percentile for gestational age. Patients with apparent chromosomal or monogenic abnormalities which induce IUGR were excluded. 28 patients were classified as ‘Likely-SRS’ according to the Nechine-Haribison clinical scoring system.

**Methods**: We performed quantitative DNA methylation analysis at DMRs of 9 imprinted loci (PLAGL1 , PEG1 , PEG10 , H19 , Kv, DLK1-MEG3 intergenic (IG), MEG3 , SNRPN and GNAS A/B) by pyrosequencing using genomic DNA isolated from peripheral blood leukocytes. When we detected methylation defects, we carried out further studies, such as comparative genomic hybridization, multiplex-ligation dependent probe amplification and microsatellite analysis, to determine the genetic causes of methylation abnormalities (microscopic chromosomal rearrangement, UPD or epimutation).

**Results**: We identified loss of methylation (LOM) at H19 -DMR in 19 cases, UPD(7)mat in 6 cases, LOM at IG-DMR and MEG3 -DMR in one case, UPD(14)mat in 5 cases, UPD(15)mat in one case, UPD(6)mat in 3 cases and UPD(20)mat in 2 cases. In addition, we detected one case with partial LOM at IG-DMR and MEG3 -DMR due to trisomy 14 mosaicism with UPD(14)mat, and another case with partial LOM at A/B DMR due to 20pter microdeletion and 20qter microduplication.

**Conclusions**: Methylation defects were detected in 39 of 183 (21%) short children born SGA. Our results highlight the clinical importance of methylation defects as genetic causes of short stature born SGA.

## Kaichi-Kida 3 The gut microbiome and insulin resistance in children born very preterm

### Valentina Chiavaroli^1,2^, Thilini N Jayasinghe^1^, Sachin Jayan^1^, Cameron Ekblad^1^, Jose’ Derraik^1^, Paul Hofman^1,2^, Elizabeth McKenzie^1^, Justin O'Sullivan^1,2^, Wayne Cutfield^1,2^

#### ^1^University of Auckland, Liggins Institute; ^2^Gravida: National Centre for Growth and Development

**Background**: In recent years the gut microbiome has been shown to influence the development of obesity and type 2 diabetes mellitus. We hypothesize that adverse early life events in preterm children may lead to alterations in the gut microbiome, which contribute to later metabolic disease. Children born preterm are at increased risk for insulin resistance, obesity, and cardiovascular disease.

**Aim**: To correlate the metabolic phenotype with the gut microbiome composition and functional capacity in children born very preterm compared to those born at term.

**Results**: Participants were healthy prepubertal children aged 5 to 11 years born very preterm (-4 · min-1(mU/l); p=0.0007). Stool metatranscriptomics identified Coriobacteriaceae and Collinsella species as being associated with children who were born preterm (highly significant with LDA score >4.0, LefSe). There were also functional changes in the activity of the microbiome in children born preterm, including glutamate and arginine metabolism, known to be involved in glucose metabolism. Changes to the metabolites within the preterm fecal and plasma correlated with the observed phenotypes.

**Conclusion**: Children born very preterm are insulin resistant with differences in gut microbiome species and activity. In preterm children we speculate that (i) these changes in the gut microbiome were established in early infancy, and (ii) the altered gut microbiome contributes to insulin resistance.

## Kaichi-Kida 4 Genetic etiology study of 90 complete growth hormone deficiency patients by targeted next-generation sequencing

### Jia-Tong Hou^1^, Shi-Yao Wang^1^, Han-Ze Du^1^, Hui-Juan Zhu^1^, Ci-Ren A-San^2^, Hong-Bo Yang^1^, Lin-Jie Wang^1^, Feng-Yng Gong^1^, Hui Pan^1^

#### ^1^Department of Endocrinology, Key Laboratory of Endocrinology of Ministry of Health, Peking Union Medical College Hospital, Chinese Academy of Medical Sciences & Peking Union Medical College; ^2^Binhai Genomics Institute, BGI-Tianjin, Tianjin Enterprise Key Laboratory of Clinical Molecular Diagnostic

**Objectives**: This study aimed to discover the genetic mutations and identify the etiologies in a cohort of complete growth hormone deficiency patients, and to establish a new genetic testing method—targeted next-generation sequencing (NGS) in genetic evaluation of complete growth hormone deficiency and verify its potential utility.

**Methods**: A total of 90 complete growth hormone deficiency patients were included in the study. Twenty-three genes related to growth hormone deficiency were sequenced parallel by the combination of NGS and targeted genomic enrichments. The clinical significances of variants detected in patients were evaluated on the basis of the extant clinical guideline.

**Results**: These patients were divided into two categories based on clinical diagnosis, including 56 with idiopathic growth hormone deficiency (IGHD) and 34 with multiple pituitary hormone deficiencies (MPHD). We identified 20 gene variants as the possible genetic causes for 18 (20%) of the 90 growth hormone deficiency patients, which included 12(66.7%) of IGHD patients and 8 (33.3%) of MPHD patients. Of the variants detected, 6 were previously reported and the others were not reported. Of the 12 IGHD patients with possible genetic causes, 4 were identified with CHD7 mutation, 2 with GHSR mutation, 2 with VPS13B mutation, 1 with ALMS1 mutation, 1 with SEMA3E mutation, 1 with a novel homozygous deletion in chromosome 17: 61994532- 61996228 of the whole GH1 gene and 1 with a novel heterozygous duplication in chr3 F57231913-57234310 of the whole HESX1 gene. Among the 6 MPHD patients with possible genetic causes, 2 were identified with GLI2 mutation, 3 with SOX3 mutation, 1 with LHX4 mutation and 1 with a novel homozygous deletion in chromosome 17: 61994532- 61996228 of the whole GH1 gene.

**Conclusions**: The method of combination of NGS and targeted genomic enrichments is able to explain 20% of the molecular etiologies of the GHD patients. This study confirmed the high efficiency and precise of such a comprehensive genetic evaluation in clinics. It provides a good way to a large-scale genetic study of GHD. Part of patients with pathogenic gene mutations may not show the corresponding phenotypes. Different patients with the same genetic mutation can lead to variable phenotypes.

**Key words**: growth hormone deficiency, multiple pituitary hormone deficiency, genetic etiology, next-generation sequencing Ctargeted genomic enrichments.v

## Kaichi-Kida 5 Diagnostic Application of Targeted Exome Sequencing for Skeletal Dysplasia

### Sung Yoon Cho^1^, Seok Bae^3^, Nayoung K. D. Kim^3^, Ok-Hwa Kim^4^, Tae Joon Cho^5^, Sung Won Park^6^, Young Bae Sohn^7^, Woong-Yang Park^3^, Dong-Kyu Jin^1^

#### ^1^Department of Pediatics, Samsung Medical Center; ^2^Department of Pediatics, Myongji Hospital; ^3^Samsung Genome Institute, Samsung Medical Center; ^4^Department of Radiology, Woorisoa Children's Hospital; ^5^Department of Pediatric Orthopaedics, Seoul National University Childrens Hospital; ^6^Department of Pediatics, Cheil General Hospital & Womans’ Health Care Center; ^7^Department of Medical Genetics, Ajou University Hospital

Identification of causative genes for skeletal dysplasia (SD) is important to provide an exact diagnosis and decide treatment modalities and to counsel the patients. Due to the genetic heterogeneity in SD, the high throughput method can be adapted for the efficient diagnosis. To this end, a new diagnostic pipeline were designed to screen 260 reported candidate genes for SD. A total of 22 patients diagnosed with skeletal dysplasia, or suspected to have it were recruited over one year. Their clinical diagnosis were hypochondroplasia, osteogenesis imperfect type I, Sticker syndrome, pseudohypoparathyroidism, osteopetrosis. pycnodysostosis, spondyloepiphyseal dysplasia strudwick type, and ischiospinal dysostosis. We applied targeted exome sequencing (TES) on 260 genes in the 22 probands with SD. Potential causative variants determined using TES were confirmed by filtering steps. These variants were then filtered out with clinical features, inheritance pattern and a co-segregation study in the family and allele frequency in normal 197 control subjects. Finally, we detected 18 causative variants, and these variants were in the FGFR3, GANS, COL1A1, COL1A2, COL2A1, LRP5, CLCN7, CTSK, BMPER, and TNFSF11 genes bringing the total detection rate in all the patients to be 68.2% (15/22). These variants were validated using Sanger sequencing. Despite the advent of whole genome and whole exome sequencing, we propose TES as a screening and diagnostic tool at least for SD to find mutations based upon its efficacy and cost-effectiveness.

## O1-1 Diabetes Mellitus is Associated with Intrauterine Hyperglycemia regardless of Maternal Obesity

### Won Im Cho^1^, Hye Rim Chung^1^, Hak C. Jang^2^, Joon-Seok Hong^3^, Jung Sub Lim^4^, Min Jae Kang^5^, Young Ah Lee^6^, Choong Ho Shin^6^, Sei Won Yang^6^

#### ^1^Division of Endocrinology and Metabolism / Department of Pediatrics, Seoul National University Bundang Hospital; ^2^Division of Endocrinology / Department of Internal Medicine, Seoul National University Bundang Hospital; ^3^Department of Obstetrics and Gynecology, Seoul National University Bundang Hospital; ^4^Department of Pediatrics, Korea Cancer Center; ^5^Department of Pediatrics, Hallym University Sacred Heart Hospital; ^6^Department of Pediatrics, Seoul National University Children's Hospital

**Background**: It has not been conclusively established whether intrauterine hyperglycemia affects prepubertal offspring obesity regardless of maternal obesity. This study evaluates the effect of maternal obesity and hyperglycemia on the fat mass (FM) of prepubertal offspring of mothers with gestational diabetes mellitus (OGDM).

**Methods**: FM was determined by dual-energy X-ray absorptiometry in 22 OGDM aged 5-6 years, and was compared with that of 49 age- matched offspring of normoglycemic mothers. The relationship between FM and the results of a 100 g oral glucose tolerance test (OGTT) during the second trimester was analyzed in OGDM.

**Results**: FM was higher (3,548 g vs. 2,245 g) and lean mass was lower (15,609 g vs. 16,941 g) in OGDM compared with control subjects, respectively, whereas body mass index (BMI) did not significantly differ between the two groups. FM and truncal FM positively correlated with glucose levels, especially 0 hour glucose level (r = 0.580 and *P* = 0.005, *r* = 0.655 and *P* = 0.001, respectively). After adjusting for maternal prepregnancy BMI, total FM (*r* = 0.535, *P* = 0.012) and truncal FM (*r* = 0.535, *P* = 0.003) of OGDM correlated with maternal 0 h glucose levels of the 100 g OGTT.

**Conclusions**: Increased FM of OGDM aged 5-6 years is associated with intrauterine hyperglycemia regardless of maternal obesity.

## O1-2 Relationship of lower heart rate variability with risk for metabolic syndrome in patients with childhood-onset craniopharyngioma

### Hae Woon Jung^2^, Young Ah Lee^1^, Hwa Young Kim^3^, Gyung Min Lee^4^, Ji Young Kim^1^, So Youn Kim^1^, Kyung A Jeong^1^, Keun Hee Choi^1^, Jung-Eun Cheon^5^, In-One Kim^5^, Choong Ho Shin^1^, Sei Won Yang^1^

#### ^1^Department of Pediatrics, Seoul National University College of Medicine; ^2^Department of Pediatrics, Kyung Hee University Medical Center, Department of Pediatrics; ^3^Kangwon National University Hospital; ^4^Department of Radiology, Konyang University Hospital, Department of Pediatrics; ^5^Seoul National University College of Medicine

**Objective**: Autonomic nervous system (ANS) dysfunction with hypothalamic involvement (HI) is implicated in the development of obesity and metabolic complications. In patients treated for childhood onset craniopharyngioma, we investigated changes in ANS activity according to the extent of HI and presence of obesity, using measures of heart rate variability (HRV). Risks for being metabolically unhealthy were analyzed in relation to HRV changes.

**Methods**: From March 2014 to January 2016, HRV indices of overall variability [standard deviation NN interval (SDNN) and total power (TP)], parasympathetic modulation [root mean square of successive RR interval differences (RMSSD) and high frequency (HF)], and sympathetic or sympathovagal modulation [low frequency (LF) and LF/HF ratio] were measured in 48 patients (28 males) aged 10-30 years with HI after craniopharyngioma treatment at Seoul National University Children’s Hospital. The extent of HI was graded on magnetic resonance imaging. Anthropometric measurements, fasting glucose, insulin, lipid panel, and blood pressure were obtained.

**Results**: Patients with extensive HI showed increased BMI z-scores (*P* = 0.008), waist circumference (*P* = 0.037) and insulin resistance (homeostasis model assessment of insulin resistance, HOMA-IR, *P* = 0.043). HRV indices were decreased with extensive HI, but showed no differences between obese and non-obese. SDNN, TP, RMSSD, and LF were all decreased with extensive HI. Obese patients with concomitantly reduced overall variability (by SDNN or TP) showed increased HOMA-IR (*P* < 0.05, for both), triglycerides (*P* < 0.05, for both), blood pressure (*P* < 0.05, for both), and decreased HDL cholesterol (*P* < 0.05, for both). Risk of being metabolically unhealthy was increased in patients with both obesity and reduced overall variability (*P* < 0.05, for both).

**Conclusion**: Extensive HI is associated with obesity as well as decreases in overall variability and parasympathetic modulation. Obese patients with concomitant reduced HRV had higher risk of being metabolically unhealthy.

## O1-3 Relationship between Asymmetric dimethylarginine in Umbilical Cord Plasma and Birth Weight Follows a U-shaped Curve

### Junji Takaya^1,2^, Yuko Tanabe^2^, Yuichi Kuroyanagi^2^, Kazunari Kaneko^2^

#### ^1^Department of Pediatrics, Kawachi General Hospital, Department of Pediatrics; ^2^Kansai Medical University

**Objective**: Placental transport of nutrients is dependent on vascular development, which determines blood flow to the placenta. The ability of the uterine artery to dilate during pregnancy may be specifically related to upregulation of multiple pathways for production of nitric oxide (NO). Asymmetric dimethylarginine (ADMA), an L-arginine analog and a nonselective NO synthase inhibitor is associated with cardiovascular and metabolic disorders. In addition, epidemiological studies confirm that the relationships between human birth weight and adult obesity, hypertension, or insulin resistance follow U-shaped curves. We hypothesized that increased ADMA and decreased NO might underlie the initial pathophysiologic events leading to insulin resistance. To test the hypothesis, the relationship between birth weight and ADMA or nitric oxide parameters was evaluated.

**Methods**: The study group consisted of 41 singleton subjects with gestational ages ranging from 36-41 wk, and birth weights ranging from 1,798-3,822 g. The subjects were divided into 9 infants with small for gestational age (SGA) and 32 with appropriate for gestational age (AGA). Their cord plasma ADMA, insulin, insulin-like growth factor-1(IGF-1), and adipocytokine levels were determined using enzyme-linked immunosorbent assays. The plasma NOX [nitrite (NO2-)+nitrate (NO3-)] levels were measured using the colorimetric assay.

**Results**: The relationship between birth weight and ADMA levels followed a U-shaped curve rather than inverse linear associations expected over a full range of birth weight distribution. ADMA positively correlated with birth weight in AGA group (*p*< 0.001, R=0.590), and inversely correlated with birth weight in SGA group (*p*< 0.05, R=-0.741). Plasma ADMA was inversely correlated with adiponectin (*p*< 0.05, R=-0.289) and quantitative insulin sensitivity check index (QUICKI) (*p*< 0.05, R=-0.294) in all subjects, while not correlated with NOx. Plasma glucose, ADMA, NOx, and resistin concentrations did not differ significantly between SGA and AGA group. However, plasma insulin, IGF-1, leptin, adiponectin and QUICKI were lower in the SGA group than in the AGA group.

**Conclusions**: Plasma ADMA levels in cord blood might be an early marker of fetal growth and insulin resistance. High plasma ADMA levels may represent prenatal programming of insulin resistance, and may be used as a predictor of adult diseases. An analysis of all of these reports would generate that the relationship between birth weight and these metabolic abnormalities in adult life can be described by a U-shaped curve. Our results indicate a possible role of ADMA in fetal life for future disorders characterized by insulin resistance.

## O1-4 The Effect of Sfrp5, Wnt5a, Adiponectin, and Chemerin on Blood Pressure Regulation in Obese Children

### Yan C Yin

#### The Second Affiliated Hospital of Xi'an Jiaotong University, Pediatric

The aim was to evaluate the associations of Sfrp5 and Wnt5a with blood pressure (BP), and to examine whether BP can be influenced by changes in adipocytokines, such as Sfrp5, adiponectin, chemerin, and hsCRP, in obese children after lifestyle intervention. A cross-sectional study was conducted in 263 obese children. In addition, a 6-month lifestyle intervention was performed in a subgroup of 89 obese children with hypertension. Anthropometric parameters, clinical data, adiponectin, chemerin, Sfrp5, and Wnt5a were measured at baseline and after lifestyle intervention. Sfrp5 and adiponectin levels were significantly lower in obese children with hypertension, but Wnt5a, hsCRP, and chemerin levels were elevated in obese children with hypertension. In multivariable linear regression analysis, Sfrp5, Wnt5a, adiponectin, chemerin, and hsCRP were associated with both standard deviation score-systolic blood pressure (SDS-SBP) and -diastolic blood pressure (SDS-DBP). Lifestyle intervention resulted in a significant improvement in BP and weight loss. These were accompanied by significant decreases in hsCRP and chemerin, and significant increases in Sfrp5 and adiponectin, whereas Wnt5a was not changed. Furthermore, the changes in Sfrp5, adiponectin, chemerin, and hsCRP act as partial mediators of the relationship between weight loss and BP reduction. Although Sfrp5 and Wnt5a levels correlated with BP at baseline, after lifestyle intervention, Sfrp5 is more sensitive to reduction in BMI and BP compared to Wnt5a, and the relationship between weight loss and BP reduction were partially mediated by changes in Sfrp5. So we speculate if Sfrp5 and Wnt5a each play a role in regulating BP, it must be different roles.

## O1-5 The association of HLA-DR, DQ genotypes and CTLA4 polymorphisms with a presence of thyroid antibody in Japanese children with Type 1A diabetes

### Tadayuki Ayabe, Misako Okuno, Tomoyuki Kawamura, Tokuo Mukai, Takahiro Mochizuki, Shouji Nakayama, Emiko Tachikawa, Yasusada Kawada, Ichiro Yokota, Shigetaka Sugihara

#### Japanese Study Group of Insulin Therapy for Childhood and Adolescent Diabetes (JSGIT)

**Introduction**: We have made it clear that among Japanese children with Type 1A diabetes (T1AD), the thyroid autoantibody (T-Ab) positive rate is 26.6%, higher in females, and rises depending on age in females. The aim of our study was to clear the genetic factors which associate with a high risk of T-Ab possession in Japanese pediatric T1AD.

**Subjects and Methods**: HLA-DRB1 and DQB1 were analyzed for the registered 909 T1AD patients (the average age at the time of the registration was 12 years and 5 months old and 217 cases had T-Ab) in the Japanese Study Group of Insulin Therapy for Childhood and Adolescent Diabetes, and the relation with the T-Ab (someone with more than one of the anti-thyroid peroxidase antibody and the anti- thyroglobulin antibody) was considered. CTLA4 rs231775 and rs3087243 polymorphisms were analyzed for 905 T1AD patients and compared with 455 healthy controls. Then, the influence on T-Ab possession of CTLA4 SNPs for 905 T1AD patients was considered. The contribution to the T-Ab possession was considered by using logistic regression analysis, making these genotypes (HLA-DRB1, DQB1, and CTLA4 SNPs), gender and age explanatory variables.

**Results**: The susceptible HLA-DRB1 and DQB1 alleles, haplotypes and genotypes to T1AD didn’t associate with the risk of T-Ab possession. In pediatric T1AD, CTLA4 rs231775 had a significantly high frequency of G allele compared with controls regardless T-Ab presence (OR: 1.56, *p*<0.001) and rs3087243 had a significantly high frequency of G allele compared with controls only in T-Ab positive T1AD (OR: 1.87, *p*<0.001). 2 SNPs of CTLA4 had a significantly high frequencies of G allele compared with T-Ab negative T1AD in T-Ab positive T1AD. The age (OR: 3.55~4.98), the sex (OR: 2.11) and CTLA4 rs3087243 (OR: 1.63) were statistically significant effects on the risk of the T-Ab possession with logistic analysis.

**Conclusions**: This study newly demonstrated CTLA4 rs231775 conferred susceptibility to T1AD regardless T-Ab presence, and females with allele G of CTLA4 rs3087243 were at high risk to possess T-Ab in the age dependence in Japanese pediatric T1AD.

## O1-6 The Protective Effects of Adenovirus-mediated IL-10 Gene and Anti-CD20 Monoclonal Antibody on the Pancreatic β Cells of NOD Mice in the Early Stage of Natural T1D Onset

### Li Tang^1,2^, Li Cheng^2^, Fan Lei^2^, Tian Fei^2^, Tang Aiping^2^

#### ^1^Division of Endocrinology and Metabolism, QingDao Women and Children's Hospital1; ^2^Department of Pediatricsm QingDao University

**Objective**: To investigate the protective effects of Adenovirus-mediated IL-10 gene and anti-CD20 monoclonal antibody (mAbs) on the pancreatic β cells in nonobese diabetes (NOD) mice with type 1 diabetes mellitus (T1D) at early stage.

**Methods**: 35 female NOD mice at onset of diabetes and aged 17–20 weeks old were randomly divided into 5 groups. Mouse 1,2,3,4 and 5 groups were intravenously injected 500ug of anti-CD20 mAbs, 500ug of anti-CD20 mAbs combined with 100ul of Ad-IL-10, 100ul of Ad- IL-10, 100ul of Ad-GFP, 100ul of normal saline respectively. All mice were monitored for blood glycose everyday, and sacrificed 9 weeks after injection. Their serum levels of C-peptide were measured and the degree of insulitis were observed. Apoptosis related gene and protein were detected.

**Results**: (1) The blood glucose level of mice in group 2 was reduced. (2) A majority of the insulitis was grade 0-1 in group 2, and grade 2-3 in group 5. (3) The apoptosis rate of pancreatic β cells was significantly lower in group 2 than that in the group 3,4,5, p<0.05. (4) Immunohistochemistry indicated that IL-10 could be highly expressed locally in the pancreatic islets. (5) The results of qPCR and western blot showed that combined intervention exerted protective effects on pancreatic β cells through activating Bcl-2 anti-apoptosis pathway, inhibiting the expression of TNF- α and Fas, and blocking caspase-8--caspase-3 as well as caspase-9--caspase-3 apoptosis pathways.

**Conclusion**: Combined intervention could reduce the apoptosis of pancreatic β cells of NOD mice in the early stage of T1D, and had certain protective effects on the residual pancreatic β cell function.

## O2-1 Clinical outcome and mutation spectrum of the StAR gene in patients with congenital lipoid adrenal hyperplasia

### Eungu Kang^1^, Yoon-Myung Kim^1^, Jin-Ho Choi^1^, Gu-Hwan Kim^2^, Beom Hee Lee^1,2^, Han-Wook Yoo^1,2^

#### ^1^Department of Pediatrics, Asan Medical Center Children's Hospital, University of Ulsan College of Medicine; ^2^Asan Medical Center Children's Hospital, University of Ulsan College of Medicine, Medical Genetics Center

**Purpose**: Congenital lipoid adrenal hyperplasia (CLAH) is caused by mutations in the StAR gene and StAR protein plays a critical role for transport of cholesterol to mitochondria. The aim of this study was to investigate StAR mutation spectrum and associated clinical and endocrinologic characteristics in patients with CLAH.

**Methods**: The study included 45 patients with CLAH from 42 unrelated families diagnosed by clinical features, endocrine profile, and mutation analysis. Clinical features, endocrine data, and radiologic finding were reviewed retrospectively. Seven exons and associated intronic flanking regions of the StAR gene were amplified by PCR and directly sequenced.

**Results**: Most patients (42/45, 93.3%) with StAR defect presented with adrenal crisis in the neonatal period, while 3 late-onset patients with skin hyperpigmentation after age 2 years. Among 45 patients, 19 were genetic males with 46,XY karyotype, 21 had a 46,XX, whereas the other 5 patients’ karyotype was not available. Patients with non-classic type CLAH presented with skin hyperpigmentation and chronic adrenal insufficiency without salt-wasting crisis after age 12 months. Two genetic females having compound heterozygous mutations showed late onset adrenal insufficiency, while one genetic male with compound heterozygous for p.Q258* and p.R272H was incompletely virilized and raised as a boy. Three pubertal-aged genetic females exhibited spontaneous breast development at age 10 to 13 years. One of them showed spontaneous menarche at age 13 years and experienced ovarian cyst torsion at age 14 years. Mental retardation was found in two patients. One patient died of adrenal crisis at the age of 2 months due to poor adherence to medication. The genotype of the StAR was clarified in all patients, identifying 9 different mutations. Of these, p.Q258* was the most common (86.9%, 73/84 alleles), suggesting founder effect.

**Conclusion**: CLAH has wide clinical spectrum from life-threatening adrenal insufficiency in early infancy to chronic adrenal insufficiency thatcan present much later in life. CLAH was known to be mainly caused by p.Q258* mutation in Korea by founder effect. However, this study indicate that mutations of the StAR gene have diverse spectrum with various mutations.

## O2-2 Biochemical diagnosis of 17 α -hydroxylase deficiency by urinary steroid metabolites in Japanese children

### Yuhei Koyama^1^, Keiko Homma^2^, Eishin Ogawa^3,4^, Ichiro Miyata^5^, azumichi Onigata^6,7^, Tomonobu Hasegawa^8^

#### ^1^LSI Medience Co. Special Pharmacology Analysis Department, Controlled Substance erated Tsting Group; ^2^Keio University Hospital, Central Clinical Laboratories; ^3^Department of Pediatrics, Teikyo University School of Medicine; ^4^Department of Pediatrics, Tohoku niversity School of Medicine; ^5^Department of Pediatrics, The Jikei University School of edicine; ^6^Department of Pediatrics, Shimane University Faculty of Medicine; ^7^Department of Pediatrics, Gunma University School of Medicine, Department of Pediatrics; ^8^Keio niversity School of Medicine

**Background**: 17 α -hydroxylase deficiency (17OHD) is one of congenital adrenal hyperplasia due to CYP17A1 gene abnormality. Affected 46,XY patients usually have female external genitalia. Corticosterone (B) metabolites and cortisol (F) metabolites ratio, a theoretical biochemical marker of 17a-hydroxylase activity, was reported to be increased in 17OHD. However, no data are available to distinguish 17OHD from cytochrome P450 oxidoreductase deficiency (PORD) which is a disease theoretically showing similar urinary steroid metabolites pattern.

**Objective**: The objective of this study was to establish differential diagnostic approach of 17OHD from PORD using urinary steroid profile.

**Methods**: We recruited genetically confirmed 5 Japanese children of 46,XY 17OHD, age between 6 months - 17 years, and 254 controls (22 PORD, 25 disorders of sex development (DSD), and 207 non-disease controls. We measured urinary steroid metabolites by gas chromatography mass spectrometry (mg/g creatinine) including B metabolites (B-group; 5a-, 5b-tetrahydrocorticosterone and 5a-, 5b-tetrahydro-11-dehydrocorticosterone), F metabolites (F-group; 5a-, 5b-tetrahydrocortisol and 5a-, 5b-tetrahydrocortisone), and pregnanetriolone (Ptl). We calculated B-group/F-group ratio.

**Results**: Twelve patients (all five 17OHD and 7 out of 22 PORD) showed B-group/F-group ratio of more than 1 (upper figure). Among 12 patients, Ptl showed no overlap between 17OHD and PORD using cutoff value of 0.3 mg/g creatinine (lower figure).

**Discussion**: These data indicated that biochemical differential diagnosis of 17OHD from PORD in Japanese children was possible by urinary steroid metabolites; B-group/F-group ratio and Ptl with cutoff values of 1 and 0.3 mg/ g creatinine, respectively. Future studies are needed to assess these urinary biochemical markers can distinguish 17OHD from isolated 17,20-lyase deficiency, another phenotype of CYP17A1 gene abnormality and adrenal tumor, likely showing similar urinary steroid metabolites pattern of 17OHD.

**Fig. 1 (abstract O2-2).**
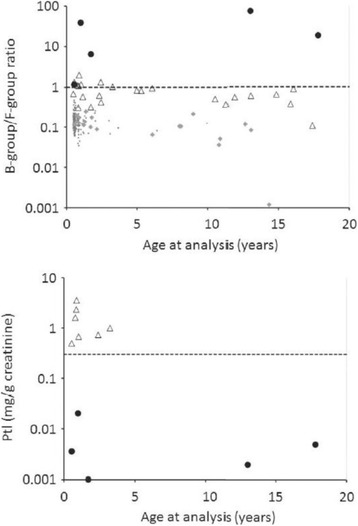
See text for description

## O2-3 Induction of Cyp21a1 with AAV vector ameliorates systemic steroid metabolism in a mouse model of congenital adrenal hyperplasia

### Yasuhiro Naiki^1^, Mami Miyado^2^, Reiko Horikawa^1^, Noriyuki Katsumata^2^, Shuji Takada^3^, Maki Fukami^2^

#### ^1^Division of Endocrinology and Metabolism, National Center for Child Health and Development; ^2^Department of Molecular Endocrinology, National Research Institute for Child Health and Development; ^3^Department of Systems BioMedicine, National Research Institute for Child Health and Development

**Background**: Congenital adrenal hyperplasia (CAH) due to steroid 21-hydroxylase (21-OH) deficiency (21-OHD) is an autosomal recessive disorder, in which CYP21A2 mutations or deletions result in underproduction of glucocorticoid and mineralocorticoid, and overproduction of androgens. Patients with CAH are treated with oral steroid supplementation, but optimal control of blood steroid levels remains difficult. Thus, new therapeutic approaches are still needed. Previously, adenovirus-mediated administration of human CYP21A2 to adrenal glands rescued the phenotype of a mouse model of 21-OHD. To date, adeno-associated virus vectors became a good candidate with its weak immunogenicity and are capability of delivering genes to various tissues to maintain stable expression. In this study, we examined whether transduction of murine Cyp21a1 in extra-adrenal tissues could rescue steroid metabolism in 21- OHD mice.

**Methods**: A naturally occurring mouse model of 21-OHD was obtained by mating heterozygous pairs. Heterozygous pregnant mothers received daily injections of dexamethasone from late pregnancy to the day of delivery, to prevent deaths of newborn pups. Homozygous newborn mice received daily injections of corticosterone and fludrocortisone during the first 3 weeks after birth. A serotype-2 AAV vector containing Cyp21a1 cDNA and a cytomegalovirus promoter was constructed and we injected the AAV vector to the thigh muscles of the homozygote mice aged between 3 and 10 months (n = 4 for Cyp21a1- containing vector and n = 2 for control). Serum progesterone and DOC levels were measured before and 4 weeks after injection. One AAV-injected mouse was subjected to monthly blood sampling during 8 months after injection.Progesterone and DOC in serum samples were measured by liquid chromatography tandem mass spectrometry.

**Results**: Serum progesterone/DOC ratios were markedly reduced in all four animals at 4 weeks after injection. The effect had been continued until 8 months after injection. (Figure)

**Conclusion**: These results indicate that extra-adrenal induction of Cyp21a1 ameliorates steroid metabolism in 21-OHD mice for long term. This study suggests a novel therapeutic strategy for congenital adrenal hyperplasia, which warrants further investigations.

**Fig. 1 (abstract O2-3).**
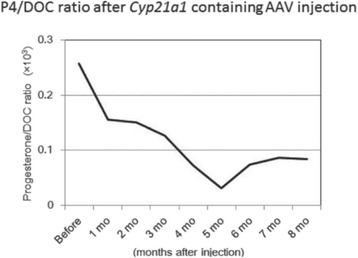
See text for description

## O2-4 The effect of glucocorticoid treatment on bone mineral density in children with congenital adrenal hyperplasia: systematic review and meta-analysis

### Agustini Utari^1,2^, Siti RF Saldi^3^, Bambang Tridjaja^1^, Jose R Batubara^1^

#### ^1^Division of Pediatric Endocrinology, Department of Pediatric, Faculty of Medicine , University of Indonesia/ Cipto Mangunkusumo Hospital; ^2^Division of Pediatric Endocrinology, epartment of Pediatric, Faculty of Medicine , Diponegoro University ; ^3^Clinical Epidemiology and Evidence-Based Medicine (CEEBM) Unit, Faculty of Medicine , University of Indonesia/ Cipto Mangunkusumo Hospital

**Background**: Congenital Adrenal Hyperplasia (CAH) is a disorder characterized by the impaired activity of one of the enzymes needed for cortisol synthesis. The most common cause of CAH is the 21-hydroxylase deficiency that leads to cortisol and aldosterone deficiency in addition to androgen excess. Therapy in these cases is a long-life treatment of glucocorticoid and often mineralocorticoid. Many reports consider these patients were at risk of glucocorticoid induces osteoporosis. However, bone tissue is significantly affected by androgens and glucocorticoid. There were conflicting results regarding the effect of glucocorticoid treatment on bone mineral density (BMD) in CAH patients. Objectives: The objectives of this present study were to evaluate the effect of glucocorticoid treatment on BMD in children with CAH compared to normal healthy children.

**Search methods**: We performed literature search of studies using Cochrane Library, MEDLINE, EBSCO, PROQUEST, and another database to identify studies of BMD and CAH through May 2015. We also searched the reference lists of articles and contacted researchers in the field. We retrieved English language articles for review, and we searched for both published and unpublished studies.

Selection criteria: Randomized controlled trial, cohort, case control and cross sectional studies was considered. The type of participants was CAH patient who has already on glucocorticoid treatment at least two years. The comparison for these patients were normal healthy children. The outcome measure in this review was Whole BMD Z-score and Lumbar Spine BMD Z-score, which were measured by dual-energy X-ray absorptiometry (DXA).

**Data collection and analysis**: Two authors reviewed (AU and JB) independently abstracts for inclusion and read full- text articles to extract data. The risk of bias was assessed regarding randomisation, allocation sequence concealment, blinding, incomplete outcome data, selective outcome reporting, and other biases.

**Main Results**: We included nine studies involving 222 patients for systematic review; however only four studies involving 84 patients could be included in the meta-analysis. There were no randomized studies that met inclusion criteria. Thus we use non-randomised study that fulfilled the inclusion criteria. The meta-analysis showed that there was no significant mean difference between Whole BMD Z-Score and Lumbar spine BMD Z-Score among children with CAH who treated with glucocorticoid compared to normal healthy child (p=0.57, 95% CI, -0.46-0.84 and p = 0,86; CI 95%, -2,3 – 1,94, respectively).

**Conclusions**: Whole BMD and Lumbar spine BMD Z-Score in children with CAH treated with the glucocorticoid is similar with normal children.

## O2-5 Late-onset glucocorticoid responsive circulatory instability of preterm infants

### Hye Rim Chung^1^, Won Im Cho^1^, Chang Won Choi^2^, Byeong il Kim^2^

#### ^1^Division of Endocrinology and Metabolism / Department of Pediatrics, Seoul National University Bundang Hospital; ^2^Division of Neonatology / Department of Pediatrics, Seoul National University Bundang Hospital

**Background**: An increasing number of preterm infants were treated with glucocorticoids for late-onset circulatory instability (CI) thought to be caused adrenal insufficiency. However, the presence of adrenal insufficiency, which is presented with late-onset CI in preterm infants, is not fully verified yet. We aimed to describe the incidence and clinical features of late-onset CI which responded to glucocorticoid replacement, and to determine the frequency of low serum cortisol values that meet criteria for relative adrenal insufficiency in these preterm infants.

**Methods**: Among 1,840 preterm infants who were admitted to a single neonatal intensive care unit between 2010 and August 2014, 47, who showed clinical signs of CI after 7 days-old and were recovered shortly after hydrocortisone replacement, were enrolled. Clinical signs of CI included hypotension, hyponatremia, hyperkalemia and oliguria. Infants with infection, hypovolemia, and cardiac problems were excluded. Medical records including serum cortisol levels during CI were reviewed retrospectively.

**Results**: Median gestational age (GA) of 47 infants was 28+2 weeks (range, 24+0 - 33+6 weeks), and median birth weight was 995 g (range, 430 - 2,275 g). Median age of development of CI was 20 days (range, 8 - 49 days). Incidence of CI was 2.6% (47/1,840) in all preterm infants; 8.65% (47/543) in infants of ≤ 34 weeks of GA; 18.2% (22/110) in infants of ≤ 28 weeks of GA. Incidence of CI in infants below 1,500g birth weight was 13% (40/302). Median serum cortisol level during CI was 8.0 μ g/dL (range, 2.0 - 12.0 μ g/dL), which met the criteria of vs. 129.3 mmol/L, P <0.001), and hourly urine output (3.7 vs . 1.7 mL/kg/hour, *P* <0.001) were decreased, and serum potassium levels (4.64 vs .5.99 mmol/L, P <0.001) were increased when compared the levels before CI. The clinical signs of CI were improved within 24 hours after hydrocortisone replacement in all 47 infants.

**Conclusions**: Our findings suggest that adrenal insufficiency is presented with late-onset glucocorticoid responsive CI, and is not rare in preterm infants.

## O2-6 Comprehensive Steroid Profile in Classic 21-Hydroxylase Deficiency: Clinical and Hormonal Correlations

### Karn Wejaphikul^1,2^, Hataichanok B. Kongmanas^1^, Pongsathorn ittichan^3^, Watchara Sirisuwan^3^, Thiti Snabboon^3^, Taninee Sahakitrungruang^1^

#### ^1^Faculty of Medicine, Department of Pediatrics, Chulalongkorn University; ^2^Faculty of Medicine, Department of Pediatrics, Chiang Mai University; ^3^Faculty of Medicine, Department of Internal Medicine, Chulalongkorn University

**Introduction**: Optimal treatment of congenital adrenal hyperplasia (CAH) due to 21-hydroxylase deficiency (21-OHD) requires monitoring of clinical parameters and steroid markers such as 17-hydroxyprogesterone (17-OHP), androstenedione, and testosterone. However, an increase of 17-OHP levels is commonly observed during pubertal period, due to gonadal co-secretion of 17-OHP. Since the accumulated 17-OHP can be converted to 21-deoxycortisol (21-DOC) via 11-hydroxylation in adrenals only. We hypothesize that 21-DOC may serve as a better adrenal steroid marker for treatment monitoring in CAH youth.

Objectives: We aim to evaluate the utility of comprehensive serum adrenal steroids by liquid chromatography-tandem mass spectrometry (LC-MS/MS) as markers of clinical control in CAH youth, and compare two different 17-OHP and testosterone assays (LC-MS/MS vs. commercial immunoassays).

**Methods**: A cross-sectional study of 37 patients (24 females) with classic 21-OHD aged 1-26 years were enrolled. Single serum samples were collected at 8 AM before the morning dose of glucocorticoid to measure steroid panels by LC-MS/MS and immunoassays. Patients were classified as being in “good control” or “poor control” based on clinical criteria including signs of androgen excess, degree of hyperpigmentation, bone age advancement, and ACTH levels. Comparisons of serum steroid concentrations were performed between two groups. The receiver operating characteristic (ROC) curves were used to determine the cut-off values for diagnosing “poor control”.

**Results**: Serum 17-OHP, progesterone, androstenedione, testosterone, and DHEA concentrations were higher in “poor control” group, but 21-DOC and DHEAS levels were not different. The ROC curves showed that 17-OHP, androstenedione, progesterone, and testosterone concentrations were the best hormonal predictors of clinical poor control with areas under the curves (95%CI) of 0.91 (0.79-1.0), 0.92 (0.82- 1.0), 0.88 (0.76-1.0), 0.87 (0.73-0,99), P 0.001, respectively. By the Bland & Altman plot, the differences of 17-OHP and testosterone levels between LC-MS/MS and immunoassay were 9.2 ng/mL (95%CI -82 to 101), and 0.6 ng/mL (95%CI -2.1 to 3.2), respectively. The higher levels of 17-OHP or testosterone exhibited higher discrepancy between two methods.

**Conclusion**: Serum 17-OHP, androstenedione, and testosterone concentrations are of significance in diagnosing clinical poor control. 21-DOC is not useful for treatment monitoring in CAH. Despite decent overall correlation, absolute 17-OHP and testosterone concentrations differed substantially between two different assays. Frequent monitoring of clinical parameters and standardized methods of steroid assays are crucial to diminish the

## O3-1 Analysis of endocrinopathy as late effects in Childhood cancer survivors at a single institute in Japan

### Tomoko Yoshida^1^, Kanako Nakao^1^, Yuta Chiba^1^, Yumiko Terada^1^, Asuko Ogiwara^1^, Keisuke Yoshii^1^, Chikako Kiyotani^1,2^, Yoko Shioda^2^, Tomoo Osumi^2^, Daisuke Tomizawa^2^, Motohiro Kato^2^, Kimikazu Matsumoto^2^, Yasuhiro Naiki^1^, Reiko Horikawa^1^

#### ^1^Division of Endocrinology and Metabolism, National Center for Child Health and Development; ^2^National Center for Child Health and Development, Children's Cancer Center

**Background**: The survival rates for children and adolescents with malignant cancers have increased these days, along with the improvement of treatments modality. Consequently, the childhood cancer survivors (CCSs) with late treatment-related complications, especially with endocrinopathy are increasing.

**Objective**: To assess the frequency and characteristics of endocrinopathy of CCSs in single institute in Japan.

**Methods**: 272 CCSs from 2002 to 2016 were involved in this study. Patients with craniopharyngioma were excluded because of no chemotherapy and radiotherapy. 71 patients with benign disorders, complicated with other syndromes, or last cancer treatment within one year were excluded from 272 CCSs. The number of original diseases are: solid tumors 83 (neuroblastoma, langerhans cell histiocytosis, retinoblastoma, rhabdomyosarcoma, hepatoblastoma, Wilms’ tumor), brain tumors 51 (medulloblastoma, germinoma, teratoma, optic glioma, ependymoma), hematological malignancies 67 (Acute lymphocytic leukemia, acute myeloid leukemia, lymphoma). The medical history and the hormonal data of 201 patients (98 males, 103 females, aged 2 ~ 19 (average 12) years) at our endocrine follow-up clinic for CCSs were obtained retrospectively.

**Results**: Over all, growth hormone deficiency were observed in 32 patients (20 in brain tumor (39.2% in brain tumor) and 12 in others (8.0% in other tumor)), thyroid dysfunction in 22 (12 in brain tumor (23.5%) and 10 in others (6.6%)), adrenal dysfunction in 18(8 in brain tumor (15.7%) and 10 in others (6.6%)), respectively. Among those with brain tumors, AVP replacement was performed in 9 (17.6%). In 146 patients with pubertal age (male>13 years and female>11years), sex hormone replacement were necessary in 15 (10.3%). GnRH agonist was prescribed for 21 (10.4%) in children below 12 years old, except 1 case, because of central precocious puberty or early puberty for height. Hormone replacement was more common in patients with hypothalamic-pituitary tumors. Patients with neuroblastoma (n=27) also showed higher frequency of hormone replacement therapy. Adult height was obtained from 5 patients with neuroblastoma, 3 of them showed adult height less than -4SD without GHD.

**Assessment**: Patients with brain tumor was at higher risk of pituitary dysfunction. Patients with neuroblastoma, when compared to those with other peripheral solid tumor, tended to have growth failure and hormone deficiency suggesting that intense chemotherapy and radiation therapy may have disrupting effect on endocrine system and resistance to growth hormone.

**Conclusion**: Endocrinopathy is common for CCSs. We need to continue surveillance to know more to follow them up appropriately.

## O3-2 Genome-wide analysis of differential DNA methylation in Silver-Russell syndrome

### Di Wu, Chunxiu Gong

#### Department of Endocrinology, Genetics and Metabolism, Beijing Children's Hospital, Capital Medical University

**Background**: Silver-Russell Syndrome (SRS) is clinically heterogeneous disorder characterized by low birth weight, postnatal growth restriction and variable dysmorphic features. The aetiology of SRS is complex and current evidence strongly implicates imprinted genes. Although approximately half of all patients exhibit DNA hypomethylation at the H19/IGF2 imprinted domain, and around 7%-10% have maternal uniparental disomy of chromosome 7(UPD (7) mat), no clear mechanism is apparent in the other patients. In this study, we aim to look for further DNA methylation defects in SRS patients.

**Methods**: We measured DNA methylation in 7 SRS patients and 5 controls at >485,000 CpG sites using DNA methylation microarrays. We analyzed methylation changes genome-wide as well as at known imprinted regions to identify SRS-associated epimutations. Then we used bisulfite sequencing and digital PCR to identify the differentially methylated regions (DMRs) we found.

**Results**: Our analysis identifies epimutations at the previously characterised domains of H19/ IGF2, providing proof of principle that our methodology can detect DNA methylation changes at imprinted loci. In addition we discovered a novel imprinted gene OSBPL5 associated with SRS and located at chromosome 11p14with the probe cg25963939 hypomethylated in 4/7 patients (P=0.023, β =-0.243). We also report DMRs in other genes including TGF β 3 AHSF1 AGAP43 ANOTCH4 AMYH14, which might be associated with SRS by GO pathway analysis.

**Conclusions**: We identified the probe cg25963939, located at the 5’UTR of imprinted gene OSBPL5, as a noval DMRs, which associated with SRS. This findings provide a new mechanism of SRS etiology and aid the further stratification of SRS patients by molecular phenotypes.

## O3-3 Serum antimüllerian hormone and inhibin B as potential markers for progressive central precocious puberty in girls

### Linqi Chen, Ting Chen, Haiying Wu, Fengyun Wang, Xiuli Chen, Ngrong Xie

#### Division of Endocrinology and Metabolism / Department of Pediatrics, The Children’s Hospital of Soochow University

This abstract is not included as it has already published.

## O3-4 Genotype and phenotype of 101 Vietnamese Patients with Congenital Hyperinsulinism

### Dung Chi Vu^1^, Duong Anh Dang^1^, Ngoc Thi Bich Can^1^, Khanh Ngoc Nguyen^1^, Thao Phuong Bui^1^, Hai Thanh Le^1^, Dat Phu Nguyen^1^, Dien Minh Tran^1^, Ellard Sian^2^, Flanagan E Sarah^2^

#### ^1^Department of Endocrinology, Metabolism and Genetics, National Children's Hospital; ^2^University of Exeter Medical School. UK, Molecular Genetics, Royal Devon & Exeter NHS

**Background**: Hyperinsulinemic hypoglycemia (HH) is a consequence of unregulated insulin secretion by pancreatic β -cells. Congenital HH is caused by mutations in genes involved in regulation of insulin secretion (ABCC8 , KCNJ11 , GLUD1 , CGK , HADH , SLC16A1, HNF4A and UCP2 ). Severe forms of congenital HH are caused by inactivating mutations in ABCC8 and KCNJ11, which encode the two components of the pancreatic β -cell ATP-sensitive potassium channel.

**Objective and hypotheses**: Our aim is to identify mutations in the ABCC8 and KCNJ11, HNF4A and GLUD genes, and to describe genotype and phenotype correlations of Vietnamese children with congenital hyperinsulinism.

**Method**: A prospective study was conducted on 101 cases with congenital hyperinsulinism diagnosed and treated at Vietnam Children’s Hospital from January 2007 to June 2016. Patients were selected by using inclusion criteria of Hussain K (2008). All exons of ABCC8; KCNJ11 , HNF4A and GLUD1 were amplified from genomic DNA and directly sequenced.

**Results**: Mutations were identified in 52 cases (51.5%) including mutations of ABCC8 gene (46 cases; 45.5%), Among these cases 26 with homozygous/compound heterozygous of ABCC8 and 20 cases with one paternal/maternal mutation of ABCC8 gene); KCNJ11 (5 cases; 5.0%), HNF4A (1 case; 1.0%). 100% of cases with homozygous/compound heterozygous recessive mutations or one paternal dominant mutation of ABCC8 gene did not respond to diazoxide treatment and required 95% pancreatectomy or octreotide injection. Other cases without identified mutations responded to diazoxide and/or glucose infusion.

**Conclusion**: children with congenital hyperinsulinism should be performed mutation analysis which helps in making diagnosis and treatment decision. Families of children with congenital hyperinsulinism should be given genetic counseling. Prenatal diagnosis should be performed as well as follow - up and treatment should be given to children with congenital hyperinsulinism immediately after birth.

## O3-5 Short and long-term outcome of patients with congenital hyperinsulinism

### Yumiko Terada, Yusuke Fujisawa, Yuta Chiba, Yasuko Ogiwara, Tomoko Yoshida, Kanako Nakao, Keisuke Yoshii, Yasuhiro Naiki, Reiko Horikawa

#### Division of Endocrinology and Metabolism, National Center for Child Health and Development

**Background**: Congenital hyperinsulinism (CHI) occurs in 1/25000 to 1/50000 birth and frequently becomes the cause of persistent hypoglycemia in neonates. Most of persistent hypoglycemia states are caused by genetic defects. Some can be managed by medication, some can be cured by surgical resection of focal pancreatic lesion, but some are intractable and uncontrolled by any medication.

**Objective**: To evaluate the adequacy of treatment choice and long-term prognosis for CHI patients in one institute.

**Methods**: We retrospectively investigated clinical course, laboratory data, diagnosis, treatment modality, and short and long-term outcome in patients with hyperinsulinemic hypoglycemia through their medical records from 2002 to 2016.

**Results**: 41 patients from 3-month-old to 46-year-old (current age), diagnosed as transient or persistent hyperinsulinemic hypoglycemia were involved in this study. Observation periods are from 3 months to 14 years. Most of the patients were diagnosed in their first to second days after birth, while 9 patients were diagnosed at 3 to 9-month-old. 12 cases (31.6%) were transient, and 10 out of these 12 were born preterm or LFD (Light for date). Among 41 patients, 12 (31.6%) were born HFD (Heavy for date), 9 of which had ATP-sensitive potassium channel abnormalities. 19 patients (46%) were diagnosed as CHI. Genetic evaluations were performed in 21 patients; particular gene mutations were found in 12 patients (57%) (ABCC8 gene mutation in 9, KCNJ11 gene mutation in 1, GLUD1 gene mutation in 2 ). The long-term prognosis of 19 CHI patients, 1 case was treated only by diet control, 8 cases continue diazoxide, 1 case was controlled by octreotide, 1 case by diazoxide and octreotide combination, and 8 cases had partial pancreas resection or subtotal pancreas resection. The decision of partial resection was decided upon the result of 18F-DOPA PET-CT. Among those who had subtotal (>95%) resection, 4 patients became insulin-dependent diabetes mellitus, and 2 cases continuing the treatment for hyperinsulinemic hypoglycemia. Considerable mental retardation is observed in only one grown-up case.

**Conclusions**: Genetic testing and imaging study using 18F-DOPA PET-CT, in addition to clinical evaluation, are helpful for the decision of treatment choice. Long-term neurological outcome with adequate medical and surgical treatment seems to be better in recent cases when compared to old case, however, further evaluation is necessary.

## O3-6 Rare frequency of mutations in pituitary transcription factor genes in patients with combined pituitary hormone deficiency or isolated growth hormone deficiency in Korea

### Yoon-Myung Kim Kim^1^, Eungu Kang^1^, Jin-Ho Choi^1^, Chang-Woo Jung^1^, Sun Hee Heo^2^, Minji Kang^2^, Gu-Hwan Kim^3^, Beom Hee Lee^1^, Han-Wook Yoo^1^

#### ^1^Department of Pediatrics, Asan Medical Center Children's Hospital, University of Ulsan College of Medicine; ^2^Asan Medical Center Children's Hospital, University of Ulsan College of Medicine, Asan Institute for Life Sciences; ^3^Asan Medical Center Children's Hospital, University of Ulsan College of Medicine, Medical Genetics Center

**Purpose**: Combined pituitary hormone deficiency (CPHD) is caused by mutations in pituitary transcription factors involved in the development of the hypothalamic-pituitary axis. Minor allele frequencies of genes involved in CPHD are so low and varies substantially between ethnicities. This study evaluated the frequency of mutations in the most relevant transcription factor genes (POU1F1 , PROP1 , LHX3 , LHX4 , and HESX1 ) in patients with CPHD and isolated growth hormone deficiency (IGHD).

**Method**: This study included 27 patients with IGHD and CPHD. The following parameters were analyzed: age at diagnosis, height, weight, birth history, combined anterior pituitary function tests, and sellar magnetic resonance image (MRI) findings. Mutation analysis of the POU1F1 , PROP1 , LHX3 , LHX4 and HESX1 genes were performed using genomic DNA from peripheral blood leukocytes.

**Results**: IGHD was observed in 4 patients and CPHD in 23 patients. Age at diagnosis was 8.28 ± 7.25 years (range, 0.2–16.9 years) in IGHD and 13.48 ± 10.46 years (range, 0.2–35 years) in CPHD (P = 0.37). Serum IGF-1 and peak GH levels after GH provocation tests were significantly low in patients with CPHD compared to those of IGHD (P <0.05). The sellar MRI findings found structural abnormalities in 3 patients with IGHD (75%) and 21 patients with CPHD (91.3%) (P = 0.62). Mutation analysis identified homozygous c.326G>A (p.R109Q) mutations in HESX1 in a patient with CPHD. No mutation was identified in POU1F1 , PROP1 , LHX3 , and LHX4 genes in the other patients.

**Conclusion**: Patients with CPHD had severe GHD than those with IGHD. Patients with GHD should be evaluated for other systemic diseases and endocrine functions by means of ophthalmologic examination, sellar MRI, and combined anterior pituitary function tests. As the mutation frequency of pituitary transcription factors is rare in patients with CPHD and IGHD, further research should be done to investigate other causative genes or environmental factors.

## O4-1 A reversible albumin-binding once-weekly growth hormone (GH) treatment (somapacitan; NNC0195-0092) induces a dose- dependent IGF-I response similar to daily GH therapy in children with growth hormone deficiency (GHD)

### Michael Højby Rasmussen^2^, Tadej Battelino^1^, Jean de Schepper^3^, Nehama Zuckerman-Levin^4^, Zoran Gucev^5^, Lars Savendahl^6^

#### ^1^University of Ljubljana, Faculty of Medicine; ^2^Novo Nordisk A/S, Global Development; ^3^Division of Paediatric Endocrinology, Universitair Ziekenhuis Brussel; ^4^Rambam Medical Center, Pediatric and Obesity Clinic; ^5^Medical Faculty Skopje, University Paediatrics Clinic; ^6^Department of Women's and Children's Health, Karolinska Universitetssjukhuset

This randomised, open-label, active-controlled, dose-escalation trial (NCT01973244) investigated single-dose exposure and insulin-like growth factor (IGF)-I response to somapacitan in 32 children with GHD after a 7–10-day GH-washout period. Pre-pubertal children (aged 6–13 years) were assigned equally to four cohorts: 0.02, 0.04, 0.08 and 0.16 mg/kg somapacitan, pending safety assessments performed at each dose level increase. Within each cohort, six children were randomised to a single dose of somapacitan and two were randomised to Norditropin® SimpleXx® once daily at a fixed dose of 0.03 mg/kg/day for a week. The once-weekly dose range of somapacitan was expected to cover the clinically relevant dose range in GHD as guided by simulations of the anticipated PK and IGF-I response in children with GHD. There were dose-dependent increases in somapacitan serum concentrations and in baseline-adjusted IGF-I AUC (0–168h) (area under the IGF-I concentration–time curve from 0–168h) and maximum concentration (Cmax). Compared with once-daily GH, IGF-I responses were lower with 0.02 mg/kg somapacitan (estimate: 0.56 ng*h/mL [95%CI: 0.44; 0.71], p <0.0001), but similar with 0.04 and 0.08 mg/kg (estimated ratios, approximately 1:1) and higher with 0.16 mg/kg (1.25 ng*h/mL [95%CI: 0.98; 1.59], p =0.0672). Only an initial peak in IGF-I response to 0.08 mg/kg was outside the reference range (+2 to –2 SDS). A similar dose-dependent response was observed for IGF-binding protein-3 (IGFBP-3) with the initial peak being largest with 0.16 mg/kg somapacitan (2.3 mg/kg vs. 0.6–1.3 mg/kg for other doses) and subsequent stabilisation within reference range. No serious adverse events with either treatment and few local tolerability issues were observed (four transient injection site reactions in three children all receiving 0.16 mg/kg somapacitan). All somapacitan doses were well tolerated with no clinically relevant safety or immunogenicity issues observed.

In a model-based analysis, steady-state PK and IGF-I responses were simulated across dose levels and compared to daily GH treatment for Cmax and average concentration (Cavg). In this model, an IGF-I dose of 0.04 mg/kg is expected to provide Cmax IGF-I levels matching average daily GH treatment; 0.08 mg/kg is expected to provide Cavg IGF-I levels matching average daily GH treatment; 0.16 mg/kg is expected to provide higher IGF-I levels, with average concentrations within +2 SDS after a single dose or at steady state.

In conclusion, the single-dose results indicate that doses of 0.04, 0.08 and 0.16 mg/kg may be suitable for once-weekly administration of somapacitan and may be effective for the treatment of childhood GHD.

## O4-2 Machine Learning and Transcriptomics Accurately Predict Diagnosis and Severity of Childhood Growth Hormone Deficiency

### Philip Murray^1^, Adam Stevens^1^, Ekaterina B Koledova^2^, Pierre Hatelain^3^, Peter Clayton^1^

#### ^1^University of Manchester and Royal Manchester Children's Hospital; ^2^Merck KGaA; ^3^University Claude Bernard

**Background**: The diagnosis of Growth Hormone Deficiency (GHD) involves the use of multiple GH stimulation tests which require day case admission, multiple blood samples and the administration of pharmacological agents. Replacing these tests with a single blood sample would be a major advance for patients being evaluated for GHD.

**Aim**: To assess the utility of gene expression (GE) profiling and candidate SNP analysis for the diagnosis and classification of GHD.

**Method**: Pre-pubertal treatment-naïve children with GHD (n=98) were enrolled from the PREDICT study and controls (n=26) acquired from online datasets. Whole blood gene expression (GE) was determined with Affymetrix HU133v2.0 microarrays. Genotyping was performed on DNA extracted from whole blood using Illumina GoldenGate for 1536 SNPs, located on 103 candidate genes. The correlation between GE and peak GH was investigated using rank regression and a Random Forest algorithm tested for prediction of the presence of GHD and in classification of GHD into severe (peak GH>4 μg/L ). For GHD severity classification, data on age, gender, baseline IGF-I and IGFBP-3 levels were added to the Random Forest model along with SNP genotype. Performance was assessed using Area under the Receiver Operating Characteristic Curve (AUC-ROC). A biological network of GE related to peak GH levels was generated and cluster hierarchy assessed.

**Results**: Rank regression identified 347 probesets representing 271 genes where expression correlated with peak GH concentrations: (R = + 0.28, p<0.01). These 347 probesets gave an AUC of 0.98 (sensitivity 100%, specificity 96%) for predicting GHD status (GHD versus controls). At the DNA level, 18 SNPs in 12 genes were associated with peak GH concentrations; 16/18 were intronic and none were rare (defined as Minor Allele Frequency <1%). The function of the genes associated with the SNPs included pituitary transcription factor (POU1F1 ), generation of oestrogen (CYP19A1 ), IGF binding (IGFBP1 ), apoptosis (BCL2), cell cycle (CCND3 ) and signal transduction (PTPN1 , RARA ). Random Forest analysis was also able to accurately predict GHD severity with an AUC of 0.93 using transcriptomic data but not improved with addition of demographic, biochemical or SNP genotype data.

**Conclusion**: GE profiling differentiates normal subjects from those with GHD and, in a cohort of GHD subjects, accurately predicts GHD severity. It may therefore be a useful tool in clinical practice to aid in the diagnosis of GHD, potentially replacing two GH stimulation tests with a single blood sample.

## O4-3 Comprehensive clinical studies in 30 patients molecularly diagnosed with Temple syndrome

### Masayo Kagami^1^, Keisuke Nagasaki^2^, Rika Kosaki^3^, Reiko Horikawa^4^, Yasuhiro Naiki^4^, Shinji Saitoh^5^, Toshihiro Tajima^6^, Akie Nakamura^1^, Keiko Matsubara^1^, Maki Fukami^1^, Tsutomu Ogata^1,7^

#### ^1^Department of Molecular Endocrinology, National Research Institute for Child Health and Development; ^2^Department of Homeostatic Regulation and Development, Niigata University Graduate School of Medical and Dental Science; ^3^Department of Clinical Laboratory Medicine, National Center for Child Health and Development; ^4^Division of Endocrinology and Metabolism, National Center for Child Health and Development; ^5^Department of Pediatrics and Neonatology, Nagoya City University Graduate School of Medical Sciences; ^6^Department of Pediatrics, Jichi Children's Medical Center Tochigi; ^7^Department of Pediatrics, Hamamatsu University School of Medicine

**Background**: Human chromosome 14q32.2 carries paternally expressed genes and maternally expressed genes together with the germline- derived DLK1-MEG3 intergenic differentially methylated region (IG-DMR) and the postfertilization-derived MEG3 -DMR. Consistent with this, maternal uniparental disomy 14 (UPD(14)mat), and epimutations and microdeletions affecting the imprinted region of paternal origin result in discernable and non-specific clinical features such as pre- and postnatal growth failure, hypotonia, early onset puberty, and small hands. Recently, this clinically recognizable disorder has been named “Temple syndrome” (TS14) (OMIM 616222) and identified in 58 patients. Clinical features of TS14 overlap with that of Prader-Willi syndrome (PWS) and Silver-Russell syndrome (SRS). Here, we performed comprehensive clinical studies in 30 patients molecularly diagnosed with TS14.

**Subjects and Methods**: We performed methylation analysis for the IG-DMR and the MEG3 -DMR in 186 patients with SRS-like phenotype without 11p15 loss of methylation or UPD(7)mat,157 patients with PWS-like phenotype with normal methylation pattern of the SNRPN - DMR, 202 patients with SGA-short stature (SGA-SS), together with 33 patients with TS14 phenotype. For patients with hypomethylation of these DMRs, we carried out structural analysis to detect paternal microdeletion, and microsatellite analysis to detect UPD(14)mat. To collect detailed clinical data of all patients, we used a comprehensive questionnaire from attending physicians.

**Results**: Thirty Japanese patients with TS14, UPD(14)mat (n=21); epimutations (n=5); microdeletions (n=3); and unknown (n=1), were identified. Their clinical diagnoses were TS14 (n=5), PWS (n=13), SRS (n=6), SGA-SS (n=2), and others (n=4). Fifteen patients had the score of 4 or more in Netchine-Harbison SRS clinical scoring. The combinatory method of PNA-NGS can detect somaticedian SD of birth weight, birth length, present height, and present weight are –2.1, –2.7, –2.3 and –1.6, respectively. Early onset puberty was identified in 11 out of 15 patients older than 4 years. Motor development was delayed in early infancy due to muscular hypotonia. Median developmental/intellectual quotient (DQ/IQ) was 83 (rage, 53–114), and two out of 15 patients who reached school age needed special education. Hyperlipidemia was identified in four out of 21 patients whose laboratory data were obtained. The median BMI was 15.1 (range, 10.9–37.3)

**Discussion**: We show detailed clinical features and course of 30 Japanese TS14 patients. Because of non-specific clinical features, clinical diagnosis of TS14 was difficult. Methylation analyses for the DMRs on chromosome 14 should be considered in patients with SRS- or PWS- compatible phenotype but no abnormal methylation pattern of the DMRs on chromosome 7, 11, and 15, as well as in patients with SGA-SS and hypotonia.

## O4-4 Genome-wide copy-number analysis of fifty Silver-Russell syndrome patients without known etiology

### TakanobuInoue^1,2^, Akie Nakamura^1^, Tomoko Fuke^1^, Kazuki Yamazawa^1^, Shinichiro Sano^1^, Keiko Matsubara^1^, Akira Oka^2^, Maki Fukami^1^, Masayo Kagami^1^

#### ^1^Department of molecular endocrinology, National Center for Child Health and Development; ^2^Department of Pediatrics, The University of Tokyo Hospital

**Background**: Silver-Russell syndrome (SRS) is a rare disorder associated with pre- and postnatal growth failure and characteristic features. SRS is genetically heterogeneous. H19 -differentially methylated region (DMR) hypomethylation (H19 -hypo) and maternal uniparental disomy chromosome 7 (upd(7)mat) can be detected in approximately 50% of SRS patients, and the etiology of remaining patients is unknown. Recently, some cases with Temple syndrome, upd(16)mat, upd(20)mat, and submicroscopic chromosomal abnormality were recognized in SRS patients without known etiology, but sample size of these cohort was small.

**Patients and Method**: This study consisted of fifty SRS patients diagnosed based on Netchine-Harbison SRS clinical scoring system (NH- CSS). To clarify the exact frequency of patients with PCNV in SRS patients without known etiology, we included only patients with normal methylation pattern for nine DMRs (H19 , Kv, IG, MEG3 , PLAGL1 , PEG1 , PEG10 , SNRPN , and GNAS A/B). We extracted genomic DNA from peripheral leucocytes. Oligoarray comparative genomic hybridization was performed in all patients using 8 × 60K catalog array (Agilent technology). To determine whether copy number variations identified in the patients were pathogenic or not, we utilized the information from Database of Genomic Variants and medical publications. The frequency of clinical features between patients with pathogenic copy number variants (PCNV) and those with H19 -hypo or upd(7)mat (previously reported by us) was analyzed by Fisher’s exact probability test.

**Result**: PCNV were detected in five patients in our cohort. The abnormalities included 4p microdeletion, 11p15 microduplication (two patients), 17p12 microduplication, and mosaic 18 trisomy. Clinical features of patients with PCNV are shown in the Table. Of note, among the patients with PCNV four patients presented congenital heart diseases and three patients developed epilepsy. The frequency of relative macrocephaly and body asymmetry was lower in the SRS patients with PCNV than in H19 -hypo group.

**Discussion**: We identified five patients with PCNV in fifty SRS patients satisfying N-H CSS. Our study revealed exact frequency of patients with PCNV in SRS patients without known etiology. We suggest that copy number analysis would improve genetic diagnosis of the SRS patients without known etiology, especially those with atypical features including congenital heart defect or epilepsy.

**Table 1 (abstract O4-4).**
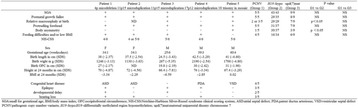
See text for description

## O4-5 Efficacy and safety of recombinant human growth hormone treatment in patients with short stature related to bone and cartilage diseases

### Guangling Li^1^, Shiyao Wang^1^, Huijuan Zhu^1^, San A^2^, Hongbo Yang^1^, Linjie Wang^1^, Fengying Gong^1^, Zimeng Jin^1^, Hui Pan^1^

#### ^1^Peking Union Medical College Hospital, Chinese Academy of Medical Science, Department of Endocrinology, Key Laboratory of Endocrinology, National Health and Family Planning Commission; ^2^Binhai Genomics Institute, BGI-Tianjin, Tianjin Enterprise Key Laboratory of Clinical Molecular Diagnostic

**Objective**: To study the clinical characteristics of patients with short stature related to bone and cartilage diseases (BCD), and evaluate the efficacy and safety of the recombinant human growth hormone (rhGH) treatment in BCD patients.

**Methods**: Clinical data of 33 cases of BCD patients were retrospectively analyzed. Eleven of them had undergone genetic testing. Based on receiving rhGH treatment or not, all BCD patients were divided into the treated group (BCD-T) and the untreated group (BCD-C). Twenty- one age/gender matched growth hormone deficiency (GHD) patients were collected as the control (GHD-T) group. Clinical and laboratory parameters were analyzed between each follow-up and group. The safety of rhGH treatment in BCD patients was also evaluated.

**Results**: Thirty-three BCD patients with mean age of 7.74 years old and mean initial height standard deviation score (Ht-SDS) of -4.50 included 18 achondroplasia/hypochondroplasia (54.5%) patients, 5 spondyloepiphyseal dysplasia (15.2%) patients, 3 multiple epiphyseal dysplasia patients, and 7 others. All patients underwent genetic testing existed gene mutations. Each of FBN1, FGFR3 and COL2A1 mutations revealed in 2 patients. The other mutations included COL9A1, NPR2, TRAPPC2, RUNX2 and CENPJ . All 15 patients in the BCD-T group showed significant improvement in growth velocity (GV) and Ht-SDS after 12-month treatment (P<0.05). While 18 patients in BCD-C group had no change in GV and Ht-SDS before and after follow-ups (p>0.05). All changes of GV and Ht-SDS in the BCD-T, BCD-C, and GHD-T groups were shown in figure1 and figure2. The rhGH dosage needed in the BCD-T group was significantly higher than that in GHD-T group (p<0.05). Insulin-like growth factor-1 (IGF-1) levels were both significantly increased in the BCD-T and GHD-T groups after rhGH treatment (p<0.05). No serious adverse reaction occurred during the follow-ups.

**Conclusions**: Though the effect of rhGH therapy is poorer in BCD patients compared with that in GHD patients, an appropriate dose of rhGH therapy is still helpful to improve the GV and Ht-SDS. The study of the long-term safety of rhGH treatment in BCD patients is still required.

**Key words**: short stature; bone and cartilage diseases; recombinant human growth hormone

**Fig. 1 (abstract O4-5).**
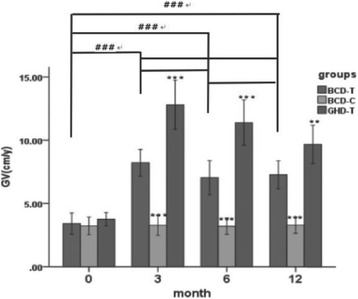
See text for description

## O4-6 A hybrid Fc-fused human growth hormone, GX-H9, shows a potential for semi-monthly administration in both adult and pediatric growth hormone deficiencies

### H. Michael Keyoung^1^, Aryaev Mykola^2^, Eun Jig Lee^3^, Jochen Schopohl^4^, Tae Kyung Kim^1^, Young-Joo Ahn^5^, Jung-Won Woo^1^, Woo Ick Jang^5^, Young- Chul Sung^1^

#### ^1^Department of pathology of the newborns and premature infants, Genexine, Inc.; ^2^Municipal Institution; ^3^Department of Internal Medicine IV, Ludwig-Maximilians University Munich; ^4^Yonsei University College of Medicine, Endocrinology/ Internal Medicine; ^5^Handok, Inc

GX-H9 is a hybrid Fc-based long-acting recombinant human growth hormone (hGH). The safety, tolerability, and PK/PD were assessed in a single ascending dose study in healthy volunteers and in multiple sequential dose studies in patients with adult (AGHD) and pediatric growth hormone deficiencies (PGHD). The efficacy of GX-H9 was compared to that of a daily recombinant hGH.

A double-blind, randomized, placebo-controlled, single ascending dose Phase 1 study of GX-H9 was conducted in 4 groups of healthy subjects (n=32) with four sequential dose levels (0.2, 0.4, 0.8 or 1.6 mg/kg). Currently, a Phase 2, randomized, active-controlled, open-label, sequential dose study of GX-H9 (0.1 mg/kg/weekly, 0.2 and 0.3 mg/kg/semi-monthly) is being conducted in patients with AGHD (n=45). In addition, a Phase 2, randomized, active-controlled, open-label, multiple dose study of GX-H9 with weekly and semi-monthly administrations is being conducted in patients with PGHD (n=48).

Single doses of GX-H9 were well tolerated at all dose levels in healthy volunteers. No safety concerns were noted, including absence of any lipoatrophy or anti-drug antibodies. Geometric mean of t1/2 ranged between 69.2 and 138.0 hours. IGF-1 serum concentrations increased in a dose-dependent manner between 0.2 and 1.6 mg/kg. The interim Phase 2 results in AGHD have indicated that administration of 0.1 mg/ kg/week and 0.3mg/kg/every other week with GX-H9 for 12 weeks were safe and efficacious. The weekly treatment of 0.1mg/kg in AGHD patients demonstrated the mean increase in IGF-1 comparable with those receiving 6 μg/kg of Genotropin® daily for 12 weeks (101.3 ± 31.2 ng/mL vs 109.1 ± 45.0 ng/mL, respectively). A limited interim analysis of PK and IGF-1 level in PGHD study was performed for the first 12 subjects after completing a single dose period, demonstrating the safety of GX-H9 and dose-dependent PK profile in pediatric patients. The administration of higher doses showed a potential for semi-monthly treatment of GX-H9 in both AGHD and PGHD. The data from ongoing Phase 2 studies will be presented.

## O5-1 Function study of NKX2-1 gene c.799G>T mutation, which caused congenital hypothyroidism and central nervous system disorders

### Shiyao Wang, Huijuan Zhu, Hui Pan, Hongbo Yang, Linjie Wang, Zimeng Jin, Fengying Gong

#### Department of Endocrinology, Key Laboratory of Endocrinology, National Health and Family Planning Commission, Peking Union Medical College Hospital, Chinese Academy of Medical Science

**Background**: NK2 homeobox 1 (NXK2-1) is a relatively common responsible gene in congenital hypothyroidism (CH). It plays a very important role in the development of thyroid, lung and central nervous system (CNS). We identified a de novo heterozygous NKX2-1 mutation c.799G>T (p.Val235Phe, NP_001073136.1) in a pair of twin boys who had primary CH and ataxia. As the most common NKX2-1 gene mutation, the pathologic mechanisms of the c. 799G>T mutation is still undetermined.

**Methods**: We introduced the mutation c.799G>T in an NKX2-1 expression vector, and then analyzed the protein expression differences in HEK293 cells between wildtype and mutant vectors through transfection experiments and Western blot. After co-transfected the expression vectors and reporter vectors harboring TG/TPO /SP-B gene promoters, we performed the dual-luciferase assay to evaluate the function of NKX2-1 c.799G>T heterozygous mutation. Furthermore, we also analyzed local structure variations of mutant NKX2-1 p.V235F using bioinformatics software.

**Results**: Compared with wildtype NKX2-1, the expression of mutant NKX2-1 p.V235F was significantly reduced in HEK293 cells. All TG, TPO, and SP-B promoters could be activated by wildtype NXK2-1. TG promoter was activated in a dose-dependent pattern, while TPO and SP-B promoters were not. All TG, TPO or SP-B promoters were failed to be efficiently activated by mutant NKX2-1 p.V235F. And dose changes of mutant NKX2-1 p.V235F didn’t change the transcription activity of TG, TPO or SP-B promoters induced by wildtype NKX2-1. Local structure analysis of mutant Phe235 showed abnormal contacts with nearby amino acids and Arg221. Surface charge distribution also changed in this mutant model.

**Conclusions**: NKX2-1 haploinsufficiency in TG promoter activation and decreased TG transcriptional activity induced by mutant NKX2-1 p.V235F may cause hypothyroidism in NXK2-1 c.799G>T patients. Our study discovered part of the pathologic mechanisms of NXK2-1 c.799G>T mutation.

**Keywords**: NKX2-1 related disorder; Congenital hypothyroidism; Thyroglobulin gene promoter; Thyroperoxidase gene promoter; Surfactant protein-B gene promoter

**Fig. 1 (abstract O5-1).**
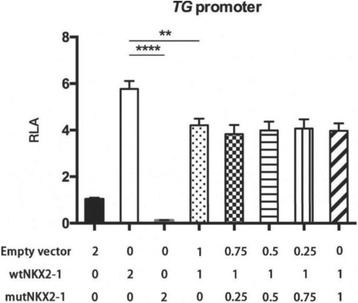
See text for description

## O5-2 A variety of clinical presentation in congenital hypothyroidism caused by mutations in the thyroglobulin gene

### Aoi Kawakita^1^, Yukiyo Yamamoto^1^, Kazuyasu Kubo^1^, Mami Eguchi^1^, Reiko Saito^1^, Motohide Goto^1^, Rinko Kawagoe^1^, Yasusada Kawada^1^, Koichi Kusuhara^1^, Satishi Narumi^2^, Tomonobu Hasagawa^3^

#### ^1^Department of Pediatrics, University of Occupational and Environmental Health; ^2^Department of Molecular Endocrinology, National Research Institute for Child Health and Development; ^3^Department of Pediatrics, Keio University School of Medicine

**Background**: Mutations in the thyroglobulin (TG) are important genetic causes of congenital hypothyroidism, characterized by congenital goiter and low serum TG. Importantly, these patients are at elevated risk for developing thyroid cancer. Here, we report clinical features of four cases with various TG gene mutations.

**Case report**: Case 1 was born with remarkable goiter and his TSH was high (80 μ U/ml) at neonatal mass screening (MS). At 10 day-old, thyroid function tests confirmed overt hypothyroidism with low serum TG (11.5ng/ml) and L-T4 replacement started. Ultrasonography showed remarkable goiter. At re-evaluation in 3 year-old, TRH-test showed elevated basal TSH and exaggerated response (13.34 → 97.02 IU/ ml). 123I scintigraphy showed high uptake (68.89%) and negative perchlorate discharge. Ultrasonography still exhibited thyroid enlargemant.

Case 2 and 3 are male siblings of 14 and 12 year-old at first visit to our hospital. Their TSH levels were high at MS (Case 2: 110.1 μ U/ml,

Case 3: 64.3 μ U/ml). Thyroid function tests confirmed overt hypothyroidism and L-T4 replacement started. Serum TG and thyroid size were not evaluated at neonatal period. At 14 and 12 year-old, they had no goiter and examinations revealed euthyroid status with undetectable serum TG.

Case 4 is 40 day-old at first visit to our hospital. His TSH level was high (13.2 μ U/ml) at MS. Thyroid function tests confirmed overt hypothyroidism and L-T4 replacement started. No data about serum TG and thyroid size at neonatal period was available. At 40 day-old, he had no goiter and examinations revealed euthyroid status with normal TG level (47.2 ng/ml). At re-evaluation in 3 year-old, TRH-test showed elevated basal TSH and exaggerated response (17.39 → 109.3 IU/ml). 123I scintigraphy showed high uptake (54.86%) and negative perchlorate discharge. His thyroid size was normal in ultrasonography.

Genetic analysis identified heterozygous mutations in the TG gene (Case 1; c.4177A>T, pK1393X, c.3790T>C, pC1264R, Case 2 and 3; c.4177A>T, p.K1393X, c.4235delT, p.L1412fs, Case 4; c.1006T>C, p.C336A, c.7006C>T, p.R2317X).

**Discussion**: Cases with TG mutations have exhibited variability in clinical presentation. Goiter was often not evaluated at neonatal period and serum TG did not decrease in some cases. Careful diagnosis of goiter by ultrasonography should be performed. High uptake in 123I scintigraphy and negative perchlorate discharge are important findings at re-evaluation. According to these findings, TG mutations should be considerd even in cases with no goiter and normal serum TG. Active detection of TG mutation is expected to decrease future thyroid cancer risk.

## O5-3 Genetic and imaging analyses of sibling cases with congenital hypothyroidism revealed the distinct clinical courses despite the presence of the identical mutation

### Mikiko Koizumi^1^, Shinsuke Onuma^1^, Mariko Nakacho^1^, Yasuko Syoji^1^, Masanobu Kawai^1^, Yuri Etani^1^, Shinobu Ida^1^, Chiho Sugisawa^2^, Kiyomi Abe^2^, Satoshi Narumi^2^, Tomonobu Hasegawa^2^

#### ^1^Department of Gastroenterology and Endocrinology, Medical Center and Reserch Institute for Maternal and Child Health; ^2^Department of Pediatrics, Keio University

**Introduction**: Mental retardation is a severe complication of congenital hypothyroidism (CH) and preventable by early diagnosis and therapeutic intervention. More than half of the cases with thyroid dyshormonogenesis, which accounts for 15 % of CH, is caused by the mutation of a single gene. Sibling cases of CH are occasionally observed and clinical courses are sometimes different among them. In the current study we performed imaging and genetic analysis in 7 patients from three familial cases of CH to understand the association between pathogenesis of CH and their clinical courses.

**Subjects and methods**: Seven patients from three familial cases of CH were recruited in the study. The type and cause of hypothyroidism was determined based on radioisotope scanning with the perchlorate discharge test and ultrasonograph. Genomic DNA was extracted from peripheral blood and genetic analysis was performed using targeted next-generation sequencing. The identified mutation was confirmed using the Sanger sequencing method.

**Results**: Family 1: A 19-year-old man was positive for neonatal mass screening (NMS) and diagnosed as having CH. His 15-year-old brother was negative for NMS, but subsequently diagnosed as CH at 4-month old, and synthetic thyroxin was initiated. Iodine transport defect was noted and a heterozygous mutation in the NIS gene was identified in both patients.

Family 2: A 14-year-old boy was positive for NMS and diagnosed as CH. His 11-year-old brother was also positive for NMS, but synthetic thyroxin was not initiated as TSH levels declined and thyrotropin levels were maintained within normal range. Thyroid iodine organification defect was detected and homozygous mutation in DUOX2 gene was identified in the former case, whereas a heterozygous mutation was present in the latter case.

Family 3: The proband is a 17-year-old girl. She was positive for NMS and diagnosed as CH. Her 14- and 10-year old brothers were negative for NMS, but subsequently diagnosed as subclinical CH. Thyroid hypoplasia was noted and no mutations were identified in the proband.

**Discussion and Conclusion**: Early therapeutic intervention is critical to improve the intellectual outcomes in CH patients; however, NMS sometimes fails to detect CH especially when the hypothyroidism is trivial. Here we present a sibling case of CH (Family 1) who followed different clinical courses despite the presence of identical mutation, suggesting the importance to monitor the thyroid function in children when there exists CH sibling(s) in the family even if the NMS test was negative.

## O5-4 Comprehensive analysis of seven Toll-like receptor genes with autoimmune thyroid disease in Korean children: Sexual dimorphism in polymorphisms of TLR 4 gene might influence female predominance of AITD

### Won Kyoung Cho^1^, Jung-Pil Jang^2^, Moonbae Ahn^1^, Min Ho Jung^1^, Tai-Gyu Kim^2,3^, Byung-Kyu Suh^1^, Shin Hee Kim^1^, Kyoung Soon Cho^1^, So Hyun Park^1^

#### ^1^Department of Pediatrics, College of Medicine, The Catholic University of Korea; ^2^Department of Microbiology, College of Medicine, The Catholic University of Korea; ^3^College of Medicine, The Catholic University of Korea, Catholic Hematopoietic Stem Cell Bank

**Background**: The Toll-like receptors (TLRs) are germline-encoded receptors that play an essential role in initiating the immune response against pathogens. In this study, we assess the association of TLR polymorphism with autoimmune thyroid disease (AITD) in Korean children.

**Methods**: Seven Toll-like receptor genes (TLR-1, -2, -3, -4, -5, -6, -9) including 15 single-nucleotide polymorphisms were analyzed on 104 Korean children with AITD [Hashimoto¢s disease (HD) = 40, Graves¢ disease (GD) = 60 (thyroid-associated ophthalmopathy (TAO) = 29, non- TAO = 31)] and 192 healthy individuals.

**Results**: The allele frequencies of 15 SNPs in AITD patients and controls are shown in Table 3. For overall AITD cases, the frequencies of these alleles had no statistical difference with controls. When categorized by disease subgroup, GD showed lower frequencies of the TLR4 rs1927911 T allele (cP <0.018) and HD showed an lower frequencies of the TLR3 (-7) rs3775296 C allele (cP <0.044) than the control group. Between GD and HD group, the frequencies of the TLR4 rs1927911 CC genotype in HD (cP < 0.048) was lower, whereas TLR4 rs1927911 T allele in HD (cP < 0.032) showed higher frequencies than in GD. When categorized GD by sex, female-GD group showed higher frequencies of the TLR4 rs1927911 CC genotype (cP < 0.026) and lower frequency of the TLR4 rs1927911 T allele (cP < 0.017) than control. Male-GD group showed lower frequency of the the TLR4 rs1927911 CT genotype (cP < 0.031) than control. Between female and male group in GD, the frequencies of TLR4 rs10759932 CC genotype (cP <0.006) and TLR4 rs1927911 TT genotype (cP < 0.024) in male were higher, whereas TLR4 rs10759932 T allele (cP < 0.004) and TLR4 rs1927911 C allele (cP < 0.016) in male were lower than female-GD.

**Conclusions**: Our results suggest that TLR- 3 and -4 gene polymorphisms may contribute to the pathogenesis of AITD and TAO. Sexual dimorphism in polymorphisms of TLR 4 gene might influence female predominance of AITD.

## O5-5 A Malaysian family with an activating mutation (S505R) in the Thyroid-stimulating Hormone Receptor (TSHR) gene

### Noor Shafina Mohd Nor^1^, Johari Mohd Ali^2^

#### ^1^Faculty of Medicine, Universiti Teknologi MARA (UiTM); ^2^Faculty of Medicine, University of Malaya, Department of Molecular Medicine

**Introduction**: Childhood hyperthyroidism occurs less commonly in children than hypothyroidism, yet far more symptomatic. Hyperthyroidism in children is mostly due to autoimmunity, predominantly as a result of Graves’ disease. Non-autoimmune hyperthyroidism caused by activating mutations in the Thyroid-stimulating Hormone Receptor (TSHR) gene occurs to a much lesser extent.

**Case report:** We report a Malay family with three affected individuals (a mother and her two daughters). The two sisters, aged 5 years and 1 month old and 2 years and 5 months old were under General Paediatricians follow up since infancy for symptomatic hyperthyroidism. Despite high dose of anti-thyroid medication (carbimazole), their thyroid function tests continued to be deranged (high fT4, suppressed TSH). They were then referred to our Paediatric Endocrinology clinic for further management. The family history revealed that the mother was presumed as having Graves’ disease since the age of 15 and had radioactive iodine therapy at the age of 24 years due to failure of medical treatment. She subsequently became hypothyroid and currently requiring daily L-thyroxine replacement. On examination, both siblings have palpable goiters with mildly prominent eyes. Thyroid antibodies screening were all negative. Thyroid ultrasound scan showed diffuse thyroid disease with lymphadenopathies. Bone ages were also advanced. In view of the findings, TSHR mutation analysis was done for the two siblings and the parents. Direct DNA sequencing of the TSHR gene revealed a heterozygous cytosine-to-adenine transversion in exon 10 in the two siblings and the mother. This missense mutation was not found in the father. The mutation leads to serine to arginine substitution, affecting TSHR codon 505 (S505R). The S505R mutation had previously been characterized and was observed to cause the activation of TSHR protein (gain- of-function mutation).

**Discussion**: In conclusion, the evidence of non-autoimmune hyperthyroidism in several family generations warrants initiation of mutation analysis to detect the possible mutation in the TSHR gene leading to the clinical entity. Affected family members harbouring the exact same mutation may exhibit variable phenotypes with regards to the age of onset and severity of hyperthyroidism.

**Consent for publication:** The authors declare that written informed consent was obtained for publication.

## O5-6 Allogeneic stem cell transplantation (HSCT) in childhood and thyroid cancer as the most frequent secondary solid tumor following HSCT with total body irradiation

### Marta Snajderova^1^, Petr Sedlacek^2^, Petra Keslova^2^, Renata Formankova^2^, Petr Riha^2^, Jan Stary^2^

#### ^1^Division of Endocrinology and Diabetes / Department of Pediatrics, 2nd Faculty of Medicine, Charles University in Prague and University Hospital Motol; ^2^Department of Pediatric Hematology and Oncology, 2nd Faculty of Medicine, Charles University in Prague and University Hospital Motol

**Backgroud**: Allogeneic hematopoietic stem cell transplantation (HSCT) is a potentially curative therapy for a variety of malignant and non- malignant disorders. Improved outcome leads to increasing attention to late complications in long-term survivors. Secondary cancer belongs to the most serious complications.

**Aims**: Occurrence of secondary solid tumors following allogeneic HSCT was analyzed.

**Methods**: We have evaluated clinical and laboratory data (including fT4, TSH, thyroid antibodies, thyroid function and ultrasound imaging) in 499 patients (315 M, 184 F) who underwent 545 allogeneic HSCT at a median age 8.4 years since 1989 till 2014. Of them 60% (300/499) patients are still alive and disease free.

**Results**: We have documented secondary malignancy in 29 patients (5.8%). Of them 13 developed post-transplant lymphoproliferative disorder at a median time 0.3 (0-1.8) years after HSCT. Secondary solid tumor was diagnosed in further 16 patients (3.2%) at a median time 11.4 (range 5.4-17.8) years after HSCT (thyroid carcinoma n=8, carcinoma of oral cavity n=3, malignant schwannoma n=2, melanoma n=1, peritoneal mesothelioma n=1, breast cancer n=1). All patients with secondary solid tumor underwent surgery and/or chemo-radiotherapy and are alive. 15/16 patients (93.8%) with secondary solid tumor received total body irradiation (TBI) 12-14.4 Gy as a part of conditioning regimen. Papillary carcinoma (in all 8 cases micronodular form, T1 or T2 stage) was the most frequent secondary solid tumor (5F, 3M; 50% of secondary solid tumors) diagnosed at a median time 10.8 years (range 5.4-17.0) after HSCT. At the time of diagnosis three patients were treated with thyroxine for autoimmune thyroid disease, one for hypothyroidism and another one for nodular goiter. All but one had HSCT for malignant disease and 7/8 received TBI.

**Conclusions**: The early diagnosis is one of the key tasks of long-life multidisciplinary post-transplant care including regular ultrasound evaluation of thyroid gland and neck especially more than 5 years after HSCT and namely after irradiation. The incidence of complications following allogeneic HSCT in childhood namely after TBI is increasing within time. Papillary thyroid carcinoma was the most frequent secondary solid tumor detected. Supported by MHCZ for conceptual development 00064203 University Hospital Motol.

## O6-1 First FGF9 mutation in a patient with a 46,XY disorder of sex development

### Makoto Ono^1,2^, Anthony Bird^2^, Stefanie Eggers^3^, Brittany Croft^2,3^, Stefan Bagheri-Fam^2^, Janelle Ryan^2^, Andrew Kueh^4^, Peter Stanton^2^, Tim Thomas^4^, Andrew Sinclair^3^, Masayo Harada^5^, Vincent Harley^2^

#### ^1^Department of Paediatrics, Tokyo Bay Urayasu Ichikawa Medical Centre; ^2^Hudson Institute of Medical Research, Clayton, Centre for Reproductive Health; ^3^Murdoch Childrens Research Institute, Parkville; ^4^Walter and Eliza Hall Institute of Medical Research; ^5^Department of Clinical Anatomy, Tokyo Medical and Dental University

**Background**: Disorders of sex development (DSDs) include 46, XY gonadal dysgenesis (GD), where a specific genetic diagnosis is made in only ~30% of patients. Improved understanding of the genetic causes of 46, XY GD is therefore required to better inform clinical diagnosis and management. Among the genes induced by upstream SRY-SOX9 signalling to promote male sex determination is fibroblast growth factor (FGF) 9. Expressed within the pre-Sertoli cell lineage, FGF9 suppresses the female program of gonadal development via its receptor FGFR2. In mouse both are critical for testis determination as FGF9/FGFR2 knockout mice show XY sex reversal. Despite this, to date no FGF9 gene mutations/deletions/insertions have been identified in human DSD patients.

**Results**: We identified an FGF9 variant, a maternally derived heterozygous single nucleotide substitution c.583G>A (p.D195N), in a 46,XY GD female presented with delayed puberty, primary amenorrhea, clitromegaly, Müllerian duct remnants and raised gonadotrophin levels using 1032 DSD gene targeted parallel sequencing. In silico analysis predicted the D195N variant to be deleterious for FGF9 protein function, and in vitro studies indicated the D195 residue lies at the homodimerisation interface. Purified recombinant FGF9-D195N protein showed a reduced affinity for heparin, a property necessary for stable FGF-FGFR complexes, and reduced ability to induce Sertoli cell proliferation in vitro . To model the D195N mutation in vivo , Fgf9D195N/+ knockin mice were generated via CRISPR/Cas9 gene-editing. Pilot studies showed that E15.5 Fgf9D195N/D195N embryonic XY gonads exhibit a truncated male-specific coelomic blood vessel, and immunofluorescence analysis revealed ectopic expression of the female meiotic marker γ H2AX, indicative of sex reversal. We also investigated gonadal development in Fgf9N143T/N143T mice which also carry a mutation in the FGF9 homodimerisation domain. Likewise, E15.5 Fgf9N143T/ N143T embryonic XY gonads show a truncated coelomic blood vessel and partial sex reversal.

**Conclusion**: These results suggest that FGF9 homodimerisation and heparin binding are required for FGF9 function in testes determination. In addition, human FGF9 mutations may be the cause of a subset of hitherto undiagnosed human DSD patients.

## O6-2 The etiology and differential diagnosis of 46,XYDSD of 88 cases

### Yan Song Ning, Chun gong Xiu

#### Beijing Children's Hospital affiliated to Capital Medical University, Endocrine Department

**Aims**: Retrospectively reviewed the etiology, clinical features and differential diagnosis of 88 46,XYDSD.

**Methods**: Collect from September 2008 to December 2015 in Beijing Children's Hospital and gene detection of 300 cases, selecting the diagnosed of 46, XYDSD of 88 cases except CHH that compared their clinical symptoms and check and laboratory examination.

**Result**: The social gender of the 88 case was that male: 38, female: 50 aged 4 months to 17 years old, average 3.71 years. Clinical diagnosis were androgen insensitivity syndrome (AIS) in 33 cases (37.5%), 5 alpha reductase deficiency (5 alpha RD) in 31 cases (35.2%), 17 alpha hydroxylase deficiency in 11 cases (12.5%); relatively rare in turn Nr5a1 gene mutation in 7 cases (8.0%), 17 beta hydroxysteroid dehydrogenase deficiency in 2 cases (2.3%), 3 beta hydroxysteroid dehydrogenase deficiency in 2 cases (2.3%), Fraiser syndrome (FS) and Denys Drash syndrome (DDS) in 1 case (1.1%). The main clinical manifestations of abnormal vulva: The external Prader classification of AIS, 5 alpha RD and 17 alpha hydroxylase in children showed no significant difference; and the rest of the rare case of Prader classification for 0-3. There was no significant difference in the distribution of testicular position in various diseases. In accordance with the hCG classification experiments between Leydig cell function in normal and interstitial function is not normal statistical grouping: interstitial cells of AIS and 5 alpha RD is normal, normal T/DHT, and between the two kinds of diseases were no difference; and 17 alpha hydroxylase deficiency, Nr5a1 gene mutation, 17 beta hydroxysteroid dehydrogenase deficiency, 3 beta hydroxysteroid dehydrogenase deficiency disease,'s and DDS showed interstitial cells are not normal after hCG stimulation.

**Conclusion**: There are many overlapping clinical manifestations, the differential diagnosis of 46, XYDSD was difficultiesThere were no differences between the Prader grading and testicular position of the body in different cause, but significant differences in the distribution of the different causes. Other rare disorders as 17 beta - hydroxy steroid dehydrogenase deficiency, 3 beta - hydroxy steroid dehydrogenase deficiency,'s and DDS require individual specific analysis. Internal genital organs in various diseases had no significant difference, pure sex hormone detection in diagnosis is unreliable .HCG stimulation test can divid into testicular Leydig cells normal and abnormal group, but specific disease is not easy to identify. T/DHT have disorders identification, for the above, when there is doubt, should take gene detection for further confirmed.

## O6-3 Mutation frequency in Japanese male patients with severe form of hypogonadotropic hypogonadism

### Takeshi Sato^1^, Satoshi Narumi^1,2^, Tomohiro Ishii^1^, Koji Muroya^3^, Yumi Asakura^3^, Masaki Takagi^4^, Masanori Minagawa^5^, Hiroaki Suzuki^6^, Chiho Sugisawa^7^, Jun Saito^7^, Yukihiro Hasegawa^4^, Masanori dachi^3^, Tomonobu Hasegawa^1^

#### ^1^Department of Pediatrics, Keio University School of Medicine; ^2^Department of Molecular Endocrinology, National Research Institute for Child Health and Development; ^3^Department of Endocrinology and Metabolism, Kanagawa Children's Medical Center; ^4^Department of Endocrinology and Metabolism, Tokyo Metropolitan Children's Medical Center; ^5^Department of Endocrinology, Chiba Children's Hospital; ^6^Department of Internal Medicine (Endocrinology and Metabolism), University of Tsukuba, Faculty of Medicine; ^7^Department of Endocrinology and Metabolism, Yokohama Rosai Hospital

**Background**: While previous studies have shown that 20–30% of Caucasian male hypogonadotropic hypogonadism (MHH) patients harbored causative gene mutations, genetic backgrounds of Japanese MHH patients have not been fully elucidated. Moreover, we do not know mutation frequency in MHH patients applicable to clinical practice.

**Aim**: To estimate mutation frequency in Japanese male patients with severe form of HH.

**Methods**: In this study, we defined MHH as male patients meeting all of the following criteria: (i) 18 years of age or older, (ii) no spontaneous testicular enlargement (volume ≥ 4 mL), (iii) low luteinizing hormone levels regardless of history of treatment for puberty induction and acquisition of fertility. We enrolled Japanese MHH probands, excluding CHARGE syndrome, combined pituitary hormone deficiency, and chromosomal abnormalities. We sequenced 32 genes implicated in HH using the MiSeq instrument. We defined mutations as variants meeting any of the following criteria: (i) variants reported as pathogenic, (ii) nonsense, frameshift, or splice site variants not observed in dbSNP, and Human genetic variation database, (iii) missense variants not observed in databases, conserved across species, and predicted as damaging by in silico analyses with Polyphen-2 and SIFT.

**Results**: Twenty male patients were included, ranging in age from 18 to 37 years, with a median age of 24 years. Nine out of 20 patients (45.0%, 95% confidence interval: 23.2–66.8%) harbored gene mutations; 4 FGFR1 , 4 KAL1 , and 1 SOX2. Oligogenic mutations were not identified.

**Discussion**: The proportion of detecting gene mutations in Japanese MHH patients was relatively high compared to Caucasian MHH patients, possibly due to our strict inclusion criteria. FGFR1 and KAL1 mutations were the two leading causes in Japanese MHH patients, which was consistent with the previous studies on Caucasian.

**Conclusion**: Mutation frequency in Japanese male patients with severe form of HH is 45.0%.

## O6-4 The incidence of intracranial lesions in central precocious puberty is overestimated

### Jong Seo Yoon^1^, Cheol Hwan So^1^, Hae Sang Lee^1^, Jung Sub Lim^2^, Jin Soon Hwang^1^

#### ^1^Division of Endocrinology and Metabolism / Department of Pediatrics, Ajou University School of Medicine; ^2^Division of Endocrinology and Metabolism / Department of Pediatrics, Korea Cancer Center Hospital

**Backgroud**: Intracranial pathology has been reported in 8% of girls and 40% of boys with central precocious puberty. So the current guidelines recommend that all boys with central precocious puberty and girls with central precocious puberty at under 6 years of age should have a brain MRI. And it is controversial whether all girls who develop central precocious puberty between 6 and 8 years of age require brain MRI.

**Objectives**: We evaluated the intracranial lesions in Korean girls and boys with central precocious puberty.

**Methods**: Total 369 patients (257 girls and 112 boys) diagnosed for central precocious puberty since 2003 at Ajou university Hospital underwent brain MRI. The patients were categorized into group A (normal findings) and group B (the other findings). We evaluated clinical and biochemical factors associated with precocious puberty and studied relationship between the factors and the outcome of their MRI.

**Results**: Group A included 238 among 257 girls (92.6%) and 105 among 112 boys (93.8%). Group B were detected in 19 among 257 girls (7.4%) and 7 among 112 boys (6.2%). Group B consisted of pituitary gland hyperplasia, Rathke’s cleft cyst , and pineal cyst ; 11 of 19 (4.3%), 5 of 19 (1.9%) and 4 of 19 (1.1%) in girls and 4 of 7 (4.3%), 2 of 7 (1.9%) and 1 of 7 (0.9%) in boys. None of group B had a pathological brain lesion associated with central precocious puberty. Girls in group B had higher mean basal LH levels (p 0.031) and stimulated LH levels (p 0.009) than girls in group A, and the statistical difference was particularly significant in pituitary gland hyperplasia. However, there was no statistically significant difference between two groups of boys.

**Conclusions**: Intracranial lesions were rare in girls and boys with central precocious puberty unlike the previously known incidence. So brain MRI should not be regarded as routine screening tools for evaluation of central precocious puberty. So we think that it is necessary to discuss again for the incidence of intracranial lesion in central precocious puberty.

## O6-5 Detection of somatic activating GNAS mutations in girls with isolated autonomous ovarian cyst by next generation sequencing

### Hironori Shibata^1^, Satoshi Narumi^1,2^, Tomohiro Ishii^1^, Koji Muroya^3^, Yumi Asakura^3^, Masanori Adachi^3^, Goro Sasaki^4^, Takumi Shibazaki^5^, Yosuke Hara^5^, Tomonobu Hasegawa^1^

#### ^1^Department of Pediatrics, Keio University School of Medicine; ^2^Department of Molecular Endocrinology, National Research Institute for Child Health and Development; ^3^Department of Endocrinology and Metabolism, Kanagawa Children's Medical Center; ^4^Department of Pediatrics, Tokyo Dental College Ichikawa General Hospital; ^5^Department of Pediatrics, Shinshu University School of Medicine

**Background**: McCune-Albright syndrome (MAS) caused by somatic activating GNAS mutations is characterized clinically by the classic triad of fibrous dysplasia (FD), café-au-lait skin spots, and GnRH-independent precocious puberty (PP) due to autonomous ovarian cyst. In a previous report, somatic activating GNAS mutations were found in 13 (33.3%) of 39 ovarian samples from girls with isolated autonomous ovarian cyst (J Clin Enocrinol Metab 2004; 89: 2107). In the same report, a series of nested PCR and restriction enzyme digestion detected somatic activating GNAS mutations in only 3 (7.7%) in 39 peripheral blood leucocytes (PBL) samples. We reported that next generation sequencing (NGS) detected somatic activating GNAS mutations sensitively from PBL samples in MAS (PLoS One 2013; 8: e60525).

**Objective**: To determine if we could detect somatic activating GNAS mutations in girls with isolated autonomous ovarian cyst by NGS using PBL samples.

**Method**: The study included 8 girls with GnRH-independent PP due to isolated autonomous ovarian cyst. We excluded cases with fibrous dysplasia (FD) and/or café-au-lait skin spots, the other classic triad of MAS. We performed both NGS and combinatory method of peptide nucleic acids (PNA) probe with NGS (PNA-NGS) using PBL samples from all patients.

**Results**: We detected somatic activating GNAS mutat ions in one (12.5%) by NGS and 5 (62.5%) by PNA-NGS of PBL samples (Table.1).

**Conclusion**: The combinatory method of PNA-NGS can detect somatic activating GNAS mutations sensitively from PBL samples in girls with isolated autonomous ovarian cyst. Our data suggest that somatic activating GNAS mutations is the major cause of isolated autonomous ovarian cyst.

**Table 1 (abstract O6-5).**
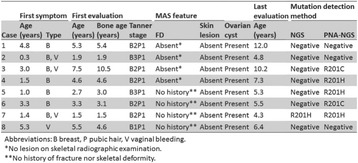
Characteristics of 8 girls with isolated autonomous ovarian cyst

## O6-6 Effects of cryptorchidism on testicular function in Chinese boys

### Liya Xu^1^, Wuhen Xu^2^, Pin Li^1^

#### ^1^Department of Pediatric Endocrinology, Shanghai Children's Hospital Affiliated to Shanghai Jiaotong University School of Medicine; ^2^Department of Labratory Medicine, Shanghai Children's Hospital Affiliated to Shanghai Jiaotong University School of Medicine

**Objective**: To investigate the effects of different types of cryptorchidism on testicular Leydig-cells and Sertoli-cells function in different ages of Chinese boys.

**Methods**: Included were 101 Chinese prepubertal cryptorchid boys and 136 controls (0.5-13 years). Those who have been operated were also included in case group(Orchiopexy is performed in 1-3yr). Each group was divided into five subgroups: 0.5-1year (yr), 1-3yr, 3-8yr, 8-11yr, 11-13yr. The patients were analyzed by age subgroups, unilateral vs bilateral and high-position vs low-position. LHRH & hCG stimulation tests, serum-Anti-Müllerian-Hormone(AMH) and inhibin-B(InhB) were examined.

**Results**: Effects of different ages on testicular function: Compared with other age subgroups in case group, testosterone-values after hCG stimulation tests was significantly higher in patients 0.5-1yr(p < 0.01); Compared with the control group, serum-AMH and InhB were significantly lower in the cryptorchid boys (*p*< 0.01). Especially, AMH-values in 1-8yr subgroups and InhB-values in 0.5-8yr subgroups were significantly lower (*p* < 0.01). Effects of high-position vs Low-position on testicular function: Compared with low-position group, testosterone-values after hCG stimulation tests in high-position group were significantly lower(*p*=0.007). Moreover, AMH-values and InhB values in high-position group were respectively significantly lower(*p*=0.001, *p*=0.012). Effects of unilateral vs bilateral on testicular function: Testosterone-values after hCG stimulation tests, AMH-values and InhB-values between unilateral and bilateral groups all have no significant difference (*p*>0.05).

**Conclusion**: 1. The Sertoli-cells function is firstly affected in cryptorchid boys before puberty, no matter orchiopexy has been performed or not, and InhB decreased earlier than AMH which occurs during 0.5-1yr, while AMH decreased more obviously after 1yr. However, the Leydig- cells function isn't severely affected in 1yr. 2.The effects of cryptorchidism on Leydig-cells and Sertoli-cells function are related to the issue of high or low-position, but not of unilateral or bilateral cryptorchidism.

## O7-1 Excessive gestational weight gain is associated with adverse health outcomes in the offspring at age 7 years

### Valentina Chiavaroli^1^, Jose’ G B Derraik^1^, Sarah A Hopkins^1^, Janene B Biggs^1^, Sumudu N Seneviratne^1^, Lesley M E McCowan^2^, Wayne S Cutfield^1^, Paul L Hofman^1^

#### ^1^University of Auckland, Liggins Institute; ^2^Department of Obstetrics and Gynaecology, Faculty of Medical and Health Science, University of Auckland

**Background**: Excessive gestational weight gain (GWtG) has been recognized as an important early-life risk factor for childhood obesity. We aimed to examine whether excessive GWtG was associated with alterations in body composition and metabolism in the offspring of primiparous mothers who participated in a randomised controlled trial of exercise regimen during pregnancy.

**Methods**: Of the initial 84 women and their offspring who participated in the trial, follow-up data were available on 52 mothers with GWtG information and their children. 35 mothers (67%) were determined to have excessive GWtG as per IOM guidelines, with the remaining 17 mothers having GWtG in the normal range. Note that the proportion of participants in each group that exercised during pregnancy was similar (60% and 54%, respectively). Children underwent clinical assessments at approximately 7.6 years, including body composition by DXA and fasting blood tests. All statistical analyses were adjusted for important confounders, in particular maternal BMI at trial recruitment (approximately 20 weeks of pregnancy).

**Results**: Birth weight SDS was not significantly different in the offspring of mothers with excessive or normal GWtG, when adjusted for maternal BMI (0.01 vs -0.23 SDS, respectively; *p*=0.34). Children born to mothers with excessive GWtG had a less favourable lipid profile with lower HDL (1.57 vs 1.75 mmol/L; *p*=0.038), higher triglycerides (0.87 vs 0.63 mmol/L; *p*=0.003), and higher total cholesterol to HDL ratio (3.06 vs 2.53 mmol/L; *p*=0.018), as well as increased abdominal adiposity (0.64 vs 0.55 android to gynoid fat ratio; *p*=0.043), even though macronutrient intake was similar in both groups. Boys born to mothers with excessive GWtG (n=19) were heavier (weight SDS 0.82 vs -0.01; *p*=0.010), had higher BMI SDS (0.35 vs -0.41 SDS; *p*=0.012) and greater abdominal adiposity (0.62 vs 0.45 android to gynoid fat ratio; *p*=0.010) than those born to mothers with normal GWtG (n=9). There was a small number of girls with metabolic data, but girls born to mothers with excessive GWtG (n=9) had higher fasting insulin concentrations (8.0 vs 5.4 mU/L; *p*=0.033) and were more insulin resistant (HOMA-IR 1.72 vs 1.16; *p*=0.048) than those born to mothers with normal GWtG (n=5), despite adjustment for body fat.

**Conclusions**: Independently of maternal BMI early in pregnancy, excessive gestational weight gain is associated with adverse health outcomes in the offspring at approximately 7.6 years of age, with some indication of sex-dependent effects.

## O7-2 Growth seasonality during preschool childhood in nursery school children

### Tsuyoshi Isojima^1,2^, Noriko Kato^3^, Susumu Yokoya^4^, Toshiaki Tanaka^5^, Atsushi Ono^6^, Hiroshi Yokomichi^7^, Zentaro Yamagata^7^, Soichiro Tanaka^8^, Hiroko Matsubara^9^, Mami Ishikuro^10,11^, Masahiro Kikuya^10,11^, Shoichi Chida^12^, Mitsuaki Hosoya^6^, Shinichi Kuriyama^9,10,11^, Shigeo Kure^8,10^

#### ^1^Department of Pediatrics, Teikyo University School of Medicine; ^2^Department of Pediatrics, Graduate School of Medicine, The University of Tokyo; ^3^Department of Early Childhood and Elementary Education, Jumonji University; ^4^Department of Medical Subspecialties, National Center for Child Health and Development; ^5^Department of Pediatrics, Japanese Association for Human Auxology; ^6^School of Medicine, Fukushima Medical University; ^7^Department of Health Sciences, Interdisciplinary Graduate School of Medicine and Engineering, University of Yamanashi; ^8^Department of Pediatrics, Graduate School of Medicine, Tohoku University; ^9^Department of Disaster Public Health, International Research Institute of Disaster Science (IRIDeS), Tohoku University; ^10^Tohoku Medical Megabank Organization (ToMMo), Tohoku University; ^11^Department of Molecular Epidemiology, Graduate School of Medicine, Tohoku University; ^12^Department of Pediatrics, School of Medicine, Iwate Medical University

**Background**: Healthy weight children tend to increase their weight during winter and decrease weight gain during summer. In contrast, accelerated summer weight gain has been observed among elementary overweight children. It is of great concern whether this seasonal variation occurs during preschool childhood. However, few studies examined growth seasonality during preschool childhood, and studies using longitudinal data are scarce.

**Methods**: The study population was derived from the nationwide retrospective cohort of nursery school children born from April 2, 2004, to April 1, 2005. We could gather ten consecutive longitudinal data of 15,259 children on height and weight measured in April and October from 2006 to 2010. With these data, we calculated six-month growth of these children in height, weight, and body mass index (BMI). Growth from April to October was defined as the summer period growth, while that from October to next April as the winter period growth. We analyzed longitudinal seasonal variation of nursery school children by classifying children into six groups according to the standard deviation score (SDS) of the last measurement just before they entered an elementary school (i.e. <-2SD, -2SD~ -1SD, -1SD~ 0SD, 0SD ~ +1SD, +1SD ~ +2SD, +2SD).

**Results**: Distinctive seasonal growth variation in weight and BMI was detected in the two groups of children with one or more than one BMI SDS (figure). They had accelerated summer weight and BMI gains and they could be detected from 2007 (p<0.0001). Notably, they had begun since less than three years of age in children with two or more than two BMI SDS. On the other hand, such growth seasonality was not observed in height.

**Conclusions**: Seasonal growth variation in weight and BMI was detected in relatively overweight preschool children during infancy. These findings suggested that we should be careful of summer time weight gain even in infancy for preventing the future obesity.

## O7-3 Oleate protects INS1 cells from palmitate-induced apoptosis by down-regulating ER stress and GSK3βthrought Stearoyl- CoA Desaturase 1

### Shan Huang^1^, Cai-qi Du^1^, Wei Wu^1^, Qin Ning^2^, Xiao-Ping Luo^1^

#### ^1^Department of Pediatrics, Tongji Hospital. Tongji Medical College , Huazhong University of Science and Technology; ^2^Department of Infectious Diseases, Tongji Hospital. Tongji Medical College , Huazhong University of Science and Technology

**Backgroud**: The loss of functional β -cell mass, at least in part secondary to increased β -cell apoptosis, is increasingly recognized as one of the main contributing factors to the pathogenesis of type 2 diabetes. Our previous study have identified that endoplasmic reticulum (ER) stress and GSK3 β involved in palmitate-induced β -cell apoptosis (lipotoxicity). However, the effect of oleate on β -cell is still controversial.

**Objective**: The present study investigated whether oleate protected β -cell from palmitate-induced apoptosis and its molecular mechanisms.

**Methods**: INS1 cells were cultured with varying concentrations of palmitate, oleate, TO-901317 or co-treatment of palmitate with either oleate or TO-901317. Cell viability and apoptosis were measured by CCK-8 assay, Hoechst 33342 / PI, flow cytometric assay and electron microscopy; ER stress and GSK3 β activity were assessed by RT-PCR and western blot. We also estimated intracellular triglyceride accumulation through nile red staining and electron microscopy.

**Results**: Contrary to lipotoxicity of palmitate, 0.25~0.5 mM oleate increased INS1 cells viability (p <0.05). Interesting, we identified that oleate rather than palmitate induced a significant intracellular triglyceride accumulation (p <0.05). Furthermore, co-treatment of oleate with palmitate apparently decreased palmitate-induced apoptosis by down-regulating ER stress and GSK3 β activity (p <0.05), Besides, we also found that the mRNA level of Stearoyl-CoA Desaturase 1 (SCD1) was up-regulated (p <0.05). Finally, co-treatment of Palmitate with LXR- agonist TO-901317, which directly up-regulated SCD1, also leaded to intracellular triglyceride accumulation and the reduction of ER stress and GSK3 β activity.

**Conclusion**: Oleate protected INS1 cells from palmitate-induced apoptosis by down-regulating ER stress and GSK3 β activity throught promotion of appropriate triglyceride accumulation , which related to the up-regulation of SCD1.

## O7-4 Increase of body mass index from age 1.5 to 3 years is useful in screening for a high risk of childhood obesity

### Go Ichikawa^1,2^, Junko Ichikawa^1^, Ayako Yoshida^1^, Satomi Koyama^1^, Osamu Arisaka^1^

#### ^1^Department of Pediatrics, Dokkyo Medical University; ^2^Department of Pediatrics, Nasu Red Cross Hospital

**Introduction**: Childhood obesity is routinely described as a major problem in developed and developing countries. Obesity in childhood is causative for many chronic diseases, including type 2 diabetes, cardiovascular disease and hypertension. Once obesity is present, it is challenging to treat because of multiple physiological, behavioral, and cultural feedback loops. Prevention is the key to success in obesity control, but there is no effective method for screening for high risk cases. Therefore, this study was performed to develop a method for detection of a high risk of childhood obesity.

**Methods**: A total of 298 children in a birth cohort (town F, Tochigi, Japan) were enrolled in the study. The population of this town is 18,000, with half of the people working as farmers and half commuting to nearby large cities. Children in the study were followed up in infant health checks at a health center during the preschool period, and data were stored at a regional health center. At 12 years of age, measurement of height and weight were performed at school. Children at this age with body mass index (BMI) ≥ 95th percentile and ≥ 85th percentile according to national reference data for height and weight were diagnosed as obese and overweight, respectively. Several screening methods focusing on relationships with BMI at 1.5 and 3 years old were examined by computing the odds ratio for each method in multivariate analysis. The study was approved by the ethics committee of Dokkyo Medical University.

**Results**: Data were available for 215 children. The odds ratios for obesity and an overweight status at 12 years old were 18.9 (95% confidence interval 5.9-60.8, predictive accuracy 0.92) and 16.9 (5.1-55.9, 0.84), respectively, for a BMI increase 0.5 from 1.5 to 3 years and BMI 16.8 at 3 years; 3.1 (1.2-8.2, 0.70) and 2.4 (1.2-4.9, 0.68), respectively, for a BMI increase from 1.5 to 3 years; and 5.8 (2.1-16.0, 0.84) and 3.6 (1.6-8.2, 0.78) for a BMI increase 0.5 from 1.5 to 3 years.

**Discussion**: The current study is the first to show that children with both an increase of BMI 0.5 from 1.5 to 3 years old and BMI 16.8 at 3 years old are at high risk of later childhood obesity or an overweight status. Identification of individuals at 3 years old who are at high risk for obesity later in life may provide opportunities for early interventions.

## O7-5 Cardiovascular risk factorsin children with type 1 diabetes

### Ying Zhang, Pin Li, Guo Sheng

#### Division of Endocrinology, Shanghai children's hospital of Shanghai Jiaotong University

**Background**: Cardiovascular disease as a result of atherosclerosis is the major complication among children with type 1 diabetes mellitus (T1DM). The aim of this prospective study was toexamining the presence of cardiovascular risk factors in children with T1DM.

**Methods**: We evaluated several cardiovascular risk factorsincluding atherosclerosis, artery intima-media thickness(IMT) and metabolic responsesin 122 (66 female) diabetic children (age median 12.6 ± 2.7 years, mean HbA1c of 7.9 ± 1.1%) with T1DM, and 105 non-diabetic children (age median 12.5 ± 2.8 years) were enrolled as normal controls.

**Results**: The diabetic children had significantly higher carotid intima-media thickness (cIMT) and aorticIMT (aIMT) than the controls, meanwhile, the diabetic patientsalso had significantly higher values than the controls for diastolic wall stress (DWS), incremental elastic modulus (IEM) and for flow mediated dilatation (FMD). Tumor necrosis factor- α (TNF- α), Interleukin -4 (IL-4), high-sensitivity C-reactive protein (hs-CRP) and leptin were all significantly greater as compared to T1DM and controls. The difference was not significant between patients and controls for othercardiovascular risk factors.In children with T1DM, cIMTandaIMTwere both correlated with several risk factors includingage, weight, body mass index (BMI), duration of diabetes,waist/hip ratio, total cholesterol, triglyceridesand apolipoprotein B, in addition to common risk factors, cIMTwas also associated with systolic blood pressure (BP). Inflammatory factor TNF- α was just dependently associated with weight, duration of diabetes, total cholesterol, triglycerides and apolipoprotein B. Other risk factors such as height, diastolic BP, LDL/HDL-cholesterol, apolipoproteinA TandS-creatinine level were all not independent risk factors ofcardiovascular diseasein T1DMchildren.

**Conclusions**: Our results suggested that children patients with T1DM are associated with early impairment of the common carotid and aortic artery structure and function and that diabetic state may be the main risk factor for artery wall stiffening and thickening, which are of considerable concern as possible early events in the genesis of atheroma.

## O7-6 Research on the role which FTO plays on the regulation of PI3K, MAPK pathway

### Peng li Bao, Ge Li Liu

#### Pediatrics, General Hospital of Tianjin Medical University

**Objective**: FTo explore if the the change of the expression of FTO regulate the PI3K and MAPK pathways, and finally destroy the endothelial function.

**Methods**: Using RNA interference technology to build FTO-shRNA lentivirus vector, infect HUVEC cells to silence the FTO gene and to up-regulate the expression of FTO, then use Western Blot to prove the effect. To detect the phosphorylation levels of ERK1/2, Akt CS6K1 and eNOS, NO level was detected by nitrate reductase method.

**Results**: Recombinant plasmid was constructed successfully. Silence of the FTO gene leads to the phosphorylation level decreasing of Akt, eNOS, ERK, S6K1 and decreased NO production. Over expression of FTO results to PI3K and MAPK pathway activated, Akt and eNOS, ERK1/2, S6K1 phosphorylation levels increased and NO synthesis more.

**Conclusion**: FTO expression can regulate PI3K and MAPK pathway, suggesting that PI3K-Akt-eNOS pathway and ERK/MAPK pathway play an important role in endothelial injury leadsby obesity, providing new direction for research on Mechanism of cardiovascular function destroy results by obesity.

## O8-1 Clinical Trial of Asfotase Alfa in Patients with Hypophosphatasia (HPP) in Japan

### Taichi Kitaoka^1^, Toshihiro Tajima^2^, Keisuke Nagasaki^3^, Toru Kikuchi^3^, Katsusuke Yamamoto^4^, Toshimi Michigami^5^, Satoshi Okada^6^, Ikuma Fujiwara^7^, Masayuki Kokaji^8^, Hiroshi Mochizuki^9^, Tsutomu Ogata^10^, Koji Tatebayashi^11^, Atsushi Watanabe^12^, Shuichi Yatsuga^13^, Takuo Kubota^1^

#### ^1^Department of Pediatrics, Osaka University Graduate School of Medicine; ^2^Department of Pediatrics, Hokkaido University School of Medicine; ^3^Division of Pediatrics / Department of Homeostatic Regulation and Development, Niigata University Graduate School of Medical and Dental Sciences; ^4^Department of Pediatric Nephrology and Metabolism, Osaka Medical Center and Research Institute for Maternal and Child Health; ^5^Department of Bone and Mineral Research, Osaka Medical Center and Research Institute for Maternal and Child Health; ^6^Department of Pediatrics, Hiroshima University Graduate School of Biomedical & Health Sciences; ^7^Department of Pediatrics, Tohoku University School of Medicine; ^8^Department of Pediatrics, Showa General Hospital; ^9^Division of Endocrinology and Metabolism, Saitama Children's Medical Center; ^10^Department of Pediatrics, Hamamatsu University School of Medicine; ^11^Depatment of Pediatrics, Nagara Medical Center; ^12^Division of Clinical Genetics, Nippon Medical School Hospital; ^13^Department of Pediatrics and Child Health, Kurume University School of Medicine

**Background**: Hypophosphatasia (HPP) is a rare skeletal disease caused by mutations in the gene coding the tissue-nonspecific alkaline phosphatase (TNSALP). The most severe type of HPP is often associated with respiratory failure due to the defect of calcification. Other types of HPP also have many complications including failure-to-thrive, hypercalcemia, premature loss of teeth and bone deformity. Recently, an enzyme replacement therapy using Asfotase Alfa (AA), a bone-targeted recombinant human TNSALP, has been developed to ameliorate the complications of HPP.

**Methods**: In an open-label, single-arm, multi-centered clinical study, we evaluated the safety and efficacy of treatment with AA in patients with HPP. We assessed the safety of repeated subcutaneous injections of AA as primary outcome measures along with the number of adverse events (AEs) including injection reactions. Efficacy assessments as secondary outcome measures included overall survival, respiratory status, rickets severity, physical growth, and gross motor development.

**Results**: Thirteen patients, 9 females and 4 males, were enrolled in this study; they ranged in age at baseline from day 0 to 34 years. Six patients had perinatal form, five had infantile form, one had childhood form, and one had adult form. Total 195 AEs reported, and the most frequent were injection site reactions (45%). Serious AEs that possibly related to the treatment were convulsion and hypocalcemia observed in the patients of prenatal type. In contrast, hypercalcemia and/or hyperphosphatemia were also observed in 3 patients of infantile form, and low calcium and/or low phosphorus formula started to these patients. As the efficacy of the treatment, all the patients survived. While total 8 patients required some forms of respiratory support at the baseline, 5 of them were free from respiratory support including oxygen supply at the end of the study. Baseline radiographs in all the patients except 2 patients with closed growth plate showed undermineralized metaphyses observed as hypophosphatasia-associated skeletal lesions. Mineralization was gradually improving with all the patients. Gross motor milestone was evaluated in 10 infants. Developmental milestones were also improved after the treatment.

**Conclusions**: The clinical trial revealed the improvement of skeletal, respiratory, and physical symptoms in patients with HPP by AA. Careful monitoring of serum calcium and phosphate levels is necessary because hypocalcemia, hypercalcemia, and hyperphosphatemia may occur. (ClinicalTrials.gov number, NCT02456038; UMIN CTR number, UMIN000014816).

## O8-2 BGN Mutations in X-Linked Spondyloepimetaphyseal Dysplasia

### Sung Yoon Cho^1^, Jun-Seok Bae^2^, Nayoung K.D. Kim^2^, Francesca Forzano^3^, Katta Mohan Girisha^4^, Dongsup Kim^5^, Kyoung Yeul Lee^5^, Shiro Ikegawa^6^, Noriko Miyake^7^, Gen Nishimura^8^, Andrea Superti-Furga^9^, Jürgen Spranger^10^, Ok-Hwa Kim^11^, Woong-Yang Park^2^, Dong-Kyu Jin^1^

#### ^1^Department of Pediatrics, Samsung Medical Center, Sungkyunkwan University School of Medicine; ^2^Samsung Genome Institute, Samsung Medical Center, Sungkyunkwan University School of Medicine; ^3^Division of Medical Genetics, Galliera Hospital, Via Volta 6; ^4^Department of Medical Genetics, Kasturba Medical College, Manipal University; ^5^Department of Systems Biology, Korea Advanced Institute of Science and Technology; ^6^Laboratory for Bone and Joint Diseases, RIKEN Center for Integrative Medical Sciences; ^7^Department of Human Genetics, Yokohama City University Graduate School of Medicine; ^8^Department of Pediatric Imaging, Tokyo Metropolitan Children's Medical Center; ^9^Department of Pediatrics, Centre HospitalierUniversitaire Vaudois, University of Lausanne (CHUV); ^10^Im Fuchsberg 14; ^11^Department of Radiology, Woorisoa Children's Hospital

Spondyloepimetaphyseal dysplasias (SEMDs) comprise a heterogeneous group of autosomal-dominant and autosomal-recessive disorders. An apparent X-linked recessive (XLR) form of SEMD in a single Italian family was previously reported.We have been able to restudy this family together with a second family from Korea by segregating a severe SEMD in an X-linked pattern. Exome sequencing showed missense mutations in BGN c.439A>G (p.Lys147Glu) in the Korean family and c.776G>T (p.Gly259Val) in the Italian family; the c.439A>G (p.Lys147Glu) mutation was also identified in a further simplex SEMD case from India. Biglycan is an extracellular matrix proteoglycan that can bind transforming growth factor beta (TGF-b) and thus regulate its free concentration. In 3-dimensional simulation, both altered residues localized to the concave arc of leucine-rich repeat domains of biglycan that interact with TGF-b. The observation of recurrent BGN mutations in XLR SEMD individuals from different ethnic backgrounds allows us to define “XLR SEMD, BGN type” as a nosologic entity.

**Fig. 1 (abstract O8-2).**
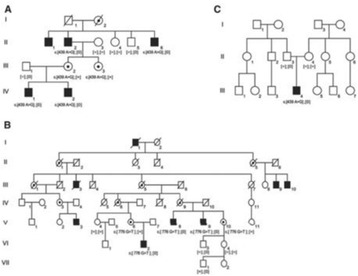
See text for description

**Table 1 (abstract O8-2).**
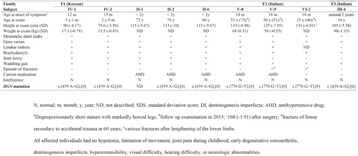
Clinical Findings of the Affected Individuals with XLR SEMD BGN Type

## O8-3 Effect of high dose monthly maternal cholecalciferol supplementation during breastfeeding on infant and maternal vitamin d status at 5 months post-partum: a randomized controlled trial

### Benjamin J Wheeler^1,2^, Barry J Taylor^1^, Peter Herbison^3^, Jillian J Haszard^1^, Adel Mikhail^1^, Shirley Jones^1^, Michelle J Harper^4^, Lisa A Houghton^4^

#### ^1^Department of Women's and Children's Health, Dunedin School of Medicine, University of Otago; ^2^Southern District Health Board, Paediatric Endocrinology; ^3^Dunedin School of Medicine, University of Otago, Preventive and Social Medicine; ^4^Department of Human Nutrition, University of Otago

Many countries recommend daily infant vitamin D supplementation during breast feeding, but compliance is often poor. A monthly, high dose maternal regimen may offer an alternative strategy for dual maternal and infant supplementation, but its efficacy is unknown.

In a New Zealand population not offered routine vitamin D supplementation, we aimed to determine the effect of two different monthly maternal doses of cholecalciferol (D3) on both maternal and infant 25-hydroxyvitamin D (25[OH]D) status during the first 5 months of breastfeeding.

Using a randomized, double blind, placebo-controlled design, breastfeeding women (n=90, mean age 32.1 years) were assigned to receive either placebo, 50,000 IU, or 100,000 IU of D3 /mo from Week 4 to Week 20 postpartum. Change in maternal and infant serum 25(OH)D from baseline to week 20 was calculated for each group and analysed using ANCOVA.

At 20 weeks, changes in maternal serum 25(OH)D were significantly greater in those assigned to D3 versus placebo (P<0.05), mean ± SD 17.3 ± 32.7, 30.2 ± 25.9, and 40 ± 29.3 nmol/L in the placebo, 50,000 IU, and 100,000 IU groups, respectively. Mean changes in infant serum 25(OH)D were 37.4 ± 51.9, 44.7 ± 44.7, and 52.8 ± 46.4 nmol/L in the placebo, 50,000 IU, and 100,000 IU groups respectively; however, these were not significantly different between groups (p>0.1). After adjustment for season of birth, vitamin D-fortified formula intake, and infant skin colour, the effect size for the 100,000 IU group was 19.1 nmol/L (95% CI 2.5 to 35.6; p=0.025) higher compared to placebo. For infants, maternal D3 supplementation at a dose of 100,000 IU /mo during the first 5 months of breastfeeding potentially benefits vitamin D status. While attractive from a compliance perspective, further studies are required to determine optimum dose and dosing frequency before this type of strategy is recommended as an alternative to daily infant supplementation.

## O8-4 Establishment of a human growth plate model with iPS cell-derived cartilage

### Takeshi Kimura^1,2^, Kie Yasuda^1^, Yoko Miyoshi^1^, Akihiro Yamashita^2^, Noriyuki Tsumaki^2^, Keiichi Ozono^1^

#### ^1^Department of Pediatrics, Graduate School of Medicine, Osaka University; ^2^Department of Cell Growth and Differentiation, Center for iPS Cell Research and Application, Kyoto University

**Background**: Endochondral ossification in the growth plate cartilage (GPC) determines the future length and shape of long bones. Several skeletal dysplasias cause growth plate dysfunction and result in severe short stature. There have been many studies on mice GPC function and development, but little is known about human GPC. We have already reported that human iPS cell-derived cartilage (hiPSC-Cart), when implanted into the subcutaneous spaces of the SCID mice for one year, formed skeleton composed of epiphyseal cartilage- and diaphyseal bone-like structure.

**Aim**: The aim of this study is to establish a practical hiPSC-GPC model for investigation about human growth and disease modeling.

**Method**: We used four hiPSC lines derived from healthy control and patients with skeletal dysplasias (thanatopholic dysplasia, achondroplasia and hypochondroplasia). hiPSC-Carts were transplanted into the subcutaneous space of SCID mice at various ages. Their ossification was observed with X-ray images over time and evaluated histologically.

**Result**: Histological analysis showed that transplanted hiPSC-Carts have a zonal arrangement similar to GPC, and type X collagen, a specific marker of hypertrophic chondrocytes, is positive in the transition between cartilage and bone. Furthermore, the ossification of hiPSC-Carts is promoted in younger mice. For example, GPC-like structures are formed 4 weeks after transplantation into 4 weeks-old mice. Epiphyseal cartilage-like structures express human vimentin, but other skeletal components do not. These results collectively suggest that endochondral ossification of hiPSC-Carts depends on regulation inside the host. Interestingly, we observed abnormal GPC-like structures in the patient- specific iPSC-Carts. The hypertrophic zones of these disease models showed irregular structures and cell sizes that were smaller than healthy control samples. These changes reflected the severity of the original diseases.

**Conclusion**: We have established an in vivo hiPSC-GPC model. The model likely contributes to further investigation for human growth.

## O8-5 Vitamin D Deficiency and its Relationship with Cardiac Iron and Function in Patients with Transfusion-Dependent Thalassemia at Chiang Mai University Hospital

### Prapai Dejkhamron^1^, Karn Wejaphikul^1^, Tuanjit Mahatumarat^1^, Suchaya Silvilairat^1^, Pimlak Charoenkwan^1^, Suwit Saekho^2^, Kevalee Unachak^1^

#### ^1^Department of Pediatrics, Faculty of Medicine, Chiang Mai University; ^2^Department of Radiological Technology, Faculty of Associated Medical Sciences, Chiang Mai University

**Background**: Vitamin D deficiency (VDD) is common in patients with thalassemia. There were conflicting findings on relationship between VDD and cardiac iron. Previous study reported that VDD may be associated with iron-related cardiomyopathy plausible linking L-type calcium channel activity to cardiac iron loading in these patients. Chronic heart failure was also well described in VDD. Parathyroid hormone (PTH) was positively correlated to cardiac iron in patients with thalassemia. However, a recent study was unable to identify the association between vitamin D and cardiac iron.

**Objectives**: We aim to determine the prevalence of VDD and to explore the impact of VDD on cardiac iron and cardiac function in patients with transfusion-dependent thalassemia (TDT).

**Method**: A prospective cohort study in patients with TDT (>12 transfusions/year), aged 8-25 years, who were followed at Chiang Mai University Hospital during Sep 2013-March 2015 was conducted. Patients with liver disease, renal disease, type 1 diabetes, malabsorption, hypercortisolism, malignancy, and contraindication for magnetic resonance imaging (MRI), were excluded. Calcium, phosphate, alkaline phosphatase (ALP), parathyroid hormone (PTH), 25-hydroxy vitamin D (25(OH)D) were measured. The mean hemoglobin level in the year preceding the study was calculated. Additionally, cardiac iron (cardiac T2*) was determined by MRI, and left ventricular ejection fraction (LVEF) was evaluated by echocardiography.

**Results**: Sixty-one (33M/28F) patients with TDT were enrolled. The prevalence of VDD was 50.8% (31/61). The mean hemoglobin in the last 12 months and the mean ferritin during the past 5 years were significantly correlated with 25(OH)D level (p=0.026 and p=0.026, respectively). Patients with severe cardiac iron deposition (T2* ≤ 20 ms) had tendency lower 25(OH)D than those with cardiac T2*>20 ms (15.5 ± 4.7 vs 20.7 ± 7.0; p=0.065). The medians (ranges) of 25(OH)D level were 15.4 (7.9-19.8) and 21.9 (20.2-48.6) ng/mL in VDD and non- VDD group, respectively. Sex, age, serum calcium, phosphate, ALP, PTH, liver iron concentration, cardiac T2*, and LVEF were not statistically different between the groups with or without VDD. Patients with VDD had significantly lower hemoglobin levels as compared to those without VDD (7.6 ± 0.9 vs 8.1 ± 0.9; p=0.043).

**Conclusion**: VDD is prevalent in patients with TDT and is correlated with hemoglobin level. The correlations of VDD to cardiac iron uptake and function are warranted in the large population with TDT.

## O9-1 FGFR1 mutations in four patients with congenital hypogonadotropic hypogonadism and split-hand/foot malformation: implications for the FGFR1 proximal promoter region

### Kohnosuke Ohtaka^1^, Yasuko Fujisawa^1^, Rie Yamaguchi^1^, Fumiko Kato^1^, Hideaki Yagasaki^1^, Tatsuya Miyoshi^2^, Yukihiro Hasegawa^2^, Tomonobu Hasegawa^3^, Hideaki Miyoshi^4^, Fumio Takada^5^, Momori Katsumi^6^, Maki Fukami^6^, Tsutomu Ogata^1^

#### ^1^Department of Pediatrics, Hamamatsu University School of Medicine; ^2^Division of Endocrinology and Metabolism, Tokyo Metropolitan Children's Medical Center; ^3^Department of Pediatrics, Keio University school of Medicine; ^4^Division of Rheumatology, Endocrinology and Nephrology, Hokkaido University Graduate School of Medicine; ^5^Department of Medical Genetics, Kitasato University Graduate School of Medical Sciences; ^6^Department of Molecular Endocrinology, National Research Institute for Child Health and Development

**Background**: Heterozygous loss-of-function mutations of FGFR1 are known to cause multiple disorders including congenital hypogonadotropic hypogonadism (CHH), CHH with anosmia/hyposmia (Kallmann syndrome, KS), and CHH with split-hand/foot malformation (CHH-SHFM), with variable expressivity and penetrance.

**Objective**: The objective of this study was to examine FGFR1 in four Japanese patients with CHH-SHFM.

**Patients**: This study consisted of four Japanese patients (cases 1–4) with CHH-SHFM. Case 1 was a 3-month-old boy with micropenis, undetectable serum LH and testosterone at mini-puberty, and right split hand. Case 2 was a 17-year-old boy with no pubertal development, undetectable serum LH and testosterone, and bilateral split hands and feet. Case 3 was a 34-year-old female with primary amenorrhea, low serum LH (0.4 mIU/mL) and undetectable serum E2, and left split hand. Case 4 was a 2-month-old boy with micropenis, undetectable serum LH and testosterone at mini-puberty, bilateral split hands and feet, and cleft lip and palate.

**Results**: Direct sequencing identified two heterozygous missense mutations (p.G97S in case 1 and p.R744T in case 2) and a heterozygous splice donor site mutation (IVS12+1G>T in case 3). The two missense mutations had drastically reduced luciferase activities, without a dominant negative effect. The splice donor site mutation yielded a small amount of mRNA skipping exon 12; in addition, it was predicted to produce two different types of aberrant mRNAs satifying the condition for nonsense-mediated mRNA decay by in silico analysis. In case 4, although no mutation wasfound on the coding exons 2-18, a microdeletion around non-coding exon 1 was revealed by MLPA analysis. This microdeletion was localized to a roughly 7.2-12.7 kb region by array CGH analysis, and was determined to be 8,312 bp long by direct sequencing for a PCR product obtained with primers flanking the microdeletion. Quantitative real time PCR analysis using primers for exons 8 and 9 indcated roughly halved FGFR1 expression in immortalized lymphoblastoid cells. In silico analysis indicated the presence of promoter-compatible H3K4Me3 marks and CpG sites around exon 1, and luciferase assays using various genomic fragments around exon 1 showed high transactivation function for a 470 bp segment encompassing the 5’ part and the just upstream region of exon 1.

**Conclusion**: The results are consistent with heterozygous loss-of-function mutations of FGFR1 being relevant to the development of CHH- SHFM, and indicates the presence of the FGFR1 promoter around non-coding exon 1.

## O9-2 Low Anti-Mullerian hormone level may indicate ovarian dysfunction in pubertal female childhood cancer survivors

### Kanako Tanase-Nakao, Yusuke Fujisawa, Kazuko Mizutani, Yumiko Terada, Yuta Chiba, Tomoko Yoshida, Yasuko Ogiwara, Keisuke Yoshii, Yasuhiro Naiki, Reiko Horikawa

#### Division of Endocrinology and Metabolism / Department of Pediatrics, National Center for Child Health and Development

**Background**: Along with the improvement of cancer treatments, the number of childhood cancer survivors (CCSs) is increasing. Together with this, long-term endocrine complications such as gonadal dysfunction are gathering more attention. Anti-Mullerian hormone (AMH) implies ovarian function in female. However, the clinical utility of AMH on evaluation of gonadal function in children and adolescent of CCSs is not well established.

**Objective**: To investigate the relationship between AMH concentration and gonadotropin (Gn) level in female CCSs and to investigate its usefulness in evaluation of gonadal function.

**Subjects & Method**: Thirty-nine samples of 30 female CCSs (aged 0-18) were involved in this study. We retrospectively analyzed the data of AMH and baseline Gns and/or Gn levels after LHRH stimulation test, obtained from June 2012 to June 2016 in our center. AMH was measured by ELISA and Gns were measured by CLIA.

**Result**: Mean age at diagnosis for primary cancer was 6.6 ± 5.2 years old and mean age at measurement of AMH was 8.9 ± 5.5 years old. All the samples were obtained after treatments for cancer were conducted, 16 samples were within 1 year and 23 samples were beyond 1 year from the treatment. Primary cancers were 3 brain tumors, 14 solid tumors other than brain and 13 hematologic cancers. Data measured under hormone replacement therapy was excluded. Mean AMH levels of 13 samples in 12 cases with age < 6 years old was 0.7 ± 2.2ng/ ml, 8 samples in 4 cases with age >6 years old and prepuberty was < 0.1ng/ml, and 18 samples in 14 cases with puberty was 1.8 ± 3.4ng/ ml. There was no statistically significant association between AMH concentration and gonadotropin levels throughout all age group. However, Gn levels in pubertal cases with undetectable AMH level (< 0.10 ng/ml) were remarkably high when compared with pubertal cases with detectable AMH ( ≥ 0.10 ng/ml). Mean baseline LH level (n=9) was 30.4 ± 34.7 mIU/ml, mean peak LH level (n=5) was 86.9 ± 61.4 mIU/ml, mean baseline'sH level (n=9) was 52.7 ± 42.5 mIU/ml and mean peak'sH level (n=5) was 401.1 ± 708.2 mIU/ml in cases with undetectable AMH. In contrast, mean baseline LH level (n=9) was 1.8 ± 1.7 mIU/ml, mean peak LH level (n=3) was 1.1 ± 1.3 mIU/ml, mean baseline'sH level (n=9) was 3.1 ± 1.7 mIU/ml and mean peak'sH level (n=3) was 6.6 ± 5.7 mIU/ml in cases with detectable AMH.

**Conclusion**: Undetectably low AMH in pubertal female CCSs is associated high Gn levels and may indicate primary hypogonadism.

## O9-3 Genetic diagnosis of 8 lipoatrophy patients in one single center via whole exome sequencing

### Jia-tong Hou, Shi-yao Wang, Lin-jie Wang, Hong-bo Yang, Hua-bing Zhang, Feng-ying Gong, Hui Pan, Hui-juan Zhu

#### Department of Endocrinology, Key Laboratory of Endocrinology of Ministry of Health CPeking Union Medical College Hospital, Chinese Academy of Medical Sciences & Peking Union Medical College

**Background**: Lipoatrophy is a group of rare diseases characterized by lacking subcutaneous adipose tissue. It is agreed that lipoatrophy is closely associated with various genetic variations. Despite lots of genes had been defined as causal genes in many types of genetic lipodystrophy, the molecular mechanisms of some rare lipoatrophy patients with distinctive phenotypes remain unclear.

**Methods**: Eight patients with adolescent-onset lipoatrophy features were recruited from Department of Endocrinology, Peking Union Medical College Hospital. Clinical and biochemical parameters of the patients are collected in details. Whole exome sequencing was performed in all 8 patients, aiming to identify the potential gene mutations linked with their lipoatrophy manifestations.

**Results**: All the patients had generalized fat loss and thin mottled skin from the adolescent period. Two of them had progeroid features. Clinical features of the patients were as follows: fasting insulin (43.93 ± 7.66) μIU/ml, fasting glucose (5.7 ± 2.9) mmol/l, glycosylated hemoglobin (6.1 ± 0.8)%; triglycerides (3.57 ± 1.40) mmol/l, total cholesterol (4.31 ± 0.99) mmol/l, low-density lipoprotein cholesterol (2.52 ± 0.60) mmol/l, high density lipoprotein cholesterol (0.87 ± 0.25) mmol/l, body fat rate (28.6 ± 9.0)%. With whole-exome sequencing, we identified one POLD1 mutation (c.1812_1814delCTC, p.Ser605del) in two patients, one WRN mutation (c.3020delG, p.Gly1007Alafs*16) in one patient, one CIDEC mutation(c.686C>T, p.Thr229Met) in one patient, two INSR mutations(c.4028G>A, p.Arg1343Gln and c.1534A>G, p.Ile512Val) in two patients, and two LMNA mutations(c.898G>C, p.Asp300His and c.29C>T, p.Thr10Ile) in two patients. While one patient showed no genetic variations.

**Conclusions**: Through whole-exome sequencing, eight gene mutations probably linked with lipoatrophy were detected. All gene mutations detected could cause different subtypes of lipodystrophy. When patients reveal lipoatrophy and progeroid features from the adolescent period, the diagnosis of lipoatrophy should be taken into consideration. Our study expanded the spectrum of clinical manifestations and genetic variations of lipodystrophy. Like all previously reported patients, these cases were diagnosed with whole exome sequencing, highlighting the clinical utility of next generation sequencing in the diagnosis of rare, complex disorders.

**Key words**: Lipodystrophy, whole exome sequencing, genetics, diagnosis

## O9-4 Adult height and growth pattern in patients with classic congenital adrenal hyperplasia

### SeokJin Kang, Mo Kyung Jung

#### Yonsei University College of Medicine, Severance Children's Hospital, Endocrinologic Pediatrics

**Background**: Congenital adrenal hyperplasia (CAH), mostly caused by 21-hydroxylase deficiency, is autosomal recessive disorder characterized by impaired cortisol synthesis. It can be presented with a combination of aldosterone and cortisol deficiency and androgen excess. Therefore, excess production of androgen and glucocorticoid replacement can result to early bone maturation and ultimately diminished adult height (AH).

**Objectives**: The purpose of this study was to obtain objective data on AH with classic CAH patients and analyze the affecting factors on AH. Also we evaluated growth pattern during age increase.

**Study design**: We retrospectively reviewed the longitudinal medical records of 40 children with classic CAH (male [n=19]: 9 salt-wasting (SW), 10 simple-virilizing (SV); female [n=21]: 8 SW, 13 SV) who reached AH at Pediatric endocrinology clinic of Severance hospital from 1977 to 2015. We also analyzed the affecting factors on AH, and assessed growth patterns with serial height standard deviation score (SDS) dividing into following stages of growth: early childhood (0-4.99 years), mid-childhood (5-9.99 years), and adolescence (10-15 years).

**Results**: AH (162.7 ± 9.72 cm) was significantly shorter than the midparental height (MPH) (172.5 ± 3.40 cm) in male patients (P <0.001), and similarly, AH (154.5 ± 6.45 cm) was significantly shorter than the MPH (158.7 ± 2.96) in female patients (P =0.002). Accordingly, the AH SDS was meaningfully lower than the MPH SDS in both sex (males: P <0.001, females: P =0.002). Considering subtypes, SV had tendency to attain shorter AH than SW. In addition, the affecting factors on AH were analyzed that they were not significantly associated with subtype, age at diagnosis, dose of steroid, except MPH. Height SDS for chronologic age showed gradual decrement during childhood to adolescence (males: 0.5 ± 2.51 at early childhood, 0.8 ± 2.26 at mid-childhood, 0.2 ± 1.62 at adolescence; females: -0.4 ± 1.40, -0.2 ± 2.01, -0.3 ± 1.42 at those same periods). The final AH SDS was -1.6 ± 1.98 in males and -0.81 ± 1.45 in females.

**Conclusion**: Reduced AH was observed in children with classic CAH compared with their given parental height, regardless of sex and subtype. Furthermore, the height SDS tended to decrease in accordance with age increase, so this finding suggests that proper intervention about growth assessment is needed in children with CAH.

## O9-5 Gene Expression Profiles of Children with GH Deficiency (GHD) prior to Treatment with Recombinant Human Growth Hormone (r-hGH) are Associated with Growth Response Over Five Years of Therapy

### Adam Stevens^1^, Philip Murray^1^, Ekaterina B Koledova^2^, Pierre Chatelain^3^, Peter Clayton^1^

#### ^1^University of Manchester and Royal Manchester Children's Hospital; ^2^Merck KGaA; ^3^University Claude Bernard

**Background**: The relationship between long-term growth response in GHD and pre-treatment whole genome gene expression (GE) as an integrated biomarker of the consequences of being GHD has never been explored. Prediction of long-term response to r-hGH therapy would allow better decision making about start and maintenance doses and hence cost:benefit.

Objective and hypotheses: To assess the relationship of pre-treatment GE to growth response on r-hGH in GHD children over five years of therapy.

**Method**: Pre-pubertal children with GHD (n=50) were enrolled from the PREDICT studies (NCT00256126 and NCT00699855). Affymetrix U133 plus 2.0 microarrays, analysed using Gene Expression Barcode 3.01, were used to determine whole blood GE prior to treatment. Growth response to r-hGH treatment was determined using Height velocity (HV) over the five years of therapy. Two groups of patients were defined by assessing growth response over the five years of treatment, (G1) always above and (G2) always below the median. The effect of gender, age and distance to target height (DTH) on GE were accounted for as covariates. Network models of gene expression and random forest analysis, a machine learning analytical technique, were used to relate GE to growth response using area under the curve (AUC) of the receiver operating characteristic. The robustness of GE markers was assessed using permutation testing (n=1000).

**Results**: There was no difference in gender, age and DTH (p>0.05) between the HV groups (G1 and G2) at the start of treatment. Baseline GE could predict growth response consistently above and below the median over five years with an AUC of 0.86 and 0.89 respectively. These observations were confirmed by permutation analysis. Genes were identified (p<1x10-5) unique to G1 patients (n=69) or to G2 patients (n=72). Network models of GE prioritised 94 of these 141 genes. PIK3R1 gene expression (p=1.2x10-9), associated with cell proliferation and known to be activated by GH, was related to G1. DDX58 gene expression (p=2.2x10-10), associated with RNA secondary structure, was related to G2.

**Conclusion**: In pre-treatment GHD we have identified gene expression associated with growth response over five years of therapy. Further assessment to determine predictive value and functional significance of gene subsets is required.

**Reference**

1. McCall et al Nucleic Acids Research. 2014 Jan; 42:D938–D943.

## O9-6 Further evidence for the involvement of the specific ESR1 haplotype in the susceptibility to estrogenic endocrine disruptors

### Yasuko Fujisawa^1^, Rie Yoshida^2^, Francesco Massart^3^, Naoyuki Kamatani^4^, Maki Fukami^2^, Tsutomu Ogata^1^

#### ^1^Department of Pediatrics, Hamamatsu University School of Medicine; ^2^Department of Endocrinology and Metabolism, National Research Institute for Child Health and Development; ^3^Department of Pediatrics, University of Pisa; ^4^Institute of Data Analysis, StaGen Co. Ltd

We have revealed that ESR1 encoding estrogen receptor a carries a ~ 50 kb linkage disequilibrium (LD) block in its 5’ region, and that a specific “AGATA” haplotype markedly raises the susceptibility to the development of male genital abnormalities by estrogenic endocrine disruptors (EEDs) in Japanese population. Here, we report further evidence for the involvement of the specific “AGATA” haplotype in the susceptibility to EEDs.

Identification of a 2,244 bp microdeletion in an absolute linkage disequilibrium with the specific “AGATA” haplotype We sequenced the region encompassing the LD block in two homozygotes for the specific haplotype and two subjects without this haplotype, and found a 2,244 bp microdeletion within intron 6 of the ESR1 gene in the homozygotes only. Subsequent analysis in Japanese subjects including 35 homozygotes and 151 heterozygotes for the “AGATA” haplotype and 188 subjects lacking this haplotype demonstrated an “absolute LD” between this microdeletion and the “AGATA” haplotype.

Examination of the ESR1 haplotype and the microdeletion in Italian population Next, we studied Italian boys with hypospadias (n=13) and cryptorchidism (n=80), and Italian control boys (n=150). The same LD block and the same four major haplotypes as in Japanese population were identified, and the “AGATA” haplotype was significantly associated with the external genital abnormalities as well. Furthermore, a nearly complete absolute LD was identified between the microdeletion and the specific haplotype, except for two subjects. This argues that in Italian population, the same genetic susceptibility factor is also involved in the development of male external genital abnormalities.

Comparison of the frequency of the mictodeletion between affected subjects and normal controls The frequency of the microdeletion allele was significantly higher in all groups of affected subjects. Consequent genotype analysis indicated that the frequency of the miclodeletion homozygotes was significantly higher in the Japanese HS (17.94%) than in the control boys (3.26%) as well as in Italian population (HS+CO; 3.23%, control boys; 0.67%). Comparison of the frequency of the susceptibility factor between different generations We compared the frequency of the normal homozygotes or heterozygotes for the susceptibility factors between the two generations. The frequency of the homozygotes/heterozygotes of the microdeletion was significantly lower in the normal child (39/98) than in the normal adult (167/311). This implies of that male subjects carrying the microdeletion are more susceptible to have genital abnormalities under the elevated levels of the EEDs.

## P1-1-1 Status and Trends in use of insulin analog for Japanese children and adolescents with type 1 diabetes mellitus and its association with glycemic control

### Yukiyo Yamamoto^1,2^, Toru Kikuchi^1,3^, Tatsuhiko Urakami^1,4^, Kohji Tsubouchi^1,5^, Goro Sasaki^1,6^, Haruo Mizuno^1,7^, Yuki Abe^1,8^, Kazuteru Kitsuda^1,9^, Shigetaka Sugihara^1,10^

#### ^1^The Japanese Study Group of Insulin Therapy for Childhood and Adolescent Diabetes (JSGIT); ^2^Department of Pediatrics, University of Occupational and Environmental Health Japan; ^3^Department of Pediatrics, Faculty of Medicine, Saitama Medical University; ^4^Department of Pediatrics, Nihon University School of Medicine, Department of Pediatrics; ^5^Chuno Kosei Hospital; ^6^Department of Pediatrics, Tokyo Dental College Ichikawa General Hospital; ^7^Departments of Pediatrics and Neonatology, Nagoya City University Graduate School of Medical Sciences; ^8^Department of Pediatrics, Niigata City General Hospital; ^9^Department of Pediatrics, Kitasato University; ^10^Department of Pediatrics, Tokyo Women's Medical University Medical Center East

**Background and Purpose**: Treatment for type I diabetes mellitus (T1DM) has been greatly changing by common use of insulin analog and continuous subcutaneous insulin infusion (CSII). We investigated current status and trends in use of insulin analog for Japanese children and adolescents with T1DM and its association with glycemic control.

**Subjects**: The study subjects consisted of 1090 patients, 449 males and 641 females (79 patients; 0-5 years of age, 280 ; 6-10 years of age, 731 : 11-19 years of age) enrolled in 4th cohort of JSGIT (from 1st to 3rd period: March 2013 to February 2014).

**Results and Discussion**: Almost all patients were being treated with multiple daily injections (MDI) or CSII. The proportion of CSII increased (MDI vs CSII; 71.8% vs 23.4%, respectively at 1st period to 69.8% vs 25.8%, respectively at 3rd period), especially in 0-5 years of age. Within patients with MDI, use of frequent injections with more than 5 times a day increased. On the other hand, use of twice basal injections decreased, suggesting that use of frequent bolus injections adapted to lifestyle were increasing. The most frequently used basal insulin was Glargine (G), followed by Detemir (D) and Degludec (T). However, G and D decreased (G: 529 patients at 1st period to 424 at 3rd period, D: 217 to 163), whereas T increased (34 to 123). The most frequently used bolus insulin was Aspart (A), followed by Lispro (L) and Glulisine (AP). The most frequently used insulin in CSII was A, followed by L and AP. In terms of bolus and CSII, trends are similar during this period. The most frequently used basal-bolus combination was G-A, followed by D-A. However, both combinations decreased (G-A: 397 to 312, D-A: 201 to 150), whereas T-A increased (25 to 96). The median hemoglobin A1c (HbA1c) in patients using each insulin analog and combination were 8.0%(G), 8.0%(D), 8.2%(T), 8.0%(A), 8.0%(L), 8.2%(AP), 7.9%(CSII-A), 8.2%(CSII-L), 7.6%(CSII-AP), 8.0 %( G-A), 7.8 %( G-L), 8.1 %( G-AP), 8.0%( D-A), 7.7 %( D-L), 7.6 %( D-AP), 8.2 %( T-A), 8.6 %( T-L), 8.4 %( T-AP). Among patients using any insulin analog for basal and CSII, patients using T and CSII-L had significantly higher levels of HbA1c. On the other hand, there was no difference among any bolus, CSII, and basal- bolus combinations. These findings are the result from database of 1st year in this cohort. Continuous investigation is needed under longer- term use.

## P1-1-2 Next-generation sequencing-based screening of monogenic mutations in 43 Japanese children clinically diagnosed with type 1B diabetes

### Kikumi Ushijima^1^, Maki Fukami^1^, Tadayuki Ayabe^1^, Misako Okuno^1,2^, Satoshi Narumi^1^, Tsutomu Ogata^3^, Nobuyuki Kikuchi^4^, Tomoyuki Kawamura^5^, Tatsuhiko Urakami^2^, Ichiro Yokota^6^, Shin Amemiya^7^, Shigetaka Sugihara^8^

#### ^1^Department of Molecular Endocrinology, National Research Institute for Child Health and Development; ^2^Department of Pediatrics and Child Health, Nihon University School of Medicine; ^3^Department of Pediatric, Hamamatsu University School of Medicine; ^4^Department of Pediatric, Yokohama City Minato Red Cross Hospital; ^5^Department of Pediatric, Osaka City University School of Medicine; ^6^Division of Pediatric Endocrinology and Metabolism, Shikoku Medical Center for Children and Adults, Department of Pediatric; ^7^Department of Pediatric, Saitama Medical University Faculty of Medicine; ^8^Department of Pediatric, Tokyo Women's Medical University Medical Center East

**Background**: Diabetes mellitus is classified into type 1 (T1D), type 2, other specific types, and gestational diabetes. T1D lacking diabetes- associated autoantibodies are termed as type 1B (T1BD). The group of “other specific types of diabetes mellitus” includes monogenic diabetes such as neonatal diabetes and maturity-onset diabetes of the young caused by genetic defects in the insulin secretion pathway. Because clinical characteristics of those monogenic forms and T1BD are partially overlapping, children with monogenic diabetes could be clinically diagnosed with T1BD, especially during childhood.

**Objectives**: The objectives of this study was to clarify the prevalence and clinical consequences of monogenic mutations in Japanese children clinically diagnosed with T1BD.

**Methods**: We studied 43 Japanese children from 42 families diagnosed with T1D at age between 0.5 and 16.0 years and had no diabetes- associated autoantibodies. The participants were recruited from 30 hospitals during the fourth cohort study of the Japanese Study Group of Insulin Therapy for Children and Adolescent Diabetes. We performed genetic analysis using the HaloPlex target enrichment system (Agilent) and a next-generation sequencer HiSeq (Illumina) to screen mutations in 30 genes known to cause monogenic diabetes.

**Results**: Four of the 43 participants had heterozygous missense mutations in the insulin gene (INS ). No mutations were observed in the remaining 29 genes. The INS mutations (p.G75C, p.C96F and p.V42A) were hitherto unreported. The p.C96F mutation-carrying children were siblings, whose mother was also affected by T1D. No significant differences were observed in body mass index-Z score between the INS mutation carriers and non-carriers (-0.4 vs -0.9, p = 0.26). Age at diagnosis was significantly younger in the INS mutations carriers than that of non-carriers (2.7 vs 9.4 years, p = 0.025).

**Conclusions**: The results indicate that small proportion of children clinically diagnosed with T1BD children with onset age >0.5 years had monogenic mutations. Mutation screening of those children is helpful not only to understand the molecular pathogenesis but also to provide individualize management, including genetic counseling.

## P1-1-3 Association Between Leptin, Soluble Leptin Receptor, and Free Leptin Index with Body Mass Index in Elementary School Children in Medan, Indonesia

### Siska Mayasari Lubis^1^, Jose RL Batubara^2^, Harun Alrasyid Damanik^3^, Miswar Fattah^4^

#### ^1^Pediatric Endocrinology Division, Child Health Department, Medical School, University of Sumatera; ^2^Pediatric Endocrinology Division, Medical School, University of Indonesia; ^3^Department of Nutritional Sciences, Medical School, University of Sumatera; ^4^Department of Molecular Biology, Prodia Widyahusada Laboratory

**Introduction**: In recent years, obesity is becoming an epidemic health problem. Leptin is known to play an important role in the pathogenesis of obesity. Levels of soluble leptin receptor (sOB-R) can provide an indication of free leptin, are postulated to play an important role in the pathophysiology of energy homeostasis. The free leptin index (FLI), which may be a more accurate determinant of leptin function.

**Objective**: To evaluate the association between leptin, sOB-R, and FLI with body mass index (BMI) in elementary school children in Medan, Indonesia.

**Methods**: We conducted a case control study in ten elementary schools in Medan, Leptin and sOB-R were measured by enzyme link immunosorbent assay (ELISA). The ratio of serum leptin to sOB-R provides a measure of the free leptin index (FLI).

**Results**: A total of 202 children between 6 and 12 years old were recruited in this study. We found significantly association between body mass index with leptin, sOB-R, and FLI (p <0.05). Soluble OB-R levels were lower in children with obesity than normal weight children (28.1 ± 8.1, 37.7 ± 9.3 ng/mL, respectively), but, leptin and FLI levels were higher than control (756.9 ± 493.4, 160,7 ± 284.7 ng/mL, respectively).

**Conclusions**: This study showed a decrease in levels of sOB-R and increase levels of leptin and FLI in children with obesity, and there were significantly associations between Leptin, sOB-R, and FLI with BMI.

**Keywords**: Leptin, free leptin index, soluble leptin receptor, body mass index, obesity

## P1-1-4 Erythropoietin ameliorates obesity and glucose intolerance on high fat dietary obese mice through the activation of erythropoietin receptor/STAT3 pathway and the up-regulation of FGF21 secretion on classical brown adipose tissue

### Hisakazu Nakajima^1^, Kazuki Kodo^1^, Satoru Sugimoto^1,2^, Ikuyo Itoh^1,3^, Keiichi Shigehara^1^, Shota Fukuhara^1^, Hidechika Morimoto^1^, Jun Mori^1^, Taichiro Nishikawa^1,4^, Kitaro Kosaka^1,5^, Hajime Hosoi^1^

#### ^1^Department of Pediatrics, Kyoto Prefectural University of Medicine; ^2^Department of Pediatrics, Ayabe Municipal Hospital; ^3^Department of Pediatrics, Tanabe Central Hospital; ^4^Department of Gastroenterology and Hepatology, Kyoto Prefectural University of Medicine; ^5^Department of Pediarics, Saiseikai Kyoto Hospital

**Background, Aims and Objectives**: We hypothesized that classical brown adipose tissue (cBAT) could play a crucial role in the anti-obesity effects of erythropoietin (Epo). Our study highlights the mechanism in which Epo treatment could upregulate energy expenditure and improve glucose homeostasis through cBAT in obese mice fed with a high-fat diet (HFD).

**Methods**: C57BL/6J mice had been fed with HFD since the age of 4 weeks (HFD mice). We administered recombinant human Epo (200IU/kg) to some HFD mice intraperitoneally, 3 times per week for 4 weeks (HFD+Epo mice). Blood glucose, serum insulin and FGF21 were monitored and an intraperitoneal glucose tolerance test (IPGTT) was performed on the two groups. We analyzed the interscapular BAT (iBAT) and the liver harvested from both groups using molecular biology and physiological methods.

**Results**: Body weight, blood glucose and serum insulin were decreased in HFD+Epo mice compared with HFD mice. Serum level of FGF21, interscapular surface temperature and oxygen consumption were higher in HFD+Epo mice in comparison with HFD mice. The IPGTT showed that the levels of blood glucose were lower in HFD+Epo mice than in HFD mice. The weight of iBAT was larger in HFD+Epo mice than in HFD mice, whereas that of white adipose tissue was decreased in HFD+Epo mice than in HFD mice. The mRNA and protein levels of UCP1, beta3ADR, PRDM16, PPAR α and FGF21 in the iBAT were higher in HFD+Epo than in HFD mice. However, the mRNA and protein level of FGF21 in the liver was similar between the two groups. In addition, the mRNA levels of PEPCK and G6Pase in the liver were lower in HFD+Epo mice compared with HFD mice. Epo treatment significantly stimulated phosphorylation of Epo receptor (EpoR) /STAT3 in the iBAT in a HFD condition.

**Conclusions**: The activation of EpoR/STAT3 pathway in the cBAT by extrinsic Epo could contribute to the upregulation of energy expenditure and the improvement of glucose homeostasis by increasing the secretion of FGF21 on the cBAT in HFD mice.

## P1-1-5 Basal and bolus insulin requirement in children with type 1 diabetes on sensor augmented pump in our hospital

### Yusuke Mine, Satomi Tanabe, Masako Aoki, Misako Okuno, Junichi Suzuki, Tatsuhiko Urakami

#### Department of Pediatrics and Child Health, Nihon University School of Medicine

**Introduction**: Previous s tudies have demonstrated total basal insulin dose (TBD)/ total daily insulin dose (TDD) as about 40-50% and TDD as about 0.8-1.2U/kg/day in children with type 1 diabetes on CSII. Since Feb, 2015 sensor augmented pump (SAP) is available in Japan, and we expected the improvement of HbA1c level and the adjustment of the bolus basal ratio. Therefore, we investigated the changes in HbA1c level, TDD (U/kg/day) and TBD/ TDD (%) among children under 18 years old with type 1 diabetes after starting SAP therapy in our hospital.

**Subjects and methods**: Subjects included 11 children with type1 diabetes (M/F= T/ U, 9.0 ± 8.7 years old). They were classified into good glycemic control group (HbA1c<7.5 n=4, 9.9 ± 8.8 years old) and the poor glycemic control group (HBA1c>8.5 n=4, 10.3 ± 5.0 years old) based on HbA1c level after starting SAP therapy. TBD/TDD and TDD also investigated between the two groups.

We compared HbA1c level, TBD/TDD and TDD after and before SAP treatment in all subjects, and also compared TBD/TDD and TDD between the good and poor glycemic control groups.

**Results**: HbA1c level were significantly lowered by using 0.8% after using SAP (from 8.6% to 7.8%, p=0.036). In 7 out of 11 patients, HbA1c levels were lowered by 0.5%, however, TBD/TDD and TDD didn’t change significantly after using SAP (p=0.846 vs p=0.76). BD/TDD showed significant difference between the good and poor glycemic control groups (median 28% vs 43%, P=0.03). Whereas, TDD between the two groups didn’t change significantly (median 0.855U/kg/day vs 0.875U/kg/day, p=1.0).

**Discussion**: SAP therapy effectively lowered HbA1c level in children with type 1 diabetes. Overall, TBD/TDD and TDD didn’t change, but TBD/TDD in the good glycemic control group was significantly lower than in the poor glycemic control group. These results suggested that good glycemic control was achieved by increasing the bolus based on the sensor glucose levels.

## P1-1-6 The cutoff values of indirect indices for measuring insulin resistance in Korean children and adolescents

### Heon-Seok Han^1^, JunWoo Kim^1^, Kyung Hee Yi^2^

#### ^1^Division of Endocrinology / Department of Pediatrics, Chungbuk National University College of Medicine; ^2^Department of Pediatrics, Wonkwang University Sanbon Medical Center

**Purpose**: We investigated the prevalence rate of metabolic syndrome (MetS) among Korean children and aolescents, and the distribution of indirect index of insulin resistance (IR) using homeostasis model of insulin resistance (HOMA-IR) and triglyceride and glucose (TyG) index. Also we suggested the cutoff values of HOMA-IR and TyG index to screen high risk group of MetS.

**Methods**: Data from 3,3313 Korean subjects (1,756 male and 1,556 female, aged 10-18 years) were included from the Korean National Health and Nutrition Examination Survey (K-NHANES) conducted during 2007-2010. We used three different criteria of MetS by de Ferranti et al., Cook et al., and International Diabets Federation (IDF). The cutoff values of HOMA-IR and TyG indx were obtained from the receiver- operation characterisitics curves.

**Results**: According to the criteria of MetS in the order of de Ferranti et al., and Cook et al., and IDF, the prevalence rates of MetS in male and female were 13.9% and 12.3%, 4.6% and 3.6%, and 1.4% and 1.8%, respectively. The cutoff values of HOMA-IR and TyG index were 2.94 and 8.41, 3.29 and 8.48, and 3.54 and 8.66, respectively. Each cutoff values from the three criteria approximately corresponds to 50th, 75th, and 75th to 90th percentiles of normal distribution of HOMA-IR and TyG index.

**Conclusion**: We presented the prevalence rate of MetS, the distribution of indirect index of IR, and the cutoff values to screen high risk group of MetS in Korean children and adolescents. The usefulness of these criteria needs to be verified by further evaluatin.

## P1-1-7 Early Development of Decreased β-cell Insulin Secretion in Children and Adolescents with HbH Disease and Its Relationship with the Hematological Severity

### Pairunyar Nakavachara^1^, Worarat Kajchamaporn^1^, Vip Viprakasit^2^

#### ^1^Department of Pediatrics, Faculty of Medicine Siriraj Hospital, Division of Pediatric Endocrinology, Mahidol University; ^2^Department of Pediatrics, Faculty of Medicine Siriraj Hospital, Division of Pediatric Hematology and Oncology, Mahidol University

**Background**: Endocrinopathies related to iron overload are common among transfusion-dependent thalassemia (TDT) patients. Recently, studies have shown that endocrinopathies such as adrenal insufficiency, vitamin D deficiency, low bone mass and glucose intolerance are also quite common among non-transfusion dependent thalassemia (NTDT) patients.

Although HbH is the most common NTDT in Southeast Asia, little is known regarding the glucose metabolism among these patients. The purpose of our study was to evaluate the glucose tolerance among young HbH patients.

**Methods**: We investigated β -cell function (HOMA- β ) and insulin sensitivity (HOMA–IR) by performing OGTT among 32 children and adolescents with HbH disease, mean aged 15.5 (range; 10.7-24.9) years. Patients were divided into two groups based on the types of HbH which were deletional (--/ α -, n = 16) and non-deletional HbH (--/ αα T, n = 16). We compared demographic, hematological data, HOMA-β and HOMA–IR between deletional vs. non-deletional groups. We also compared demographic and hematological data between normal vs. abnormal HOMA- β and normal vs. abnormal HOMA–IR groups. Statistical analyses were performed using the unpaired t-test, the Mann-Whitney U-test and the chi-squared test as appropriate. Statistical significance was considered at P value of < 0.05.

**Results**: All patients had normal glucose tolerance. However, 40.6% and 21.9% of patients had abnormal HOMA- β (decreased β -cell insulin secretion) and abnormal HOMA–IR (insulin resistance), respectively. Non-deletional patients had significantly lower height Z-score and functional hemoglobin levels [7.4 ± 1.2 vs. 8.5 ± 1.3 g/dL, P=0.018 ] but higher serum ferritin levels [237.9 (range 60.5 to 1881.8) vs. 87.9 (range 38.2 to 414.0) ng/mL, P=0.012 ] than deletional HbH patients. Interestingly, patients with abnormal HOMA- β had significant lower weight Z-score and functional hemoglobin levels [7.6 ± 1.5 vs.8.3 ± 1.1 g/dL, P=0.039 ] than those with normal HOMA- β . Meanwhile, patients with abnormal HOMA–IR had significant higher weight Z-score and higher percentage of overweight and obesity than those with normal HOMA-IR. The types of HbH disease were not significantly different between normal vs. abnormal HOMA- β groups and normal vs. abnormal HOMA–IR groups.

**Conclusion**: A high prevalence of decreased β -cell insulin secretion was shown for the first time among young HbH patients in this study. The severity of anemia as determined by the levels of functional hemoglobin seemed to be related with decreased β -cell insulin secretion independent of the types of HbH disease. Prospective studies to evaluate glucose metabolism among children and adolescents with HbH disease who have impaired β -cell insulin secretion are needed.

## P1-1-8 Increased prevalence of autoimmune thyroid disease in children and young adults with type 1 diabetes in Japan

### Shino Odagiri, Satsuki Nishigaki, Takashi Hamazaki, Yuko Hotta, Kayako Hashimura, Masakazu Hirose, Tomoyuki Kawamura, Haruo Shintaku

#### Department of Pediatrics, Osaka City University Graduate School of Medicine

**Background**: Type 1 diabetes (T1D) is frequently associated with other autoimmune disease, including autoimmune thyroid disease (AITD). There is a paucity of understandings of AITD in children and young adults with T1D. Here we investigated the clinical features of T1D patients with AITD.

**Subjects and methods**: Data were analyzed from 446 patients with T1D (2.2-51.7 years old; the mean age, 20.3 ± 9.1 years; 57.7% female), who visited our department during April 2014-March 2016, by using the medical records.

**Results**: Among 446 patients with T1D, total 30 patients (76.7% female) were diagnosed with any of autoimmune diseases; 29 AITD, 1 SLE, 1 celiac disease, 1 ITP. There were 2 patients with three autoimmune diseases, one was T1D with Hashimoto’s thyroiditis (HT) and ITP, and the other was T1D with HT and celiac disease. Out of the 29 T1D patients with AITD, 18 patients were diagnosed with Graves’ disease (GD) (84.2% female) and the rest of 11 patients (60% female) were diagnosed with clinical HT requiring treatments. Antithyroid antibodies (thyroglobulin and thyroid peroxidase) were examined in 271 T1D patients. 77 (28.4%) out of the 271 patients were positive for any of the antibodies. Mean ages of T1D onset were not significantly different among T1D patients with or without AITD. The mean age at diagnosis of GD and HT was 18.0 and 8.2 years old, respectively. Out of 19 patients who developed GD, 15 patients developed GD after T1D was diagnosed. Mean interval was 12.8 years. While out of 10 patients who developed HT, 6 patients developed HT after T1D was diagnosed. Mean interval was 7.1 years. All of T1D patients with GD were controlled by antithyroid drug therapy, and none of them needed surgery or radiotherapy. Relapse of GD after remission occurred in 2 patients.

**Conclusions**: Prevalence of AITD in T1D patients was higher compared with general population. Especially female patients with T1D have higher risk of AITD than male patients with T1D. GD tends to develop several years after the onset of T1D and careful monitoring should be considered. Antithyroid antibodies’ positivity was high in T1D patients but most of the cases did not develop clinical HT. In conclusion, this study supports the recommendation for regular examination of thyroid antibodies and thyroid function in children and young adults with T1D.

## P1-1-9 Factors Associated with the Presence and Severity of Diabetic Ketoacidosis at Diagnosis of Type 1 Diabetes in Korean Children and Adolescents

### Hye Jin Lee^1^, HyeohWon Yu^1^, Hae Woon Jung^2^, Young Ah Lee^1^, Jae Hyun Kim^3^, Hye Rim Chung^4^, Jae Ho Yoo^5^, Eun Young Kim^6^, Jeesuk Yu^7^, Choong Ho Shin^1^, Seong Yong Lee^8^, Sei Won Yang^1^

#### ^1^Division of Endocrinology and Metabolism / Department of Pediatrics, Seoul National University Children's Hospital; ^2^Division of Endocrinology and Metabolism / Department of Pediatrics, Kyung Hee University Medical Center; ^3^Division of Endocrinology and Metabolism / Department of Pediatrics, Inje University College of Medicine, Ilsan Paik Hospital; ^4^Division of Endocrinology and Metabolism / Department of Pediatrics, Seoul National University Bundang Hospital; ^5^Division of Endocrinology and Metabolism / Department of Pediatrics, Dong A Univ Coll of Med; ^6^Division of Endocrinology and Metabolism / Department of Pediatrics, Chosun University, College of Medicine; ^7^Division of Endocrinology and Metabolism / Department of Pediatrics, Dankook University Hospital; ^8^Division of Endocrinology and Metabolism / Department of Pediatrics, Seoul National University College of Medicine, SNU-SMG Boramae Medical Center

Diabetic ketoacidosis (DKA) is a major life-threatening complication of type 1 diabetes (T1DM). The aim of this study was to identify the risk factors for the presence and severity of DKA at onset of T1DM in Korean children and adolescents.

A retrospective chart review of children and adolescents newly diagnosed as T1DM from January 2000 to May 2015 was done in 7 secondary and tertiary centers in South Korea. Eligible subjects were < under age of 20 years and had records on the presence or absence of DKA at T1DM diagnosis. The severity of DKA was categorized as mild (venous blood pH<7.3), moderate (venous blood pH<7.2) and severe (venous blood pH<7.1). Data was collected on age, height, body weight at diagnosis, body weight after stabilization, pubertal status, family history of diabetes, delayed diagnosis of T1DM, preceding infection, health insurance status, and parental education.

A total of 361 children and adolescents diagnosed with T1DM were included. The mean age was 8.8 ± 4.0 years and 37 patients were younger than 3 years of age and 91 patients were older than 12 years of age. 53.7% (n=194) of the patients were female. Overall 48.9% (n=177) of the patients presented with DKA at T1DM diagnosis. Thirty six percent of all patients (n=137) was not diagnosed as T1DM at the first medical consultation and in 13% (n=47), a preceding infection was present. The risk for DKA at diagnosis was 2.8 times (P=0.022) higher in patients younger than 3 years of age, and was 1.8 times (P=0.007) higher in patients older than 12 compared to patients between 3 and 12 years old. Lower serum c-peptide levels (P<0001), presence of preceding infection (P=0.001), and delayed diagnosis (P=0.02) significantly increased the risk of DKA at T1DM diagnosis. Multivariate analysis revealed that age greater than 12 (OR=2.8, PDelayed diagnosis, being too young to express symptoms or being old enough to be relatively free from parental care, and preceding infection increased the risk of DKA at T1DM diagnosis. Insurance status, parental educational level and preceding infection affected the severity of DKA. These results imply that alertness of the physician and public awareness of the symptoms of diabetes are needed to decrease the incidence and the severity of DKA at diagnosis of T1DM.

## P1-1-10 Decreased serum growth differentiation factor 15 level in pediatric patients with type 1 diabetes mellitus

### Takako Sasaki, Shuichi Yatsuga, Miyuki Kitamura, Junko Nishioka, Yasutoshi Koga

#### Department of Pediatrics and Child Health, Kurume University School of Medicine

**Introduction**: Growth differentiation factor 15 (GDF15) is a member of the BMP family of TGF-beta superfamily proteins, has been reported to increase in a various disorders including diabetes mellitus, cardiac/renal insufficiency or cancers. Several reports described that the elevation of GDF15 is the useful biomarker for diabetic nephropathy, or diabetic cardiomyopathy in adult. However, there are no reports about serum GDF15 in relation with the diagnostic purpose of type 1 diabetes mellitus (T1DM) in children. In this study, we investigated whether GDF15 is the good biomarker for diagnostic purpose of T1DM in children.

**Methods**: We collected serum samples in pediatric patients with T1DM and type 2 diabetes mellitus (T2DM), who were enrolled at Kurume University Hospital from April 2012 to June 2016. We also collected serum samples of pediatric voluntary healthy controls from July 2013 to August 2013. We measured serum GDF15 level. We also checked age, body weight, and height. Anti-GAD antibody, anti-insulin antibody, and anti-IA2 antibody were measured in all T1DM and T2DM patients at diagnosis. The study protocol was approved by the institutional review boards of all centers (coordinator: Kurume University Hospital, #13099). Written informed consent was obtained from all subjects before enrollment.

**Results**: Thirty-two (female; 16) pediatric T1DM, 8 (female; 4) pediatric T2DM and 60 (female; 23) controls were collected. The mean (SD) of age in T1DM, T2DM and controls was 9.80 (4.38), 9.54 (4.32), and 13.4 (2.05) years of age, respectively. The median of GDF15 in T1DM, T2DM and controls was 291.0, 414.0, and 453.5 ng/mL, respectively. No patients had diabetic retinopathy nor diabetic nephropathy. GDF15 was lower in T1DM compared to controls (p<0.0001), and T2DM (p=0.0243). GDF15 in T2DM was not significantly different compared to controls (p=0.42). GDF15 in T1DM was no significant difference from autoantibodies, respectively. (Anti-GAD antibody; p=0.66, anti-insulin antibody; p=0.57, anti-IA2 antibody; p=0.51).

**Discussion**: GDF15 in pediatric T1DM was significantly decreased than those seen in the controls and T2DM. Our results implied that the GDF15 may be a useful biomarker to distinguish T2DM in the patient autoantibodies were negative. GDF15 has been reported to be increased in T2DM in adult, our data did not show increase in T2DM in children. Those discrepancies may remain to be solved in future.

**Conclusions**: GDF15 was lower in T1DM in children than those in controls and T2DM, suggested that GDF15 may be a useful biomarker for T1DM in children.

## P1-1-11 Differences level plasma visfatin on obese adolescent based on insulin resistance level acording to homeostasis model assessment insulin resistance

### Eka Agustia Rini^2^, Indra Ihsan^1^, Rismawati Yaswir^1^

#### ^1^APPES, Paediatric Endrocrinology M. Djamil Hospital Padang; ^2^ISPAD, Paediatric Endocrinology M. Djamil Hospital Padang

**Background**: Adipose tissue has been proven to be not merely a site for energy storage but also the largest endocrine organ that secretes various adipocytocine. Plasma visfatin is predominantly secreted from visceral adipose tissue, has insulin-mimetic effects and the level closely linked to insulin resistance.

**Objective**: The aim of this study was to investigate the differences plasma visfatin level between obese and non obese adolescents and also between obese adolecent with insulin resistance and without insulin resistance.

**Methods**: This was a cross-sectional study and conducted in three Senior High School in Padang. Twenty eight obese adolescents and 28 control were enrolled in the study. The age of the subjects ranged from 14-18 years. Obesity criteria was measure based on body mass index (BMI). Fasting serum glucose level measured by glucose hexokinase fotometrik method and serum insulin was measured by chemiluminessence immunoassay method. Plasma visfatin was measured with enzyme-linked immunosorbent assay (ELISA). The insulin resistance index was estimated from fasting serum insulin and glucose level using the formula. Differences in the variables were tested using independent t-test and Mann-Whitney depending on the distribution of the variables.

**Result**: The plasma visfatin level was higher in the obese than in the control group 2,55 (SD 1,54) ng/ml vs 1,61 (SD 0,64) ng/ml, p: 0,005, and the insulin resistance group had higher plasma visfatin level than non resistance group 3,61 (SD 1,59) vs 1,96 (SD 1,18), p: 0,004

**Conclusion**: There were increasing level plasma visfatin toward increase of HOMA-IR in obese adolescents.

Key word: obese adolescents, insulin resistance, plasma visfatin.

## P1-1-12 Survey about Carbohydrate Education for the childhood and adolescent type 1 diabetes

### Tomoyuki Kawamura^1^, Masakazu Hirose^1^, Yuko Hotta^1^, Sachi Tsujiai^5^, Yuri Ota^5^, Hiroko Yoshioka^5^, Kayako Hashimura^1^, Takashi Higashide^2^, Yoneo Kashihara^2^, Tomomi Hashimoto^2^, Kayo Kimura^3^, Aono Mayumi^4^, Aono Shigeo^4^

#### ^1^Pediatrics, Osaka City University Graduate School of Medicine; ^2^Hug-Hug Kids Clinic; ^3^Kimu Clinic; ^4^Terada-cho Child Clinic; ^5^Osaka City University Hospital, Pediatric ward

**Introduction**: Carbohydrate Counting (CC) has been going to be popular for type 1 diabetes (T1D) in these days in Japan. It has passed ten years since we published the first Japanese text about CC of Japanese foods in 2006, and have started to educate our patients CC first in Japan. For educating CC to the childhood T1D, their age and development should be considered. We developed the original education method of CC. It is comprised with two parts of, the estimation carbohydrate amount and the calculation of insulin dose. The “Carb” as a unit which is equal to 10 g of carbohydrate is used. For the training of the estimation, the “Carb-flashcards” are applied. For the training of calculation, the simplified calculation sheets are used. There is few reports about the CC education for the childhood and adolescent T1D so far.

**Objectives**: To elucidate the educative status of CC among T1D patients in our clinic.

**Methods**: The questionnaire was carried out to T1D patients in our clinic.

**Results**: Data were obtained from 108 patients or 64 parents. The age of patients was 3-45 years old (Median 17 years old), The disease duration was 0-20 years (Median 8 years). The ratio of MDI/Pump was 46%/56%. The first education of CC was provided, 27% was only for the parents, 41% was for both the patients and parents, and 26% was only for the patients. In the cases of infant and toddler (below 6 years old), the CC education was usually provided only for the parents. More than 60% of patients above 9 years old can use CC by themselves. Some patients at 7 years old can estimate the amount of carbohydrate in food and calculate the insulin dose by CC by themselves. Seventy-one percent of patients use the “Carb”. The others use “gram”. Among the patients using Pump therapy, 60 % of them use the bolus calculator function of the pump. Many patients have mastered CC with 1 years. The duration to master CC, has become shorted significantly in the recent onset patients. That was not related to the therapies of MDI or pump.

**Conclusion**: The CC education should be begun at 6-7 years old of patients. Our recent education method of CC has become efficient.

## P1-1-13 Trends in the prevalence of extreme obesity among Korean children and adolescents from 1998 to 2014

### Hyo-Kyoung Nam, Hye Ryun Kim, Young Jun Rhie, Kee-Hyoung Lee

#### Department of Pediatrics, College of Medicine, Korea University

**Objectives**: Although recent studies reported that prevalence of childhood obesity stabilized around early 2000s, national trends of extreme obesity in Korea have not been studied. We aimed to assess the prevalence of obesity and extreme obesity for Korean children and adolescents and the impact of extreme obesity on metabolic syndrome.

**Subjects and Methods**: Data for 21,402 children and adolescents aged 2 to 19 years (11,188 boys and 10,214 girls), were obtained from the Korean National Health and Nutrition Examination Surveys during 1998-2014. We used age- and sex-specific body mass index (BMI) percentiles and examined the trends in the prevalence of obesity and extreme obesity based on criteria suggested by the Korean national reference standard (KCDC) and an US Centers for Disease Control and Prevention (CDC). We used the definition of metabolic syndrome from the modified the National Cholesterol Education Program Adult Treatment Panel III (NCEP ATP III).

**Results**: The prevalence of extreme obesity using the KCDC criteria was higher than using the CDC criteria. No significant increases in the prevalence of both overweight and obesity were found among boys and girls aged 2-19 between 1998 and 2014. Significant increases in the prevalence of extreme obesity were detected among adolescents aged 10-19, especially in boys. The rates of the metabolic syndrome combined with extreme obesity were 51.6% and 34.4% in teenage boys and girls, respectively. The odds ratios of extreme obesity on metabolic syndrome compared with normal BMI were 60.7 and 34.7 in teenage boys and girls, respectively.

**Conclusions**: Despite the prevalence of childhood overweight and obesity in South Korea stabilized since the early 2000s, the prevalence of extreme obesity is increasing especially in teenage boys. Given the greater morbidity risks for the extreme obesity, monitoring and preventing progression to extreme obesity become more important.

## P1-1-14 The seasonal variations of HbA1c chronologically reduced with improvement of glycemic control

### Mie Mochizuki^1^, Toru Kikuchi^2^, Tatsuhiko Urakami^3^, Nobuyuki Kikuchi^4^, Tomoyuki Kawamura^5^, Kisho Kobayashi^6^, Koji Kobayashi^1^, Nobuo Matsuura^7^, Nozomu Sasaki^2^, Shigetaka Sugihara^8^, Shin Amemiya^2^, The Japanese Study Group of Insulin Therapy for Childhood and Adolescent Diabetes (JSGIT)^9^

#### ^1^Department of Pediatrics, University of Yamanashi, Department of Pediatrics; ^2^Saitama Medial University; ^3^Department of Pediatrics and Child Health, Nihon University School of Medicine; ^4^Department of Pediatrics, Yokohama City University Medical Center; ^5^Department of Pediatrics; ^6^Kobayashi childrens clinic, Osaka City University Graduate School of Medicine; ^7^Department of Early Childhood Education, Seitoku University; ^8^Department of Pediatrics, Tokyo Women's Medical University Medical C Kobayashi Clinic enter East; ^9^The Japanese Study Group of Insulin Therapy for Childhood and Adolescent Diabetes (JSGIT)

**Objective**: The Japanese Study Group of Insulin Therapy for Childhood and Adolescent Diabetes (JSGIT) showed that glycemic control in Japanese children with type 1 diabetes had improved through changes in insulin regimens and using a combination of insulin analogues since 1995. Fluctuation of HbA1c values, well-known seasonal variations, are composed of high HbA1c values in winter and low HbA1c values in summer in diabetic patients. Although there are various reports of improved glycemic controls in diabetic patients, little is known about whether the seasonal variation has changed with times. This study was driven by the question of how the seasonal variation of glycemic control in patients with type 1 diabetes had changed from 1995 to 2012, using the data of 1995, 2000 and 2008 cohorts of JSGIT.

**Methods**: Participants included 662 patients in the 1995 cohort, 791 patients in the 2000 cohort and 859 subjects in the 2008 cohort. We conducted longitudinal analyses on changes in seasonal variations of HbA1c. Seasonal variation per year, HbA1c, was calculated as HbA1c value in winter minus HbA1c value in summer. Statically analysis were performed by linear regression or Mann–Whitney U test.

**Results**: Average HbA1c values have decreased year by year (average HbA1c (%) = -0.0672*year +143.1543, P<0.0001). HbA1c values in winter were higher than those in summer, with statistically significance in following periods except 2003 and 2007 (P<0.05). HbA1c had become smaller year by year (average HbA1c (%) = - 0.0032*year + 6.7736, P=0.0440).

**Conclusions**: HbA1c values in Japanese children with type 1 diabetes had significantly improved with seasonal variation; almost 0.22%. The seasonal variations of HbA1c were chronologically improved, but there were obvious seasonal variations in 2012. Further study is warranted whether advanced treatment and support for type 1 diabetes may ameliorate seasonal variation of glycemic control.

## P1-1-15 Differences in levels of Retinol Binding Protein 4 ( RBP4 ) on Adolescent Obesity Based on Insulin Resistance Levels According Homeostasis Model Assessment Insulin Resistance ( HOMA - IR )

### Eka Agustia Rini^2^, Ronaldi Noor^1^, Eti Yerizel^1^

#### ^1^APPES, Paediatric Endrocrinology M. Djamil Hospital Padang; ^2^ISPAD, Paediatric Endocrinology M. Djamil Hospital Padang

**Background**: Obesity has become a global problem and continues to be a major problem in many poor and developing countries and has reached the alarm. Obesity leads to insulin resistance in childhood. Retinol binding protein (RBP4), secreted primarily from the liver and adipose tissues, was recently proposed as a link between obesity and insulin resistance. The role of RBP4 in pediatric obesity and its relationship with insulin resistance are not elucidated.

**Objective**: The objective of the study was to determine the status of RBP4 levels in obese and lean adolescent and the relationship between RBP4 levels and insulin resistance based on Index of Homeostasis Model Assessment Insulin Resistance (HOMA - IR ).

**Method**: This was a cross-sectional study and conducted in three Senior High School in Padang. Samples included 14-18 years old obesity and normal weight adolescent (n=56). We measured body mass index (BMI) and serum RBP4. Insulin resistance was assessed using HOMA-IR index.

**Result**: Similar RBP4 levels were found in the obese vs. non- obese group group (p > 0,05). Higher RBP4 levels were found in the insulin resistance vs. non-insulin resistance group but the differences was not significant (p > 0,05)

**Conclusion**: There were no significant differences in mean levels of RBP4 in the group of obese adolescents compared with non- obese adolescents . So also in the group of obese adolescents with insulin resistance compared with those without insulin resistance

**Keywords**: Obesity, Retinol binding protein 4 (RBP4), insulin resistance

## P1-1-16 Fat or Fit- Search for predictors of metabolic complications in obese Indian children?

### Anurag Bajpai^1^, Rashmi Kapoor^2^, Rishi Shukla^1^, Manisha Gupta^1^

#### ^1^Division of Pediatric Endocrinology, Department of Endocrinology, Regency Hospital Limited; ^2^Department of Pediatrics, Regency Hospital Limited

**Background**: Metabolic complications are the main cause of concern in obese children. Body mass index (BMI) alone is however not a reliable predictor of these complications as they are not observed in many children with very high BMI. This heterogenity in the prevalence of metabolic complications for the same level of BMI makes identification of their pointers a desirable goal.

**Aim**: To identify predictors of metabolic complications in Indian children with obesity.

**Design**: Ongoing prospective observational study (April 1 2015 onwards). 205 children (120 boys, 5.1-18 years) with obesity underwent clinical assessment, complication screening (oral glucose tolerance test, lipid profile, fasting insulin and SGPT) and dual enhanced X Ray absorpimetery (DEXA) scan for body composition (body fat percentage and android to gynoid fat ratio). Predictors for metabolic complications were assessed using a step wise linear regression model.

**Results**: Metabolic complications observed in study included low HDL in 103 children (50.2%), high triglyceride levels in 51 (24.9%), pre diabetes in 44 (21.4%) and type 2 DM in 12 (5.9%). Children with metabolic complications had higher waist circumference standard deviation score (SDS, p =0.01), DEXA assessed total body fat percentage (p = 0.01) and android to gynoid ratio (p = 0.02) but similar body mass index (BMI) SDS (p = 0.1) compared to those without metabolic complications. HOMA IR levels correlated significantly with body fat, android to gynoid ratio and waist circumference SDS but not with body mass index SDS.

**Conclusions**: Metabolic complications are common in Indian children with obesity. High waist circumference SDS, AG ratio and total body fat are predictors to these complications. Their assessment would help target preventive and therapeutic interventions in obese children.

**Fig. 1 (abstract P1-1-16).**
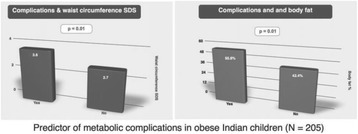
Predictor of metabolic complications in obese Indian children (N = 205)

## P1-1-17 Detection of common pathogenic genes in children with special type of diabetes mellitus and its clinical application

### Zhuhui Zhao, Wei Lu, Ruoqian Cheng, Li Xi, Xiaojing Li, Rong Ye, Zhong Lu, Dijing Zhi, Miaoying Zhang, Zhangqian Zheng, Feihong Luo

#### Department of Pediatric Endocrinology and Inborn Metabolic Diseases, Children's Hospital of Fudan Universit

**Objectives**: To explore the clinical value of common pathogenic gene detection in the diagnosis and treatment in hyperglycemia infants and children.

**Subjects and Methods**: Subjects were in-patients with hyperglycemia, age of onset before 1 year-old Cor insulin antibody negative and with family history of diabetes. Gene sequencing for ABCC8 , KCNJ11 , INS and GCK were performed and potential mutations were analyzed. The patients with ABCC8 and KCNJ11 gene mutations were treated with sulfonylurea, patients with GCK mutations were given the lifestyle intervention and others with insulin.

**Results**: Total 21 patients were enrolled, 15 patients were found with pathogenic gene mutations, 52.4% in ABCC8 gene and KCNJ11 gene (11/21). The patients with KCNJ11 or ABCC8 gene mutation are with average age 2.01 ± 1.62 months or 2.52 ± 2.60 months, respectively. GCK gene mutations were detected in children with age of onset more than or equal to 12 months, at 58.33 ± 43.02 months of age. There existed significant statistical difference among the onset ages of the three genetic variants, P = 0.001. The onset random blood glucose levels were significantly higher in the patients with INS gene mutation (66.70 mmol/L) than those of GCK gene mutation patients (9.73 + 1.97mmol/ L, P=0.003). 11 patients with ABCC8 or KCNJ11 gene mutation were treated with sulfonylurea and 9 patients reached euglycemia.

**Conclusions**: Mutations in potassium channel related genes (KCNJ11 and ABCC8) were the most common cause of neonatal diabetes in Chinese. Sulfonylurea therapy was effective and euglycemia were reached in most of the patients with the mutations in KCNJ11 and ABCC8 . Patients who were diagnosed hyperglycemia before 1 year-old Cor with negative antibody testing and family history of diabetes were referred for gene testing, even by targeted next-generation sequencing of all known related genes. The target therapy based on gene diagnosis is more effective and improvement of life quality.

## P1-1-18 Maturity-onset diabetes of the young (MODY) in Hong Kong Chinese pediatric population - Are we different from the West?

### Lai Ka Samantha Lee^1,2^, Wai Chun Sammy Wong^2,3^, Ho Chung Yau^1,2^, Wing Yan Jennifer Tsang^1,2^, Yuet Ping Liz Yuen^2,4^, Gary Wong^1,2^

#### ^1^Department of Paediatrics, The Prince of Wales Hospital; ^2^Department of Paediatrics, The Chinese University of Hong Kong; ^3^Department of Paediatrics, The Alice Ho Miu Ling Nethersole Hospital; ^4^Department of Chemical Pathology, The Prince of Wales Hospital

**Objectives**: To investigate the period prevalence, clinical presentation, and molecular genetics of patients diagnosed MODY in the Chinese pediatric population of Hong Kong.

**Methods**: A retrospective study of Chinese patients with genetically confirmed MODY aged from birth to 18 years under the care of the 2 major pediatric departments of the Hong Kong New Territories East Cluster (NTEC) of Hospital Authority from 1st January 2010 to 31st December 2015. Non-Chinese patient was excluded. For the calculation of the estimated period prevalence, only patients aged below15 years were included as government population statistics stratified age at below 15 years.

The Electronic Patient Record System was employed to retrieve the clinical and genetic data. Descriptive statistics employed for data analysis.

**Results**: The period prevalence of Chinese patients with MODY, aged below 15 years, was 58.5 cases per 1,000,000 populations from 1st January 2010 to 31st December 2015 in the NTEC catchment areas. For those aged 18 years or below, there were 9 Chinese MODY patients, of whom 2 were related siblings. Two patients diagnosed MODY 3, the rest MODY 2. The F:M was 2:1 and mean age 10-year-5-month. All patients were non-obese. The presenting HbA1c was 5.3% - 6.7% in MODY 2, 7.5-7.7% in MODY 3. Family history was positive in 8/9, and 6/8 involved 2 generations (not counting the index patient). Anti-islet antibodies were tested and negative in 4 patients. A novel frame-shift mutation, heterozygous GCK c.1239delinsAA, p.(Tyr413*), was detected in the 2 related siblings, whereas the rest had been reported in non- Chinese studies.

**Conclusion**: Our study reported the first period prevalence of MODY 2 and 3 in Chinese pediatric patients in Hong Kong. This showed MODY was not uncommon in Chinese, as compared to the estimated prevalence of 68-108 cases per million (aged <= 25years) from 1996 to 2009 in UK (Shields et al. Diabetologia. 2010 Dec;53(12):2504-8). The limitation of our estimation included: (1) The case identification was based on clinical suspicion rather than protocol, (2) Due to limitation of age stratification of population statistics, only prevalence for those under 15 years was calculated. The clinical features were similar to reported in Caucasian studies. A larger sample is required to delineate any founder mutations for Chinese patients with MODY.

## P1-1-19 Micronutrient deficiency in overweight / obese adolescents: Dual burden of malnutrition

### Vandana Jain, Babita Upadhyaya, Anuja Agarwala

#### Department of Pediatrics, All India Institute of Medical Sciences

**Introduction**: Several micronutrients deficiencies affect functional and physical performance of the children. Frequent consumption of junk food may be associated with micronutrient deficiency. This study was conducted to assess macro- and micronutrient intake in overweight/ obese adolescents aged 10-16 yrs.

**Materials and Methods**: 214 overweight adolescents (147 boys) aged 10 to 16 y were enrolled. Dietary assessment was done by one day 24 hr recall and quantitative food frequency questionnaire methods. The intakes were compared with the Recommended Dietary Allowance (RDA) for age and gender for each child. Data was presented as mean ± SD and as proportion (%) with intake above or below the RDA.

**Results**: Mean age was 11.9 ± 1.6 y and BMI SDS 2.3 ± 1.1. The mean daily energy intake was 2922 ± 838 Kcal, with the mean contribution from protein, carbohydrate and fat

being 11.3%, 59.8% and 29.3%, respectively. Mean daily fiber intake was 8.6 ± 3.8 g. Energy and fat intake exceeded the RDA in 75% and 95% subjects, respectively, while fiber intake was less than the RDA in 95%. The mean micronutrient intake of the subjects and the proportion whose intake was less than the RDA is presented in Table 1. Deficiency of Iron, Vitamin B12 and Zinc was seen in more than 60% of the subjects, while Magnesium, Folic acid and Vitamin C intakes were optimal in a majority.

**Discussion**: While energy and fat intake exceed the RDA in overweight adolescents, they have lower intake of fiber and essential micronutrients, s u c h a s i r o n , B 1 2 a n d z i n c . O v e r w e i g h t adolescents have a dual burden of malnutrition and should be encouraged to have more servings of fruits, vegetables and whole grains.

**Table 1 (abstract P1-1-19).**
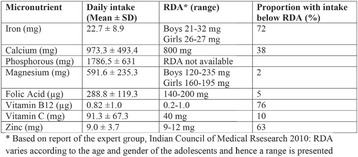
Mean micronutrient intake and proportion with intake less than RDA

## P1-1-20 A case of diabetic ketoacidosis associated with rhabdomyolysis and acute kidney injury

### Rieko Yazawa^1^, Noriyuki Takubo^2^, Akimi Ishikawa^2^, Takuro Iwasaki^2^, Tomoaki Yokokura^2^, Yu Shimizu^2^, Rie Yamaguchi^2^, Kei Fukushima^2^, Mayuko Tsubahara^2^, Tetsuo Shono^2^, Hidenori Haruna^2^, Toshiaki Shimizu^1^

#### ^1^Department of Pediatrics and Adolescent Medicine, Juntendo University Graduate School of Medicine; ^2^Department of Pediatrics, Juntendo University Faculty of Medicine

**Background**: Diabetic ketoacidosis (DKA) associated with rhabdomyolysis has previously been reported and may significantly influence primary care.

**Case**: A 14-year-old female patient with a one-week history of fatigue was admitted to our hospital with dyspnea and impaired consciousness in December 2015. A urine examination performed at her school when she was 8 -years -old had been positive for sugar. On admission, a biochemical examination of the patient’s urine and blood revealed the following: pH, 6.864, HCO3-, 4.7 mmol/L, serum glucose level, 716 mg/dL (39.7mmol/L), glycated hemoglobin, 15.8%, serum ketone level, 13,066 μmol/L, urinary C-peptide, 45 μg/d and positive for urine ketones and negative for pancreas–related autoantibodies. The patient was diagnosed with type 2 diabetes mellitus (T2DM) and DKA. Metabolic acidosis was attenuated by intravenous infusion with 10 mL/kg saline and 0.1 U/kg/h insulin. Within 21 h of therapy initiation, the levels of serum creatinine (Cre, 1.33 mg/dL, 117.6μmol/L), serum creatine kinase (CK, 2359 IU/L) and urinary myoglobine (20,000 ng/ mL) were increased, and the patient presented with rhabdomyolysis and acute kidney injury. The patient’s blood pressure and urine volume were maintained, and consciousness was gradually restored. As the blood glucose levels had not decreased after admission, the dosage of insulin infusion was increased to 0.2 U/kg/h. Subsequently, the blood glucose level was normalized and urinary ketones were undetectable. Serum Cre and CK levels were improved 32 h later. However, the patient presented with oliguria and systemic edema 43 h later, and was administered 0.2 g/kg albumin and 10 mg furosemide. Systemic edema and urine volume were improved from the 4th day following hospitalization, and serum Cre and CK levels were normalized on the 7th day. The patient was discharged on the 19th day.

**Conclusion**: In cases of severe DKA, the complication of rhabdomyolysis should be considered and the serum CK level and renal function should be carefully observed.

**Consent for publication:** The authors declare that written informed consent was obtained for publication.

## P1-1-21 NEONATAL HYPERINSULINEMIC HYPOGLYCEMIA, PARENTAL HISTORY OF DIABETES AND HNF4A MUTATIONS - PRELIMINARY OBSERVATIONS OVER 1 YEAR

### Suresh Chandran^1^, Hui Yi Chng^2^, Lim Joyce^3^, Sian Ellard^4^, Khalid Hussain^5^, Victor Samuel Rajadurai^6^, Fabian Yap^7^

#### ^1^Department of Neonatology, KK Women's and Children's Hospital; ^2^Paediatrics, Duke NUS Medical School; ^3^Paediatrics, NUS Yong Loo Lin School of Medicine; ^4^Paediatrics, Lee Kong Chian Imperial School of Medicine, Nanyang Technological University

**Background**: Early identification of HNF4A mutations in neonates with HH can facilitate the management of both the child and family.

**Aims**: To identify HNF4A mutations in neonates with prolonged hyperinsulinaemic hypoglycemia (HH) and parental history of diabetes.

**Methods**: Neonates delivered at our institution requiring glucose infusion rate (GIR) >10mg/kg/min after 48h of life from April 2015 to March 2016 were identified. We collected demographic data and determined if there was history of diabetes in the baby’s parents. We analyzed the HNF4A status of those with positive parental history of diabetes.

**Results**: There were 4 babies whose parents had pre-existing diabetes. Of these 4 neonates with parental history (2 male, all of Malay ethnicity), 1 had paternal and 3 had maternal history of pre-existing diabetes. Patient 1 (dad with diabetes from age 15y) was diagnosed with a heterozygous novel HNF4A missense mutation p.345Y, which was paternally inherited as a de novo mutation. Patient 2 (mum with diabetes from 18y) did not consent for genetic study. Patient 3 (mum with diabetes from 13y) and Patient 4 (mum with diabetes from 18y) were tested negative for HNF4A. These neonates were among twenty-five (56% male) neonates who satisfied the criteria for HH at 48h. These 25 HH neonates were out of 11923 babies born at KK Hospital over 1 year (incidence 2.1/1000). There were 10 Chinese (40%), 10 Malay (40%) and 5 babies of other races.

**Conclusion**: The presence of pre-existing diabetes in parents of neonates with HH may suggest a genetic etiology. Our preliminary data indicates that HNF4A gene testing should be performed in a larger number of neonates with HH and positive parental history of pre-existing diabetes before a definitive recommendation can be made.

## P1-1-22 Insulin pump therapy in type 1 diabetes: The Indian experience

### Manpreet Sethi, Archana Dayal Arya

#### Division of Pediatric Endocrinology / Department of Pediatrics, Sir Ganga Ram Hospital

**Background**: Insulin pumps have been used for the management of type 1 diabetes for over 15 years in the west. However similar experience is lacking in India where multi dose insulin injections still form the mainstay of management of type 1 diabetes. In a first study of its kind from India, we attempt to highlight the effectiveness, safety and superiority of insulin pump use in type 1 diabetes.

**Objective and hypotheses**: To determine the impact of insulin pump therapy on short and long term glycaemic control, body mass index (BMI), rate of severe hypoglycaemia and diabetic ketoacidosis (DKA) in children, adolescents and young adults.

**Method**: Retrospective analysis of data from case records of patients at our clinic. Out of the 64 patients on insulin pump, 52 were included in the study. Age of the patients at initiation of insulin pump ranged from 3 years to 26 years. Reckoning age-wise categorisation at 5 year intervals, patients were stratified into 5 groups (0-5, 6-10, 11-15, 16-20, 21-25 yrs). Data regarding pre-pump HbA1C (average HbA1C in the 6 months before starting insulin pump), pre-pump BMI (BMI at last visit before pump), pre-pump episodes of hypoglycaemia and DKA and post pump HbA1C (at 6months and one year after pump initiation), post pump BMI (BMI at 6 months after pump) and post-pump episodes of hypoglycaemia and DKA (in the one year after pump) in each patient was recorded and compared.

**Results**: Of the 52 patients included, only 27 followed up for one year or more after initiation of pump and 25 had visits only upto 6 months. There was a drop in HbA1C and increase in BMI across all age groups with the maximum difference seen in the oldest age group after starting pump therapy. Only 3 episodes of DKA were recorded after pump therapy against 10 episodes in the pre pump period. However there were 3 episodes of severe hypoglycaemia in the post pump period as compared to 2 episodes in the pre pump period.

**Conclusion**: This study suggests that insulin pump therapy is effective, safe and superior in children, adolescents and young adults with type 1 diabetes.

## P1-2-1 Lower adiponectin, higher ALT level, lower AST/ALT ratio, and higher VAT value may suggest a risk for the onset of type 2 diabetes in obese children

### Yuki Yasuda, Hisafumi Matsuoka, Shigetaka Sugihara

#### Department of Pediatrics, Tokyo Women's Medical University Medical Center East

**Objective**: An association between type 2 diabetes mellitus and metabolic syndrome (MS) has been suggested in obese children. We compared the characteristics of fat accumulation, blood chemistry, and adipokine levels among obese children with type 2 diabetes or MS without type 2 diabetes. We then analyzed the threshold value of the risk factor for type 2 diabetes.

**Methods**: One hundred and seven obese children visiting our outpatient clinic were enrolled in this study. The subjects were divided into 3 groups: Group A, obese children with type 2 diabetes (n = 19); Group B, obese children with metabolic syndrome but without type 2 diabetes (n = 19); and Group C, obese children without type 2 diabetes or metabolic syndrome (n = 69). Biochemical examinations and the measurement of serum adiponectin and leptin levels were performed for each patient. The visceral adipose tissue (VAT) and subcutaneous adipose tissue (SAT) levels were measured using cross-sectional computed tomography (CT) images obtained at the umbilical level. An ROC analysis was performed according to the presence of MS or diabetes mellitus. The statistical analysis was performed using JMP pro 12. This study was approved by the ethics committee of Tokyo Women’s Medical University.

**Results**: Group A tended to have higher VAT values and VAT/SAT ratios and lower leptin and adiponectin levels, compared with Groups B and C. In Group A, the serum ALT levels were significantly higher and the AST/ALT ratio was significantly lower than those in Group C. In Group B, the VAT and SAT values were higher than those in Group C. An ROC analysis for the onset of type 2 diabetes showed that the optimal cut-off point for adiponectin was 6.4 μ g/mL (AUC = 0.859), while those for ALT, the AST/ALT ratio, and VAT were 35 IU/L (AUC = 0.821), 0.85 (AUC= 0.794), and 78 cm2 (AUC = 0.713), respectively. No significant correlation between the HbA1c and adiponectin levels was seen in obese children without diabetes. Group A had a significantly higher frequency of a family history of type 2 diabetes than Group B.

**Conclusion**: These results suggest that an adiponectin level of less than 6.4 μ g/mL, an ALT level of more than 35 IU/L, an AST/ALT ratio of less than 0.85, and a VAT value of more than 78 cm2 may indicate a risk for type 2 diabetes in obese Japanese children.

## P1-2-2 South Indian mothers with gestational diabetes mellitus have normal BMI: Offspring have markers of neonatal adiposity

### Ahila Ayyavoo^1,2^, Thiyagarajan Satheesh^1^, Satheesh Gayathri^1^, Palany Raghupathy^1^

#### ^1^Department of Pediatric Endocrinology, G. Kuppuswamy Naidu Memorial Hospital; ^2^University of Auckland, Liggins Institute

**Background**: Studies from developed countries have exposed an elevated risk for obesity and type 2 diabetes mellitus in individuals exposed to gestational diabetes mellitus(GDM) during fetal life. Maternal obesity and pregnancy weight gain are risk factors for development of GDM. Risk for GDM is seen rarely in non-obese mothers. GDM affects up to 17.8% of pregnancies in urban south India, 13.8% in semi-urban areas and 9.9% in rural regions. Children born to GDM mothers have disproportionate growth and are prone to complications in the newborn period and later life.

**Aim**: Analyse the growth of neonates born to GDM mothers in south-India using Ponderal index and other anthropometry.

**Methodology**: Birth weight, gestational age, head, chest & mid-arm circumference and length of neonates born at GKNM Hospital, Coimbatore from April 2014-March 2015 were recorded 0-48 hours after birth. History of maternal diabetes, antenatal illnesses, pre-pregnancy weight, weight before delivery and mother’s height were recorded from clinical records. Ponderal index was calculated as weight(grams) x 100/ length3(cm) and body mass index(BMI) was calculated as weight(kilograms)/ length2(metres).

**Results**: 745 babies were analysed (GDM: non-GDM = 192:553). Ponderal index was elevated in GDM offspring (mean difference=0.06; p=0.008). Mid-arm circumference (mean difference=0.28cms;p=0.001) and chest circumference(mean difference=0.44cms;p=0.01) were higher in neonates of GDM mothers. Head circumference (p=0.25), length (p=0.06) and birth weight (p=0.17) were similar in both groups. Proportion of boys: girls (p=0.99), duration of gestation (p=0.25), socio-economic status (p=0.66) and birth order (p=0.78) were similar too. Caesarean and instrumental deliveries were high amongst GDM mothers (79% in GDM and 68% in non-GDM;p=0.01). Pre-pregnancy BMI of GDM mothers (mean=24.94): non-GDM (mean=23.56) were within normal range, though mean difference was 1.38(p=0). GDM mothers were heavier in the pre-pregnancy period (mean difference = 2.283kgs; p=0.008). But, pregnancy weight gain was higher in non-GDM (11.96kgs) than GDM mothers (10.97kgs; p=0.019). No difference was noted in Ponderal index with respect to treatment modalities for GDM (p=0.95).

**Discussion**: Babies born to GDM south Indian mothers have disproportionate body composition including elevated Ponderal index, mid-arm & chest circumference suggestive of neonatal adiposity even for minor variations in BMI(difference between groups=1.38) within the normal range and lower pregnancy weight gain. Normal Indian neonates have intrauterine origin of adiposity, central adiposity and hyperinsulinemia. As observed in the Pima Indians, GDM increases the risk for later obesity in the offspring irrespective of maternal obesity.

**Conclusion**: Risk for later diabetes, obesity and other metabolic disorders in the unique non-obese Indian GDM scenario could be elucidated only in larger longitudinal studies.

## P1-2-3 A Novel heterozygous mutation of WFS1 gene leading to constitutive ER stress is the cause of Wolfram syndrome

### Shuntaro Morikawa^1^, Toshihiro Tajima^1,2^, Akie Nakamura^1,3^, Takeshi Yamaguchi^1^, Katsura Ishizu^1^, Tadashi Ariga^1^

#### ^1^Department of Pediatrics, Hokkaido University Graduate School of Medicine; ^2^Department of Pediatrics, Jichi Children's Medical Center; ^3^Department of Molecular Endocrinology, National Research Institute for Child Health and Development

**Background**: Wolfram syndrome (WS) is a disorder characterized by the association of early-onset, insulin-dependent diabetes mellitus, diabetes insipidus, deafness, and progressive optic atrophy. The disease is caused by mutations of WFS1 gene located on 4p16 encoding protein that is called WFS1. This protein composes the tetramer which is expressed especially in pancreatic islet β -cells and the resident component of the endoplasmic reticulum (ER) membrane. During the protein synthesizing process in ER, the accumulation of misfolded and unfolded protein occurs in a certain frequency (known as “ER stress”). This ER stress is attenuated by the activation of the unfolded protein response (UPR), which recovers and maintains the ER function. Since WFS1 is known as the component of UPR, the mutant WFS1 results in unresolvable ER stress conditions and cell apoptosis. This is the major cause underlying the development of symptoms in WS.

**Case report**: We report a Japanese female WS patient with novel heterozygous mutation in WFS1 gene. She was admitted to our hospital for poor weight gain and DM. Her growth failure was evident at 3 months old and congenital cataract was noticed at 7 months old. Her auditory brainstem response (ABR) test revealed severe bilateral hearing loss. Her WFS1 gene showed a heterozygous twelve bases deletion in exon 8, resulting in 4 amino acid in-frame deletion (p.N325_I328del). Methods: We analyzed the functional consequence of the patient WFS1 using luciferase assay, western blotting and fluoresence analysis in vitro .

**Results**: (1) The patient mutant WFS1 (p.N325_I328del) raises the ER stress, ATF6 reporter activity and NFAT reporter activity in the absence of thapsigargin, indicating that this mutation induces a constitutive ER stress and elevation of the cytoplasmic Ca2+ concentration. (2) p.N325_I328del monomer has a dominant negative effect over the wild type monomer. (3) Induced constitutive ER stress is canceled by the chemical chaperone (4-phenylbutyric acid). (4) p.N325_I328del reduces the mRNA expression levels of SERCA2b (sarcoplasmic/endoplasmic reticulum Ca2+ transport ATPase 2b).

Since SERCA2b is required for ER and cytoplasmic Ca2+ homeostasis, these results raise the possibility that p.N325_I328del causes constitutive ER stress and cell apoptosis through ER Ca2+ depletion.

**Conclusion**: We report a severe WS patient caused by novel heterozygous mutation of WFS1 gene. The patient mutant WFS1 induces constitutive ER stress and cell apoptosis through Ca2+ efflux from ER. These results provide new insights into the roles of WFS1 in UPR mechanism.

**Consent for publication:** The authors declare that written informed consent was obtained for publication.

## P1-2-4 Sodium pyruvate treatment improved endogenous insulin secretion in a patient with mitochondrial diabetes

### Tadayuki Ayabe^1^, Takeshi Inoue^2^, Yuji Oto^2^, Nobuyuki Murakami^2^, Yasutoshi Koga^3^, Ryoichi Sakuta^2^

#### ^1^Department of Molecular Endocrinology, National Research Institute for Child Health and Development; ^2^Department of Pediatrics, Dokkyo Medical University Koshigaya Hospital; ^3^Department of Pediatrics and Child Health, Kurume University Graduate School of Medicine

**Background**: Mitochondrial diabetes is a rare form of diabetes mellitus, and reveals progressive decline in endogenous insulin secretion. Sodium pyruvate treatment has been reported to be a potential therapeutic choice for fatigability in patients with mitochondrial diseases. However, the effect of sodium pyruvate treatment for glucose intolerance in patients with mitochondrial diabetes remains to be clarified.

**Case presentation**: Water-based sodium pyruvate solutions (0.5 g/kg/day) were administrated orally to a Japanese man with mitochondrial diabetes and myopathy caused by the m.14709T-C mutation. He was already diagnosed with diabetes mellitus and started insulin self- injection. He did not have any kind of islet autoantibodies. To evaluate therapeutic effects, we measured urinary C-peptide, HbA1c and total daily insulin dose (TDD) 6 months later. His urinary C-peptide level improved from 4.3 to 17.2 mcg/day after 1 day and to 30.2 mcg/day after 6 months of sodium pyruvate treatment. He experienced no adverse event such as diarrhea resulting from sodium pyruvate treatment, except episodes of mild hypoglycemia. To avoid hypoglycemia, his TDD could be reduced from 33 Units/day to 20 Units/day. Despite reduction of TDD, his HbA1c declined from 6.5% to 5.9%.

**Conclusions**: Sodium pyruvate treatment improved endogenous insulin secretion and resulted in reduced TDD in a patient with mitochondrial diabetes. Sodium pyruvate treatment may be a potential therapeutic choice for patients with mitochondrial diabetes.

## P1-2-5 Impact of change in leptin/adiponectin and MDA-LDL in childhood obesity associated with eosinophilic inflammation of the airway and whole body

### Norihiko Azuma, Tomoko Ootani, Yuuki Yasuda, Hisafumi Matsuoka, Shigetaka Sugihara

#### Pediatrics, Tokyo Women's Medical University Medical Center East

**Background**: Childhood obesity has been suggested to be a risk factor for bronchial asthma. However, the pathological association between body fat accumulation and eosinophilic inflammation in the airway is not well known.

**Objective**: We examined the relationship between clinical factors of obesity and eosinophilic inflammation based on fractional exhaled nitric oxide (FeNO) measurements and peripheral blood eosinophil counts.

**Methods**: Thirty-two obese children (age: 6-15 years, mean age: 10.5 years) attending our outpatient clinic were enrolled in this study. Cases with diabetes mellitus, any endocrine disease, or bronchial asthma were excluded. We measured the height, weight, waist circumference, blood pressure, and FeNO of each subject. Blood samples were taken to examine the blood eosinophil count, IgE, AST, ALT, γ -GTP, TG, HDL- Chol, MDA-LDL, uric acid, insulin, hs-CRP, leptin, adiponectin and PAI-1 level in the morning after an overnight fast. The correlations between these values and FeNO or the peripheral blood eosinophil counts were then analyzed. A multivariable analysis was performed using FeNO and the peripheral blood eosinophil counts as objective variables. The statistical analysis was performed using JMP pro 12. This study was approved by the ethics committee of Tokyo Women’s Medical University.

**Results**: The mean BMI-z score of the 32 obese children in this study was 1.8. No single correlation between either FeNO or the eosinophil counts and the BMI-z score was found. A significant single correlation between FeNO and MDA-LDL was found (r = 0.38, P = 0.034). A multiple regression analysis revealed that leptin/adiponectin , leptin, adiponectin, the eosinophil counts, and γ GTP were selected for the

FeNO model (R2: 0.70, P < 0.0001), while AST/ALT, ALT, MDA-LDL, and IgE were selected for the eosinophil count model (R2: 0.64, P < 0.0001). Discussion: FeNO reflects eosinophil inflammation of the airway. On the other hand, the peripheral eosinophil counts reflect systemic inflammation. Our data demonstrated that the increased visceral fat accumulation associated with adipokine changes, such as the leptin/ adiponectin level, and oxidative stress markers, such as MDA-LDL, were strongly correlated with eosinophilic inflammation in the airway and whole body.

**Conclusion**: Increased visceral fat accumulation during childhood obesity may induce eosinophilic inflammation in both the airway and the whole body through adipokine changes and oxidative stress.

## P1-2-6 The Risk Factors of Cardiac Autonomic Neuropathy in Youth with Type 1 Diabetes

### Hye Jin Lee^1^, Hwa Young Kim^2^, Hae Woon Jung^3^, Gyung Min Lee^4^, So Youn Kim^1^, Kyung A Jeong^1^, Keun Hee Choi^1^, Young Ah Lee^1^, Sei Won Yang^1^, Choong Ho Shin^1^

#### ^1^Division of Endocrinology and Metabolism / Department of Pediatrics, Seoul National University Children's Hospital; ^2^Division of Endocrinology and Metabolism / Department of Pediatrics, Kangwon National University Hospital; ^3^Division of Endocrinology and Metabolism / Department of Pediatrics, Kyung Hee University Medical Center; ^4^Division of Endocrinology and Metabolism / Department of Pediatrics, Konyang University Hospital

Cardiac autonomic neuropathy (CAN) is an often overlooked chronic and serious complication of diabetes. Reduced heart rate variability (HRV) with parasympathetic loss is the earliest subclinical marker of CAN. The purpose of this study was to investigate predictors for reduced overall HRV and parasympathetic loss in youth with childhood-onset type 1 diabetes mellitus (T1DM) without microvascular complications.

A total of 113 patients with T1DM (19.7 ± 4.4 years, 55 males), who were followed up ≥ 5 years, were enrolled between January, 2014 and June, 2015 at Seoul National University Children’s Hospital. To evaluate overall HRV and parasympathetic activity, time domain [standard deviation of mean NN intervals (SDNN) and root mean squared difference of successive NN intervals (RMSSD)] and frequency domain [total power (TP), high frequency (HF)] indices were measured using 5-min ECG recording using SA-2000E (Medicore Co. Korea). Multivariate regression model was constructed using possible covariates (age, sex, diabetes duration, mean HbA1c, diastolic blood pressure [BP], non-HDL cholesterol, smoking, drinking). The mean age at T1DM diagnosis were 7.9 ± 4.0 years and diabetes duration was 11.7 ± 4.4 years (mean HbA1c 8.3 ± 1.1%). The mean HbA1c and diastolic BP were inversely related to SDNN (P= 0.013 and P= 0.035, respectively) and TP (P= 0.012, and P= 0.007, respectively) after adjusting for covariates, indicating that both poor glycemic control and high BP were significant predictors for reduced HRV in patients with T1DM. The mean HbA1c and diastolic BP were also inversely associated with RMSSD (P= 0.012 and P= 0.002, respectively), HF (P= 0.014 and P= 0.007, respectively) after adjusting for covariates, suggesting that both poor glycemic control and high BP were significant predictors for parasympathetic denervation in patients with T1DM. Both poor glycemic control and hypertension (especially diastolic) were independent predictors for reduced overall HRV and parasympathetic denervation in youth with T1DM. Optimizing glycemic and BP control are important to maintain HRV and prevent CAN.

## P1-2-7 Metabolic risk factors in Korean adolescents with severe obesity: Results from the Korea National Health and Nutrition Examination Surveys (K-NHANES) 2007-2014

### Cho Won Kyoung^1^, Han Kyungdo^2^, Ahn Moon Bae^1^, Park Yong-Moon^3^, Byung-Kyu Suh^1^, Min Ho Jung^1^, Yong-Gyu Park^2^, Shin Hee Kim^1^, Kyoung Soon Cho^1^, So Hyun Park^1^

#### ^1^Department of Pediatrics, College of Medicine, The Catholic University of Korea; ^2^Department of Biostatistics, College of Medicine, The Catholic University of Korea; ^3^National Institute of Environmental, Health Sciences, National Institutes of Health, Epidemiology Branch

**Backgrounds**: Severe obesity in adolescents has become an important global public health issue. However, little information is available for analyzing the associations between abnormal metabolic risk factors and severe obesity in Korean adolescents.

**Methods**: This is a cross-sectional study. Among 7197 subjects aged 10–18 years who participated in the 2007-2014 K-NHANES and checked anthropometric data, 1326 adolescents (male = 744, female = 582) with age and sex specific BMI ≥ 85th percentile were included. These obese adolescents were classified by increasing levels of BMI percentile according to the following categories: overweight (85th ≤ BMI 95th percentile), obesity (95th ≤ BMI 120% of 95th BMI), severe obesity ( ≥ 120% of 95th BMI or BMI 35, whichever was lower), and extreme severe obesity ( ≥ 140% of 95th BMI or BMI 40, whichever was lower).

**Results**: The prevalence of overweight, obesity, severe obesity and extreme severe obesity were 5.6%, 6.2%, 5.9% and 0.9% in 10-18-year- old Korean adolescents, respectively. Among the obese adolescents, the proportions of overweight, obesity, severe obesity and extreme severe obesity were 30%, 33.4%, 32%, and 4.7%, respectively. With increasing level of obese categoty, the mean levels of total cholesterol (TC, P for trend < 0.001), triglyceride (TG, P for trend <0.001), low density lipoprotein (LDL, P for trend <0.001), HbA1C (P for trend < 0.036), systolic blood pressure (SBP, P for trend < 0.001) were increased and high density lipoprotein (HDL, P for trend < 0.001) was decreased. With increasing level of obese categoty, the incidence of TC ≥ 200 mg/dL (P < 0.007), HDL 40 mg/dL or 50 mg/dL in girls older than 16 years-old (P <0.001), LDL ≥ 130 mg/dL (P < 0.004), TG ≥ 150 mg/dL (P < 0.003), HbA1C ≥ 5.8% (P < 0.006), SBP ≥ 130 mmHg (P < 0.003) increased, respectively.

**Conclusions**: Adolescents with severe obesity have more metabolic risk factors than less severe obese form adolescents. Early recognition of severe obesity is important for assessing and management of current morbidity in adolescents.

## P1-2-8 Associations Between Serum Vitamin D and Glucose metabolism in 3-year-old Japanese Children - NCCHD cohort study

### Yuta Chiba, Yasuko Ogiwara, Tomoko Yoshida, Yumiko Terada, Kazuko Mizutani, Yusuke Fujisawa, Kanako Nakao, Keisuke Yoshii, Yasuhiro Naiki, Reiko Horikawa

#### Division of Endocrinology and Metabolism, National Center for Child Health and Development

**Background and Objective**: Vitamin D (VD) is known to modify pancreatic β cell function and affects glucose homeostasis. VD deficiency can be a risk factor for insulin resistance and diabetes. In the previous studies, the associations between VD and glucose homeostasis were reported in late childhood and puberty. On the other hand, there were a few reports in early childhood about this relationship. The objective of this study was to examine the associations among serum concentrations of VD, glucose metabolism and its association factors in early childhood.

**Subjects and methods:** 653 infants (male = 341, 52.2%) at 3-years of age, participating in the NCCHD birth cohort were included in this study. Random blood samples were obtained during morning hours. Serum 25-hydroxyvitamin D (25(OH)D) was measured by LC-MS/MS. Serum insulin (IRI) was measured by CLIA, and plasma glucose level was measured by commonly used enzyme method. HOMA-IR and BMI were calculated from the biochemical and physical data. Statistical analysis was performed using linear regression analysis and t-test.

**Results**: Serum 25(OH)D levels were 23.8 ± 6.1 ng/ml imean ± SD Arange 9.0 `42.6 ng/ml ). There were no significant difference of 25(OH) D levels between male and female (23.9 ± 6.1 ng/ml vs 23.6 ± 6.1 ng/ml) . Significant negative correlation between 25(OH)D and serum glucose (r |0.22 Ap 0.01) was observed. The number of subjects with VD deficiency ( 20 ng/ml) and VD sufficiency (>20 mg/ml) were 28.2% (n=184) and 71.8 % (n=469), respectively. VD deficient group had significantly higher glucose level (73.7 ± 15.2 mg/dl vs 69.2 ± 15.8 mg/dl, p<0.01) and higher HOMA-IR (1.22 ± 1.25 vs 1.01 ± 0.82, p<0.02) than in VD sufficient group. Although there were nosignificant difference, VD deficient group tended to have higher insulin levels (6.3 ± 5.1 vs 5.7 ± 4.0, p 0.14) and BMI (15.8 ± 1.2 vs 15.6 ± 1.1, p 0.15) compared to VD sufficient group.

**Conclusion**: Although there is a limitation of interpretation since this study was conducted in the non-complete fast condition, our data suggested that Vitamin D deficiency may affect glucose homeostasis from infancy.

## P1-2-9 The ratio of glycated albumin to HbA1c estimates recent past glycemic control

### Ikuma Musha^1,2,8^, Toru Kikuchi^1,8^, Mie Mochizuki^3,8^, Junya Akatsuka^1,8^, Akira Ohtake^1,8^, Kisho Kobayashi^3,8^, Nobuyuki Kikuchi^4,8^, Tomoyuki Kawamura^5,8^, Tatsuhiko Urakami^6,8^, Shigetaka Sugihara^7,8^, Shin Amemiya^1,8^

#### ^1^Department of Pediatrics, Saitama Medical University, Department of Pediatrics; ^2^umagaya General Hospital; ^3^Department of Pediatrics, Yamanashi University, Department of Pediatrics; ^4^Yokohama City Minato Red Cross Hospital; ^5^Department of Pediatrics, Osaka City University Graduate School of Medicine; ^6^Department of Pediatrics and Child Health, Nihon University School of Medicine; ^7^Department of Pediatrics, Tokyo Women's Medical University Medical Center East; ^8^The Japanese Study Group of Insulin Therapy for Childhood and Adolescent Diabetes

**Objectives**: While HbA1c (A1C) has been used as the gold standard of glycemic control, it is difficult to estimate glycemic control within a month in case of the measurement every 3 or 4 months. We aimed to clarify whether the change in the ratio of glycated albumin (GA) to A1C could estimate the recent past glycemic control based on the difference in these half-lives.

**Methods**: We examined individual long-term consistency of GA/A1C ratio in 306 childhood-onset type 1 diabetes patients whose GA and A1C were measured simultaneously more than 10 times in the observational study every 4 months as a period. A concordance between a change of A1C during previous one month ( Δ A1C) and GA/A1C ratio at test was examined, where A1C value was expressed by both NGSP (National Glycohemoglobin Standardization Program) and IFCC (International Federation of Clinical Chemistry) numbers.

**Results**: We confirmed individual consistency of GA/A1C ratio by the significant correlation of quadric curve between the products of GA/ A1C ratio at different period over time. The concordance rates of Δ A1C with the difference between GA/A1C ratio at test and the intrinsic value as the individual mean of GA/A1C ratios were higher than those with SD score of GA/A1C ratio at test as a whole cohort. When Δ A1C improved ≤ -0.3% (NGSP) or ≤ -2.0mmol/mol (IFCC) and worsened ≥ +0.3% or ≥ +2.0mmol/mol, the concordance rate of Δ A1C using NGSP number was more accurate to estimate the deterioration of glycemic control. However, the concordance rate of improved recent past glycemic control was not significant in using either NGSP number or IFCC number. In addition, a statistically significant positive correlation was observed between GA/A1C ratio and A1C in NGSP number.

**Conclusions**: A change of GA/A1C ratio from the intrinsic value was useful to estimate the deterioration in the glycemic control within a month. The discrepancy of estimation was considered to depend on the difference of A1C expressed by IFCC or NGSP number, due to what is measured as A1C in each standardized system. Since A1C in NGSP number contains non-glycated hemoglobin, GA/A1C ratio may show a positive relation as A1C gets increased in the NGSP standardization. Thus estimation using NGSP number of deteriorated glycemic control was apparently higher than that using IFCC number.

## P1-2-10 Studay on autophagy genes expression and its regulation mechanism in rats with intrauterine growth restriction

### Min Yang, Ying Xin, Chao Yan Li, Dan Zhang

#### Department of Pediatric Endocrinology, Shengjing Hospital of China Medical University

**Background**: The mechanism of IUGR resulting in metabolic syndrome continues poorly understood and controversial. Normal development of pancreas is crucial for the normal structure and function maintenance. Some studies suggest that autophagy plays an important role in the development and maintenance of individual cell function. The aims of this work were: (1) to compare the differences in autophagy, between β -cells in normal rats and IUGR rats; (2) to study the regulation mechanism of autophagy in rats with IUGR.

**Methodology/Principal Findings**: Compared with normal rats, rats with IUGR had higher levels of autophagy-related proteins LC3B-II, beclin-1. Furthermore, cytotrophoblasts cultured under hypoxia (2% oxygen) in the presence or absence of nutlin-3 (a p53 activity stimulator) had higher levels of LC3B-II. Protein expression in IUGR group compared with the normal group. mTOR and P70S6K (Thr389) expression were decreased (P <0.05), P-ULK1 (Ser757) was up-regulated (P <0.05). High fat diet could activate mTOR and P70S6K (Thr389), and P-ULK1 (Ser757) expression was down regulated (P <0.05).

**Conclusion**: Elevated levels of autophagy were in pancreas of rats with IUGR in the middle-late develpment period, as well as that in adult rats, which might lead to poor development of pancreas. Decreased mTOR sinaling in IUGR rats could lead to the increase of autophagy. Autophagic activity of β cells of islet of IUGR rats may be normal in early adulthood.

## P1-2-11 A case study of immune dysregulation, polyendocrinopathy, enteropathy, X-linked syndrome

### Pin Li, Zhiying Zhu, Dandan Yuan, Shasha Zhou, Guoying Yao

#### Division of Endocrinology and Metabolism, Affiliated Children Hospital of Shanghai Communication University

**Objectives**: Immune dysregulation, polyendocrinopathy, enteropathy, X-linked syndrome (IPEX syndrome) is a rare recessive X-linked hereditary disease, which is characterized by different clinical manifestations including autoimmune endocrinopathies, refractory diarrhea and eczema. It is caused by FOXP3 gene mutation, which results in abnormality of regulatory T cell, leading to destruction of autoimmune stability, causing multiple symptoms, and therefore has poor prognosis. We would like to discuss the current research of the disease by studying the diagnose and treatment of a 6-year-old boy with IPEX syndrome, in order to avoid misdiagnosing.

**Methods**: The initial finding of the patient was insulin-dependent diabetes mellitus iIDDM ) when he was 2 years old. Repeated respiratory tract infections during the treatment suggested the immune dysregulation with a markedly increase IgE level. Diabetes nephropathy was diagnosed when he had proteinuria at the age of 4, with concomitant repeated intestinal infection, with eczema around the mouth, electrolyte disorders and malnutrition. Therefore, he was clinically diagnosed as IPEX syndrome and took a genetic analysis.

**Results**: We identified a missense mutation in his FOXP3 gene (c.1010G>A Cp.Arg337Gln). His mother was an asymptomatic female gene carrier (heterozygous) while his father had no mutation in this gene loci. Hence this child was diagnosed with IPEX syndrome and treated with methylprednisolone.

**Conclusions**: We’ve diagnosed the IPEX syndrome through the clinical features and gene analysis. Children with early-onset IDDM, refractory diarrhea and eczema should be considered the possibility of IPEX syndrome and FOXP3 gene analysis will provide a guiding suggestion in diagnosing. During the acute stage, supporting therapy including infection control, glucose regulation and parenteral nutrition should be taken to improve symptoms, while immunosuppressant agents or hematopoietic stem cell transplantation (HSCT) will play an important role in the further treatment.

## P1-2-12 Development of MyDiabetes website. A web-based education programme for children & adolescent with type 1 diabetes mellitus (T1DM)

### Rokiah Ismail^1^, Azriyanti Anuar^6^, Emma Foster^2^, Katie Haighton^3^, Timothy Cheetham^4^, Hilary Hartley^5^, Ashley Adamson^2^, Siti Zarina Yaakop^6^, Muhammad Yazid Jalaluddin^6^

#### ^1^University Malaya Medical Centre Dietetic Department; ^2^Newcastle University, Human Nutrition Research Centre; ^3^Newcastle University, Institute of Health & Society; ^4^Department of Paediatric Endocrinology, Institute of Genetic Medicine, Newcastle University; ^5^Department of Paediatric Endocrinology, Royal Victoria Infirmary, Newcastle upon Tyne; ^6^Department of Paediatrics, University Malaya Medical Centre, Endocrine Unit

**Background**: Diabetes education is essential in diabetes care. Internet can be a tool to provide educational intervention in children with T1DM.

**Aim**: The objective is to develop a web-based education programme to assist in diabetes management and to provide support for children with T1DM in Malaysia.

**Methods**: Data was collected in three phases using a mix-method approach. There were 91 participants; 64 children with T1DM, 12 parents and 3 clinicians from Malaysia. An additional 12 Malaysian children who were living in Newcastle were invited to participate in the study.

In phase one, the data was collected using both qualitative and quantitative method to understand the challenges of children with T1DM (n=52). They were asked to identify regularly consumed carbohydrate-rich foods. In phase two, a semi-structured interview and an open- ended questionnaire given to healthy children in Newcastle (n=12). This is to analyse views and general usability of the website. In the final phase, children with T1DM and their families (n=12) were recruited and introduced to the website. They were guided on how to use it at home for six months. Semi-structured interviews were conducted on these children, their parents and clinicians (n=27). An additional set of questionnaires were given to the children only (n=12) to analyse participants’ views, experiences and acceptance of the website.

**Results**: All children reported that the website is useful to obtain information on carbohydrate content of foods and drinks. They used it to adjust insulin accordingly. They reported that they have made changes in their food choices based on the information obtained from the website. Most of them did not record their blood glucose (BG) regularly into the BG meter diary in the website. The majority n=10 (83%) felt more confident in managing their diet, insulin, and monitoring BG. Seventeen percent (n=2) reported that the website did not help to improve confidence level or manage their diabetes better. The clinicians indicated that the website was helpful in the clinic setting to teach and review children’s BG and dietary intake. The clinicians reviewed that the website had suitable application for the children to use for self-education and self-management system.

**Conclusion**: This study suggested that the website provides additional benefit to the children and their parents in terms of improving knowledge on carbohydrate content, diet choices, insulin adjustment and confidence level. The website is useful for the clinicians to help educate children with T1DM during clinic visits.

## P1-2-13 Sleep Deprivation Increases the Risk for Obesity in Adolescents

### Ah-Reum Kwon^1^, Mo Kyung Jung^1^, Hyun Wook Chae^1^, Duk Hee Kim^2^, Ho-Seong Kim^1^

#### ^1^Department of Pediatric Endocrinology, Severance Children's Hospital, Yonsei University College of Medicine; ^2^Department of Pediatric Endocrinology, Sowha Children's Hospital

**Study Objective**: To investigate whether sleep deprivation is a risk factor for obesity in adolescents.

**Design**: Cross-sectional study.

**Setting**: Korean National Health and Nutrition Examination Survey.

**Participants**: Subjects were 4,126 adolescents, aged 12-18 years old, who participated in the Korean National Health and Nutrition Examination Survey IV and V, conducted between 2007 and 2012.

**Measurements and Results**: Subjective sleep duration was evaluated as a risk factor for obesity. Hours of sleep were self-reported. Anthropometric measurements, a questionnaire about medical history and lifestyle, and biochemical laboratory measurements were conducted. Body mass index (BMI) z-scores were decreased with longer sleep duration. Subjects were classified according to sleep duration as those who slept ≥ 9 h/night, those who slept 7 to 9 h/night, and those who slept < 7 h/night. Odds of obesity were assessed according to sleep duration. Adolescents who slept < 7 h/night had higher odds of being obese (OR = 1.39, 95% CI 1.04-1.87) than adolescents who slept ≥ 7 h/night. One additional hour of sleep duration reduced obesity risk 11% (OR = 0.89 95% CI 0.81-0.98) and decreased BMI z-scores at the rate of 0.08. These finding were more significant in boys than in girls. Physical activity, energy intake, and stress (as index scores) did not increase the risk for obesity.

**Conclusions**: Sleep deprivation increases the risk for obesity in adolescents, while the degree of physical activity and energy intake may be not associated with obesity.

## P1-2-14 Fulminant Type 1 Diabetes Mellitus in Japanese Children and Adolescents

### Kentaro Shiga^1,7^, Tatsuhiko Urakami^2,7^, Junichi Suzuki^2,7^, Yasuhiko Igarashi^3,7^, Hanako Tajima^4,7^, Shin Amemiya^5,7^, Shigetaka Sugihara^6,7^

#### ^1^Yokohama City University Medical Center, Children's Medical Center; ^2^Department of Pediatrics, Nihon University Hospital; ^3^Department of Pediatrics, Igarashi Children's Clinic; ^4^Nippon Medical School; ^5^Department of Pediatrics, Saitama Medical University; ^6^Department of Pediatrics, Tokyo Women's Medical University Medical Center East; ^7^The Japanese Study Group of Insulin Therapy for Childhood and Adolescent Diabetes (JSGIT)

**Objective**: Fulminant type 1 diabetes mellitus (FT1DM) is a subtype of T1DM characterized by remarkably abrupt onset. Reportedly, the frequency of FT1DM is approximately 20% of adult onset-T1DM in Japan. On the contrary, few reports have described FT1DM in pediatric patients. The purpose of this study is to determine the frequency and clinical characteristics of FT1DM in Japanese children and adolescents.

**Method**: A 2-phase questionnaire survey was sent to the members of the Japanese Study Group of Insulin Therapy for Childhood and Adolescent Diabetes (JSGIT) regarding their clinical experience with FT1DM with the age at onset below 16 years. This group is consisted of pediatric diabetologists from 79 hospitals and is the largest study group for pediatric T1DM in Japan.

**Results**: Responses to the questionnaires were obtained from 54 of 79 hospitals (collection rate, 68.4%). Overall, 9 hospitals had treated 17 pediatric patients with FT1DM (5 patients, 4 patients and 2 patients in 1 hospital each, and 1 patient each in 6 hospitals). The distribution of patient age was biphasic, including young children aged below 5 years and children above 8 years of age. The clinical characteristics of the disease in this population, such as severity of the symptoms at onset and precursor symptoms, did not differ from those of adult-onset FT1DM.

**Conclusion**: Among children and adolescents, the frequency of FT1DM is rather rare compared to those of adult. Since FT1DM is a life- threatening metabolic state, early as possible and accurate diagnosis and an appropriate therapy are crucial. Pediatricians should take enough notice of the diagnosis of FT1DM.

## P1-2-15 Combined therapy with pump and long acting insulin for type 1 diabetes mellitus caused by STAT1 mutation

### Takahiro Tomoda^1^, Misako Okuno^2^, Akihito Sutani^1^, Atsumi Tsuji-Hosokawa^1^, Kosuke Imai^1^, Kenichi Kashimada^1^, Tatsuhiko Urakami^2^, Tomohiro Morio^1^

#### ^1^Department of Pediatrics and Developmental Biology, Tokyo Medical and Dental University; ^2^Nihon University School of Medicine, Pediatrics and Child Health

**Background**: STAT 1 gain-of-function (GOF) is the most common genetic cause of autosomal dominant chronic mucocutaneous candidiasis (AD-CMC) and underlies a variety of infectious and autoimmune features. AD-CMC is a rare condition, and type1 diabetes mellitus (T1DM) is one of the concerning complications of GOF STAT1 mutation. However, clinical details of T1DM with AD-CMC have not been clarified. Here we report a clinical course of a case with STAT 1 GOF who developed T1DM at the age of three months.

**Case report**: Since developing T1DM, she received insulin therapy with continuous subcutaneous insulin infusion (CSII), and the control was acceptable for her age with 8.2% of HbA1c. Since the age of 3 years, she repeated to candidiasis at scalp, genitalia and oral cavity. She was referred to our hospital for closer examination for immunodeficiency, and heterozygous missense mutation (Met202Glu) in STAT1 was identified, diagnosing her with AD-CMC. Simultaneously, the control of her blood glucose became worse with more than 10% of HbA1c, and the control was not improved by introducing sensor augmented pump (SAP) at the age of 7 years. Her blood glucose fluctuated violently due to unstable effect of insulin therapy. By close monitoring, we learned that instability of insulin effect was more prominent on the second day after changing an infusion set and reservoir, requiring changing the set every 2 days. She frequently developed a subcutaneous nodule at infusion site, and we suspected that unexpected inflammation due to AD-CMC has occurred in site, resulting in unstable effects of the insulin therapy by blocking the tube or by affecting insulin absorption in site. For the sake of stable supply of insulin, we introduced injection of long acting insulin together with SAP, and her blood glucose level was remarkably improved from 11.3% to 8.7% of HbA1c.

**Discussion**: STAT 1 is involved in various signaling inflammatory pathways of IFN- γ , IFN- α / β , IL-27, IL-6 and IL-21. We assume that GOF mutation of STAT1 may activate those pathways, subsequently causing unexpected inflammatory reaction to insulin or to stimulation by catheter.

**Conclusion**: Insulin pump therapy accompanied with long acting insulin would be a useful option for the therapy of T1DM with STAT1 mutation.

## P1-2-16 Permanent neonatal diabetes mellitus due to a G32S heterozygous mutation in the insulin gene

### Xiao-Qin Xu, Ke Huang, Fang Hong

#### Division of Endocrinology, Children's Hospital of Zhejiang University, School of Medicine

## P1-2-17 Sensitivity and specificity of USG as compared to MRI for diagnosing fatty liver in overweight adolescents

### Vandana Jain^2^, Manisha Jana^1^, Alec Correa^2^, Babita Upadhyaya^2^

#### ^1^Department of Radiology, All India Institute of Medical Sciences; ^2^Department of Pediatrics, All India Institute of Medical Sciences

**Background**/ **Objective**: Non-alcoholic fatty liver disease (NAFLD) has emerged as a significant complication of childhood obesity affecting 30-50% of all obese children in various studies. Ultrasonography (USG) is typically used as the screening tool. The present study was undertaken to determine the sensitivity and specificity of USG for diagnosis of NAFLD in overweight adolescents as compared to measurement of hepatic fat fraction by MRI.

**Methodology**: The study was conducted at Departments of Radiology and Pediatrics, All India Institute of Medical Sciences, New Delhi after ethical approval and voluntary informed consent. Thirty-four overweight adolescents (mean BMI SDS 2.3 +/- 1.1), aged between 10 – 15 years were included in the study. They underwent grey scale sonography in a single state of the art ultrasound scanner by a single observer. NAFLD grading was done as 0 (absent), 1 (mild), 2 (moderate) and 3 (severe) on USG. Chemical shift MR imaging with dual echo Dixon method was performed. Fat fraction (FF) was calculated from the in phase and opposed phase images as FF= signal in in-phase (IP)- signal in opposed phase (OP)/ 2 x IP.

NAFLD grading in MRI was done as present (0) and absent (1); taking a cut off of 5% FF for the presence of fatty liver.

**Results**: Fatty liver was present in 22 (22/34; 64.7%) cases on USG, and 16 (16/34; 47.1%) on MRI. In 22 cases the MRI and USG findings were congruent. In three cases, MRI detected fatty liver with a fat fraction of 7.5, 9 and 11.9 but USG failed to detect fatty liver. In 9 cases, USG detected NAFLD but on MRI it was not designated as fatty liver as the fat fraction was < 5% (0.85- 4.9). In 8 of these 9 cases, the USG diagnosis was mild NAFLD. Considering MRI as the gold standard, the sensitivity and specificity of USG were 0.81 and 0.50, respectively.

**Conclusion**: USG is more susceptible to subjectivity and inter and intra-observer variability, which may miss mild NAFLD, as well as overdiagnose it. The sensitivity is acceptable but specificity is low. Hence USG can be used as a screening test for NAFLD, but the diagnosis should ideally be confirmed by estimation of hepatic fat fraction by MRI.

## P1-2-18 Think beyond type 1 and 2 diabetes

### Antony Fu

#### Princess Margaret Hospital, Paediatrics and Adolescent Medicine

A teenage girl initially presented to our department for short stature. She was a product of non-consanguinous parents with Chinese background. She has also been followed up by opthalmology and surgery for retinopathy and sensorineural hearing loss respectively.

Almost a year after presentation, she started to developed nocturia, polyuria and polydipsia. Investigations showed hyperglycemia, ketosis without acidosis, HbA1c 14.1 %, borderline low C-peptide and negative anti-islet cell antibody. She has been put on multi-dose insulin for presumably type 1 diabetes since then.

Another year after insulin treatment, she complained of anorexia and frequent abdominal pain. Subsequent workup revealed primary hypoparathyroidism. Therefore she was put on calcium supplement and caltriol with satisfactory response.

Putting diabetes, primary hypoparathyroidism, hearing loss and retinopathy together, we kickstarted the genetic study which confirmed the diagnosis of Kearns-Sayre syndrome. Her lactate and creatine kinase levels were normal, recent echocardiogram and electrocardiogram were both unremarkable. She was treated with Riboflavin and Coenzyme Q10 thereafter.

This case outlines the importance of thinking outside the box - especially when we encounter co-morbidities beyond the scope of traditional type 1 and 2 diabetes. Earlier recognition and treatment could make a difference.

**Consent for publication:** The author declares that informed consent was obtained for publication.

## P1-2-19 The Prevalence of Mental Health Issues among Thai Children and Adolescents with Type 1 Diabetes

### Somsongla Pirompuk^1^, Sunsanee Ruangson^2^, Jeerunda Santiprabhob^1^, Supawadee Likitmaskul^1^

#### ^1^Division of Endocrinology and Metabolism / Department of Pediatrics, Faculty of Medicine Siriraj Hospital, Mahidol University; ^2^Division of Psychology / Department of Pediatrics, Faculty of Medicine Siriraj Hospital, Mahidol University

**Introduction**: Type 1 diabetes mellitus (T1D) is a non-curable disease which requires continuous patient self-management for maintaining blood glucose control hence preventing related complications. Many studies have found associated incidences of psychiatric disorders in children and adolescents with T1D for example depression, anxiety or eating problems. Furthermore the pediatric patient’s parents have also suffered from conflicts over patient management of T1D and, in general, a lower quality of life resulting from the burden of taking care their children and taking time off work.

**Objectives**: Our study aims to determine the prevalence of mental health issues and to analyze several factors affecting patient’s mental health including gender, parental marital status, primary care givers, family income, duration of diabetes, insulin regimen and frequency of self-monitoring of blood glucose (SMBG) and the association between mental health issues and glycemic control.

**Methods**: A cross-sectional descriptive study had been done with T1D patients and parents who attended the pediatric diabetes clinic during 1 August 2015 to 31 January 2016 using standardized screening questionnaires. For the children, both the Children’s Depression Inventory (CDI) Thai version and the Eating Attitudes Test-26 (EAT-26) Thai version were used. For the parents, the Thai Youth Checklist (TYC) for analyzing behavioral and emotional problems was used.

**Results**: Seventy-nine T1D (50 females, 29 males) patients and sixty-eight care-givers were enrolled. Mean patients age was 12.3 ± 2.8 years, mean diabetes duration was 4.9 ± 2.7 years. The screening results found that T1D patients had positive behavioral/emotional problems, depression, and eating problems at 16.2% (11/68), 22.7% (17/75) and 33.3% (7/21), respectively. No positive screening results were found in patients with good glycemic control as noted by HbA1c of less than 7.5%. In addition, there were no associated factors between all mental health issues and glycemic control among patients with HbA1c <7.5%, 7.5–9% and >9% (p >0.05). The same could be said for the gender, primary care giver, family income, duration of diabetes, insulin regimen and number of SMBG per day factors. Nevertheless a divorced family status was significant indicator related to a patients’ eating problems (p = 0.024).

**Conclusions**: Childhood and adolescents with T1D have an increased risk of developing eating disorders, depression and behavioral/emotional problems, leading to deterioration of glycemic control, thus increased awareness as well as early detection in diabetic children and adolescents is required.

## P1-2-20 Adiponectin, Interleukin-6 and high-sensitivity CRP levels in overweight/ obese Indian children

### Vandana Jain^1^, Ajay Kumar^1^, Jaivinder Yadav^1^, Anuja Agarwala^1^, Lakshmy Ramakrishnan^2^, Naval Vikram^3^

#### ^1^Division of Endocrinology / Department of Pediatrics, All India Institute of Medical Sciences; ^2^All India Institute of Medical Sciences, Cardiac Biochemistry; ^3^All India Institute of Medical Sciences, Medicine

**Background/ objectives**: Interleukin (IL)- 6 and high-sensitivity CRP (hsCRP) are markers of inflammation associated with risk of cardiovascular disease and diabetes. Low Adiponectin levels are associated with insulin resistance. The aim of this study was to assess serum IL-6, hsCRP and Adiponectin and their correlation with conventional risk factors for cardiovascular disease and diabetes in overweight/obese Indian children.

**Methodology**: Children between 7-15 years, with BMI > 85th centile were enrolled from Pediatric outpatient at AIIMS, New Delhi. Weight, height, waist circumference (WC) and BP were measured. Fasting blood sample was drawn for glucose (BG), total cholesterol (TC), HDL cholesterol (HDL), triglycerides ( TG), adiponectin, IL-6 and hsCRP. The following cut-offs were taken to define abnormality: BG > 100mg/dl, TC ≥ 200mg/dl, LDL ≥ 130mg/dl, HDL 150mg/dl, Adiponectin 3 mg/L and IL-6 > 10 pg/ ml. Data was expressed as mean/ median and proportion with abnormal values. Correlations were checked between adiponectin, IL-6 and hsCRP and BMI, WC and BG.

**Results**: Eighty-four subjects (48 boys) with mean age 10.2 ± 1.9 yrs, and mean BMI z-score 2.7 ± 0.8 were enrolled. Two-thirds were prepubertal and 67.3% had abdominal obesity. Mean systolic and diastolic BP was 113 ± 10 and 74 ± 10 mm Hg, with hypertension in 15.5%. The biochemical parameters are summarized in Table 1. Low HDL was noted in 35.1%, impaired fasting glucose in 10.7% and low Adiponectin in 16.5%. High inflammatory mediators, IL-6 in 54.4% and hsCRP in 49.4% constituted the commonest abnormality. Adiponectin was inversely correlated with WC (r= -0.28, p=0.047). IL-6 was positively correlated with BMI (r=0.23, p= 0.09), and BG (r=0.24, p=0.08). IL-6 was higher in children with impaired BG as compared to those with normal BG (107 (IQR 22.5 – 197.5) vs. 8.5 (5-116) pg/ml, p=0.06).

**Conclusions**: Inflammatory mediators hsCRP and IL-6 were elevated in half of the subjects. Inverse correlation between Adiponectin and waist circumference and positive correlation between IL-6 with BMI and fasting glucose indicated the usefulness of these markers in pediatric population.

**Table 1 (Abstract 1-2-20):**
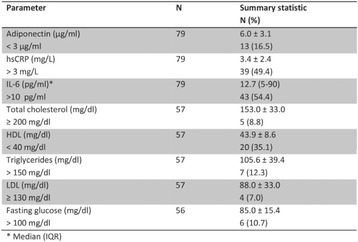
See text for description

## P1-2-21 Assessment of Oral Hygiene Status in Obese with Type 1 Diabetes Mellitus and Normal Weight Adolescents

### Zerrin Orbak^2^, Recep Orbak^1^, Yerda Ozkan^1^

#### ^1^Department of Periodontology, Ataturk University Faculty of Dentistry; ^2^Department of Pediatric Endocrinology, Ataturk University Faculty of Medicine

**Objective**: The oral hygiene level in obes with Type 1 Diabetes Mellitus needs to be elucidated. The purpose of this study was to evaluate the oral hygiene status in obese with Type 1 Diabetes mellitus and normal weight adolescents.

**Methods**: A cross-sectional survey of 13-15-year-old adolescents was conducted on a sample of totaly 36 who were admitted to pediatric endocrinology clinic in 2014 and 2015. The sample was divided into equal two groups: ODM (obese with Type-1 Diabetes Mellitus; n = 18; 11 boys and 7 girls, mean age 13.21) and N (normal weight; n = 18; 9 boys and 9 girls, mean age 14.01). The subjects were asked to complete a questionnaire concerning medical history and dietary/oral hygiene habits. For anthropometric evaluation, the body mass index (BMI)-for-age was used. In oral examinations, the study used Oral Hygiene Index (OHI) and Gingival Bleeding Index (GBI) for the evaluation the oral hygiene status. Statistical differences were evaluated Chi-square, odds ratio (OR), Wilcoxon and Pearson correlation tests were used (P < 0.05).

**Results**: In both groups, the participants displayed similar oral hygiene habits According to the total sample, 2/18 ODM (11.1 %) and 8/18N (44.4 %) had good OHI, while 7/18 ODM (38.9 %) and 2/18 N (5.5%) were classified in a low level of OHI, with a significance between the groups (p < 0.001), even after sorting by age. According to the classification of GBI, 18/18 ODM (100.0%) and 13/18 N (72.2%) had GBI 1 (bleeding gingiva), and 0/18 ODM and 5/18 N (27.7%) were classified as GBI 0 (healthy gingiva), with a significance between the groups (p <0.001), even after sorting by age.

**Conclusions**: This study indicated that oral hygiene level were significantly lower in the ODM group.

## P1-2-22 Construction of remote monitoring system of the elementary school upper grades health checkup data

### Takanori Motoki^1^, Maki Kariyazaki^2^, Satoko Tsuru^2^, Ichiro Miyata^1^

#### ^1^Department of Pediatrics, Jikei University School of Medicine; ^2^The University of Tokyo, Health Social System Engineering Laboratory

**Background**: A school nurse and a school physician do screening of short stature and overweight by height and weight measured in the school health examination. Furthermore, in the municipality, practitioners are doing medical examination to preventing for children’s unhealthy lifestyle to applicants. However the pediatric endocrinologists of the ward sometimes examine the first visit patients with short stature, highly obese or type 2 diabete patients who have passed a few years from the onset.

**Objective**: To reveal how many children who have the extent physique problems from height and weight data obtained from school health check of the ward there are. To build a regional cooperation system not to miss the patients who hospital consultation from the onset becomes too late. This study shows the change of the frequency the elementary school upper grades child with problems in physique of 2 years.<br />

**Methods**: We analyzed the height and weight data which had been input to the academic affairs system and which were the total of 14 times in the school medical examination from April 2011 to September 2015. Sharing the results with the Medical.

**Result**: We were able to obtain the data of 10,022/10031 children (in fiscal 2014/2015) to sixth graders from fourth graders who belonged to the public elementary school of the ward. 254/320 students with tall stature (Total 2.67%/3.19%: 4th Grade 2.55%/3.05%, 5th 2.79%/3.08%, 6th 2.36%/3.44%), 147/108 students with short stature (Total 1.55%/1.08%: 1.27%/1.01%, 1.58%/0.92%, 1.59%/1.31%), 740/1054 obese students (Total 7.52%/10.51%: 7.50%/11.18%, 7.58%/11.22% 7.54%/9.14%), 560/825 slimming students (Total 5.69%/8.22%: 4.47%/10.73%, 5.88%/7.79%, 7.07%/6.23%).

**Conclusion**: Unlike propotion of the tall stature, that of short stature was about 1.0-1.6% not follow Gauss distribution. According to the school year it goes up, because the students that have problems in physique poor weight gain, obesity, short stature was observed tends to increase, there is a need for early intervention.

## P1-3-1 Monogenic mutations in patients with non-obstructive azoospermia and oligozoospermia

### Shigeru Nakamura^1,2^, Mami Miyado^1^, Kazuki Saito^1,3^, Momori Katsumi^1,4^, Yoshitomo Kobori^5^, Yoko Tanaka^6^, Hiromichi Ishikawa^7^, Atsumi Yoshida^8^, Hiroshi Okada^9^, Hideo Nakai^2^, Tsutomu Ogata^1,9^, Maki Fukami^1^

#### ^1^Department of Molecular Endocrinology, National Research Institute for Child Health and Development; ^2^Department of Pediatric Urology, Jichi Medical University, Children's Medical Center Tochigi; ^3^Department of Comprehensive Reproductive Medicine, Graduate School, Tokyo Medical and Dental University; ^4^Department of NCCHD Child Health and Development, Graduate School, Tokyo Medical and Dental University; ^5^Department of Urology, Dokkyo Medical University Koshigaya Hospital; ^6^Department of Pediatrics, Tokyo Dental College Ichikawa General Hospital; ^7^Tokyo Dental College Ichikawa General Hospital, Reproduction Center; ^8^Department of Pediatrics, Kiba Park Clinic, Reproduction Center; ^9^Hamamatsu University School of Medicine

**Background**: Azoospermia and oligozoospermia are multifactorial conditions that affect more than 1% of adult men. To date, 26 genes have been implicated in the development of non-obstructive azo/oligozoospermia. However, there is no single report of systematic mutation screening of these genes in patient cohorts. Thus, the significance of monogenic mutations in the etiology of azo/oligozoospermia remains unknown.

**Objective**: To clarify the frequency of mutations in known probable pathogenic genes in Japanese patients with non-obstructive azo/ oligozoospermia.

**Materials and Methods**: The study group consisted of 48 Japanese patients with non-obstructive azo/oligozoospermia. Next generation sequencing was performed for coding regions of ART3 , BPY2 , DBY, DNMT3L , FKBP6, FKBPL , KLHL10, MEI1 , MSH4 , PARP2 , PLK4, PRDM9 , PYGO2 , RBMY , SEPTIN12 , SOHLH1 , SPATA17 , STRA8 , SYCP3 , TAF4B , TAF7L , TEX11 , UBR2, USP9Y, ZMYND15 and ZNF230. In this study,

we focused on nucleotide alterations that affect protein sequences or splice sites. Nucleotide changes whose frequency in the general population is more than 1.0% were excluded as common polymorphisms. Functional consequences of missence variants were predicted by two in silico assays.

**Results**: A total of 19 nucleotide alterations were identified in 16 of 48 patients. These variants consisted of 4 hitherto unreported substitutions and 15 rare polymorphisms. Of the 19 nucleotide alterations, 9 were assessed as “possibly/probably damaging” or “deleterious” by in silico assays. In addition, 3 substitutions affected splice sites. Of these, c.346-1G>A in SOHLH1 and c.A511G in TEX11 have been reported as the cause of azoospermia.

**Conclusions**: This study indicates that nucleotide alterations in known probable pathogenic genes account for a certain fraction of cases with non-obstructive azo/oligozoospermia. These results need to be validated in future studies.

## P1-3-2 Recurrent pancreatitis in early infancy due to severe hypertriglyceridemia from a novel mutation of lipoprotein lipase treated with glucose insulin infusion

### Ahila Ayyavoo^1,2^, Paul Hofman^2,4^, Nikhil Phadke^3^, Emma Glamuzina^4^, Palany Raghupathy^1^

#### ^1^Department of Pediatric Endocrinology, G. Kuppuswamy Naidu Memorial Hospital; ^2^University of Auckland, Liggins Institute; ^3^Phadke Hospital, GenepathDx; ^4^Auckland City and Starship Children's Hospital, Adult and Paediatric National Metabolic Service

**Background**: Familial lipoprotein lipase(LPL) deficiency is an extremely rare autosomal recessive disorder of lipoprotein metabolism occurring in 1 per million population. LPL is responsible for the clearance of chylomicrons(large lipoprotein molecules) from circulation. LPL is produced by myocytes and adipocytes, transported to the vascular endothelium for lipolysis of triacylglycerols in chylomicrons. LPL deficiency could happen due to mutations of LPLgenes/apoCII/apoA5/GPIHBP1/LMF1.

Clinical features associated with LPL deficiency include recurrent abdominal pain, poor growth, xanthomatosis, hepatosplenomegaly, lipemic plasma. Severe complications such as recurrent pancreatitis and lipid encephalopathy could happen. Treatment options include dietary fat restriction, use of medium chain triglycerides, fibrates, plasmapheresis and alipogene tiparvovec gene therapy. Acquired hypertriglyceridemia(HTGL) observed in patients with diabetes mellitus improve with insulin, dietary fat restriction, fibrates or niacin. Acquired LPL deficiency is observed during insulin deficiency and could be improved with insulin therapy. Hence, we tried the option of glucose- insulin infusion in a 2 month old boy with recurrent pancreatitis.

**Case Scenario**: Third born 2 month old boy of 3rd degree consanguineous parents had developed fever and vomiting at 10 days of life. Laboratory investigations revealed lipemic serum. He had suffered four episodes of acute pancreatitis by 2 months. Lipid profile is available in Table 1. A homozygous novel mutation was detected in exon 6 of the LPL gene c.904T>C/ p.Cys302Arg which created a missense mutation in the coding region. Other investigations were normal.

**Treatment**: Baby was treated with pancreatitis protocol. Extremely low dose of insulin was infused simultaneously with glucose along-with hourly glucose monitoring. Baby did not have any episodes of pancreatitis after the glucose-insulin intravenous infusion. He is currently on fat-restricted diet with medium chain triglycerides(MCT) and growing normally. Another schedule of IV glucose-insulin infusion helped reduce the triglycerides back to near normalcy at 9 months of age while oral fibrates and sub-cutaneous insulin were ineffective.

**Future**: Subcutaneous insulin needs to be tried on a few more occasions with regular glucose monitoring to enable effective management at home. Glucose-insulin therapy should be replicated in more patients and could be a cheaper option for familial LPL deficiency.

**Table 1 (abstract P1-3-2).**
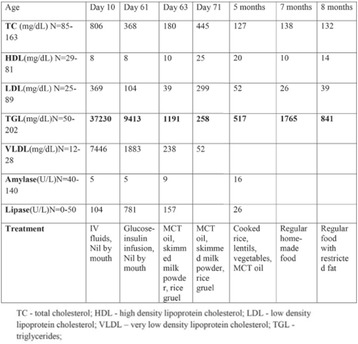
See text for description

## P1-3-3 Glycemic Levels in Normal Newborn-Preliminary Results

### Raphael DR Liberatore Junior, Nathalia Azevedo, Daniela Daltoso, Jose C Simon

#### Division of Pediatric Endocrinology-Pediatric Department, Ribeirao Preto Medical School

The definition of glycemic normal variation in the newborn period is one of the most difficult patterns in neonatology. Different values are proposed based mainly in data from blood glucose samples obtained in the 60’s from the last century.

We proposed to check glycemic interstitial levels in the first 24 hs in a group of normal newborns using a glycemic sensor (Medtronic IproII) implanted subcutaneously in the very first hour after born. All babies were born with more than 38 weeks of gestational age, with no medical problem, and Apgar Score of more than 8 in the first minute. The mothers were not in use of any medication and have no know medical condition. All babies received only breast milk from their own mother and nothing else, including water. The study was approved by ethical committee and the sensor was installed only after write permission from the mother and the father. The results were achieved and showed minimum, maximum, median and standard deviation.

30 normal babies with gestational age between 38 and 41 weeks were studied. The weight ate the born range between 2610 and 3825 grams. The minimum value ranged between 40 and 67 mg/dl, median 47,5 mg/dl. The maximum value ranged between 53 and 99 mg/dl, median 59,5 mg/dl. Normal curve for minute after born will be showed.

The results show that interstitial glycemic levels in normal babies are very close to normal levels in older children. Those results would be very important to define normality of glycaemia.

## P1-3-4 Novel homozygous SCNN1B mutation responsible for fatal systemic Pseudohypoaldosteronism Type 1

### Ahila Ayyavoo^1^, Sowmya Ravikumar^2^, Meenal Agarwal^3^

#### ^1^Department of Pediatric Endocrinology, G. Kuppuswamy Naidu Memorial Hospital; ^2^Department of Neonatology, G. Kuppuswamy Naidu Memorial Hospital; ^3^Phadke Hospital, GenepathDx

**Background**: Generalised pseudohypoaldosteronism type1(PHA1) is an extremely rare salt wasting disorder with hyponatremia, hyperkalemia and metabolic acidosis with elevated levels of aldosterone. Homozygous or compound heterozygous inactivating mutations of the alpha, beta or gamma sub-units of amiloride-sensitive epithelial sodium channel(ENaC) could result in PHA1(OMIM #264350). While severe salt wasting happens from kidneys, colon, sweat, salivary glands in autosomal recessive generalised PHA1(arPHA1), the autosomal dominant PHA1 is a mild disease. The child reported here is the first SCNN1B mutation arPHA1 from India with a novel mutation too.

**Case Scenario**: Term boy born by caesarean section to non-consanguineous parents developed purulent conjunctivitis and acute otitis media on Day 3 of life which was unresponsive to standard management. Baby was admitted on Day7 with normal activity, 16% weight loss and dehydration.

Investigations revealed severe hyperkalemia (10.2mEq/L), hyponatremia (127mEq/L) and acidosis (pH=7.2). Serum creatinine, calcium, phosphorus and creatine kinase were normal. Child was treated with anti-hyperkalemic measures (parenteral calcium gluconate, glucose- insulin infusion, salbutamol nebulisation, bicarbonate, per-rectal potassium binders), parenteral antibiotics, oral hydrocortisone and fludrocortisone with a provisional diagnosis of congenital adrenal hyperplasia. Synacthen stimulation test revealed normal 17OH progesterone, DHEAS and cortisol peak. Serum aldosterone (>150ng/dL; normal=1-16) and plasma renin activity (12.44ng/ml/hour;normal=0.15-2.33) were elevated with persistently elevated serum potassium (K+) and reduced urinary potassium. PHA1 was diagnosed and oral salt was added after stopping hydrocortisone. Electrocardiogram revealed severe hyperkalemia. Since K+ continued to be high, peritoneal dialysis was undertaken after a difficult catheter insertion. K+ dropped to normal levels (4.5mEq/L) and baby was discharged home on oral salt and per-rectal potassium binders. Child returned within a week with severe hyperkalemia and succumbed inspite of all treatment measures.

**Genetics**: Modified NGS panel with custom bioinformatic pipeline run revealed homozygous novel variant in exon 8 of the SCNN1B gene c.1212C>A /p.Tyr404Ter (Y404X) which resulted in premature termination of the amino acid chain. Not previously reported in databases, but pathogenic as per all prediction algorithms.

**Conclusion**: Clinical suspicion, prompt diagnosis and management of electrolyte abnormalities would probably help children with less severe phenotype. Initiation of routine mutation analysis in suspected PHA1 Indian children would help in research into successful treatment measures.

**Consent for publication:** The authors declare that written informed consent was obtained for publication.

**Fig. 1 (abstract P1-3-4).**
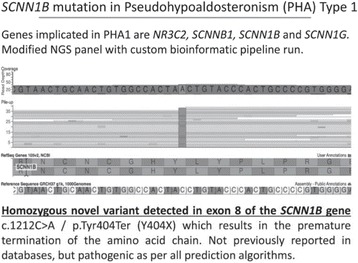
See text for description

## P1-3-5 Effects of tolvaptan, an oral vasopressin antagonist, in the treatment of hyponatremia in a pediatric patient with chronic SIADH

### Sunao Sasaki, Hidetoshi Sato, Yohei Ogawa, Keisuke Nagasaki, Akihiko Saitoh

#### Division of Pediatrics, Niigata University Medical and Dental Hospital

**Background**: Tolvaptan is a vasopressin V2 receptor antagonist for the treatment of euvolemic and hypervolemic hyponatremia. Tolvaptan has demonstrated eficacy in adult patients with syndrome of inappropriate antidiuretic hormone secretion (SIADH); however, there are only a few reports of SIADH in children. Moreover, use of tolvaptan for the treatment of SIADH is not covered by the medical insurance in Japan. SIADH is usually transient and self-limited and can be controlled by short-term water restriction and sodium supplementation. However, the management of chronic SIADH is more difficult, especially in children.

**Patient report**: The patient was a 4-year-old Japanese boy. A delayed language development was noted at a medical check-up for children, at 3 years of age. A brain magnetic resonance imaging (MRI) revealed midbrain tumor and ventricular enlargement. He had undergone an endoscopic third ventriculostomy and tumor biopsy at 3 years and 4 months of age; finally, he was diagnosed as having pilocytic astrocytoma. Since then, his serum sodium concentration fluctuated in the range of 120~130 mEq/L denoting asymptomatic hyponatremia. He had generalized tonic-clonic seizure with fever at the age of 4. We diagnosed chronic SIADH based on his clinical data. In spite of water restriction (800 mL/day) and oral sodium supplementation, his hyponatremia did not improve. Then, on anticonvulsant treatment, he developed afebrile convulsions several times. He was administered oral tolvaptan at 15 mg daily (0.5 mg/kg/day) together with mild water restriction (1000 mL/day). A serum sodium concentration of 130 -135 mEq/L was achieved, and the drug was well tolerated without any side effects. Therefore, the dose of tolvaptan was increased to 22.5 mg daily (0.8 mg/kg/day). After tolvaptan treatment, no seizures were reported.

**Conclusion**: In this patient with chronic SIADH after the surgical treatment for pilocytic astrocytoma, tolvaptan was effective and safe in maintaining the serum sodium concentrations at 130 mEq/L or above. Further clinical studies are necessary for understanding the effect and safety of the long-term use of tolvaptan in children.

**Consent for publication:** The authors declare that written informed consent was obtained for publication.

## P1-3-6 The effect of letrozole on the reproductive function and linear growth in the early pubertal rat

### Juan Lin, Mei Hua Ma

#### Department of Pediatrics, The Third Affiliated Hospital, Sun Yat-Sen University

**Objective**: To provide for the basis of pathological change for the effect of Letrozole on the reproductive function and linear growth in the early puberty rats.

**Method**: At 3 weeks (20-22 days) of age, 14 of 20 SD rats were randomly divided into experimental group (male and female half ), treated with letrozole solution 1 mg/kg/d, 6 of control group (male and female half ), treated with 0.1 ml distilled water, both groups take for 30 days. Every 3 to 4 days weighing, measuring length (nose long anal), the left tibia weight, after 30 days, anesthesia to death and take bilateral ovaries rats (female), testis (male) and left tibial tissue section, using HE staining to observe each group rats ovary (female), testis (males) and the left tibia pathological changes of organizations.

**Results**: 1.The testicular tissue of sperm cells development, mature sperm counts, sertoli cell development and interstitial cells of experimental group had no significant change compared with control group. 2.The experimental group had ovarian cystic structure and atresia follicles significantly increased than control group, appeared the polycystic ovary (PCOS) change, and significantly reduce the granular cell layer. 3.Letrozole had a tendency to increase on the growth plate in the early puberty rats (male, female), which is more obvious in the male rat.

**Conclusion**: 1.30 days use of letrozole in the early puberty male rats had no obvious influence on testicular tissue, its sperm cells development, mature sperm counts, sertoli cell development and interstitial cells had no significant change compared with control group. 2.30 days use of letrozole can cause polycystic ovary change in the early pubertal female rat. 3.30 days ues of letrozole had a tendency to increase on the growth plate in the early puberty rats (male, female), which is more obvious in the male rat.

## P1-3-7 Endocrine dysfunctions in Mandibular Hypoplasia, Deafness, Progeroid Features (MDP) Syndrome: A Case Report and Review of the Literature

### Ting Chen, Linqi Chen, Haiying Wu, Fengyun Wang, Xiuli Chen, Rongrong Xie

#### Children's Hospital of Soochow University, Endocrinology and Metabolism

**Objective**: Mandibular hypoplasia, deafness, and progeroid features and lipodystrophy (MDPL) syndrome is an autosomal dominant disorder characterized mainly by mandibular hypoplasia, sensorineural deafness, progressive lipodystrophy, and hypogonadism in male. This syndrome is caused by heterozygous mutations in POLD1 gene. Short stature was described in half of the reported cases.

**Subjects and methods**: We report here the clinical description of a 10-year-old boy first presented with short stature and hypogonadism. After GH treatment, his fat loss gradually became obvious. We clarified his genetic bases through whole-exome sequencing analysis, and verified the results via sanger sequencing. We also carefully examined the GH/IGF-1 axis, hypothalamic-pituitary-testicular axis, and islet function in the MDPL patient.

**Results**: This article reports the 13th MDPL patient with de novo p. S605del mutation in POLD1 , and the first MDPL patient in Eastern Asia. We found that GH levels after GH stimulation tests were normal, while baseline IGF-1 level was extremely low. The rhGH therapy was effective in accelerating growth velocity, but it would accelerate fat loss and should be contradicted. We also found that the patient had extremely low baseline AMH and testosterone after HCG stimulation. Glucose and insulin levels were normal during OGTT in the patient.

**Conclusions**: Our findings suggested that MDPL syndrome was a possible diagnosis in boys with both short stature and hypogonadism, and rhGH therapy should be started only when this syndrome was excluded. Our study provided better understandings of the endocrine disorders in MDPL syndrome.

## P1-3-8 Mutation analysis of BSCL2 gene in two patients with type 2 congenital generalized lipodystrophy

### Ruimin Chen, Xin Yuan, Ying Zhang

#### Division of Endocrinology and Metabolism, Fuzhou Children's Hospital of Fujian, Teaching Hospital of Fujian Medical University

**Context**: Type 2 congenital generalized lipodystrophy (CGL2, OMIM 269700) is a rare autosomal recessive disease, characterized by the generalized absence of adipose tissue at birth or in early infancy. Mutations in BSCL2 gene have been reported to be responsible for CGL2. Objective To analyze the clinical characteristics of two patients with CGL2, and to investigate the BSCL2 gene in two pedigrees.

**Methods**: Medical history, clinical manifestations, physical examination, laboratory data, and ultrasonography findings were analyzed from two patients with CGL2. Blood samples from both families were obtained for genome DNA extraction. And then the 2742 genes of inherited diseases were amplified by PCR and sequenced.

**Results**: Two patients mainly showed generalized lack of body fat with extreme muscularity from birth, hirsutism, skin pigmentation especially necks and armpits; abnormal faces with empty cheeks, growth acceleration, advanced bone age; hepatomegaly, one of the patients presented with macropenis, renal hypertrophy, and mental retardation; laboratory data showed hypertriglyceridemia, hypercholesterolemia, and low high-density lipoprotein cholesterol. During the follow-up, they appeared bad temper, irritability, with aggressive behavior, abdominal ultrasound of patient 1 prompted fatty liver. They were treated with low-fat, high-carbohydrate diet, blood lipids were controlled to some degree. BSCL2 gene of patient 1 showed a homozygous mutation of c.728dupG, p.Ile262Hisfs * 12, whose parents were carriers of the

heterozygous mutation; BSCL2 gene of patient 2 showed a compound heterozygous mutation: missense mutation c. 713G> A, p.Gly238Asp from paternal; nucleotide repeat c.728dupG, p.Ile262Hisfs * 12 from maternal.

**Conclusion**: We describe two patients with classic clinical manifestations of CGL2 confirmed by genetic sequence analysis. Of them, the c.713G>A, p.Gly238Asp is a novel mutation in BSCL2 gene, previously unreported.

## P1-3-9 X chromosomal deletion due to microhomology-mediated break-induced replication in a boy with Xp22.3 contiguous gene deletion syndrome: Implications for novel genomic defects leading to Kallmann Syndrome

### Koki Nagai^1,2^, Hirohito Shima^1,3^, Miki Kamimura^3^, Junko Kanno^3^, Ikuma Fujiwara^4^, Erina Suzuki^1^, Satoshi Narumi^1^, Akira Ishiguro^2^, Maki Fukami^1^

#### ^1^Department of Molecular Endocrinology, National Research Institute for Child Health and Development; ^2^Department of Postgraduate Education and Training, National Center for Child Health and Development; ^3^Department of Pediatrics, Tohoku University School of Medicine; ^4^Department of Pediatric Endocrinology and Environmental Medicine, Tohoku University Graduate School of Medicine

**Background**: KAL1 is one of the causative genes for Kallmann syndrome (KS), a rare genetic disorder characterized by hypogonadotropic hypogonadism and anosmia. KS is frequently associated with various complications including renal aplasia. Submicroscopic X chromosomal rearrangements involving KAL1 underlie KS either as an isolated anomaly or as a component of contiguous gene deletion syndromes. The genomic basis of these rearrangements remains largely unknown, although non-allelic homologous recombination and non-homologous end- joining have been implicated in a few cases.

**Objective**: To report molecular findings of a patient with KS due to a contiguous gene deletion syndrome.

**Case report**: A 6 month-old boy presented with bilateral cryptorchidism, micropenis, and ichthyosis at his trunk and limbs. His height was 67.4 cm (-0.17 SD), stretched penile length was 2.0 cm (-3.6 SD) and testicular diameters were 8-9 mm bilaterally. His mental development was age-appropriate. GnRH stimulation test at 6 months of age showed normal LH response (< 0.07 to 2.51 mIU/mL) and increased's H response (0.68 to 24.71 mIU/mL). Brain magnetic resonance imaging detected olfactory sulcus and bulbs but no olfactory tracts nor tubercles. Abdominal ultrasonograms and renal scintigrams showed left renal aplasia.

**Molecular analysis**: Array-based comparative genomic hybridization detected a 2.7-Mb deletion at Xp22.3. Breakpoint characterization revealed that the deletion was 2,735,696 bp in physical length and contained NLGN4X, VCX3A, HDHD1, STS, VCX, PNPLA4, VCX2, and VCX3B , in addition to exons 8-14 of KAL1 . The breakpoints shared no long homology but had microhomology of 2-bp. Both breakpoints resided at the margin of Alu repeats. Nucleotide stretches were absent at the fusion junction.

**Discussion**: We characterized a microdeletion in a boy with KS. KS and ichthyosis of the boy can be ascribed to the deletion of KAL1 and STS , respectively. Since the patient lacked NLGN4X and VCX genes involved in the brain function, his mental development should be re-evaluated in future studies. Breakpoint structure indicates that the deletion arose from a break-induced replication mediated by 2-bp microhomology. Notably, the two breakpoints resided at the margin of Alu repeats. Since a high concentration of repetitive elements is known to predispose replication-based errors, the deletion in our patient could have been facilitated by Alu repeats.

**Conclusion**: The results indicate that microhomology-mediated break-induced replication can underlie Xp22.3 contiguous gene deletion syndromes including KS. Alu repeats may be involved in the development of replication errors involving KAL1.

**Consent for publication:** The authors declare that written informed consent was obtained for publication.

## P1-3-10 Androgen measurement in women with polycystic ovary syndrome: Comparison between immunoassays and liquid chromatography-tandem mass spectrometry

### Kazuki Saito^1,2^, Toshiya Matsuzaki^3^, Mami Miyado^1^, Momori Katsumi^1^, Shigeru Nakamura^1,4^, Minoru Irahara^3^, Tsutomu Ogata^5^, Maki Fukami^1^

#### ^1^Department of Molecular Endocrinology, National Center for Child Health and Development; ^2^Department of Comprehensive Reproductive Medicine, Graduate school, Tokyo Medical and Dental University; ^3^Department of Obstetrics and Gynecology, Graduate School of Biomedical Scientces, Tokushima University; ^4^Department of Pediatrics, Jichi Medical University; ^5^Department of Pediatrics, Hamamatsu University School of Medicine

**Context**: Patients with polycystic ovary syndrome (PCOS) frequently develop hyperandrogenemia. So far, blood androgens in PCOS patients have been measured primarily by immunoassays. However, accumulating evidence suggests that liquid chromatography-tandem mass spectrometry (LC-MS/MS) is more accurate in steroid qualification than immunoassays.

**Objective**: To compare the results of immunoassays and LC-MS/MS in androgen measurement in PCOS patients and eumenorrheic women. Materials and Methods: Blood samples were obtained from 31 PCOS patients and 28 eumenorrheic women. Serum levels of testosterone and androstenedione were analyzed by chemiluminescent enzyme immunoassay (CLEIA) and radioimmunoassay (RIA) respectively, and by LC- MS/MS. The results of the patients were compared to normal ranges (the mean ± 2.0 standard deviation of the control individuals).

**Results**: Testosterone values of each individual were closely matched between CLEIA and LC-MS/MS (correlation coefficient in patients, 0.852; in controls, 0.898). The correlation coefficients were comparable between the patient and control groups (p = 0.470). Androstenedione values in each individual determined by LC-MS/MS and RIA were closely matched in controls (correlation coefficient, 0.908), but less correlated in patients (correlation coefficient, 0.717; p = 0.026). Testosterone measurement by CLEIA yielded false positive and negative results in one and three cases respectively, and androstenedione measurement by RIA provided false positive and negative results in six and four cases respectively.

**Discussion**: The results are consistent with the previously proposed notion that the results of immunoassays are affected by cross-reacting steroids. The effects of cross- reaction seem to be more prominent in androstenedione measurement than in testosterone measurement, and in PCOS patients than in eumenorrheic women. Notably, androstenedione measurement by RIA yielded false positive (indicated by gray boxes in the figure)/negative (indicated by black boxes in the figure) results in 10 of 31 PCOS patients. Since increased blood levels of testosterone and androstenedione is associated with the risk of metabolic diseases in PCOS patients, precise androgen measurement using LC-MS/MS would serve to improve the long-term prognosis of these patients.

**Conclusions**: In PCOS pat ients, bloo d levels of androgens, particularly those of androstenedione, should be measured by LC-MS/MS.

**Fig. 1 (abstract P1-3-10).**
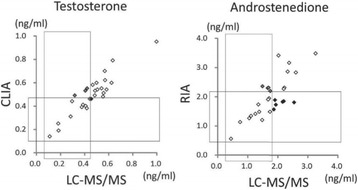
See text for description

## P1-3-11 Clinical and Genetic Study of One Case with Complex Glycerol Kinase Deficiency

### Yu Yan, Xingxing Zhang, Donghai Liu, Xingfang Li

#### Department of Pediatrics, The Second Xiangya Hospital of Central South Universityg

**Background**: Complex glycerol kinase deficiency (CGKD), also called Xp21 contiguous gene deletion syndrome, is a rare X linked recessive disorder which may including congenital adrenal cortical dysfunction (AHC), glycerol kinase deficiency (GKD), Duchenne muscular dystrophy (DMD), chronic granulomatous disease (CGD), retinitis pigmentosa (RP), Xpter - Alan islands eye disease (AIED), etc.

**Objective**: To analyze the clinical and genetic features of CGKD, to provide a more accurate diagnosis in children, while improving clinician awareness of the disease.

Methods: Summarize the clinical characters of a patient diagnosed with CGKD. And analyzing his gene mutation by using EDTA anticoagulant for the specimens with single-gene disorders whole exon sequencing, and verifying the result by PCR electrophoresis.

**Results**: A 52-day-old boy characterized with progressive deepening skin pigmentation and slow weight gain. Auxiliary examinations showed electrolyte disorders (hyponatremia, hyperkalemia), low serum cortisol, high adrenocorticotropic hormone (ACTH), damaged liver function, significantly increased creatine kinase, elevated blood triglycerides, normal blood sugar and cholesterol. Results of single gene disorders whole exon sequencing indicated the child has NR0B1 Exon1-2, IL1RAPL1 Exon2-11, TAB3 Exon5-11, GK Exon1-21, DMD Exon48-79 gene deletions, which were homozygous mutation.

**Conclusion**: The clinical manifestations of the patient and results from the single gene disorders whole exon sequencing once again proved CGKD relate to the X chromosome gene deletion near Xp21. It has been revealed that NR0B1, IL1RAPL1, GK, DMD gene are respectively related to adrenal cortical dysfunction, X-linked mental retardation 21, glycerol kinase deficiency, Duchenne muscular dystrophy. However, the association between TAB3 gene deletion and CGKD has not reported in the literature, which may be a new discovery of the CGKD-related mutations, and further broaden the CGKD gene mutation spectrum.

**Consent for publication:** The authors declare that written informed consent was obtained for publication.

## P1-3-12 Familial Neurohypophyseal Diabetes Insipidus Due to a Mutation in the Arginine Vasopressin-Neurophysin II Gene

### Chan Jong Kim, Eun Mi Yang

#### Department of Pediatrics, Chonnam Natl. Univ. Hospital

Autosomal dominant familial neurohypophyseal diabetes insipidus (adFNDI) is an inherited disorder of free water conservation characterized by childhood onset polyuria, polydipsia and dehydration caused by arginine vasopressin deficiency. A variety of disease-causing mutations of the arginine vasopressin neurophysin II gene (AVP) on chromosome 20p13 have been described. A four year old body with polyuria and polydipsia was shown to have central diabetes insipidus using the water deprivation test and a vasopressin challenge test. His family history was consistent with autosomal transmission of the polyuric syndrome, with affected family members in three generations, including several females. Direct sequencing of the AVP gene showed a heterozygous missense mutation in exon 2 of the AVP gene (Cys98Gly). This mutation was predicted to yield an abnormal AVP precursor in its neurophysin II moiety and the function of neurophysin as a carrier protein for AVP would be impaired. The proband's mother and additional three family members have the same mutation. Presence of this mutation suggests that the portion of the neurophysin peptide encoded by this sequence is important for the appropriate expression of vasopressin. We present a mutation of AVP in a Korean family suffering with adFNDI over three generations.

## P1-3-13 Efficacy of recombinant human growth hormone therapy for children with different causes of short stature

### Feng Xiong, Xueshuang Zhang

#### Department of Endocrinology, Chongqing Medical University Affiliated Children's Hospital

**Introduction**: To investigate differences in clinical efficacy and safety of using recombinant human growth hormone (rhGH) in Chinese children with different causes of short stature.

**Methods**: From August 2005 to October 2015, 73 children (43 males and 30 females, aged 9.5 ± 3.3 years) with growth hormone deficiency (GHD), 56 children i22 males and 34 females, aged 8.9 ± 2.7 years ) with Idiopathic Short Stature (ISS), 50 children(aged 9.9 ± 2.9 years) with Turner syndrome (TS) and 21 children(8 males and 13 females, aged 9.9 ± 2.9 years) with small for gestational age (SGA) were enrolled from the endocrine clinic of Chongqing Medical University Affiliated Children's Hospital. Height(Ht), growth velocity(GV), height standard deviation score (HtSDS), body mass index(BMI), body mass index standard deviation score(BMI SDS), bone age(BA), final adult height(FAH) and side effects were observed during 2-4 years of rhGH treatment.

**Results**: 1, The GVs of the enrolled children were significantly increased in the first year treatment, respectively (11.23 ± 2.63) cm/year, (9.91 ± 1.67) cm/year, (8.45 ± 1.83) cm/year and (9.78 ± 1.72) cm/year, then decreased progressively during each succeeding treatment year. 2, The HtSDSs of the enrolled children were improved year by year, and the change of HtSDS ( Δ HtSDS) in children with GHD(2.12 ± 1.12) was greater as compared to children with ISS(1.51 ± 0.82), TS(1.23 ± 0.73), and SGA(2.07 ± 1.04). 3, The FAH in some of the enrolled children was documentedand found to be close to their target height. 4, After 2-4 years of rhGH treatment, the BMI and BMI SDS of the enrolled children were slowly increased. 5, During 2 `4 years of rhGH treatment, the bone age and puberty of the enrolled children didn’t advance, and adverse reactions were rare.

**Conclusion**: There was a great effect of rhGH treatment on growth in children with GHD, ISS, TS and SGA, However Cthe efficacy of rhGH differed greatly in children with different causes of short stature, and children with GHD had the most obvious effect, followed by children with SGA, ISS and TS.

## P1-3-14 Exercise capacity and left ventricular function in adolescents born post-term: an MRI study

### Silmara Gusso, Mrinal Murali, Paul L Hofman, Jose Derraik, Janene Biggs

#### University of Auckland, Liggins Institute

**Background**: Children born post-term ( ≥ 42 weeks gestation) may be at risk of adverse health outcomes in adulthood. Recent studies showed that metabolic abnormalities, including reduced insulin sensitivity, dyslipidaemia, and alterations in blood pressure are already present in childhood. These abnormalities are similar to those reported in small-for-gestational-age and preterm children and adolescents, who also have reductions on exercise capacity and cardiovascular alterations during adolescence. It is not known whether adolescents born post-term have similar cardiovascular impairments. Therefore, we investigated aerobic capacity and cardiovascular function in adolescents born post-term in comparison to peers born at term (37-41 weeks of gestation).

**Methods**: 51 participants were recruited: 25 born post-term and 26 controls aged 14.3 years (range 12–20 years). Body composition (DXA), exercise capacity (VO2max), 24-hour ambulatory blood pressure monitoring, and fasting blood tests were performed in all participants. Left ventricular structure and function were assessed by MRI scans at rest and during submaximal exercise, using a compatible cycle ergometer.

**Results**: There no observed differences in weight, height, body composition, blood parameters, or 24-h blood pressure between groups. Exercise capacity was lower in the post-term vs control group, with more marked differences seen when NZ Europeans were examined separately (46.3 vs 51.4 ml/kgffm/min; p=0.041). Diastolic blood pressure was higher at peak exercise in those born post-term (62 vs 57 mmHg; p=0.03). There were no differences in left ventricular volumes and mass between groups at rest and during submaximal exercise.

**Conclusion**: Adolescents born post-term have reduced exercise capacity when compared to match term-born controls. However, measurements of central cardiac function showed no differences between the two groups. Hence, we hypothesize that the reduction in exercise capacity in adolescents born post-term could be associated with changes in the peripheral vascular system.

## P1-3-15 Efficacy of administration of desmopressin orally disintegrating tablets via an enteral feeding tube to two children with disabilities and central diabetes insipidus

### Mari Hasegawa, Keiji Nogami, Midori Shima

#### Department of Pediatrics, Nara Medical University

## P1-3-16 Gene analysis and literature review of Autosomal dominant hypocalcemia type 1 -case report

### Linqi Chen, Hui Sun, Haiying Wu, Fengyun Wang, Ting Chen, Xiuli Chen, Rongrong Xie

#### Division of Endocrinology and Metabolism / Department of Pediatrics, The Children's Hospital of Soochow University

**Objective**: The purpose of this study was to investigate the clinical and genetic characteristics of autosomal dominant hypocalcemia type 1.

**Method**: Targeted sequencing was used on a children who was accurately diagnosed as autosomal dominant hypocalcemia type 1 in Children’ s Hospital Affiliated to Soochow University to analyze the major clinical manifestations of the disease. An analysis of the CaSR genes was made on the patient.

**Result**: The patient was a girl, 1 months 11 days old, and had recurrent seizures for more than three weeks, aggravating for three days. Laboratory work-up revealed hypocalcemia, hypomagnesemia, hyperphosphatemia, suppressed PTH, increased urinary calcium-to-creatinine. The child was clinically highly suspected of autosomal dominant hypocalcemia type 1. Targeted sequencing showed a mutation in exon 3 Gene, c.392G>A, p.cys131Tyr, and both parents did not harbor the child’s mutation, indicating that her mutation had arisen de novo.

**Conclusion**: Autosomal dominant hypocalcemia type 1 is caused by gain-of-function mutations in the CaSR gene, manifests familial or sporadic hypercalciuric hypocalcemia. One pathogenic mutations (c.392G>A, p.cys131Tyr) in CaSR gene was found.

**Consent for publication:** The authors declare that written informed consent was obtained for publication.

## P1-3-17 Diazoxide-unresponsive congenital hyperinsulinism in a Tertiary Hospital in North-Eastern of Thailand: case report

### Jaturat Petchkul

#### Division of Endocrinology and Metabolism / Department of Pediatrics, Sanpasitthiprasong Hospital

**Introduction**: Congenital hyperinsulinism (CHI) is the most common cause of intractable hypoglycemia during newborn and infantile period which resulting from inappropriate insulin secretion by pancreatic β -cell . The incidence is approximately 1: 50,000 live births. Mutation in ABCC8 and KCNJ11, encoding subunit of ATP- sensitive K+ (KATP) channel in pancreatic β cell, are found in more than 50% of patients with CHI. These 2 mutation causing severe CHI and unresponsiveness to K ATP channel agonist, diazoxide. Here, we report 2 cases of diazoxide-unresponsive CHI due to ABCC8 mutation.

**Case1**: A 36 weeks male infant, BW 3,490 g was born from non-consanguineous parents. He presented with hypoglycemia at birth and was given IV concentrated glucose up to 10 mg/kg/min. Investigations revealed blood sugar 1.16 mmol/L, insulin 7.9 μIU/L, cortisol 3.5 μg/dL and negative serum ketone. ACTH stimulation was normal. He was diagnosed hyperinsulinemic hypoglycemia of infancy. After treated with diazoxide 20 mg/kg/day, hypoglycemia still persisted. Nifedipine use for decreased insulin secretion was given at starting dose of 0.3 mg/ kg/day then titrated up to 1 mg/kg/day, but frequently hypoglycemia persisted. Genetic study showed ABCC8 missense mutations. He was referred for near total (about 98 %) pancreatectomy. After pancreatectomy, diazoxide and nifedipine were discontinue and rate of iv glucose gradually decreased until 4 mg/kg/min. Few days later, his blood sugar was < 2.8 mmol/L again then diazoxide was restarted. At present, the patient is 1 year and 7 months old. The treatment are diazoxide 17 mg/kg/day, octreotide 12 μg/kg/day and corn starch. His weight and height below 3rd percentile and delay language.

**Case2**: A 6 weeks old female infant presented with 4 episodes of hypoglycemia seizure. Her blood sugar was 1.61 mmol/L while insulin level was 6.9 μIU/L. Other hormone test were normal. She was diagnosed hyperinsulinemic hypoglycemia of infancy. Diazoxide was given up to 17mg/kg/day but she still frequently hypoglycemia. Genetic study showed ABCC8 nonsense mutations. The patient was referred for 18-F DOPA PET scan and focal pancreatectomy was done. After pancreatectomy, she still needed diazoxide 4.5 mg/kg/day for normalize blood sugar.

**Conclusion**: although rare, CHI should be evaluated in newborn and infant who had persistent hypoglycemia and need glucose concentration > 8 mg/kg/min. Genetic study should be done as a guide for prognosis and treatment strategy. Hypoglycemia must be promptly treated with high concentration glucose, diazoxide, octreotide to prevent brain damage. When medical are failure, surgical treatment is required.

**Consent for publication:** The authors declare that written informed consent was obtained for publication.

## P1-3-18 Clinical and genetic findings in Chinese patients with methylmalonic aciduria: from heterogeneity to clinical strategy

### Zheng Zhangqian, Luo Feihong, Lu Wei

#### Children's Hospital of Fudan University, Endocrinology and IEM

**Objective**: To analyze the heterogeneity of in frequent symptoms among Chinese children with methylmalonic aciduria (MMA) originated from a mixed geneti background.

**Subjects and Methods**: We investigated 26 Chinese patients (17 males and 9 females) diagnosed by elevated urinary MMA, elevated serum C3, C3/C2 ratio and decreased serum free carnitine. Genetic diagnoses of these children were made by sequencing of MUT , MMAA , MMAB , MMACHC , MMADHC , LMBRD1 , MCEE , SUCLG1 , SUCLG2 and ABCD4 gene. The clinical and biochemical features were analyzed.

**Results**: We identified a considerable variation between clinical manifestations of these children. Symptoms of MMA varied from mild vomiting to severe encephalopathy and the age onset from 3 days to 13 years. Less frequent symptoms (<10%) such as hydrocephalus, isolated pulmonary hypertension, peripheral neuropathy and diabetic ketoacidosis were found as the first signs in MMA patients. Mutatios in MUT , MMACHC and MMADHC gene were identified in 22 patients. Among these mutations, two novel missense mutations in MUT gene (c.1540C>A and c.505G>T) were identified. In 2 patients with isolated pulmonary hypertension, we found the same mutation sites and so that in 2 patients with hydrocephalus (Table 3).

**Conclusions**: The heterogeneous clinical manifestation identified among our Chinese patients with MMA indicated that the genetic profiles of MMA patients vary from ethnics and there were phenotype-genotype correlations according to our genetic sequencing results.

## P1-3-19 New mutation in pseudohypoparathyroidism type Ia

### Zerrin Orbak

#### Department of Pediatric Endocrinolpgy, Ataturk University Medical Faculty

The GNAS gene encodes the alpha-subunit of the stimulatory G proteins, which play a crucial role in intracellular signal transduction of peptide and neurotransmitter receptors. Heterozygous inactivating maternally inherited mutations of GNAS lead to a phenotype in which Albright hereditary osteodystrophy is associated with pseudohypoparathyroidism type Ia.

The GNAS gene of a 5-year-old boy with brachydactily, mental retardation, obesity, pseudohypoparathyroidism and congenital hypothyroidism was investigated. We found a heterozygous mutation of exon 6 (p.Gln171*(c.511C>T)).

This report demonstrates the new mutation at the GNAS gene in a patient who was thought to have pseudohypoparathyroidism type Ia.

## P1-4-1 Somapacitan, a once-weekly reversible albumin-binding growth hormone (GH) derivative, is well tolerated and convenient in adults with GH deficiency (AGHD): results from a 26-week randomised, controlled phase 3 trial

### Michael Højby Rasmussen^3^, Gudmundur Johannsson^1^, Ulla Feldt-Rasmussen^2^, Ida Holme Håkonsson^3^, Henrik Biering^4^, Patrice Rodien^5^, Shigeyuki Tahara^6^, Toogood Andrew^7^

#### ^1^Department of Internal Medicine, University of Gothenburg; ^2^Rigshospitalet, Copenhagen University Hospital, Medical Endocrinology; ^3^Novo Nordisk A/S, Global Development; ^4^MediCover Berlin-Mitte MVZ; ^5^CHU Angers Centre Hospitalier Universitaire, Département d'endocrinologie-diabétologie-nutrition; ^6^Department of Neurosurgery, Nippon Medical School; ^7^Department of Endocrinology, Queen Elizabeth Hospitals Birmingham

GH replacement as daily s.c. injections for patients with AGHD can be cumbersome. Somapacitan (Novo Nordisk), a once-weekly reversible albumin-binding GH derivative, has been shown in short-term trials to be well tolerated in healthy adults and in patients with AGHD. This trial was a multinational, multicentre, randomised (2:1), open-label, active-controlled trial (NCT02382939; REAL 2) investigating the safety, tolerability and treatment satisfaction of once-weekly somapacitan versus once-daily GH (somatropin) in patients with AGHD.

Ninety-two patients (diagnosed with AGHD, previously treated with somatropin for ≥ 6 months, male or female, aged 18–79 years) were randomised to once-weekly somapacitan or once-daily somatropin. During the first 8 weeks, somapacitan and somatropin doses were titrated according to serum insulin-like growth factor-I (IGF-I) standard deviation score (SDS) levels in order to achieve IGF-I levels within the normal range and preferably between 0 and 2 SDS; a fixed dose was received for the remaining 18-week period. Treatment satisfaction was assessed using the Treatment Satisfaction Questionnaire for Medication-9 (TSQM-9), with an increase in scores signifying an increase in treatment satisfaction. A similar pattern and rate of adverse events (AEs) and serious AEs was observed with the two treatments. The most frequently occurring AEs for somapacitan and somatropin were nasopharyngitis, headache and fatigue. Mild and transient injection site reactions were observed only in the somapacitan group. After dose titration, the IGF-I levels were maintained in both treatment groups. No anti-somapacitan or anti-GH antibodies were detected. Using a mixed model for repeated measurements with treatment, GHD onset type, gender, and region as factors and baseline as a covariate, all nested within week as a factor, a statistically significant difference in change from baseline to end of treatment period in convenience score was observed with once-weekly somapacitan being more convenient than once-daily somatropin. Using the same analysis model, no statistically significant difference in change from baseline to end of treatment period between once-weekly somapacitan and once-daily somatropin was shown for the effectiveness and global satisfactions scores.

In conclusion, somapacitan was well tolerated and no safety issues were identified. These data indicate that somapacitan may serve as a well-tolerated, once-weekly treatment for AGHD and may be more convenient for patients than once-daily treatment.

## P1-4-2 Incidences of Type 2 Diabetes Mellitus and Neoplasm in Growth Hormone Treated Children in Japan

### Susumu Yokoya^1^, Tomonobu Hasegawa^2^, Keiichi Ozono^3^, Hiroyuki Tanaka^4^, Susumu Kanzaki^5^, Toshiaki Tanaka^6^, Kazuo Chihara^7^, Nan Jia^8^, Christopher J Child^9^, Noriyuki Iwamoto^10^, Yoshiki Seino^11^

#### ^1^Department of Medical Subspecialties, National Center for Child Health and Development; ^2^Department of Pediatrics, Keio University School of Medicine; ^3^Department of Pediatrics, Osaka University Graduate School of Medicine / Faculty of Medicine; ^4^Department of Pediatrics, Okayama Saiseikai General Hospital; ^5^Department of Pediatrics, Tottori University Faculty of Medicine; ^6^Tanaka Growth Clinic; ^7^Department of Internal Medicine, Hyogo Prefectural Kakogawa Medical Center; ^8^Eli Lilly and Company, USA, Lilly Research Laboratiries; ^9^Eli Lilly and Company, UK, Lilly Research Laboratiries; ^10^Eli Lilly Japan K.K., Medical Science; ^11^JCHO Osaka Hospital

**Background**: The safety of growth hormone (GH) treatment in children with short stature has generally been demonstrated, with no significant adverse events reported in association with GH treatment after more than 20 years of postmarketing use. The safety record of GH remains good, which is supported by evidence from the follow-up of tens of thousands of children. However, the potential risk of GH therapy leading to diabetes mellitus and certain tumoral diseases remains a concern because GH acts as an insulin counterregulatory hormone and promotes cell proliferation. Accordingly, there is a high demand for results of large scale observational studies.

**Objective**: The primary objective of the Genetics and Neuroendocrinology of Short Stature International Study (GeNeSIS) was to assess the safety and efficacy of HUMATROPE®, a GH preparation manufactured by Eli Lilly and Company, in the treatment of pediatric patients with short stature (NCT01088412). We report our findings in the GH-treated Japanese pediatric population focusing on the incidence of type 2 diabetes mellitus and occurrence of neoplasms.

**Methods and Results**: A total of 2,356 patients were registered for enrollment in the study between 2000 and 2013. Of these 2,308 had ≥1 follow-up visit allowing for assessment of safety. During a mean study observation period of 3.3 years, type 2 diabetes mellitus occurred in 3 patients (0.13%) and SPIDDM related to MELAS in 1 patient (0.04%). Neoplasms were reported in 12 patients (0.52%), including 1 patient with de novo germinoma, and 5 with craniopharyngiomas (4 were recurrences and 1 was newly assessed by the investigator), the remainder were benign, typically dermatological, neoplasms.

**Discussion**: In the present study, pre-existing craniopharyngioma was found in 25 of the 57 patients with growth hormone deficiency (GHD) due to intracranial tumors, recurring in 4 of the 25 patients (16%). The global KIGS data published in the Acta Paediatrica (2006) and Lancet (2000) revealed a recurrence-free survival rate of 63% for craniopharyngioma patients and an incidence rate of 0.12% for diabetes mellitus during a GH treatment, neither of which was different from our results.

**Conclusion**: The incidence of diabetes mellitus determined in the present study did not differ from previous reports of GH-treated pediatric patients with short stature, and there was no apparent increase in the risk of new neoplastic lesions or malignant tumors.

## P1-4-3 Sequence variations in genes of the GH-IGF-1 axis in children with idiopathic short stature

### Atsushi Hattori^1^, Yuko Katoh-Fukui^1^, Keiko Matsubara^1^, Maki Igarashi^1^, Erina Suzuki^1^, Akie Nakamura^1^, Hiroyuki Tanaka^2^, Keisuke Nagasaki^3^, Koji Muroya^4^, Reiko Horikawa^5^, Shinobu Ida^6^, Toshiaki Tanaka^7^, Tsutomu Kamimaki^8^, Tsutomu Ogata^9^, Maki Fukami^1^

#### ^1^Department of Molecular Endocrinology, National Research Institute for Child Health and Development; ^2^Department of Pediatrics, Okayama Saiseikai General Hospital; ^3^Department of Homeostatic Regulation and Development, Niigata University Graduate School of Medical and Dental Sciences, Division of Pediatrics; ^4^Department of Endocrinology and Metabolism, Kanagawa Children's Medical Center; ^5^Division of Endocrinology and Metabolism, National Center for Child Health and Development; ^6^Department of Gastroenterology and Endocrinology, Osaka Medical Center and Research institute for Maternal and Child Health; ^7^Department of Pediatrics, Tanaka Growth Clinic; ^8^Shizuoka City Shimizu Hospital; ^9^Department of Pediatrics, Hamamatsu University School of Medicine

**Background**: Short stature patients with mutations in known genes of the GH-IGF-1 axis are usually diagnosed by specific physical or endocrinological findings. Although recent studies have identified genetic defects in GHR and IGF-1 in a few patients clinically diagnosed with idiopathic short stature (ISS), it remains unknown whether mutations in genes of the GH-IGF-1 axis are common in children with ISS. Objective: To elucidate the frequency of sequence variations in genes of the GH-IGF-1 axis in children clinically diagnosed with ISS.

**Subjects and Methods**: The study group consisted of 96 Japanese children (63 males and 33 females) with height below − 2.0 stadard deviation score based on the Japanese growth reference. Patients with syndromic short stature, apparent GH deficiency, thyroid disorders, and other chronic diseases were excluded from this study. We also excluded individuals with SHOX haploinsufficiency and those born small for gestational age. We analyzed the coding exons of GH1 , GHRHR , GHSR , GHR , IGF1 , and IGF1R by next generation sequencing. In this study, we focused on nonsynonymous variants and nucleotide changes at splice sites. We excluded common variants whose allele frequency in the general population was more than 1.0%. The functional consequences of nucleotide alterations were assessed by in silico analyses.

**Results**: Seven nucleotide changes were identified in 10 children. Of these, three were hitherto unreported, while remaining four have been submitted to the Single Nucleotide Polymorphism Database. Four missense substitutions (one in GHR , two in GHRHR , and one in IGF1R ) of four individuals were predicted as “damaging”.

**Discussion**: Possibly damaging variations affecting the GH-IGF-1 axis were identified in four of 96 children with ISS. Clinical significance of these variations needs to be validated in future studies. The results of this study indicate that sequence variations in genes of the GH-IGF-1 axis play a minor role in the etiology of ISS.

## P1-4-4 Mouse Polycomb Cbx2 Functions in Skeletal Lineage

### Yuko Katoh-Fukui^1^, Miyuki Shindo^2^, Rie Natsume^3^, Kenji Sakimura^3^, Hideki Tsumura^2^, Maki Fukami^1^

#### ^1^National Center for Child Health and Development, Molecular Endocrinology; ^2^National Center for Child Health and Development, Laboratory Animal Facility; ^3^Niigata University, Department of Cellular Neurobiology, Brain Research Institute

**Background/Aims**: Normal growth in childhood depends on multiple hormones, secreted molecules, and intracellular factors that regulate the activity of growth plate and bones. Recently, in vivo functional analyses of genes in mice revealed numerous epigenetic regulators controlling chondrogenesis and osteogenesis. For instance, phylogenically conserved Polycomb group (PcG) proteins play indispensable epigenetic role in development and growth. So far, we generated mice nullizygous for Cbx2, one of the members of Polycomb repressive complex 1 (PRC1), and reported the bone growth defects in ESPE 2013 and JSPE 2014. Yet, it remains unclear whether Cbx2 in bones contributes to their own growth, because of the ubiquitous expression of Cbx2 in the developing embryo. To elucidate functions of Cbx2 in the skeletal lineage, we generated conditional Cbx2 knockout mouse (Cbx2 mscKO), in which Cbx2 was deleted in skeletal lineage progenitor.

**Results**: Ossification delay was observed in the prenatal Cbx2 mscKO (Cbx2flox/flox:Prrx1-Cre+ ). For Cbx2 mscKO generation, a cross breading of Prrx1-Cre skeletal lineage specific deleter line (Jackson Laboratory, USA) and Cbx2flox/flox line was carried out. We generated Cbx2flox/flo line by intercrossing Cbx2tm1a/Wtsi (MRC, the Wellcome Trust Sanger Institute, UK) and Actb-flp knock-in mice (Established by Dr. Sakimura, Niigata University, Japan). All mouse line utilized in this study was established and maintained in C57Bl/6 genetic background.

**Discussion**: In the skeletal lineage, one of the members of PRC1 component, Cbx2, has an essential role for the bone growth. PcG proteins configure two major multimeric protein complexes; PRC1 and PRC2. PRC1 and PRC2 are tethering around the large number of developmental key regulator genes and cooperate to regulate their expression. Recently, PRC2 member Ezh2 was deleted in the skeletal lineage using Prrx1- Cre+ (Ezh2 mscKO) (Dudakovic et al., 2015). In the report, defects in the bone formation in newborn mscEzh2 KO were demonstrated. Similar functions of Ezh2 and Cbx2 in bone formation are consistent with the proposed model of cooperative functions of PRC1 and PRC2.

## P1-4-5 Practical growth charts for Japanese children with Noonan syndrome

### Tsuyoshi Isojima^1,2^, Satoru Sakazume^3^, Tomonobu Hasegawa^4^, Tsutomu Ogata^5^, Toshio Nakanishi^6^, Toshiro Nagai^2^, Susumu Yokoya^7^

#### ^1^Department of Pediatrics, Teikyo University School of Medicine; ^2^Department of Pediatrics, Graduate School of Medicine, The University of Tokyo; ^3^Department of Pediatrics, Dokkyo Medical University Koshigaya Hospital, Koshigaya; ^4^Department of Pediatrics, Keio University School of Medicine; ^5^Department of Pediatrics, Hamamatsu University School of Medicine; ^6^Department of Pediatric Cardiology, The Heart Institute, Tokyo Women's Medical University; ^7^Department of Medical Subspecialties, National Center for Child Health and Development

**Background**: Noonan syndrome (NS) is a clinically and genetically heterogeneous syndrome characterized by distinctive facial features, short stature, congenital heart diseases, and other comorbidities. NS-specific growth charts are essential for NS care, but currently no such charts are available for Asian populations. Therefore, we have recently established growth references of height, weight, and body mass index (BMI) for Japanese individuals with Noonan syndrome. The purpose of this study is to draw practical growth charts of height, weight, and BMI, and newly construct those of weight for height (WFH). In addition, we compare these charts to those of Turner syndrome (TS) or normal population.

**Methods**: The population for this study was the same as the previous study. Briefly, we conducted a nationwide survey by collaborating with three academic societies in Japan. We obtained the data of 356 clinically diagnosed NS subjects from 20 hospitals. The LMS method was used for establishing growth charts.

**Results**: A total of 308 subjects (boy: 159, girl: 149) were analyzed after excluding 48 subjects because of missing auxological data, presence of complications affecting growth, and extreme longitudinal growth aberrations which lay more or less than three standard deviation scores (SDS) from the mean. The practical charts were constructed with 3,249 mixed longitudinal and cross-sectional measurements. According to the charts, the adult height is 157.3 ± 7.4 cm for boys (-2.3 ± 1.3 SDS) and 146.8 ± 6.9 cm for girls (-2.1 ± 1.3 SDS), respectively, when it is defined as the mean height at the age of 20 years. Growth pattern of height in girls with NS is similar to that of TS below age five but there are gradual increase and pubertal spurt resulting in 1.2SD taller adult height than TS. Growth pattern of BMI in NS is similar to that of normal populations and TS girls until approximately five years of age, but gradual increase after BMI rebound age is milder. As for the WFH reference, WFH in NS is almost the same as normal populations. In contrast, WFH in NS girls is almost the same as those in TS girls below the height of 100 cm, but above 120 cm there is a lack of more rapid increase of the WFH evident in TS girls.

**Conclusion**: Practical growth charts for Japanese individuals with NS were established. These charts are expected to be adopted and used in various clinical settings.

## P1-4-6 Infantile growth pattern of girls with Turner syndrome

### Satsuki Nishigaki^1^, Takashi Hamazaki^1^, Akitoshi Tsuruhara^2^, Toshiko Yoshida^3^, Takuji Imamura^4^, Hiroshi Inada^5^, Keinosuke Fujita^1^, Haruo Shintaku^1^

#### ^1^Department of Pediatrics, Osaka City University Graduate School of Medicine; ^2^Department of Pediatrics, Izumi municipal hospital; ^3^Department of Pediatrics, Osaka Saiseikai Senri Hospital; ^4^Department of Pediatrics, PL general hospital; ^5^Osaka City Public Health Office

**Background**: Short stature is the key to diagnosis for Turner syndrome (TS) in childhood and adolescence. We often encountered some girls with TS already had growth retardation from their infantile period but growth patterns during this period have not been well described. Objective: To characterize growth patterns for TS during their infantile period and relations to clinical diagnosis.

**Subjects and methods**: A retrospective review of medical records was performed on Japanese girls with TS in our hospital who were not on GH therapy until 3 years old.

**Results**: Growth records of twenty four girls with TS were subjected to analysis. Mean changes in height SDS from birth to 1 month, 3months, 18 months and 3 years were +0.4, 0.0, -0.9 and -1.1 respectively, indicating catch-up growth was observed only during the first few months and the growth was retarded thereafter. No correlation between karyotypes and growth patterns was observed. Next we divided into two groups: A) height SDS decreased below -2SD by 3 years of age (n=11), B) height SDS remained above -2SD at age 3 (n=13). Height at birth was significantly lower in group A (-1.58 ± 1.54SD) than B (-0.36 ± 0.79SD). There was no significant difference between two groups regarding with the growth patterns from birth to 3 years old. Median age at diagnosis was 4 years old in group A but it was 9 years old in group B.

**Discussion and Conclusion**: In Japan, the 3 year checkup is the last regular checkup before the preschool checkup and short stature is important health problem which prompts physicians to refer children to pediatric endocrinologists. Therefore we observed that girls who didn’t have apparent short stature at the checkup tended to be diagnosed with TS later. Early diagnosis is anticipated before problems at school occur. Since growth retardation in girls with TS was observed as early as 3 months old, it is important to pay attention to the change in infantile growth rate as well as height-for-age.

## P1-4-7 Safety and efficacy of daily rhGH treatment up to near adult height in Japanese short children born SGA: final report from a multi-center, open-label, long-term study

### Toshiaki Tanaka^1^, Susumu Yokoya^2^, Yuko Hoshino^3^, Shintaro Hiro^3^, Nobuhiko Ohki^3^

#### ^1^Tanaka Growth Clinic; ^2^National Center for Child Health and Development; ^3^Pfizer Japan Inc

**Objective**: We conduct a multicenter, open-label, study to evaluate safety and efficacy of long-term treatment of Genotropin® in short children born SGA without epiphyseal closure. This represents the unique clinical trial in Japanese subjects treated with continuously high dose of rhGH (0.067 mg/kg/day) until near adult height (NAH).

**Method**: Sixty-seven short children born SGA were included from 20 sites in the one-year treatment, Study 002 (Tanaka, et al. 2008), and were randomized to receive two different doses (0.033 or 0.067 mg/kg/day). After completing one-year of treatment in Study 002, out of 62 subjects who were entered in this study, 61 were treated with the dose of 0.067 mg/kg/day and continued until pre-specified treatment termination criteria were fulfilled. This study was continued after the approval of the indication as a post-marketing clinical trial (Clinicaltrials. gov: NCT01859949). Descriptive statistics for each endpoint were summarized by treatment group based on assignment in Study 002. NAH was defined in this study as an “annual height velocity < 2 cm after achieving peak velocity at puberty” or “reaching an epiphyseal closure (centrally measured by the TW2-RUS method or investigator’s evaluation)”; these are the treatment termination criteria.

**Result and Conclusion**: Treatment-related adverse events were 54 events in 22 (36.1%) of the 61 subjects throughout the treatment period. Most of the observed adverse events were mild to moderate in severity. Two subjects experienced adverse event leading to permanently discontinuation and two subjects had treatment-related serious adverse event, although which outcomes to have resolved. No clinically significant changes were observed for HbA1c levels and bone maturation throughout the study. Neither new safety information nor safety concern was identified in this long-term study. Twenty subjects (11 boys and 9 girls) reached NAH (mean age (year); mean cm [height SDS]), 16.0; 159.1 [-1.56] in boys and 14.3; 146.9 [-1.61] in girls, at the end of treatment. We have confirmed the safety and efficacy of high dose rhGH treatment in the more than a decade long-term clinical trial in Japan.

## P1-4-8 Study of mRNA expression of Insulin/IGF axis in neonatal period

### Masanobu Fujimoto^1^, Yuki Kawashima Sonoyama^1^, Aya Imamoto^1^, Naoki Miyahara^1^, Fumiko Miyahara^1^, Rei Nishimura^1^, Mazumi Miura^1^, Keiichi Hanaki^2^, Susumu Kanzaki^1^

#### ^1^Division of Pediatrics and Perinatology, Tottori University Faculty of Medicine; ^2^Department of Women's and Children's Family Nursing, Tottori University Faculty of Medicine

**Context**: Hypoglycemia in neonate is caused by hyperinsulinism, inadequate glycogen stores and impaired glycogenolysis or gluconeogenesis. However the pathophysiological condition still remains unknown, and the contribution of the insulin/insulin-like growth factor I (IGF-I) signaling pathway including insulin receptor substrate (IRS) to neonatal hypoglycemia are not clearly known.

**Objective**: To elucidate relationship between glucose level and insulin/IGF-I signaling in appropriate for gestational age (AGA) and small for gestational age (SGA) neonates.

**Patients and Methods**: Neonates born in the Perinatal Medical Center of Tottori University Hospital were enrolled. We measured glucose level, serum levels of insulin and IGF-I of samples collected from umbilical cord or venous blood at birth. Using same samples, we evaluated the expression of IGF-1 receptor (IGF1R ), insulin receptor (INSR ), insulin receptor substrate-1 (IRS-1 ), IRS-2 , glucose transporter 2 (SLC2A2 ) and SLC2A4 mRNA by quantitative real-time PCR.

**Results**: Forty three neonates (38 AGA neonates and 5 SGA neonates) were enrolled. Mean gestational age was 38.5 weeks (range 37-41 weeks). Mean birth weight in AGA was 3,006g (mean ± SD: 0.19 ± 0.92) and in SGA; 1,832g (-2.6 ± 0.82). Blood glucose level of SGA was significantly lower than that in AGA [SGA, 40.8 ± 12.3 mg/dL vs. AGA, 85.8 ± 33.7 mg/dL]. Serum insulin and IGF-1 levels of AGA were slightly higher than those of SGA. As both groups did not show any increase of insulin to glucose ratio, they did not have hyperinsulinism. IRS-2 and SLC2A4 mRNA expression were significantly higher in SGA group. There was no difference in the expression of IGF1R, INSR, IRS-1 and SLC2A2 mRNA in between SGA and AGA.

**Discussion and Conclusions**: The expression of IRS-2 protein is increased by undernutrition, and reduced by insulin. It was suggested that the increased expression of IRS-2 mRNA on SGA was caused by fetal undernutrition, and hyperinsulinism was not occurred in fetal period on SGA in our study. These findings reveal that intrauterine growth restriction induce the change of insulin/IGF axis, and provide the important information of neonatal hypoglycemia.

## P1-4-9 Adult height in Japanese Prader-Willi syndrome patients with growth hormone treatment

### Yoriko Hatta^1,2^, Takashi Kamijo^3^

#### ^1^Department of Pediatrics, Japan Red Cross Nagoya Daiichi Hospital; ^2^Iwayama Pediatric Clinic; ^3^Nagoyaka Children's Clinic

**Background**: Prader-Willi syndrome(PWS) is a neuro-developmental genetic disoeder caused by lack of expression of genes on the paternally inherited chromosome 15q11.2-q13 region.Short stature is one of the most prominent feature of them. In Japan, growth hormone (GH) treatment was approved in January 2002 for the indication of short stature ( below -2SD of normal people) and growth failure due to PWS.Adult heights of untreated Japanese PWS patients are reported 147.7 ± 7.7cm i-4.0 ± 1.3SD ) for male and 141.2 ± 4.8cm i-3.2 ± 0.9SD ) for female.

**Objective**: To search for effectiveness of GH, we determined the the final height and body mass index (BMI) of PWS patients.

**Patients and methods**: Final height and BMI was reviewed in the medical records of 26 PWS patients, treated with and without Growth hormone at Nagoyaka Children’s Clinic. Nine patients were male and

14 patients were female.Three patients who treated with GH for less than 5 years were excluded. Height was expressed as standard deviation scores (SDSs) of normal Japanese people.

**Results**: Table

**Discussions**: Final mean height in Japanese PWS patients without GH is -4.0 ± 1.3SD for male and -3.2 ± 0.9SD for female. It can be said short stature is more prominent in male. Final height after GH is -0.2SD ± 1.1 for male and -1.0SD ± 0.5 for female. The effect of GH for final height is greater for male than female. Mean BMI is 21.5 ± 4.1 for male and 20.3 ± 3.8 for female. Considering BMI is higher in female than male by nature, the improvement of BMI is more remarkable in female.

**Conclusion**: Our data clearly show administration of GH to PWS patients improved final height and BMI. It is suggested the effect is more prominent for male than female. It is not sure the change will continue for life and contribute to better quality of life. Further studies will be required to determine the long term effect.

**Table 1 (abstract P1-4-9).**
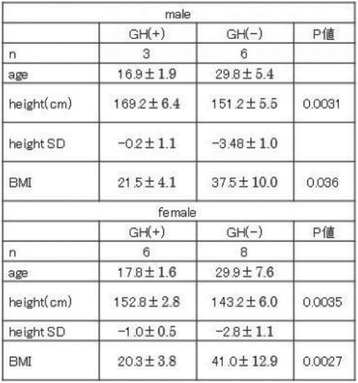
See text for description

## P1-4-10 Idiopathic short stature children with free T4 in the low-normal range during the first year of therapy with recombinant human growth hormone needs attention

### Wei Wang, Ling Li Shi, Zhi Rui Cui

#### Third Affiliated Hospital of Zhengzhou University, Pediatric Endocrinology Clinic

**Background**: Appropriate L-thyroxine (L-T4) substitution is necessary for the optimal effect of growth hormone (GH) administration on growth rate. A decrease in free thyroxine (FT4) levels at recombinant human GH (rhGH) therapy onset has been reported by several studies. However, their results are conflicting. The aim of the present study was to evaluate the effects of thyroid hormone supplementation on growth rate in children with ISS and low normal FT4 who are receiving GH therapy.

**Methods**: We selected 64 prepubertal children with FT4 levels in the lowest third of the normal range (FT4:11.5-15.2 pmol/L) as Lower FT4 groupand they were divided randomly into two subgroups: L-T4 treated subgroup included 29 ISS children with L-T4 (0.5 to 3.0 mg/kg/d) from the beginning of rhGH therapy, non-L-T4 treated subgroup included 33 ISS children received placebo during the same period. We also selected 39 ISS children with FT4 in the upper two-thirds of the normal range (FT4:18.9-22.7 pmol/L) as Higher FT4 group(control group). In all children, height velocity (HV) were assessed before and after 1 year of rhGH therapy, whereas thyroid stimulating hormone (TSH), FT4, FT3 and insulin-like growth factor-I (IGF-I) were assessed before treatment and after 3 to 6 months and 1 year of treatment.

**Results**: During 1st year of rhGH administration, Lower FT4 grouphad lower FT3, FT4, TSH, IGF-I SDS compared with higher FT4 group and their HV was significantly lower(9.64 ± 0.75 vs 11.09 ± 0.63/12 months). However, in Lower FT4 group, after L-T4 supplementation, L-T4 treated subgroup had higher FT4, FT3, TSH and IGF-I SDS concentrations compared with those non-L-T4 treated subgroup and their HV was significantly higher( 12.03 ± 0.70 vs 9.64 ± 0.75/12 months).

**Conclusions**: In children with ISS, the negative effect of thyroid hormone deficiency on growth rate should be considered when the FT4 level is in the low-normal range prior to rhGH treatment.

## P1-4-11 Growth and glucose metabolism after allogenic bone marrow transplantation for thalassemia major

### Wenqin Lao^1^, Liyang Liang^2^, Hui Ou^2^, Zhe Meng^2^, Lele Hou^2^

#### ^1^Department of Pediatrics, The Second Affiliated Hospital of Guangzhou Medical University; ^2^Department of Pediatrics, Sun Yat-sen Memorial Hospital

**Background**: Growth failure and abnormal plasma glucose level are common in patients with thalassemia major (TM), which are usually due to iron overload after repeated blood transfusion. Patients after successful bone marrow transplantation (BMT) will be free from further blood transfusion and thus spared from complications of iron overload. This study aimed at determining the incidence of abnormal glucose level in TM patients and studying the height difference between the TM patients who underwent BMT and who did not.

**Objective and hypotheses**: The “BMT group” consisted of 19 patients with TM who had undergone BMT and were followed up for at least two years. 57 TM patients of similar age who did not undergo BMT were recruited as the “non-BMT group”.

**Method**: The age at BMT and present age, weight, height, height standard deviation score (SDS) and weight SDS values, serum ferritin (SF), the fast blood glucose and insulin level were evaluated.

**Results**: The mean age was 10.3 ± 3.6 years and transplantation age was 6.29 ± 3.4 years. The SF of the BTM was lower than the non-BTM group (P =0.002). The height SDS score in BTM was found better than the non-BTM (P = 0.039). There were no statistical differences in fast blood glucose and insulin level between two groups. 15.79% i3/19 ) of BTM patients had insulin resistance, while it was 14.81%(8/54) in non-BTM group iP =1 ). None of the BTM had impaired fast glucose (IFG) or diabetes mellitus (DM). While in non-BTM group, the prevalence was 18.5% i10/54 ) and 3.7% i2/54 ) respectively ip =0.029 ).

**Conclusion**: Allogeneic BTM may improve short stature of TM patients. Although BMT didn’t alter the abnormal glucose status of TM completely, it prevented this disease from exacerbating. The patients should be followed up regularly after transplantation.

## P1-4-12 A case with duplication of 20qter and deletion of 20pter due to maternal pericentric inversion, presenting with Silver- Russell syndrome-like phenotypes

### Chie Takahashi^1^, Akie Nakamura^2^, Mayuko Tamura^1^, Hiroyuki Tanaka^1^, Tsuyoshi Isojima^1^, Akira Oka^1^, Masayo Kagami^2^, Sachiko Kitanaka^1^

#### ^1^Department of Pediatrics, The University of Tokyo Hospital; ^2^Department of Molecular Endocrinology, National Research Institute for Child Health and Development

## P1-4-13 A Novel de novo Germline Mutation Glu40Lys in AKT3 causes Megalencephaly with Growth Hormone Deficiency

### Keiko Nagahara^1,2^, Masaki Takagi^1,3^, Takanari Fujii^2^, Gen Nishimura^4^, Mitsuhiro Kato^2^, Kazushige Dobashi^2^, Kazuo Itabashi^2^, Satoshi Narumi^3^, Tomonobu Hasegawa^3^

#### ^1^Department of Endocrinology and Metabolism, Tokyo Metropolitan Children's Medical Center; ^2^Department of Pediatrics, Showa University School of Medicine; ^3^Department of Pediatrics, Keio University School of Medicine; ^4^Department of Radiology, Tokyo Metropolitan Children's Medical Center

**Background**: Gain-of-function mutations in the v-akt murine thymoma viral oncogene homolog 3 (AKT3 ) have been reported to cause megalencephaly-related syndromes. To date, three gain-of -function germline AKT3 mutations have been reported in 6 MCAP/MPPH patients. Here, we describe a novel germline mutation, p.Glu40Lys, in AKT3 causing megalencephaly with growth hormone (GH) deficiency.

**Case report**: The patient, a 5-year-old Japanese boy, is the first child of non-consanguineous healthy parents. He was vaginally delivered at 40 weeks of gestation. His birth weight was 4.2 kg (+ 2.9 SD), length was 54.0 cm (+ 2.5 SD), and head circumference (HC) was 39.0 cm (+ 4.4 SD). He was referred to us at the age of 7 months because of poor body weight gain and hypotonia. He had frontal bossing, and was unable to hold his head steady. Brain magnetic resonance imaging (MRI) showed neither polymicrogyria nor ventricular dilation. At 15 months of age, he was admitted to our hospital by hypoglycemia. Hyperinsulinism and central adrenal insufficiency were excluded based on the analysis of critical samples. Low serum GH level (de novo novel heterozygous c.118G>A transition (p.Glu40Lys) in AKT3 . The phosphorylation in CHO cell level of the E40K AKT3 variant was upregulated relative to control, indicating that E40K ATK3 was a gain-of-function mutation.

**Discussion**: In this case, it is indicated that hypoglycemia is induced by GH deficiency. The severity of the meurogical changes in our patient was milder than in patients with other gain-of-function germline AKT3 mutations. To our knowledge, this is the first case of megalencephaly and GH deficiency, harboring a germline de novo mutation in AKT3. The association between AKT3 mutations and GH deficiencies remains to be elucidated. Therefore, further studies are necessary to determine the independent contribution of AKT3 mutations to the development of GH deficiency

**Consent for publication**: The authors declare that written informed consent was obtained for publication.

## P1-4-14 Onset of puberty in extremely low birth weight children born small for gestational age and treated with GH

### Shinsuke Onuma, Shinnosuke Tsuji, Mikiko Koizumi, Hiroyuki Yamada, Yasuko Shoji, Masanobu Kawai, Yuri Etani, Shinobu Ida

#### Department of Pediatric Gastroenterology, Nutrition and Endocrinology, Osaka Medical Center and Research Institute for Maternal and Child Health

**Background**: The effects of growth hormone (GH) treatment have not been sufficiently evaluated in extremely low birth weight (ELBW) children born small for gestational age (SGA). One of the objectives of GH treatment for SGA short stature is to achieve normal adult height. Because the height at the onset of puberty is critical to determine the adult height, we evaluated the effects of GH treatment in SGA short stature with ELBW on the onset of puberty.

**Subjects and Methods**: Fifteen children (10 boys and 5 girls), who were born with ELBW and received GH treatment for SGA short stature in our hospital, were recruited in this study. GH treatment was initiated before the onset of puberty, and the height and the age of pubertal onset was analyzed retrospectively based on the medical records.

**Result**: The median gestational age was 29.1 weeks (range; 26.3 `33.1 weeks), and the median birth weight was 680g (range; 356 `978g). At the initiation of GH treatment, the mean age was 5.0 years (range; 3.2 `8.4 years), and the mean height SD score was |3.0. The median age at onset of puberty was 11.4 years in boys and 10.4 years in girls. The mean height at onset of puberty was 135.3 cm in boys and 130.1 cm in girls. The mean change in height SD score from the initiation of GH treatment to pubertal onset was {1.6.

**Discussion and Conclusion**: The current study revealed that the timing of pubertal onset was not accelerated by GH treatment in SGA children with ELBW. Moreover, GH treatment resulted in an increase in height SD score at the pubertal onset. These findings indicate that GH treatment is an effective approach to normalize the adult height without affecting the timing of puberty in SGA children with ELBW; however, the effects of GH treatment on the adult height in these children are not fully understood. Further studies are clearly required to reveal the effects of GH treatment in SGA children with ELBW.

## P1-4-15 Nipbl deletion in pyramidal neurons results in postnatal growth retardation and early lethality

### Xiaohui Wu, Chun Chao Zou

#### Department of Endocrinology, Children's Hospital of Zhejiang University School of Medicine

Cornelia de Lange syndrome (CdLS) is a genetically and clinically heterogeneous disorder characterized by growth and mental retardation. It is caused by mutations in core cohesin subunits SMC1A, SMC3 and RAD21, or their regulators NIPBL and HDAC8. Nipbl accounts for most cases of CdLS. It has been implied NIPBL has a “dual role” in loading cohesin and in cohesin-independent gene regulation. Yet, its role in the maintenance of balance of neural function remains unknown. To address these questions, selective deletion of Nipbl was achieved in late postnatal (CaMKII aNipblcKO) developmental stages. Although born healthy with expected Mendelian frequencies, CaMKII aNipblcKO exhibited severe growth retardation and pre-weaning lethality. As demonstrated by ISH or real-time PCR, the mRNA level of nipbl at P3 in CaMKII-Cre; nipbl flox/flox was comparable to that in the littermate heterozygous CaMKII-Cre; nipblflox/+ and CaMKII-Cre; nipbl+/+, but was largely diminished at P15 Cand the absence of NIPBL in hippocampus and cortex was dramatic, but also reduction of NIPBL mRNA was shown in olfactory bulb and hypothalamus, consistent with the expression pattern of iCre in previous report. At birth, the appearance and size of the CaMKII aNipblcKO pups were similar to their WT and heterozygous littermates, however, by P3, CaMKII aNipblcKO mice failed to thrive and displayed severe growth retardation. Closer examination revealed that CaMKII aNipblcKO failed to suckle effectively and had stomachs devoid of milk. Suckling reflex is thought to be processed by neural networks involving the olfactory bulb and the hippocampus. The brain size of the homozygous pups and control group was significantly comparable. Detailed histological examination revealed the cerebral cortex of CaMKII aNipblcKO was remarkbly thinner than the controls. Together, these data suggest that nipbl in neurons controll the suckling response at this stage.

## P1-4-16 A 3-year-old girl with 46,XX, upd(14)mat/47,XX,+14 mosaicism caused by postzygotic duplication of a maternally derived chromosome 14

### Kikumi Ushijima^1,2^, Syuichi Yatsuga^1^, Akie Nakamura^2^, Yasutoshi Koga^1^, Maki Fukami^2^, Masayo Kagami^2^

#### ^1^Department of Pediatrics and Child Health, Kurume University School of Medicine; ^2^Department of Molecular Endocrinology, National Research Institute for Child Health and Development

**Background**: The common symptoms of trisomy 14 mosaicism are growth retardation, developmental delay, facial dysmorphic features, abnormal ears, cardiac anomalies and genitourinary abnormalities. Approximately 40 cases of trisomy 14 mosaicism with a wide range of symptoms have been reported. Maternal uniparental disomy of chromosome 14 (upd(14)mat) is also a rare chromosomal disease. The main clinical symptoms of upd(14)mat are prenatal and postnatal growth retardation, hypotonia, and early-onset puberty. We present a severely short statured 3-year-old girl, who had trisomy 14 mosaicism with upd(14)mat.

**Case report**: A 3-year-old girl was referred to our hospital due to short stature, failure to thrive (height: 78.8cm (-5.08 SD), weight: 8.77kg (-3.31 SD)) and developmental delay. She was born at term, but her height and weight were lower than 10th percentile at birth. She had mild facial dysmorphic features, ear malformation, deafness, coloboma and closed ventricular septal defect. Endocrine tests including thyroid function and growth hormone measurements showed normal findings. Methylation analysis using genomic DNA extracted from her leukocytes was performed, and we identified mild hypomethylation at the MEG3-DMR at the 14q32.2 imprinted region. Genome wide comparative genomic hybridization array revealed no specific copy-number change, but fluorescence in situ hybridization analysis revealed trisomy 14 mosaicism in blood (proportion of trisomy 14 cells was 46.8%). Microsatellites analysis of chromosome 14 resulted in the diagnosis of trisomy 14 mosaicism and upd(14)mat.

**Discussion**: We report the second and youngest case of trisomy 14 mosaicism with upd(14)mat. Previous cases of trisomy 14 mosaicism had mild to moderate short stature, moreover, upd(14)mat patients also exhibited short stature. Considering the severe short stature in our case, we believe that the combination of trisomy 14 mosaicism and upd(14)mat severely affects stature growth.

**Conclusion**: We report severe short stature associated with trisomy 14 mosaicism and upd(14)mat. Detailed genetic analysis is required for patients with severe short stature. Follow up studies of our patient will clarify whether she develops other symptoms of upd(14)mat such as early-onset puberty.

**Consent for publication:** The authors declare that written informed consent was obtained for publication.

## P1-4-17 Acromicric Dysplasia Caused by a Novel Heterozygous Mutation of FBN1 and Experience of Growth Hormone Treatment

### Su Jin Kim^1^, Sung Won Park^2^, Young Bae Sohn^3^, Sung Yoon Cho^4^

#### ^1^Department of Pediatrics, Samsung Medical Center, Sungkyunkwan University School of Medicine; ^2^Department of Pediatrics, Cheil General Hospital, Dankook University College of Medicine; ^3^Department of Medical Genetics, Ajou University Hospital; ^4^Department of Pediatrics, Myongji Hospital, Seonam University College of Medicine

Acromicric dysplasia (AD, MIM: 102370) is a rare skeletal dysplasia characterized by short stature, short hands and feet, mild facial dysmorphism, normal intelligence, and characteristic radiographic findings of the hands. AD is caused by a mutation in the fibrillin 1 (FBN1 ) gene. Here, we describe the clinical findings and an experience of growth hormone (GH) treatment in a child with AD caused by a novel heterozygous mutation, c.5282C>T (p.Thr1761Ile), in FBN1 (NM_000138.4). The patient showed growth retardation, short and stubby hands and feet, mild facial dysmorphism, and normal intelligence. A skeletal survey at the age of 11 years showed delayed bone age, brachymetacarpy, and acetabular dysplasia. He has been diagnosed as partial GH deficiency and treated with GH for 3.5 years with good response. Similar to the previous reports, the mutation in our patient was located in the TB5 domain, which suggests that this domain of FBN1 is responsible for the pathogenesis of AD.

## P1-4-18 Effect of Growth Hormone Therapy on Height Velocity in Korean Children with Idiopathic Short Stature (ISS): a Randomised Controlled Trial

### Woo Yeong Chung^1^, Premila Paranchothy^2^, Byung-Kyu Suh^3^

#### ^1^Paediatrics Department, Inje University Busan Paik Hospital; ^2^Merck Ltd, Asia Pacific Regional Medical Affairs; ^3^Department of Pediatrics, The Catholic University of Korea

**Background**: Idiopathic short stature (ISS) is the commonest cause of referral to paediatric endocrinology clinics and is also a frequent cause of referral to general paediatricians and primary care health workers. For the children affected, there is an unmet need to improve height prognosis and growth rates.

Aim: The aim of the current study was to evaluate the effects of recombinant human growth hormone (GH, Saizen®, Merck) treatment on height velocity (HV) in Korean pre-pubertal children ( ≥ 5 years of age) with ISS.

**Methods**: A 12-month, open-label, randomized, two-arm, parallel-group, Phase III study conducted in nine centres throughout South Korea. Patients were randomised 2:1 to receive either 0.067mg/kg/day GH for 12 months (treatment group, n=60, 31 males) or 6 months of no treatment then 6 months 0.067 mg/kg/day GH (control group, n=30, 17 males); assessments were at screening, baseline (Month 0) and at Months 3, 6, 9, and 12. The primary efficacy endpoint was change in ( Δ ) HV from baseline to 6 months of study. Secondary endpoints included Δ HV over 12 months, Δ Height (cm) and Δ Height SDS over 6 and 12 months, Δ IGF-I and Δ IGFBP-3 levels (Siemens Immulite 2000 immunoassay system), adherence and safety.

**Results**: At 6 months, the mean Δ HV from baseline was significantly greater (*p*<0.0001) in the treatment group ( Δ HV = 4.45 ± 2.70 cm/year, LS mean = 4.47, SE = 0.35; n=59) than in the control group ( Δ HV = 1.01 ± 3.15 cm/year, LS mean = 0.99, SE = 0.54; n=29). In the GH treatment group (n=59), the increase in HV of 4.45 ± 2.70 cm/year observed after 6 months, compared to the baseline value, was also clinically relevant. Patients treated with GH for 12 months (n=52) continuously demonstrated a significant increase in Δ Height SDS compared to the control group (n=27), both at 6 months (difference between LS means 0.51 [95% CI 0.42, 0.60]; p<0.0001) and at 12 months (difference between LS means 0.33 [95% CI 0.21, 0.45]; p<0.0001), respectively. Serum IGF-I levels at 6 months were significantly increased (*p*<0.0001) in the treatment group (122.57 ± 96.9 μ g/L) versus the control group (8.30 ± 41.57 μ g/L). No treatment-related SAEs were reported and there were no treatment discontinuations due to adverse events.

**Conclusions**: Korean children with ISS receiving Saizen® GH showed a significantly increased HV and change in height after 6 months of treatment and the increased growth was maintained over 12 months. Saizen® GH was well tolerated.

## P1-4-19 Outcome of Growth Hormone Therapy on children with idiopathic growth hormone deficiency: an Indian experience

### CECIL Christopher Khakha^1^, Pragya Jain^1^, Goldee Singh^1^, Amit Jaggi^1^, Deepika Cecil Khakha^2^

#### ^1^Department of Pediatrics, E.S.I. Hospital , Okhla Indutrial Area Phase-1; ^2^All India Institute of Medical Sciences , College of Nursing

**Background**: Recombinant Growth Hormone(rhGH) therapy has been into use for the last three decades but it became more accessible for use in Indian children in the last decade . The authors were fortunate to use it and are sharing their clinical experience.

**Aim**: To study the outcome of recombinant Growth Hormone on children with Idiopathic Growth Hormone deficiency.

**Method**: A prospective cohort of 138 short statured children attending Pediatrics OPD during period from May 2005 to December 2013 who received recombinant Growth Hormone therapy .

**Results**: During the study period 138 children were labeled as “Short Stature” based on clinical , auxological and biochemical criteria. However 58 children (37 boys & 21girls) were identified as having Short Stature due to Idiopathic Growth Hormone Deficiency and received minimum two years of rhGH therapy. 14 children were less than 8 years and 44 children were more than 8 years at the time of diagnosis. All these children were Growth Hormone deficient (Average peak GH levels on provocative testing was 3.90 +/- 4.05 ng/ml). Average initial height at beginning of therapy was 124.06 cm (minimum 80 cm and maximum 146 cm). Average height SDS at start of treatment was – 2.73 (minimum -4.8 and maximum -2.1). 26 children completed rhGH treatment during the study period and 24 reached their predicted height in their target range. Average final height at end of therapy was 156.99 cm (maximum 167.8 cm and minimum 133.8 cm). Average Height SDS at end of therapy was -0.98(minimum -3.38 and maximum 0.1). Average height gain in first year of treatment was 9.0 cm (maximum 11.5 cm and minimum was 8.0 cm) and in the second year of treatment was 7.5 cm(maximum 9.0 cm and minimum 6.5 cm). Of the children who took treatment for extended period, they gained on average 6.4 cm during third year, 5.9 cm during fourth year and 5.4 cm during fifth year of rhGH therapy. There were no reported adverse effects of the therapy.

**Conclusion**: Indian Idiopathic Growth Hormone deficient children respond well to rhGH therapy and the usage of rhGH is relatively safe.

## P1-4-20 GnRH agonist treatment for central precocious puberty can improve the final adult height in Chinese girls

### Ying Yanqin, Hou Ling, Liang Yan, Xiaoping Luo

#### Pediatric Department, Tongji Hospital, Tongji Medical College, Huazhong University of Science and Technology

**Objective**: To study the outcomes of GnRHa on final adult height in Chinese ICPP girls.

**Methods**: In this study, we retrospect our 10 years data from 3 clinical hospitals between 2004 to 2014, and 101 girls with ICPP received GnRHa therapy more than 6 months reached their adult height were enrolled. These patients were treated by Leuprorelin or Triprorelin without recombined human growth hormone. Height, bone age, midparent height, HtSDS, sexual development, therapy duration and PAH at the start of GnRHa and end of GnRHa, and final adult height were calculated. We compared not only PAH at start of GnRHa and end of GnRHa, and final adult height, but also the percentage of short stature according to their midparent height and normal Chinese girls’ height distribution. At the same time, we evaluated the correlation between adult height and other factors.

**Results**: 101 girls with ICPP were enrolled in this study. Their average age at the start of puberty was 7.1 ± 0.75 years, and they started to therapy with GnRHa at 8.4 ± 0.84 years, bone age were 10.6 ± 0.53 years. Their PAH at end of GnRHa (153.1 ± 5.37 cm) increased significantly compared to that at start of GnRHa (158.4 ± 6.00cm), P<0.001, and their final adult height (157.0 ± 4.82) increased significantly compared to PAH at the start of GnRHa(P<0.001), but there was no difference between PAH at end of GnRHa and FAH(p>0.05). After GnRHa therapy, most of ICPP girls reached their midparent height compared to that at start of GnRHa(P<0.01). FAH was positively correlated with height at start of GnRHa, height at end of GnRHa, PAH at start and end of GnRHa, and also midparent height (R2=0.43, 0.61, 0.82, 0.89 and 0.75, P<0.001), while FAH was not correlated with duration of treatment, BA at start of GnRHa(R2=-0.08, 0.1, p>0.05). The percentage of adult short stature and reached midparent height increased significantly after GnRHa therapy compared to that at start of GnRHa(60.6% vs 30.4% and 67.85% vs 94.64%, P<0.05, respectively).

**Conclusions**: GnRHa therapy to ICPP girls can achieve the final height effectively, and after GnRHa therapy, most of patients reach their midparent height and less of patients become adult short stature.

## P1-4-21 Comparison of growth in children from rural pune, India, on various growth references

### Rahul Jahagirdar, Priya Gupta, Vaman Khadilkar, Ruma Deshpande, Sanjay Lalwani

#### Department of Pediatric Endocrinology, Bharati Vidyapeeth University Medical College Pune

**Introduction**: Continued growth monitoring is a quick, easy, inexpensive and non-invasive method of assuring the general wellbeing of the children. Children in rural areas have high prevalence of anaemia, underweight and stunting.

Evaluation of growth necessitates the use of growth charts. We tried to evaluate the growth parameters of school children on the Indian 1992, Indian 2015 and CDC 2000 references to assess the growth performance of rural school children in India.

**Aim**: To assess the growth parameters of height, weight and BMI of rural children and adolescents of Pune, India on the various growth references.To assess the suitability of the charts for the present Indian rural population

**Material and Methods:** This was an Observational, Cross sectional field study across socioeconomic class conducted for around 22 months on 2162 children, (1151) boys ( 5-15 years) from rural Pune. Z scores according to the Indian 1992; Indian 2015 and CDC 2000 references for height, weight and BMI were calculated for comparison.

**Observation and Results**: Each of these categories were further divided into 3 more sub categories depending on the type of school the children attended to see if there was a significant difference. Indian 1992 charts consistently gave the least prevalence for undernourished categories like stunting, wasting and underweight. It misses out on picking many undernourished children as its lower cut offs are very low and for the same reason it classified more children as taller or overweight.CDC estimated far too many children as underweight, stunted and wasted as it has very high cut off values. Indian 2015 charts are the most appropriate for the rural children of Pune. It showed prevalence of stunting among the children is 10.9 %, and tall children are only 0.6%. The prevalence of wasting is 12.7 %, Underweight children account for 6.9%, overweight for 6.4% and obese for 2.0%. According to Indian 2015 charts, Z score analysis of weight shows that the prevalence severely underweight and underweight , severe wasting and wasting, severe stunting and stunting is more in girls as compared to boys. School wise categorisation showed that the among the children of the fully paid schools, the prevalence of undernourished states like stunting, wasting and underweight were least, while the prevalence of overweight and obesity were the highest.

## P1-4-22 A secular trend of ages at menarche and spermarche in Korean adolescents over a decade: using the Korea Youth Risk Behavior Web-based Survey (KYRBS) 2006-2015

### Hae Kim^2^, Eun Mi Jung^1^, Eun Hee Ha^1^

#### ^1^Department of Preventive Medicine, School of Medicine, Ewha Womans University; ^2^Department of Pediatrics, School of Medicine, Ewha Womans University

**Purpose**: It is wildly noted that spermarche begins at SMR 3 in boys and menarche begins at SMR 3-4 in girls. While previous studies have demonstrated that the mean age of menarche has been declining constantly, little is known about the secular trend in mean age at spermarche. Furthermore, it is rare that a study reports the secular trend in mean ages of both menarche and spermache in Korean population. The objective of this study is to reveal the pubertal trend in age at menarche and spermarche in Korean adolescents over the past decade since 2006.

**Methods**: Data collected from the KYRBS between 2006 and 2015 were used for the analysis. KYRBS is a cross-sectional, nationwide school- based web survey with a stratified multistage probability sampling design. It has a national representative sample of Korean students in Grades 7-12 and is conducted annually. Participants are Korean adolescents randomly assigned from 400 middle and 400 high schools. We conducted a cross-sectional analysis of self-reported age at menarche and spermarche. Reponses from those who have not experienced menarche/spermarche at the time of the survey were not included for the analysis. Descriptive statistics were calculated to demonstrate the general characteristics of the study population.

**Results**: The sample size of a survey varies from 68,043 to 75,643, of which approximately 52-3% are boys and 46-47% are girls. Through years, the minimum average age of the study population is 14.94 (SD=0.02) in 2006, and the maximum is 15.15 (SD=0.02) in 2010. Timing of menarche and spermarche tends to shift downward; while increases of 5.43% and 0.16% in experiencing menarche and spermarche, respectively, were observed in Grade 7, decreases of 3.57% and 5.43% in experiencing menarche and spermarche, respectively, were observed in Grade 9. In addition, the mean age at menarche in 2015 was 12.241 years, which showed a decrease from 12.457 years in the 2005. The mean age at spermache in 2015 was 12.997 years, which showed a notable decrease from 13.148 years in the 2005.

**Conclusion**: We have found that ages at menarche and spermarche have been falling in the Korean adolescents for the last 10 years. Further analysis including potential risk factors of early menarche and spermarche needs to be performed to identify underlying causes of these secular trends in the Korean adolescents.

## P1-4-23 Nutritional status and anaemia among primary school children in mymensingh district

### Mohammad Hasan Mahmudul

#### Department of Paediatrics, Mymensingh Medical College

**Introduction**: The school age is nutritionally significant because this is the time to built-up body stores of nutrients in preparation for rapid growtg of adolescence.

**Objective**: The purpose of the study is to observe nutritional status and anaemia among the school aged children and to compare the urban and rural children as well as to see the correlation between malnutrition and anaemia .

**Methods**: It is a descriptive cross sectional study. data were collected from 600 primary school children of mymensingh district for a period of one year.

**Results**: Among the primary school children in mymensingh 15.1% were wasted, 22.1% were stunted, 2.3%were both stunted and wasted, 60.4% children were within normal limit. Malnutrition were more in rural areas in comparison with urban area. In rural areas severly underweight, moderately underweight children 72.4%, 73.6% and corresponding result in urban areas were 27.6%,26.4% respectively. In rural area severly stunted, moderately stunted children were 100%,58.6% and in urban area they were 0%,41.4% respectively. Again severly wasted and moderately wasted children were 62.5%, 59.5% rural areas and 37.5%, 40.5% in urban areas respectively. Malnutrition among girls were more then the boys.71% of our primary school children were anaemic. Again rural children are more sufferer. Anaemia was more in low income group and illiterate mother's child. Some anaemic children are malnourished and some were not but there were significant relationship between anaemia and malnutrition.

**Conclusion**: A large portion of our primary school children suffered from malnutrition and anaemia and both the conditions were more prevalent in rural areas. an endocrinological survey could be done to asertain any endocrinological cause realted behind this growth deficiency.

## P1-4-24 Efficacy and safety of recombinant human growth hormone treatment in six Chinese patients with Noonan syndrome

### Zhi-Hui Liu, Shi-yao Wang, Hui Pan, Hong-Bo Yang, Lin-jie Wang, Feng-Ying Gong, Zi-meng Jin, Hui-juan Zhu

#### Department of Endocrinology, Key Laboratory of Endocrinology of Ministry of Health, Peking Union Medical College Hospital, Chinese Academy of Medical Sciences & Peking Union Medical College

**Background**: Noonan syndrome (NS) is an autosomal dominant inherited disorder, characterized by short stature, special face features, and congenital heart disease. Previous studies showed that recombinant human growth hormone(rhGH) can significantly improve the patient's adult height. But there are no reports about clinical manifestations or rhGH treatment responses in Chinese NS patients.

**Methods**: 1. Six NS patients were collected from May 2009 to August 2015 in Department of Endocrinology, Peking Union Medical College Hospital with series of physical examination, laboratory tests, and genetic screening results. 2. Five patients were prescribed with rhGH with the daily dose of 0.04-0.06 mg/kg subcutaneously in the evening and made the regular follow-up. 3. Ten age/gender matched Turner syndrome (TS) patients and 10 growth hormone deficiency (GHD) patients were also collected in the study, aiming to analyze the efficacy differences of rhGH treatment in NS, TS, and GHD patients.

**Results**: 1. Clinical manifestations: all patients manifested short stature(average height -3.6 ± 0.9 SDS) and typical facial features. Five of them had congenital heart disease, four of them showed skeletal system abnormalities. 2. Genetic test results: PTPN11 is the most common affected gene in NS patients (4/6). The other gene mutations including KRAS (1/6) and SOS1 (1/6). 3. Five of six patients were treated with rhGH. The annual growth rate of the patients was significantly increased than before (P <0.01). One patient entered 3-10th percentile of the normal range of height in the 1-year follow-up, while the remaining four patients’ height was still less than 3rd. 4. The age-matched NS and TS patients had the similar efficacy of rhGH treatment in every follow-up. However, compared with age/gender matched GHD patients, NS patients revealed less treatment benefit. 5. Adverse events: two NS patients emerged subclinical hypothyroidism during the follow-up, levothyroxine was administered to correct their thyroid function.

**Conclusion**: NS patients had relatively common symptoms and clinical features. Gene screening is the definite way to confirm the diagnosis. rhGH showed the significant efficacy and safety in the treatment of NS. But the careful assessment of adverse events is still needed in the follow-up.

**Key words**: Noonan syndrome; Turner syndrome, Growth hormone deficiency, rhGH

## P1-4-25 Enzyme replacement therapy ameliorated growth impairment of Mucopolysaccharidosis type II patients

### Yusuke Hamada, Touko Shibuya, Kenichi Yamamoto, Hidehito Kondo, Kaori Irahara, Takanobu Otomo, Norio Sakai, Keiichi Ozono

#### Department of Pediatrics, Graduate School of Medicine, Osaka University

**Background**: Mucopolysaccharidosis type II (MPSII) is X-linked lysosomal storage disorder due to deficiency of iduronate-2-sulfatase, which leads to variable symptoms by the accumulation of heparan sulfate and dermatan sulfate in various tissues and organs. Patients with MPSII generally show overgrowth until the age 3. However their growth is gradually impaired, and eventually patients show short statue. We investigated the effectiveness of enzyme replacement therapy (ERT) to the growth.

**Method/Patients**: We analyzed 10 patients ages at initiation of ERT were 1 to 28 yrs. We divided patients into two groups by the age of the beginning of ERT. 6 patients were before the age of 3yrs (early ERT group), and 3 patients were after 9yrs (late/no ERT group). We compare their growth among these groups by height (HT) and body weight (BW). The length of metacarpal bone (LMB) was measured only in early ERT group. Patients with growth hormone deficiency were excluded.

**Result**: Average HT SDs were in early ERT group; +1.7 at 3yrs, +1.2 at 5yrs and +0.8 at 8yrs, and in late/no ERT group; +0.4 at 3yrs, -0.47 at 5yrs and -3.0 at 8yrs. Average BW SDs were in early ERT group; +3.0 at 3yrs, +1.2 at 5yrs and +1.5 at 8yrs, and in late/no ERT group; +3.6 at 3yrs, +1.9 at 5yrs and -0.4 at 8yrs. Average LMB was -0.6SD at 3yrs and -1.0SD at 8yrs in the early ERT group.

**Discussion**: The effectiveness of ERT was observed with HT, but less for LMB, suggesting that improved joint contracture by ERT contributed more to HT improvement. Earlier diagnosis and treatment are very important to normal growth.

## P1-5-1 Fifty cases of congenital hypothyroidism re-evaluation taken in our hospital

### Akito Sutani, Takeru Yamauchi, Ryuichi Nakagawa, Nozomi Matsuda, Yuichi Miyakawa, Risa Nomura, Atsumi Tsuji-Hosokawa, Shigeru Takishima, Yohei Matsubara, Kei Takasawa, Misako Nagatsuma, Makoto Ono, Kenichi Kashimada, Tomohiro Morio

#### Pediatrics and Developmental Biology, Tokyo Medical and Dental University

**Background**: Congenital hypothyroidism is insufficient production of thyroid hormone which is necessary for normal metabolism, growth and brain development, and its incidence is estimated 1 for 2000~3000 birth in Japan. LT4 supplementation is given priority to precise diagnosis of hypothyroidism, therefore, in order to confirm the diagnosis and to identify the pathophysiology of the condition, the patients are recommended to be re-evaluated at the age of 3 y.o. or after. However, there are few reports to show the details of the re-evaluation to date.

**Objects and Methods**: We retrospectively analyzed the 50 patients (29 males and 21 females) who were diagnosed with congenital hypothyroidism during their neonatal or early infantile period and re-examined during from 2009 to 2015.

**Result**: The diagram of the results was indicated below. The re-examination was performed from 3 to 12y.o.. Out of fifty cases, thirty seven cases (74%) were identified by newborn- screening (NBS), and twenty one patients (42%) revealed positive results on TRH test. Thyroid dysgenesis accounted for 61% (13/21) in permanent CH that was lower than previously reports. Dyshormogenesis that showed elevated level of 123I uptake or perchlorate discharge, were observed in 28% and 44% of permanent and transient CH, respectively. Our data suggested that there are substantial numbers of transient CH pat ients w ith subclinical dyshormogenesis. There were statistically significant difference of the l-T4 dosage between permanent and transient CH, 3.14 ± 1.01 μ g/kg/day and 2.00 ± 0.72 μ g/kg/day (*p*<0.001), respectively.

**Conclusion**: Our present study gave an overview of the clinical details of congenital hypothyroidism, and suggested that substantial number of patients classified transient CH would have mild dyshromogenesis, requiring careful follow–up is necessary even after re-examination.

**Fig. 1 (abstract P1-5-1).**
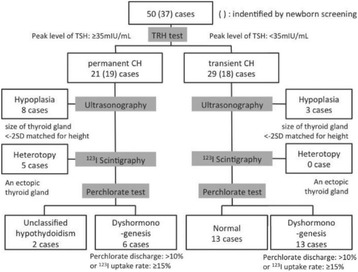
See text for description

## P1-5-2 Mild Thyroid Peroxidase Defect: A Case Series

### Satoshi Narumi^1,2^, Kiyomi Abe^2^, Larry A Fox^3^, Miki Kamimura^4^, Junko Kanno^4^, Ikuma Fujiwara^4,5^, Keisuke Fukudome^6^, Zenichi Sakaguchi^6^, Maki Fukami^1^, Tomonobu Hasegawa^2^

#### ^1^National Research Institute for Child Health and Development, Molecular Endocrinology; ^2^Keio University School of Medicine, Pediatrics; ^3^Nemours Children's Clinic, Endocrinology; ^4^Tohoku University Hospital, Pediatrics; ^5^Tohoku University Graduate School of Medicine, Pediatric Endocrinology and Environmental Medicine; ^6^Takamatsu Red Cross Hospital, Pediatrics

**Context**: Thyroid peroxidase (TPO) is a thyroid-specific membrane-bound enzyme that plays a crucial role in iodination of the thyroglobulin (Tg) protein. The TPO defect, which is caused by biallelic mutations of TPO , is a well-established genetic form of congenital hypothyroidism (CH). More than 100 mutation-proven cases have been published, but only four had a negative result in newborn screening for CH. Here, we report three TPO mutation-carrying hypothyroidism patients that were missed by newborn screening, and had relatively mild thyroid phenotypes.

**Patients**: Three patients were born at term after uneventful pregnancy and delivery, and had a negative result in newborn screening for CH. They were treated with levothyroxine after the diagnosis of hypothyroidism. Patient 1 developed goiter at age 8 years. Blood test showed mild hypothyroidism (TSH 6.47 mU/L, FT4 0.99 ng/dL, FT3 4.10 pg/mL) with an elevated serum Tg level (1,057 ng/mL; reference <30). 123I uptake was high (65% at 4 h; reference 5-15), and the perchlorate discharge test was positive (35.4%; reference <10). Patient 2 had mild developmental delay and was followed up by primary care physician. At age 1 year, she developed goiter, and received her first thyroid function tests, revealing a high serum TSH level (41.5 mU/L), a low FT4 (0.59 ng/dL) level and a high FT3 level (5.85 pg/mL). The serum Tg level was high (3,640 ng/mL). 123I scintigraphy was not performed. Patient 3 had mild developmental delay but the growth was normal. He developed goiter at age 8 years, and thyroid function test showed a slightly high serum TSH level (5.9 mU/L), a low total T4 level (3.5 μ g/dL; reference 6.0-13.8), and a high total T3 level (2.20 ng/mL; reference 0.60-1.81). 123I uptake was elevated (72% at 4 h). Perchlorate discharge test was not performed. His goiter was not resolved by levothyroxine treatment, and he finally received thyroidectomy at age 17 years.

**Genetic analysis**: Sequencing of TPO revealed compound heterozygous mutations in Patient 1 (p.Asp224del / c.820-2A>G), seemingly compound heterozygous mutations in Patient 2 (p.Arg540* / p.Ile657Thr), and a homozygous mutation (p.Trp527Cys) in Patient 3.

**Conclusions**: The TPO defect can be missed by newborn screening. Considering preserved thyroid hormone-producing capacity in Patients 1 and 3, the negative results were possibly associated with residual TPO activity. All three patients had high T3/T4 ratio, indicating elevated type 2 deiodinase activity in their thyroids.

## P1-5-3 Two patients with congenital central hypothyroidism caused by a novel mutation of the IGSF1 gene

### Makiko Oguma, Mizuki Kobayashi, Masayo Yamazaki, Koji Yokoyama, Toshihiro Tajima, Takanori Yamagata

#### Department of Pediatrics, Jichi Medical University

**Context**: Congenital central hypothyroidism(C-CH) is rare disease in which thyroid hormone deficiency is caused by sufficient thyrotropin(TSH) stimulation of a normally-located thyroid gland. Recently, deficiency of immunoglobulin super family member 1(IGSF1) has been demonstrated to cause C-CH.We report Japanese brothers with C-CH caused by a novel mutation of the IGSF1 gene.

Patient finding: As an elder brother showed developmental delay and constipation at one age, he was referred to our hospital. Serum FT4 and TSH were 0.57 ng/dL and 0.67 μIU/mL, respectively. Therefore he was diagnosed as having C-CH. A younger brother showed symptoms of CH, such as poor feeding, hypothermia and prolonged jaundice at 6 days of age. Based on clinical and biochemical evaluation the diagnosis of C-CH was done. Serum FT4 and TSH were 0.38 ng/dL and 1.10 μIU/mL, respectively. In addition, these patients had several characteristic features of IGSF1 deficiency such as heavy birth weight, obesity and hypoprolactinemia.

**Result**: The genetic analysis revealed a novel IGSF1 mutation (c.3607del s, p.W1159G’sx8) in the both patients.

**Conclusion**: We report Japanese siblings with C-CH caused by IGSF1 deficiency. As IGSF1 deficiency shows neurological developmental delay in some patients, it is important for diagnosis of C-CH and prompt initiation of L-T4.

## P1-5-4 Correlation between T3 and Tricuspid valve regurgitation for Graves’ thyrotoxicosis in children and adolescent

### Cheol Hwan So^1^, Jong Seo Yoon^2^, Hae Sang Lee^3^, Jung Sub Lim^4^, Jin Soon Hwang^5^

#### ^1^Division of Endocrinology and Metabolism / Department of Pediatrics, Ajou University School of Medicine; ^2^Division of Endocrinology and Metabolism / Department of Pediatrics, Korea Cancer Center Hospital

**Background and objectives**: Atrioventricular regurgitation, such as tricuspid regurgitation (TR), mitral regurgitation (MR) and mitral valve prolapse (MVP) has high incidence in hyperthyroidism, but only few studies have been conducted in children. Therefore, in the current study, we investigated the incidence and clinical importance of cardiovascular abnormalities in children with Graves’ disease which is the most common cause of pediatric hyperthyroidism. We also evaluated the association between thyroid hormone levels and cardiac abnormalities.

**Methods**: Fifty-one pediatric patients (42 girls/9 boys, 12.29 ± 2.89 years of age) with newly diagnosed thyrotoxicosis due to Graves’ disease were recruited by retrospective medical record review from May 2010 through April 2016 at the Department of Pediatrics in Ajou University Hospital. Medical history, laboratory determinations, complete physical examination results, electrocardiographic findings and echocardiographic findings were recorded for all patients.

**Results**: Major laboratory finding was free T4 3.70 ± 0.88 ng/dL, T3 403.6 ± 165.89 ng/dL, TSH 0.17 ± 0.44 μ IU/mL, TSH receptor- stimulating antibody 36.45 ± 45.15 U/ml. Sinus tachycardia was present in all patients (n=51, 100%), while atrial fibrillation was none. MVP was present in six patients (6 girls, 11.8%). All of them had MR (14 girls, 27.5%). TR observed in nine patients (8 girls/1 boy, 17.6%). TR with MR observed in four patients (3 girls/1 boy, 7.8%). One patient had TR with MVP (1 girl, 2%), two patients had aortic regurgitation (AR, 2 girls, 3.9%) and three patients has left ventricular hypertrophy (LVH, 1 girl/2 boys, 5.9%). There were no differences between group of abnormal echocardiographic findings (n=24, 47.1%) and the normal finding group (n=27, 52.9%) in laboratory findings, symptoms and signs. Likewise, MVP with MR, MR, TR with MVP, AR and LVH were no differences in laboratory finding, symptoms and signs. TR (pressure gradient, mean 38.4mmHg, range 32~54, SD 6.47) was significant correlation with T3 (p 0.031).

**Conclusions**: We observed high T3 level correlated with TR incidence. So we should be concerned with cardiovascular complication, because TR is a pre-state of right heart failure. Furthermore, based on our echocardiographic research, we found that cardiac abnormalities (47.1%) are more likely to happen to children who have a Graves’ thyrotoxicosis. Thus, we suggest that echocardiography should be regarded as routine screening tool to detect cardiac abnormalities in pediatric Graves’ thyrotoxicosis.

## P1-5-5 The clinical predictive factors for differentiation transient congenital hypothyroidism from congenital hypothyroidism patients

### Se Young Kim, Min Sub Kim

#### Department of Pediatrics, Bundang Jesaeng General Hospital, Daejin Medical Center

**Purposes**: The aim of this study was to recognize difference between transient congenital hypothyroidism (TCH) from permanent congenital hypothyroidism (PCH) by determining clinical characteristics, laboratory tests and imaging studies.

**Methods**: We performed retrospective study using database of the patients with congenital hypothyroidism treated with or without Levo- Thyroxine at Bundang Jesaeng General Hospital, from January 1998 to February 2016. Their ages, birth weights, gestational ages, symptoms, ages at diagnosis and treatment were recorded. We measured TSH, free thyroxine(FT4), triiodothyronine(TT3) levels at diagnosis and treatment, and those levels at one, two and three months after treatment. Thyroid scan(Tc99m scintigraphy) and thyroid ultrasonography reports were described.

**Results**: Among the 282 neonates included in this analysis, 51 were diagnosed with congenital hypothyroidism. The sex distribution was male 51% (26/51) vs. female 49% (25/51). Their initial postnatal ages of starting Levo-thyroxine treatment were 26.97 ± 15.4 days. 27 patients out of 51 were identified as TCH and 24 were revealed PCH. In the TCH patients group, male patients were 66.7% (18/27) and female patients were 33.3% (9/27). Ages of initial treatment of TCH were 31.15 ± 13.78 days. The sex distribution of PCH was male 33.3% (8/24) vs. female 66.7% (16/24). Ages of initial treatment of PCH patients were 27.93 ± 31.07 days. The mean duration of treatment in TCH group were 28.94 ± 13.89 months. Serum TSH levels were measured at diagnosis of PCH group (mean 150.49 , median 77.70 , 25- 75% 43.8-185 IU/mL) were significantly higher than those of TCH group (mean 30.29 , median 21.40 , 25-75% 17.1-291 IU/mL) (p<0.001). FT4 levels (PCH;0.73 ± 0.50ng/dL vs. TCH;1.17 ± 0.53ng/dL, p<0.02) and TT3 levels (PCH;1.36 ± 0.68ng/mL, vs. TCH;1.90 ± 0.35ng/mL, p<0.019) measured at start of treatment also showed significant differences. FT4 level measured at two months later from start of treatment in PCH group were significantly higher than TCH group (p<0.034). Required treatment doses were significantly different only that of 2-years of therapy. Thyroid USG were normal in 53.3% of patients with PCH. Comparably, those of all patients of TCH group were normal. We found another difference in Tc99m scintigraphy reports of both group. The sizes of thyroids in patients of PCH group were bigger than TCH group (p<0.033).

**Conclusion**: According to these data, we might consider initial measurements of serum TSH, FT4, T3 and size of thyroid as predictive factors in categorizing TCH from PCH.

## P1-5-6 Rapid generation of Oatp1c1KO mouse by the CRISPR/Cas9 system to serve as an animal model for thyroid hormone transport defect in humans when combined with Mct8 deficiency

### Hideyuki Iwayama^1^, Shuji Takada^2^, Okumura Akihisa^1^, Refetoff Samuel^3^

#### ^1^Aichi Medical University, Pediatrics; ^2^National Center for Child Health and Development, Systems BioMedicine; ^3^The University of Chicago, Medicine

**Introduction**: MCT8 gene mutations produce thyroid hormone (TH) deficiency in human brain, causing severe neuropsychomotor abnormalities not correctable with TH. Mice have two TH transporters, Mct8 and Oatp1c1, although human have only MCT8. Mct8 single knockout (KO) mouse did not show neuropsychomotor abnormalities. Therefore, the ideal animal model for Mct8 deficiency is Mct8 /Oatp1c1 double KO mouse. In this study, we generated Oatp1c1 KO mice by CRISPR/Cas9, the new powerful genome editing tool, to obtain Mct8 / Oatp1c1 double KO mice.

**Method**: To produce mutant mice, guide RNAs were designed by CRSPR direct program. The gRNAs used in this study were: Oatp1c1 gRNA1 5’–attcagcgcaacctgccggaggg-3’, gRNA2 5’-ggacggatatccgactgtaaagg-3’. Template DNAs for in vitro transcription were generated by PCR amplification of the ORFs of hCas9 and gRNAs from each plasmid using Prime STAR (TaKaRa). The PCR products were purified and used for the in vitro RNA synthesis using mMessage mMachine T7 kit. Mouse zygotes were obtained by mating superovulated wild type BDF1 females and wild type BDF1 males. RNAs of hCas9 and gRNA were mixed just before microinjection into the cytoplasm of zygotes and the injected embryos were incubated at 37 degree until they were transfeered into pseudopregnant females at the two-cell stage. Genomic DNA was extracted from the tail or ear tips of pups, and the genomic sequences around the gRNA target sites were PCR amplified and sequenced directly, or were cloned into the plasmid and sequenced. The electropherograms of each sequence were classified into four caegories: wild type (no mutation and no overlapping peaks), hetero-like mutation (double peaks, which started from the gRNA target site), minor mosaic-like mutation (one of the double peaks is dominant to the other) and Indels (Both alleles have the same insertion and/or deletion).

**Result**: We obtained 28 pups from surrogate mothers. One mouse died at day 33. Of 27 mice, 24 were males and 3 were females; 15 mice were classified into hetero-like mutation, 3 were minor mosaic-like mutation, 9 were Indels. One mouse was hard to be classified. No mouse was wild type. The mutation rate was 26/27 (96.3%). We will report the phenotype of Mct8 /Oatp1c1 double knokckout mice at the meeting.

**Conclusion**: CRISPR/Cas9 system can provide high efficient and rapid generation of KO mice.

## P1-5-7 Rapid growth and early metastasis of papillary thyroid carcinoma in an adolescent girl with Graves’ disease

### Kazuhiro Shimura^1,2^, Tomohiro Ishii^2^, Hironori Shibata^2^, Ken Hoshino^3^, Tatsuo Kuroda^3^, Kaori Kameyama^4^, Norisato Mitsutake^5^, Kiminori Sugino^6^, Jaeduk Yoshimura Noh^7^, Tomonobu Hasegawa^2^

#### ^1^Department of Pediatrics, Kawasaki Municipal Hospital; ^2^Department of Pediatrics, Keio University School of Medicine; ^3^Department of Pediatric Surgery, Keio University School of Medicine; ^4^Department of Pathology, Keio University School of Medicine; ^5^Department of Radiation Medical Sciences, Nagasaki University, Atomic Bomb Disease Institute; ^6^Department of Surgery, Ito Hospital; ^7^Department of Internal Medicine, Ito Hospital

## P1-5-8 Thyrotoxicosis longer than 5 weeks after 22 weeks of gestation is required for the development of central hypothyroidism due to maternal Graves’ disease

### Yukako Nakano, Masahiro Goto, Yukihiro Hasegawa

#### Division of Endocrinology and Metabolism, Tokyo Metropolitan Children Medical Center

**Introduction**: Central hypothyroidism (CH) in neonates born to mothers with poorly-controlled Graves’ disease (GD) was first reported in 1988. For the development of this type of CH (mGD-CH), the following factors must be present: (1) passage of maternal thyroid antibodies through the placenta; (2) negative feedback in the fetal pituitary-thyroid axis; (3) requisite duration of thyrotoxicosis in both the mother and the fetus.

**Objective**: To elucidate the minimum duration of maternal and fetal GD for the development of this disorder.

**Materials and Methods**: “Pubmed” was searched for clinical information on 20 cases of mGD-CH from 8 papers published between 1988 and 2010. All diagnoses were re-confirmed. Maternal thyrotoxicosis had been found either before pregnancy or during pregnancy except in 1 mother who was diagnosed on postpartum day 7. The diagnosis of mGD-CH (serum free T4 1.0 microgram/dL and TSH 7.5 mU/L) was made between postpartum day 7 and 425. Finally, the duration of maternal and fetal thyrotoxicosis was calculated on the assumption that conditions (1) and (2) were established by the gestational age of 22 weeks.

**Results**: The timing of the diagnosis of maternal GD during pregnancy ranged from the gestational age of 14 to 31 weeks. In the patient with the latest diagnosis of maternal GD during pregnancy at GW 31, the timing of the delivery was GW 37, suggesting that the duration of thyrotoxicosis for both the mother and fetus was 6 at least weeks. The timing of delivery varied from 27 to 41 weeks. In the most premature case at 27 weeks, the period of maternal and fetal thyrotoxicosis was 5 weeks, as calculated by subtracting 22 weeks from 27 weeks. The minimum period of thyrotoxicosis was thus estimated to be more than 5 weeks.

**Discussion**: Our assumption that factors (1) and (2) were established at GW 22 stemmed from the following facts: transplacental passage of TRAb has been reported at GW 21 (Mckenzie, et al. 1992); fetal GD presenting an undetectable TSH level and markedly elevated thyroxine has been documented at GW 20 (Racover, et al. 1999); intra-amniotic thyroxine therapy for fetal hypothyroidism was effective at GW 22. (Hanono, et al. 2009). In conclusion, thyrotoxicosis in both the mother and fetus after GW 22 for a duration of more than 5 weeks was required for the development of mGD-CH.

## P1-5-9 A successful switch experience from high-dose PTU to MMI on day 4 of Graves’ thyroid storm in a 14-year-old girl

### Hiroyuki Shinohara^1,2^, Atsushi Iwabuchi^2^, Akiko Yamada^2^, Tomomi Kai^2^, Takahiro Kido^2^, Yoshiaki Kato^2^, Tomohiro Kamoda^2,3^, Ryo Sumasaki^2^

#### ^1^Department of Pediatrics, Ibaraki Seinan Medical Center Hospital; ^2^Department of Pediatrics, Faculty of Medicine, University of Tsukuba; ^3^Department of Pediatrics, Ibaraki Prefectural Central Hospital

**Background**: The standard pharmacological treatment strategy of thyroid storm has just been published in 2016 Japan Society of Pediatric Endocrinology Guideline. Although thiamazole (MMI) is recommended to be used as the first choice in Graves’ disease, the effect of propylthiouracil (PTU) to block the conversion from T4 to T3 in peripheral tissues encourages clinicians to use against thyroid storm in very early phase, and switch to MMI later. Nevertheless, the optimal timing of the switch has not been established to date and recently, pediatric cases with thyroid storm are quite rare in developed countries.

**Case**: The patient was initially diagnosed as having Graves’ disease at age 8 years in a rural area of Philippines and prescribed with MMI. She quit hospital visiting soon after the diagnosis. At age 12 years, she continuingly had fatigue and palpitation. At age 14 years, the family moved to Japan. On the day of onset, she had a strong dyspnea and was unable to lie flat. She was brought to the tertiary care center to be diagnosed as thyroid storm and transferred to PICU in our hospital. The patient was fully conscious and orthopneic. Bilateral exophthalmos and diffuse goiter were noted. Both the fT3 and fT4 were beyond the measurable range. The patient was treated with dexamethasone 8mg, PTU 1,200mg and iodine 200mg. Four days after the treatment, fT3 was decreased to the measurable range (8.0pg/mL). We immediately switched PTU to MMI 80mg. On 6th day, we reduced the dose of MMI into 60mg, switched dexamethasone to hydrocortisone. On 11th day, the symptoms and test results further improved and we reduced MMI into 30mg along with introduction of levothyroxine to prevent hypothyroidism and suspended iodine. On day 16, the patient was discharged from the hospital.

**Discussions**: In this case, high dose PTU rapidly suppressed fT3 w ithin 4 days. Free T3 drop to measurable range can be a good indicator of optimal timing of switching PTU to MMI. Relatively higher dose of MMI as 80mg might be required to keep suppressing the thyroid just after the switch.

**Consent for publication:** The authors declare that written informed consent was obtained for publication.

**Fig. 1 (abstract P1-5-9).**
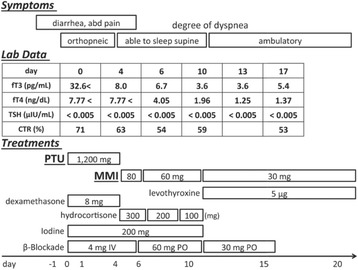
See text for description

## P1-5-10 Two adolescent cases of Moyamoya disease concurrent with Graves’ disease

### Ikue Hata^1^, Yuko Isozaki^1^, Masao Kawatani^1^, Hisako Hayashi^3^, Miori Yuasa^1^, Kenichiro Kikuta^2^, Yosuke Shigematsu^1^

#### ^1^Department of Pediatrics, Faculty of Medical Sciences, University of Fukui; ^2^Department of Neurosurgery, Faculty of Medical Sciences, University of Fukui; ^3^Department of Pediatrics, Fukui Prefectural children's rehabilitation center

## P1-5-11 Hyperthyroidism after Bone Marrow Transplantation: A Report of Two Cases

### Hiroyuki Ishiguro^1,2^, Hiromi Hyodo^2,3^, Shunichi Kato^4^

#### ^1^Department of Pediatrics, Isehara Kyodo Hospital; ^2^Department of Pediatrics, Tokai University School of Medicine; ^3^Department of Pediatrics, Japanese Red Cross Hadano Hospital; ^4^Department of Cell Transplantation & Regenerative Medicine, Tokai University School of Medicine

**Background**: The thyroid dysfunction is one of the frequently seen complications after bone marrow transplantation (BMT). Although hypothyroidism is the most common thyroid disease after BMT, hyperthyroidism is a rare condition. Herein, we report a series of 2 patients who were euthyroid before BMT but developed hyperthyroidism after transplantation.

**Objective**: Case reports and the frequency of hyperthyroidism after BMT in our institute.

**Case 1**: A 10-year-old boy was diagnosed with adrenoleukodystrophy and underwent transplantation twice from his HLA-unmatched sister when 10 years of age. Conditioning regimens consisted of thoracoabdominal irradiation + Busulfan (Bu) + Cyclophosphamide (CY) + Antithymocyte globulin (ATG) for the first BMT and Bu + CY + ATG for the second BMT due to a rejection of the first BMT. At the time of both BMTs, his thyroid function tests were normal, respectively, and his donor had no history of thyroid disease. Prophylaxis against graft-versus- host disease (GVHD) was used short-term methotrexate (sMTX) and cyclosporine (CyA). He had acute GVHD presenting with nodular rash and prednisolone was initiated. Although subclinical compensated hypothyroidism was demonstrated after the first BMT, hyperthyroidism occurred 3 years after the first BMT. He was treated with methimazole.

**Case 2**: A 15-year-old boy was diagnosed with severe aplastic anemia and underwent transplantation from his HLA-matched sister when 15 years of age. Conditioning regimens consisted of CY + ATG. Prophylaxis against GVHD was used sMTX and CyA. He had no acute and chronic GVHD. Hyperthyroidism occurred 3 years after BMT. After he was followed without treatment for 9 months, we started to treat with methimazole.

**Results**: Systematic evaluation of thyroid function tests in 156 who underwent BMT and are follow-up at our institute gave a rate of hyperthyroidism at 1.3% in this study.

**Conclusion**: The clinician should be alert to hyperthyroidism as a possible complication after BMT.

## P1-5-12 Central hypothyroidism secondary to maternal hyperthyroidism - a case series from a Children's Hospital

### Song Hai Lim, Sheng Chai Tan

#### Department of Paediatrics, Sabah Women and Children's Hospital

**Introduction**: Maternal hyperthyroidism can lead to variable thyroid abnormalities found in the babies, with potential detrimental outcomes. Central hypothyroidism (CH) is one of the rarer form of thyroid dysfunction described in this entity.

**Aim**: To describe a case series of central hypothyroidism found in babies born to mothers with hyperthyroidism from a Children’s Hospital.

**Method**: All cases of CH resulted from maternal hyperthyroidism being followed up in a paediatric endocrine clinic from 1 January 2014 to 31 May 2016 were retrieved. Their clinical features and progress of illness were summarised. Diagnosis is based on low free thyroxine (fT4) concentration associated with inappropriately normal or low Thyroid Stimulating Hormone (TSH) level.

**Results**: There are total eight cases identified, including a pair of twins. The twins were born small and prematurely, another two babies were born prematurely with appropriate size, the rest were born at term with appropriate weight. For maternal diagnoses, there were four cases of hyperthyroidism, presumptively Graves’ disease, and the rest are toxic thyroid nodule, multinodular goiter and subclinical hyperthyroidism. Evidence of CH developed immediately after birth in two cases, two to six weeks later after a transient period of euthyroidism in five cases, and after 3 months of hyperthyroidism which required Carbimazole treatment in the last case. Poorer control of maternal hyperthyroidism was found in the three babies who developed immediate CH and initial hyperthyroidism. The mean duration of follow-ups was 10.6 ± 8.3 months. All were still dependent on thyroxine, with a mean dosage of 4.2 ± 1.4 mcg/kg/day. Nevertheless, two cases had shown partial recovery of TSH from 13 and 19 months respectively. Developmental milestones were normal for all cases, except slight gross motor delay for the case with initial hyperthyroidism. Thyrotropin releasing hormone stimulation test to confirm the CH was not done due to limited resource.

**Conclusion**: The relatively high number of this cohort from a single centre warrants a further evaluation of the real prevalence of CH secondary to maternal hyperthyroidism. Careful monitoring of neonatal thyroid function born to these mothers is important, as CH will not be detected by TSH-based screening method and may developed after a period of euthyroidism.

## P1-5-13 Neonatal thyroid dysfunction born from mothers with thyroid disease

### Sung Won Park^1^, Young Bae Sohn^2^, Sung Yoon Cho^3^, Su Jin Kim^4^

#### ^1^Department of Pediatrics, Cheil Hospital, Dankook University College of Medicine; ^2^Department of Medical Genetics, Ajou University Hospital; ^3^Department of Pediatrics, Samsung Medical Center, Sungkyunkwan University School of Medicine; ^4^Department of Pediatrics, Myongji Hospital, Seonam University College of Medicine

**Aim**: To evaluation of thyroid function in neonates born from mothers affected by thyroid disease in order to define follow-up thyroid function test is needed for these children.

**Methods**: Records were reviewed for all newborn with thyroid dysfunction of mothers who were born in Cheill General Hospital, from May, 2012 to May, 2015. All neonates were tested for thyroid function by measurement of thyroxine (T4) and thyroid stimulating hormone (TSH) in 3rd or 4th day. Babies whose first results was abnormal or who were born from mothers affected by thyroid disease were tested for thyroid function repeatedly by measurement of free thyroxine (fT4) and TSH at about 2weeks and at one month of life. Data concerning etiology of maternal thyroid disease during pregnancy were retrospectively collected. Neonates who were born from women with thyroid disease and having anti-thyroglobulin (ATG) or anti-microsomal (AMA) antibodies were assigned as group I, women with hypothyroidism who did not have autoantibodies were assigned as group II, and women without thyroid problems were assigned as group III.

**Results**: In group I, 12(6.1%) infants were diagnosed with compensated hypothyroidism and started levothyroxine therapy. The median age of diagnosis of hypothyroidism was 37.4 days. 20(10.2%) infants had hyperthyrotropinemia at 1st test. 24(12.2%) infants had transient hyperthyrotropinemia. The median period of normalization of TSH was 112.7 days. Especially, 5 neonates were diagnosed with hypothyroidism, despite of normal results of 1st screening test. 35 infants (9.7%) in group II had hyperthyrotropinemia at 1st test. 9 (2.5%) of them were diagnosed with compensated hypothyroidism and started levothyroxine therapy. The median age of diagnosis of hypothyroidism was 70.4 days. 64 (17.7%) infants had transient hyperthyrotropinemia. The median period of normalization of TSH was 117.5 days. Especially, 3 neonates were diagnosed with hypothyroidism, despite of normal results of 1st screening test. In group III, 468 infants had hyperthyrotropinemia at the 1st test and a spontaneous completely normalization of TSH value was observed. 47 of them were diagnosed with compensated hypothyroidism and started levothyroxine therapy.

**Conclusion**: Persistent hyperthyrotropinemia requiring replacement therapy is observed in 6.1% of group I, 2.5 % of group II. According to our experience, follow-up is recommended in these newbornsNewborn infants born from women with thyroid disorders should be followed closely for thyroid dysfunction.

## P1-5-14 The effect of levothyroxine on thyroid volume in euthyroid children with autoimmune thyroiditis. A systematic review of clinical trials

### Annang Giri Moelyo^2^, Bambang Tridjaja^1^, Indah Suci Widyahening^3^

#### ^1^Faculty of Medicine, Division of Endocrinology / Department of Pediatrics, University of Indonesia; ^2^Faculty of Medicine, Division of Endocrinology/ Department of Pediatrics, Sebelas Maret University; ^3^Faculty of Medicine, Division of Community Medicine, University of Indonesia

**Background**: Children with autoimmune thyroiditis may manifest as overt hypothyroidism, subclinical hypothyroidism, euthyroid or hyperthyroidism. Although there is no consensus on treating euthyroid condition in thyroiditis autoimmune children, some studies showed efficacy of levothyroxine in decreasing thyroid volume, improving thyroid function, and stabilizing immunological process.

**Objectives**: to determine the effect of levothyroxine on thyroid volume changes, thyroid functions and thyroid antibodies in euthyroid children with autoimmune thyroiditis.

**Methods**: Systematic review was performed in euthyroid children with autoimmune thyroiditis. Electronic databases search (the Cochrane Library, MEDLINE, EBSCO, Proquest, clinicaltrials.gov and other sources) and non-electronic search (handsearch of journals, conference proceeding) were done. Randomised and quasi-randomised controlled trials comparing levothyroxine to control in all euthyroid children with autoimmune thyroiditis were selected. Two person independently extracted data, assessed the risk of bias, analyzed pooled data from included study by random effect model and performed sensitivity analysis.

**Results**: Two studies (80 participants) were included from 48 studies identified from searching methods. Only thyroid volume SDS data could be aggregated. Mean difference of thyroid volume change was -1,18 (95%CI; -1,65; -0,70) SDS between levothyroxine and control. Mean difference of TSH, fT4, TPOAb and TgAb change between levothyroxine and control were -0,24 mU/liter (IK95% -1,96; 1,12); -0,76 pmol/liter (95%CI -4,77; 3,25); 135,2 U/ml (95%CI -319,8; 590,2); and -75,53 U/mililiter (95%CI -255,32; 104,26). Risk of goiter was decreasing with levothyroxine treatment (OR 0,06; IK95% 0,01-0,28).

**Conclusion**: Levothyroxine treatment for euthyroid children with autoimmune thyroid might reduce thyroid volume (SDS) and risk of goiter.

Treatment of levothyroxine do not conclusively improve changes of TSH, fT4, TPOAb and TgAb.

**Keyword**: autoimmune thyroiditis, euthyroid, levothyroxine, thyroid volume

**Fig. 1 (abstract P1-5-14).**
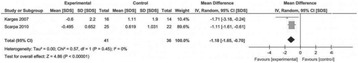
The effect of levothyroxine on thyroid volume SDS in euthyroid children with autoimmune thyroiditis

## P1-5-15 Does PTEN tumor suppressor gene responsible for papillary thyroid carcinoma in a 14 years old adolescence: a case report

### Frida Soesanti, Bambang Tridjaja, Jose RL Batubara, Aman Pulungan

#### Division of Endocrinology/Department of Paediatrics, Faculty of Medicine Universitas Indonesia- Cipto Mangunkusumo Hospital, Jakarta, Indonesia

**Introduction**: Thyroid cancer is one of the most common causes of malignancy in childhood, accounting for 0.5%-3% of all childhood malignancy. Radiation exposure has been established as one of the risk factors to develop thyroid cancer; but there are also several genes associated with development of thyroid cancer such as RET/TRK, BRAF, RET/PTC, PTEN tumor suppressor gene, and GNAS-1 gene mutation.

**Case**: A-14 years old male adolescents referred to our clinic due to left thyroid enlargement since a year prior with no signs and symptoms of hypothyroidism or hyperthyroidism. No weight reduction was found. The first thyroid ultrasound revealed diffuse enlargement of the left thyroid lobe and the hormonal assay showed normal FT4 and TSHs level. He received L-thyroxin treatment for one year with no reduction in goiter size. They were high incidence of malignancy running through his family .i.e. thyroid malignancy, breast malignancy, and lymphoma. His second US examination revealed left lobe enlargement with the size of 7.3 x 6.4 x 4.9 cm with multiple hyperechoic and hypoechoic nodules. Anti-TPO and anti thyroglobulin were negative. His thyroid scan showed multiple cold nodules. He then underwent FNAB which appropriate with benign adenomatous goiter. He continued to received L-thyroxin treatment for the next 6 months, but loss to follow up for 5 months and returned with further enlarge thyroid. CT-Scan of the thyroid revealed diffuse enlargement of left lobe with the size of 8.8 x 5.9 x 6.3 cm with necrotic spots. No lymph nodes enlargement found. Right thyroid lobe was normal. He underwent the second FNAB with the result of atypical cell of undetermined significance and was advised to do FNAB within in the next three months. The third FNAB revealed carcinoma thyroid. He then underwent total thyroidectomy and result of thyroid biopsy was appropriate with papillary thyroid carcinoma with solid and follicular variant.

**Conclusions**: This patient showed an evolution from benign thyroid nodule to thyroid carcinoma in an adolescence with time span of one and half years. High incidence of malignancy in his family raise suspicion of the possibility of genetic predispotition. PTEN gene is one of the gene that should be consider in this patients considering the occurrence of thyroid, breast and lymph node malignancy in his family. Further studies and collaborations with other centers are needed to support this hypothesis.

## P1-5-16 The role of maternal TSH receptor antibodies in the pathogenesis of neonatal thyroid disorders

### Eva Al Taji, Olga Hnikova

#### Department of Pediatrics, 3rd Faculty of Medicine, Charles University

**Background**: The transplacental passage of maternal thyrotropin receptor (TSHR) antibodies in the case of maternal autoimmune thyroid disease influences the function of fetal and neonatal thyroid gland. The clinical picture depends on the functional activity of TSHR antibodies

their ability to inhibit (typically in maternal autoimmune atrophic thyroiditis) or activate (in maternal Graves-Basedow thyrotoxicosis) TSH receptor.

**Objective**: It has been described previously that maternal autoantibodies binding to TSHR can cause fetal and neonatal hyperthyroidism or congenital hypothyroidism. The remission of this condition can be expected in 3-6 months with disappearing of antibodies.

**Case reports:** We have followed three girls with congenital hypothyroidism and two patients with neonatal hyperthyroidism with high levels of TSHR antibodies at diagnosis. The cases of congenital hypothyroidism were diagnosed in nation-wide neonatal screening based on high levels of TSH. Their mothers were treated for chronic lymphocytic thyroiditis. Two neonatates with severe neonatal thyrotoxicosis were diagnosed based on clinical symptoms in neonatal period. One mother had undiagnosed Graves-Basedow thyrotoxicosis, the second underwent thyroidectomy for the same condition prior to pregnancy.

**Conclusions**: It is recommended to follow levels of TSHR antibodies in pregnant women with active Graves-Basedow disease but also in those who underwent thyroidectomy or radioiodine therapy for this condition previously as antibodies may persist. Depending on maternal TSHR antibodies levels, the close follow up of the fetus and neonate can be required. Even if transplacental passage of TSHR antibodies is not a frequent cause of congenital hypothyroidism, we perform TSHR antibodies testing in all newborns with congenital hypothyroidism and also in their mothers.

## P1-6-1 Identical NR5A1 Missense Mutations in Two Unrelated 46,XX Individuals with Testicular Tissues

### Maki Igarashi^1^, Kei Takasawa^2^, Akiko Hakoda^3^, Junko Kanno^3^, Shuji Takada^4^, Mami Miyado^1^, Toshihiro Tajima^5^, Ryohei Sekido^6^, Tsutomu Ogata^1,7^, Kenichi Kashimada^2^, Maki Fukami^1^

#### ^1^Departments of Molecular Endocrinology, National Research Institute for Child Health and Development; ^2^Department of Pediatrics and Developmental Biology, Tokyo Medical and Dental University (TMDU); ^3^Department of Endocrinology, Miyagi Children's Hospital; ^4^National Research Institute for Child Health and Development, Systems BioMedicine; ^5^Department of Pediatrics, Hokkaido University School of Medicine; ^6^University of Aberdeen, Institute of Medical Sciences; ^7^Department of Pediatrics, Hamamatsu University School of Medicine

The genetic basis of SRY -negative 46,XX testicular/ovotesticular disorders of sex development (DSD) remains largely unknown. Although mutations in NR5A1 are known to cause 46,XY gonadal dysgenesis and 46,XX ovarian insufficiency, this gene has not been implicated in testicular development of 46,XX gonads.

We performed sequence analysis of 28 genes known to be involved in gonadal development in 8 patients with SRY -negative 46,XX testicular/ ovotesticular DSD. Nucleotide alterations were screened by next generation sequencing and confirmed by Sanger sequencing. As a result, we detected identical NR5A1 mutations (p.R92W) in two unrelated patients. These patients carried no additional mutations in tested genes. The patients had testicular or ovotesticular tissues in the gonads and lacked uterus or vagina. The NR5A1 mutation was absent in the clinically normal mothers of the patients. The mutation was not found in exome databases or in 200 unaffected control individuals. In silico analyses suggested that the p.R92W is probably pathogenic and induces conformational changes at the DNA-binding site. In vitro assays demonstrated that, compared with wildtype NR5A1, the mutant protein was less sensitive to NR0B1 induced suppression on the SOX9 enhancer sequence core element. Our findings provide the first indication that NR5A1 is a causative gene for 46,XX testicular/ ovotesticular DSD.

## P1-6-2 Long-term clinical course in three patients with MAMLD1 mutations

### Yasuko Fujisawa^1^, Maki Fukami^2^, Tomonobu Hasegawa^3^, Ayumi Uematsu^4^, Koji Muroya^5^, Tsutomu Ogata^1^

#### ^1^Department of Pediatrics, Hamamatsu University School of Medicine; ^2^Department of Molecular Endocrinolog, National Research Institute for Child Health and Development; ^3^Department of Pediatrics, Keio University School of Medicine; ^4^Department of Endocrinology and Metabolism Unit, Shizuoka Children's Hospital, Shizuoka; ^5^Department of Endocrinology and Metabolism, Kanagawa Children's Medical Center

**Introduction**: MAMLD1 on chromosome Xq28 is known as a causative gene for 46,XY disorders of sex development; however, clinical information is virtually limited in patients of infancy to early childhood. Here, we report long-term genital and hormonal findings in three previously described Japanese patients with MAMLD1 mutations.

**Subjects**: We report 3 parients; patients 1 and 2 with p.E197X and patient 3 with p.R726X. Patients 1–3 exhibited penoscrotal hypospadias with chordee, microphallus, bifid/hypoplastic scrotum, and/or bilateral cryptorchidism/retractile testes.

**Course**: In the mini-pubartal period to early childhood, all patients showed sufficiently high serum basal or hCG-stimulated testosterone values. Subsequently, patient 1 had low serum hCG-stimulated testosterone value (126 ng/dL) at 13 11/12 years of age, and manifested microphallus (4.5 cm), relatively small testes (left 8 mL and right 10 mL), Tanner stage 3 genitalia and pubic hair development at 18 3/12 years of age. Similarly, patients 2 and 3 showed mild hypergonadotropic hypogonadism at 7 0/12 and 9 9/12 years of age, respectively, with serum GnRH-stimulated LH values of 5.5 and 7.2 mIU/mL and'sH values of 10.3 and 19.8 mIU/mL and hCG-stimulated testosterone values of 70 and 80 ng/dL, respectively. Testis ultrasound studies delineated microlithiasis in patients 1 and 3.

**Conclusion**: These results imply for the first time deterioration of testicular function with age in patients with pathologic MAMLD1 mutations.

## P1-6-3 Characterization of androgen production in a genetic male infant with prenatally diagnosed POR deficiency

### Hiroyuki Ono^1^, Chikahiko Numakura^2^, Seiji Tsutsumi^3^, Keiko Honma^4^, Tomonobu Hasegawa^5^, Fumiko Kato^1^, Tsutomu Ogata^1^

#### ^1^Department of Pediatrics, Hamamatsu University School of Medicine; ^2^Department of Pediatrics, Yamagata University; ^3^Department of Obstetrics and Gynecology, Yamagata University; ^4^Department of Laboratory Medicine, Keio University; ^5^Department of Pediatrics, Keio University

**Context**: In male fetuses, androgens could be produced in three tissues: (1) the fetal testis that would produce the frontdoor-derived testosterone (T) (which is further converted to dihydrotestosterone (DHT) in the external genital tissues) and the backdoor-derived DHT; (2) the placenta that produces T from DHEA, and (3) the fetal adrenal that could produce the backdoor-derived DHT in co-operation with permanent adrenal. In this regard, previous studies in genetic males with P450 oxidoreductase (POR) deficiency (PORD) have suggested that testicular T and DHT production is compromised, whereas placental T production and the fetal adrenal-related DHT production may be exaggerated in PORD. To examine this notion, we studied the androgen production properties in a genetic male infant with prenatally diagnosed PORD.

**Patient**: This Japanese genetic male infant was prenatally diagnosed as having PORD, on the basis of severely undermasculinized external genitalia (he was initially regarded as a female), radio-humeral synostosis on computed tomography, and maternal virilization. Shortly after birth, chromosome analysis showed a 46,XY karyotype, and direct sequencing revealed compound heterozygous mutations of POR (c.601C>T, p.Q201X in exon 6 and c.1370G>A, p.R457H in exon 12).

**Methods**: Steroid metabolites were measured by LC-MS/MS, using longitudinal blood samples of this patient and umbilical blood samples from control males (n=5) and females (n=5).

**Results**: 17-hydroxyprogesterone (17-OHP4), androstenedione (A-dione), T, and DHT values showed transient marked elevation around birth (at birth and one day of age) in this patient as compared with controls. However, the A-dione/T and T/DHT ratios were comparable between the patient and controls at that time, and the backdoor-derived 5 α -androstane,3α,17 β -diol (5α,3α-A-diol) value was comparable between the patient and the controls. In addition, T and DHT were higher in control males than in control females, and other steroid metabolites including those on the backdoor pathway were comparable between male and female controls.

**Discussion**: These results imply that elevated T and DHT around birth of this patient is primarily derived from the placenta, and that the relevance of the backdoor pathway to the elevated DHT remains minor, if any. In addition, the backdoor pathway is unlikely to be operating in the fetal testis around the birth.

## P1-6-4 The relationship between clinical phenotypes and mutations of MAMLD1 in children with hypospadias

### Yongfen Lyu, Pin Li

#### Division of Endocrinology and Metabolism, Shanghai Children's Hospital

**Objective**: To verify the relationship between clinical phenotypes of hypospadias and mutations of MAMLD1.

**Methods**: Seventy-two patients were diagnosed to be hypospadias in department of endocrinology and department of urinary surgery in our hospital. Among all the patients, 69 were with normal karyotype and enrolled as the studied group. Fifty healthy boys were employed as the controls. Peripheral Blood were collected for DNA extraction. For the studied group, PCR primer was designed and direct sequencing was performed for screening for MAMLD1 mutations in six coding exons and the flanking region. Those mutated exons were examined for the control group.

**Results**: Thirty-five of all the 72 patients (48.6 %) were isolated hypospadias. The other 37 cases (51.4%) were complicated by other genitourinary system malformations, including 12 cases with micropenis and/or underdeveloped testicles. Abnormal karyotype was identified in 3 patients, and all were karyotype as 46, XX (SRY+ in 1 case and SRY- in 2 cases). Six types of MAMLD1 mutations were detected in exon 2, 3, 5, 7 in studied group, including c.5A G (p.D2G), IVS4-364C/A, c.1910A G ip.N637S), c.2208T C, c.2227 G A (p.E742K) and IVS8-144C/T. All were single nucleotide polymorphism except c.5A G (p.D2G), a newly discovered point mutation. The frequency of IVS4-364C/A was significantly different between patients and controls, and it was also significantly different between patients with and without micropenis and/or underdeveloped testicles.

**Conclusion**: Chromosome abnormality is not the leading cause of other genitourinary system malformations complicated with hypospadias. Mutations of MAMLD1 maybe closely related to hypospadias in Chinese. c.5A G ip.D2G ) is the newly discovered point mutation in this work. IVS4-364C/A is associated with underdeveloped testicles and/or micropenis in hypospadias patients.

## P1-6-5 Genetic knockout of Mamld1 reduces testicular size but permits normal fertility in adult male mice

### Mami Miyado^1^, Kaoru Yoshida^2^, Kenji Miyado^3^, Katsumi Momori^1,4^, Kazuki Saito^1,5^, Shigeru Nakamura^1,6^, Tsutomu Ogata^1,7^, Maki Fukami^1^

#### ^1^Department of Molecular Endocrinology, National Research Institute of Child Health and Development; ^2^Toin University of Yokohama, Biomedical Engineering Center; ^3^Department of Reproductive Biology, National Research Institute of Child Health and Development; ^4^Department of NCCHD Child Health and Development, Graduate School, Tokyo Medical and Dental University; ^5^Department of Comprehensive Reproductive Medicine, Graduate School, Tokyo Medical and Dental University; ^6^Department of Pediatric Urology, Jichi Medical University, Children's Medical Center Tochigi; ^7^Department of Pediatrics, Hamamatsu University School of Medicine

**Background**: Mastermind-like domain containing 1 (MAMLD1 ) on the human X chromosome is a causative gene for 46,XY disorders of sex development. Recently, progressive deterioration of testicular function was documented in three boys with loss-of-function MAMLD1 mutations. However, the function of MAMLD1 in adult males has not been investigated.

**Objective**: To clarify the role of MAMLD1 in the reproductive function of adult male mice.

**Materials and Methods:** We analyzed phenotypic characteristics of adult male mice lacking Mamld1 (KO). Testes were obtained from 8 week-old wild-type (WT) and KO mice, and subjected to histological and histochemical analyses. Sperm were analyzed using computer-assisted semen analysis system and microscope. Serum steroid metabolites were measured by liquid chromatography-tandem mass spectrometry. WT and KO male mice were mated with WT female mice and the number of pups was counted on the day of delivery.

**Results**: At 8 weeks of age, Mamld1 KO male mice exhibited no discernible abnormalities, except for reduced testicular size. Testes of KO mice showed reduced diameter of seminiferous tubules and decreased number of mitotic cells stained by anti-proliferating cell nuclear antigen antibody. Daily sperm production was impaired in KO mice, although epididymal sperm count was comparable between WT and KO animals. Sperm collected from the epididymis of KO mice showed normal morphology, motility, and in-vitro-fertilizing ability. Furthermore, blood testosterone levels and the number of pups were comparable between WT and KO male mice.

**Conclusions**: These results indicate that MAMLD1 is involved in postnatal testicular development and sperm production, however is dispensable to maintain male fertility in mice.

## P1-6-6 NR0B1 Frameshift Mutation in a Boy with Precocious Puberty and Normal Adrenal Function

### Hirohito Shima^1,2^, Shuichi Yatsuga^3^, Akie Nakamura^1^, Shinichiro Sano^1^, Takako Sasaki^3^, Noriyuki Katsumata^1^, Erina Suzuki^1^, Tsutomu Ogata^4^, Satoshi Narumi^1^, Maki Fukami^1^

#### ^1^Department of Molecular Endocrinology, National Research Institute for Child Health and Development; ^2^Department of Advanced Pediatric Medicine, Tohoku University School of Medicine; ^3^Department of Pediatrics and Child Health, Kurume University School of Medicine; ^4^Department of Pediatrics, Hamamatsu University School of Medicine

**Background**: Inactivating mutations in NR0B1 , a gene encoding DAX1, cause X-linked adrenal hypoplasia congenita. While most NR0B1 mutations lead to adrenal crisis during infancy of early childhood, p.Gln37*, p.Trp39*, and certain other mutations result in late-onset or latent adrenal insufficiency. A small fraction of boys with NR0B1 mutations develop precocious puberty in addition to adrenal insufficiency. Objective: To report a boy with an NR0B1 mutation who exhibited central precocious puberty without adrenal insufficiency.

**Case report**: At 4 years of age, the patient showed signs of puberty: pubic hair of Tanner stage 3, external genitalia of Tanner stage 4, and testes of 6 mL in size. He was tall (117 cm, +3.2 SD) and had advanced bone age (8.5 years). Laboratory examination detected elevated serum levels of testosterone and gonadotropins and hyperresponses of gonadotropins to GnRH stimulation. Blood electrolyte and adrenal hormone levels were within normal ranges. Brain magnetic resonance imaging detected no abnormalities. The patient was diagnosed with idiopathic central precocious puberty. Treatment with a GnRH analog partially ameliorated the hormonal abnormalities, but did not improve the physical symptoms. On his latest visit at 7 years and 6 months of age, the patient showed no clinical signs of adrenal insufficiency. Blood levels of ACTH and cortisol were normal.

**Molecular analysis**: The patient was subjected to mutation screening of 32 genes known to control the hypothalamic-pituitary-gonadal axis. The analysis identified a maternally inherited hemizygous 1-bp deletion in exon 1 (p.Glu3fsAla*16) of NR0B1 . The mutation was predicted to generate an N-terminally truncated hypomorphic protein, similar to that produced by p.Gln37*and p.Trp39*. No pathogenic mutations were found in other tested genes.

**Conclusions**: We identified an NR0B1 frameshift mutation in a boy clinically diagnosed with idiopathic central precocious puberty. The results expand the clinical manifestations of NR0B1 mutations to include male central precocious puberty without adrenal insufficiency.

NR0B1 mutations likely underlie testosterone overproduction through both GnRH-dependent and -independent mechanisms.

**Consent for publication:** The authors declare that written informed consent for study enrollment including publication was obtained from the parents of the patient.

## P1-6-7 Effect of intramuscular testosterone enanthate treatment on penile length in boys with micropenis

### Kenichi Kinjo^1^, Yasuko Fujisawa^2^, Yohei Masunaga^2^, Hiroyuki Ono^2^, Kounosuke Ohtaka^2^, Shinichi Nakashima^2^, Hirokazu Saegusa^1^, Tsutomu Ogata^2^

#### ^1^Department of Pediatrics, JA Shizuoka Koseren Enshu Hospital; ^2^Department of Pediatrics, Hamamatsu University School of Medicine

**Background**: Micropenis is a heterogeneous condition defined as significantly small penis (stretched penile length [SPL] below − 2.5 SD of the mean in age-matched control boys) which is free from associated external genital ambiguity such as hypospadias. Intramuscular testosterone enanthate (TE) has widely been utilized in boys with micropenis, to increase the penile length.

**Objective**: The purpose of this study was to examine the effect of TE treatment on SPL in Japanese patients with micropenis.

**Methods**: We retrospectively examined 48 Japanese patients with micropenis aged 3–65 months (median 10 months) who were seen at our University Hospital between the years 2012–2015. The SD score was calculated using the reference values for SPL in Japanese boys (Ishii, 2014). All cases were treated with TE (25 mg injection) for 1–5 times (median 2 times; mean 2.13 times).

**Results**: The first treatment increased the SPL by 0.05–2.00 cm (median 0.50 cm) in the 48 patients, with the Δ SDS of 0.05–11.77 (median 1.49). After entire course of treatments the SPLs increased from 1.00–3.30 cm (median 2.5 cm) to 3.00–4.00 cm (median 3.5 cm). A single patient with an extremely small penis who had defective testosterone production because of severely hypoplastic testes showed a marked response to TE (SPL, from 1.00 cm to 3.50 cm; SPL-SDS; from − 13.19 to − 0.27). After excluding this particular patient, the 47 patients were divided into two groups on the basis of the SPL responses to the first TE treatment; (1) good responders whose SPLs were increased by 0.5 cm (n = 29), and (2) poor responders whose SPLs increased by < 0.5 cm (n = 18). Subsequent TE treatment was performed more frequently in poor responders than in good responders (1–5 times, median 2 times, mean 2.7 vs. 1–4 times, median 2 times, mean 1.8 times). After such treatment, the SPLs became 3.54 cm ( − 0.78 SDS) in good responders and 3.45 cm ( − 0.95 SDS) in poor responders (P = 0.47).

**Discussion**: The effect of the first injection on SPL is compatible with previous reports. In all cases, SPLs after treatments reached above 3 cm that is a satisfying length for standing urination. No apparent adverse effects were observed.

**Conclusion**: TE therapy is favorable and well-tolerated treatment for micropenis.

## P1-6-8 A novel mutation of KISS1R causing a normosmic isolated hypogonadotropic hypogonadism

### Keisuke Yoshii^1,2^, Yasuhiro Naiki^1^, Reiko Horikawa^1^

#### ^1^Division of Endocrinology and Metabolism, National Center for Child Health and Development; ^2^Hôspital Robert Debré, Laboratoire de Biochimie Inserm U1141

**Background**: Kisspeptin receptor, which is also called as GPR54 and encoded by KISS1R, is a G-protein-coupled receptor expressed in GnRH neurons. Its ligand kisspeptin is known as strong regulator of GnRH secretion. Loss of function mutations in KISS1R have been reported in very few patients with normosmic isolated hypogonadotropic hypogonadism (nIHH). Objective: To describe the phenotype of the nIHH female patient with a novel homozygous KISS1R mutation and to characterize functionally this mutation. The patient was a 28 year-old Senegalese woman with primary amenorrhea. She was the second child born to consanguineous parents. Her height was 182 cm, her weight 55 kg. Breast development was scored Tanner stage 2, pubic and axillary hair growth could not be evaluated because of depilation. She had no evidence with hyposmia. Hormone assays revealed low levels of estradiol and inappropriate normal levels of gonadotropins. Brain MRI showed normal pituitary and olfactory bulbs. Pelvic ultrasonography showed a small uterus and right small ovary with follicles. The left ovary was not visualized. Her karyotype was 46,XX.

**Method**: Exon and exon-intron boundaries of nIHH candidate genes (KISS1 , KISS1R , TAC3 TACR3 , GNRH1 , GNRHR ) were sequenced. Functional analysis of the mutated receptor was performed by intracellular inositol phosphate measurement, western blot and immunofluorescence staining in heterologous cells.

**Results**: A novel homozygous mutation c.953T>C was identified in the proband, leading to the amino acid substitution of leucine 318 by proline (p.L318P). Functional analysis showed impaired inositol phosphate generation under kisspeptin stimulation. The mutated receptor was not detected at the cell surface in transfected HEK 293 cells. Western blot analysis showed the absence of mature glycosylated receptor but the presence of an immature form.

**Conclusion**: The nIHH observed in this patient is due to a novel loss-of-function mutation in KISS1R . The L318P substitution impedes the intracellular trafficking of KiSSR. This patient is a novel candidate to a treatment by a chemical chaperone to rescue expression of the mutated receptor at the cell surface.

## P1-6-9 Metformin attenuated lipid metabolism and glycometabolism, insulin resistance and aromatase in rats with sexual precosity

### Shanshan Zhang

#### General Hospital, Tianjin Medical UniversityDepartment of Pediatrics

**Objective**: To investigate the effects of metformin on the glycometabolism, lipid metabolism, and insulin resistance, as well as estrogen level and aromatase expression in the adipose tissues.

**Methods**: Rats were fed on a high fat diet to induce insulin resistance and sexual precosity. The animals were randomly divided into the following groups, including normal control (n=8), high fat group (n=8), low dose of metformin group (n=8), and high dose of metformin group (n=8). Fasting insulin (FINS) in serum was determined using commercial radioimmunoassay kit. The serum estradiol (E2), total cholesterol (TC), triglyceride (TG), low density lipoprotein (LDL) and high density lipoprotein (HDL) were determined using the Glamour 1600 automatic biochemistry analyzer (Metrolab, Argentina). Real-Time PCR was carried out to determine the expression of Cyp19a1 mRNA in the kidney and ovary tissues. Western blotting analysis was performed to determine the aromatase expression.

**Results**: At week 6, the levels of FINS, FPG, HOMA-IR, E2, TC, TG, and LDL were remarkably elevated in the high fat group compared with the normal control, while the level of HDL in the model group was obviously lower than that of the normal control. The expression of aromatase in the high fat group was about 1.5-fold increase compared with that of the control group (P<0.05). Compared with the high fat group, metformin could remarkably down-regulated the expression of aromatase, especially the animals subjected to high dose of aromatase. Compared with normal control, the expression of Cyp19a1 was remarkably elevated in the high fat group, which was nearly 2.0-fold higher than the normal control. However, such phenomenon was completely reversed after metformin group, especially the high dose group.

**Conclusions**: High fat diet could induce sex precosity and insulin resistance in female rats. Metformin could decrease the insulin resistance, and the E2, and aromatase protein and mRNA expression in a dose dependent manner.

## P1-6-10 Clinical, endocrine characteristics and genetic analysis of Korean children with McCune - Albright syndrome: A retrospective cohort study

### Sung Yoon Cho^1^, Eun- Kyung Cho^1^, Jinsup Kim^1^, Chang-Seok Ki^2^, Ji-Eun Lee^3^, Sujin Kim^4^, Sung Won Park^5^, Young Bae Sohn^6^, Dong-Kyu Jin^1^

#### ^1^Department of Pediatrics, Samsung Medical Center, Sungkyunkwan University School of Medicine; ^2^Department of Laboratory Medicine & Genetics, Samsung Medical Center, Sungkyunkwan University School of Medicine; ^3^Departments of Pediatrics, Inha University Hospital, Inha University Graduate School of Medicine; ^4^Department of Pediatrics, Myongji Hospital, Seonam Univeristy College of Medicine; ^5^Departments of Pediatrics, Dankook University College of Medicine, Cheil General Hospital & Woman's Health Care Center; ^6^Department of Medical Genetics, Ajou University Hospital, Ajou University School of Medicine

**Background**: McCune–Albright syndrome (MAS) is a rare disease and defined by the triad of fibrous dysplasia (FD), cafe’-au-lait spots, and peripheral precocious puberty (PP). Because of the rarity of this disease, only a few individuals with MAS have been reported in Korea. We describe the various clinical, endocrine manifestations, and genetic analysis of fourteen patients with MAS in Korea.

**Methods**: Patient's clinical data including peripheral PP, FD, and other endocrine problems were reviewed retrospectively. In addition, treatment experiences of letrozole on five patients with peripheral PP were described. Mutant enrichment with 3’-modified oligonucleotides - polymerase chain reaction (MEMO-PCR) was performed on eight patients to detect mutation in GNAS using blood. MEMO-PCR is a simple and practical method that enables nondestructive selection and enrichment of minor mutant allele in blood.

**Results**: The median age at diagnosis was 5 years 2 months (range from 18 months to 16 years). Eleven patients were female and three male. Thirteen patients showed FD. All female patients showed peripheral PP at onset, and three patients subsequently developed central PP. There was a significant decrease in estradiol level after two years of letrozole treatment. However, bone age was advanced in four patients. Two patients had clinical hyperthyroidism and two patients had growth hormone excess with pituitary microadenoma. c.602G>A (p.Arg201His) in GNAS was detected in two patients in blood (by MEMO-PCR) and c.601C>T (p.Arg201Cys) in GNAS was detected in one patient in pituitary adenoma tissue (by Sanger sequencing).

**Conclusions**: This study described various clinical manifestations of fourteen patients with MAS in single center in Korea. This study first applied MEMO-PCR on MAS patients to detect GNAS mutation. Because a broad spectrum of endocrine manifestations could be found in MAS, multiple endocrinopathies should be monitored in MAS patients. Better treatment options for peripheral PP with MAS are needed.

## P1-6-11 Two brothers with ATRX mutation having micropenis without other features of ATR-X syndrome

### Mami Eguchi^1^, Yukiyo Yamamoto^1^, Reiko Saito^1^, Motohide Goto^1^, Shunsuke Araki^1^, Kazuyasu Kubo^1^, Rinko Kawagoe^1^, Yasusada Kawada^1^, Koichi Kusuhara^1^, Takeshi Sato^2^, Satoshi Narumi^2^, Tomonobu Hasegawa^2^

#### ^1^Department of Pediatrics, University of Occupational and Environmental Health; ^2^Department of Pediatrics, School of Medicine, Keio University

**Background**: Mutations in the ATRX gene cause X-linked alpha-thalassemia/mental retardation (ATR-X) syndrome. Typically ATR-X syndrome is characterized by severe mental retardation (>95%), alpha thalassemia (90%), facial abnormalities during infant (>90%), genital abnormalities (80%), microcephaly (75%) and short stature (65%). These genital abnormalities extend from hypospadias with microphallus to ambiguous female external genitalia. ATRX mutations cluster mainly in the ATRX-DNMT3-DNMT3L (ADD) domain ( `50%) and helicase domain ( `20%). The patient of ATRX mutation who has micropenis without other features such as mental retardation and facial abnormality was unreported previously.

**Cases**: Two of three brothers having micropenis visited our hospital. The characteristic data of three brothers were described in Table. We sequenced 26 genes implicated in 46XY disorders of sex development using MiSeq instrument. We found an ATRX sequence variation (c.3063G>C, p.Glu1021Asp), which is located outside ADD and helicase domains in all three brothers, confirmed by PCR-direct sequencing. This variation was not registered on the databases of SNPs. Glu1021 was conserved in rhesus, mouse, dog, elephant, chicken and Xenopus tropicalis. The sequence-based mutation prediction for c.3063G>C was damaging by Polyphen-2, and tolerated by SIFT. The brilliant cresyl blue stain for red blood cells is underway, being the most sensitive for detection of alpha thalassemia.

**Discussion**: A c.3063G>C (p.Glu1021Asp) must be a novel mutation, because of no registration of databases of SNPs, conservation of Glu1021 evolutionarily, and in silico analyses. Two of three brothers had micropenis while another didn’t. The phenotypic variation may be caused by other genetic and/or environmental factors. Intriguingly two brothers who had micropenis didn’t have other feature of ATR-X syndrome. ATRX gene analysis for patients with micropenis is warranted to find similar cases.

**Conclusion**: We identified a novel mutation of ATRX gene, c.3063G>C (p.Glu1021Asp), in two brothers who had only micropenis.

**Table 1 (abstract P1-6-11):**
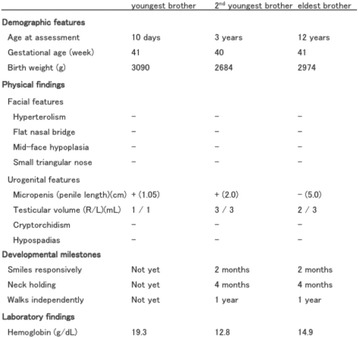
Characteristic data of 3 brothers having c.3063G>C, p.Glu1021Asp

## P1-6-12 The change in body mass index and insulin resistance after one-year treatment of gonadotropin-releasing hormone agonists in girls with central precocious puberty

### Park Jina

#### Department of Pediatrics, Inje University College of Medicine, Ilsan Paik Hospital

**Aims**: Gonadotropin-releasing hormone agonist (GnRHa) has been used as a therapeutic agent of central precocious puberty. There has been a concern of increased obesity after the GnRHa treatment. The purpose of this study is to compare the body mass index (BMI) and insulin resistance during the first year of GnRHa treatment in girls with CPP.

**Methods**: A total of 83 girls aged between 7.0-8.9 years with breast development and a peak LH of ≥ 5 IU/L after GnRH stimulation test was included as the patient group. The control group was 48 prepubertal girls. BMI and Indices related to insulin resistance, such as homeostasis model assessment of insulin resistance (HOMA-IR) and quantitative insulin sensitivity check index (QUICKI), were used to compared between two groups before the treatment and among the patient group before and after 1 year of GnRHa treatment.

**Results**: There was no statistical difference in BMI z-score between the control group and the patient group before the treatment. Fasting insulin and HOMA-IR were higher in the patient group, whereas FGIR and QUICKI were higher in the control group. (all P<0.001). In normal weight subjects in the patient group, BMI z-score was significantly increased during a year of GnRHa treatment (-0.1 ± 0.7 vs. 0.1 ± 0.8; P<0.001), whereas HOMA-IR and QUICKI showed no difference. In overweight subjects of the patient group, BMI z-score and HOMA-IR were not significantly different, whereas QUICKI was significantly decreased during GnRHa treatment (0.35 ± 0.03 vs. 0.33 ± 0.02; P=0.044).

**Conclusion**: The girls with precocious puberty showed increased insulin resistance compared to the control group. During the GnRHa treatment, the normal weight group showed increased BMI z-score without increase of insulin resistance, whereas the overweight group seemed to show increased insulin resistance without significant change in BMI z-score. Long-term follow up for the change of BMI and insulin resistance in patients with precocious puberty was needed.

## P1-6-13 46,XY disorder of sex development associated with a 2.2 Mb microdeletion in 9q33.3 including the NR5A1 gene with markedly elevated testosterone

### Takeshi Yamaguchi^1^, Toshihiro Tajima^1,2^, Shuntaro Morikawa^1^, Katsura Ishizu^1^, Tadashi Ariga^1^, Kimihiko Moriya^3^, Akie Nakamura^1,4^, Maki Igarashi^4^, Maki Fukami^4^

#### ^1^Department of Pediatrics, Hokkaido University School of Medicine; ^2^Department of Pediatrics, Jichi Children's Medical Center; ^3^Department of Renal and Genitourinary surgery, Hokkaido University School of Medicine; ^4^Department of Molecular Endocrinology, National Research Institute for Child Health and Development

**Background**: Although variations in key genes controlling gonadal development have been identified, the majority of 46,XY disorders of sex development (46,XY DSD) are unexplained after karyotyping and/or sequencing of known causal genes. We assessed a patient with unexplained 46,XY DSD using array comparative genomic hybridization (aCGH), and identified a 2.2Mb deletion in 9q33.3 including the NR5A1 gene, encoding SF-1/Ad4BP.

**A report of case**: The patient of 46, XY female is now 1 year and 5 months old. Left renal agenesis, bilateral cleft lip and palate were pointed out by prenatal ultrasound. She was delivered normally at 39 weeks of gestation with a birth weight of 2962g with normal female genitalia, minor dysmorphisms of ptosis, curled hair and rib hump. When an abdominal MRI scan was performed at 8 months of age, bilateral inguinal masses were identified, but female internal genitalia was not. Karyotype was 46,XY. Further endocrinological evaluation showed that serum testosterone was 163 ng/dL(normal range 12 `21 ng/dL) and AMH 82.6ng/mL (normal range 14 `466ng/mL), respectively. Serum LH and'sH showed normal response to GnRH stimulation. After hCG stimulation serum testosterone increased from 347.2ng/dL to 932.1ng/ dL and T/DHT ratio was normal. As she showed marked increase of testosterone after hCG stimulation, she was suspected to have androgen resistance. Molecular analysis of the AR gene did not show any mutation, however aCGH identified a heterozygous 2.2Mb deletion in 9q33.3. Her developmental milestones are almost normal so far.

**Discussion**: Testosterone levels elevated in our patient similar to androgen resistance, however there is no genetic defect of the AR gene. Instead a 2.2 Mb microdeletion in 9q33.3 including the NR5A1 gene was identified. Facial dysmorphism, cleft palate, and renal agenesis may be related to other genes in the 2.2 Mb deleted region. Similar to our case, it has been reported that some patients with NR5A1 mutations had ambiguous genitalia despite high serum testosterone levels. The exact reason for why androgen resistance occurred is not clear. Further investigation is necessary. In conclusion, our case further expands on the range of mutations associated with NR5A1 and aCGH studies in patients presenting with unusual or syndromic DSD is useful.

## P1-6-14 Two Chinese Preschool Girls with 46, XY Disorders of Sex Development Due to17β-Hydroxysteroid Dehydrogenase type 3 (17β-HSD3) Deficiency with Novel Mutation in The HSD17B3 Gene

### Jia jia Chen, chun xiu gong, Yan Ning Song

#### Division of Endocrinology and Metabolism, Beijing children's hospital

The enzyme 17 β -hydroxysteroid dehydrogenase type 3 (17 β -HSD3) belonging to the short-chain dehydrogenase reductase (SDR) protein superfamily, catalyzes the conversion of androstenedione (AD) to testosterone (T) in the testes. And its deficiency is a rare disorder of sex development in 46,XY individuals. Mutations in the HSD17B3 gene confer a spectrum of 46,XY disorders of sexual organ development ranging from completely undervirilized external female genitalia, predominantly female, ambiguous, to predominantly male with micropenis and hypospadias. We report two chinese preschool girls with the 46,XY karyotype and 17 β -HSD3 deficiency, showing different degrees of genital ambiguity, increased androstenedione and decreased testosterone levels, and testosterone to androstenedione ratio < 0.8 after human corionic gonadotropin (hCG) stimulation. Both patients had been raised as females, and female gender identity was maintained in case 1, while case 2 chose the male gender. Molecular analysis revealed a previously unreported compound heterozygosis for c.242C>T Ac.172G>T Cof the HSD17B3 gene in case 1. In addition, compound heterozygosis for c.645A>C Ac.239G>A of the HSD17B3 gene which had reported, were identified in case 2.

## P1-6-15 A case of partial androgen insensitivity syndrome caused by a novel mutation of the AR gene

### Megumi Iwahashi^1,2^, Ayako Ozawa^1,2^, Takeshi Sato^3^, Satoshi Narumi^3^, Tomonobu Hasegawa^3^, Ichiro Miyata^2^

#### ^1^Department of Pediatrics, The Jikei University Katsushika Medical Center; ^2^Department of Pediatrics, The Jikei University School of Medicine; ^3^Department of Pediatrics, Keio University School of Medicine

## P1-6-16 Stage-specific expression of AMH and AMHR2 in germ cells during spermatogenic cycle of rat

### Masanori Ohta^1,2^, Yoshinao Z Hosaka^3^, Tetsuji Ohyama^4^, Yoshiaki Yamano^3^, Kenji Ohyama^1^

#### ^1^Department of Pediatrics, University of Yamanashi; ^2^Pediatrics, Tsuru municipal hospital; ^3^Faculty of Agriculture, Tottori University; ^4^Faculty of Medicine / Department of Mathematics and Statistics, Oita University

**Background**: Recently, we first clarified that spermatocytes expressed mRNAs and proteins of anti-Müllerian hormone (AMH) and AMH type II receptor (AMHR2) in rats aged 21 days (21d) by immunohistochemical staining and in situ hybridization. However, the physiological role of AMH on mature testis is not entirely clear, although AMH is abundant in seminiferous tubules. In this study, therefore, we planned to clarify the expression pattern of AMH and AMHR2 in germ cells of mature rat testis during spermatogenic cycle.

**Subjects**: SD rat testes and isolated germ cells of 49d testes.

**Methods**: Quantitative RT-PCR, in situ hybridization (ISH) and immunohistochemical staining (IHCS) of AMH and AMHR2 in 49d testes.

**Results**: In quantitative RT-PCR, germ cells isolated from 49d testes expressed Amh and Amhr2 .In ISH, signals of Amh and Amhr2 in spermatocytes were detected at all stages of spermatogenic cycle, especially intense signals were detected at stage VIII. Amh and Amhr2 signals in round and elongated spermatids were negative at all stages, those in spermatogonia were unclear, and in Sertoli cells were faint.In IHCS, expression of AMH in spermatocytes was negative or weak at stage I-IV, but that was strongly observed at stage VII-XIV. The strongest expression of AMH was observed at stage VIII. Expression of AMH in round spermatids was clearly observed at stage I-IV, but that was decreased at stage VII, and diminished in elongated spermatids at stage VIII-XIV. Expression of AMHR2 in spermatocytes was negative or weak at stage I-IV, but that was strongly observed at stage VII-XIV. Unlike expression of AMH in spermatids, the expression of AMHR2 was found in round and elongated spermatids of all stages, especially clearly observed in cell membranes of round spermatids at stage VII-VIII.

**Discussion**: In this study, we first clarified that spermatocytes and spermatids coexpressed AMH and AMHR2 stage-specifically during spermatogenic cycle in 49d rat testes. When spermatocytes differentiated to round spermatids, AMH and AMHR2 were transferred from spermatocytes to round spermatids. The pattern of expressions of AMH and AMHR2 indicate that AMH mainly produced by spermatocytes acts round and elongated spermatids through AMHR2 in paracrine fashion during all stages. It appears likely that AMH involves on morphological change in spermatids from round form into elongated form at stage VIII to IX, and the change in elongated form during stage IX to XIV and I to VIII.

## P1-6-17 CHARGE syndrome with a CHD7 mutation accompanied by a mutation in SOX2

### Miki Kamimura^1^, Hirohito Shima^1,2^, Erina Suzuki^2^, Chisumi Sogi^1^, Junko Kanno^1^, Maki Fukami^2^, Ikuma Fujiwara^3^

#### ^1^Department of Pediatrics, Tohoku University Hospital; ^2^Department of Molecular Endocrinology, National Research Institute for Child Health and Development; ^3^Department of Pediatric Endocrinology and Environmental Medicine, Tohoku University Graduate School of Medicine

**Background**: CHARGE syndrome is a clinically heterogeneous syndrome named by specific features such as coloboma of the eye Cheart defects Catresia of the choanae Cretardation of growth and/or development Cgenital and/or urinary abnormalities and ear abnormalities. Loss of function mutations in CHD7 are found in most cases. Microphthalamia syndrome caused by SOX2 mutations sometimes associated with hypogonadism. Case presentation FA boy was referred to us at 5 months of age because of micropenis. He was pointed out a right cupped ear Cright facial palsy and bilateral sensorineural hearing loss in the neonatal period. He had a small penis with length of 1.1cm and bilateral cryptorchidism. He also had a square face DThere were no congenital heart defects on cardiac ultrasonography, but an ophthalmologic examination revealed bilateral optic nerve hypoplasia. He grew well and his karyotype was normal (46, XY). Dilatation of the right lateral semicircular canal and vestibule and absence of the right anterior semicircular canal and the right stapes were revealed by computed tomography. Brain MRI analysis revealed a normal pituitary. He was diagnosed clinically as CHARGE syndrome according to the criteria by Blake, but since optic nerve hypoplasia was not seen frequently in CHARGE syndrome, we underwent gene analysis for 29 genes that related to hypogonadotropic hypogonadism by next generation sequencing. In addition to a novel missense mutation p.Arg1940Cys in exon 29 of CHD7, we identified a novel nonsense mutation p.Tyr110X in exon 1 of SOX2.

**Discussion**: Most of the features of this patient would be caused by the mutation in CHD7 because he was compatible with CHARGE syndrome clinically, except for optic nerve hypoplasia which might be caused by the mutation in SOX2. As SOX2 is also related to the development of inner ear and male genitalia, his genital abnormalities and hearing loss could be caused by the both mutations in CHD7 and SOX2 . Since SOX2 is reported to interact with CHD7, it is possible that mutations in both genes modify the phenotype of the patient.

**Consent for publication:** The authors declare that written informed consent was obtained for publication.

## P1-6-18 Outcomes of gonadotropin-releasing hormone agonist treatment in obese girls with central precocious puberty

### Kim Hye Ryun, Nam Hyo-Kyoung, Rhie Young-Jun, Lee Kee-Hyoung

#### Department of Pediatrics, Korea University College of Medicine

**Purpose**: The prevalence of obesity is at high rate in girls with central precocious puberty (CPP). There has been no reports about the effects after gonadotropin-releasing hormone agonist (GnRHa) treatment in obese patients with CPP. We aimed to compare the clinical course and the effect of GnRHa treatment in obese girls with normal weight girls.

**Methods**: We reviewed the medical records of 192 girls with CPP who had been treated with GnRHa. The patients were classified as normal weight (n=110) and obese group (n=82). We compared chronological age (CA), bone age (BA), the standard deviation score (SDS) of height, body mass index (BMI), predicted adult height (PAH) and laboratory findings at baseline, 1 year and the end of GnRHa treatment.

**Results**: Mean BMI at baseline were 16.87 ± 1.30 kg/m2 in normal weight group and 20.86 ± 1.35 kg/m2 in obese group. At the beginning of GnRHa treatment, the CA, BA and PAH was similar between the two groups. The difference between BA and CA was significantly higher in obese group than normal weight group at baseline and end of treatment. The difference between BA and CA in both groups significantly decreased compared with baseline during treatment respectively (P =0.000). The height-SDS for BA at 1 year and the end of treatment significantly showed increase in both groups. The height-SDS for BA between groups was similar during treatment. The PAH increased significantly in both groups after treatment compared with baseline (P =0.000). At the end of therapy, the PAH in obese group (159.99 ± 3.34 cm) was similar to that of the normal weight group (159.19 ± 3.22 cm). Factors influencing end PAH in obese group were initial height, baseline height-SDS for BA, height increment during 1st year, duration of treatment and basal'sH.

**Conclusion**: The GnRHa treatment in obese girls with CPP improved height outcome similar to that in the normal weight girls. Obesity may not affect the treatment efficacy in CPP.

## P1-6-19 Nourishing “Yin” -removing “Fire” Chinese herbal mixture delays puberty onset through hypothalamic mTOR signaling in female rats

### Jian Yu^1^, Gulan Zeng^1^, Xinghui Han^1^, Yonghong Wang^1^, Zhanzhuang Tian^2^

#### ^1^Department of Integrative Medicine, Children's Hospital of Fudan University; ^2^Department of Neurobiology and Integrative Medicine, Shanghai Medical College of Fudan University

**Background**: The nourishing “Yin”-removing “Fire” (NYRF) Chinese herbal mixture can effectively delay the onset of puberty in children with precocious (early) puberty. In our current study, female rats undergoing puberty were used as experimental models to explore the effects of NYRF herbal mixture on puberty onset and expression of hypothalamic mTOR gene and protein levels in these rats. We further investigated the neurobiological mechanism by which this Chinese medicine delays the puberty onset.

**Methods**: Forty female 20-day-old (d20) SD rats were randomly divided into Chinese herbal mixture (CHM) and normal saline (NS) groups (20 rats in each group). Rats in CHM and NS groups were treated with the NYRF mixture and an equal volume of normal saline, respectively, from d22. Five rats in each group were sacrificed on d28 and d31 (before onset of puberty). On d34, the rest 10 rats from each group were sacrificed to obtain samples. Serum luteinizing hormone (LH), follicle stimulating hormone (FSH) and estradiol iE2 ) levels were analyzed by ELISA. Hypothalamic mTOR mRNA expression levels were determined by RT-PCR and hypothalamic p-mTOR protein levels were assayed by western blot.

**Results**: The vaginal opening time in the CHM group was significantly delayed (P < 0.05). On d31, serum LH and E2 levels were significantly lower in the CHM group (P < 0.05), in comparison with those in the NS group, and the expression levels of hypothalamic mTOR mRNA and p-mTOR protein were significantly lower (P < 0.05).

**Conclusion**: Nourishing “Yin”- removing “Fire” Chinese herbal mixture can delay puberty onset in rats, and down-regulate the expression levels of mTOR mRNA and p-mTOR protein within the hypothalamus. Therefore, the mechanism by which this Chinese herbal mixture delays puberty onset may be associated with the inhibition of the hypothalamic mTOR signaling pathway.

**Keywords**: nourishing “Yin”-removing “Fire” Chinese herbal mixture; mTOR signal; puberty onset.

## P1-6-20 The effect of letrozole on the reproductive function and linear growth in the early and middle period of puberty boy

### Juan Lin, Mei Hua Ma

#### Department of Pediatrics, The Third Affiliated Hospital, Sun Yat-Sen University

**Objective**: To provide clinical data for the effect of letrozole on the reproductive function and linear growth in the early and middle period of puberty boy.

**Method**: 43 early and middle pubertal boy with seriously damaged predict adult height, treated with letrozole 1.5 mg/m2/d Po( ≯ 2.5mg/d) were enrolled as treatment group. 48 cases of healthy puberty boy (include boy with idiopathic short stature) were enrolled as control. According to the letrozole treatment time, divide into short time group, mid-long time group, long time group, the control group as the match.

**Results**: After letrozole treatment, testicular volume and Genital stage of treatment group with short time and mid-long time group were significantly increased compared with the corresponding control group. The testicular volume and T in control group had a good positive correlation, as well in treatment group (r = 0.803, P < 0.001 and r = 0.835, P < 0.001).The testosterone levels in each group of treatment group were significantly higher than accordingly control group, as well as serum'sH, LH, LH/FSH level. While E2 level with long time group of treatment group significantly reduced than the control group. 17 cases of control group, 13 cases of treatment group had serum AMH, INHB level tested before and after intervention. The control group of serum AMH level had decreasing trend in puberty, blood testosterone values elevated gradually, and serum AMH and T show a significantly negative correlation (r = -0.466, p=0.059). While the treatment group of serum AMH and T show significant positive correlation (r=0.586.p=0.035), differ from normal AMH change trend. Serum INHB level in two groups after intervention all had different level rise, and INHB in control group increase more than the treatment group, but there was no statistically significant difference.Children in long time group of treatment group had more evident bone age inhibition than control group.

**Conclusion**: The testicular volume change and testosterone levels changes had high consistency, suggesting it may clinical preliminary judge serum testosterone levels according to the size of testis. Letrozole treatment can obviously promote the secondary sex characters development in adolescent boys. The AMH change trend with letrozole treatment group differ from normal trend, prompt that it may have inhibitory effect on testis maturity. Letrozole treatment group with its serum INHB level had a little different with control group, it can not deny that testis sertoli cells function had unaffected completely, further study needed. Children with long-term letrozole treatment had bone age delayed.

## P1-6-21 A case of newborn Kallmann syndrome diagnosed by micropenis and ANOS1/KAL1 gene abnormality

### Hajime Yasuhara^1^, Shingo Okamoto^2^, Mari Hasegawa^3^, Shinji Fukui^4^, Hideki Minowa^1^

#### ^1^Divison of Neonatal Intensive Care, Nara Prefecture General Medical Center; ^2^Department of Internal Medicine, Okamoto Internal Medicine and Pediatrics Clinic; ^3^Department of Pediatrics, Nara Medical University; ^4^Department of Urology, Nara Prefecture General Medical

## P1-6-22 Investigation of polychlorinated biphenyls effects on intracellular estrogen metabolism in ovarian cell culture

### Selim Kilic^2^, Ediz Yesilkaya^1^, Adem Kocak^1^, Recai Ogur^1^, Aysun Bideci^2^, Fatma Demirel^3^, Vural Kesik^1^, Selin Elmaoullar^1,3^, Erkan Sari^1^, Onur Akin^1^, Ahmet Bolat^1^, Ahmet Tas^1^, Esra Doger^3^

#### ^1^Gülhane Military Medical Academy; ^2^Gazi University Faculty of Medicine, Pediatric Endocrinology; ^3^Yıldırım Beyazıt University Pediatric Endocrinology

Premature puberty and thelarche are frequent conditions for female children, but the etiologic reasons have not been obviously demonstrated yet. Many underlying causes are known and endocrinologic disruptors are one of them. Among endocrinologic ones, PCBs may cause this kind of conditions due to their high toxicity and long-term effects. Intracellular estrogen metabolism comprising the step starts with the synthesis of pregnenolone from cholesterol. In these metabolic pathways where many intermediate compounds were synthesized and destroyed by CYP family of enzymes in this metabolic pathway, many enzymes including CYP enzyme family (CYP11 A, CYP17, CYP19, CYP3A4, CYP1A1 / 2 and CYP1B1), catechol O-methyltransferase (COMT) enzyme and 17-beta-hydroxysteroid dehydrogenase 1-2 enzymes, are involved. Aroclor 1254 and 1260 had a general inhibitory effect on enzymes of intracellular mechanisms and decreased estrogen mRNA expression levels in varied degrees. We studied Aroclor 1254 and 1260, the CYP11, CYP17, CYP19, COMT and 17beta-hidroksteroiddehidrogenaz (17ß-HSD) 1-2 enzyme mRNA expressions in OVCAR3 and HGF-1 cells series. We detected that Aroclor 1254 and Aroclor 1260 are lowering enzyme mRNA expression levels in various amounts by inhibiting the attendant enzymes in estrogen metabolism in OVCAR3 and HGF-1 cell series. Since this inhibition is blocked totally or partially in the presence of fulvestrant, we determined that Aroclor 1254 and Aroclor 1260 are made their effects through the estrogen receptors.

## P1-6-23 Premature ovarian insufficiency in 5 patients with leukemia post hematopoietic stem cell transplantation

### Wenjing Li^1^, Chunxiu Gong^1^, Maoquan Qin^2^, Huyong Zheng^2^

#### ^1^Department of Endocrinology, Genetics and Metabolism, Beijing Children's Hospital, Capital Medical University; ^2^Beijing Children's Hospital, Capital Medical University, Hematology Oncology Center

**Objective**: To assess the relationship between premature ovarian insufficiency iPOI ) and hematopoietic stem cell transplantation (HSCT) through the investigation of the clinical characteristics of POI post HSCT in 5 patients with leukemia.

**Methods**: Follow-up the gonadal functions of the patients post HSCT after 7 years to diagnose POI, and collected and analyzed the clinical characteristics through the clinical data.

**Result**: POI is defined as a primary ovarian defect characterized by absent menarche (primary amenorrhea) or premature depletion of ovarian follicles before the age of 40 (secondary amenorrhea) with hypergonadotropism (follicle-stimulating hormone,'sH > 40IU/l) and hypoestrogenism. The patients were diagnosed with leukemia aged from 6.5 to 10.2, and underwent HSCT at the age from 7.4 to 10.9. POI was diagnosed about 0.2 to 4 years later post HSCT in 5 girls, clinical data shown in table 1.

The first visit of one patient’s visiting at endocrinal clinic was 4 year post HSCT. She had breast developed spontaneously, but she didn’ t have menstruation spontaneously 3 years later. Only one patient complained of amenorrhea. The other 4 patients diagnosed by interim sexual hormone screening with elevated gonadotropin levels. The sex and gonadotropin hormone levels were normal in 3 patients post chemotherapy and before HSCT. 2 patients took pelvic ultrasonography post HSCT and before POI onset, with size over 0.3cm of follicle detected. All the POI patients showed that the levels of LH and'sH were elevated; meanwhile the serum estradiol levels were decreased. The levels of AMH and inhibin B were decreased also. There were no detectable follicles in 4 patients by pelvic ultrasonography.

**Conclusion**: POI post HSCT was happened at pre-adolescent or early adolescent stage. POI clinical symptoms were occult that only one patient complained of primary amenorrhea. Most of the patients were identified through screening. Pediatric POI is more likely happened at 0.5 to 1 year post HSCT. To early diagnosis of POI, or prevent POI onset is our new research direction. Recommend regular inspection and evaluation of gonadal function before and post HSCT.

**Table 1 (abstract P1-6-23).**
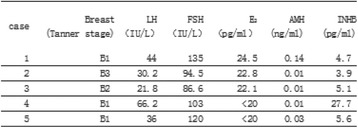
See text for description

## P1-6-24 Determination of serum and urinary levels of polychlorinated biphenyls in girls precocious puberty and premature thelarche

### Ediz Yesilkaya^1^, Adem Kocak^1^, Recai Ogur^1^, Aysun Bideci^1,2^, Fatma Demirel^3^, Vural Kesik^1^, Selin Elmaogullar^1,3^, Erkan Sari^1^, Onur Akin^1^, Ahmet Bolat^1^, Ahmet Tas^1^, Esra Doger^2^, Selim Kilic^1^

#### ^1^Pediatric Endocrinology, Gülhane Military Medical Academy; ^2^Pediatric Endocrinology, Gazi University Faculty of Medicine; ^3^Pediatric Endocrinology, Yıldırım Beyazıt University Pediatric Endocrinology

Premature puberty and thelarche are frequent conditions for female children, but the ethiologic reasons have not been obviously demonstrated yet. Many underlying causes are known and endocrinologic disruptors are one of them. Among endodocrinologic ones, PCBs may cause this kind of conditions due to their high toxicity and long-term effects. The aim of this study is to investigate potential relationship between PCBs and premature puberty and thelarche. Serum and urine PCB levels of female children with premature puberty and thelarche were compared with same-age-group healthy female children. Serum and urine PCB levels were measured with GC-EDC device and mRNA expressions in cell cultures were evaluated with RT-PCR. In overall interpretations of serum samples, PCB 138, PCB 151 and PCB 101 were most frequently detected. In control group, mostly PCB 151 was detected, but in premature puberty and thelarche group, most commonly PCB 138 was detected. In overall interpretations of urine samples, PCB 151, PCB 18 and PCB 66 were most frequently detected. In control group, mostly PCB 151 was detected, but in premature puberty and thelarche group, yet again, most commonly PCB 52 was detected.

## P1-6-25 Use of dihydrotestosterone cream in 5-alpha reductase 2 deficiency

### Ho-Chung Yau

#### Department of Paediatrics, Prince of Wales Hospital

**Background**: 5-alpha reductase 2 deficiency is an autosomal recessive disorder characterized by lack of virilization in male infants due to failure to convert testosterone to dihydrotestosterone. Use of dihydrotestosterone (DHT) cream has been successfully promoting phallic growth in infants with this condition. We reported the effects of use of DHT cream in our patients.

**Case Studies**: Patient 1 was born at 36 weeks by vaginal delivery with birth weight 2415gram. Antenatal scan showed bilateral prominent labioscrotal folds and suspicious testes inside but genital tubercle not enlarged. Parents were non-consanguineous. There was no family history of note. Examination at birth revealed no dysmorphism nor midline defect. There was penoscrotal hypospadias with chordee. Phallic length measured 2.0cm and width 0.8cm. Both gonads were palpable in bifid scrotum. At day 1 of life, his luteinizing hormone (LH) was 4.4IU/ L, follicle-stimulating hormone (FSH) 1.9IU/L and testosterone 8.4nmol/L. Karyotype was 46,XY. Ultrasound showed both testes in scrotum and no Mullerian structure. Mutational analysis of SRD5A2 gene confirmed compound heterozygous mutations. Spot urine steroid profiling at 5 months of age suggested 5-alpha reductase 2 deficiency. At 12 months of age, stretched penile measured 2.3cm and width 0.8cm. Hence, hypospadias surgery was deferred. DHT cream was started at 0.27mg/kg/day. At 17 months of age, stretched penile length measured 3.5cm and width1.5cm. Hypospadias surgery became feasible.

Patient 2 was born full term in mainland China. Antenatal course was uneventful. Parents were non-consanguineous. There was no family history of note. Karyotype was 46,XY. Blood tests at 3 months of age showed LH 1.30IU/L,'sH 0.94IU/L, testosterone 8.84nmol/L and dihydrotestosterone 13ng/dL (reference range 16-79ng/dL). Ultrasound was unremarkable. Examination at 11 months of age revealed no dysmorphism nor midline defect. There was scrotal hypospadias with chordee. Phallic length measured 1.5cm and width 0.7cm. Both gonads were palpable in prepenile bifid scrotum. Hypospadias surgery was deferred until favourable penile size. Spot urine steroid profiling at 23 months of age suggested 5-alpha reductase 2 deficiency. Mutational analysis of SRD5A2 gene confirmed compound heterozygous mutations. At 29 months of age, stretched penile length measured 2.0cm and width 0.8cm. DHT cream was started at 0.17mg/kg/day. At 36 months of age, stretched penile length measured 3.0cm and width 1.0cm. Hypospadias surgery became feasible.

**Conclusion**: Use of DHT cream at 0.17-0.27mg/kg/day for a period of 4-7 months was effective in promoting penile growth prior to hypospadias surgery in our patients. No adverse effect had been reported.

**Consent for publication:** The author declare that written informed consent was obtained for publication.

## P1-7-1 Mortality and incidence of misidentification of sex assignment in patients with congenital 21-hydroxylase deficiency identified by a newborn screening program in Japan (2001-2015)

### Kei Takasawa^1,2^, Tomohiro Ishii^2,3^, Satoshi Okada^2,4^, Hotaka Kamasaki^2,5^, Takuo Kubota^2,6^, Hironori Kobayashi^2,7^, Hirotake Sawada^2,8^, Keisuke Nagasaki^2,9^, Chikahiko Numakura^2,10^, Shohei Harada^2,11^, Kanshi Minamitani^2,12^, Shigetaka Sugihara^2,13^, Masanori Adachi^2,14^, Toshihiro Tajima^2,15^

#### ^1^Department of Pediatrics and Developmental Biology, Tokyo Medical and Dental University; ^2^The Japanese Society for Pediatric Endocrinology, the Committee for the Newborn Screening; ^3^Department of Pediatrics, Keio University School of Medicine; ^4^Department of Pediatrics, Hiroshima University Graduate School of Biomedical & Health Sciences; ^5^Department of Pediatrics, Sapporo Medical University School of Medicine; ^6^Department of Pediatrics, Osaka University Graduate School of Medicine; ^7^Department of Pediatrics, Shimane University School of Medicine; ^8^Division of Pediatrics, University of Miyazaki Faculty of Medicine; ^9^Department of Homeostatic Regulation and Development, Niigata University Graduate School of Medical and Dental Sciences, Division of Pediatrics; ^10^Department of Pediatrics, Yamagata University School of Medicine; ^11^Division of Neonatal Screening, National Center for Child Health and Development; ^12^Department of Pediatrics, Teikyo University Chiba Medical Center; ^13^Department of Pediatrics, Tokyo Women's Medical University Medical Center East; ^14^Department of Endocrinology and Metabolism, Kanagawa Children's Medical Center; ^15^Jichi Children's Medical Center Tochigi, Pediatrics

**Background**: Congenital adrenal hyperplasia is a potentially lethal condition and was assumed to be associated with excess mortality due to adrenal crisis. In Japan, a nationwide newborn screening program for congenital adrenal hyperplasia due to 21-hydroxylase deficiency (21- OHD) has been carried out since 1989, and it has detected one case in approximately every 18,000-22,000 births. Our committee reported 12 cases of death during 12 years after the introduction of the newborn screening (1989-2001), suggesting that mortality is estimated to be one in 25-80 21-OHD patients in childhood.

**Objective**: To review mortality and causes of death in 21-OHD patients since 2001 and to clarify incidence of misidentification of sex assignment in 21-OHD patients

Design: We performed a questionnaire survey for all councilors of the Japanese Society for Pediatric Endocrinology. Clinical information of cases of death or misidentification of sex assignment was collected from each attending physician in a secondary survey.

**Results**: Eighty five out of 178 questionnaires were available for the analysis (response rate: 48%). Only two deaths including one 2-years- old case with influenza-associated adrenal crisis and another adult case were reported. The mortality was estimated to be one in 400 21-OHD patients in childhood and improved from that before 2001. Furthermore, we detected two cases required changing sex status on the family register in infant. Both cases had salt-wasting type of 21-OHD and Prader stage V of ambiguous genitalia. After changing sex status, both cases did not reveal treatment noncompliance or gender confusion.

**Conclusion**: Our report suggested that enlightenment and dissemination of knowledge on 21-OHD throughout the newborn screening might contribute to improvement of mortality in 21-OHD patients. Furthermore, call for further attention to the aspect of disorders of sex development in 21-OHD might be required.

## P1-7-2 Infantile body weight gain up to 60 days of age is associated with height at 1 year of age in classical 21-hydroxylase deficiency

### Masahiro Goto^1^, Kazuhisa Akiba^1^, Kenichi Kashimada^2^, Koji Muroya^3^, Masanori Adachi^3^, Tsuyoshi Isojima^4^, Yukihiro Hasegawa^1^

#### ^1^Division of Endocrinology and Metabolism, Tokyo Metropolitan Children's Medical Center; ^2^Department of Pediatrics and Developmental Biology, Tokyo Medical and Dental University; ^3^Department of Endocrinology and Metabolism, Kanagawa Children's Medical Center; ^4^Department of pediatrics, Graduate school of medicine, The University of Tokyo

**Background**: Growth suppression becomes evident by 1 year of age in patients with classical 21-hydroxylase deficiency (21OHD), and may result in reduced adult height.

Objective: To clarify the relationship between body weight gain in early infancy and height at 1 year of age in the classical 21-hydroxylase deficient population.

**Subjects and Methods**: 92 patients from 3 medical institutions in Japan were enrolled. The inclusion criteria were the diagnosis of classical 21OHD and the availability of height and body weight records for at least 6 different time points between birth and 1 year of age. Height and body weight on days 0, 30, 60, 90, 180, 270, and 360 were calculated by a linear fit between their pre- and post-measurements. The SD (standard deviation) of these auxological parameters was calculated, and the sequential changes were examined. The degree of change in the SD from day 0 was calculated on each designated date.

**Results**: Mean height SD decreased significantly from 0.45 on day 0 to -1.34 on day 180 but showed no significant change afterwards. Mean body weight SD decreased significantly from 0.45 on day 0 to -0.59 on day 60. Significant correlation was observed between Δ body weight SD (day 0-60) and Δ height SD (day 0-360) (see Figure below).

**Conclusion**: Growth suppression in patients with classical 21OHD was discussed in relation to high dose glucocorticoid supplementation. Our results suggested that poor body weight gain in early infancy may be another factor affecting prognosis in terms of height.

**Fig. 1 (abstract P1-7-2).**
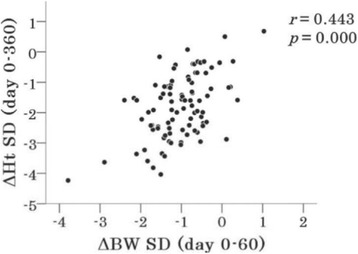
See text for description

## P1-7-3 Steroid Hormone Profiles of 6- to 14-Year-Old Normal Male Children by LC-MS Method

### Bingyan Cao, Chunxiu Gong, Di Wu, Xuejue Liang, Wenjing Li, Min Liu, Chang Su, Miao Qin, Xi Meng

#### Division of Endocrinology and Metabolism, Beijing Children’s Hospital, Capital Medical University

**Objectives**: To establish reference intervals for steroid hormones of 6- to 14-year-old normal male children using the LC-MS method and study the variation pattern of steroid hormones in terms of age and sexual maturation.

**Subjects and Methods:** A total of 820 male children with normal weight aged 6 to 14 years from Shunyi District, Beijing, participated in this research study. The puberty status was assessed by trained pediatricians according to the Tanner method (Tanner genital staging: stage G1, prepuberty; stage G5, postpuberty). Pregnenolone, 17 α hydroxyprogesterone, corticosterone, dehydroepiandrosterone (DHEA), and androstenedione were measured by the Liquid Chromatography-Mass Spectrometry method (LC-MS). Free testosterone was measured by the chemiluminescence immunoassay method (CLIA). Statistics were analyzed with SPSS 17.0. An abnormal distribution was described by the median and inter-quartile ranges. The reference range used 2.5 percentile as the lower limit and 97.5 percentile as the upper limit. Interclass variance was analyzed with the Kruskal-Wallis test.

**Results**: Median pregnenolone and 17áhydroxyprogesterone concentrations of normal male children did not exhibit significant variance at the adrenarche ages, from approximately 6-8 years old (before puberty initiation) to 9 years old. After the age of 10-11 years, the concentrations increased with age. The level of corticosterone did not change significantly with age. Median levels of DHEA, androstenedione, and free testosterone increased with age. Pregnenolone displayed a significant increase from stage G1 to G2 and remained steady from stage G3 onward. 17 α -hydroxyprogesterone rose evidently with the progression of puberty from stage G1 to G3 and slowed from stage G4. Corticosterone did not change with the progression of puberty. DHEA and androstenedione exhibited significant increases from stage G1 to G2 with no evident intersection and increased at a lower rate after stage G3. Free testosterone levels rose sharply during puberty from stage G1 to G3 without evident intersection and rose slowly after stage G4.

**Conclusion**: The concentration of the precursor of mineralocorticoids in the zona glomerulosa (ZG) of male children from the age of 6 to 14 years does not relate to age or the progression of puberty; however, the concentrations of glucocorticoids and their precursors in the zona fasciculate are associated with pubertal progression. The levels of androgens increase with age and sexual maturation.

## P1-7-4 Etiology of Primary Adrenal Insufficiency in Children: A 29-Year Single Center Experience

### Melati Wijaya, Huamei Ma, Minlian Du, Yanhong Li, Hongshan Chen, Qiuli Chen, Jun Zhang, Song Guo

#### Division of Endocrinology and Metabolism/ Department of Pediatrics, The First Affiliated Hospital of Sun Yat Sen University

**Introduction**: Primary Adrenal Insufficiency(PAI) in children is rare, potentially lethal but treatable. The etiology of PAI in children differs substantially from that in adult population, diagnostic investigation is quite challenging.

**Objective**: To investigate the etiology and clinical features of children with PAI

**Method**: Retrospective study between Sept 1989 and Mar 2016

**Result**: 427cases were included (228males, 199females). Median age at diagnosis was 1.66(10th-90th C0.06~8.73 ) y respectively.An identified diagnosis (clinical or genetic) was obtained in 93.4% children with PAI (399/427). In the other twenty cases (6.6%, 28/427), though the etiology unidentifiable, congenital adrenal hyperplasia(CAH) had been excluded. Of which 399 cases of known etiology, i1 )351 cases (88.0%) were CAH, which 21 hydroxylase deficiency(21OHD) were the most common etiology (341cases,97.2%). The other CAH formed were 17 hydroxylase deficiency(17OHD) (5cases,1.4%), 11 hydroxylase deficiency(11OHD) (3cases,0.9%), congenital lipoid adrenal hyperplasia(CLAH) (2 cases, 0.6%). i2 )48 cases (12.0%) were non-CAH etiology. The etiology of this group were Adrenoleuokodystrophy(ALD) (22cases,45.8%), DAX1 mutation(19cases,39.6%), Autoimmune Polyglandular Syndrome(APS)(3cases,6.8%), Triple A Syndrome(AS)(2cases,4.2%), Steroidogenic factor (SF1) mutation (1case,2.1%), Adrenalectomy (1case,2.1%). Comparison based on sexual phenotype of 58 cases non-CAH 21OHD. Male phenotype patients were predominantly in this study (49/58, 84.5%). ALD was the most common etiology of male phenotype PAI (49%, 22/49). The other were DAX1 mutation 38.8%(19/ 49) CAPS 6.1%(3/49). The 11OHD, CLAH, AS and adrenalectomy only accounted for 2.0% (1/49) of cases. Female phenotype patients were fewer than male(15.5%, 9 cases). 17OHD was the most common in female, accounted for 44.4% (4/9) of cases. The other etiology: 11OHD, LCAH, SF1, AS were rarer, which accounted for 22.2% (2/9), 11.1% (1/9), 11.1% (1/9), 11.1% (1/9) of cases respectively. PAI Clinical features: genital ambiguity was the common manifestation, accounted for 42.4%. The other features include gastrointestinal symptoms35.4% Cgrowth failure26.7%, gonadal related symptom(premature pubarche, sexual infantilism, amenorrhea) 21.1%, hyperpigmentation9.8%, demyelination of central nervous system3.3%, prolonged jaundice2.3%, fatigue2.3%, convulsion2.3%. 62.3% patients presented 2 or more onset of symptoms, 4.2% patients with adrenal crisis onset. Physical examination showed that only 57.6% patients were exhibiting hyperpigmentation.

**Conclusion**: 1) PAI in pediatric population is commonly in congenital forms, with CAH being the most frequent etiology. 2) Children with PAI have wide range symptoms, lack specificity. 3) Diagnosis of the underlying cause of PAI in children is recommended. Identification of a genetic etiology can provide information about prognosis and treatment.

## P1-7-5 Mutation spectrum of the CYP21A2 gene and clinical outcome of patients with steroid 21-hydroxylase deficiency in Korea

### Jin-Ho Choi^1^, Eungu Kang^1^, Yoon-Myung Kim^1^, Beom Hee Lee^1^, Gu-Hwan Kim^2^, Han-Wook Yoo^1^

#### ^1^Asan Medical Center Children's Hospital, University of Ulsan College of Medicine, Pediatrics; ^2^Asan Medical Center Children's Hospital, Medical Genetics Center

**Purpose**: Steroid 21-hydroxylase deficiency is the most common enzymatic defect of congenital adrenal hyperplasia (CAH) caused by inactivating mutations in the CYP21A2 gene. This study investigated mutation spectrum of CYP21A2 and clinical outcome in patients with 21-hydroxylase deficiency.

**Patients and methods**: This study included 157 patients with 21-hydroxylase deficiency from 143 independent pedigrees. The diagnosis was confirmed by by biochemical or molecular analysis of CYP21A2 using differential PCR amplification of CYP21A2 and CYP21A1P , followed by entire CYP21A2 gene sequencing and multiplex ligation dependent probe amplification analysis.

**Results**: Of a total of 157 patients, 115 patients (73.2%) were salt-wasting form, 41 patients (26.1%) were simple-virilizing form, and 1 patient (0.6%) had non-classical form. One hundred and two patients of salt-losing 21-OHD (102/115, 88.7%) were diagnosed in the neonatal period. Five genetic female patients (46,XX) with simple-virilizing form of 21-OHD were assigned as male sex because of delayed diagnosis. Molecular analysis of CYP21A2 was performed in 134 pedigrees (99 with salt-wasting form and 35 with simple-virilizing form). The c.293- 13A>G was the most common mutation in patients with salt-wasting form (63/198 alleles, 31.8%), whereas p.I172N was the most frequent in simple-virilizing form (27/70 alleles, 38.6%). Forty patients (29.9%) had homozygous mutation for large gene deletion (9/134, 6.7%), c.293- 13A>G (19/134, 14.2%), p.I172N (4/134, 3.0%), p.R356W (4/134, 3.0%), c.1451_1452delinsC (2/134, 1.5%), p.S371G (1/134, 0.7%), and c.923_924insC (1/134, 0.7%), whereas 94 patients (71.2%) were compound heterozygotes.

**Conclusions**: There was a broad spectrum of the clinical and hormonal phenotypes of 21-hydroxylase deficiency depending on the genotype. Further research is needed to identify modifier genes in 21-hydroxylase deficiency, which could explain the phenotypic variability of androgen effects.

## P1-7-6 Developmental and physical outcome in children exposed to dexamethasone in utero for the prenatal treatment of 21-hydroxylase deficiency

### Yusuke Fujisawa, Yuuta Chiba, Yumiko Terada, Yasuko Ogiwara, Tomoko Yoshida, Kazuko Mizutani, Kanako Nakao, Keisuke Yoshii, Yasuhiro Naiki, Reiko Horikawa

#### Division of Endocrinology and Metamolism/Department of Pediatrics, National Center for Child Health and Development

**Introduction**: In 21-hydroxylase deficiency (21OHD), prenatal dexamethasone treatment has been performed in order to prevent virilization of affected girls by suppressing excessive fetal adrenal androgen, and consequently to avoid reconstructive surgery. Effectiveness of this therapy has been widely reported whereas animal studies and epidemiological data have indicated adverse effects including psychosocial developmental delay and possible late metabolic effects.

**Objective**: To determine physical and developmental outcome of children treated with dexamethasone in utero.

**Subjects and Treatment**: The prenatal dexamethasone treatment was approved by the ethics committee of our institution. 14 mothers of patients with 21OHD orally administered dexamethasone at a dose of 20 μ g/kgBW/day (max 1mg/day) from 6-11 weeks of gestation, after obtaining informed consents. At 11-13 weeks of gestation, chorionic villi sampling was performed, sex chromosome and genetic analysis were undertaken; the treatment was discontinued when the fetus was a boy or an unaffected girl. Only the mothers with affected female fetuses continue oral dexamethasone until delivery.

**Results**: Four out of 15 fetuses (one twins) were affected girls. One of these four was lost by elective abortion. Three affected girls were born with completely normal female genitalia. One of them had mild skin defect on forehead. Another one had partial permanent teeth defect. In 11 others, one was an affected boy, 6 were unaffected girls and 4 were unaffected boy. They showed no deformities. All 14 cases have been followed for their physical and mental outcome. One unaffected girl whose mother developed pregnancy induced hypertension at mid-gestation was born prematurely at 29 weeks, has shown normal motor development but had mild developmental delay with'sIQ 77 by WISC- IV at 6 years old. Other 13 have passed normal developmental milestone. In 3 cases, we conducted further developmental evaluations; 3-year- old affected girl showed total IQ 94 with PM (posture-motor) 105 and LS (lingual-social) 87 by new K-type developmental test; 4-year-old affected boy showed total IQ 91 with LS 94 and CA (cognition-adaptation) 89 by new K-type developmental test; 6 − year-old unaffected boy showed'sIQ 114 with PRI 124 and VCI 99 by WISC-IV.

**Conclusion**: Prenatal dexamethasone treatment with current regimen perfectly prevents virilizaion in affected female fetuses. However, its safety and long term developmental and metabolic/physical effects should be carefully further monitored.

## 1-7-7 The level of LH and'sH would be a monitoring marker for adult male 21-OHD patient

### Ryuichi Nakagawa^1^, Akito Sutani^1^, Tsuji Atsumi^1^, Yohei Matsubara^1^, Shigeru Takishima^1^, Kei Takasawa^1^, Kentaro Miyai^1,2^, Makoto Ono^1,3^, Toshikazu Oonishi^1,4^, Kenichi Kashimada^1^, Tomohiro Morio^1^

#### ^1^Department of Pediatrics and Developmental Biology, Tokyo Medical and Dental University; ^2^Department of Endocrinology and Metabolism, Tokyo Metropolitan Children's Medical Center; ^3^Department of Pediatrics, Tokyo Bay Urayasu Ichikawa Medical Center; ^4^Department of Pediatrics, Kinki Central Hospital

**Background**: Infertility is one of the major problems of male adult patients with 21-hydroxylase deficiency (21-OHD) because substitution therapy of glucocorticoid for preventing adrenal crisis is often insufficient to reduce androgen synthesis from adrenal cortex. The clinical details of male adult 21-OHD patients are not clarified, and there is no consensus of the treatment for preserving fertility in male adult 21- OHD patients.

**Patients and methods**: We carried out retrospective analysis of nine male 21-OHD patients (1: non-classical form, 2: simple virilizing form, 6: salt wasting form) who were followed up continuously from neonatal period to adulthood when they reached their final height. All patients were treated with glucocorticoid according to the guideline. It is reported that the patients with oligozoospermia had a mean testicular volume (TV).

**Result**: Three and six patients were categorized in group A and B, respectively. LH and'sH of group A were significantly lower than those of group B (p =0.02). LH/FSH and TV were positively correlated (r =0.45) with the formula of, LH(mIU/ml) = 0.1445 × TV (ml)+ 0.093 and'sH (mIU/ml)= 0.3035 × TV (ml)+ 0.1532. The mean level of LH and'sH of all patients was 1.9mIU/mL[0-2.6] and 3.0mIU/mL [0-4.6], respectively. The level of 17-OHP was correlated with TV (r =-0.45), but it was no difference between group A and B (p =0.3). Urinary pregnanetriol was not correlated with TV (r =-0.086) nor different between group A and B (p =0.2). Interestingly, TV and BMI was negatively correlated (r =-0.69, p =0.041).

**Discussion**: Our data suggested that basal gonadotropin is correlated with TV and would be a good marker to predict TV. Based on the formula mentioned above, in order to maintain TV 10ml or more, LH 1.5mIU/mL or'sH 3.0mIU/mL would be sufficient. The sensitivity and specificity of the cut-off level was 83% and 100%, respectively. The negative correlation between BMI and TV suggested that in some patients, it is difficult to preserve fertility without overtreatment of glucocorticoid.

**Conclusion**: LH>1.5mIU/mL or'sH>3.0mIU/mL would be sufficient to preserving fertility in 21-OHD adult male patients, but careful monitoring for iatrogenic Cushing’s syndrome is essential.

## P1-7-8 Hydrocortisone dose between 180 and 360 days may change Ht-SDS during the first 1 year of life with 21 OHD

### Kazuhisa Akiba^1^, Masahiro Goto^1^, Yoshihiko Morikawa^2^, Kenichi Kashimada^3^, Koji Muroya^4^, Masayoshi Adachi^4^, Yukihiro Hasegawa^1^

#### ^1^Division of Endocrinology and Metabolism, Tokyo Metropolitan Children's Medical Center; ^2^Tokyo Metropolitan Children's Medical Center, Clinical Research Support Center; ^3^Department of Pediatrics and Developmental Biology, Tokyo Medical and Dental University; ^4^Department of Endocrinology and Metabolism, Kanagawa Children's Medical Center

**Background**: In many patients with 21-hydroxylase deficiency (21OHD) adult height is not optimal. Height SD scores (Ht-SDS) of infancy are already compromised, which was speculated to be due to glucocorticoid overtreatment. Objective FTo show Ht-SDS during the first 1 year of life in 21OHD patients and their relationship with the doses of glucocorticoid.

**Subjects and Methods**: The subjects were 84 children with 21OHD who were treated with hydrocortisone (HC) and fludrocortisone at 3 institutions in Japan. Ht SDS at birth, 180 and 360 days were analyzed. The integrated dose of HC at each interval was calculated. The study population was divided into “good prognosis group (GPG; N=12)” and “poor prognosis group (PPG; N=72)”, where the difference in height SD score (SDS) between at birth and at 360 days of age was ≥ -0.5 SD and < -0.5 SD, respectively.

**Results**: Ht SDS were similarly decreased from birth to 180 days. Ht SDS from 180 to 360 days in GPG was increased significantly whereas those in PPG was not. GPG received significantly higher integrated HC dose between 180 and 360 days (“HC 180-360d”) (p<0.05). When the threshold of HC (180-360d) was set at 21.5 mg/m2/day, the sensitivity and specificity for GPC or PPG was 65% and 83%, respectively.

**Conclusion**: We may predict PPG with good specificity by using HC(180-360d) with cut-off value at 21.5 mg/m2/day.

**Fig. 1 (abstract P1-7-8).**
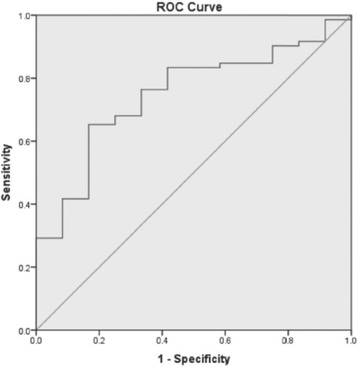
See text for description

## P1-7-9 Adrenal insufficiency observed in three children following administration of opioids through theμtype receptor

### Nozomi Matsuda^1^, Masahiro Goto^2^, Ichiro Watanabe^2^, Kazutoshi Akiba^2^, Shinji Higuchi^2^, Kentaro Miyai^2^, Naoki Shimizu^2^, Yukihiro Hasegawa^2^

#### ^1^Division of Endocrinology and Metabolism / Department of Pediatrics, Kawaguchi Municipal Medical Center; ^2^Division of Endocrinology and Metabolism / Department of Pediatrics, Tokyo Metropolitan Children's Medical Center

## P1-7-10 GnRH-independent Precocious Puberty in a Thai boy with NR0B1 novel mutation causing X-linked Adrenal Hypoplasia Congenita

### Karn Wejaphikul^1^, Prapai Dejkhamron^1^, Chanisa Suthiworachai^2^, Taninee Sahakitrungruang^3^, Kevalee Unachak^1^

#### ^1^Department of Pediatrics, Faculty of Medicine, Chiang Mai University; ^2^Department of Bioscience, Faculty of Science, Chulalongkorn University; ^3^Department of Pediatrics, Faculty of Medicine, Chulalongkorn University

**Introduction**: X-linked adrenal hypoplasia congenita (AHC) is caused by mutations of the NR0B1 (DAX1) gene. Clinical characteristics include primary adrenal insufficiency in infancy and hypogonadotropic hypogonadism in later life. Although pubertal failure is a key feature, GnRH- independent precocious puberty (GIPP) was established as an atypical presentation of this disease which may complicate the diagnosis.

**Case report**: A 1-month-old boy presented with salt wasting adrenal crisis. An ACTH stimulation test exhibited poor cortisol response (rose from 8.4 to 10.9 μ g/dL at 60 min) and moderately elevated 17-hydroxyprogesterone (17OHP) levels (baseline 480 and stimulated 7160 ng/dL). After initial bolus, hydrocortisone (HC) was replaced at the dose of 12.5 mg/m2/day with a provisional diagnosis of 21-hydroxylase deficiency (21-OHD) and later was gradually decreased to 5 mg/m2/day due to rapid weight gain and suppressed 17OHP levels. At the age of 10 months, he developed acne, rapid growth with penile enlargement (SPL 8.5 cm, testes 3 mL bilaterally). Testosterone levels were elevated (3.2 ng/mL) with low DHEAS and 17OHP levels. LHRH stimulation test demonstrated a GIPP (stimulated LH 7.5 IU/L (NR <15),'sH 3.9 IU/L). Brain and adrenal glands imaging were unremarkable. After increment of hydrocortisone dose, acne and genital development were ceased and testosterone was normalized (0.3 ng/mL). During follow up period, acne, rapid height gain with advanced bone age and elevated testosterone levels recurred when the hydrocortisone dose was inadequate. A repeated ACTH stimulation test (after 5 days HC suspension) at 11 years of age showed low stimulated cortisol and steroidogenic precursors suggesting AHC. Thus NR0B1 gene was sequenced and c.363delG, p.Gly122Valfs*142 novel mutation was found.

**Discussion**: Our patient presented with adrenal insufficiency with GIPP which was controlled with higher dose of hydrocortisone replacement. The low steroidogenic precursors with persistently low 17OHP during treatment led us to perform a genetic testing for AHC. The association between AHC and GIPP had been rarely described. Although the exact mechanism of premature sexual development in this condition is still unclear, there are some postulated hypotheses include high ACTH stimulated testicular steroidogenesis via melanocortin receptor type 1(MCR1) and autonomous Leydig cell hyperplasia in testes. Further studies are needed to confirm these hypotheses.

**Conclusion**: Our study extends the phenotypic and mutational spectrum of DAX-1 mutation to include peripheral precocious puberty and novel mutation. Primary adrenal insufficiency in male with low steroidogenic precursors are important diagnostic clues of AHC.

## P1-7-11 Array Comparative Genomic Hybridization and fluorescence in situ hybridization in the Diagnosis of Sex Reversal - Case Report

### Zhe Meng, Liyang Liang, Lina Zhang, Lele Hou, Zulin Liu, Xiangyang Luo, Zhanwen He, Dongfang Li

#### Department of Pediatrics, Sun Yat-Sen Memorial Hospital, Sun Yat-Sen University

**Aims**: To detect the genomic aberration in a child with disorder sexual development using the new technique of array Comparative Genomic Hybridization (aCGH) and fluorescence in situ hybridization.

**Methods**: Clinical characteristics and venous blood samples of this child were collected. Related examinations such as endocrine functions are conducted. Genome DNA was extracted and underwent fluorescence in situ hybridization and aCGH analysis.

**Results**: This child was 15 years old, with phenotype of male. His main complain was short stature and external genital dysplasia. He got penis growth and growth acceleration at 13 years old but the growth significantly slowed down at 15 years old. His voice change and pubic hair appeared at 14 years old. Physical examination showed poor scrotum development, pubic hair sparse and male distribution of PH3. Sex hormones showed'sH 44.05mIU/mL CLH 24.51mIU/mL CT 318.28ng/dl, T has no response after HCG stimulation. Testicular ultrasound showed the volume of testis was small, normal morphology and homogeneous internal echo. Karyotype analysis showed 46, XX and SRY positive. aCGH analysis showed CNV was (1) ChrXp22.33 missing, (2) ChrYp11.31 - p11.2; ChrYp11.2 repetition. Copy number of ChrYp11.2 increased with the long arm of Y chromosome missing.

**Conclusion**: This 46XX sex reversal child had copy number missing at Xp22.33 where contains ARSE gene. ARSE gene missing resulted in cartilage dysplasia and short stature. The copy number of SRY-containing ChrYp11.2 increase and the long arm of Y chromosome missing determined the social gender for this child is male. aCGH analysis confirmed that part of the sperm Y chromosome of this child’s father translocated to the end of the short arm of X chromosome during meiosis (X/SRY). FISH detection illustrated that the SRY gene translocated at the end of the short arm of X chromosome, confirming that the gender of male was determined by Yp - Xp translocation. The gene missing in the long arm of Y chromosome confirmed that this child failed to got Y gene from his father.

**Consent for publication:** The authors declare that written informed consent was obtained for publication.

## P1-7-12 A girl with congenital adrenal hyperplasia due to P450 oxidoreductase deficiency (PORD)

### Ying Liu, Xiao-bo Chen

#### Department of Endocrinology, Children's Hospital Affiliated to Capital Institute of Pediatrics

## P1-7-13 Allgrove Syndrome : Case Series

### Manpreet Sethi, Archana Dayal Arya

#### Division of Pediatric Endocrinology / Department of Pediatrics, Sir Ganga Ram Hospital

We recently worked with three patients, who presented with Allgrove Syndrome. Since only a few such cases have been reported thus far, it’s relevant to describe presentations of this rare autosomal recessive disorder, characterised by Alacrimia, Achalasia, & insensivity to ACTH.

The patients included an 8-year old boy (Case 1), an 18 month old girl (Case 2), and a 3.5 year old boy (Case 3). The primary complaint in each case was progressive darkening of complexion, while two of the three presented with hypoglycaemic seizures and easy fatigability. When queried, all the patients admitted to an absence of tears since birth. Case 2 had a history of frequent vomiting at onset. Family history for Case 1 was positive for absent tears & Achalasia in respect of an elder sibling, for which he was operated. This however was without symptoms of adrenal insufficiency. Case 2 & 3 were progeny of second-degree consanguinity. Predictably, on clinical examination, all patients were noted to have hyper-pigmentation & hypotension, two had hypoglycaemia (Case 1 & 3) & one had hyponatremia (Case 2). Lab reports likewise indicated low cortisol and high ACTH values across cases. All three patients responded favourably to hydrocortisone therapy, showing improvement in hyper-pigmentation and growth. Subsequently during follow-up, Case 1 was incidentally diagnosed at 14 years of age with Achalasia, though asymptomatic. Case 2 was diagnosed with Achalasia within a year of presentation, only when incidence of vomiting became more frequent, while in respect of Case 3, no such symptoms have presented thus far. Genetic testing was abjured due to cost constraints in each case.

Key insights from case experiences are: Adrenal insufficiency must be suspected in children presenting with hyoglycemic seizures, especially when accompanied with hyperpigmentation. History of absent tears should be ascertained in all cases of adrenal insufficiency. Save absence of tears which usually presents since birth, progression of other symptoms – Achalasia, Adrenal insufficiency maybe in any order in cases of Allgrove Syndrome.

**Consent for publication:** The authors declare that written informed consent was obtained for publication.

## P1-7-14 National Issue: Access Medicine for Congenital Adrenal Hyperplasia in Indonesia

### Syeda Tazkia (T) Noor^1^, Fia Afifah (A) Mutiksa^1^, I Wayan Bikin (B) Suryawan^2^, Aman Bhakti (B) Pulungan^2^

#### ^1^Faculty of Medicine, University of Indonesia; ^2^Indonesian Pediatric Society

**Introduction**: Most of CAH cases in Indonesia is salt losing type, resulting in a low level of cortisol and aldosterone. Cortisol is replaced by synthetic steroid treatment called hydrocortisone. However, hydrocortisone is not available in Indonesia.

**Objective**: To explain how hospital, pediatricians, and related parties resolve the unavailability of CAH medicine in Indonesia.

**Method**: This research is using a qualitative approach that is conducted on June 2016 by interviewing the head of Cipto Mangunkusumo Hospital (RSCM), pharmacy installation, Medicine distributor (Indofarma & Kimia farma), KAHAKI (the CAH club Indonesia), self hydrocortisone provider pediatrician in Malang and Semarang. The technique of data analysis is content analysis from the interview in verbatim form.

**Result**: This study shows that CAH medicine procurement in Indonesia is purchased independently because of medicine production by Indofarma is just approved by National Agency of Drugs and Foods Controls Indonesia but it will take long time to be distributed, while medicine procurement that has been done by special access Scheme (SAS) of Indonesian Ministry of Health is only available on 2014-2015. CAH medicine procurement done in several different ways. RSCM has been cooperating with medical distributors such as Indofarma (2007- 2014) and Kimia farma (2014–present). But the complex system and poor communication have made the medicine is unavailable for the last three months that has forced the patients to buy the medicine to KAHAKI themselves or they can get methylprednisolone with suited dose as an alternative. Pediatricians in Malang and Semarang buy the medicine from Australia and Amsterdam by asking help to Indonesian citizen who will return to Indonesia as a courier, or asking to the other pediatricians who have the medicine. On 2006 –2011, KAHAKI cooperate with CLAN (Carrying & Living as Neighbors) to get the hydrocortisone tablet donation but today they buy the medicine from Singapore once in three weeks. Self procurement medicine also meets some difficulties such as limited number of medicine, customs issues, also high cost.

**Conclusion**: Indonesia has difficulties to access hydrocortisone. CAH procurement in Indonesia has been done independently but not well-organized that cause limited access to this medicine. Furthermore, it is needed to produce the medicine by local pharmacy industry and the complex bureaucracy must be solved, so that CAH medicine will be available through Jaminan Kesehatan Nasional systems.

**Key words**: Congenital adrenal hyperplasia, hydrocortisone, Indonesia

## P1-7-15 Metachronous virilizing adrenocortical adenoma after 10 year follow up in 12 year old girl

### Voraluck Phatarakijnirund, Nawaporn Numbenjapon, Phairuch Chaiyakul

#### Division of Endocrinology / Department of Pediatrics, Phramongkutklao hospital and college of medicine

Adrenocortical tumor is a rare tumor in children. The most common presentation is androgen excess with or without cortisol excess and vast majority of them had unilateral tumor. However, contralateral tumor has been occasionally found. Here we report a 12-year-old girl who developed contralateral virilizing adrenocortical adenoma 10 years after resection of first tumor. The proposita is now 12 years old girl. She was first seen at age of 1.5 years with history of acne and pubic hair that had been notices 9 months before. On physical examination, she had deep voice, acnes, pubic hair, and enlarged clitoris measuring 2 cm. Breast tanner was stage 1, no growth spurt and her bone age was 24 months. Laboratory analysis showed DHEAs > 1,000 mcg/dL, testosterone 786 ng/dL, ACTH 7.2 pg/mL with loss of diurnal variation of cortisol; 8 am and midnight cortisol were 20.39 and 18.4 mcg/dL, respectively. Chromosome study was normal 46,XX. MRI showed heterogeneous enhanced right adrenal mass size 7 x 7 x 7.5 cm with internal bleeding. No evidence of liver, lung and bone metastasis were found. A right total adrenalectomy was performed and histological examination revealed an adrenocortical adenoma. Postoperatively, her clinical of androgen excess was improved. She was follow-up at regular intervals. Ten year later on 2016, she developed sign of androgen excess again, including deep voice and pubic hair tanner stage 4. Investigations reveal a markedly increase DHEAs level of 1,230 mcg/dL and testosterone level of 302 ng/dL. Other hormonal profiles were normal. A well-defined border left suprarenal mass size 3.6 x 4.7 x 4.2 cm was demonstrated by CT abdomen. No evidence of distance metastasis in liver, lung and bone. The patient underwent left total adrenalectomy and intra-operative finding showed a round mass diameter of 8 cm with no evidence of capsule invasion. Histological diagnosis of adrenocortical adenoma was made according to the modified Weiss criteria score of 2 from 7. Tissue immunohistochemistry study was positive for TP53 staining. The result of TP53 germline testing of patient and parent is pending. No postoperative complication and her DHEAs level decreased to 46 mcg/dL. She was discharge home with steroid replacement therapy. Bilateral virilizing adrenocortical adenoma is a rare condition and metachronous tumor can occur after long duration of first diagnosis. Underlying genetic cause especially TP53 mutation should be concerned and follow up of subsequent tumor is necessary.

**Consent for publication:** The authors declare that written informed consent was obtained for publication.

## P1-7-16 11β- hydroxylase deficiency due to a novel compound heterozygous mutation and literature review

### Wei Wu, Yun Li, Xiuqing Chen

#### Zhejiang University, Children's Hospital

To analyze the clinical features and CYP11B1 gene mutations of a family with 11 β -hydroxylase deficiency (11 β -OHD). Physical examination and laboratory tests were done on a 4 years old girl and gene mutation screening was conducted in her and her parents. The adrenocorticotropic hormone (ACTH), 17-hydroxyprogesterone(17-OHP) were normal and testosterone, DHEA, estradiol, serum sodium levels increased while potassium and renin levels decreased. Bilateral adrenal enlarged according to CT scan. A novel compound heterozygous mutation R453Q/R374W was found in the patient and the mother was found to carry R374W allele and the father was found to carry R453Q allele. R453Q was a missense mutation previously reported to cause the disease. R374W was a novel missense mutation that was predicted to lead to decreased 11 β -hydroxylase activity. The indicators of the patient recovered and keep normal under hydrocortisone.

## P1-8-1 Serum 25-Hydroxyvitamin D3 levels of one-month-old term infants in Tokyo using liquid chromatography tandem mass spectrometry

### Kaori Hara^1^, Kazushige Ikeda^1^, Tomonobu Hasegawa^1^, Yuhei Koyama^2^, Yasuhiro Wada^2^

#### ^1^Department of Pediatrics, Keio University school of Medicine; ^2^LSI Medience Corporation, Medical Solution segment

**Background**: 25-hydroxyvitamin D (25(OH)D), which is the major circulating form of vitamin D, is usually measured by immunoassay or competitive protein binding assay. However, recent studies have shown discrepancy among assays mainly due to cross reactivity with vitamin D metabolites. Liquid chromatography tandem mass spectrometry (LC-MS/MS) is the gold standard for the measurement of serum 25(OH)D levels characterized by (1) separating 25(OH)D3 and 25(OH)D2, and (2) separating 25(OH)D3 and other vitamin D3 metabolites. In this study, we evaluated serum 25(OH)D3 levels of term infants using LC-MS/MS.

**Subjects and methods**: 192 term infants born in Tokyo (latitude 35 degrees-north), from June, 2014 to April, 2016 were enrolled, who were originally assessed for jaundice at the age of one month. We measured serum concentration of 25(OH)D3 and 25(OH)D2 by LC-MS/MS. Serum calcium, phosphorus, alkaline phosphatase, intact PTH, 1, 25-dihydroxy- vitamin D, and total bilirubin were measured concurrently. We compared serum 25(OH)D3 level between exclusively breast-fed infants and mixed- fed by Mann-Whitney U test. We also analyzed serum 25(OH)D3 levels in each month for possible seasonal variation by Steel-Dwass test.

**Results**: 102 infants out of 192 (53.1%: 95%CI, 45.8-60.3%) had serum 25(OH)D3 levels below the lower limit of detection (4 ng/mL). None of the infants had detectable 25(OH)D2. All infants were asymptomatic and did not have hypocalcemia (ie, calcium level 3 levels compared to mixed- fed (p<0.01) (Figure). No statistical difference was observed depending on month.

**Discussion and Conclusions**: We measured serum 25(OH)D3 levels of one-month-old term infants using LC-MS/MS for the first time in Japan. Our data indicate that about half of healthy infants at one month of age in Tokyo have serum 25(OH)D3 level of less than 4ng/mL. We think that significantly lower serum 25(OH)D3 level of exclusively breast-fed is due to lower content of vitamin D3 in breast milk than formula milk. We could not find any seasonal variation of serum 25(OH)D3 levels in Tokyo.

**Fig. 1 (abstract P1-8-1).**
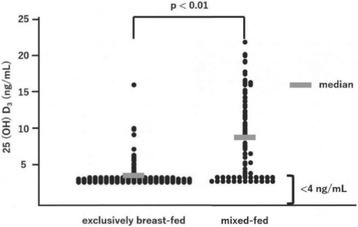
Serum 25(OH)D_3_ levels between exclusively breast-fed and mixed-fed

## P1-8-2 A novel genetic syndrome caused by dominantly-inherited ORAI1 mutations: a combination of hypoparathyroidism and tubular aggregate myopathy

### Rie Kawakita, Azumi Sakakibara, Yukiko Hashimoto, Yuki Hosokawa, Tohru Yorifuji

#### Division of Pediatric Endocrinology and Metabolism, Osaka City General Hospital

**Introduction**: Luminal Ca2+ depletion from the endoplasmic reticulum (ER) triggers activation of Ca2+ release-activated Ca2+ (CRAC) channels in the plasma membrane to slowly replenish the level of Ca2+ in ER. Dominant mutations in ORAI1 , encoding a protein composed of CRAC channels, were recently identified in several families with combined hypocalcemia and myopathy. Here we describe a boy and his mother with familial hypoparathyroidism and equinus foot deformity due to this rare genetic disorder.

**Case report**: The proband is a 14-year-old boy born to non-consanguineous Japanese parents. He developed severe hypocalcemia (serum Ca 1.37 mmol/L) shortly after birth which was treated with alfacalcidol. Hypoparathyroidism was diagnosed by serum low level of intact PTH (16 pg/mL) despite hypocalcemia (Ca 1.77mmol/L) and hyperphosphatemia (IP 2.94 mmol/L). No mutation was found in the CASR gene. His motor development had been normal until 2-3 years of age when equinus gait appeared. He was referred to our hospital at 8 years of age for elevated serum creatine kinase (CK) of more than 2000 IU/L. Brain and spinal MRI were not remarkable, but brain CT revealed ectopic calcification in the subcortical white matter. His gait abnormality has been slowly progressive. At age 13 years, he is still ambulatory with mild mental retardation. His mother also had a clinical history of hypoparathyroidism, diabetes, muscle weakness and equinus contractures on both feet. She was diagnosed with tubular aggregate myopathy at 45 year of age. Whole exome sequencing of the family members revealed a heterozygous c.292G>A (G98S) mutation in the ORAI1 gene.

**Discussion**: Endo et al. reported that the CRAC channel is constitutively activated by the heterozygous ORAI1 gain-of-function mutations so that Ca2+ influx from the extracellular space to the cytoplasm persists irrespective of Ca2+ stores in ER. Hypoparathyroidism was present in all patients with a heterozygous c.292G>A mutation in ORAI1 identified to date. Although further elucidation of pathogenic mechanisms about disease phenotypes associated with mutations in ORAI1 is needed, we should take ORAI1 genetic testing into consideration in patients who displayed hypocalcemia and muscle weakness accompanied by increased serum CK levels and/or joint contractures.

**Acknowledgement**: We thank Dr. Satoru Noguchi (National Center of Neurology and Psychiatry) for assistance in DNA sequencing.

## P1-8-3 Beckwith-Wiedemann syndrome and pseudohypoparathyroidism type Ib in a patient with multilocus imprinting disturbance

### Shinichiro Sano^1,2,3^, Keiko Matsubara^2^, Keisuke Nagasaki^4^, Toru Kikuchi^5^, Kazuhiko Nakabayashi^6^, Kenichiro Hata^6^, Maki Fukami^2^, Masayo Kagami^2^, Tsutomu Ogata^2,3^

#### ^1^Department of Pediatrics, Hamamatsu Medical Center; ^2^Department of Molecular Endocrinology, National Research Institute for Child Health and Development; ^3^Department of Pediatrics, Hamamatsu University School of Medicine; ^4^Department of Homeostatic Regulation and Development, Niigata University Graduate School of Medical and Dental Sciences, Division of Pediatrics; ^5^Department of Pediatrics, Saitama Medical University; ^6^Department of Maternal-Fetal Biology, National Research Institute for Child Health and Development

Recent studies have identified multilocus imprinting disturbance (MLID) with hypo- or hyper-methylated at differentially methylated regions (DMRs) in a subset of patients with imprinting disorders (IDs). However, most patients exhibit clinical features of the original ID only. Here, we report a 14-year-old Japanese female patient diagnosed as having Beckwith-Wiedemann syndrome (macroglossia, umbilical hernia, overgrowth, and transient neonatal hypoglycemia) and pseudohypoparathyroidism type Ib (PTH resistance without Albright’s hereditary osteodystrophy). Molecular studies revealed marked hypomethylation at the Kv -DMR, A/B -DMR, XLas -DMR, AS -DMR and hypermethylation at the NESP55 -DMR, and variable methylation defects at four additional DMRs, in the absence of copy number alteration and uniparental disomy of the chromosomes harboring such DMRs. No mutation was found in CDKN1C and GNAS , as well as in genes known to be associated with MLID such as ZFP57 , NLRP2 , NLRP7 , KHDC3L , and NLRP5 . It is likely that the methylation defects at the Kv -DMR and the GNAS -DMRs (A/B -DMR, XLas-DMR, AS -DMR, and NESP55 -DMR) were sufficient to cause clinically recognizable IDs, whereas the remaining methylation defects were insufficient to result in clinical consequences or took place at DMRs with no disease-causing imprinted gene(s). The development of MLID and the two IDs of this patient may be due to a mutation in a hitherto unknown gene for MLID, or to a reduced amount of DNA methyltransferase-1 (DNMT1) available for the methylation maintenance of DMRs because of the consumption of DNMT1 by the maintenance of X-inactivation. In support of the latter notion, co-existence of two IDs has primarily been identified in female patients, and MLID has predominantly been identified as loss of methylations.

**Consent for publication:** The authors declare that written informed consent was obtained for publication.

## P1-8-4 Genotype-phenotype correlation in six cases of hypophosphatasia - c. 1559delT mutation in ALPL presents heterogeneous phenotypes

### Junko Kanno^1^, Sayaka Kawashima^1^, Chisumi Sogi^1^, Miki Kamimura^1^, Toshimi Michigami^2^, Shigeo Kure^1^, Ikuma Fujiwara^3^

#### ^1^Department of Pediatrics, Tohoku University school of Medicine; ^2^Department of Bone and Mineral Research, Osaka Medical Center and Research Institute for Maternal and Child Health; ^3^Department of Pediatric Endocrinology and Environmental Medicine, Tohoku University school of Medicine

**Introduction**: Hypophosphatasia (HPP) is an inherited metabolic disease characterized by impaired bone mineralization due to decreased tissue nonspecific alkaline phosphatase (TNSALP) activity. HPP is caused by loss-of-function mutations in the ALPL encoding TNSALP. Enzyme replacement therapy (ERT) for HPP was approved recently, and severe patients can be saved. We present manifestations and genetic backgrounds of 6 HPP cases.

**Case 1**: A neonate with no family history of bone disease was pointed out lower limb shortening in utero. He showed a mild dyspnea and tonic convulsions soon after birth. His serum ALP was extremely low (16 IU/l) and radiograph of cranium and long bones showed hypomineralization. Sequence analysis of ALPL revealed homozygous mutation of c.1559delT. Initiating ERT has significantly improved skeletal mineralization.

**Case 2**: A 2-year-old girl was suspected for osteogenesis imperfecta due to shortening and flexura of limbs in utero. She had no breathing problems after birth, and skeletal mineralization defect and low serum ALP (27 IU/l) were recognized. She was a compound heterozygote of c.1559delT and p.E191G. On 5 months ERT was started and the improvement of bowing of long bones were remarkable.

**Case 3**: An 8-year-old boy was a brother of case 2. He lost 6 deciduous teeth by 3-4 years of age. ALP was 136 IU/l. He was a compound heterozygote of c.1559delT and p.E191G as his sister, though he experienced no deformity or fracture.

**Case 4**: A 3 year-old girl lost seven deciduous teeth by 3 years of age with no other symptoms. ALP was 255 IU/l, bone mineral density was low. She was a compound heterozygote of c.1559delT and p.F310L.

**Case 5**: A 1-month- old boy was hospitalized for convulsion. ALP was low (140 IU/l) and his limbs showed a slight ossification defect radiographycally. He was a heterozygote of p.M62I, and his father shared the same mutation.

**Case 6**: A 6-year-old girl was a triplet and was diagnosed as HPP by low serum ALP (150 IU/l) and high urinary phosphoethanolamine levels during NICU hospitalization. Her metaphysis was slightly irregular radiographycally and bone mineral density was low.

**Discussion**: The symptoms of HPP are various including those who carry homozygous c.1559delT mutation, and even in siblings who have the same mutations. Since severity of HPP is wide-ranging, it is important to accumulate experience of ERT and to consider indication of ERT for HPP patients.

## P1-8-5 The effects of whole-body vibration therapy on physical function, bone and muscle mass in adolescents with mild to moderate musculoskeletal disability

### Silmara Gusso, Patricia Colle, Janene Biggs, Jose Derraik, Paul L Hofman

#### University of Auckland, Liggins Institute

**Background**: Adolescents with musculoskeletal disability have decreased mobility resulting in reduced bone and muscle mass. There is a paucity of non-invasive therapeutic interventions aimed at increasing muscle mass and muscle function, as well as bone health in this population. We aimed to evaluate the effect of 20 weeks of whole-body vibration therapy (WBVT) on muscle function and bone health of adolescents with musculoskeletal disability.

**Methods**: 24 adolescents (50% females) aged 16.4 ± 2.3 years with mild to moderate musculoskeletal disability (Gross Motor Function Classification System II and III) underwent WBVT 9 minutes per day, 4x per week, for 20 weeks on a Galileo platform. Clinical assessments were performed at baseline and after 20 weeks, including 6-minute walk test, whole-body dual-energy X-ray absorptiometry, peripheral quantitative computed tomography of the non-dominant tibia, as well as muscle force and power using a ground reaction force plate.

**Results**: WBVT increased lean mass overall (+991 g; p=0.004). There were also consistent improvements in bone mass, with bone mineral content increasing in whole body (+43 g; p=0.019) and spine L1-L4 (+2 g; p=0.001). Similarly, bone mineral density also increased in spine L1-L4 (+0.023 g/cm2; p=0.030). Participants reduced the time taken to perform the chair test (-1.2 seconds; p<0.001), improved the distance covered in the 6-minute walk test (+40 m; p<0.001), and improved balance on standing position (-1.7 cm2 in standing ellipse area; p=0.004).

**Conclusion**: 20 weeks of whole-body vibration therapy led to increases in muscle and bone mass, while also improving mobility, having therefore a positive effect on the health and well-being of children with musculoskeletal disability.

## P1-8-6 Fanconi syndrome in a patient with McCune-Albright syndrome

### Taisuke Nogayama^1^, Hironori Shibata^1^, Kazuya Matsumura^1^, Hiroki Matsuura^2^, Yosuke Hara^2^, Tomoki Kosho^3^, Satoshi Narumi^1^, Tomohiro Ishii^1^, Midori Asazu^1^, Tomonobu Hasegawa^1^

#### ^1^Department of Pediatrics, Keio University School of Medicine; ^2^Department of Pediatrics, Shinshu University School of Medicine; ^3^Division of Clinical and Molecular Genetics, Shinshu University Hospital

## P1-8-7 Clinical assessment of hypercalciuria and hypomagnesemia in patients with Bartter syndrome and Gitelman syndrome

### Wenjing Li, Chunxiu Gong, Chang Su, Bingyan Cao, Di Wu, Min Liu, Xuejun Liang

#### Department of Endocrinology, Genetics and Metabolism, Beijing Children's Hospital, Capital Medical University

**Objective**: Bartter syndrome (BS) and Gitelman syndrome (GS) have similar clinical manifestations. It’s hard to be distinguished by the symptoms and laboratory, even though the genetic analysis. The study was based on the analysis of clinical data of 72 patients of BS and GS, and tries to find some useful parameters to help to differentiate diagnose.

**Methods**: To summarize the clinical data, and to analyze the correlation between urinary calcium, blood magnesium and clinical symptoms. Result: 72 cases patients aged from 2 months to 15.5 years (median 1.75years), sex ratio of boy: girl is 52:20. All patients had hypokalemia, metabolic alkalosis, normal blood pressure and the levels of plasma rennin, angiontension and aldosterone elevated. The ratio of urine calcium/creatinine over 0.2 was considered as a mark of hypercalciuria. The age of hypercalciuria group was 2.64 ± 2.95 yrs old, and that of ratio < 0.2 was 8.26 ± 4.49 yrs (P = 0.00). The SDS of weight was -2.58 ± 1.11 and -1.59 ± 1.26 respectively (P < 0.005). The age of patients with hypomagnesemia( serum magnesium < 0.8mmol/l ) was 7.88 ± 4.47 yrs Cand the age of normal serum magnesium was 3.78 ± 4.14 yrs (P = 0.001), the SDS of weight were -2.42 ± 1.60 and -1.42 ± 1.13 (P = 0.005). There was no significant difference between the two groups with the ratio of urine calcium/creatinine. Correlation analysis showed that urinary calcium/creatinine ratio positive correlated with serum magnesium (R =0.355, P= 0.008)

**Conclusion**: The patients with hypercalciuria were younger than that of normal urine calcium, and coincidently with poor nourished and growth. The GS patients with hypomagnesemia usually had mild symptoms. The clinical types and genotypes of BS and GS were often overlapped. The parameters of hypercalciuria and hypomagnesemia were useful in the differential diagnosis of BS and GS.

## P1-8-8 Comparison of bone health parameters in overweight/ obese and normal weight adolescents

### Alec Correa^1^, Manisha Jana^2^, Vandana Jain^1^

#### ^1^Division of Endocrinology / Department of Pediatrics, All India Institute of Medical Sciences; ^2^Department of Radiology, All India Institute of Medical Sciences

**Introduction**: Fat has a complex interaction with bone though adipokines and inflammatory mediators. This study was conducted to evaluate the relation between obesity and bone health in adolescents.

**Methods**: This cross sectional study was conducted at All India Institute of Medical Sciences, Delhi. Healthy adolescents between 10 and 16 years of age with BMI above 85th percentile on Indian charts were recruited as cases (overweight/obese group, n=48), and those with BMI between 10th-75th percentile were recruited as controls (*n*=29). Bone mineral density (BMD) and concentration (BMC), and body fat was measured by whole body dual energy X-ray absorptiometry (DEXA, Discovery, Hologic Inc, USA). Bone speed of sound (SOS, a measure of structural quality as well as BMD) of distal radius and mid tibia was measured by quantitative ultrasonography (Sunlight Omnisense, Beam Med Ltd, Israel). Serum 25OHD, leptin, adiponectin and osteoprotegerin were measured.

**Results**: Comparison of age, BMI SDS, biochemical parameters, SOS and DXA of the overweight/obese group and normal group is presented in Table 1. While bone SOS was significantly higher in the normal weight group, BMC and BMD were higher in the overweight/ obese group for subtotal body (total minus head), thoracic spine and limbs. In overweight/obese group, total body fat was negatively correlated with lumbar spine (r -0.38, p 0.02) and pelvis BMC (r -0.34, p 0.04); osteoprotegerin was negatively correlated with subtotal BMD (r -0.35, p 0.03); leptin was positively correlated with whole body BMD (rho 0.37, p 0.02) and radius SOS(rho 0.3, p 0.03) and adiponectin was negatively correlated with tibia SOS z-score(rho-0.33, p 0.02). In normal weight group, BMI SDS was positively correlated with total body (r 0.52, *p* <0.01) as well as regional BMC and BMD.

**Conclusion**: Bone ultrasound and DXA showed contrasting results, with overweight/obese group having lower SOS but higher BMD, suggesting that the bone may be structurally of poorer quality in the overweight/obese. Some degree of adiposity may benefit, while excessive fat my adversely affect bone health. Bone health parameters correlated positively with leptin and negatively with osteoprotegerin and adiponectin.

**Table 1 (abstract P1-8-8).**
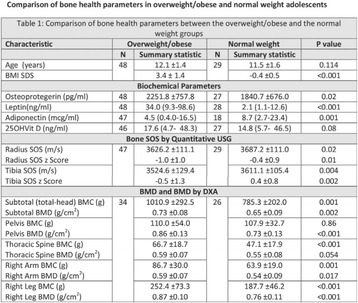
Comparison of bone health parameters in overweight/obese and normal weight adolescents

## P1-8-9 The third Japanese case of hypomagnesemia with secondary hypocalcemia

### Yusuke Mizuno^1^, Satoshi Narumi^1,2^, Keisuke Nagasaki^3^, Tomonobu Hasegawa^1^

#### ^1^Keio University School of Medicine, Department of Pediatrics; ^2^Departments of Molecular Endocrinology, National Research Institute for Child Health and Development; ^3^Division of Pediatrics, Department of Homeostatic Regulation and Development, Niigata University Graduate School of Medical and Dental Sciences

## P1-8-10 Vitamin D levels in children with Hashimoto's thyroiditis: before and after l-thyroxine therapy

### Rakesh Kumar^1^, Navendu Chaudhary^1^, Devi Dayal^1^, Naresh Sachdeva^2^

#### ^1^Department of Paediatrics, Post Graduate Institute of Medical Education and Research, Chandigarh; ^2^Department of Endocrinology, Post Graduate Institute of Medical Education and Research, Chandigarh

**Background**: There is high prevalence of Vitamin D deficiency (VDD) in Hashimoto’s thyroiditis (HT) as reported in literature. However, it is uncertain whether VDD is a cause or effect of HT. The effect L- thyroxine replacement on vitamin D levels in children with HT has not been studied.

**Objective and hypotheses:** To study vitamin D level of newly diagnosed children with HT and to observe the change in vitamin D level after L-thyroxine therapy.

**Method**: A prospective observational study was conducted on 35 consecutive children (less than 12years old) who were newly diagnosed with HT, and not yet started on L-thyroxine, over a one year study period. Children with concomitant chronic kidney/liver disease, Celiac disease, on anti-tubercular therapy/antiepileptics or glucocorticoids and who received vitamin D supplements in last 6 months were excluded. Serum 25 (OH) D levels was estimated before starting L-thyroxine and on follow-up. Vitamin D levels were compared with historical controls (from a study from same centre on healthy children of similar age and epidemiological profile).

**Results**: The mean vitamin D level in cases at diagnosis was significantly low as compared to controls (33.34 ± 16.93 nmol/L Vs 65.13 ± 30.57 nmol/L; p value<0.0001). Out of 22 Vitamin D deficient children with HT, who received vitamin D therapy, 7 (31.8%) remained deficient even at follow-up. Thirteen patients (with sufficient/insufficient vitamin D levels) who were not supplemented with vitamin D had a fall in vitamin D levels in follow-up. However, degree of fall in Vitamin D was not statistically significant.

**Conclusion**: Children with HT were observed to have low vitamin D levels at diagnosis and L-thyroxine therapy lead to further compromise in vitamin D levels.

## P1-8-11 Efficacy of monthly alendronate infusion for osteogenesis imperfecta

### Ikuma Fujiwara^1,2^, Chisumi Sogi^1^, Sayaka Kawashima^1^, Miki Kamimura^1^, Junko Kanno^1^

#### ^1^Tohoku University Hospital, Department of Pediatrics; ^2^Department of Pediatric Endocrinology and Environmental Medicine, Tohoku University Graduate School of Medicine

**Background**: Osteogenesis imperfecta (OI) is a heritable skeletal disease mostly caused by mutations in either COL1A1 or COL1A2 that encode type I collagen. Treatment with bisphosphonate (BP) for OI patients has been successfully performed worldwide. Of these BPs, pamidronate infusion is commonly used, and administration of zoledronic acid biannually has been reported recently. But there are some concerns about using a potent BP with a longer treatment interval for children or a BP with large infusion volume for infants.

**Method**: Nine patients with OI (5 type I, 3 type III, 1 type IV; ages from 0 to 12 years, average 5 years) were treated with monthly alendronate (ALN) infusion with a dose of 18μg/kg (2ml/kg) over 1 hour. Four of the patients received previous therapy. Oral calcium was supplemented for a week after ALN infusion. Lumbar bone mineral density (BMD) was measured by DXA every 6 months.

**Results**: Six months of ALN treatment increased BMD by 15.5 % from baseline in average. During that period, 4 fractures were reported. Febrile episode was seen in two patients after the initial infusion of ALN. Respiratory distress occurred in a type IIb/III infant after the third ALN infusion. Symptoms related hypocalcemia such as seizure were not reported during the treatment.

**Discussion**: ALN infusion is used for adult osteoporosis patients with a dose of 900μg. In this study, the initial dose of 18μg (2ml)/kg was selected with assumption of adult BW was 50 kg. The dose 2 mls per kg body weight of ALN would be safe and well tolerated, and could be enough to increase BMD in OI patients. But in severe patients, increase of the dose may be necessary.

**Conclusion**: Monthly ALN infusion may be safe and efficacious for OI even in younger age.

## P1-8-12 vitamin D-dependent rickets type 1 caused by a novel CYP27B1 mutation

### Jin-Kyung Kim^1^, Chang-Ho Lee^1^, Ji-Hyun Na^1^, Ja-Hyun Jang^2^

#### ^1^Department of Pediatrics; ^2^Green Cross Genome, Catholic University of Daegu School of Medicine

Vitamin D-dependent rickets type I (VDDR1) is a rare autosomal recessive disorder caused by mutations in the 25-hydroxyvitamin D 1- α-hydroxylase gene (CYP27B1). Mutations in CYP27B1 disrupt or lead to a loss of the 1- α -hydroxylase activity. 1a-OHase deficiency impedes the activation of vitamin D. We present a case of 20 months-old boy presented with failure to thrive (9.5kg, <3percentile), 76.5cm, <3percentile), and inability to walk. Frontal bossing, delay in closure of anterior fontanelle, rachitic rosary and widening of wrists were noted on physical examination. Laboratory findings are hypocalcaemia (calcium: 5.8 mg/dL), elevated serum levels of alkaline phosphatase (2509 U/L) and intact-parathyroid hormone (238 pg/mL) with low level of 1,25(OH)2 D3 (11.2 pg/mL) despite normal concentrations of 25(OH)D3 (38.6 ng/mL). Radiograph of the wrist and hand showed classic sign of rickets with widening, cupping, and fraying of the distal radius and ulna metaphyses with an associated increase in the thickness of the growth plate. CYP27B1 was analyzed by sequencing of the entire coding region, and 2 novel mutations in exon 1 were found. Both parents are heterozygous carriers of the mutation. A heterozygous c.57_69del (p.Glu20Profs*2) mutation led to the substitution of a glutamic acid by a proline on amino acid position 20 and a second heterozygous c.171dupG (p.Leu58Alafs*275) duplication mutation led to the substitution of a leucine by a alanine on amino acid position 58. Both mutations result in a frameshift and the creation of premature stop codons. After treatment with calcitriol and supplemental calcium, his biochemical and clinical improvement and catch up growth were observed.

## P1-8-13 An ectopic parathyroid adenoma associated with hypercalcemia in a child

### Hidechika Morimoto^1^, Hisakazu Nakajima^1^, Shota Fukuhara^1^, Keiichi Shigehara^1^, Kazuki Kodo^1^, Ikuyo Itoh^1^, Jun Mori^1^, Hajime Hosoi^1^, Shigehisa Fumino^2^, Taizo Furukawa^2^, Tatsuo Tajiri^2^, Sanae Kagayama^3^, Shinsuke Adachi^3^

#### ^1^Department of Pediatrics, Kyoto Prefectural University of Medicine; ^2^Department of Pediatric Surgery, Kyoto Prefectural University of Medicine; ^3^Department of Pediatrics, Fukuchiyama City Hospital

Primary hyperparathyroidism in children is very rare. We report a pediatric case of an ectopic parathyroid adenoma. The patient was a 13-year-old boy who developed abdominal pain and hematuria in June 2014. Ultrasonography at another hospital revealed hydronephrosis, and computed tomography (CT) revealed a ureteral calculus. Blood tests showed elevated levels of serum calcium (12.1mg/dL) and intact parathyroid hormone (PTH) (102 pg/mL). 99mTc-MIBI single photon emission computed tomography and 3-dimensional contrast-enhanced CT revealed a small tumor in the superior anterior mediastinum. We diagnosed ectopic primary hyperparathyroidism. Cinacalcet was administered, after which, serum calcium levels decreased, and abdominal pain and hematuria remitted. However, the patient had persistent nausea, a side effect of cinacalcet. Antiemetic drugs were needed for treating the nausea until surgery. In March 2015, thoracoscopic resection was successfully performed for an ectopic parathyroid tumor (adenoma), which was intraoperatively detected in the thymus by using a gamma-ray detection probe. We also monitored serum intact PTH levels before and after resection to ensure adequate tumor resection; the level decreased from 154 pg/mL to 22 pg/mL. Serum calcium and intact PTH levels have remained within the normal ranges, with no apparent recurrence. When an ectopic parathyroid adenoma associated with hypercalcemia is detected, tumor resection is the first choice for treatment. Cinacalcet effectively decreased the serum levels of calcium and intact PTH and prevented the manifestations of hypercalcemia in our case. This drug is useful as a bridge treatment during preparation for surgery of an ectopic parathyroid adenoma.

**Consent for publication:** The authors declare that written informed consent was obtained for publication.

## P1-8-14 Evaluation of 3-year FOSTEO (Forum Osteogenesis Imperfecta) Indonesia

### Mentari Syarifuddin^1^, Trivanie S Elona^1^, Pulungan B Aman^1,2,3^

#### ^1^Universitas Indonesia, Faculty of Medicine; ^2^Cipto Mangukusumo Hospital - Faculty of Medicine, Universitas Indonesia, Endocrinology Division / Department of Health Child; ^3^Indonesian Pediatric Society

**Background**: FOSTEO (Forum Osteogenesis Imperfecta) was established on November 18th 2013 replacing PERKOI (Perkumpulan Keluarga (penderita) Osteogenesis Imperfecta) and cooperates with CLAN, as a community for osteogenesis imperfecta (OI) family in Indonesia to share each other. Since establishment until now, FOSTEO had given large impact to the members and Indonesian people. This study aims to evaluate the performance of FOSTEO.

**Methods**: A Study was conducted in May-June 2016 involving 126 members of FOSTEO and OI patients in Indonesia. Data for cross-sectional study were based on FOSTEO evaluation online questionnaire, patient/guardian interviews as members of FOSTEO, doctors, and other parties who involved in management of OI in Indonesia. FOSTEO members who did not fill questionnaire completely or were unreachable via WhatsApp group and phone call would be dropped out.

**Results**: From 126 OI patients in registry data, 49 patients (38.89%) filled the quetionnaire, 48 patients (38.09%) were lost contact, 12 patients (9.52%) did not answer phone calls, 8 patients (6.35%) died and 9 patients (7.14%) were not members of FOSTEO. Among 49 patients’ guardians who filled the questionnaire and were interviewed, found that most of patients were children 2-4 years old (32.65%). Most of respondents said that FOSTEO was helpful for them (85.71%) and for Indonesia (79.59%). The advantages mentioned such as they found a new family, not feel alone anymore, shared information, arranged treatment schedule, able to do drugs sharing, and available wards searching. Fifty one percent members of FOSTEO could not attend FOSTEO regular events or programs because of the distance, and 36.73% respondents said that the events were not held on schedule. Some provinces/areas such as Central Java, Makassar and Papua, provincial community do not have formal representatives, thus routine events and information delivery were not done equally. This study also found that most respondents agree that treatment management was easy, medications and wards were available, distance between home to hospital was not an obstacle, health insurance was helpful, and doctors were competent. However, 19 respondents said government had not showed any concern to OI patients, and 32 respondents stated that primary health care physicians could not detect OI, although FOSTEO already held wishbone day every year.

**Conclusion**: FOSTEO gave huge benefits to its members and Indonesian people. Hopefully, FOSTEO could be better in giving information about osteogenesis imperfecta to increase awareness of Indonesian people about this non-communicable disease.

**Keyword**: FOSTEO evaluation, osteogenesis impefecta, Indonesia, wishbone day

## P1-8-15 A novel COL10A1 mutation in a Metaphyseal chondrodysplasia, Schmid type case presented with an atypical course of clinically diagnosed vitamin D deficient rickets

### Hiroyuki Tanaka^1^, Mayuko Tamura^1^, Chie Takahashi^1^, Tsuyoshi Isojima^1^, Nobuhiko Haga^2^, Akira Oka^1^, Sachiko Kitanaka^1^

#### ^1^Department of Pediatrics, Graduate School of Medicine, the University of Tokyo; ^2^Department of Rehabilitation Medicine, Graduate School of Medicine, the University of Tokyo

**Background**: Metaphyseal chondrodysplasia Schmid type (MCDS) is an autosomal dominant inherited disorder caused by mutations in COL10A1 gene. MCDS is characterized by bow-legs, short stature, coxa vara, and normal facial appearance. The symptoms and the radiographic findings are similar to those of rickets. The increasing incidence of vitamin D-deficient rickets sometimes leads us to overlook the possibility of MCDS among children with bow-legs.

**Case**: This 3-year-old boy was born at 38 weeks to non-consanguineous healthy parents. His birth weight and length were 2808g and 50.0cm, respectively. At 16 months of age, he was referred to the previous institution because of bow-legs with short stature (-2.0SD). He was breastfed but spent outdoors naturally. Radiography revealed irregular metaphysis consistent with rickets. Blood tests showed slightly elevated ALP (1,366 U/mL) and low 25(OH)D levels (19.9 ng/mL), and normal levels of Ca (10.6 mg/dL), IP (4.8 mg/dL), intact PTH (33 pg/ mL), FGF23 (29 pg/mL) and 1,25(OH)2D (66 pg/mL). Since vitamin D-deficiency could not be ruled out, he was treated with oral vitamin D supplement. His ALP level decreased to 845 U/mL, but his radiographic abnormalities and bow-legs were not improved. He was referred to our institution for further investigation. At age 3 years, his height SD level decreased to -2.6SD (88.2cm). Bow-legs, wide gait, and enlargement of the joints were observed. Laboratory tests showed normal ALP, Ca, IP, PTH levels, and 25(OH)D level of 16 ng/mL. From those data and the treatment course, we suspected of MCDS.

Genetic analysis: The genetic analysis of COL10A1 gene was performed with informed consent and the approval of the Ethics Committee. It revealed a novel heterozygous mutation, c.2015T>C(p.F672S). This mutation was not found in his parents, indicating a de novo mutation. It was not found in dbSNP. The amino acid is well conserved among various species. In Silico analyses predicted this mutation as disease causing.

**Discussion**: We found a novel de novo COL10A1 mutation in a boy who had been treated as rickets with atypical clinical course. The mutated amino acid was located in the C-terminal non-collagenous (NC1) domain that is required for collagen trimer assembly. The reported COL10A1 mutations in MCDS are predominantly clustered in the NC1 domain. Considering together with the in Silico analyses, p.F672S might be the causative mutation for MCDS in this patient. In case of atypical clinical or therapeutic course rickets, COL10A1 gene analysis would be useful to distinguish MCDS from vitamin D-deficient rickets.

**Consent for publication:** The authors declare that written informed consent was obtained for publication.

## P1-8-16 Locally Manufactured Telescopic Rods in Treatment of Osteogenisis Imperfecta Cost Effective Approach for Developing Country

### Nasir Saleem Saddal, Jamal Raza

#### Department of Pediatric Surgery, National Institute of Child Health Karachi

**Aims of study**: Osteogenesis imperfecta (OI) is an inherited disorder of connective tissue characterized by bones fragility. Bisphosphonate therapy and surgical interventions are two main treatment options. For surgical intervention intramedullary rods are accepted mode of treatment. The standard implant cost is more than 2000 US dollars including the shipment cost also which is out of reach of patients from developing world. We are using locally manufactured implant made of medical grade steel in OI. The purpose of this study was to find out implant safety and quality of life in terms of ambulation and socialization.

**Methodology**: This descriptive case series was conducted at Paediatric Surgical department of National Institute of Child Health Karachi, Pakistan over a period of two years from 2013 to 2015. Material used was locally manufactured telescopic rods made of internationally specified medical grade steel. Standard technique as described by Sofield-Miller was used. All patients were also receiving biphosphonates. Patients were kept in hospital overnight and then followed up in Outpatient at regular intervals. Clinical and radiological evaluation was done. Ambulatory status were also analyzed. Minimum follow up was of three months and maximum of twelve months. Descriptive statistics were used like frequencies and numbers to present data.

**Results**: Total number of patients operated was twelve. A total of 19 long bones were operated in these patients. All patients had moderate and severe form of OI with history of multiple fractures. There were 7 males and five females. The age ranged from 4 year to 14 year with mean age of + 8.5 year. This included eight femora and eleven tibia. Number of femora operated was 08 and tibia in 11 patients. There was no early complication in all the patients and hospital stay was 24 to 48 hours. Pain was the only complaint in early period. Other complications found were migration of rod in three patients, fracture post trauma in one patient and bending of rod in one patient. All of these complication were already been described internationally .The cost of the locally manufactures rod is approximately 90 US dollars.

**Conclusion**: The ambulatory status improved after the telescopic rods surgeries, especially when it was associated with intravenous administration of biphosphonates. Locally manufactured rods are as effective as international rods. There is a huge cost saving. The use of locally manufactured rods allowing many affected children to be benefit.

## P1-8-17 A case of Bartter syndrome with severe failure to thrive and electrolyte abnormalities associated with cholelithiasis

### Yuichi Mushimoto^1^, Shuichi Suzuki^1,2^, Sayo Mori^1^, Yoshinori Koga^3^, Yoshio Zaizen^3^, Kenichi Miyako^1^

#### ^1^Department of Endocrinology and Metabolism, Fukuoka Children's Hospital; ^2^Department of Pediatrics, Self Defense Forces Fukuoka Hospital; ^3^Department of Pediatric Surgery, Fukuoka Children's Hospital

**Background**: Bartter syndrome is a disorder characterized by hypokalemic metabolic alkalosis with hypercalciuria and salt wasting. Bartter syndrome and cholelithiasis are respectively very rare diseases in infants. However, a few studies have reported an association between these two conditions. We report a case of Bartter syndrome associated with cholelithiasis in an infant.

**Patient case**: A 3-month-old girl with a genetic diagnosis of Bartter syndrome type 3 was receiving high-volume electrolyte supplementation in addition to indomethacin and spironolactone. An ultrasound examination performed to screen for the cause of failure to thrive unexpectedly revealed asymptomatic gallstones, which were followed up with no treatment. At the age of 11 months, the patient was admitted because of vomiting and electrolyte abnormalities. She had poor growth for her age (length: 62.6 cm; -3.93 SD, weight: 5.2 kg; BMI- SDS -2.46 SD). Serum laboratory values were as follows: WBC 13,120/μL, CRP 0.35 mg/dL, Na 134 mEq/L, K 2.4 mEq/L, urinary Na 60 mEq/L, urinary K 154.3 mEq/L, AST 300 IU/L, ALT 154 IU/L, T-Bil 0.9 mg/dL, pH 7.431, HCO3- 33.8 mmol/L, and BE 8.1 mmol/L. Intravenous rehydration and electrolyte supplementation improved the electrolyte abnormalities, while vomiting persisted. On the 12th hospital day, she had discolored stool, and an abdominal ultrasound examination disclosed bile duct stones associated with biliary dilatation. Gallstone dissolution therapy with ursodeoxycholic acid was begun and led to spontaneous evacuation of the gallstones after 11 days of treatment. Calculus analysis showed that she had a calcium phosphate stone, not a bilirubin stone, which is more common in infants.

**Conclusion**: The predisposing factors for the formation of gallstones in neonates and infants include prematurity, necrotizing enterocolitis, total parenteral nutrition, starvation, dehydration, and treatment with ceftriaxone or furosemide. Bartter syndrome typically presents with dehydration and urinary electrolyte loss similar to that produced by furosemide. Therefore, patients with Bartter syndrome might be at risk for cholelithiasis. Abdominal ultrasound screening for cholelithiasis should be performed in patients with Bartter syndrome who present with repeated vomiting without an obvious cause.

**Consent for publication:** The authors declare that written informed consent was obtained for publication.

## P1-8-18 Osteogenesis Imperfecta: an institutional review from South India

### Leena Priyambada, Sarah Mathai, Georgie Mathew, Anna Simon

#### Division of Paediatric Endocrinology / Department of Paediatrics, Christian Medical College

**Aims**: To describe the clinical profile of children with Osteogenesis Imperfecta (OI)

**Methods**: Retrospective chart review of children with OI who were treated by the Paediatric Endocrine division of Christian Medical College, Vellore between 2009-2016.

**Treatment protocol:** Pamidronate is the only bisphosphonate used. Children receive 8-9 mg/kg/year during the first year and 4-5 mg/kg/year during the subsequent years of treatment. Infants are commenced on Pamidronate at 4 weeks of age when diagnosed antenatally. During infancy, Pamidronate injections are given every 2 months till 6 months and thereafter every 3 months. Older children are given injections every 3-4 months. After the infusion calcium and calcitriol are supplemented for 7 days. Treatment outcomes observed are reduction in fracture frequency and bone pain as well as improvement in ambulation. Improvement in DEXA BMD z scores are compared annually with the each child’s baseline BMD Z scores.

**Results**: Data of 27 (15 males) children aged 0-16 years were included. Of the 10 infants in the group, 3 were neonates who were diagnosed antenatally. Almost half the children had OI type-3 (severe), 24% had OI type-4, and 20% had OI type-1. Blue sclera was seen in 78% of children, and 28% had dentinogenesis imperfecta. Baseline mean LS spine BMD z score was -4.31. The mean follow up duration was 34.8 (0-103) months. Antenatally diagnosed infants received 8.2 mg/kg in the first year. Older children received a mean of 4.98+2.74 mg/kg of pamidronate, and 2.54 cycles in the first year. The main reasons for delay in the injection cycles were due to fractures and osteotomies. Bone pain, ambulation, motor development and fracture frequency improved. Sixteen (57%) children were fracture free in the 1st year of treatment. Flu-like symptoms was seen only in 2, and hypocalcemia was seen in 3 of all the pamidronate infusions.

**Conclusion**: Pamidronate is a safe and effective therapy for children with OI and improves clinical symptoms and morbidity. Neonates with severe OI should be started early on Pamidronate. Optimum doses of Pamidronate could not be achieved in many due to fractures and surgical procedures.

## P1-8-19 Myhre syndrome: a progressive connective tissue disorder with short stature

### Risa Nomura^1,2^, Maki Gau^1^, Takeru Yamauchi^1,2^, Akito Sutani^1^, Atsumi Tsuji-Hosokawa^1^, Yohei Matsubara^1^, Kentaro Miyai^1,3^, Susumu Hosokawa^1^, Gen Nishimura^4^, Kenichi Kashimada^1^

#### ^1^Department of Pediatrics and Developmental Biology, Graduate School, Tokyo Medical and Dental University; ^2^Department of Pediatrics, Tsuchiura Kyodo General Hospital; ^3^Department of Endocrinology and Metabolism, Tokyo Metropolitan Children's Medical Center; ^4^Department of Pediatric Imaging, Tokyo Metropolitan Children's Medical Center

Myhre syndrome (MS) is a malformation syndrome that mainly affects connective tissues. The clinical hallmarks include mental and growth deficiency, generalized muscular hypertrophy, joint restriction, mixed hearing loss, and unusual facies including midface hypoplasia with prognathism, belephalophimosis, and short philtrum. MS also shows mild, but distinctive, skeletal alterations, such as thick calvaria, large pedicles, abbreviated long bones, and brachydactyly. MS was firstly reported in 1981, and SMAD4 was found to be the disease-causing gene in 2012. To date, more than 50 affected individuals have been known. It is intriguing that most cases were diagnosed in the school age, and the youngest affected individual was a 4-year-old girl. This fact raises a suspicion that MS is a late-onset disorder that is postnatally progressive.

We report here on an affected Japanese boy, whose clinical features become fully manifest over a period of mid-childhood. He was born at 38 weeks of gestation. Birth length and weight were normal. At age 3.5 years, he was referred to us due to short stature and mild mental retardation. Height was 88cm (-2.40 SD) and weight 13.4kg (-0.74 SD). He showed mild brachydactyly and thick skin. Cardiac assessment revealed mild pulmonary hypertension with complete right bundle branch block (CRBBB). Radiological examination demonstrated thick calvarium, brachymetacarpia, and fusion of C2-C3 vertebrae. No specific diagnosis was made. At age 8 years, he showed progressive joint contracture and skin thickness. Short philtrum, thin upper lip, prognathism and obesity were evident. Repeated skeletal survey revealed large pedicles, hypoplastic ilia, and deterioration of brachydactyly. The latter was ascertained based on the metacarpophalangeal (MCPP) pattern profile analysis (Z-score: -2.0 SD at age 3.5 years and -3.5SD at 8 years). These clinical and radiological findings led us to a Sanger analysis for SMAD4 , which showed a heterozygous mutation (c.1498A > G). In conclusion, our experience recapitulated what were previously reported in MS patients, and suggested the postnatal progressive nature of the disorder.

**Consent for publication:** The authors declare that written informed consent was obtained for publication.

## P1-8-20 Nutritional Rickets: A preliminary study in Sri Lanka

### Navoda Atapattu, Chamidri Randika Naotunna, Vasundara Vithanage, Ruwan Samararathna

#### Lady Ridgeway Hospital, Endocrinology and Diabetic Unit

**Introduction**: Nutritional rickets remains the single most common cause of rickets in many parts of the world. As Sri Lanka is a tropical country with adequate sun exposure throughout the year, Vitamin D deficiency is not considered as a diagnostic possibility.

**Objective**: Describe the aetiological classification of Rickets in Sri Lanka.

**Methodology**: Children who presented to the Endocrinology unit of Lady Ridgeway Hospital from September 2014 to May 2016 with radiological evidence of Rickets were included. Patients with chronic renal insufficiency and chronic liver disease were excluded. Duration of exposure to sun light and exclusive breast feeding, maternal vitamin D intake during lactation were obtained apart from socio-demographic data. Vitamin D level and bone profile was done.

**Results**: A total number of 61 children were included in our study. The mean age of presentation among the study group was 23 months while the age ranged from 3 months to 46 months. There was no statistically significant sex predisposition among the study group. 90% of children presented with bowing of legs.6.5% presented with hypocalcaemic seizures and 3.2% with delay in walking. 82% of children were exclusively breast fed for 6 months and were continued to breast feed up to the presentation and only 31% of mothers had vitamin D supplementation during lactation. 57.3% of children had no sun exposure at all. Among the cohort of 61 patients 10 patients( 16.4.4% ) had vitamin D levels below 30nmol/L and 33 patients ( 54% ) had levels between 30-50nmol/L. 2 (3.2%) patients were diagnosed with vitamin D dependant rickets.

**Discussion**: This preliminary study supports that continuing breast feeding beyond 6 months of age predispose children to nutritional rickets and the suboptimal maternal vitamin D supplementation during lactation may indicate maternal vitamin D deficiency on top of childhood vitamin D deficiency. Though we have adequate sun exposure still majority of our children did not get adequate sun exposure predisposing them to vitamin D deficiency. As majority of our patients had insufficient levels of vitamin D it points out the possibility of co existing calcium deficiency among this cohort.

**Conclusion**: This preliminary study further stresses the need of large scale community based studies in identifying the aetiology of rickets in Sri Lanka, Vitamin D supplementation during infancy, place of introducing fortification of food with vitamin D, importance of sun exposure.

## P2-1-1 Insulin pump initiation and education - current paediatric practice in Australia and New Zealand

### Yasmin H Abdul Aziz^1^, Hesham S Al-Sallami^1^, Esko Wiltshire^2^, Jinny Willis^3^, Jenny Rayns^4^, Joanna McClintock^5^, Natalie Medlicott^1^, Benjamin J Wheeler^4,6^

#### ^1^University of Otago, Dunedin School of Pharmacy; ^2^Department of Paediatrics and Child Health, University of Otago; ^3^Don Beaven Medical Research Centre; ^4^Dunedin Public Hospital, Pawediatric Endocrinology; ^5^Waikato District Health Board, Paediatric Diabetes; ^6^University of Otago, Women's and Children's Health

**Aims**: There are no published clinical guidelines on the training and education for paediatric and adolescent patients commencing insulin pump therapy. The study aims were to:

Describe current clinical practice regarding initiation of insulin pump therapy in children and adolescents with type 1 diabetes in hospitals in Australia and New Zealand.Identify differences in clinical practice both within and between these two countries.

**Methods**: Paediatric Diabetes Nurse Specialists from all New Zealand diabetes teams (n =16), and tertiary centres in Australia (n =9) were identified and approached by email and/or phone. For those consenting to participate, structured oral interviews were conducted. The questions covered basic hospital demographics, and various aspects of insulin pump initiation, including pump start planning, education and follow-up monitoring.

**Results**: Response rate was 16/16 for New Zealand, and 6/9 for Australia. Diabetes centres varied in size from 50 – 1154 patients; and frequency of pump use from 20% - 40%. Clinical practice differed both within and between each country, with differences seen relating to: the structure and duration of pump starts varying from 6 to 21 hours; access to funding; polarised views on use of advanced features, varying from immediate to delayed use; and the use of the continuous glucose monitoring (13/22). Location of pump starts also varied, with both in- (2/22) and outpatient (13/22) approaches seen, as well as a mixture of the two (7/22). The motivations and beliefs relating to these various pump start approaches also varied.

**Conclusion**: This is the first study to investigate current paediatric clinical practice in relation to insulin pump initiation. The lack of consistency seen between centres reflects a lack of consensus/guidance from the medical literature. Lessons may be learnt, and further rationalisation and improvement in education remains possible by combining and adopting strengths from different centres.

## P2-1-2 Increase of body mass index (BMI) from age 1.5 to 3 years augments the degree of insulin resistance corresponding to BMI at 12 years of age

### Go Ichikawa^1,2^, Junko Ichikawa^1^, Ayako Yoshida^1^, Satomi Koyama^1^, Toshimi Sairenchi^3^, Osamu Arisaka^1^

#### ^1^Department of Pediatrics, Dokkyo Medical University; ^2^Department of Pediatrics, Nasu Red Cross Hospital; ^3^Department of Public Health, Dokkyo Medical University

**Introduction**: Excess body fat (adiposity) is an important independent risk factor for development of insulin resistance in adults and children. Some obese children do not develop metabolic comorbidities of obesity, whereas some children are more metabolically sensitive to adiposity. The mechanism underlying this phenotype is not explained by adiposity levels, although metabolic sensitivity to adiposity may differ between ethnic groups. To interpret this mechanism, we hypothesized that an increase in body mass index (BMI) from about 1.5 to 3 years old, a period normally characterized by a decreased or stable BMI, may augment insulin resistance corresponding to BMI at adolescent age.

**Method**: All 192 children (101 boys and 91 girls) in a birth cohort (F town) in Tochigi prefecture in Japan were enrolled in the study. All of the children in the study were followed up in infant health checks at a health center during the preschool period, and data were stored at a regional health center. At 12 years of age, fasting blood sampling and measurement of blood pressure were performed at school. Fasting blood samples were collected for determination of plasma glucose and insulin. The participants were divided into groups by gender and based on an increase or no increase in BMI from age 1.5 to 3 years. In statistical analysis, regression coefficients between log-transformed BMI and log-transformed HOMA-IR were computed in regression analysis stratified by gender and change of BMI from 1.5 to 3 years.

**Result**: The augmented degree of log-transformed HOMA-IR per log-transformed BMI was significantly higher in girls who had a BMI increase from 1.5 to 3 years compared to those who did not have this increase. A similar tendency was observed in boys, but without a significant difference.

**Discussion**: This suggests that children who show an increase of BMI from 1.5 to 3 years, a period normally characterized by a decreased or stable BMI, are more prone to developing insulin resistance at 12 years of age. From a practical standpoint, identification of individuals with a higher cardiometabolic health risks may allow more aggressive intervention for these persons than for their lower-risk peers.

## P2-1-3 Nucleotide substitutions in CD101, the human homolog of diabetes mellitus susceptibility gene in non-obese diabetic mouse, in patients with type 1 diabetes mellitus

### Misako Okuno^1,2^, Yoshihito Kasahara^3^, Noriyuki Takubo^4^, Michiko Okajima^3^, Shigeru Suga^5^, Junichi Suzuki^2^, Tadayuki Ayabe^1^, Tatsuhiko Urakami^2^, Tomoyuki Kawamura^6^, Nobuyuki Kikuchi^7^, Ichiro Yokota^8^, Toru Kikuchi^9^, Shin Amemiya^9^, Tsutomu Ogata^1,10^, Maki Fukami^1,2^

#### ^1^Department of Molecular Endocrinology, National Research Institute for Child Health and Development; ^2^Department of Pediatrics and Child Health, Nihon University School of Medicine; ^3^Department of Pediatrics, Kanazawa University; ^4^Department of Pediatrics, Juntendo University; ^5^Department of Pediatrics, National Hospital Organization Mie Hospital; ^6^Department of Pediatrics, Osaka City University; ^7^Department of Pediatrics, Yokohama City Minato Red Cross Hospital; ^8^Department of Pediatrics, Division of Pediatric Endocrinology and Metabolism, Shikoku Medical Center for Children and Adults; ^9^Department of Pediatrics, Saitama Medical University; ^10^Department of Pediatrics, Hamamatsu University School of Medicine; ^11^Department of Pediatrics, Tokyo Women's Medical University Medical Center East

**Objectives**: Genome wide association studies have identified more than 50 susceptibility genes for type 1 diabetes mellitus. However, low frequency risk variants could remain unrecognized. The present study aimed to identify novel type 1 diabetes mellitus susceptibility genes by newly established methods.

**Methods**: We performed whole-exome sequencing and genome-wide copy-number analysis for a Japanese family consisting of two patients with type 1 diabetes mellitus and three unaffected relatives. Further mutation screening was carried out for 127 individuals with sporadic type 1 diabetes mellitus. The functional consequences of identified substitutions were evaluated by in silico analyses and fluorescence- activated cell sorting of blood samples.

**Results**: Familial molecular analysis revealed co-segregation of the p.V863L substitution in CD101, the human homolog of an autoimmune diabetes gene in the non-obese diabetic mouse, with type 1 diabetes mellitus. Mutation screening of CD101 in 127 sporadic cases detected the p.V678L and p.T944R substitutions in two patients. The p.V863L, p.V678L, and p.T944R substitutions were absent or extremely rare in the general population and were assessed as “probably/possibly damaging” by in silico analyses. CD101 expression on monocytes, granulocytes, and myeloid dendritic cells of mutation-positive patients was weaker than that of control individuals.

**Conclusions**: These results raise the possibility that CD101 is a susceptibility gene for type 1 diabetes mellitus.

## P2-1-4 Molecular mechanisms of insulin resistance in two cases of primary insulin receptor defect- associated diseases

### Atsumi Tsuji^1^, Kei Takasawa^1^, Risa Nomura^1^, Yuichi Miyakawa^1^, Chikahiko Numakura^2^, Atsushi Hijikata^3^, Tsuyoshi Shirai^3^, Yoshihiro Ogawa^4^, Kenichi Kashimada^1^, Tomohiro Morio^1^

#### ^1^Department of Pediatrics and Developmental Biology, Tokyo Medical and Dental University; ^2^Department of Pediatrics, Yamagata University School of Medicine; ^3^Faculty of Bioscience, Nagahama Institute of Bio-Science and Technology; ^4^Department of Molecular Endocrinology and Metabolism, Tokyo Medical and Dental University

**Introduction**: Defects of the insulin receptor gene (INSR ) cause clinically wide spectrums of congenital insulin resistance. Monoallelic defects develop milder type, insulin-resistant diabetes mellitus with acanthusis nigricans (IRAN, type A) with autosomal dominant inheritance. On the other hand, leprechaunism (Donohue syndrome), which is caused by biallelic defects of INSR with autosomal recessive inheritance, is an extremely rare and the severest condition with lethal phenotype during infantile period. We detected two missense mutations in our cases of primary insulin-receptor (INSR) defects, leprechaunism and IRAN, type A, and reduced mRNA expression in the former case.

**Methods**: [Case1] 11 year-old male was referred to our hospital because of acanthosis nigricans in the axilla and insulin resistance (HOMA- IR:16.9). [Case2] 8 day-old female born with a birth weight of 1234g at 36 weeks of gestation was referred to us for an elfin-like face, acanthosis nigricans, and severe insulin resistance (plasma glucose level:>11.0 mmol/L and IRI:7430-46400 pmol/L). Direct sequencing of INSR gene revealed a missense mutation for each case. We performed in vitro analysis including INSR phosphorylation assay, insulin binding assay and qRT-PCR for mRNA of INSR to confirm the two mutations are responsible for the phenotype.

**Results**: The heterozygote mutations c.3436G>A (p.Gly1146Arg) and c.294C>A (p.Ser98Arg) were identified in Case1 and Case2, respectively. Gly1146Arg is previously reported in a diabetic case without precise functional analyses, and Ser98Arg is a novel mutation. Gly1146 is located on tyrosine kinase domain and Gly1146Arg resulted in impairment of INSR phosphorylation, while Ser98 is located on the ligand- binding domain of INSR and assay for insulin binding revealed disruption of INSR by Ser98Arg. Three dimensional model analyses supported these results. In addition to the missense mutation Ser98Arg, mRNA expression of INSR in her lymphocytes was reduced at qRT-PCR assay in the leprechaunism case.

**Discussion**: We concluded two missense mutations are responsible for primary insulin resistance. In case2, reduced expression level of mRNA, together with defect of INSR by Ser98Arg, resulted in severest phenotype. Further, our present study suggested that reduced expression of INSR should be considered as a possible cause for insulin resistance.

**Consent for publication:** The authors declare that written informed consent was obtained for publication.

**Fig. 1 (abstract P2-1-4).**
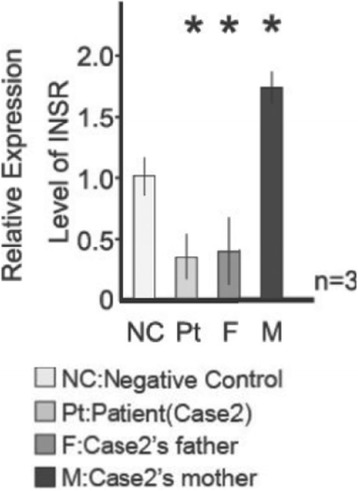
See text for description

## P2-1-5 Evaluation of Hip/HeightP Ratio as an Index for Adiposity and Metabolic Complications in Obese Children: Comparison with Waist-related Indices

### Kazushige Dobashi^1^, Kenichiro Takahashi^1^, Daisuke Tanaka^1,2^, Junya Toyoda^1^, Takuya Ishikawa^1^, Keiko Nagahara^1^, Yuya Nakano^1^, Yoshifusa Abe^1^, Kazuo Itabashi^1^

#### ^1^Department of Pediatrics, Showa University School of Medicine; ^2^Showa University Graduate School of Health sciences

**Aim:** To prevent obesity in middle age, early precautions and interventions are required during childhood. Therefore, it is important to find an indicator that can accurately predict the degree of both adiposity and metabolic complications in children. In 2011, a new anthropometric indicator, body adiposity index (BAI; hip/height1.5 – 18), was proposed as a good marker for adiposity in adults. The aim of this study was to investigate whether BAI and other hip/heightP ratios are useful in obese children.

**Method**: Ninety obese Japanese children, 55 boys and 35 girls, who visited our University Clinic, were enrolled. The age was 9.92 ± 2.6 (mean ± SD) years. In Japan, a child was considered to be obese when the percentage overweight (POW) exceeded 20%. POW = 20% means that one’s body weight is 120% of standard body weight in 2000. The mean POW and BMI-for-age percentile value were 51.6 ± 18.8 (%) and 97.8 ± 2.5 (percentile), respectively. We set the power value of the hip/heightP 0, 0.5, 1, 1.5, and 2 and studied the association with overweight indices, biochemical data, and fat area measured by computed tomography. Waist circumference, waist/height ratio, and waist/hip ratio were also evaluated.

**Results**: Hip/height was more closely correlated with POW, BMI-percentile, and percentage body fat than hip/height1.5 (BAI). The correlation coefficient of hip/height with POW (r = 0.855) was the highest among the studied hip/heightP indices. The waist/height was also highly correlated with POW (r = 0.879). The approximate line to predict POW was 411 × hip/height – 207 and 308 × waist/height – 131, respectively. If one’s hip/height is >0.63 or waist/height is >0.59, he or she is considered to be severely obesity, POW > 50%. Hip and hip/height0.5 were more closely correlated with visceral fat area than hip/height and hip/height1.5. Hip and hip/height0.5 were significantly correlated with serum insulin level. Only hip was also significantly associated with dyslipidemia. All hip/heightP indices were not significantly correlated with alanine aminotransferase (ALT). Waist was significantly correlated with serum lipids, ALT, and insulin.

**Conclusion**: Hip/height is a better marker for overweight (adiposity) in obese children than BAI. However, hip/height and BAI are not useful to predict metabolic complications. Waist/height is also a marker for overweight, but not for visceral adiposity. Waist circumference itself is considered to be the best index for obese children overall at this time because waist significantly reflects the metabolic complications.

## P2-1-6 Novel mutation of INSR gene identified in a Korean family with Rabson-Mendenhall syndrome

### Yoo-Mi Kim^1^, Chong Kun Cheon^1^, Han-Wook Yoo^2^, Su Young Kim^1^

#### ^1^Pediatric endocrinology/Department of pediatrics, Pusan National University Children's Hospital; ^2^Department of Pediatrics, Asan Medical Center Children's Hospital

**Objective**: Donohue syndrome or leprechaunism (OMIM: 246200) and Rabson-Mendenhall syndrome (OMIM: 262190) are two rare autosomal recessive conditions caused by mutations in the INSR gene. We reported an infant with Rabson-Mendenhall syndrome and premature thelarche who has a novel mutation of INSR gene.

**Case**: A 7-month-old girl visited our hospital due to fasting hypoglycemia. Not only fasting hypoglycaemia, but also postprandial hyperglycemia with severe hyperinsulinemia were found. This subject showed hirsutism, anteverted nostrils, dry and loose skin, and brachydactyly. Typical acanthosis nigricans was observed on the neck, axillae, and inguinal area. The girl was born at 39 weeks of gestational age with 2.17 kg of birth weight. The height and weight at visit were 3rd percentile, respectively and the breast were in Tanner stage II. Pelvic unltrasound showed multiple cysts in the bilateral ovaries and estradiol, and DHEA-S were also elevated. The serum glucose, insulin, and C-peptide during fasting were 48 mg/dL, >300 μIU/mL, and 28.6 ng/mL, respectively. The HbA1c was 6.8%. Direct sequencing of INSR revealed compound heterozygous mutations of c.356C>T (p.Ala119Val) in exon 2 and c.2978C>A (p.Ala993Asp) in exon 16. A novel nonsynonymous variant, p.Ala993Asp, within a highly conserved regionwas considered as a pathogenic mutation by in silico tools (SIFT and polyphen-2). Each mutation was inherited from the parents, respectively. Metformin was administrated and glucose was controlled within 70 to 250 mg/dL.

**Conclusions**: This study describes the clinical course of an infant patient with Rabson-Mendenhall syndrome and the molecular characteristics of compound heterozygous INSR mutations including a novel missense mutation.

**Consent for publication:** The authors declare that written informed consent was obtained for publication.

## P2-1-7 Neonatal diabetes: some unique presentations

### Pragya Mangla, Manoranjan Tripathy, Siddhnath Sudhanshu, Kriti Joshi, Vijayalakshmi Bhatia

#### Department of Endocrinology, Sanjay Gandhi Postgraduate Institute of Medical Science

Genetic diagnosis is crucial in neonatal diabetes mellitus (NDM) for therapy and prognostication. We present unique genetic and clinical features in 4 patients with NDM.

Patient 1 has Wolcott- Rallison syndrome (WRS) with two novel missense compound heterozygous mutations in exons 12 and 13 of the EIF2AK3 gene (c.1975 G>A/c.2809 C>T). Presentation was at 2 months of age, with diabetic ketoacidosis, anemia and liver failure. At last follow up visit at 3 years of age, she had normal development, no further episode of hepatic failure, renal failure and anemia. Remarkably, she does not have any skeletal dysplasia till now.

Patient 2 has a homozygous missense mutation in exon 5 of the ABCC8 gene which is known to cause transient NDM (TNDM) in heterozygous but not in homozygous form. The child presented at the age of 36 days with diabetic ketoacidosis. He was initially started on insulin but after getting the genetic report, was successfully switched over to glibenclamide. By 14 months of age, the drug was tapered and stopped. At 36 months of age, he is still euglycemic. Both parents have normal glucose tolerance so far.

Patient 3, diagnosed at 2.5 years of age, has a homozygous glucokinase (GCK) missense mutation (p.Gly44Ala). She was symptomatic since 8 months of age, had intrauterine growth retardation, motor delay, seizures and hemiparesis. She also has some dysmorphic features like depressed nasal bridge, bilateral epicanthic fold, short 4th and 5th metacarpals. These neurological and dysmorphic features are unusual in GCK PNDM. Her younger male sibling (homozygous for the same mutation) is a known case of NDM since 22 days of life. Both the parents have been newly diagnosed with diabetes and are heterozygous for the mutation. They belong to an endogamous community, but have a non- consanguineous marriage. Mother has a 2 generation family history of diabetes with onset in young adulthood.

**Conclusion**: Genetic diagnosis plays an important role in the management algorithm of neonatal diabetes. WRS has highly variable phenotype. Some homozygous activating ABCCD8 mutations may be associated with TNDM. GCK mutation can have varied presentation within a family. Acknowledgement: We are grateful to Royal Devon and Exeter NHS Foundation Trust and Prof. Hattersley for carrying out the genetic studies for all the babies.

**Consent for publication:** The authors declare that written informed consent was obtained for publication.

## P2-1-8 Increase in Peripheral Intermediate Monocytes is Associated with the Development of Recent-Onset Type 1 Diabetes Mellitus in Children

### Xiaoya Ren^1,2^, Wenjun Mou^2^, Chang Su^1^, Xi Chen^2^, Hui Zhang^2^, Bingyan Cao^1^, Xiaoqiao Li^1^, Xunyao Wu^2^, Di Wu^1^, Jingang Gui^2^, Chunxiu Gong^1^

#### ^1^Department of Endocrinology, Genetics and Metabolism, Beijing Children's Hospital, Capital Medical University; ^2^Beijing Children's Hospital, Capital Medical University, Laboratory of Immunology, Beijing Pediatric Research Institute

**Aims**: Type 1 diabetes mellitus (T1DM) is an autoimmune disease characterized by destruction of the beta-cells of the pancreas. Monocytes play important roles in antigen presentation and cytokine production for the proper immune response in the development and progression of T1DM. This study aimed to analyze different monocyte subsets in the peripheral blood of recent-onset T1DM children in respect to the proportion change and likely relevance to the residual islet beta cells function.

**Methods**: To determine if monocytes were abnormally changed in T1DM, 4-color flow cytometry analysis was performed in a cross- sectional study of 31 T1DM children and 22 healthy controls. In our hospital HbA1c was tested by the method of High Performance Liquid Chromatography (HPLC), and insulin and C-peptide was measured by a Roche cobase 601 analyser. All statistical analyses were performed using SPSS.

**Results**: Our demonstration that the percentage and absolute number of intermediate monocytes in peripheral blood were expanded in patients with T1DM inumber Fpatients 3.73 ± 2.60 × 104, control 2.43 ± 1.14 × 104 Ct =2.07 CP<0.05; percentage: patients 13.47 ± 1.50, control 10.60 ± 1.48 Ct =6.55, P <0.05 ). The increase of the intermediate monocytes in patients was positively associated with HbA1c (r=0.45, P<0.05), but negatively associated with serum insulin (r=-0.62, P<0.05) and C-peptide (r=-0.57, P<0.05). In addition, the frequency of CD45RO+CD3+ memory T cells were found increase in patients, which exhibits a significant positive correlation with increased intermediate monocytes (r=0.44, P<0.05).

**Conclusions**: As the intermediate monocytes were shown to have proinflammatory activity, they are likely to implicate in the impaired function of b cells, which have deleterious consequences for the development of T1DM. Further characterization of the function of intermediate monocytes could pave the road for understanding the relationship between these pro-inflammatory cells and the destruction of b cells in T1DM children.

## P2-1-9 Serum dipeptidyl peptidase 4 activity in children with type 1 diabetes mellitus indicates insulin insensitivity

### Atsushi Iwabuchi^1^, Tomohiro Kamoda^1^, Kana Tamai^1^, Hiroyuki Shinohara^1^, Isho Izumi^2^, Takeki Hirano^3^, Ryo Sumazaki^1^

#### ^1^Department of Child Health, University of Tsukuba; ^2^Department of Pediatrics, Ibaraki Children's Hospital; ^3^Hirano Children's Clinic

**Background**: Dipeptidyl peptidase 4(DPP4) is an enzyme well known with its action in inactivating GLP-1 and its inhibitor has been already used for adult type 2 diabetes. Recent reports elucidated increased expression of DPP4 in visceral adipocytes of obese adults and hepatocytes of adults with non-alcoholic fatty liver disease. These findings support the hypothesis that DPP4 plays a role in pro-inflammatory pathways in adipose tissues to enhance local insulin resistance. The relationship between DPP4 and insulin resistance is well studied among adults but not in children.

**Aim**: The aim of this study was to elucidate the relationship between DPP4 and insulin resistance in type 1 diabetes mellitus (T1DM) by measuring serum DPP4 activity and evaluating metabolic markers related to insulin resistance.

**Subjects**: Forty-three Japanese children with T1DM (17 boys and 26 girls, mean age 9.3 yr) and 26 age- and gender-matched control children (14 boys and 12 girls, mean age 9.7 yr) were enrolled. Methods: Blood samples were obtained from each child in the fasting state. Serum DPP4 activities were measured by fluorometric assay. Blood glucose, HbA1c, glutamic acid decarboxylase antibodies (GADA), C-peptides and adiponectin levels were measured in the T1DM children. In the control children, serum DPP4 activities, blood glucose and adiponectin levels were measured.

**Results**: Serum DPP4 activities were significantly higher in the T1DM children than in the controls (3.57 ± 0.99 vs.2.67 ± 0.77U/mL, p<0.001). In the T1DM children, DPP4 activities were not correlated with HbA1c, blood glucose, or duration of diabetes. A significant negative correlation was found between DPP4 activities and serum adiponectin levels in the T1DM group (r=-0.35, p<0.05).

**Conclusions**: Serum DPP4 activity was increased in T1DM children, whereas it was not associated with glycemic control. This study indicates the possibility that DPP4 in T1DM children plays a significant role in enhancing insulin resistance when it was measured by decreased adiponectin level as surrogate marker. Further mechanisms are to be elucidated specially when harboring clinical application of DPP4 inhibitor to T1DM children.

**Fig. 1 (abstract P2-1-9).**
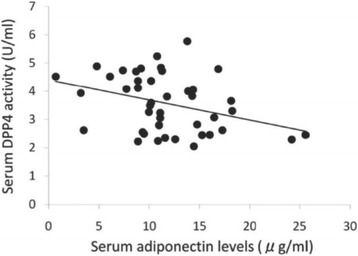
See text for description

## P2-1-10 The Effect of Sfrp5, Wnt5a, Adiponectin, and Chemerin on Blood Pressure Regulation in Obese Children

### Yan C Yin

#### Pediatric, The Second Affiliated Hospital of Xi'an Jiaotong University

The aim was to evaluate the associations of Sfrp5 and Wnt5a with blood pressure (BP), and to examine whether BP can be influenced by changes in adipocytokines, such as Sfrp5, adiponectin, chemerin, and hsCRP, in obese children after lifestyle intervention. A cross-sectional study was conducted in 263 obese children. In addition, a 6-month lifestyle intervention was performed in a subgroup of 89 obese children with hypertension. Anthropometric parameters, clinical data, adiponectin, chemerin, Sfrp5, and Wnt5a were measured at baseline and after lifestyle intervention. Sfrp5 and adiponectin levels were significantly lower in obese children with hypertension, but Wnt5a, hsCRP, and chemerin levels were elevated in obese children with hypertension. In multivariable linear regression analysis, Sfrp5, Wnt5a, adiponectin, chemerin, and hsCRP were associated with both standard deviation score-systolic blood pressure (SDS-SBP) and -diastolic blood pressure (SDS-DBP). Lifestyle intervention resulted in a significant improvement in BP and weight loss. These were accompanied by significant decreases in hsCRP and chemerin, and significant increases in Sfrp5 and adiponectin, whereas Wnt5a was not changed. Furthermore, the changes in Sfrp5, adiponectin, chemerin, and hsCRP act as partial mediators of the relationship between weight loss and BP reduction. Although Sfrp5 and Wnt5a levels correlated with BP at baseline, after lifestyle intervention, Sfrp5 is more sensitive to reduction in BMI and BP compared to Wnt5a, and the relationship between weight loss and BP reduction were partially mediated by changes in Sfrp5. So we speculate if Sfrp5 and Wnt5a each play a role in regulating BP, it must be different roles.

## P2-1-11 Duration of effect between new long-acting insulin glargine U300 and degludec in type 1 diabetes patients; a crossover clinical trial

### Yuko Hotta^1^, Kayako Hashimura^1^, Yoneo Kashihara^2^, Tomomi Hashimoto^2^, Takashi Higashide^2^, Shigeo Aono^3^, Tomoyuki Kawamura^1^

#### ^1^Department of Pediatrics, Osaka City University Graduate School of Medicine; ^2^Pediatrics, Hug Hug Kids Clinic; ^3^Pediatrics, Teradacho Kodomo Clinic

**Background**: It is said that the effect of new long acting insulin glargine U300 and degludec continue over 24 hours from injection. It is unclear that which is more useful and effective.

**Subject and method:** We conducted a crossover study to compare the effect of new long-acting insulin in patients with type 1 diabetes. 9 young type 1 diabetes patients participated in this study with informed consent. All patients were treated by multiple daily injection therapy, and they were randomly assigned to change insulin degludec or glargine 300. After 2 months use, they switched to another long-acting insulin. On the last day of each phase, they skip their long acting insulin injection until next morning. We compared about blood glucose (BG) differences from bedtime BG to fasting BG between degludec and glargine U300.

**Results**: Mean age and diabetes duration was 15.9 ± 6.0 years old, and 8.5 ± 7.3 years respectively. Before registration of this study, their mean HbA1c was 7.8 ± 1.0%, and mean total daily dose was 63.5 ± 24.8 units (1.01U/kg/day). Mean long acting insulin dose was 24.4 ± 11.1 Unit/day. Mean HbA1c in degludec phase (7.6 ± 0.8%; 1M and 7.5 ± 0.6; 2M) was lower than glargine U 300 phase (7.9 ± 1.0%; 1M and 8.0 ± 0.9%; 2M). However, there was not significant difference between two groups (p = 0.604). Insulin requirement of glargine U300 phase (28.8 ± 18.9 U; 1M and 27.7 ± 20.2 U; 2M) was more than that of degludec phase (24.1 ± 11.3 U; 1M and 24.4 ± 12.9 U; 2M). Also there was not significant difference between two groups (p = 0.378). One patient required approximately 1.5 times glargine U300 dose (75U) than degludec (52U). After skipping injection, the BG change was from 174.1 ± 61.3 mg/dl to 233.6 ± 103.6 mg/dl in glargine U300 phase. In degludec phase, the BG change was from 169.3 ± 81.5 mg/dl to 269.1 ± 71.3 mg/dl. There was not significant difference between two groups (p = 0.386).

**Conclusion**: There was not a significant difference between insulin glargine U300 and insulin degludec about approximately 1.5 day effect. Young patients with type 1 diabetes tend to require more glargine U300 dose than insulin degludec.

## P2-1-12 Association of tumor necrosis factor (TNF) - α gene polymorphisms with overweight and nonalcoholic fatty liver disease in Indian overweight adolescents

### Anil Kumar, Ahmad Nayeem, Oshima Jain, Vandana Jain

#### Division of Endocrinology/Department of Pediatrics, All India Institute of Medical Sciences

**Background/Objectives:** Non alcoholic fatty liver disease (NAFLD) is a complex metabolic condition leading to chronic liver disease. Tumor necrosis factor (TNF)- α is an inflammatory mediator that may have a role in pathogenesis of NAFLD. The aim of the study was to investigate the association of polymorphisms in the promoter region of TNF- α gene with overweight and NAFLD in Indian adolescents.

**Methods**: Overweight adolescents were enrolled from Pediatric OPD, AIIMS, Delhi. NAFLD was diagnosed by ultrasonography. Healthy lean adults aged 40-60 years were recruited as controls after excluding diabetes and NAFLD. Polymerase chain reaction and restriction fragment length polymorphism (PCR-RFLP) were undertaken to detect SNPs: -238 (rs 361525), -863 (rs 1800630) and -1031 (rs 1799964) in promoter region of TNF- α gene. Genotype frequencies were compared with Asian HapMap- HCB population. The genotype distribution for all 3SNPs was in Hardy-Weinberg equilibrium.

**Results**: 208 overweight adolescents (130 (62.5%) with NAFLD) and 88 lean adult controls without NAFLD were enrolled. The comparison of frequency of wild type, heterozygous and homozygous mutants for TNF- α genes is presented in Table 1. We found that the overweight subjects with NAFLD as well as those without NAFLD had significantly higher frequency of homo mutant and hetero mutant alleles of -863 and -1031 SNPs of TNF- α in comparison to controls. Genotype frequency distribution of -238 SNP of TNF- α was similar between subjects and controls.

**Conclusion**: Our results indicate that -863 and -1031 promoter gene polymorphisms of TNF- α may be associated with increased risk of overweight in adolescents. However, they do not confer additional risk of NAFLD in overweight adolescents.

**Table 1 (abstract P2-1-12).**
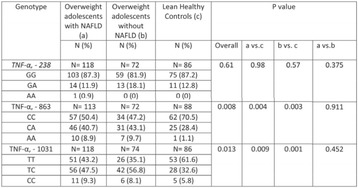
Comparison of frequency of wild type, heterozygous and homosygous mutants for the *TNF- α* SNPs between overweight subjects (with and without NAFLD) and lean healthy adult controls

## P2-1-13 The analyses of the influence of iron overload in glucose metabolism in thalassemia major patients and the related mechanism

### Wenqin Lao^1^, Liyang Liang^2^, Hui Ou^2^, Zhe Meng^2^, Lina Zhang^2^

#### ^1^Department of Pediatrics, The Second Affiliated Hospital of Guangzhou Medical University; ^2^Department of Pediatrics, Sun Yat-sen Memorial Hospital

**Objective**: This study aimed at determining the characteristics of the glucose homeostasis and the status of the β -cell function of the patients with β -thalassemia major ( β -TM) in our hospital and studying the potential factors responsible for secondary abnormal glucose of TM patients, in order to provide the clinical evidences for early diagnosis and treatment of abnormal glucose induced by iron overload.

**Method**: A total of 57 (36 males) transfusion-dependent β -TM patients were in the “TM group ”; 25 age-and gender- matched healthy teenagers were recruited as the “control group”. The height, weight, fast blood glucose(FBG) and insulin level, serum ferritin (SF), superoxide dismutase (SOD ) and ultrasensitive C-reactive protein(hs-CRP) were evaluated in all subjects. Insulin resistance index (IRI), insulin sensitivity index and β -cell function index (BFI) were also estimated.There were 36 patients estimating cardiac T2* and liver T2*.

**Result**: 1 The TM group had significantly higher levels of FBG, FINS and HOMA-IRI than the control group. But HOMA-ISI and HOMA- β FI were lower with no statistic sense in HOMA- β FI. None of the control group had IFG or DM. In TM group, the prevalence was 24.56% and7.02% respectively. There were 43.33% of patients with cardiac T2* < 20 ms and 96.27% of patients with liver T2* < 6.3ms. 2 The incidences of abnormal glucose among different ages, the levels of SF and the severity of cardiac iron overload were different. And the incidence increased with them.3 The levels of SOD and hs-CRP were higher in the TM group than in the control group.4 The result of logistic regression analysis indicated that the cardiac T2* was a significant predictor for the incidence of abnormal glucose in TM patients.

**Conclusion**: 1 In our center the prevalence of IFG and DM was 24.56% and7.02% respectively. 2 Iron overload was the main cause of abnormal glucose even the secondary diabetes mellitus in β -TM patients.Factors like age over 5, the level of SF over 2000ng/ml and moderate degree of cardiac iron increased the incidences of abnormal glucose in β -TM patients. The cardiac T2* was an independent risk for the incidence of abnormal glucose in TM patients. 3 The pathophysiological procedure of secondary of DM in TM was peripheral insulin resistance happened first; then insulin secretion decline as it exacerbated; finally the function of β cell became failure, which was similar to the type 2 diabetes.

## P2-1-14 The efficacy of Vitamin D supplementation on the improvement of serum 25(OH)D3 and HbA1c levels in pediatric patients with type 1 diabetes mellitus

### James Q Chin^1,2^, Sioksoan C Cua^2^, Lorna R Abad^2^

#### ^1^Section of Pediatric Endocrinology and Metabolism, Vicente Sotto Memorial Medical Center; ^2^Section of Pediatric Endocrinology and Metabolism, University of the Philippines- Manila, Philippine General Hospital

**Background**: Vitamin D has hormonal activities in humans. Its most widely known function is its role in bone metabolism and calcium homeostasis. Studies have outlined clinical evidence on the non-classical role of vitamin D in many autoimmune diseases, type 1 diabetes mellitus (T1DM), included. Studies have suggested a link between vitamin D deficiency in early life and the development of T1DM later in life. Locally, a significant proportion of T1DM patients have Vitamin D inadequacy.

**Objective**: This study aims to study the effect of Vitamin D supplementation on the Vitamin D status and HbA1c levels of T1DM pediatric patients with vitamin D inadequacy.

**Subjects and Methods:** A prospective cohort, interventional study of 34 subjects with T1DM ( 21 females, 13 males ) with mean age of 14.5 ( SD 5.23, 11.07-18.49 ), and mean duration of 5.74 years. All subjects had (1) a diagnosis of diabetes following the International Society of Pediatric and Adolescent Diabetes (ISPAD) diagnostic criteria, (2) had baseline measurements of 25(OH)D, HbA1c, ALT, Creatinine, and Sun Exposure Score, (3) received Vitamin D supplementation, thereafter, in addition to routine T1DM management, and (4) had post supplementation measurements of 25(OH)D, HbA1c and Sun Exposure Score.

**Results**: At baseline, Vitamin D deficiency was noted in 100% of the subjects, with an average 25(OH)D level of 6.93 ng/ml ( SD 6.3; 1.4-15.1). Further, 64.71% of the subjects had poor glycemic control, with an average HbA1c of 9.42% ( SD 5.67, 5.3 - 17 ). Post-supplementation, Vitamin D levels improved, with 11.76% of subjects having sufficient levels and 58.82% having insufficient levels. The mean 25(OH)D post- supplementation was 23.21 ng/ ml ( SD 9.77, 13.6 - 37.7 ). Post-supplementation, 61.76% of subjects still had poor glycemic control, with an average HbA1c level of 9.82% ( SD 4.2, 5.4 - 14.7 ). There was a statistically significant increase in 25(OH)D level post-supplementation ( p < 0.01 ). However, there was no statistically significant change in HbA1c levels ( p = 0.32 ).

**Conclusion**: Vitamin D deficiency is prevalent in patients with T1DM. Vitamin D supplementation in these patients was associated with a statistically significant increase in 25(OH)D levels. Despite this, there was no statistically significant change noted in the HbA1c levels of the subjects. There is a need to look into other factors contributing to the glycemic control in these subjects.

## P2-1-15 Risk Factors of Recurrent Diabetic Ketoacidosis among Type 1 Diabetes Mellitus Children in Dr. Hasan Sadikin General Hospital

### Novina Na Novina^1,2^, Hadiati HR Rabbani^4^, Deni DKS Sunjaya^3^

#### ^1^Division of Endocrinology / Department of Child Health, Dr. Hasan Sadikin General Hospital; ^2^Division of Endocrinology / Department of Child Health, Faculty of Medicine Universitas Padjadjaran; ^3^Department of Public Health, Faculty of Medicine Universitas Padjadjaran; ^4^Faculty of Medicine Universitas Padjadjaran

**Background**: Type 1 Diabetes Mellitus (T1DM) prevalence in Indonesian children is increasing. Ketoacidosis is the leading cause of mortality in children with T1DM. Ketoacidosis is life-threatening acute complication that could be preventable. This study aimed to identify risk factors that are responsible for recurrence to reduce mortality from that and increase quality of life of children with T1DM.

**Method**: A sequential mixed method study was conducted among nineteen T1DM children who had ketoacidosis between 2010 and 2014 at Department of Child Health in Dr. Hasan Sadikin General Hospital, Bandung. We reviewed the medical records to identify the risk factors followed by interviewed parents after got the consent for qualitative analysis through phenomenology approach.

**Results**: Nine patient from 19 children included had recurrent ketoacidosis. Twelve of patients are female which seven of them had recurrence. All patients showed poor glycemic control. Type of insulin therapy was not implicated to recurrence state. Insulin therapy compliance contributed to ketoacidosis recurrence. Control compliance was involved to insulin therapy compliance. Patients and parents knowledge and understanding toward T1DM, and geographical and economical access to medical care influences the control compliance.

**Conclusion**: Main risk factor of recurrent ketoacidosis is insulin therapy compliance. In the context of new scheme health insurance implementation, insulin therapy compliance was related to hospital control compliance. Counselling was needed to enhance knowledge and understanding of patients and parents toward T1DM. Government and society role were expected to overcome the opportunity cost.

**Keywords**: Diabetic ketoacidosis, recurrence, risk factors, type 1 diabetes mellitus

## P2-1-16 Clinical and molecular characterization of children with neonatal diabetes mellitus at a tertiary care centre in north India

### Vandana Jain^1^, Amit Satapathy^1^, Jaivinder Yadav^1^, Sian Ellard^2^, Elisa De Franco^2^, Mohan Viswanathan^3^, Radha Venkatesan^3^

#### ^1^Division of Endocrinology / Department of Pediatrics, All India Institute of Medical Sciences; ^2^University of Exeter Medical School, Exeter, Institute of Biomedical and Clinical Science; ^3^Madras Diabetes Research Foundation

**Objective**: To study the genetic mutations and clinical profile in children with neonatal diabetes mellitus (NDM)

**Methods**: Genetic evaluation and clinical follow- up of children with a diagnosis of NDM at Department of Pediatrics, AIIMS, Delhi in last 7 years

**Results**: Out of 12 infants with NDM, 8 had permanent type. Pathogenic genetic mutations were identified in 8 (66.7%) children. Three infants with mutations in KCNJ11 gene and 1 with mutations in ABCC8 gene were switched to oral sulfonylurea. Mutations in INS gene were identified in 3 infants and ZFP57 mutation in differentially methylated region of chromosome 6q24 in 1. The median age at initial presentation was 8 weeks, mean birth weight was 2.4 ± 0.5 kg and presentation with diabetic ketoacidosis was seen in 6 infants (50%). The current median age is 2 yrs (range 5.5 months to 7 yrs) and mean HbA1c is 7.0 ± 0.9% (Table 1).

**Conclusion**: NDM is a heterogeneous disorder. Identification of the genetic cause guides clinical management. With concerted team effort, optimal glycemic control, growth and development are achievable in the children on sulfonylureas as well as those requiring insulin.

**Table 1 (abstract P2-1-16).**
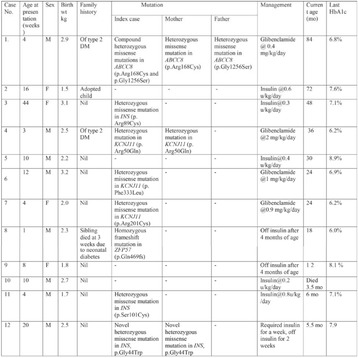
Summary of clinical, demographic and genetic mutation profile of the cases

## P2-1-17 Acute Effects of Blood Transfusion on Insulin Sensitivity and β-Cell Function in Transfusion Dependent β-Thalassemia/ HbE Disease

### Somboon Wankanit, Pat Mahachoklertwattana, Ampaiwan Chuansumrit, Preamrudee Poomthavorn

#### Division of Endocrinology / Department of Pediatrics, Ramathibodi hospital, Mahidol university

**Background**: Pancreatic iron deposition in regularly blood-transfused thalassemic patients causes β -cell dysfunction and diabetes. Insulin resistance is the other mechanism contributed to the development of diabetes. However, the acute effects of blood transfusion in pre-existing iron-overload thalassemic patients on insulin sensitivity and β -cell function are unknown.

**Methods**: Fifty children, aged 5-20 years with β -thalassemia/HbE disease were enrolled. Oral glucose tolerance tests (OGTT) were performed twice, before and at 1 week after blood transfusion. Insulin sensitivity and β -cell function indices [homeostatic model assessment (HOMA) of insulin resistance (HOMA-IR), HOMA of β -cell function (HOMA- β ), insulinogenic index (IGI), whole body insulin sensitivity index (WBISI) and disposition index (DI)] were calculated from glucose and insulin levels obtained during the OGTT. Hemoglobin (Hb) and serum ferritin were measured from the baseline blood samples obtained at each OGTT.

**Results**: Median Hb and serum ferritin were significantly increased at 1 week following transfusion from 8.6 to 10.1 g/dL (p <0.001) and 1764 to 2160 ng/mL (p < 0.001), respectively. β -Cell function indices were significantly increased following the transfusion [median IGI: 0.48 vs. 0.61 (p=0.03) and median HOMA- β: 74.3 vs. 82.0 (p=0.045)]. However, insulin resistance tended to increase following the transfusion as demonstrated by decreasing median WBISI (11.9 vs. 10.1, p=0.196) and increasing median HOMA-IR (0.76 vs. 0.85, p=0.113), however, the statistical significance was not reached. Median DI also tended to increase (5.26 vs. 6.26, p=0.094). Only posttranfused IGI was correlated with posttransfused Hb (r=0.34, p=0.017).

**Conclusions**: Acute iron loading following blood transfusion potentially aggravated insulin resistance in β -thalassemia/HbE patients and might result in a compensatory increase in insulin secretion.

## P2-1-18 The association between frequency of Self-monitoring of blood glucose and optimal glucose control in youth with type 1 diabetes youth

### Eun Young Joo^1^, HJ Kim^1^, YH Hong^2^, YL Shin^2^, JE Lee^1^

#### ^1^Department of Pediatrics, Inha University College of medicine; ^2^Department of Pediatrics, Soonchunhyang University college of medicine

**Objective**: More frequent self-monitoring of blood glucose testing (SMBG) has been founded to be associated with lower HbA1c levels in studies of youth with type 1 diabetes (T1DM). We evaluated the relationship between the daily number of SMBG measurements and HbA1c level in T1DM adolescents. For optimal glycemic control of T1DM adolescents, the factors in related to frequency of SMBG were assessed.

**Method** : This study was administered among youth with type 1 diabetes (n=61, 13-19yr) at an outpatient clinic or diabetic camp with single structured self report questionnaires.

**Results**: Mean daily number of SMBG measurement was 3.6 ± 2.2 times and mean HbA1c level was 8.5 ± 1.4%. A higher daily number of SMBG measurement were associated with a lower HbA1c (p < 0.001). Odds ratio of the group with frequent testing ≥ 5 times a day for predicting HbA1c < 8% was 3.69 (95% CI 1.093-12.477). Frequency of daily SMBG in school was strong positively correlated with a higher number of daily SMBG measurement (p < 0.001).

**Conclusion**: For performing adequate diabetic care in youth with T1DM, frequency of daily SMBG is important. The support for SMBG measurement in school might help the optimal glucose control in T1DM adolescents.

## P2-1-19 Successful of sulfonylurea treatment in a 10-yr-old boy with neonatal diabetes mellitus with KCNJ11 mutation and intermediate DEND syndrome

### Pimonsri Hantanasiriskul, Supawadee Likitmaskul, Jeerunda Santiprabhob

#### Division of Endocrinology and Metabolism/ Department of Pediatrics, Faculty of Medicine Siriraj Hospital, Mahidol University

**Background**: Permanent neonatal diabetes mellitus (PNDM) is a rare form of diabetes, characterized by the onset of diabetes before age of 6 months. 50% of cases are caused by an activating mutation in KCNJ11, encoding the Kir6.2 subunit of ATP-sensitive potassium (KATP) channel. 20% have associated neurologic features like DEND (Developmental Delay, Epilepsy, Neonatal Diabetes) or intermediate DEND (iDEND) syndrome. Now it is known that the majority of PNDM patients with KATP mutations respond to oral therapy which is sulfonylurea.

**Case presentations:** A 4-week old male baby, presented to the hospital for high grade fever, during the work up was found to have persistent hyperglycemia so the diagnosis of neonatal diabetes was made. His past medical history was significant for intrauterine growth restriction (birth weight 1850 g:< 3rd percentile. He was started with conventional insulin regimen until 8 yr old. We first saw him when he was 9 yr old, then switched him to basal bolus regimen. He had markedly developmental delayed, functioning as 1 yr 6 mo old. EEG was normal. Anti-insulin and anti-GAD autoantibodies were negative. Genetic study for neonatal diabetes was done at 9 yr and 5 mo old, showed a heterozygous missense mutation of KCNJ11, encoding Kir6.2, c.175G>A (p.Val59Met).

Transition of treatment to oral sulfonylurea (glibenclamide) was attempted at 10 yr old. Glibenclamide was initiated at 0.2 mg/kg/day divided to 2 equal doses. Dose was gradually increased to 2.8 mg/kg/day over 3 weeks and then insulin was stopped. Blood glucose was well controlled without episodes of hypoglycemia. HbA1c has been lower than when the patient was on insulin injection regimen, as shown on table 1. No other side effects of sulfonylurea have been noticed, such as leukopenia and elevated liver enzymes.

**Conclusion**: W e report the PNDM case due to KCNJ11 mutation with intermediate DEND syndrome w ith successfully switching from insulin injection to sulfonylurea at 10 years of age. An early genetic diagnosis provides clinicians w ith valuable information for the patient’s clinical management, suggesting the possibility of treatment change and awareness of development of neurological features.

**Consent for publication:** The authors declare that written informed consent was obtained for publication.

**Table 1 (abstract P2-1-19).**
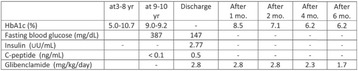
Glycemic control and glibenclamide dosage before and after swithching to sulfonylurea

## P2-1-20 DKA with Severe Acute Kidney Injury in an Adolescent Boy: a case report

### Kansuda Ariyawatkul

#### Department of Pediatrics, Panyananthaphikkhu Chonprathan Medical Center Srinakharinwirot University

**Background**: Acute kidney injury (AKI) is a rare complication of diabetic ketoacidosis (DKA) in children and adolescent. Reported mortality in AKI complicating DKA was 40%.

**Objective**: To describe our experience of treating acute kidney injury in an adolescent boy with severe DKA as first manifestation of diabetes.

**Results**: A 13-year-old boy was presented at our pediatric emergency with decreased level of consciousness and Kussmaul breathing. We reviewed there was a history of abdominal pain, polyuria, polydipsia and significant weight loss. When he arrived at hospital, he was stupor and severe dehydration. Vital signs revealed tachycardia and high blood pressure. On admission, our patient was found to have severe DKA. Laboratory investigation revealed serum urea of 27 mg/dL and creatinine of 1.11 mg/dL. Intravenous fluid resuscitation was administered immediately, followed by continuous insulin infusion and potassium chloride. On the next day, his renal function deteriorated with serum urea of 40 mg/dL, creatinine 3.7 mg/dL. His urinalysis showed 3+ glucose, 2+ protein and 3+ ketone without RBC and WBC. Fractional excretion of sodium and urea were calculated and equivalent to 22.3% and 56.3%, respectively. Potassium phosphate was initiated along with intravenous fluid. Although his DKA and level of consciousness improved and he had good urine flow, his serum creatinine progressively increased within the first five days with maximum serum creatinine of 7.77 mg/dl. Ultrasonography of both kidneys showed prominent in size with slightly increased parenchymal echogenicity of both kidneys. Potassium phosphate and bicarbonate were given to correct electrolyte disturbance. Amlodipine was started to reduce blood pressure and nephrotoxic drug was avoided. Then his serum creatinine gradually declined to normal range within 16 days without renal replacement therapy, in conjunction with amlodipine and bicarbonate were discontinued. He was discharged home with basal-bolus insulin regimen and follow up 1 month later. His blood pressure was 106/61 mmHg and serum creatinine was 0.6 mg/dL.

**Conclusion**: The acute kidney injury in DKA possibly result from decreased intravascular volume due to osmotic polyuria and gastrointestinal loss. Early recognition of AKI along with appropriate fluid and electrolyte management is important to prevent deterioration in renal function.

**Fig. 1 (abstract P2-1-20).**
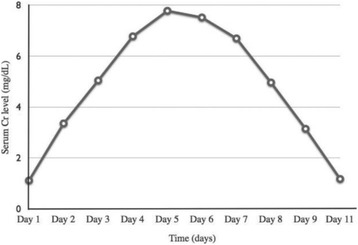
See text for description

## P2-1-21 2 Cases Suffered from Type 1 Diabetes Mellitus with Ketotic Acidosis and Henoch-Schöenlein Purpura

### Jia Jia Chen

#### Division of Endocrinology and Metabolism, Beijing Children's Hospital

Type 1 Diabetes Mellitus (T1DM) and Henoch-Schonlein purpura (HSP) are autoimmune diseases. Here we presented two boys with diabetic ketosis and HSP. The two boys with past medical history of T1DM more than 3 years were admitted for the main symptoms of abdominal pain, vomiting. The colicky abdominal pain associated with diffuse was acute and the parts changing and was not fixed, however, slight abdominal signs with only mild tenderness and muscle tension. The rash occurred after abdominal pain. And in the course of the disease, gastrointestinal bleeding happened. Serum IgEs in the two cases were significantly elevated, while 1 case of IgA increased. Complement and CD series of the two cases were normal. Parenteral nutrition support was used for the two boys with severe gastrointestinal symptoms. After the symptoms remissioned, we gave them gradually sugar-free rice soup, rice porridge, free of animal protein diets without sugar residue. Glucocorticoid 1-2 mg/ (kg. d) intravenous used actively could prohibit the inflammatory response. At the same time, the two boys with T1DM, intravenous insulin was used to control blood sugar. Until the gastrointestinal symptoms and signs disappeared respectively on the 37th day and the 20th day in hospital, with the intravenous nutrition stopping, insulin subcutaneous injection 4 times/day insteaded. The amount of insulin were 0.72 IU/kg. (d) and 1.18 U/kg. (d) with dietary calorie equivalent to 50% of the normal. So, when the T1DM patient with complaints such as abdominal pain, vomiting, we need to pay attention to the possibility of precipitated by HSP. Patient with severe intestinal mucosa involvement, the treatment with glucocorticoid actively is needed with parenteral nutrition, while controlling blood sugar has more difficulty.

## P2-2-1 Greater maternal BMI early in pregnancy is associated with increased adiposity in 7-year-old offspring, but without adverse effects on metabolism

### Valentina Chiavaroli, Jose’ G B Derraik, Sarah A Hopkins, Raquel O Rodrigues, Wayne S Cutfield, Paul L Hofman

#### University of Auckland, Liggins Institute

**Background**: There is growing evidence that maternal overweight or obesity during pregnancy is associated with an increased risk of obesity in their children. However, gestational weight gain is a confounding factor, and it is unclear whether there are associated metabolic effects on the offspring. Thus, we aimed to examine whether maternal BMI at 20 weeks of gestation was associated with alterations in body composition and metabolism in the offspring of primiparous mothers who participated in a randomised controlled trial of exercise regimen during pregnancy.

**Methods**: Of the initial 84 women and their offspring who participated in the trial, follow-up data were available on 56 mothers and their children. 28 mothers were overweight/obese early in pregnancy (approximately 20 weeks of gestation) and 28 were of normal weight, with a similar proportion of participants in each group who exercised during pregnancy (61% and 57%, respectively). Children underwent clinical assessments at approximately 7.6 years, including body composition by DXA and fasting blood tests (i.e. glucose, insulin, and lipid profile), while their nutritional intake was estimated from food diaries. All statistical analyses were adjusted for important confounders, in particular gestational weight gain.

**Results**: Greater maternal BMI was associated with increased weight SDS ( β =0.107; p=0.0004), BMI SDS ( β =0.093; p=0.002), percentage body fat ( β =0.546; p=0.006), and android fat ( β =0.528; p=0.040), but lower percentage lean mass ( β ;=-0.486; p=0.007) in the offspring in childhood. Children born to overweight/obese mothers were heavier (weight SDS 0.71 vs 0.06; p=0.002) and had greater BMI SDS (0.41 vs -0.21; p=0.003). There were no differences in fasting metabolic parameters in children born to normal weight or overweight/obese mothers. However, despite a similar energy intake, children of overweight/obese mothers had a lower daily intake of dietary fibre (18 vs 23 g; p=0.009) and a greater proportion of their energy intake was derived from fats (32% vs 26%; p=0.008), including saturated fats (15% vs 12%; p=0.027).

**Conclusions**: Greater maternal BMI early in pregnancy is associated with increased adiposity in the offspring at age 7.6 years independently of gestational weight gain, but without observed adverse effects on fasting metabolic parameters. However, the increased adiposity in the children of overweight/obese mothers in our cohort may be in part explained by poorer dietary habits.

## P2-2-2 Left ventricular mass in offspring of diabetic mothers: at 5-7 years old

### Rista Lestari, Noormanto, Madarina Julia

#### Department of Child Health, Faculty of Medicine, Universitas Gadjah Mada/ Dr. Sardjito Hospital

**Background**: Newborns of diabetic mothers have increased risk for cardiac left ventricular (LV) hypertrophy. Diabetic pregnancy is also associated with increased risk for obesity and hypertension, as well as for later cardiovascular morbidity and mortality.

**Objectives**: This study aimed to see the interaction between being the offspring of a diabetic pregnancy, hypertension and obesity to the risk to have increased left ventricular mass (LVM) and altered LV geometry in childhood.

**Methods**: We conducted a retrospective cohort study on 23 offspring of diabetic mothers and 23 sex and age matched control children at the age of 5-7 years old. LVM and LV geometry were assessed using M-mode echocardiography and indexed for height2.7. Data analyses were adjusted for birth weight, current overweight/ obesity status and blood pressure.

**Results**: Prevalence of increased LVM/height2.7 was higher in children of diabetic mothers, i.e 43.5% vs. 8.7% in the control group, RR (95%CI) 5.0 (1.2-20.4), p < 0.007. The association between maternal diabetes and increased LVM persisted after adjustment for age, sex, birth weight, current overweight/ obesity status and blood pressure, regression coefficient (95%CI): 5.7 (1.4-10.1), p=0.01. Together, maternal diabetes, overweight/ obesity status and blood pressure contributed 50% to the increase. Children of diabetic mothers were more likely to have altered LV geometry, RR (95%CI) 6.0 (1.5-23.9), p < 0.001.

**Conclusion**: Maternal diabetes is a risk factor for increased LVM and altered LV geometry in childhood.

## P2-2-3 Clinical characteristics in schoolchildren with renal glucosuria detected by a urine glucose screening program at schools in Tokyo metropolitan area during 2000-2016

### Tatsuhiko Urakami, Yusuke Mine, Masako Aoki, Misako Okuno, Junichi Suzuki

#### Department of Pediatrics, Nihon University School of Medicine

Renal glucosuriais is considered a rare condition with decreased renal tubular resorption of glucose in the absence of hyperglycemia. On the other hand, we have conducted an annual urine glusoce screening program at schools, and detected children with diabetes at the early stage. We also have identified some cases with renal glucosuria based on a positive result for glucosuria with normal glucose metabolism. We hereby report clinical characteristics in schoolchildren diagnosed with renal glucosuria detected by the screening program from 2000 to 2015. During the study period, 3,309,631 children, 6-15 years old, participated in the screening program, and 350 showed positive results for glucosuria. We performed an OGTT to evaluate glucose intolerance. As a result, 102 children were diagnosed as diabetes, and 246 (70.3%) were finally identifies as renal glucosuria. Frequency of male was 50.3%, and the mean age at diagnosis was 11.0 ± 2.4 years. Forty (11.4%) children had overweight (BMI SDS>+2.0), whereas only 5 (1.3%) had underweight (BMI SDS<-2.0). 251 (71.7%) had family members suspecting to have this disorder in the first- and second-degree relatives. All cases showed normal glucose tolerance in the absence of insulin resistance and impaired insulin secretion. In conclusion, renal glucosuria is revealed not a rare condition among schoolchildren with glucosuria. This disorder seems to be strongly inherited and to show less commonly body weight loss despite continually excretion of glucose in urine.

## P2-2-4 Strikingly high prevalence of non-alcoholic fatty liver disease in overweight Indian adolescents: Clinical and biochemical correlates

### Vandana Jain^1^, Manisha Jana^2^, Babita Upadhyaya^1^, Nayeem Ahmad^1^, Oshima Jain^1^, Ashish Upadhyaya^3^

#### ^1^Department of Pediatrics, All India Institute of Medical Sciences; ^2^Department of Radiology, All India Institute of Medical Sciences; ^3^Department of Biostatistics, All India Institute of Medical Sciences

**Objectives**: We aimed to assess the prevalence of non-alcoholic fatty liver disease (NAFLD) among overweight adolescents, and to study its association with clinical and biochemical parameters.

**Methods**: 218 overweight adolescents (151 boys) aged 10 to 16 y were recruited. BMI, waist circumference, BP and body fat (FM%) were measured. Standard deviation scores (SDS) were calculated for BMI and WC using Indian reference data. NAFLD was diagnosed and graded by ultrasonography. Fasting blood sample was drawn for glucose, ALT, AST, insulin, triglyceride, apolipoprotein C3 (ApoC3), tumor necrosis factor α (TNF α ), adiponectin, total and high- density lipoprotein (HDL) cholesterol. Homeostatic model assessment of insulin resistance (HOMA-IR) was calculated. Parents’ BMI was checked and USG for NAFLD done. Biochemical and clinical variables were compared between the two groups. ROC analysis and logistic regression was done to find out parameters associated with NAFLD.

**Results**: Mean age was 11.9 ± 1.6 y and BMI SDS 2.3 ± 1.1. NAFLD was seen in 62.5% adolescents (95% C.I. 56.2 – 69.4%); being mild in 40.4%, moderate in 18.8% and severe in 3.3%. Among the other complications of overweight, low HDL (3.5) in 43.5%, hypertension in 22.9%, impaired fasting glucose in 9.3%, and metabolic syndrome (by IDF criteria) in 26%. Comparison of clinical and biochemical parameters in adolescents with and without NAFLD are presented in Table 1. ROC analysis showed that WC SDS > 1.4, ALT > 33U/L, BMI SDS >2.2, body fat% >37.7, Insulin >15 and HOMA-IR >3.2 were associated with NAFLD with AUC of 0.73, 0.70, 0.69, 0.63 and 0.68, respectively. On multiple logistic regression, BMI SDS, ALT and insulin were independently associated with NAFLD with odds ratios (95% CI) of 2.92 (1.53-5.59), 2.72(1.42-5.21) and 1.97(1.02-3.80), respectively.

**Conclusions**: Strikingly high prevalence of NAFLD was noted among overweight Indian adolescents. BMI and waist circumference SDS, FM%, BP, acanthosis nigricans, ALT, AST, HOMA-IR and insulin were higher among adolescents with NAFLD while age, gender distribution, pubertal status, lipid profile, adiponectin, TNF- α and parents/ BMI and fatty liver status were similar. NAFLD was independently associated with BMI, serum ALT and insulin.

**Table 1 (abstract P2-2-4).**
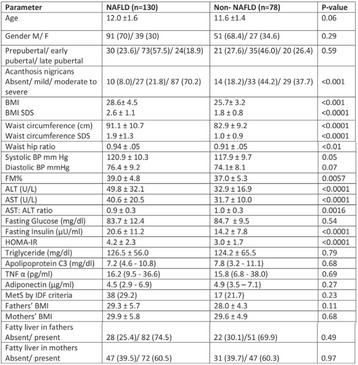
Comparison of clinical and biochemical parameters between subjects with and without NAFLD (USG done in n=208 adolescents)

## P2-2-5 Use of Professional Continuous Glucose Monitoring in Children with Type 1 Diabetes Mellitus: An Open Label Randomized Control Trial

### Ravi K Teja^1^, Rakesh Kumar^1^, Devi Dayal^1^, Naresh Sachdeva^2^

#### ^1^Department of Paediatrics, Post Graduate Institute of Medical Education and Research; ^2^Department of Endocrinology, Post Graduate Institute of Medical Education and Research

**Background**: Frequent self monitoring of blood glucose (SMBG) is the only accurate method available for insulin dose titration T1DM patients. Professional continuous glucose monitoring (p-CGM) is blinded recording of glucose trends over 5-7 days and helps physicians to guide insulin titration to patient.

Objective: To assess efficacy of insulin dose adjustments, based p-CGM plus SMBG in improving glycemic control compared to SMBG alone.

**Methods**: Trial design: Open label, randomized control trial (CTRI Ref. No 2015/04/008867)

**Participants**: Children (2 - 10 years) with T1DM for at least 6 months, on basal-bolus insulin regimen and SMBG for at least 3 times a day. Children having DKA within 2 months were excluded.

**Intervention**: Subjects in the Intervention group were placed on p-CGM (iPro®2 Professional CGM, Medtronic, USA) for 3-5 days along with regular SMBG. Data from p-CGM was analyzed by physician and used to guide insulin titration along with SMBG over following 3 months. Control group had only SMBG for titrating insulin doses.

**Outcome**: Change in HbA1c 3 months after p-CGM.

**Randomization**: Done using computer generated random number list. Group allocation was concealed from investigator and participants using opaque sealed envelopes.

**Results**: Numbers randomized : Out of 310 patients screened, 68 eligible patients were randomized, 34 each to either arms (Figure 1). Recruitment: closed

**Numbers analyzed** : Thirty children in intervention group and 33 in control group. Intention to treat analysis was also performed. Outcome: There was more decreased unit change in HbA1c, percentage of low sugar records and total insulin requirement per day, after 3 months follow-up, in intervention group. However, difference was not significant except for total insulin Units/kg/day (p=0.014). In sub-group analysis of children with baseline HbA1c > 7.5%, there was a significant mean fall of HbA1c by 1.27%.

**Harms**: Accidental removal of p-CGM in 2 patients and for hospitalization in one. P-CGM caused local redness (10/34) and local irritation (12/34).

**Conclusions**: Addition of p-CGM along with SMBG may help in adjusting insulin dose more effectively especially in children with higher baseline HbA1c.

**Fig. 1 (abstract P2-2-5).**
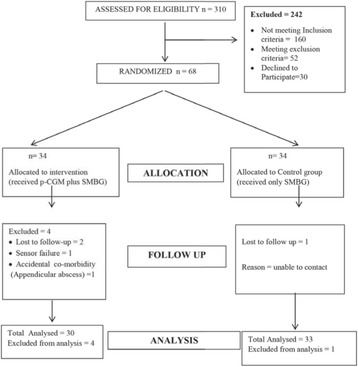
Study flow diagram

## P2-2-6 Diabetic screening system for school children in Atsugi city

### Maki Saito^1,2^, Takanori Motoki^2^, Akiro Ito^1,2^, Takeru Ito^1,2^, Asako Tajima^2^, Aizan Hirai^3^, Naoko Tajima^4^, Ichiro Miyata^2^

#### ^1^Department of Pediatrics, Atsugi City Hospital; ^2^Department of Pediatrics, The Jikei University School of Medicine; ^3^Chiba Cerebral and Cardiovascular Center; ^4^Department of endocrinology, The Jikei University School of Medicine

**Background**: In Japan, diabetic screening for school children using urinalysis has been performed to detect diabetes mellitus during childhood throughout the country. However, inadequate follow-up system after urinary screening caused several problems. The rate of participation in workup examination still remains less than 30%. Also, accurate annual incidences of children with type 1 diabetes mellitus (T1DM) and type 2 diabetes mellitus (T2DM) detected by screening of urine glucose have not been determined.

**Objective**: Under the cooperation with Atsugi city (population: around 224,000), the present study was performed to achieve full participation of children with positive urine glucose in workup examination, and to clarify accurate annual incidences of children with T1DM and T2DM in this city.

**Subjects and Method:** Subjects were school children with positive urine glucose for the past eight years from 2009 through 2016 in Atsugi city. Workup examination of diabetes mellitus, including HbA1c and anti-GAD antibody, was carried out for them at our hospital. And then we retrospectively analyzed clinical diagnosis and management of them. The results of first screening tests by urinalysis were provided for us from Atsugi city.

**Results**: A total of 146,866 school children were tested by diabetic screening system. The average participation rate for eight years was 85%. 41 of these children showed positive response of urine glucose. Continuously, all of them could receive secondary workup examination at our hospital by active support of Atsugi city. Through this screening system, four children were finally diagnosed as having T2DM. The overall incidence of T2DM was 2.70 per 100,000 per year. On the other hand, three children were diagnosed as having T1DM. The overall incidence of T1DM was 2.09 per 100,000 per year. Furthermore, one child was diagnosed as having insulin receptor abnormality (Rabson- Mendenhall syndrome), and another one was found before onset of T1DM.

**Conclusion**: We could achieve improvement of diabetic screening system for school children by close cooperation with a local government of Atsugi city, and then found eight patients with various types of DM. Our study demonstrated that the annual incidence of children with T2DM in Atsugi city was comparable with those in other municipalities (2.65-3.57 per 100,000 per year), but interestingly, the annual incidence of children with T1DM was almost 4 times higher than that in Tokyo (0.51 per 100,000 per year). The reason for this difference is unclear, and therefore further study is necessary.

## P2-2-7 Glycemic Control Indicators Levels at Diagnosis of Neonatal Diabetes Mellitus: Comparison with Other Types of Insulin- Dependent Diabetes Mellitus

### Shigeru Suzuki^1^, Akiko Furuya^1^, Yusuke Tanahashi^1^, Hiroshi Azuma^1^, Yukihiro Bando^2^, Soji Kasayama^3^, Masafumi Koga^4,5^

#### ^1^Department of Pediatrics, Asahikawa Medical University; ^2^Department of Internal Medicine, Fukui-ken Saiseikai Hospital; ^3^Department of Medicine, Nissay Hospital; ^4^Department of Internal Medicine, Kawanishi City Hospital; ^5^Department of Internal Medicine, Hakuhokai Central Hospital

**Background**: Neonatal diabetes mellitus (NDM) is a monogenic diabetes whose onset is within 6 months of age. The HbA1c and glycated albumin (GA) levels in NDM are falsely low because of high hemoglobin F levels and high albumin metabolism, respectively in early infancy. Instead, we reported that the Age-adjusted GA (Aa-GA) accurately reflected glycemic control in NDM. Glycemic control indicators (HbA1c or GA) reflect both plasma glucose and the duration of hyperglycemia in the manner of the weighted mean of the plasma glucose level. Therefore, glycemic control indicators levels at diagnosis of diabetes indicate what extent or how long hyperglycemia continues until diagnosis. However, the time course of glycemia until diagnosis of NDM, which is one of the insulin dependent diabetes, are unclear.

**Objective**: To compare glycemic control indicators levels at diagnosis between NDM and other types of insulin-dependent diabetes mellitus (fulminant type 1 diabetes [FT1D] and acute-onset autoimmune type 1 diabetes [T1AD]).

**Subjects and Methods:** The subjects consisted of eight NDM patients, eight FT1D patients, and 24 T1AD patients. Plasma glucose, HbA1c, GA and Aa-GA, which was calculated as previously reported (Aa-GA = GA x 14.0 / [1.77 x log-age (days) + 6.65].), were compared.

**Results**: Plasma glucose levels in the FT1D were significantly higher than in the T1AD, while there were no significant differences between the plasma glucose levels in the NDM and those in the FT1D or the T1AD. HbA1c and GA levels in the NDM were not significantly different from those in the FT1D, respectively, and both indicators were lower than those in the T1AD. On the other hand, Aa-GA levels in the NDM were not significantly different from those in the T1AD, and were higher than those in the FT1D.

**Conclusions**: These findings suggested that the time-course of the elevation of plasma glucose in NDM at diagnosis was close to that of T1AD. In addition, the high Aa-GA value in NDM at diagnosis for NDM indicated that it is useful for assessing the chronic hyperglycemic conditions in NDM.

## P2-2-8 Effectiveness of optical coherence tomography angiography (OCTA) for evaluating diabetic retinopathy in childhood- onset diabetes

### Masako Aoki^1^, Satomi Tanabe^1^, Yusuke Mine^1^, Misako Okuno^1^, Junichi Suzuki^1^, Tatsuhiko Urakami^1^, Hajime Onoe^2^, Yorihisa Kitagawa^2^, Hiroyuki Shimada^2^

#### ^1^Department of Pediatrics and Child Health, Nihon University School of Medicine; ^2^Department of Ophthalmology, Nihon University School of Medicine

**Background**: Early detection and prevention of diabetic retinopathy (DR) is crucial to reduce the risk of severe visual disturbance. Early stage of DR is characterized by pathological changes in macula. Optical coherence tomography angiography (OCTA) is a newly developed instrument which can precisely assess the area around macula. It does not require intravenous administration of a contrast medium and takes only 5-10 minutes for inspection, implying that less-invasive and faster than Fluorescein angiography. Even though there are increasing reports on OCTA in ophthalmology, clinical experience is still lacking in pediatric field. Thereby, we present our experience of OCTA for DR screening in patients with childhood-onset diabetes.

**Subjects and Methods:** OCTA data was collected from 1st January 2016 to 30st July 2016. 47 patients with diabetes who had been diagnosed under 15-year-old were recruited. They consisted of 36 patients with type 1 diabetes, 7 with type 2 diabetes, and 4 with maturity-onset diabetes of the young (MODY). There were 15 males and 32 females, diabetic durations were 10.3 ± 11.0years, average HbA1c (among 1-year) was 8.2 ± 1.6%. The patients underwent ophthalmoscope prior to OCTA. Data on diabetes duration, urine examinations and HbA1c levels were collected from hospital records.

**Results**: DR was detected in 12 patients. Simple DR (SDR) was observed in 50% (6/12), pre-proliferative DR (PPDR) was observed in 17% (2/12), proliferative DR (PDR) was observed in 33% (4/12) of the cases. 4 out of 6 SDR were detected using OCTA for the first time in this study period. The prevalence of DR was higher in patients who were older and had longer diabetes durations, but we found SDR in 16-year- old patient with poor glycemic control in 4-year duration of diabetes. The youngest patient who were performed OCTA was 4 years old. There were no adverse symptoms related to OCTA.

**Discussion**: Overall, in our cohort, OCTA was safe and non-invasive. Furthermore, it can easily detect early-stage DR lesions in macular area. OCTA in combination with ophthalmoscopy seems to be effective for evaluating DR. We detected SDR in an adolescent patient with poor glycemic control. OCTA seems to be effective as the additional DR screening for young patients with poor glycemic control.

**Conclusion**: OCTA is enforceable with young patients. It seems to be useful to use OCTA in combination with ophthalmoscopy as a precise and non-invasive screening for DR.

## P2-2-9 Triglycerides/HDL cholesterol ratio and total cholesterol/HDL cholesterol ratio : surrogate markers for metabolic syndrome in adolescents

### Shin-Hye Kim, Mi-Jung Park

#### Department of Pediatrics, Sanggye Paik Hospital, Inje University College of Medicine

**Background**: Reference values of triglycerides (TG) to high density lipoprotein cholesterol (HDL-C) ratio, total cholesterol (TC) to HDL-C ratio, and non-HDL-C concentrations, and their clinical utility have not been fully examined in adolescents.

Objectives: We aimed to examine the distribution of TG/HDL-C ratio, TC/HDL-C ratio, and non-HDL-C concentrations in Korean adolescents, and to explore their significance as surrogate markers for metabolic syndrome.

**Methods**: We analyzed data for 2,721 adolescents aged 10-18 years (1,436 boys and 1,285 girls) who participated in the Korean National Health and Nutrition Examination Surveys from 2008 to 2010. Metabolic syndrome was defined using the International Diabetes Federation criteria.

**Results**: The mean values of TG/HDL-C, TC/HDL, and non-HDL-C were 1.9, 3.3 and 108.6 mg/dL, respectively. TG, TG/HDL-C, and TC/HDL-C were not significantly different by gender, but non-HDL-C levels significantly higher in girls than in boys. TC/HDL-C ratio did not change with age in both genders. Non-HDL-C significantly decreased with age in boys, while age-related difference was not found in girls. TG and TG/ HDL-C increased with age in boys, not in girls. Compared with the other lipid parameters, correlation coefficient with waist circumference (WC) was highest for TC/HDL-C, while correlation coefficients with the homeostatic model assessment–insulin resistance (HOMA-IR) were highest for TG and TG/HDL-C. The areas of under the receiver operating characteristic (ROC) curve for predicting metabolic syndrome of TG (0.923), TG/HDL-C (0.947), and TC/HDL-C (0.924) were higher than HOMA-IR (0.822). Optimal cutoffs (sensitivity, specificity) of TG, TG/HDL-C, and TC/HDL-C for identifying adolescents with metabolic syndrome were 145 (82.5% 90.5%), 3.4 (85.0%, 91.0%), and 4.0 (85%, 85.6%), respectively.

**Conclusions**: TG/HDL-C and TC/HDL-C ratio might be useful surrogate markers for metabolic syndrome in Korean adolescents.

## P2-2-10 Our Experience of using Sensor Augmented Pump

### Yuko Hotta^1^, Tomoyuki Kawamura^1^, Kayako Hashimura^1^, Masahiko Joo^1,2^, Yoneo Kashihara^1,3^, Tomomi Hashimoto^1,3^, Masakazu Hirose^1^, Takashi Higashide^1,3^, Mayumi Aono^1,4^, Shigeo Aono^1,4^, Haruo Shintaku^1^

#### ^1^Department of Pediatrics, Osaka City University Graduate School of Medicine; ^2^Departement of Pediatrics, Japan Community Health care Organization Kyushu Hospital; ^3^HUG HUG Kids Clinic; ^4^Teradacho Kodomo Clinic

**Background**: In October 2014, we started to introduce the sensor augmented pump (SAP: Minimed 620G with Enlite®) for patients with type 1 diabetes for the first time in Japan. SAP which is available in Japan is only Minimed 620G with Enlite®. So far about 100 patients in our hospital had started to use SAP by April 2016, whereas over 30 patients stopped using them.

**Methods**: We retrospectively analyzed the clinical features of patients who started SAP. The reasons why some patients stopped SAP were examined. Then we identified 35 patients who had used SAP for more than one year (aged 2 to 75, HbA1c 7.63 ± 1.14 %). We retrospectively examined whether their HbA1c had improved or not. The factors which might affected on the blood glucose control were analyzed. Then we asked them how they felt about SAP by the questionnaire.

The major reasons for stopping SAP were the itching and the bothering by wearing the sensors. The level of HbA1c in 35 patients who continued SAP for 1 year, had not changed (7.63 ± 1.14 % to 7.65 ± 1.03 %). Their average blood glucose level and standard deviation had not changed (192.4 ± 81.3 to 180.7 ± 70.3). Some patients, whose HbA1c were high level before using SAP, improved significantly. The using time of the sensors per week, age, nor sex were not related to the improvement of HbA1c.

Their satisfaction about 620 G with Enlite was high (7-8 points at ten points of perfect scores), even though HbA1c in many patients was not improved. Almost all of them answered that they felt that it was comfortable and convenient to use SAP, because of showing glucose level and its trend.

Thirty % of patients stopped using 620 G with Enlite system because of the sensor troubles. Thirty-five patients so far continued using it for more than 1 year. SAP had not changed blood glucose control significantly in one year. However, many patients felt highly satisfaction to use the SAP system.

## P2-2-11 Insulin pump-associated adverse events are common, but not associated with glycemic control, socioeconomic status, nor pump/infusion set type

### Phillipa Ross^1^, Andrew R Gray^2^, Jackie Milburn^3^, Maran I Kumarasamy^4^, Fiona Wu^4^, Stephanie Farrand^5^, Jeremy Armishaw^6^, Esko Wiltshire^7^, Paul Tomlinson^1^, Benjamin J Wheeler^1,3^

#### ^1^Department of Women's and Children's Health, Dunedin School of Medicine, University of Otago; ^2^Department of Preventive and Social Medicine, University of Otago; ^3^Paediatric Endocrinology, Southern District Health Board; ^4^Auckland District Health Board, Diabetes Centre; ^5^Department of Endocrinology, Southern District Health Board; ^6^Department of Paediatrics, Tauranga Public Hospital; ^7^Department of Children's Health, University of Otago

**Background**: Insulin pumps are commonly used in the management of type 1 diabetes mellitus (T1DM). While there have been many outcome-focussed studies, only a few focused on potential adverse events (AEs), with none examining the relationship between AEs and pump/infusion set type, ethnicity nor socioeconomic status. In addition, current data on the incidence and characteristics of pump-associated AEs are confined to one paediatric centre.

**Subjects and Methods:** We approached adults, and families of children with T1DM on insulin pumps in four main New Zealand centres. Participants completed a questionnaire examining pump-related issues they had experienced in the preceding 12 months.

**Results**: Response rate was 64% with 174 of 270 eligible people participating in the study. 84% of subjects reported one or more AEs, with an overall AE incidence of 3.42 per person/year (95% CI 3.14, 3.73). An event serious enough to require a hospital presentation occurred in 9.8%, all but one reporting high ketones or diabetic ketoacidosis (DKA). Set/site problems were the AE most commonly reported (by 53% of respondents), followed by cutaneous complications (43%) and pump malfunction (38%). Few predictors of AEs (of any type) were found, however a negative binomial regression model found that a longer duration of pumping (p=0.018) and age

**Conclusions**: Insulin pump-associated AEs are very common. However, few variables are predictive of them with no relationships seen with glycaemic control, socioeconomic status, pump manufacturer nor infusion set type. Based on these findings, AEs should be anticipated in both adults and children, with anticipatory patient education and training recommended for their successful and safe use.

## P2-2-12 Evaluation of β-cell function by mixed meal tolerance test and oral glucose tolerance test in obese children and adolescents with metabolic syndrome compared to isolated obesity

### Hua Guo Li, Feng Xue Chen, Fen Jun Fu

#### Division of Endocrinology, Children's Hospital of Zhejiang University School of Medicine

**Background**: In the setting of insulin resistance, progressive islet β -cell dysfunction is especially important in the onset and progression of metabolic syndrome (MS) and diabetes. However, data of β -cell function in youth with MS or cMD icomponent metabolic disorders ) are very limited.

**Objective and hypotheses**: To evaluate islet β - cell function in obese children and adolescents with clinical diagnosis of MS, cMD or iOB to provide a reference for clinical interventions and early prevention.

**Method**: We prospectively designed three groups of participants for study: MS, cMD and iOB, clinically data from obese children and adolescents (50 iOB, 72 cMD, and 59 MS) between May, 2013, and May, 2015 were obtained. All of the participants underwent MMTT ithe liquid mixed-meal test ) and OGTT, indices of insulin secretory function and insulin sensitivity were calculated for study.

**Results**: Islet β -cell function relative to area under curve C-peptide at 120 minutes (P=.007 ) and peak CP (P=.007 ) derived from MMTT were significantly higher in the MS group compared with the iOB group both before and after adjusting for BMI. The MS group had higher HOMA- β - % compared with the cMD and iOB groups after adjusting for BMI (P=.036 P=.008 ). However, there was no difference between the cMD and iOB groups in AUC CP120, peak CP, and HOMA- β - %. The Δ I30/ Δ G30 and oDI derived from OGTT were not different among the three groups.

**Conclusion**: Obese children and adolescents with MS and cMD still had a commendable pancreatic β - cell function in response to their severe insulin resistance, offering a window in time for intervention before development of diabetes.

## P2-2-13 Associated Chemerin Level And Homeostasis Model Assesment Insulin Resistance In Obese Adolescent

### Eka Agustia Rini^2^, Silvy Dioni^1^, Eti Yerizel^1^

#### ^1^APPES, Paediatric Endrocrinology M. Djamil Hospital Padang; ^2^ISPAD, Paediatric Endocrinology M. Djamil Hospital Padang

**Background**: Childhood obesity associated with an increased risk for metabolic syndrome, such as insulin resistance. Homeostasis Model Assesment Insulin Resistance (HOMA-IR) is a chemical marker widely used for insulin resistance. Chemerin is a 18 kDa protein produced by adipose tissue, function as chemoatractant plays an important role contributing to the development of inflammation and insulin resistance.

**Objective**: To determine assosiation chemerin level and HOMA-IR in obese adolescent.

**Methods**: This was a cross-sectional study and conducted in three Senior High School in Padang. Twenty eight obese adolescents and 28 control were enrolled in the study. The age of the subjects ranged from 14-18 years. Obesity criteria was measure based on body mass index (BMI). Fasting serum glucose level measured by glucose hexokinase fotometrik method and serum insulin was measured by chemiluminessence immunoassay method. Plasma chemerin was measured with enzyme-linked immunosorbent assay (ELISA). The insulin resistance index was estimated from fasting serum insulin and glucose level using the formula.Data was analyzed using correlation test and t-test, P<0,05 was significant.

**Result**: The plasma chemerin level was higher in the obese than in the control group 121,52 (SD 2,09) ng/ml vs 97,23 (SD 2,41) ng/ml, p: 0,001, and the insulin resistance group had higher plasma chemerin level than non resistance group 133,1(SD 19,24) vs 115,09 (SD 19,52), p: 0,027. There is a weak correlation with the value chemerin levels HOMA-IR in obesity (r = 0.382; p = 0.045) and weak correlation with value chemerin levels HOMA-IR in obese insulin resistance (r = 0.297; p = 0.405).

**Conclusion**: There is a weak correlation with the value chemerin levels HOMA-IR in obesity and insulin resistance.

**Key Words**: Obese adolescents, insulin resistance, plasma chemerin.

## P2-2-14 The levels of serum antioxidant vitamins (A, E, C) in underweight and overweight-obese children with insulin resistance

### Kezban Karabag, Zerrin Orbak

#### Department of Pediatric Endocrinology, Ataturk University Medical Faculty

Insulin resistance is a common feature of childhood obesity and is considered to have a significant relationship with Type 2 diabetes mellitus and cardiovascular disease. On the other hand, the high frequency of insulin resistance was found in malnutrition, too. Changes of antioxidant vitamins levels have been reported in children with obesity and malnutrition due to lack of food intake and chronic oxidative stres in several studies. The aim of this study is to investigate the relationship between HOMA-IR (homeostasis model assessment for insulin resistance) and serum antioxidant vitamins (retinol, α -tocopherol, ascorbic acid) levels in preschool children.

76 children in our study were classified as underweight (n=15), normal weight (n=31), and overweight-obese (n=30) using parameter of body mass index. A HOMA-IR was used for the diagnosis of insulin resistance. Insulin resistance was found in 20% of underweight children and 26,7% of overweight and obese children whereas there was no child with insulin resistance in normal weight children.

The serum vitamin E ( α -tocopherol) and vitamin A (retinol) levels weren’t found statistically different in all three groups (p>0,05). The serum vitamin C levels in normal weight group is higher than those in overweight and obese. Just in overweight-obese group HOMA-IR was negatively associated with vitamin E. Low concentration of vitamin E but not vitamin A and C in children who were underweight and overweight-obese were associated with insulin resistance.

As a result, underweight children have the risk of insulin resistance so they should be investigated for insulin resistance like obese children. Also, vitamin E may have a part in insulin resistance.

## P2-2-15 Relationship between timing of diabetes self-management education after diagnosis and glycemic control in type 1 diabetes

### Khwanhatai Khemaprasit, Supitcha Patjamontri, Pornpimol Kiattisakthavee, Jeerunda Santiprabhob, Pairunyar Nakavachara, Supawadee Likitmaskul

#### Division of Endocrinology and Metabolism / Department of Pediatrics, Faculty of Medicine, Siriraj Hospital, Mahidol University

**Introduction**: Diabetes self-management education (DSME) is a process that evaluates the patient’s self-care management to confirm that they can take care themselves at home and have good glycemic control. Although DSME was provided in most newly-diagnosed diabetic patients, some of them still have poor glycemic control. Besides the education, other factors may affect glycemic control including the timing of DSME as one of the factors. Recommendations suggest starting DSME as soon as possible after diagnosis. However, parental anxiety and depression can be found in newly-diagnosed with type 1 diabetes (T1D) within several weeks may affect the perception of DSME if it is provided too soon.

**Objective**: To evaluate relationship between timing of DSME after diagnosis and glycemic control, and to determine factors affecting glycemic control in T1D patients.

**Materials and Methods**: One hundred and ten T1D patients and their parents who received DSME in the period of 2005-2014 were interviewed at time of enrollment, data collection from medical records and HbA1c was evaluated for 2 years after DSME. Their average HbA1c in 1st and 2nd year after DSME were analyzed by divided into three groups according to timing of DSME after diagnosis: less than 1 month (N=58), 1-6 months (N=30), and over 6 months (N=22).

**Results**: The mean age at diagnosis was 8.03 ± 3.36 years and mean age at receiving DSME was 8.63 ± 3.38 years. Mean HbA1c at receiving DSME, 1st and 2nd year after DSME were 11.15 ± 2.72%, 8.65 ± 1.36% and 9.07 ± 1.40%, respectively. Comparison among timing of DSME groups, less than 1 month, 1-6 months and over 6 months after diagnosis groups were demonstrated statistic significantly better glycemic control in the 1st year (HbA1c <7.5% was 22.4%, 10.0% and 4.5%, respectively, p=0.035). In multivariate analyses of potential factors for good HbA1c in 1st year, patients who received DSME within 1 month after diagnosis were likely to have better glycemic control (adjusted OR: 10.572 [95% CI: 1.184-94.433]). Other factors associated with good glycemic control were found including high parental education (p=0.022), high family income (p=0.034), more family member received DSME (p=0.025), multiple daily injections (p=0.014).

**Conclusions**: This study demonstrated timing of initial DSME and family supports were associated with glycemic control during 1st year after DSME in T1D. Early and continuous intensive education is recommended for all T1D patients and their family members.

## P2-2-16 The Clinical Characteristics of Type 2 diabetes Mellitus in Children and Adolescents

### Koji Ohsugi, Keita Numasawa, Kanako Ebina, Kentaro Shiga, Nobuyuki Kikuchi

#### Department of Pediatrics, Yokohama City University Medical Center

**Background**: During the last three decades, the incidence of type 2 diabetes mellitus (T2DM) in children and adolescents has increased in Japan, which is related to the increasing prevalence of obesity and various related health problems. T2DM symptoms progress more slowly, compared to those of type 1 diabetes mellitus, and most patients with T2DM are identified incidentally during urine glucose screening. Nevertheless, it is difficult to motivate patients with T2DM in children and adolescents to comply with their treatment, as they are often asymptomatic.

**Objectives and methods:** This study aimed to determine the clinical and glycemic control characteristics of T2DM in children and adolescents. This study included 43 patients (20 male patients and 23 female patients) who were

**Results**: The mean age at the diagnosis was 13.1 years, the mean duration of diabetes was 3.3 years, and 88.3% of the patients were identified using urine glucose screening. The HbA1c levels at the diagnosis and most recent visit were 8.51 ± 2.7% and 7.1 ± 1.8%, respectively. Approximately 80% of the patients (95% of the male patients and 65% of the female patients) were obese (percent ideal body weight over 120%), and the frequency of a family history of T2DM was 60.5%. 65.1% of the patients were receiving oral hypoglycemic agents or insulin, and 55.9% of the patients had experienced complications (e.g., hepatic dysfunction, hyperlipidemia, hyperuricemia, and hypertension).

**Discussion**: Among patients who were treated at our center, the onset of T2DM in children and adolescents was related to the progression of obesity and genetic factors, which agrees with previously reported findings. Moreover, most of our patients with T2DM are identified incidentally using urine glucose screening performed in Yokohama City. As these patients are often asymptomatic, they may not be motivated to continue their treatment. However, most of our patients ultimately achieved glycemic control using lifestyle interventions and medication. Nevertheless, we did not consider patients who discontinued their treatment, which indicates that there may be higher incidences of poorly controlled glycemia and the progression of diabetic complications. Therefore, initial and on-going education regarding T2DM may help highlight the importance of behavioral, dietary, and physical activity changes to patients with T2DM in children and adolescents.

## P2-2-17 Possible monogenic diabetes mellitus including mody is highly prevalent in Korean children with diabetes mellitus

### JungEun Moon, kyung-Mi Jang, Eun-Mi Jo, Byung-Ho Choi, Cheol-Woo Ko

#### Division of Endocrinology and Metabolism, Kyungpook National University Children's Hospital

**Background**: As the human genome is further explored, multiple genetic anomalies at different loci are being found that confer varying degrees of predisposition to diabetes. MODY is the most common form of monogenic diabetes, accounting 2 to 5 percent of diabetes. Recently, we have found and reported three noble gene variants relating to MODY in Korean children (Shim et al, Horm Res Pediatr, 2015). Objective and hypotheses: This study was done to see the frequency of possible monogenic diabetes in Korean children with diabetes mellitus and their clinical and laboratory characteristics.

**Method**: Study patients consisted of 126 children with diabete mellitus (DM) who visited our institute between 2008 and 2015. Their medical records were reviewed retrospectively. They classified into three groups; type I (T1DM), type II (T2DM) and monogenic diabetes mellitus (MDM) including MODY by the ADA classification of DM. Various clinical and laboratory data was analyzed.

**Results**: The frequencies were 48 (38%) in T1DM, 42 (33%) in T2DM, and 36 (29%) in possible MDM. Majority of possible MDM was MODY (22 out of 36, 61%). Ages (yrs) at diagnosis were 9.2 ± 4.1 in T1DM, 13.4 ± 2.4 in T2DM, and 12.3 ± 3.1 in MDM. The age at diagnosis was significantly older in T2DM compared to T1DM (p=0.000). BMI (kg/m2) was significantly higher in T2DM compared to T1DM or MDM, 25.9 ± 4.7 vs 16.9 ± 5.1 vs 19.7 ± 5.1, respectively (p=0.001). Initial fasting insulin levels (IU/mL) were significantly higher in T2DM than T1DM or MDM, 13.01 ± 9.45 vs 2.10 ± 2.68 vs 4.85 ± 5.25, respectively (p=0.000). C-peptide levels (ng/mL) were significantly higher in T2DM compared to T1DM or MDM, 3.19 ± 1.40 vs 0.29 ± 0.15 vs 1.17 ± 0.39, respectively (p=0.000). HbA1c levels (%) were significantly higher in T1DM compared to T2DM, 13.31 ± 3.09 vs 11.22 ± 2.40, respectively (p=0.001).

**Conclusions**: It appears that possible monogenic diabetes including MODY in Korean children with DM is more prevalent than expected. Further large scaled studies including genes related to monogenic DM are necessary.

## P2-2-18 Hemoglobin A1c was dissociated from plasma glucose levels in an obese Myanmar girl with diabetes mellitus

### Yoshiro Baba^1^, Kyoko Tsukui^1^, Chihiro Nakamura^1^, Makoto Anzo^1^, Tomonobu Hasegawa^2^

#### ^1^Department of Pediatrics, Tokyo Metropolitan Ohtsuka Hospital; ^2^Department of Pediatrics, Keio University School of Medicine

## P2-2-19 Insulin regimens for newly diagnosed children with type 1 diabetes in Australia and New Zealand - a survey of current practice

### Dharrshinee Selvakumar^1^, Al-Sallami S Hesham^1^, Martin de Bock^2^, Geoffrey R Ambler^3^, Paul B Aguirre^3^, Esko Wiltshire^4^, Elaine Tham^5^, Peter Simm^6^, Louise Conwell^7^, Phillipa J Carter^8^, Jinny Willis^9^, Benjamin J Wheeler^10^

#### ^1^University of Otago, School of Pharmacy; ^2^Department of Paediatric & Child Health, Princess Margaret Hospital; ^3^Childrens Hospital at Westmead, Institute of Endocrinology and Diabetes; ^4^Department of Paediatric & Child Health, University of Otago; ^5^Department of Endorinology & Diabetes, Women's & Children's Hospital; ^6^Department of Endocrinology & Diabetes, Royal Children's Hospital; ^7^Department of Endorinology & Diabetes, Royal Children's Hospital; ^8^Starship Paediatric Diabetes & Endocrinology; ^9^Don Beaven Medical Research Centre; ^10^Department of Women's & Children's Health, University of Otago

There is no consensus on the optimal insulin treatment for children newly diagnosed with type 1 diabetes (T1DM). The aims of this study were (1) to describe the prevalence of various insulin regimen use by age, country (AU vs. NZ), and region; (2) to describe other aspects of management and education relevant to new onset T1DM by country/region.

An online survey of medical professionals caring for children with T1DM in AU and NZ was undertaken (n= 110), with questions pertaining to clinic demographics; insulin regimen/dosing choices, and education.

Response rate was 91%. Differences in regimen choice and practice were seen between countries/centres. The majority of endocrinologists and centres in Australia (69%, p<0.05) favour intensive insulin therapy (MDI/CSII) at all ages. In contrast, for NZ, and Victoria, AU, for ages. This is the first study to examine individual practice with regard to treatment choices at diagnosis of T1DM. Practice varies across Australasia by clinician and location. This lack of consensus is likely driven by ongoing debate and gaps in the current paediatric diabetes evidence base, as well as by differences in clinician/centre preference, and their interpretations of the influence of various patient factors.

## P2-2-20 Treatment of Glimepiride in 6 Neonatal Diabetes Mellitus Patients Yan Sun, Guimei Li, Xiaohong

### Shang, Yu Qiao, Xiangdeng Cheng, Fengxue Wang

#### Department of Pediatrics, Provincial Hospital Affiliated to Shandong University

**Objective**: Neonatal diabetes secondary to mutations in potassium-channel subunits is a rare disease. To study the clinical features, genetic etiology and effects of glimepiride in 6 Chinese patients.

**Methods**: We reviewed the medical records of 6 NDM patients along with their follow-up details. We recorded the HbA1c, C peptide and insulin levels, and tried to find the correlation between the genetic pothogeny and glimepiride effects.

**Results**: Among 6 NDM cases, 1case was transient, recovered at 4 months old after transient therapy of intravenous insulin. One case got treated with subcutaneous insulin. The other four were tried for glimepiride transition.All were successful with decreased HbA1c levels, increased C peptide and insulin.In which, three patients had genetic examination, one had KCNJ11 mutation, one heterozygous mutations with ABCC8 and INSR, one known mutation of E1F2AK3. KCNJ11 or ABCC8gene, encoding the ATP-sensitive potassium channel (KATP channel). The significant effect time of SU was longer, HbA1c level was higher in the E1F2AK3 mutation case than in the KATP channel mutation cases.

**Conclusion**: Oral sulfonylurea is beneficial for NDM, especially for patients with KATP channel mutations. Gene detection could give indication for precise treatment.

## P2-2-21 Sleep Disorders in Adolescents with Obesity

### Ediz Yesilkaya^1^, Cengizhan Acikel^2^, Sirzat Yesilkaya^3^, Gokalp Basbozkurt^4^, Celal Saglam^4^, Erkan Sari^1^, Oguzhan Babacan^1^, Ahmet Bolat^4^, Kursat Fidanci^4^, Omer Saglam^6^, Galip Erdem^4^, Gulcin Sari^3^, Berna Fidanci^5^

#### ^1^Division of Endocrinology and Metabolism/Department of Pediatrics, Gulhane Military Medical Academy; ^2^Division of Biostatistics, Gulhane Military Medical Academy; ^3^Department of Family Medicine, Gulhane Military Medical Academy; ^4^Department of Pediatrics, Gulhane Military Medical Academy School of Nursing; ^5^Department of Pediatric Nursery, Gulhane Military Medical Academy; ^6^Department of Otorhinolaryngology-Head and Neck Surgery, Gulhane Military Medical Academy

**Objectives**: Obesity frequency has been increased in the world especially in children. It is suggested that obesity influences sleep quality. Sleep disorder has negative impact on children health. In this study it is aimed to determine association between obesity and sleep disorder.

**Methods**: Reliability and validity confirmed Turkish version of Pediatric Sleep Questionnaire (PSQ) was conducted 132 children (65 females, 67 males), diagnosed obesity by Body Mass Index, aged 9-14 in Gulhane Military Medical Faculty.

**Results**: Concerning the demographics, both groups consist of healthy and obese children at different ages (p=0.03). Additionally, each group has also evaluated considering gender difference separately in the following tests (p=0.5). Sleeping scores determined for obese and healthy children are 21.51 ± 10.62 and 15.53 ± 8.97 respectively. The sleep scores showed statistical difference between each group. As a result, that is reached that sleep quality for obese children is higher than control groups. Besides, that is resulted that gender difference has no effects on this study.

**Conclusion**: Sleep has a vital impact on healthy life and school performance. Although there is no difference between healthy and obese group for sleep disorder, it is believed that obesity has and impact on sleep quality. Chronic conditions such as obesity should be investigated as objective criteria to elaborate sleep disorder problems.

## P2-2-22 Type 2 Diabetes Mellitus Treatment is Controversial in Alstrom Syndrome with Hepatic Dysfunction; Metformin or Ìnsulin? A case report

### Ediz Yesilkaya^1^, Cengizhan Acikel^2^, Onur Akin^1^, Gokhan Ozge^1^, Erkan Sari^1^, Sinan Sari^3^, Bulent Unay^1^

#### ^1^Division of Endocrinology and Metabolism / Department of Pediatrics, Gulhane Military Medical Academy; ^2^Division of Biostatistics, Gulhane Military Medical Academy; ^3^Division of Endocrinology and Metabolism/Department of Pediatrics, Gazi University School of Medicine

## P2-2-23 Case report: Transient neonatal diabetes in a 31 weeks old Thai premature baby

### Krittha Jeerawongpanich^1^, Elisa De Franco^2^

#### ^1^Department of Pediatrics, Faculty of Medicine, Burapha University, Burapha University; ^2^University of Exeter Medical School, Naomi Berrie Fellow in Diabetes Research Molecular genetics

**Background**

Neonatal Diabetes Mellitus (NDM) is a rare condition occurring in 1:300,000 -1:400,000 live births. All patients are diagnosed with diabetes in the first 6 months of life. Two main groups have been recognized: transient NDM (TNDM) and permanent NDM (PNDM). TNDM usually remits within a few weeks or months.

All patients were diagnosed with diabetes under 6 months should undergo genetic testing for monogenic NDM.

**Case report**

He was born prematurely. His gestational age was 31+ weeks on October 4th, 2015. His birth weight was 1,540 grams. He is the first child of his family. His blood glucose has been high since birth. He received insulin therapy infusion for 32 days, then he was given insulin subcutaneously once daily. Insulin therapy lasted for a total of 60 days.

The physical examination showed that he had macroglossia and umbilical hernia.

Methylation specific analysis showed that he has a loss of maternal methylation at chromosome 6q24, confirming a diagnosis of transient neonatal diabetes (TND). TND is a rare subtype of diabetes, affecting only ~1 in 400,000 babies.

**Progression**

Now he is 13 months old (corrected age 11 months). His weight is 10.5 kg (P50-P75), height is 76 cm (P50-P75). Currently, he does not have macroglossia and umbilical hernia. His growth and development is normal. His blood glucose is 68-95 mg/dL. His last HbA1C (10 Nov 2016) is 4.9%.

**Conclusion**

70% of TND have abnormalities at the 6q24 locus which disturb expression of the imprinted genes PLAGL1 and HYMAI. Diabetes relapses in at least 50–60% of patients, usually around puberty. Prolonged follow-up is imperative.

**Consent for publication:** The authors declare that the written informed consent was obtained for publication.

**Fig. 1 (abstract P2-2-23).**
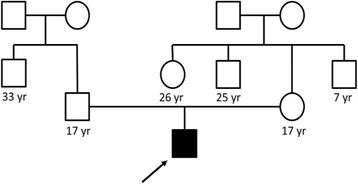
Pedigree of this patient

**Fig. 2 (abstract P2-2-23).**
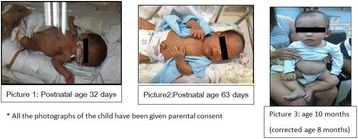
See text for description

**Fig. 3 (abstract P2-2-23).**
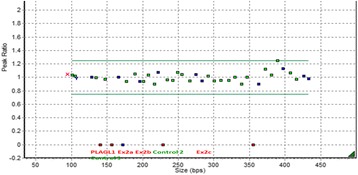
MS MLPA result showing loss of methylation for the PLAG1 Probes

## P2-3-1 Clinical features in Turner syndrome and correlation with genotypes

### Yee Lin Lee, Loo Ling Wu

#### Department of Pediatrics, Universiti Putra Malaysia

**Background**: The incidence of Turner syndrome has been reported as 1:2500 female livebirths. It’s diagnosis requires the presence of characteristic features in phenotypic females with complete or partial absence of the second sex chromosome, with or without cell line mosaicism. Recognising these phenotypic characteristics will aid in early diagnosis and improvement in management. We aimed to describe the phenotypic characteristics of Turner syndrome patients and assess the correlation of genotype with phenotype and clinical outcome.

**Methods**: This is a cross sectional study over 1 year period whereby all female patients with karyotype proven Turner syndrome who are on paediatric endocrine clinic follow up in Universiti Kebangsaan Malaysia Medical Centre from year 1995 till year 2016 were examined for physical features of Turner syndrome. Other physiologic features such as cardiac, ENT and ophthalmologic abnormalities were obtained from medical records. Patients were classified based upon karyotype into females with monosomy 45,X (Group A) and females with other X chromosomal abnormalities ie X mosaicism, Y mosaicism, ring X, isochromosome Xq and Xp (Group B). Clinical features of the two groups were analysed.

**Results**: Of the 34 patients included in the study, 16 (47.1%) were diagnosed with monosomy 45,X and the rest with other X chromosomal abnormalities. Short stature was universal in Group A versus 94.4% in group B. Short, webbed neck and edema of hands/feet were significantly more common in Group A than Group B ( 43.8-93.8% vs 16.7-38.9%). Cubitus valgus, short 4th and 5th metacarpals/metatarsals and nail dysplasia were common in both groups occurring in 83.3-94.4% of patients. All patients in Group A had no spontaneous puberty compared with 71.4% in Group B. Cardiac and ear abnormalities were more common in Group A than Group B (50-56.3% vs 11-27.8%) . Hypertension, hypothyroidism, impaired carbohydrate metabolism and learning disabilities were however more common in Group B than Group A ( 11.1-33.3% vs 0-6.3%).

**Conclusion**: Phenotypic characteristics may be subtle in Turner patients with other X chromosomal abnormalities, suggesting the importance of karyotyping as part of the work up for short stature in girls. This study also suggests that chromosomal variations may affect the phenotype and clinical outcome of Turner patients. This alludes to the importance of good cell numbers in karyotyping and the use of FISH study to enable detection of low level mosaicism which can carry clinical significance to the patient’s presentation.

## P2-3-2 Endocrine care in paediatrics Central Nervous System Germ Cell Tumor (CNSGCT)

### Wing Yan Jennifer Tsang, Wing Kin Gary Wong, Ho Chung Yau, Lai Ka Samantha Lee, Ming Kong Matthew Shing

#### Department of Paediatrics, The Chinese University of Hong Kong, Prince of Wales Hospital

**Background**: CNSGCTs account for over 10% of primary paediatric brain tumours in Asian countries. CNSGCTs are characterized with different spectrums of endocrine problems as they are commonly located near the hypothalamic-pituitary axis. In this study, we aim to review the endocrine manifestations of the local paediatrics CNSGCT tumors at presentation and after treatment.

**Methods**: We carried a retrospective review for paediatrics CNSGCT patients who were diagnosed at less or equal to 18 years and received treatment protocols in Prince of Wales Hospital Children’s Cancer Centre during the 18-year period January 1996-June 2014. Exclusion criteria included congenital germ cell tumour and oncological treatment or endocrinological surveillance provided in other hospitals.

**Results**: Total 28 patients were included in our study. 18 (64%) were male and 10 (36%) were female. Mean age was 12.5 ± 3.8 years old. The mean follow up period was 7.1 years. 19 patients (68%) had pure germinoma and 9 patients (32%) had non-germinomatous germinoma. The most common location of CNSGCT was suprasella region (46%) including 4 with bifocal tumours and 1 patient with multifocal disease. All of the patients included had received any kinds of surgery including biopsy or subsequent gross tumour resection. 82% patients had received chemotherapy, 93% had radiotherapy. Thirteen patients (41%) presented with either endocrine manifestations alone or together with neurological symptoms. Twelve of these patients have their tumours located either along hypothalamic-pituitary axis or at suprasella region. The endocrine manifestation and tumour location correlated with statistical significance (p=0.045 ). Pathology and size of germ cell tumour did not correlate with the types of presentations. Among the hypopituitarism manifestation, diabetes insipidus was most commonly presented (39%) before diagnosis of CNSGCTs. 46% of the patients had pan-hypopituitarism defining as 3 or more pituitary hormonal dysfunctions on follow up. The body height SD showed statistical significant drop from mean SD -0.339 on presentation to SD -1.13 at the final visit after completion of treatment ( p=0.004 ). Total 4 patients (14%) and 7 patients (25%) belonged to either overweight or obese groups on diagnosis and at the last follow up respectively. The change of BMI z-score from presentation to the final follow up did not show statistical significance (p=0.059).

**Conclusions**: Endocrine manifestations and complications are common in paediatrics CNSGCT. By early involvement of paediatrics endocrine subspecialty and a systematic endocrine care approach after the diagnosis and treatment of CNSGCT can provide patients a more holistic care.

## P2-3-3 Pre-operative cortisol and thyroid hormone levels and their clinical association with craniopharyngiomas and other sellar-parasellar masses in children: a five-year review

### James Q. Chin^1,2^, Sylvia C. Estrada^1^

#### ^1^University of the Philippines- Philippine General Hospital, Section of Pediatric Endocrinology and Metabolism; ^2^Department of Pediatrics, Vicente Sotto Memorial Medical Center

**Background**: Pediatric sellar and parasellar tumors include a diverse group of tumors that form a separate entity, as far as incidence, histology and responsiveness to therapy is concerned. These tumors are not uncommon, and mainly present with neuro-ophthalmic signs and symptoms. Endocrinologic dysfunction is a common abnormality seen in these patients, even in the pre-operative period, the most common of which are secondary/ central hypothyroidism and/ or hypocortisolism.

**Objective**: The study aims to determine the prevalence of central/ secondary hypothyroidism and hypocortisolism among pediatric patients diagnosed with a sellar-parasellar mass, and to describe their clinical and radiologic characteristics and to determine the presence of any association.

**Study Design**: This is a 5-year retrospective chart review.

**Material and Methods**: Fifty-six children and adolescents, 1.07-18.49 years old, median 11.34, treated in our centre in the years 2009-2014, with a diagnosis of a “sellar-parasellar” mass, were included in the study. In the analysis, clinical symptoms at the time of diagnosis, as well as, data from medical histories, physical examination, auxological data, biochemical, hormonal and radiologic parameters were analyzed.

**Results**: Among 56 patients, central/ secondary hypothyroidism was noted in 55%, while hypocortisolism was seen in 39%. Hypocortisolism in patients with sellar-parasellar mass was positively associated with the male sex (OR 5.7) and with a tissue diagnosis of craniopharyngioma (OR 6.5). Hypothyroidism was also positively associated with craniopharyngioma (OR 10.71). Further, the presence of hydrocephalus also had an association with the development of both hypothyroidism ( OR 2.33 ) and hypocortisolism ( OR 4.2).

**Conclusion/ Recommendations**: Sellar-parasellar masses are associated with significant co-existing endocrinopathies. Close attention to the history, physical examination, auxological, biochemical and radiologic parameters would facilitate early appropriate intervention and support.

## P2-3-4 Craniopharyngiomas in korean children and adolescents: systematic analysis of 49 cases with long-term follow-up in one center

### Eun Kyung Cho^1^, Aram Yang^1^, Jinsup Kim^1^, Sohn Young Bae^2^, Su Jin Kim^3^, Sung Won Park^4^, Sung Yoon Cho^1^, Dong-Kyu Jin^1^

#### ^1^Department of Pediatrics, Samsung Medical Center; ^2^Department of Medcal Genetics, Ajou University School of Medicine; ^3^Department of Pediatrics, Jeil Hospital Dankook University Collage of Medicine; ^4^Department of Pediatrics, Myungji Hospital, Seonam University Collage of Medicine

**Objective**: Craniopharyngioma (CP) is most common intracranial tumor that located in hypothalamic and pituitary region, both of which are important in endocrine function. Tumor itself and their treatment can lead to significant long-term pituitary dysfunction impaired quality of life due to their proximity to vital structures. We describe the experience of single institute on the endocrinological issues of CP in children and adolescent for 20 years.

**Methods**: The medical records of pediatric patients who got a neurosurgical operation and diagnosed with CP in single institute between 1994 and 2014 were identified. 49 patients diagnosed with CP in that period, and their medical records of endocrinological issues are analyzed retrospectively.

**Results**: Among 49 patients, the female-to-male ratio was 24:25, and the mean age of patients was 8.9 years (range 5 months - 18 years). Based on the results of cocktail test after operation, thyroid stimulating hormone (TSH) deficiency, adrenocorticotropic hormone (ACTH) deficiency, growth hormone deficiency and gonadotropin deficiency were 35 (71%), 35 (71%), 42 (85%), and 12 (24%) respectively. However, most patients practically took adrenocorticosteroid (n=47, 95%), levothyroxine (n=44, 89%), and desmopressin (n=47, 95%). 29 and 24 patients were administrated growth hormone and sex hormone respectively. The mean height, weight and BMI SDS were increased from -0.8, 0.1 and 1.1 at diagnosis to 0.6, 1.6 and 1.9 at 5 years after operation. 6 and 3 patients were dyslipidemia and type II diabetes mellitus.

**Conclusion**: Pediatric patient with CP has many endocrinological problems at diagnosis and most of problems need to take medication for long time. And they are getting obese and at risk of metabolic syndrome, dyslipidemia and type II diabetes mellitus. A better understanding and frequent surveillance of the determinants of obesity would provide preventive interventions for improving health outcomes in adulthood.

## P2-3-5 Two brothers with congenital combined pituitary hormone deficiency

### Sayaka Yamamoto^1^, Yasuhiro Ueda^1^, Daisuke Sasaki^2^, Hayato Aoyagi^1^, Toshihiro Tajima^3^

#### ^1^Department of Pediatrics, Obihiro Kyokai General Hospital; ^2^Department of Pediatrics, Hokkaido University Hospital; ^3^Department of Pediatrics, Jichi Children's Medical Center Tochigi

**Introduction**: Combined pituitary hormone deficiency (CPHD) is a condition that causes a shortage of several hormones produced by the pituitary grand. Since some neonates with severe congenital CPHD show severe hypoglycemia and respiratory failure, early recognition and prompt treatment is required. In addition, some neonates with CPHD develop cholestasis. Here, we present two brothers with CPHD.

**A report of cases**: The elder brother showed growth failure at 1 year of age. Endocrine examination showed GH and TSH deficiency. The brain MRI showed small anterior pituitary. He was diagnosed with CPHD and GH and L-T4 supplementation was started. The younger brother presented with respiratory distress and shock shortly after birth.Mechanical ventilation and catecholamine therapy were required. As he showed micropenis and cryptorchidism and family history, the diagnosis of CPHD was suspected. At 1 day of age, hydrocortisone replacement was initiated, and thereafter his circulatory dynamics improved. Endocrine studies confirmed that he had ACTH, GH, TSH, PRL and gonadotropins deficiency. The brain MRI exhibited hypoplasia of anterior pituitary and ectopic posterior pituitary. The patient developed cholestasis and sludge in the common bile duct and the gallbladder at 18 days of age. Recombinant human GH therapy was started at 19 days of age. After GH supplementation, cholestasis was gradually improved and sludge disappeared at 3 months of age. Now, he is 9 months old. His height is 69.4 cm (-0.9SD) and body weight is 8470g (-0.4SD). His head control was obtained at 4 months of age, but he could not sit on his own. His motor development is slightly delayed.

**Conclusion**: We report siblings with congenital CPHD. This report indicates that shock is rare, but one of clinical features of severe CPHD. In addition, development of biliary sludge should be carefully checked in severe CPHD. The etiology of CPHD in siblings is now investigating.

**Consent for publication:** The authors declare that written informed consent was obtained for publication.

## P2-3-6 10p deletion in DiGeorge Syndrome Phenotype and Hypoparathyroidism: A case report

### Ediz Yesilkaya^1^, Cengizhan Acikel^2^, Onur Akin^2^, Hatice Akar^3^, Erkan Sari^1^, Cengiz Zeybek^4^, Salih Kozan^3^, Bulent Unay^1^

#### ^1^Division of Endocrinology and Metabolism / Department of Pediatrics, Gulhane Military Medical Academy; ^2^Division of Biostatistics, Gulhane Military Medical Academy; ^3^Department of Medical Genetics, Gulhane Military Medical Academy; ^4^Division of Nephrology / Department of Pediatrics, Gulhane Military Medical Academy

## P2-3-7 Endocrine Function in Children with Neurohypophysial Germinoma

### Megumi Hatano^1,2^, Toshihiko Takiura^2^, Atsushi Ogawa^2^, Tetsushi Ogawa^2^, Junko Ito^2^

#### ^1^Department of Molecular Endocrinology and Metabolism, Tokyo Medical and Dental University, Graduate School of Medical and Dental Sciences; ^2^Department of Pediatrics, Toranomon Hospital

**Background**: Germ cell tumors accounts for about 20% of pediatric brain tumors. Neurohypophysial germinomas are associated with a high incidence of hormonal and metabolic abnormalities attribute to the hypothalamus and pituitary dysfunction. Few detailed assessment of endocrine functions in children with neurohypophysial germinoma have been reported.

**Objective**: This study investigated the pre and post treatment endocrine status of 13 children with neurohypophysial germinomas.

**Methods**: According to a retrospective review of medical records, we assessed the profile, clinical course and endocrinological function of the patients. Thirteen children (7 males and 6 females, median age was 11y) were diagnosed with a histological confirmation at Toranomon hospital during 2002 to 2012.

**Results**: All patients were diagnosed neurohypophysial germinoma, with a histological confirmation, although HCG or AFP levels were high only in two patients. The treatment regimens of the patients included 13 chemo-radiation therapy, one underwent HSCT (autologous sources). Three patients have received radiation therapy more than 30 Gy. One patient had local recurrence 18 months after initial treatment. Polyuria was the most frequent initial symptom and all of the 13 patients had impaired ADH secretion. More than one anterior pituitary hormones were impaired at diagnosis in all patients. Impaired GH, TSH, ACTH and ADH secretion persisted after treatment. There was some improvement in gonadotropin levels in 5 of 11 patients.

**Conclusion**: It is important to evaluate endocrine functions and carefully follow up growth and pubertal development in children with neurohypophysial germinomas.

## P2-3-8 Pituitary Cushing's syndrome in children: a case report

### Pathikan Dissaneevate, Tipaporn Thongmak, Thitiporn Borkird

#### Department of Pediatrics, Hat Yai Hospital

Pituitary Cushing’s syndrome or Cushing’s disease in childhood and adolescence is very rare. Hormonal assay and special tests should be done for diagnosis. This report showed a 12-year old girl presenting with Cushing appearance. She had a history of 16 kilograms weight gain within 6 months. Her mother noticed that she looked round face, and increased dark skin around her neck. She had no history of exogenous glucocorticoid used. On examination, BP was 140/100 mmHg. She had moon face, buffalo hump and acne. Her height and weight were 154 cm (P50-75) and 51 kg (P90), respectively. Weight for height was 116%. Laboratory tests showed loss of diurnal variation of serum cortisol, high serum ACTH, can’t be suppressed by low dose dexamethasone suppression test but can be suppressed by high dose dexamethasone suppression test. Pituitary MRI showed an inhomogeneous enhancing sellar mass, 1x1 cm in size. It was compatible with pituitary Cushing’ s disease. Tumor removal was done by transsphenoidal surgery. Pathology showed pituitary tumor, corticotroph adenoma. Six months after operation, tumor recurrence was found and hypophysectomy was done again. Post-operative complications were found such as diabetes insipidus, central hypothyroidism, adrenal insufficiency and were treated by hormonal replacement. She has normal growth parameters and needs continuous long term follow-up.

**Consent for publication:** The authors declare that written informed consent was obtained for publication.

## P2-3-9 Hypopituitarism and Septo-optic Dysplasia: A case presentantion

### Eirini Dikaiakou^1^, Elpis-Athina Vlachopapadopoulou^1^, Eirini Kaloumenou^1^, Vyron Markousis^1^, Maria Kafetzi^2^, Aspasia Fotinou^2^, Stefanos Michalacos^1^

#### ^1^Children's Hospital P. & A. Kyriakou, Dept. of Endocrinology-Growth and Development; ^2^Children's Hospital P. & A. Kyriakou, Biochemistry Dept.-Hormones Laboratory

**Introduction**: Septo-optic dysplasia (SOD), known as de Morsier syndrome, is a rare congenital malformation syndrome, which is characterized by underdevelopment of the optic nerve, pituitary gland dysfunction, and absence of the septum pellucidum (a midline part of the brain). The clinical diagnosis of SOD is made when two or more characteristics of the classic triad are present. The degree of pituitary deficiency and visual impairment can vary in the severity of clinical presentation and phenotype. The brain effects are variable as well. SOD has been associated with young maternal age, while mutations in HESX1, OTX2, SOX2 and PAX6 have been implicated in cases of SOD and congenital hypopituitarism. However, in most cases SOD is a sporadic birth defect of unknown cause and does not recur with subsequent pregnancies.

**Case presentation**: A 6 year old girl was referred to our Pediatric Endocrinology Department for investigation of short stature, impaired growth velocity, polyuria and polydipsia as well as visual disorders, such as nystagmus and decreased visual acuity. On physical examination, her height was 104 cm (< 3rd percentile) and her weight was 19 kg (50th percentile). She had no dysmorphic features. She had bilateral nystagmus. The rest of the PE was normal and she was prepubertal. Investigation of the pituitary function revealed growth hormone deficiency and partial diabetes insipidus. MRI of the brain and pituitary revealed an intact septum pellucidum with bilateral optic nerve hypoplasia, hypoplasia of the sella turcica and thinning of the pituitary gland, suggestive of SOD. Treatment with recombinant human GH and desmopressin was initiated. During follow-up, central hypothyroidism was recognized and L-thyroxine substitution therapy was initiated. She was followed regularly, having a very good response in therapy with growth acceleration and normalization of height SDS. She entered puberty spontaneously. Molecular genetic analysis is anticipated.

**Conclusion**: The presence of optic nerve hypoplasia should raise the suspicion of hypopituitarism, in terms of SOD. Moreover, as the degree of visual impairment and pituitary dysfunction are highly variable, a high level of suspicion regarding the associated endocrinopathies and prompt investigation is very important.

**Consent for publication:** The authors declare that written informed consent was obtained for publication.

## P2-3-10 Preliminary Study on the Dynamic Changes of Anti-mullerian Hormone and Inhibin B of Prepubertal Acute Lymphoblastic Leukemia Girls at the Stage of Initial Chemotherapy

### Linqi Chen, Dongling Gu, Xiuli Chen, Haiying Wu, Fenyun Wang, Rongrong Xie, Ting Chen

#### Division of Endocrinology and Metabolism / Department of Pediatrics, Children's Hospital of SooChow University

**Objective**: The objective is to know the dynamic changes of AMH and INHB of prepubertal ALL girls at the stage of initial chemotherapy and to discuss the influence of CCCG-ALL-2015 on ovarian function.

**Methods**: We chooseed 21 girls who received chemotherapy in the department of hematology in our hospital for the first onset standard-risk acute lymphoblastic leukemia.They were prepubertal girls.They all received the chemotherapy regimens. During the course of chemotherapy, determined the levels of AMH and INHB at the stages of pre-chemotherapy, after three months and six months of chemotherapy by ELISA. Then we compared the dynamic changes of AMH with the paired samples T test. We compared the dynamic changes of INHB with the Wilcoxon Matched-Pairs Signed-Ranks Test.

**Results**: We tested the AMH and INHB level in all these samples. The paired samples T test was adopted to evaluate the level of AMH. The results showed that the level of AMH after three months of chemotherapy were statistically significant lower than prechemotherapy ip=0.023<0.05 ). The level of AMH after six months of chemotherapy were statistically significant lower than pre-chemotherapy ip=0.045<0.05 ). The AMH level was no significant difference between after three months group and six months group of chemotherapy (p=0.39>0.05). The level of INHB of 12 patients were beyond the detection limit before and after Chemotherapy.we compared the changes of INHB level between pre-chemotherapy and chemotherapy for 3 months; the changes of INHB level between pre-therapy and chemotherapy for 6 months, The statistics show there is no statistical difference between any two groups.

**Conclusion**: At the initial Stage of CCCG-ALL-2015, the AMH of the girls show significant decrease and the ovarian function is disturbed. For prepubertal ALL girls who receive chemotherapy, INHB may not be suitable to choose as an indicator to evaluate the effect of the initial chemotherapy to ovarian function.

## P2-3-11 Hypothalamic hypeoadrenocorticism associated with Rathke's cleft cyst: A case of an 18-year-old woman and a review of the literature

### Fumika Kawano, Tomoyo Itonaga, Masanori Inoue, Miwako Maeda, Hiroaki Miyahara, Kenji Ihara

#### Department of Pediatrics, Oita University Faculty of Medicine

**Purpose**: Rathke’s cleft cysts (RCCs) are nonneoplastic, sellar or suprasellar epithelium-lined cysts originated from Rathke's pouch in the pituitary gland. Patients with RCCs are usually asymptomatic, but some were identified when turned symptomatic in the middle age. The charecteristics of the patients during childhood or adolescence remains unknown.

**Methods**: We describe an 18-year-old girl who sometimes suffered from malicious fatigue in the morning since her early teens and brain MRI exhibited T1 hyperintense/T2 hypointense signals between the anterior and posterior pituitary glands indicating the presence of RCC. Based on the authentic endocrinological evaluation, her adrenal function was supposed to be normal, whereas serum cortisol level was strangely dropped around noon suggesting the presence of hypothalamic adrenal dysfunction. Next, we reviewed and summarized the 8 cases reported from 2002 to 2014.

**Results**: There was no case with isolated adrenal dysfunction, and 4 of 8 cases (50%) were associated with hypogonadism, 2 of 8 (25%) with growth hormone deficiency, 2 of 8 (25%) with central diabetes insipidus, and 1 case (12.5%) with hypothyroidism.

**Conclusions**: The present girl and the previously reported cases suggested that dysfunction of the hypothalamic pituitary system might progress slowly from their teens, resulting in the multisystem dysfunction of hypothalamus and pituitary gland in their later life. We speculate that hypothalamic hypoadrenalism may be subsumed in the adolescent girls with prolonged and indefinite complaints. Brain MRI and endocrinological evaluation including daily blood cortisol profiling would be the key to get to the diagnosis of impaired central adrenal function by RCC.

**Consent for publication:** The authors declare that the written informed consent was obtained for publication.

## P2-3-12 Clinical Analysis of 24 Cases of Rathke's Cleft Cysts in Children

### You-Jun Jiang, Ke Huang, Chao-Chun Zou, Li Zhang, Chan Lai

#### Division of Endocrinology, Children's Hospital of Zhejiang University School of Medicine

**Purpose**: To evaluate the clinical features of Rathke cleft cysts (RCCs) in children.

**Methods**: A retrospective analysis was conducted in 24 patients with RCCs diagnosed by magnetic resonance(MR) between July 2010 and August 2015. Their clinical, hormonal, and imaging findings were reviewed.

**Results**: 24 patients got 1~6 years follow-up. 6 cases (25.0%) were diagnosed precocious puberty and half of them were central precocious puberty. 3 cases (12.5%) were obesity. 7 patients (29.2%) had idiopathic short stature, 8 (37.5%) had growth hormone deficiency (GHD). In all GHD cases, 1 patient combined with central diabetes insipdus, another onecombined with central hypothyroidism. Till February 2016, all of the patients were re-examined pituitary MR. Rathke cysts in 3 patients were disappeared. 2 patients got transsphenoidal pituitary cyst excision and theirs Rathke cysts were not recurrenced after 3-4 years follow-up.

**Conclusion**: Pituitary dysfunction is most common in pediatric RCCs patients. Most of Rathke’s cleft cysts pediatric patients don't need surgery therapy, some cases are self-healing.

## P2-3-13 Maternal night-fasting interval during pregnancy is associated with offspring early childhood blood pressures

### Poh Hui Wee^1^, See Ling Loy^2,3^, Jerry Yen Chan Kok^2,3^, Marjorelee T. Colega^4^, Yin Bun Cheung^5,6^, Keith M. Godfrey^7,8^, Peter D. Gluckman^4,9^, Kenneth Kwek^12^, Seang Mei Saw^10^, Yap-Seng Chong^4,11^, Falk Müller-Riemenschneider^10^, Ngee Lek^1,3^, Yung Seng Lee^4,14^, Mary Foong-Fong Chong^4,13,14^, Fabian Yap^1,3,15^

#### ^1^Department of Pediatrics, KK Women's and Children's Hospital; ^2^Department of Reproductive Medicine, KK Women's and Children's Hospital; ^3^Duke-NUS Medical School; ^4^Singapore Institute for Clinical Sciences, Agency for Science, Technology and Research (A*STAR); ^5^Duke-NUS Medical School, Center for Quantitative Medicine; ^6^University of Tampere and Tampere University Hospital, Tampere Center for Child Health Research; ^7^University of Southampton, Medical Research Council Lifecourse Epidemiology Unit; ^8^University of Southampton and University Hospital Southampton National Health Service; Foundation Trust, National Institute for Health Research Southampton Biomedical Research Centre; ^9^University of Auckland, Liggins Institute; ^10^Saw Swee Hock School of Public Health, National University of Singapore; ^11^Department of Obstetrics & Gynaecology, Yong Loo Lin School of Medicine, National University of Singapore; ^12^Department of Maternal Fetal Medicine, KK Women's and Children's Hospital; ^13^Singapore Institute for Clinical Sciences (SICS), Agency for Science, Technology and Research (A*STAR), Clinical Nutrition Research Centre; ^14^Yong Loo Lin School of Medicine, National University of Singapore and National University Health System, Department of Paediatrics; ^15^Lee Kong Chian School of Medicine, Nanyang Technological University

**Background**: Little is known about the potential programming effect of maternal chrononutrition during pregnancy on offspring's cardiovascular outcomes. We examined the association between day-time and night-time fasting intervals during pregnancy with offspring early childhood blood pressures.

**Methods**: Mother-offspring pairs (n=247) from a prospective cohort in Singapore were studied. At 26-28 weeks’ gestation, maternal longest day-time (0700 to 1859h) and night-time (1900 to 0659h) fasting intervals were determined from 3-day food diaries. Offspring systolic blood pressure (SBP) and diastolic blood pressure (DBP) were measured at 3-year-old. Offspring SBP and DBP were categorised into normal (<90th percentile) or risk of hypertension ( ≥ 90th percentile) by sex and height. Multiple logistic regressions with confounders adjustment were used to examine the association between maternal fasting intervals and offspring blood pressures.

**Results**: Means (standard deviations, SD) maternal fasting intervals were 4.7 (1.1) hours during the day-time and 10.0 (1.3) hours during the night-time. Means (SD) offspring SBP and DBP were 96.4 (8.5) mmHg and 57.3 (5.0) mmHg respectively. Each hourly increase in maternal night-time fasting interval was associated with decreased risk of hypertension in offspring (SBP: adjusted OR=0.57, 95% CI 0.33, *p* =0.043) and DBP (DBP: adjusted OR=0.45, 95% CI 0.26, 0.79, *p* =0.005). The association between day-time fasting interval and offspring blood pressures was not significant.

**Conclusion**: Longer maternal night-fasting intervals during pregnancy are associated with lower offspring risk of hypertension at 3 years of age. Studies are needed to further evaluate the effects of maternal fasting intervals during pregnancy and long-term cardiovascular health in the offspring.

**Table 1 (abstract P2-3-13).**
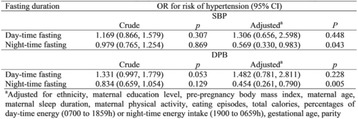
The association of maternal pregnancy fasting duration with offspring’s risk of hypertension

## P2-3-14 A Case of Atypical Pubertal Development Caused by Dopamine Antagonist

### Yuichi Miyakawa, Akito Sutani, Atsumi Tsuji, Tomohiro Morio, Kenichi Kashimada

#### Department of Pediatrics and Developmental Biology, Tokyo Medical and Dental University (TMDU)

**Background**: Symptomatic hyperprolactinemia is a rare condition in children, and may affect pubertal development by inhibiting hypothalamic-pituitary-gonadal (HPG) axis. The main causes of hyperprolactinemia are pituitary tumors and drugs, such as dopamine antagonists. Recently, dopamine antagonists have been widely used for school-age children with psychotic disorders, arising concerns for increasing the number of children with drug induced hyperprolactinemia. However, few cases have been reported to develop hyperprolactinemia during pubertal period, and the details of clinical courses of pubertal development with hyperprolactinemia have not been documented.

**Object**: We report a case of drug induced hyperprolactinemia with atypical development of puberty, and clarify the details of clinical features of drug induced hyperprolactinamia during pubertal period.

**Case**: An eight-and-a-half-year-old girl was referred to our hospital because of developing pubic hair without other obvious pubertal signs. Although she had sparse curly hairs on the labia majora (Tanner III), other pubertal signs, such as breast development and growth spurt were not observed. Despite prepubertal levels of gonadotropins and estradiol (LH: 0.2 mIU/ml,'sH: 2.7 mIU/ml, E2: 5 pg/ml), the serum level of DHEA-S, 385 ng/ml, suggested that the patient developed adrenarche. By repeated assay for PRL, we identified hyperprolactinemia (PRL: 22.5~75.1 ng/ml) that would suppress LH and'sH secretion. She had been prescribed a selective antagonist at dopamine receptors, sulpiride, for a psychosomatic problem for six months, and we suspected that hyperprolactinemia was caused by sulpiride, resulting in atypical pubertal development by suppressing HPG axis. Indeed, one month after careful cessation of sulpiride, her HPG axis was recovered to pubertal level, LH: 1.9 mIU/ml,'sH: 4.8 mIU/ml with reduced serum level of PRL, 1 ng/ml. Subsequently, she developed other pubertal signs, including growth spurt (8 cm/year) and breast development.

**Discussion**: Generally, development of pubic hair depends on adrenarche, and is independent from other pubertal sings that are caused by estradiol belonging to HPG axis, and atypical pubertal development could be caused by isolated gonadotropin deficiency such as Prader-Willi syndrome. In this case, we presume that atypical pubertal development would be the characteristics of hyperprolactinemia during pubertal period because prolactin excess does not inhibit the adrenal androgen.

**Conclusion**: One of the adverse effects of dopamine antagonist, atypical pubertal progress, developing pubic hair without other obvious pubertal signs, should be considered.

**Consent for publication:** The authors declare that written informed consent was obtained for publication.

## P2-3-15 Endocrine-related symptoms and deficits can more frequently precede at diagnosis of suprasellar tumors

### Atsushi Suzuki, Tatsushi Tanaka, Kohei Aoyama, Haruo Mizuno

#### Department of Pediatrics and Neonatology, Nagoya City University Graduate School of Medical Science

**Background**: Suprasellar tumors account for 15%–20% of all pediatric brain tumors, neurologic or ophthalmic symptoms prompting most such diagnoses. However, endocrine-related symptoms such as growth retardation, obesity, polydipsia, and polyuria and endocrinological deficits such as growth hormone deficiency, central hypothyroidism, central hypoadrenalism and central diabetes insipidus frequently precede onset of neuro-ophthalmic symptoms. Most patients with suprasellar tumors have permanent residual endocrinological deficits after treatment of their tumors by surgical excision, chemotherapy, or radiation therapy and therefore require endocrine replacement therapy to maintain optimal health and normal growth. Nevertheless, endocrine-related symptoms are not always easily identifiable and accurate evaluation of these patients’ endocrinological status is difficult. Our objective was to clarify the relationship between presenting symptoms and results of accurate endocrinological evaluation before treatment of suprasellar tumors.

**Methods**: This was a single center retrospective study of data obtained from our hospital’s medical records on patients with suprasellar tumors treated by our institution.

**Results**: Data of 12 patients who had undergone treatment of suprasellar tumors (craniopharyngioma, *n*=7; germ cell tumor, *n*=4; xanthogranuloma, *n*=1) were assessed. The chief symptoms leading to tumor diagnosis were neurologic-ophthalmic (*n*=7, 58%) and endocrinological (*n*=3, 25%). In addition to these three patients, another seven (58%) had unrecognized endocrinological symptoms prior to diagnosis; thus, endocrinological symptoms preceded diagnosis in 10/12 study patients (83%). The main unidentified endocrinological symptoms were growth retardation and polydipsia. Six of seven patients (86%) who had undergone accurate endocrinological evaluation had endocrinological deficits before tumor treatment; namely, growth hormone deficiency (*n*=6, 86%), central hypoadrenalism (*n*=4, 33%), central diabetes insipidus (*n*=4, 33%), and central hypothyroidism (*n*=2, 29%).

**Discussion**: Most patients with suprasellar tumors reportedly have some endocrinological abnormalities long before diagnosis of their tumors, only a minority of these patients being aware of endocrine-related symptoms or having endocrinological deficits identified at diagnosis. Although a few studies included suprasellar tumors that we excluded (i.e., hamartoma, optic pathway glioma, suprasellar archanoid cyst, hypothalamic-pituitary astrocytoma) and are therefore not strictly comparable, endocrine-related symptoms and deficits were more frequently present in our cohort than has previously been reported.

**Conclusion**: Patients with suprasellar tumors more often have endocrinological abnormalities at diagnosis than previously reported.

## P2-3-16 A Case of Neonatal Central Diabetes Insipidus in a Premature Infant - Challenges in Diagnosis and Management

### Andrew Sng, Kah Yin Loke

#### Department of Paediatrics, National University Health System (NUHS)

**Background**: Neonatal central diabetes insipidus (CDI) is rare as compared to nephrogenic diabetes insipidus (NDI) and it has been associated with meningitis, central nervous system malformations, intraventricular haemorrhage and hypoxic ischaemic encephalopathy.

**Case Report**: A 25 week premature male neonate with a birth weight of 804 grams had a stormy perinatal clinical course complicated by severe respiratory distress syndrome and grade III bilateral intraventricular hemorrhage. On day 69 of life, he developed polyuria with a serum sodium level of 157 mmol/L, serum osmolality of 352 mmol/kg and a decreased urine osmolality of 181 mmol/kg, which was diagnostic of diabetes insipidus. Intravenous (IV) pitressin was commenced at 0.1 mU/kg/hr and titrated upwards so as to achieve clinical and biochemical effect (Table 1). He showed a gradual response over the next 72 hours with a decrease in serum sodium to 133 mmol/L and serum osmolality to 269 mmol/kg and an increase in urine osmolality to 291 mmol/kg. Subsequently, his serum sodium remained stable within the range of 140-148 mmol/kg while on a dose of IV pitressin of 1.4 mU/kg/hr, He was then converted to oral desmopressin, the dose of which was titrated to 3 micrograms/kg/dose 12 hourly with good effect.

**Discussion**: In neonates, NDI is more common than CDI. Hence it was important to differentiate the two, especially in our patient who was a premature baby neonate. To our knowledge, this is the first report of IV vasopressin in the diagnosis of CDI. Other studies have used intranasal desmopressin which has been associated with large swings in serum sodium. The advantage with the initial use of an IV vasopressin infusion lies in the titration of the dose in a controlled and safe manner to establish response, and allows a clear differentiation of CDI from NDI. The use of vasopressin in neonates is technically challenging with regard to both the 4,000 fold dilution and administration. However when done accurately, using IV vasopressin can allow for very close and safe titration of dose to response.

**Consent for publication:** The authors declare that written informed consent was obtained for publication.

**Table 1 (abstract P2-3-16).**
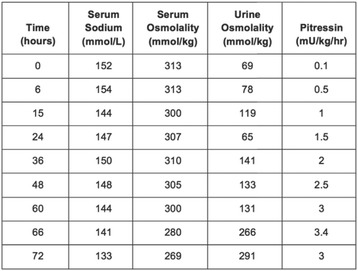
See text for description

## P2-3-17 Two cases of prolonged cholestasis caused by combined pituitary hormone deficiency in early infancy

### Masahiro Nakamura, Koji Muroya, Junko Hanakawa, Machiko Toki, Yumi Asakura, Masanori Adachi

#### Department of Endocrinology and Metabolism, Kanagawa Children's Medical Center

**Background**: Cholestasis in the neonatal and early infancy period is rare, but one of presenting features in patients with combined pituitary hormone deficiency (CPHD). Here, we report two cases where both the patients presented with prolonged cholestasis as a characteristic manifestation of CPHD.

**Case 1**: A 4-month-old boy who was born by cesarean section with birth weight of 1638 g (at 36 weeks’ gestation) with asphyxia presented failure to thrive. At 10 d of age, he discharged clay-colored stools. At 40 d of age, he was diagnosed with hypothyroidism (TSH 6.68 μ U/ mL, fT4 0.75 ng/dL) and started to take L-thyroxine. At 46 d of age, he showed liver dysfunction with cholestasis, accompanied by apparent hypoglycemia. Brain magnetic resonance imaging (MRI) at 4 months of age revealed hypoplastic anterior pituitary, attenuated pituitary stalk, and ectopic posterior lobe. GHRP-2 stimulation test showed extremely low GH, ACTH, and cortisol (F) responses (each peak level: 5.09 ng/mL, 7.9 pg/mL, 0.3 μg/dL, respectively). He was diagnosed with CPHD, and therefore GH and hydrocortisone replacement therapy was immediately initiated. The hormone replacement therapy (HRT) for 4 months normalized his liver function.

**Case 2**: A 3-month-old boy who was born at term by breech delivery with asphyxia, hypoglycemia, and microphallus presented persistent jaundice. At 26 d of age he was diagnosed with hypothyroidism (TSH 8.5 μ U/mL, fT4 0.5 ng/dL), and L-thyroxine replacement therapy was started. From 70 d of age, his liver function was deteriorating with an increased conjugated bilirubin concentration, and abdominal ultrasonography revealed some gallstones. Brain MRI indicated attenuated pituitary stalk and ectopic posterior lobe. GHRP-2 and LHRH stimulation tests demonstrated low responses of GH, F, LH, and'sH (each peak level: 10.06 ng/mL, 3.2 μg/dL, 0.87 mIU/mL, 0.57 mIU/mL, respectively). GH and hydrocortisone replacement was introduced subsequently. By the two months after starting the HRT, his liver function had been recovered.

**Discussion**: In both cases, the antecedent administration of L-thyroxine prior to that of GH and hydrocortisone could delay the patients’ recovery from cholestatic liver dysfunction, and could also lead to the extended time of failure to thrive. That is why early institution of proper HRT is necessary for CPHD patients.

**Conclusion**: Brain MRI and examination of multiple pituitary hormones should be properly conducted for infants who present such features of CPHD as prolonged cholestatic jaundice, apparent hypoglycemia, and microphallus in the males.

**Consent for publication:** The authors declare that written informed consent was obtained for publication.

## P2-3-18 Two Cases of Dopa-responsive Dystonia

### Liang Liyang, Jiang Zhuannan, Meng Zhe, Zhang Lina, Hou Lele, Liu Zulin, Lao Wenqin

#### Sun Yat-sen Memorial Hospital, Sun Yat-sen University

**Objective**: FUnderstand the research progress of dopa-responsive dystonia by reporting two cases of this rare disease.

**Methods**: FAnalysed the clinical manifestations Aclinical laboratory examination and gene sequencing of two cases of dopa-responsive dystonia patient and reviewed the related literatures.

**Results**: FClinical and Laboratory diagnosis two cases of dopa-responsive dystonia.

**Conclusion**: Fdopa-responsive dystonia is a rare disease Cits clinical characteristics include FAchildhood-onset dystonia with marked diurnal fluctuation and female predominance, for the not enough supplement of BH4 during the day Aa dramatic and sustained response to relatively low doses of levodopa, but Little effectiveness of treatment of BH4A mutations of GCH-I gene and TH gene A increased prolactin by regulation of dopamine D2 receptor clinical diagnosis of DRD remains difficult. The children who suspected the disease should be taken genetic test once diagnosed C the Comprehensive Treatment C low doses of levodopa therapy Cs hould be taken to improve the quality of life as soon as possible.

## P2-4-1 Determinants of height z-score and stunting at 2 years of age in term healthy children in Delhi

### Vandana Jain, Brijesh Kumar, Sapna Khatak, Babita Upadhyaya

#### Division of Endocrinology / Department of Pediatrics, All India Institute of Medical Sciences

**Objective**: To identify determinants of height for age z-score (HAZ) and stunting at 2 years of age

**Methodology**: This cohort study was conducted at AIIMS, New Delhi. Healthy term neonates were enrolled and followed till 2 years. Weight and length were measured at birth, 1 and 2 years using electronic weighing scale and infantometer. Caloric intake was assessed by 24 hrs recall at 1 and 2 yrs. Fasting blood samples for vitamin D (25OHD) and insulin were drawn for a subset. HAZ at 2 years was calculated by WHO Anthro, and its correlation checked with weight and length at birth and 1 yr, parents’ height and BMI, socio-economic status (SES), gestational diabetes (GDM), caloric intake, 25OHD and insulin at 1 and 2 years.

**Results**: We studied 186 children (110 boys). Median (IQR) of HAZ at 2 years was -0.87 (-1.49 to -0.07) with 17 (9.1%) children being stunted (HAZ<-2). Median 25OHD at 1 yr (*n*=93) was 4.0 (3.0-9.1) ng/ml, and at 2 yrs (*n*=78), 10.8 (6.5-17.5) ng/ml. Median (IQR) of Insulin at 1 yr (*n*=117) μU/ml was 3.1 (1.3-6.2) and at 2 yrs (*n*=101), 3.1 (1.8-4.6) μU/ml. Thirty-four (18.3%) were born to mothers with GDM. The mean ± SD of various parameters and their correlation with HAZ at 2 years are presented in Table 1. The correlation was strongest for weight and length at 1 yr. The odds ratios of stunting in the presence of various categorical variables are summarized in Table 1. Fathers’ height < 160 cm, and length at 1 yr < 71.5 cm had the highest odds for stunting at 2 yrs. Maternal BMI, GDM, SES, 25OHD and Insulin levels at 1 and 2 yrs were not correlated with HAZ at 2 years.

**Conclusion**: Height at 2 years was correlated with size at birth and 1 yr and caloric intakes at 1 and 2 years. Higher odds for stunting were seen if parents were short, length at birth or 1 yr was short, and weight and caloric intake at 1 yr were low. Serum 25OHD and Insulin levels were not correlated with HAZ.

**Table 1 (abstract P2-4-1).**
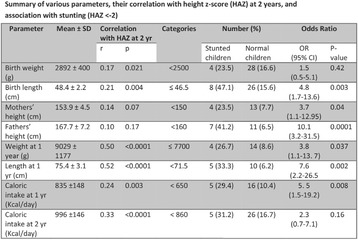
Summary of various parameters, their correlation with height z-score (HAZ) at 2 years, and association with stunting (HAZ<-2)

## P2-4-2 Long-term efficacy of recombinant human growth hormone therapy in short-statured patients with Noonan syndrome

### Eungu Kang^1^, Yoon-Myung Kim^1^, Insook Jeong^1^, Jin-Ho Choi^1^, Gu-Hwan Kim^2^, Beom Hee Lee^1,2^, Han-Wook Yoo^1,2^

#### ^1^Department of Pediatrics, Asan Medical Center Children's Hospital, University of Ulsan College of Medicine; ^2^Asan Medical Center Children's Hospital, University of Ulsan College of Medicine, Medical Genetic Center

**Purpose**: Noonan syndrome (NS) is characterized by short stature, heart anomalies, developmental delay, dysmorphic features, cryptorchidism, and coagulation defects. Several studies reported the short-term effects of recombinant human growth hormone (rhGH) treatment on the improvement of height. This study was performed to evaluate the long-term efficacy of rhGH in patients with NS in Korea.

**Methods**: This study included 15 prepubertal children with NS who received rhGH subcutaneously at a dose of 50–75 μ g/kg/day for 6 days a week for at least >3 years. Pre- and post-treatment data, such as height, weight, bone age, IGF-1, and IGFBP-3 levels, were collected every 6 months.

**Results**: Chronologic age and bone age at the start of treatment were 7.97 ± 1.81 and 5.09 ± 2.12 years, respectively. Height standard deviation score (SDS) was increased from -2.64 ± 0.64 to -1.54 ± 1.24 years after 3 years (P < 0.001). Serum IGF-1 SDS levels were elevated from -1.28 ± 1.03 to -0.10 ± 0.94 (*P* < 0.001). Height SDS was more increased in subjects without PTPN11 mutations compared to those with mutations after 3 years (P = 0.012). However, the other parameters, including bone age, IGF-1 SDS, and IGFBP-3 SDS, were not significantly different between patients with and without PTPN11 mutations.

**Conclusions**: Although this study included a relatively small number of patients, long-term rhGH therapy in NS patients was safe and effective at improving height, growth velocity, and serum IGF-1 levels, in accordance with previous studies. However, the meticulous monitoring of potential adverse events is still needed because of high dose of hGH and preexisting hyperactivity of RAS-MAPK pathway. Patients with PTPN11 mutations demonstrated a decreased response to rhGH therapy compared to those without mutations.

## P2-4-3 Impact of the great east Japan earthquake on the body mass index of milk-fed infants and toddlers: a nationwide infant survey

### Hiroshi Yokomichi^1^, Hiroko Matsubara^2^, Mami Ishikuro^3,4^, Masahiro Kikuya^3,4^, Tsuyoshi Isojima^5,6^, Susumu Yokoya^7^, Noriko Kato^8^, Toshiaki Tanaka^9^, Atsushi Ono10 ), Mitsuaki Hosoya10 ), Shoichi Chida^11^, Soichiro Tanaka^12^, Shinichi Kuriyama^2,3,4^, Shigeo Kure^12^, Zentaro Yamagata^1^

#### ^1^Department of Health Sciences, University of Yamanashi; ^2^Tohoku University, International Research Institute of Disaster Science; ^3^Tohoku University, Tohoku Medical Megabank Organization; ^4^Department of Molecular Epidemiology, Tohoku University; ^5^Department of Pediatrics, The University of Tokyo, Tokyo; ^6^Department of Pediatrics, Teikyo University School of Medicine; ^7^Department of Medical Subspecialties, National Center for Child Health and Development; ^8^Department of Early Childhood and Elementary Education, Jumonji University; ^9^Japanese Association for Human Auxology; ^10^Department of Pediatrics, Fukushima Medical University; ^11^Department of Pediatrics, Iwate Medical University; ^12^Department of Pediatrics, Tohoku University

**Objective**: A study has reported increased body mass index (BMI) of preschool children after the great east Japan earthquake (BMJ Open 2016:e010978). The results were limited to preschool children, and evidence of infant growth after the earthquake is necessary. The present study aimed to determine how the earthquake changed the growth of infants who were residing on the Pacific Ocean side of northeast Japan.

**Methods**: We collected health examination data of infants at 3, 6, 18 and 42 months of age in Fukushima, Miyagi and Iwate prefectures in Japan. For primary comparison, we defined three infant groups.

(1) ‘Milk-fed group’ included the infants who had experienced the earthquake between 3- and 6-month examinations. (2) ‘Toddler group’ included the infants who had experienced the earthquake at the age of 21–30 months. (3) ‘Unaffected group’ included the infants who had undergone the 42-month examination before the earthquake and therefore whose anthropometric data were not affected. In the strata of the prefectures and sexes, we graphically plotted the changes in BMI from 3 through 42 months of age, adjusting for gestational age, infant age in month and time points of health examinations in general linear model. Thereafter, we compared the changes in BMI between each affected vs. referent-unaffected groups.

**Results**: The analyses included 8,479 boys and 8,218 girls. In the milk-fed group at the age of 6 months, the girls in Fukushima, the boys (*p*<0.001) and girls in Miyagi and the boys and girls in Iwate represented slight to statistically significant decreases in change in BMI, compared to those in the unaffected group. In the milk-fed group in Fukushima, there were also statistically significant increases in the boys at 18 (*p*<0.01) and 42 months (*p*<0.001) and the girls at 42 months (p<0.01).

**Conclusions**: The data indicate that immediately aftermath of the earthquake, the growth of the affected suckling infants were slightly disturbed. The affected suckling infants in Fukushima also represent increased BMI at the age of 42 months and may have potential to show early adiposity rebound. The results highlight the need of medical follow-up to the infants in Fukushima.

**Fig. 1:**
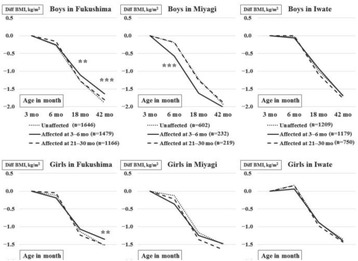
**(abstract P2-4-3).** See text for description

## P2-4-4 Metabolic and immunological features of young SGA children and the impact of 1-yr GH treatment

### Kanako Kojima-Ishii^1^, Naoko Toda^1^, Kazuhiro Ohkubo^1^, Kenji Ihara^2^, Shouichi Ohga^1^

#### ^1^Department of Pediatrics, Graduate School of Medical Science, Kyushu University; ^2^Department of Pediatrics, Faculty of Medicine, Oita University

**Background**: Children born small for gestational age (SGA) are at high risk of developing metabolic syndrome in adulthood. Growth hormone (GH) treatment for children born SGA has been reported to have favorable effects on lipid metabolism through the activating of signal transduction on metabolism, and probably, on immunity.

**Objective**: We aimed to elucidate the metabolic and immunological characteristics for SGA children and to assess their alteration by GH treatment.

**Subjects**: Twenty-six prepubertal short children (mean age 4.4 yr) born SGA were included for the study, and 26 healthy children (mean age 4.7 yr) had participated as controls.

**Methods**: All of 26 SGA patients were treated with GH. Anthropometry and measurement of hematologic, biochemical, immunological and endocrinological parameters were performed before and 1, 3, 6, 9 and 12 months after the start of GH treatment. At baseline, we compared these parameters between the SGA and control groups. We also examined the change of these parameters at each point of time from the baseline levels.

**Results**: After 12-month GH treatment, mean height SDS of SGA children changed from -3.29 SD to -2.73 SD. HbA1c level did not change significantly during the GH treatment. Total cholesterol, LDL-C and HDL-C levels were similar in both groups, and these parameters did not show significant changes during the GH treatment. SGA children had higher baseline levels of ApoA1 than controls, and the levels increased by 12-month GH treatment. The ApoB level and ApoB/ApoA1 ratio decreased by 12-month GH treatment. Among immunological parameters, monocyte counts were lower in patients than controls at base line, and increased by GH treatment. Neutrophil counts tended to be lower in patients than controls, and significantly rose after GH treatment. Flow cytometric analyses showed the NK cell ratio in peripheral leukocytes was higher in SGA patients, and decreased after GH treatment. Leptin and resistin levels were lower in patients than controls, which showed no significant change during GH treatment.

**Discussion**: Our study demonstrated the GH treatment changed apoprotein profiles toward improvement of lipid metabolism and prevention of cardiovascular disease for SGA children. The unique immunological profiles of lower neutrophils and monocytes and higher NK cell ratio in peripheral blood might affect the predisposition to metabolic syndrome for SGA children. GH treatment could significantly modify the immunological profiles.

**Conclusion**: GH treatment for SGA children potentially has favorable effects not only on the growth, but also on the lipid metabolism and immune system.

## P2-4-5 WHO 2006 Child Growth Standards overestimate short stature and underestimate overweight in Japanese children age 0-60 months

### Mikako Inokuchi^1,2^, Nobutake Matsuo^2,3,4^, John I Takayama^5^, Tomonobu Hasegawa^2^

#### ^1^Health Center, Keio University; ^2^Department of Pediatrics, Keio University School of Medicine; ^3^The Institute for Healthcare Quality Improvement, Tokyo Healthcare Foundation; ^4^National Center for Child Health and Development; ^5^Department of Pediatrics, University of California San Francisco

**Objective**: It is as yet unclear whether the WHO 2006 Child Growth Standards (WS) are applicable to East Asian populations in general. We studied the applicability of WS as standard may be appropriate in Japanese children.

**Methods**: We used the 2007-2013 Tokyo public child care center survey data (Tokyo 2007-2013 survey cohort: 3430 boys, 3025 girls, 0-60 months of age, semi-longitudinal data). For each child, z-scores for length/height and weight were calculated by comparison with both WS and the Japanese 2000 reference (JR). We adopted Cohen’s criteria (differences of 0.20 SD units, small; 0.50 SD, medium; 0.80 SD, large), in the assessment of differences in length/height and weight. The prevalence of children below and above certain thresholds for length/height and weight were obtained according to WS and JR in Japanese children (Tokyo 2007-2013 survey cohort): short stature, length/height < 3rd centile; tall stature, length/height > 97th centile; underweight, weight < 3rd centile; overweight, weight > 97th centile.

**Results**: Japanese children had significantly low mean z-scores for length/height: the mean z-score was close to -1.0 (medium-large difference for the most part) while mean z-scores for weight ranged from -0.01 to -0.62 compared with WS (small difference for the most part). (Table) Percentages of children classified as short stature, tall stature, underweight and overweight were as follows.

Short stature: 6.8-13.7% (WS), 0.3-8.0% (JR), in boys; 3.7-10.8% (WS), 0.6-7.2% (JR), in girls.

Tall stature: 0.0-1.6% (WS), 0.0-3.1% (JR), in boys; 0.0-1.8% (WS), 0.1-4.4% (JR), in girls.

Underweight: 2.4-6.8% (WS), 1.5-5.7% (JR), in boys; 0.8-6.9% (WS), 1.4-5.3% (JR), in girls.

Overweight: 0.0-1.0% (WS), 1.5-4.6% (JR), in boys; 0.0-1.6% (WS), 1.8-5.8% (JR), in girls.

**Discussion**: Compared with WS, our subjects were shorter and lighter, and substantially altered the prevalence of short stature, tall stature, underweight and overweight, overestimating short stature and underestimating overweight. These findings advocate that the WS is not appropriate for clinical use in Japanese children age 0-60 months.

**Table 1 (abstract P2-4-5).**
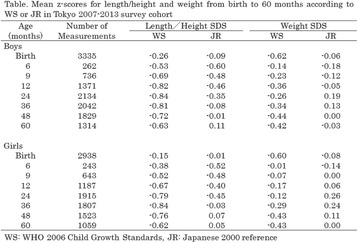
Mean z-scores for lenght/height and weight from birth to 60 months according to WS or JR in Tokyo 2007-2013 survey cohort

## P2-4-6 Postnatal growth retardation and low Insulin-like growth factor-1 production occurs by PI3KCA flameshift mutation (p.Ser552ThrfsX6) in patient with Macrocephaly-capillary malformation

### Hideaki Yagasaki^1^, Tomohiro Saito^2^, Mami Kobayashi^2^, Hiromune Narusawa^2^, Mie Mochizuki^1^, Kazumasa Sato^1^, Kisho Kobayashi^1^, Masanori Ohta^1^, Kenji Ohyama^1^, Kanji Sugita^1^

#### ^1^Faculty of Medicine / Department of Pediatrics, University of Yamanashi; ^2^Departments of Pediatrics and Neonatology, Yamanashi Prefectural Central Hospital

**Introduction**: Activating mutations in the phosphatidylinositol 3-kinase (PI3K) gene PI3KCA cause the macrocephaly–capillary malformation (MCAP) overgrowth syndrome. We detected a novel germline frameshift mutation in the PI3KCA gene in a patient with MCAP previously reported in JSPE2013 . We investigated the phenotype–genotype relationship and the pathophysiology of the PI3KCA activating mutation in this patient.

**Patient and Methods:** A male infant was born at 30 weeks gestation weighing 2998 g (+6.63 S.D), with a body length of 47.0 cm (+4.01 S.D) and head circumference of 36.5 cm (+6.23 S.D ). Physical examination revealed a peculiar face with hypertelorism, anteverted nares, thick lips, and frontal bossing and capillary malformation with a broad, prominent forehead. He also presented with hydrocephalus requiring a VP shunt. He was diagnosed with MCAP based on his clinical characteristics. At 10 months of age, the patient presented with convulsions with hypoglycemia (27 mg/dl ) and low growth hormone reaction (3.37 ng/ml). He was treated with growth and thyroid hormone replacements and assisted ventilation. An oral glucose tolerance test at 3 years old showed a massive increase in glucose to 280 mg/dl with an insufficient insulin reaction, falling to 24 mg/dl after the test. He showed severe growth retardation, and his insulin-like growth factor-1 (IGF-1) levels were extremely low or undetectable after growth hormone replacement. The patient died suddenly of unknown causes at 7 years 9 months old. Sequence analysis revealed that the patient had a previously unreported de novo heterozygous c.1658_1659delGTinsC mutation in exon 10 of the PIK3CA gene, which could have caused a p.Ser552ThrfsX6 frameshift mutation.

**Discussion**: MCAP has recently been included in the PIK3CA-related overgrowth spectrum related to PI3K hyperactivation. PI3K is distributed in multiple organs and its activity is related to growth, metabolism, and inflammation. The current patient showed characteristic in utero overgrowth and postnatal growth retardation, as well as abnormal blood glucose fluctuation and undetectable IGF-1 production. To the best of our knowledge, this patient represents the first reported case of a p.Ser552ThrfsX6 frameshift mutation in the PI3KCA gene, with an undetermined effect on PI3K activity. This is also the first report of an MCAP patient with PI3K hyperactivity, undetectable IGF-1, and insufficient insulin reaction after growth hormone replacement. Further studies are needed to clarify the effects of PI3KCA mutations on glucose and IGF-1 metabolism.

## P2-4-7 Ocular changes during growth hormone therapy in children with isolated growth hormone deficiency

### Ersoy Betül^1^, Alp Şenay^2^, Kizilay Özalp Deniz^3^, Taneli Fatma^4^, Başer Esin^5^

#### ^1^Division of Pediatric Endocrinology and Metabolism, Celal Bayar University, Faculty of Medicine; ^2^Department of Ophtalmology, Celal Bayar University, Faculty of Medicine; ^3^Division of Pediatric Endocrinology, Celal Bayar University, Faculty of Medicine; ^4^Department of Clinical Biochemistry, Celal Bayar University, Faculty of Medicine; ^5^Department of Ophtalmology, Celal Bayar University, Faculty of Medicine

**Introduction**: Growth Hormone (GH) and Insulin like Growth Factor-1 (IGF-1) are known to be involved in ocular development. However knowledge about ocular effects of GH treatment is very insufficient. The aims of this study were to evaluate ocular changes in children with isolated Growth Hormone Deficiency (IGHD) after 6 months of GH therapy and to determine the relationship between ocular and growth parameters.

**Patients and Methods:** Sixty two pubertal children with isolated GHD (32 male, 30 female) were included this study. Mean age of the patients was 11.8 ± 2.1 years. Ocular parameters comprised of determination of best corrected visual activity (BCVA) and spherical equivalent (SE) of refraction, mesaurements of intraocular pressure (IOP), central corneal thickness (CCT), Goldmann correlated IOP (IOPG), corneal hysteresis (CH), corneal resistance factor (CRF), keratometry, axial length (AL), lens thickness (LT), and retinal nerve fiber layer (RNFL) thickness. These ocular examinations were performed at baseline and repeated at 6 months. IGF-1 levels were also evaluated before and at 6 months of GH therapy.

**Results**: Significant differences in AL and spherical equivalent were found between baseline and after 6 months of GH therapy (p<0.001). AL in GHD children increased significantly compared to baseline after 6 months of GH treatment (22.81 ± 0.09 and 22.83 ± 0.09 mm, respectively), with a corresponding decrease of SE value (1.0 ± 1.0 and 0.89 ± 1.0 diopters, respectively, (p<0.001). There were no correlations between IGF levels and SE values (p>0.05). Positive correlations were found between AL and IGF-1 levels (r=0.427, p=0.022). All other ocular parameters were found to be stable during the study period (p>0.05 for all).

**Conclusion**: GH treatment in children with isolated GHD seems to affect growth of the eye ball as displayed by an increase in mean AL and a decrease in mean SE, which were related with increased IGF-1 levels. More precise conclusions about the effects of GH on eye tissues can be deduced after further follow up our patients.

## P2-4-8 Spanish ECOS Study Analysis: Adherence and Growth Outcomes with Case Studies and Socioeconomic Data

### Maria Dolores Rodríguez -Arnao^1^, Amparo Rodríguez Sánchez^1^, Ignacio Díez López^2^, Joaquín Ramírez Fernándes^3^, Jose Antonio Bermúdez de la Vega^4^, Virginia Ballano^5^, Jenny Alvarez Nieto^6^, Ekaterina Koledova^7^

#### ^1^Paediatric Endocrinology Unit, Pediatric Service, Hospital General Universitario Gregorio Marañón; ^2^Pediatric Endocrinology Unit, Pediatric Service, Hospital Universitario Araba; ^3^Pediatric Service, Hospital Universitario Príncipe de Asturias Pediatric Endocrinology Unit; ^4^Pediatric Endocrinology, Hospital Universitario Virgen Macarena; ^5^Merck S.L., Regional Clinical Operations; ^6^Merck S.L., Endocrinology, Fertility & CM, Medical Department; ^7^Merck, Endocrinology, Global Medical, Safety & CMO

**Background**: The ECOS observational study in Spain (NCT01376921) was designed to evaluate adherence to r-hGH therapy prescribed via the easypod™ electromechanical auto-injector device and to analyse factors that may influence adherence in paediatric patients. Easypod™ administers pre-set doses of Saizen® r-hGH and stores accurate records of each dose and injection taken, which can then be shared with the HCP for evaluation of the patient’s adherence.

**Objectives**: To assess the use and acceptability of easypod™ and adherence to r-hGH therapy, to assess individual patients’ dosing patterns and growth outcomes, and to assess the socioeconomic background of the parents and care givers of these patients.

**Methodology**: Adherence was determined categorically and also as %adherence over time, defined as the number of days with injections received, divided by the number of days with injections planned. Accurate individual adherence data were transcribed directly from the patients’ easypod™, while socioeconomic, demographic, auxological and diagnostic data were obtained from medical notes.

**Results**: The Spanish cohort consisted of 280 children, of whom 240 were included in the final analysis set (52% male). The majority were Caucasian (93.8%), with a diagnosis of growth hormone deficiency (GHD, 60.0%), small for gestational age (SGA, 35.8%), Turner Syndrome (TS, 3.3%) or chronic renal failure (CRF, 0.83%). Despite high overall adherence (median 98.8%, mean 94.5% [95% CI 92.8, 96.3]), growth responses varied. Patterns of missed doses proved highly individual and, in some cases, fluctuated over time, possibly reflecting changes in caregiver or other life circumstances. The different patterns of adherence may be reflected in the growth outcomes for individual cases, although different causes of GHD (idiopathic; organic, history of chemotherapy and/or radiation), with varying histories of r-hGH use and at differing stages of development and puberty can complicate interpretation of growth outcomes. Adherence often falls on entry into adolescence but use of easypod™ enabled physicians to rapidly identify patients with inadequate adherence and to help them to achieve the benefits of r-hGH treatment. At baseline, 79.6% of parents were married or cohabiting, 4.6% divorced, 0.8% single, 2.1% widowed, while 12.2% were unknown or missing. Almost 80% of injection-giving carers were employed, while 31.5% had degree level education, 35.0% had only had school level education, 9.5% had ‘other’ education and 22% had not had this recorded.

**Conclusions**: The majority of children adhere extremely well to their treatment regimen using the easypod™ device. Individual cases show distinctive patterns of adherence and growth outcomes.

## P2-4-9 Influences of GHR-exon 3 and -202 A/C IGFBP3 polymorphisms on 1 year follow-up outcome of growth hormone treatment in Korean children with growth hormone deficiency

### Joon Woo Baek, Yeon Joung Oh, Min Jae Kang, Young Suk Shim, Il Tae Hwang, Seung Yang

#### Department of Pediatrics, Hallym University Sacred Heart Hospital

**Background**: The GHR-exon3 and the -202 A/C IGFBP3 polymorphisms have been suggested to affect responses to recombinant human growth hormone (rhGH) therapy in some individuals with short stature. This study aimed to assess the influences of the two polymorphisms on treatment outcomes in patients with growth hormone deficiency (GHD).

**Methods**: In 72 (32 girls and 40 boys) children with confirmed diagnosis of GHD, genotyping and serial measurements of auxological and endocrinological parameters were performed. Forty-nine patients who remained in the prepubertal state after 1 year of GH treatment were analyzed.

**Results**: Distribution of the GHR-exon3 genotypes was as follows: d3/d3 genotype 2.8%; d3/fl genotype 15.3%; and fl/fl genotype 81.9%. Frequencies of the -202 A/C IGFBP3 genotypes were as follow: A/A genotype 55.5%; A/C genotype 38.9%; and C/C genotype 5.6%. In comparing the d3/d3 and d3/fl group with the fl/fl group, there was no significant difference in first-year height velocity (9.4 ± 2.2 cm vs. 8.1 ± 1.7 cm, P = 0.08). Likewise, comparing the A/A group with the A/C and C/C group, no significant difference was observed in height velocity (8.3 ± 2.0 cm, 8.3 ± 1.7 cm, P = 0.97). Combined analysis of the two polymorphisms showed no significant interaction on the first year height velocity.

**Conclusions**: Our results suggest that the two polymorphisms are not major factors in the modulation of interindividual growth response to GH therapy in Korean children with GHD.

## P2-4-10 Osmotic demyelination syndrome (ODS) during treatment with secondary pseudohypoaldosteronism (PHA) in infant: Case report

### Rei Nishimura^1^, Tetsuya Okazaki^2^, Yuki Kawashima^1^, Naoki Miyahara^1^, Fujimoto Masanobu^1^, Keiichi Hanaki^3^, Susumu Kanzaki^1^

#### ^1^Division of Pediatrics and Perinatology, Faculty of Medecine, Tottori University; ^2^Division of Pediatric Neurology, Faculty of Medecine, Tottori University; ^3^Department of Women's and Chilidren's Family Nursing, Faculty of Medecine, Tottori University

Secondary pseudohypoaldosteronism (PHA) in infancy is a transient condition that is characterized by lack of response to aldosterone in the distal tubule and associated with various uropathy; obstructive uropathy, vesicoureteral reflux, urinary tract infection. PHA causes hyponatremia, hyperkalemia, and metabolic asidosis. Osmotic demyelination syndorome (ODS), which includes both central pontine myelinolysis (CPM) and extrapontine myelinolysis (EPM), is serious neurological complication resulting from rapid correction of serum sodium and associated changes in serum osmolality. Recently we experienced a rare case with secondary PHA presenting with ODS.

A six-month-old Japanese boy was referred to our hospital because of failure to thrive and hyponatremia. He was delivered at 41wk gestation with birth weight 3594 g. He was healthy until the age of three months, when his body weight was 6435 g. However, when he was six-month- old, he began to vomit once a day, was admitted to our hospital because of failure to thrive and hyponatremia. On admission, he had severely dehydration, and his body weight was 5852 g. Laboratory examination indicated severe hyponatremia (109 mEq/L) and hyperkalemia (6.3 mEq/L) and metabolic asidosis. His urinalysis revealed pyuria, and the renal sonography showed grade 2 ureterohydronephrosis of left kidney. As plasma aldosterone and renin activity were markedly elevated, 22,800 pg/mL and 61 ng/ml/hr, he was diagnosed as secondary PHA. His serum sodium was normalized after five days of intravenous sodium replacement and antibiotics, but generalised edema with pleural and peritoneal effusions developed. Through diuretics administration improved his edema, he was presented with hypernatremia (153 mEq/L) on hospital day 10. Two days later, his serum sodium was normalized (143 mEq/L), but he developed generalized seizures. Diffusion– weighted (DW) magnetic resonance imaging (MRI) showed high signal intensity changes in the central pons and bilateral thalami, and he was diagnosed having CPM and EPM. With steroid pulse therapy and fosphenytoin, his neurological status improved gradually. Thereafter his serum electrocyte levels remained normal and serum aldosterone level was decreased. Six months later, both weight gain and developement were normal, MRI showed disappearance of CPM/EPM signs. It is important to consider secondary PHA associated with uropathy in infants with hyponatremia and hyperkalemia. In our case, ODS occurred despite of standardized correction of hyponatremia. Hence chronic hyponatremia might be corrected as slow as possible to prevent ODS.

**Consent for publication:** The authors declare that written informed consent was obtained for publication

## P2-4-11 Diagnostic value of growth hormone stimulation test in short children

### Jeesuk Yu, Seung Yeon Jung

#### Department of Pediatrics, Dankook University Hospital

**Background**: It is important to find the cause of short stature in children. Growth hormone (GH) stimulation test is considered as a ‘gold standard’ for the diagnosis of GH deficiency. Several pharmacologic agents including insulin, glucagon, L-dopa, or clonidine are used for GH stimulation test. This study was designed to compare the diagnostic value of GH stimulation test.

**Method**: Subjects who visited the pediatric endocrine clinic from September 2001 to February 2016 for the evaluation of short stature and underwent GH stimulation test were included in the study. Two or 3 pharmacologic agents were used for GH stimulation test. Growth hormone deficiency was defined when serum peak GH concentration was less than 10 ng/mL in at least two provocation tests. Clinical data were collected retrospectively based on medical records. We compared each test by calculating their sensitivity, specificity, positive predictive value, and negative predictive values, and by ROC curves.

**Result**: A total of 67 short children were included. Forty five (67.2%) were diagnosed with GH deficiency. Average age was 9.05 ± 3.36 years, and 55 subjects were in pre-puberty. Mean height z score was -2.40. Insulin, glucagon, L-dopa, and clonidine test were done in 67, 56, 56, and 10 subjects, and sensitivity for GH deficiency was 95.6%, 61.8%, 71%, and 90%, respectively. Specificity was 50% in insulin, 86.4% in glucagon, and 94.5% in L-dopa test. Positive predictive value for GH deficiency was highest in clonidine test (100%) followed by L-dopa test (96.4%). Negative predictive value was highest in insulin test (84.6%). In ROC curve, areas under the curve of insulin, glucagon, L-dopa tests were 0.742, 0.832, 0.839, respectively. There was no serious adverse event during GHST, except for mild hypoglycemic symptoms or transient vomiting.

**Conclusion**: Any GH stimulation test was relatively safe to perform in short children. Many patients showed different results on different growth hormone stimulation test, therefore it is reasonable to test by using 2 or more agents for the diagnosis. Insulin test showed the highest sensitivity, whereas L-dopa test showed the highest specificity. L-dopa test was the most useful test in diagnosing GH deficiency based on ROC curve.

## P2-4-12 Recuperation of height by the age of 3 for small-for-gestational age (SGA) infants who received care at Neonatal Intensibe Care Unit (NICU)

### Kazuko Yoshimura, Fusako Sasaki, Shinichi Hirose

#### Department of Pediatrics, Fukuoka University

**Background**: By age 2, approximately 90% of small-for-gestational age (SGA) infants catch up to a normal height range. However, it is known that only approximately 70% of SGA infants with less than 32 weeks gestation are able to catch up to a normal height range, which is relatively lower than that for full term infants.

**Purpose**: This study was conducted to delineate the recuperation of height in 3-year-old SGA infants who had been hospitalized and cared for at the Neonatal Intensive Care Unit (NICU).

**Subject and Method**: A total of 299 infants were admitted to NICU from January to December 2012. There were 37 cases of SGAs. Among which 22 infants with height and weight that meet the criteria of eligibility for GH treatment, excluding chromosomal abnormalities, etc., were enrolled in this study.

**Result**: Of the 22 cases, 11 (50%) did not catch up to normal height by age 2. Of these 11 cases, 4 (18%) caught up to normal height between ages 2 and 3, all of which were extremely low birth weight infants under 1500g at birth. Of the 7 cases (32%) eligible for GH treatment after being diagnosed as having short stature as a result of SGA, 5 cases had begun GH treatment between the ages of 3 and 4. Five cases out of 8 infants born with less than 32 weeks gestation were short statured as a result of SGA by age 2, and 3 cases had a height SD score under -2.5 at age 3.

**Discussion**: Many SGA infants in our hospital were extremely low birth weight infants that could not catch up to normal height, and approximately half of them were diagnosed as having short stature as a result of SGA at age 2. However, there were cases of infants under 1500g at birth catching up to normal height between ages 2 and 3, indicating a need for careful observation of changes in height and weight of the infant. Many SGA infants under care at our hospital begin GH treatment at age 3, suggesting a high recognition of SGA treatments among guardians and medical professionals.

## P2-4-13 Growth Hormone Therapy in Turner Syndrome

### Garima Chawla, Manpreet Sethi, Archana Arya

#### Division of Endocrinology, Institute of Child Health, Sir Ganga Ram Hospital

Turner syndrome (TS) is associated with short stature and primary ovarian failure. Owing to association with hypogonadism, patients with Turner Syndrome fail to experience a pubertal growth spurt but continue to grow at a slow rate for several more years. The final height deficit is approximately 20 cm. Growth hormone deficiency is not implicated in Turner short stature. However Recombinant human Growth Hormone (rhGH) has been shown to improve the height potential of girls with TS. Final height in treated girls depends upon height at start of the treatment, GH dose, duration of treatment and age of induction of puberty.

A retrospective study was carried out at Sir Ganga Ram Hospital, New Delhi, a tertiary care hospital in northern India. 27 girls were diagnosed to have Turner Syndrome from January 2008 to December 2015. History alongwith complete clinical details including screening results for other associated abnormalities and laboratory findings were noted. All patients were given daily injections of recombinant growth hormone 0.05mg/kg/day and response was measured in terms of height gain per year. Treatment was continued till height velocity (HV) dropped to 2cm/year. Final heights and near final heights (for patients with ongoing treatment) were recorded and height gain was compared in relation to the duration of therapy.

Fifteen patients who had received rhGH for a duration of more than 12 months were included in the study. Mean age of starting GHT was 11years with minimum age of 8y and maximum being 14.91yr. Mean height gain was 21.64cm. Height gain showed positive correlation with duration of rhGh therapy with a p value of 0.00001. Mean height velocity in the first year of treatment was 9.7cm/year which was significantly higher than the mean height velocity of 3.75cm/year prior to GH therapy (p <0.001). However, HV in second year of treatment 6.42cm/yr was significantly less than HV in first year of treatment (p<0.005). HRT was added at a mean age of 15.25yr at a mean bone age of 13.1yr. Final height was known in six patients. Average final height was 148.2cm + 5.06 with the maximum height being 153.2cm.

To conclude, Growth hormone is an effective treatment for improving final height in patients with Turner syndrome with best outcome if treatment is started at an early age.

## P2-4-14 Efficacies of rhGH alone and rhGH combined with low dose stanozolol on growth velocity of girls with Turner Syndrome

### Hongshan Chen, Dan Li, Minlian Du, Yanhong Li, Huamei Ma, Qiuli Chen, Jun Zhang

#### Department of Pediatrics, The First Affiliated Hospital of Sun Yat-sen University

**Objective**: To compare the different efficacies of recombinant human growth hormone (rhGH) alone and rhGH combined with low dose stanozolol on growth velocity (GV) of girls with Turner syndrome (TS).

**Methods**: Fifty-one girls with TS were divided into two groups: Group 1 (*n*=23) were treated with rhGH alone and group 2 (*n*=28) with rhGH combined with low dose stanozolol both for more than six months. The two groups were compared in terms of GV, height standard deviation score for chronological age (HtSDSca), height standard deviation score for bone age (HtSDSba), and the change of bone age during treatment periods.

**Results**: In the first year, the GV was i6.29 ± 1.44 ) and i8.13 ± 1.87 ) / in group 1 and group 2 respectively DHtSDSca changed from i-3.51 ± 0.99 ) to i-3.19 ± 1.09 ) and i-4.21 ± 1.19 ) to i-3.42 ± 1.06 ) Cand changes in ratio of BA and CA ( Δ a` ^ Δ b`) was (0.60 ± 0.39) and i0.77 ± 0.56 ) group 1 and group 2 respectively. The f u and changes of HtSDSca i group 2 were significantly better than group 1 (p<0.05) Dthe G u was negatively correlated with the age.

**Conclusions**: Compared with the therapy of rhGH alone, the one with rhGH combined with low dosage stanazolol is more effective in improving GV without accelerating bone maturation among the girls with TS.

## P2-4-15 The DNA methylation status at imprinted differentially methylated regions in the patients with hypomorphic mutations in INSR or IGF1R gene

### Keiko Matsubara^1,3^, Yuki Kawashima^2^, Kazuhiko Nakabayashi^3^, Akie Nakamura^1,4^, Takanobu Inoue^1,5^, Kenichiro Hata^3^, Masayo Kagami^1^

#### ^1^Department of Molecular Endocrinology, National Research Institute for Child Health and Development; ^2^Division of Pediatrics and Perinatology, Tottori University; ^3^Department of Maternal-Fetal Biology, National Research Institute for Child Health and Development; ^4^Department of pediatrics, Hokkaido University, Department of pediatrics; ^5^University of Tokyo

**Introduction**: Imprinted genes are expressed in parent-of-origin specific manner and play an important role in growth and development. In humans, aberrant expression of some imprinted genes causes imprinting disorders. Insulin and Insulin-like Growth Factor 1 (IGF1) are crucial in maintaining the whole organism's homeostasis. These effects are mediated through ligand activation of the tyrosine kinase activity of their receptors, Insulin receptors (IRs) and IGF1 receptors (IGF1Rs). Boucher reported that loss of IRs and IGF1R in adipose cells of mice induced the change of expression of some imprinted genes through changes of DNA methylation status at imprinted differentially methylated regions (iDMRs) independently of ligand activation (Boucher, 2014). However, it is not known whether IRs or IGF1Rs had such effects in humans. Here, we studied the methylation status at iDMRs using gDNA of the patients with hypomorphic mutations in INSR or IGF1R gene.

**Methods**: We performed DNA methylation analysis using gDNA extracted from peripheral blood lymphocytes of two patients with Donohue syndrome due to compound heterozygous mutations in INSR gene (patient 1: T910M/E1047K, 2: C280T/R863X) and three with short stature due to heterozygous mutations in IGF1R gene (patient 3: D1135E, 4: Q1250X, 5: W1249X) by Illumina HumanMethylation450 BeadChip. We compared the methylation status at known 51 iDMRs of these patients with those of 36 normal controls.

**Results**: We inquired into the data of known 51 iDMRs. Overall, there was no difference in the methylation status of gDNA between the patients with mutations in INSR or IGF1R gene and the normal control, indicating that methylation defects at iDMRs were not observed in the patients.

**Discussion**: In this study, we analyzed methylation status at iDMRs using the blood of five patients with hypomorphic mutations in INSR or IGF1R gene, and found no methylation defect. This result is not consistent with that of the experiment in which preadipocytes and adipose tissue of mice lacking IRs with or without IGF1Rs exhibited the change of DNA methylation at some iDMRs. The reason for this discrepancy would be the difference of somatic tissues examined and species-specific differences between humans and mice. Besides, the mechanisms causing IRs and IGF1Rs dysfunction were different between previous experiment and our study. For understanding the effect of IRs and IGF1Rs on the change of epigenome in humans, different somatic tissues other than blood, or more patients with other genetic background causing IRs or IGF1R dysfunction need to be examined.

## P2-4-16 A case of idiopathic short stature with novel heterozygous p.S219L mutation of GHR gene Marie

### Mitani ^1^, Tomohiro Inoguchi^1^, Tsutomu Kamimaki^1^, Takeshi Sato^2^, Maki Fukami^3^, Tomonobu Hasegawa^2^

#### ^1^Department of Pediatrics, Shizuoka City Shimizu Hospital; ^2^Department of Pediatrics, Keio University School of Medicine; ^3^Department of Molecular Endocrinology, National Center of Child Health and Development3 )

## P2-4-17 Growth Hormone Deficiency in Non- transfusion Dependent Thalassemia with Short Stature

### Khomsak Srilanchakon^1^, Navaporn Puangpakisiri^2^, Darintr Sosothikul^3^, Somlak Tongmeesee^4^, Benjamas Tanyong^4^, Yodkwan Aphikulchatkit^5^, Vichit Supornsilchai^1^

#### ^1^Division of Endocrinology, Department of Pediatrics, Chulalongkorn University; ^2^Department of Pediatrics, Chulalongkorn University; ^3^Division of Pediatric Hematology and Oncology, Department of Pediatrics, Chulalongkorn University; ^4^Department of Pediatrics, Chonburi Hospital, Chonburi; ^5^Department of Pediatrics, Police General Hospital

**Background**: Short stature is a major problem in both transfusion dependent and non-transfusion dependent thalassemia (NTDT) patients. Defects in growth hormone (GH) secretion have been reported as a cause of short stature in thalassemia major patients while being an infrequently mentioned cause in NTDT patients. The objectives of this study were to evaluate the percentage of growth hormone deficiency (GHD) present in NTDT patients presenting with short stature (SS) using two GH provocative tests (Insulin tolerance test (ITT) and Clonidine test), and to define associated factors with GHD.

**Methods**: A cross-sectional study was conducted in patients who were diagnosed as NTDT with short stature. Demographic data: gender, age, weight, height, history of blood transfusions, medications, hemoglobin, serum ferritin, growth hormone provocative test results, insulin-like growth factor-1 (IGF-1) and insulin-like growth factor binding protein-3 (IGFBP-3) levels were collected. Two different GH provocative tests were performed in NTDT cases and the percentage of GH deficiency was calculated. The predictive factors of GH deficiency in NTDT patients were analyzed.

**Results**: Five out of thirteen patients (38%) who were diagnosed as NTDT with short stature had growth hormone deficiency confirmed by using two different GH provocative tests. Four of them (30%) were diagnosed with growth hormone deficiency by decreased height velocity and GH provocative tests. There was no factors predicting growth hormone deficiency in NTDT patients with short stature found.

**Conclusions**: Growth hormone deficiency is not uncommon in patients with NTDT and short stature. This study found no factors that predicted growth hormone deficiency, possibly due to its small sample size.

**Key words**: non-transfusion dependent thalassemia (NTDT), growth hormone deficiency (GHD), Insulin tolerance test (ITT), Clonidine test

**Table 1 (abstract P2-4-17).**
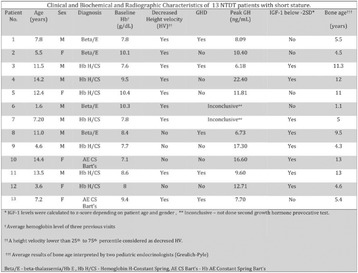
Clinical and biochemical and radiographic characteristics of 13 NTDT patients with cohort stature

## P2-4-18 Educational Program for School Nurses in Taiwan to Raise Awareness of Growth Problems and Referral Processes and Facilitate Early Diagnosis for Pupils with Short Stature

### Yi-Lei Wu^1^, Pen-Hua Su^2^, Chia-Feng Yeh^3^, I-Hsuan Wu^3^

#### ^1^Dept of Paediatrics, Changhua Christian Hospital; ^2^Dept of Paediatrics, Chung Shan Medical University Hospital; ^3^Merck Taiwan Ltd

**Background**: Short stature (SS) is defined as height or length below the third percentile for age and sex. Although short stature is a common healthcare issue with diverse etiologies, delayed diagnosis is extremely common worldwide. The practice of population height measurement to identify short stature varies widely across countries. In Taiwan, elementary or high school nurses are required to measure children’ s heights every semester and to refer children with short stature to their pediatrician. Because school nurses are the first line contacts for children and parents, they need to have adequate competencies and knowledge regarding diagnosis and treatment of short stature. However, although there is a high proportion of referrals, nurses in Taiwan are not specialized in different disease areas. School nurses might not be familiar with the post-referral processes for growth problems, such as the evaluation criteria and investigations that are required for this endocrinological disorder.

**Methods**: In July 2015, the Child Growth Association of Taiwan held two disease awareness programs among school nurses. The objective of the programs was to educate nurses on the disease and its psychological aspects and on the referral processes and methods of investigation. This, it was believed, would help lead to earlier diagnosis and would also enable the nurses to provide psychological and medical support for short stature patients and their families.

Our group of physicians developed the training programs which were attended by 850 school nurses. Two experienced pediatric endocrinologists provided insights into the diagnosis, management and care of short stature patients.

**Results**: These sessions triggered many questions and an interactive exchange between the physicians and nurses during a subsequent Q&A session. Questions included “When to refer?”, “What are the indications for GH?” and “How can parents or patients approach hospital healthcare professionals?”. A post-program survey was conducted and the majority of participants (93.3%) gave positive feedback. Many (83.3%) mentioned that they felt had better understanding of disease and treatment for short stature and felt more confident about counselling the patients and their parents.

**Conclusions**: The disease awareness programs were very successful and were well received. This initiative has emphasized that care of short stature patients resided not only with doctors but also with school nurses, who can play a vital role in identifying, counselling and following up patients with short stature.

## P2-4-19 Secular trend in growth pattern of urban Indonesian children and adolescents: comparison to WHO Growth Reference 2007

### Madarina Juria^1^, EMY Huriyati^2^

#### ^1^Department of Child Health, Faculty of Medicine, Universitas Gadjah Mada/ Dr. Sardjito Hospital; ^2^Department of Nutrition and Health, Faculty of Medicine, Universitas Gadjah Mada

**Background**: Growth pattern of children and adolescents of certain race or ethnic may differ from other races or ethnics. Likewise, secular trend may change the pattern of growth across time.

**Aims**: To assess the secular trends in growth of children and adolescents in Indonesia using data collected between 1998 to 2014.

**Methods**: Data of urban pre-pubertal children, i.e. between 5 to 8 years for girls and 5 to 9 years for boys, were collected in 1998 and 2003. Data for urban adolescents were collected in 2003 and 2012 for junior high school adolescent and in 2003 and 2014 for senior high school adolescents. Data of age, sex and height were converted into height standard deviation scores (SDS) based on WHO Growth Reference 2007.

**Results**: Data of 1708 pre-pubertal children of year 1998 were compared to 2276 pre-pubertal children of 2005. Like wise, data of 4765 urban junior high school adolescents of year 2003 were compared to 1890 urban junior high school adolescents of year 2012, while data of 775 urban senior high school adolescents of year 2003 were compared to data of 3968 urban senior high school adolescents of year 2014. Overall, there were improvements of around 0.1 to 0.3 SDS in height-for-age SDS between the two measurements. The best improvement was attained at pre-pubertal age, i.e. from mean (SD) height SDS of -0.99(0.9) to -0.63(1.3). Improvement in adolescents was less.

**Conclusion**: Secular trend in growth of Indonesian children is better before puberty.

## P2-4-20 Schimke immuno-osseous dysplasia (SIOD) : a case report and review

### Ziqin Liu, Xiaobo Chen, Fuying Song, Ying Liu

#### Division of Endocrinology and Metabolism, Capital Institute of Pediatrics

**Objective**: We reported on a 10 years old girl with Schimke immuno-osseous dysplasia (SIOD) and literature review to provide clinical and genetic materials of this rare disease.

**Methods**: Retrospective analysis of a patient who admitted because of short stature, she had normal intellectual and neurological development. Growth in early childhood and psychomotor development were normal. Growth retardation was first noticed at 5years; after that, her growth velocity was 3~4 cm/year. Her weight was 36 kg (p50-75; average for 11 years) and her stature was 123 cm (p<3; average for 7 years), with a short upper segment (upper/lower segment measurements: 59/64cm), and slightly lumbar lordosis. She had short neck and triangular face. She was found to have special facial features Cshe had multiple small café-au-lait spots Cosseous dysplasia. The blood smear as follows: hemoglobin 14.5g/dl ;lymphopenia 1.13 × 109( lymphocytes/l, normal 1.5 × 109–6.5 × 109) and platelets: 345 × 109 /l(normal 150 × 109–400 × 109). Clinical chemistry demonstrated triglyceride 0.52mmol/L(0-1.69) and hypertryglyceridemia2.13 mmol/L(0.86-1.87), albumin 44.7 g/dl(normal 35–55) and total protein in serum 68.9g/L ( normal 60–80), while glucose, electrolytes and creatinine were normal. The 24-h urine specimen demonstrated features of proteinuria. She was suspected as SIOD. After informed consent Cshe and her parents’s DNA were extracted from blood for detect gene SMARCALl. Results:The patient was a milder case. A novel Nonsense c.445C>T ip.Q149X ) was found. One reported missense mutations c.1933C>T ip.R645C ) was detected.

**Conclusions**: c.445C>T ip.Q149X ) was a novel nonsense mutation which cause polypeptide chain synthesis stopped. Compared with reported 3 SIOD cases in China, we found that main features of SIOD were short stature, cell immune deficiency, osseous dysplasia, café-au-lait spots and proteinuria. The genetype of SIOD was variable. The relationship between genetype and phenotype of SIOD remained elusive.

Key words: Schimke immuno-osseous dysplasia; short stature; SMARCALl;mutation

## P2-4-21 Efficacy of GH treatment in 6 cases of SGA short stature born with extremely low birth weight

### Seiko Hirose, Tae Kimura, Maki Saitoh, Ayako Ozawa, Erina Ono, Yukitoshi Tanabe, Masahisa Kobayashi, Ichiro Miyata

#### Department of Pediatrics, The Jikei University School of Medicine

**Background**: The efficacy of growth hormone (GH) treatment for short children born small for gestational age (SGA) has been reported. However, its effect for SGA short children born with extremely low birth weight remains unclear. In the present study, we investigated the efficacy of medium- to long-term GH treatment for those children.

**Subjects and Methods**: Subjects were 6 cases that were born with extremely low birth weight at our hospital and diagnosed as having SGA short stature at the age of 3 years. Their gestational age was ranged from 24 weeks and 3 days to 29 weeks and 6 days, and their birth weight was 417 g to 802 g. For all cases, 0.23 mg/kg/week of GH was started from 3 years old. Then, we determined the dosage of GH based on growth rate (cm/yr), Δheight SDS andΔIGF- T SDS for every one year, and examined the efficacy of treatment for 3~7 years. Furthermore, we also assessed bone age/chronological age (BA/CA) ratio longitudinally.

**Results**: The averages of growth rate,Δheight SDS andΔIGF- T SDS after one year were 8.32 cm/yr, 0.66 and 1.72, respectively. In regard to the dosage of GH from the second year, the same dose (0.23 mg/kg/week) was continued in 3 cases (Δheight SDS > 0.75). Additionally, the dose was increased to 0.35 mg/kg/week in 2 cases (0.5Δheight SDS < 0.75), and increased to 0.47 mg/kg/week in one case (Δheight SDS < 0.5). Growth rate in all cases showed each maximum value in one year from the start of treatment or the increase of GH dosage, but decreasing trend was observed subsequently. Maximum Δheight SDS in each case was 0.6 to 0.9. Δ IGF- T SDS in all cases was also reached each peak value in the first or second year, and then was decreased. Moreover, BA/CA ratio of each case was 0.6 to 1.0 before the start of this study, but it never exceeded 1.2 during the treatment period.

**Discussion**: GH treatment was effective in growth promoting for SGA short children born with extremely low birth weight. Especially, the response of growth was higher in early phase of treatment course or just after increase of the dosage. It’s important to assess the individual response and to adjust the dosage in early phase.

## P2-4-22 A patient with maternal uniparental disomy chromosome 14 treated with growth hormone

### Misuki Kobayashi^1^, Masayo Yamazaki^1^, Makiko Oguma^1^, Koji Yokoyama^1^, Ayumi Matsumoto^1^, Yasuyuki Nozaki^1,2^, Akie Nakamura^3^, Masayo Kagami^3^, Toshihiro Tajima^1^, Takanori Yamagata^1^

#### ^1^Department of Pediatrics, Jichi Medical University; ^2^Department of Pediatrics, Shin-Oyama City Hospital; ^3^Department of Molecular Endocrinology, National Center for Child Health and Development

**Background**: Maternal uniparental disomy of chromosome 14 [matUPD(14)] is a genetic condition of inherited both homologue of a maternal chromosome 14. Its clinical appearance is very similar to Prader-Willi syndrome (PWS) and the differential diagnosis is important. Recently, the efficacy of GH treatment on short stature and hypotonia has been reported in matUPD(14). Here we report a matUPD(14) patient and the effect of GH treatment.

**Patient**: The patient is a three years old boy, born at 36 weeks of gestation to nonconsanguineous parents, with a birthweight of 1436 g (-2.8 SD) and a body length of 40.5 cm (- 3.0 SD) shown to be small for gestational age (SGA). The pregnancy was established by intracytoplasmic sperm injection and frozen embryo transfer. Oligohydroamnios and fetal growth restriction had been observed since 30 weeks of gestation. After birth, he showed hypotonia, poor feeding, small hands and feet, cryptorchidism and peculiar face, however, his methylation testing for PWS was negative. At 2 years of age, further genetic analysis of MEG3 methylation testing and chromosome 14 polymorphism analysis were demonstrated and he had matUPD(14). His growth failure had not improved by the age of three, and his height was 80.4 cm (-3.6 SD) and weight was 9.8 kg (-2.5 SD) at 3 years of age. Endocrinological evaluation showed that the serum IGF-1 level was 77 ng/ml and GH secretion after arginine tolerance test (ATT) was within normal range. GH treatment was started as short stature for SGA at this time. Three months after GH treatment, his growth velocity has been improved and GH treatment is now going on.

**Discussion**: Since the efficacy of GH treatment for matUPD(14) has been reported in only a few cases, its benefit could not be determined yet. Therefore, careful follow-up of each case is required. Further accumulation of matUPD(14) cases treated with GH is important to determine the effect of GH treatment.

**Consent for publication:** The authors declare that written informed consent was obtained for publication.

## P2-4-23 Deletions at 6q14.1-q15 in an 8-year-old girl with developmental delay, autistic disorder and stereotyped movement

### Qing Zhou, Chao Chun Zou

#### Zhejiang University School of Medicine Children's Hospital, Endocrinology

## P2-4-24 Laron syndrome-a rare cause of short stature

### Sreejith Mullassery, Hammadur Rahman, Rajesh Khadgawat

#### Department of Endocrinology & Metabolism, All India Institute of Medical Sciences

Primary growth hormone (GH) resistance or GH insensitivity syndrome, a very rare cause of short stature, is characterized by a clinical appearance of severe growth hormone deficiency with high levels of circulating GH and low serum insulin-like growth factor 1(IGF 1) values. It is caused by deletions or mutations in the growth hormone receptor gene or by post-receptor defects.These patients are refractory to GH therapy and respond only to recombinant IGF 1.

A 12 year old girl, born of a second degree consanguineous marriage presented with failure to gain height since one year of age. She was born of a full term normal vaginal delivery without any significant antenatal or postnatal complications. Her birth weight was 1.8 kg while birth length was unknown. There was no history suggestive of any chronic medical illness.No history suggestive of hypoglycemia in the past. The child had an average scholastic performance.There was no family history of short stature.

Examination revealed subtle facial dysmorphism (prominent forehead, depressed nasal bridge, sparse hair, blue sclera). Tanner staging was A1B1P1. Her height was 99.5cm (<5th centile; Ht SDS -8.12, Indian standards) while weight was 14.7kg (BMI - 14.84kg/m2). The mean parental height was 149 cm. There were no stigmata of turner syndrome. External genitalia was normal. Clinical systemic examination was unremarkable. Investigations revealed a normal hemogram, renal and liver function tests. Thyroid function tests and serum cortisol were also normal. Skeletal survey ruled out skeletal dysplasia. Bone age was 9 years. Pituitary gland was normal on neuroimaging.Karyotyping was 46XX. Basal serum GH level (31.18 ng/ml) and peak level on provocation with clonidine (93.56ng/ml) were high while serum IGF1 (11.3ng/ml) and IGFBP3 (411.25 ng/dl) were low, confirming diagnosis of GH insensitivity. She was started on recombinant IGF1 therapy (Inj Mecasermin, IPSEN Limited; 40ug/kg twice daily). On six months follow-up, she gained 5cm in height suggesting a satisfactory response to therapy.

**Consent for publication:** The authors declare that written informed consent was obtained for publication.

## P2-4-25 Localized lipoatrophy induced by recombinant human growth hormone therapy in a patient with growth hormone deficiency

### Shuichi Yatsuga, Takako Sasaki, Junko Nishioka, Miyuki Kitamura, Yasutoshi Koga

#### Department of Pediatrics and Child Health, Kurume University School of Medicine

**Introduction**: One of the complications of recombinant human growth hormone (rhGH) therapy is lipoatrophy, although rare, the prevalence increases in female adults compared to men. In children, lipoatrophy is still more rarely reported. The mechanism of lipoatrophy is unknown, however, lipoatrophy for rhGH injection is said to be caused by insufficient rotation sites. The present case developed lipoatrophy in rhGH injection, and recovered after rotation of the injection sites.

**Case**: A 2.7 year old boy was born at 40 weeks and 5 days gestation; height was 49 cm and weight was 3480 g. He was referred to our hospital for short stature. Additional complications were mild developmental delay and mild autistic disorder. His height was 81.1 cm (-2.94 SD) and his weight was 11.7 kg on consultation. Screening tests were performed for short stature. Thyroid function was within normal range, bone age was delayed by 1.5 years, and IGF-I was low level at 24 ng/mL. We performed GH stimulating tests, using arginine and clonidine. The peak levels of GH were 1.59 ng/mL and 2.70 ng/mL, respectively, resulting in the diagnosis of severe GHD. Pituitary was normal size by enhanced MRI. RnGH therapy (0.20 mg/kg/week) was started. Administration was performed at the same site, subsequently, lipoatrophy gradually developed during the next 4 months. Injection of rhGH was discontinued, and after 1 month, injection of rhGH (0.19 mg/kg/week) was resumed, taking into care to administer at several sites. No lipoatrophy reoccurred using rhGH, and currently his height is 90.6 cm (-2.62 SD), and his weight is 12.6 kg (-1.6 SD) at 4.1 years old.

**Discussion**: Lipoatrophy is a very rare yet debilitating complication of rhGH injection in children. Past reports conclude that lipoatrophy develops in severe GHD due to immune tolerance deficiency. On the other hand, lipoatrophy may be induced since rhGH directly reacts to adipose tissue.

**Conclusions**: Caution is warranted for daily subcutaneous rhGH injection without injection site rotation, which may induce localized lipoatrophy for especially severe GHD patients.

**Consent for publication:** The authors declare that written informed consent was obtained for publication.

## P2-5-1 Effectiveness and Drawbacks of Methimazol + Inorganic Iodine Combination Therapy for Children with Severe Hyperthyroidism due to Graves’ Disease

### Hidemi Ohye^1^, Yo Kunii^1^, Masako Matsumoto^1^, Miho Suzuki^1^, Natsuko Watanabe^1^, Ai Yoshihara^1^, Nami Suzuki^1^, Kenji Iwaku^1^, Kei Endo^1^, Naomi Hattori^1^, Ruriko Suzuki^1^, Koji Mukasa^1^, Jaeduk Yoshimura Noh^1^, Kiminori Sugino^2^, Koichi Ito^2^

#### ^1^Department of Internal Medicine, Ito Hospital; ^2^Department of Surgery, Ito Hospital

**Background:** The effectiveness of Methimazole (MMI) + inorganic iodine combination therapy in rapid improvement of hyperthyroidism for adult with Graves’ disease (GD) has been reported, while there is little information for child and adolescent patients.

**Object**: To study effectiveness of MMI + potassium iodine (KI) combination as an initial treatment by comparing MMI alone and to reveal drawbacks of MMI+KI therapy in child and adolescent patients with severe hyperthyroidism due to GD.

**Methods**: We retrospectively studied 235 patients aged 18 years or younger who were newly diagnosed with GD between January 2005 and June 2015 (Median age 16 (7-18) yr, 207 females). Serum FT4 level at diagnosis was more than 7.0 ng/dl and observation period after ATD initiation was more than 6 months (Median period 56.2 months (6.2-621.6)). Patients were initially treated with MMI15mg+KI50mg daily (MK group, n=150) or MMI30mg daily (M group, n=85).

**Results**: The prevalence of all adverse events induced by MMI was 6.7 % in MK group and 17.6 % in M group (P<0.01). FT4 level was more rapidly normalized in MK group (Median 26 days (13-218)) than in M group (Median 49 days (14-168)) (p<0.01) in 182 patients excluding 53 patients who switched to other treatments or increased MMI dose before FT4 normalization. One drawback of MK therapy was that the rate of patients who required increase of MMI dose to normalize FT4 was significantly higher in MK group (39 patients (26.0 %)) than in M group (7 patients (7.1%)) (*p*<0.01). Thyroid volume at diagnosis was significantly larger in MMI dose increase group. (*p*<0.01) The other drawback was that FT4 rebound after KI withdrawal was found in 41 of 132 patients (31.1 %) whose data around KI withdrawal was available. KI treatment duration was significantly shorter and FT4 level at KI withdrawal was significantly higher in the rebound group (median 55 days (14-397) and 1.1 ng/dl (0.17-2.63), respectively) than in the no rebound group (median 90 days (14-1061) and 0.81 ng/dl (0.18-1.60), respectively) (*P*<0.01).

**Conclusion**: Combination of MMI and KI is an effective initial treatment for rapid improvement of hyperthyroidism and reducing adverse events in child and adolescent patients with severe GD. However, considerations in combined use of KI; Necessity of MMI dose increase to normalize FT4 and possibility of FT4 rebound after KI withdrawal in some patients, should be recognized.

## P2-5-2 Clinical relationships between genotype and phenotype in congenital hypothyroidism

### Tatsushi Tanaka, Atsushi Suzuki, Kohei Aoyama, Haruo Mizuno

#### Department of Pediatrics and Neonatology, Nagoya City University, Graduate School of Medical Science

**Background/Aims**: Congenital hypothyroidism (CH), occurring in approximately 1:2000 to 1:4000 newborns, is the most frequent congenital endocrine disorder. Several causative genes for CH have been reported. This study aimed to examine the phenotype-genotype relationship in CH with identified genetic abnormality.

**Methods**: Fifteen patients with CH, including two family cases with identified genetic abnormalities, who were treated in the Nagoya City University Hospital, were enrolled in this study. The genetic abnormalities causing CH were identified by targeted next-generation sequencing. The relationship between the genetic abnormalities and phenotypes was determined based on the medical records.

**Results**: Of these 15 patients, nine had mutation in DUOX2; four, in TSHR; and two, in TPO, All mutations were biallelic mutations. Three patients of TSHR mutation harbored the homozygous R450H mutation. The nine patients with DUOX2 mutations and four with TSHR mutation showed mean serum TSH values of 174.1 μ U/mL (from 7.1 to 743.8 μ U/mL) and 24.9 μ U/mL (from 19.8 to 33.3 μ U/mL) and mean serum fT4 levels of 0.6 ng/dL (from 0.3 to 0.7 ng/dL) and 1.4 ng/dL (from 1.3 to 1.5 ng/dL) respectively, at the first visit to the hospital. Of the patients with DUOX2 mutation, three were suspected of excessive iodine intake because of the elevated urine iodine concentrations or episode of hysterosalpingography. These patients had higher TSH levels than did patients without suspicion of excessive iodine intake did. The size of the distal femur bone nucleus at CH diagnosis was normal in all patients, except the one with TPO mutation. The mean thyroglobulin levels before treatment in four patients with DUOX2 mutation, one with TSHR mutation, and one with TPO mutation were 1248.6 ng/mL, 85.9 ng/mL, and 4610.0 ng/mL, respectively. The patients were treated with l-thyroxine, based on the identified mutation, at the following average doses: DUOX2 mutation except two patients who had stopped taking l-thyroxine 1.8μ g/kg/day (age range, 1-10years); TSHR mutation, 3.9μg/kg/day (age range, 1-11years); and TPO mutation, 2.5μg/kg/day (patient age, 3 years and 15 years).

**Conclusion**: Patients with biallelic TSHR mutation did not stop taking l-thyroxine. In contrast, patients with biallelic DUOX2 mutation would stop taking l-thyroxine or only take a very little amount. Thus, genetic analysis is beneficial for predicting the clinical course of CH.

## P2-5-3 Effectiveness and safety of radioactive iodine therapy in childhood Graves’ disease in Khon Kaen, Thailand

### Ouyporn Panamonta^1^, Songpon Getsuwan^1^, Pattara Wiromrat^1^, Manat Panamonta^1^, Sunphat Paireepinas^2^

#### ^1^Faculty of Medicine, Division of Endocrinology, Department of Pediatrics, Khon Kaen University; ^2^Faculty of Medicine, Division of Nuclear Radiology, Department of Radiology, Khon Kaen University

**Background**: Graves’ disease (GD) is the most common cause of hyperthyroidism in children and adolescents. Treatments consist of medication, radioactive iodine (RAI) therapy and surgery. Currently, RAI therapy is the first line treatment in many medical centers.

**Objective**: To evaluate the effectiveness and safety of RAI therapy in childhood Graves’ disease.

**Methods**: A retrospective study was performed in 46 GD patients, aged at onset ≤ 15 years, who had undergone RAI therapy at the aged ≥ 10 years. Goiter grading, evidence of hypothyroidism, severity of ophthalmopathy, RAI dosage and side effects of RAI therapy were evaluated.

**Results**: The cure rate was 95.6%. All participating patients had goiter reduction (*p*=0.005). Hypothyroidism was induced in 33 (71.7%) and 11 (23.9%) patients after the first and second RAI therapy. The freeT4 level and the total RAI dosage were significantly higher in the patients with failure response (p=0.001). The average time to induce hypothyroidism after the first RAI therapy was 127.5 (IQR:94.5-123.0) days. All of the patients had improvement of ophthalmopathy and none had thyroid carcinoma during the follw-up period of 42.5 (IQR: 17-52) months.

**Conclusion**: Radioactive iodine therapy is effective and safe in the treatment of children and adolescents with Graves’ disease.

## P2-5-4 Elevation of serum thyroid-stimulating hormone levels due to excess iodine in Japanese neonates

### Mika Makimura^1^, Toru Sawano^1^, Hisashi Yamashita^2^, Toshinori Nakashima^1^, Yoshihiro Sakemi^1^, Naoko Matsumoto^2^

#### ^1^Department of Pediatrics, National Hospital Organization Kokura Medical Center; ^2^Department of Pediatrics, Kitakyushu Municipal Medical Center

**Purpose**: To clarify the maximum tolerable level of iodine intake in Japanese neonates.

**Subjects and methods**: This study included a prospective observational study performed at Kitakyushu Municipal Medical Center from September 2013 through November 2014 (Group A), and a retrospective observational study performed at National Hospital Organization Kokura medical center from September 2015 through June 2016 (Group B). Subjects of Group A were born to mothers with a family history of thyroid disease (within third-degree close relatives). Subjects of Group B tested positive at the screening or had symptoms of hypothyroidism. Both groups comprised term or near-term newborns without severe complications. Serum thyroid-stimulating hormone (TSH) and free thyroxine (FT4) levels were measured in the first 5 days of life (DOL) and/or at 1 month of life (MOL). Thyroid autoantibodies, thyroglobulin, and urinary iodine (UI) were measured within 1 month after birth.

**Results**: In Group A, 13 newborns of 840 pregnant women met the selection criteria and were enrolled in this study. Three of the 13 newborns negative for thyroid autoantibodies showed slightly elevated serum TSH levels and high UI levels, but normal FT4 levels. There was a positive correlation between serum TSH and UI levels. When UI >1,500 μ g/L or >8,000 μ g/gram of creatinine was used as a cut-off, elevation of serum TSH levels exceeding the standard was predicted with 100% sensitivity and specificity. In Group B, two newborns negative for thyroid autoantibodies showed slightly elevated serum TSH and high UI levels. One tested positive and the other negative on the mass- screening test. These newborns had prolonged jaundice, poor weight gain, and/or umbilical hernias.

**Conclusion**: Prenatal exposure to excessive amounts of iodine can cause elevation of serum TSH levels and show a tendency to demonstrate excessive and prolonged TSH responses compared to older children and adults. In our study, serum TSH levels were within standard limits or slightly high (MS-negative) at 5 DOL, but gradually increased from 5 DOL to 1 MOL. These findings suggest the possibility of delayed- onset hypothyroidism. In group A, iodine-induced thyroid dysfunction was subclinical, and no cases required treatment with levothyroxine. However, in Group B, 2 neonates had clinical hypothyroidism symptoms and required levothyroxine therapy. There are many reports of iodine-induced overt thyroid dysfunction and/or goiter, suggesting that not only iodine dosing but also factors such as heredity or environment are involved, whether or not symptoms of overt hypothyroidism are present.

## P2-5-5 The management of the children born from mothers with hypothyroidism/subclinical hypothyroidism

### Yasuko Ogiwara, Tomoko Yoshida, Kanako Nakao, Yumiko Terada, Yuta Chiba, Kazuko Mizutani, Yusuke Fujisawa, Yasuhiro Naiki, Reiko Horikawa

#### Division of Endocrinology and Metabolism / Department of Pediatrics, National Center for Child Health and Development

**Background**: Hypothyroidism during pregnancy can lead to preeclampsia, anemia, and miscarriage. Maternal hypothyroidism also affects their children by causing intrauterine growth retardation and/or developmental delay; previous report showed even modest maternal hypothyroidism relates to the lower IQ of their children when compared to the euthyroid controls.

**Objectives**: To evaluate thyroid function after birth in children of hypothyroid mothers

**Subjects and methods**: 201 pregnant women, who have diagnosed as having hypothyroidism and been followed at our institute, and their children were involved in this study. The diagnosis of mothers were 95 cases with Hashimoto’s disease (29 inactive, without l-T4)(47.3%), 48 with hypothyroidism after hysterosalpingography (23.9%), 46 with subclinical hypothyroidism (TSH 5μU/mL with thyroid antibodies, 16 without l-T4) (22.9%), 5 with iodine excess (2.5%), 5 with subacute thyroiditis (2.5%), and 2 with drug-induced hypothyroidism (1.0%). Hypothyroidism after thyroidectomy in Graves’ disease was excluded from this study. Thyroid function in their children were checked at day 1 and 5, 1 months, 3 months, 6 months, 1 yr, 2yrs, 3yrs, and 6yrs.

**Results**: In mothers with hypothyroidism/subclinical hypothyroidism during pregnancy, 156 (77.6%) were treated with thyroid hormone replacement and kept euthyroid condition. 15 children were born prematurely (25-36w) and excluded from the following analysis. In 186 children, 165 children were followed up at 1months, 92 at 1 year, 28 at 3 years, 9 at 6 years, respectively. In children, TSH 5μU/mL were observed in 20 (10.8%) at day 5, 17(10.3%) at one month, 7 at 3 months ( 5.2%). Four children (2.2%) were diagnosed as transient hypothyroidism and thyroid hormone replacement was needed. These children were born from Hashimoto mothers, one with thyroid blocking antibody transferred through placenta. One baby born from Hashimoto mother had normal TSH levels at day 5 but follow-up at 1 month revealed elevated TSH level at 79.2 μU/ml and had ectopic thyroid. One with family history of congenital hypothyroidism, and one with thyroid insensitivity showed persistent hypothyroidism and on hormone replacement.

**Conclusion**: 2.2% of children born from Hashimoto mothers needed thyroid hormone replacement and about 10 % of children showed TSH levels higher than 5 μ U/ml at 5 days and 1 month of age, suggesting maternal hypothyroidism has moderate effect on thyroid function in their babies. Close follow-up is necessary, at least until one month, for babies from maternal hypothyroidism.

## P2-5-6 Diverse transcriptional activation patterns of thyroid hormone receptor alpha 1 mutations on different thyroid response elements

### Karn Wejaphikul^1,2^, Anja Van Gucht^1^, W. Edward Visser^1^, Krishna Chatterjee^3^, Theo J. Visser^1^, Robin P. Peeters^1^, Marcel E. Meima^1^

#### ^1^Department of Internal Medicine, Erasmus University Medical Center Rotterdam, the Netherlands; ^2^Department of Pediatrics, Faculty of Medicine, Chiang Mai University; ^3^University of Cambridge, United Kingdom, Wellcome-MRC Institute of Metabolic Science

**Introduction**: Mutations in the ligand binding domain of the thyroid hormone receptor alpha 1 (TR α 1) cause resistance to thyroid hormone alpha (RTH α ). The typical phenotypes include growth retardation, delayed development, macrocephaly, constipation, anemia and abnormal thyroid functions (low/low-normal FT4, high/high-normal FT3, low T4/T3 ratio and normal TSH). However, the phenotypes are various and do not necessarily correlate with the defect in T3 binding. TRs initiate gene transcription by binding to thyroid hormone response elements (TREs), which usually consist of two half site sequences arranged in either direct (DR) or inverted (IR) or everted (ER) repeat configurations. Studies of TR β mutants in RTH β indicate that the orientation of TREs can influence the functional properties of mutant receptors. Because of the high degree of homology between TR α 1 and TR β 1, we hypothesized that the transcriptional activity of TR α 1 mutants could also vary depending on TRE configuration. This may, in part, contribute to phenotypic variability in RTH α patients.

**Methods**: JEG3 cells were transfected with 20 ng of FLAG-tagged wild-type TR α 1 (WT α 1) or mutant TR α 1 expression vectors, 120 ng of luciferase reporter constructs containing either DR (MAL), IR (PAL) or ER (F2) TRE, and 60 ng of pMaxGFP control reporter. After 24 hours, cells were incubated for 24 hours with 0-10,000 nM T3. Luciferase and GFP activities were measured, and half maximal effective T3 concentration (EC50) and maximum responses were determined. Cellular receptor expression was verified by immunoblotting of nuclear extracts with FLAG antibodies.

**Results**: WT and mutant receptors were expressed at similar levels. All mutants showed a clear increase in EC50, which varied between TREs. Overall, the fold increase in EC50 was highest on ER (~80 fold), intermediate on DR (~20 fold) and lowest on IR (~10 fold). The EC50s of L287V α 1 and D211G α 1 were significantly higher compared with WT on all TREs. In contrast, P398H α 1 showed a significantly increased EC50 only on DR and ER, and A263S α 1 only on ER. The maximum response was modestly decreased for most mutants, but reached significance only for T223A α 1 (~60%WT) and P398H α 1 (~40%WT) on all TREs, except for P398H α 1 on ER.

**Conclusions**: The degree of defective transcriptional function of TR α 1 mutants does vary depending on configuration of TRE. This likely contributes to the variable tissue resistance and phenotypes seen in RTH α patients with different TR α mutations.

## P2-5-7 Fetal goitrous hypothyroidism with polyhydramniosis successfully treated by intra-amniotic administration of levothyroxine

### Mami Kobayashi^1,2^, Hideaki Yagasaki^2^, Koichi Makino^2^, Tomohiro Saito^3^, Yumiko Mitsui^2^, Kisho Kobayashi^2^, Atsushi Naito^1^, Atsushi Nemoto^1^, Kanji Sugita^2^

#### ^1^Department of Neonatology, Yamanashi prefectural central hospital; ^2^Department of Pediatrics, University of Yamanashi, Faculty of Medicine; ^3^Department of Pediatrics, Yamanashi prefectural central hospital

Fetal thyroid goiter suspicious of fetal hypothyroidism is a rare phenomenon. Large fetal goiters can obstruct the trachea and esophagus and may cause polyhydramniosis, thus complicating labor and causing neonatal respiratory distress. We present a case of fetal goitrous hypothyroidism treated by administration of levothyroxine (L-T4) into the maternal amniotic fluid. A 32-year-old woman was referred to our hospital because of polyhydramniosis with fetal thyroid goiter at 32 weeks of gestation. She was diagnosed with Graves’ disease and treated with propylthiouracil 150 mg/day during pregnancy. The fetal trachea and esophagus were compressed by the goiter. Cordocentesis was performed at 34 weeks of gestation and thyroid-stimulating hormone (TSH), free triiodothyronine (fT3), and free thyroxine (fT4) levels in the fetal blood were 97.8 μ IU/mL, 2.52 pg/mL, and 0.47 ng/dL, respectively. Maternal propylthiouracil was discontinued and LT-4 300 μ g was administered into the amniotic fluid at 35 and 36 weeks of gestation, respectively. The fetal goiter reduced in size, the trachea and esophagus lumens increased, and the amniotic fluid index decreased. A female infant weighing 3224 g was delivered by elective cesarean section at 37 weeks of gestation. The infant displayed no respiratory distress at birth and her thyroid gland was not palpable. Her thyroid function at birth was 42.45μIU/mL TSH, 1.45 pg/mL fT3, and 1.39 ng/dL fT4, and her TSH level had decreased to within the normal range by 10 days after birth. We predicted that transplacental TSAb could have damaged the infant’s thyroid function, but she remained asymptomatic and was discharged at 17 days postpartum. Her growth and development were normal at last follow-up at 2 years old. We considered that this fetal goitrous hypothyroidism could have been caused by the following: (1) excessive fetal thyroglobulin production as a result of transplacental TSAb; (2) excessive fetal thyroid function caused by maternal thyroid hormone deficiency and (3) transplacental passage of maternal PTU; or (4) other reasons, e.g., iodide overload, iodide deficiency, and congenitally abnormal synthesis of thyroid hormone. Abnormalities of fetal thyroid function have been reported to cause mental retardation, and fetal goiters and thyroid function should thus be assessed and treated appropriately.

**Consent for publication:** The authors declare that written informed consent was obtained for publication.

## P2-5-8 Co-existing hypoparathyroidism and Graves’ disease in a girl with previously undiagnosed DiGeorge syndrome

### Wai Chun Wong, Chi Tak Tong

#### Department of Paediatrics, Alice Ho Miu Ling Nethersole Hospital

**Background**: Endocrinopathies are common in patients with DiGeorge syndrome. While hypocalcemia is one of the cardinal features of DiGeorge syndrome, autoimmune thyroid problem such as Graves’ disease has been sporadically reported. However, co-existing pathology of hyperthyroidism and hypoparathyroidism in the patient can sometimes obscure the problem of hypocalcemia, and may delay the diagnosis of DiGeorge syndrome.

**Case presentation:** A 14-year-old girl was first referred for management of hyperthyroidism. She presented with palpitation and weight loss for few months. The girl was noted to have diffuse goitre with physical signs of thyrotoxicosis including sinus tachycardia and hand tremor. She also had prominent forehead and hypertelorism, which were overlooked at the first visit. Biochemically, she had elevated FT4 (>100 pmol/L; reference 12-22 pmol/L) and undetectable TSH. Anti-thyroglobulin titre and anti-thyroid microsomal titre were elevated. Her serum calcium (2.23 mmol/L; reference 2.15-2.55 mmol/L) was normal at that time. The girl was diagnosed to have Graves’ disease and was started on carbimazole. After medical treatment for a few months, her symptoms of thyrotoxicosis were gradually resolved and her FT4 was normalized (17.3 pmol/L). However, concomitantly, the girl was found to have asymptomatic hypocalcemia. Her serum calcium dropped significantly when her thyroid problem underwent good control by carbimazole. While she had normal thyroid function, her serum calcium was decreased to 1.7 mmol/L, with a low ionized calcium. Further investigations for hypocalcemia showed normal 25OH vitamin D level, normal magnesium and low urinary calcium to creatinine ratio. Parathyroid hormone level (4.0 pmol/L; reference 1.6-6.9 pmol/L) was inappropriate normal in the presence of hypocalcemia. A diagnosis of hypoparathyroidism was established. In view of her hypoparathyroidism and subtle facial dysmorphism, the possibility of DiGeorge syndrome was highly suspected. Fluorescent in situ hybridization was performed and showed a microdeletion on one of the homologs 22q11.2. She was treated with calcitriol and calcium supplement with good response.

**Conclusion**: Autoimmune disease such as Graves’ disease is reported at an increased incidence in DiGeorge syndrome. This case report illustrated that early diagnosis of DiGeorge syndrome could be challenging in the presence of co-existing hyperthyroidism. In this patient, her underlying hypoparathyroidism was undetected by the possible hypercalcemic effect of hyperthyroidism. Treatment of the Graves’ disease allowed the problem of hypocalcemia to be identified, followed by confirmation of the diagnosis of DiGeorge syndrome.

**Consent for publication:** The authors declare that written informed consent was obtained for publication.

## P2-5-9 Method- and evidence-based reference intervals for interpretation of thyroid function in the first 168 hours of life

### Philip B Bergman^1,2^, Zhong Lu^3,4^, Michelle Jayasuriya^5^, Kay Weng Choy^3^, James Doery^3,4^, Kim Lit Chin^1,2^

#### ^1^Department of Paediatric Endocrinology & Diabetes, Monash Children's Hospital; ^2^Department of Paediatrics, Monash University; ^3^Monash Health, Pathology Department; ^4^Department of Medicine, Monash University; ^5^Monash University, School of Clinical Sciences

**Introduction**: Prompt intervention can prevent permanent adverse neurological effects caused by neonatal hypothyroidism. Thyroid function changes rapidly in the first few days of life but age-related reference intervals (RIs) for TSH, FT4 and FT3 are not available to aid interpretation. This is further complicated by the imprecision of how the days of life are classified, e.g., a neonate who was born just before midnight would be classified as day-1 a few minutes after midnight rather than day-0. We have developed hour-based RIs using data mining.

**Methods**: All TSH, FT4 and FT3 results (Beckman) with date and time of collection from neonates aged

**Results**: Of the 728 neonates qualifying, 569 had time of birth available. All 569 had TSH, 415 had FT4 and 146 had FT3 results. For age ≤24h, 24.1-48.0h, 48.1-72.0h, 72.1-120.0h and 120.0-168.0h of life, the TSH RIs (2.5th-97.5th) (mIU/L) were 4.1-40.2, 3.2-29.6, 2.6-17.3, 1.0-10.3 and 1.0-8.2 respectively; the FT4 RIs (mean ± 2SD) (pmol/L) were 15.3-43.6, 14.7-53.2, 16.5-45.5, 17.8-39.4, 15.3-32.1, 14.5-32.6, 13.9-30.9 and 14.4-28.6 respectively; and the FT3 RIs (mean ± 2SD) (pmol/L) were 5.0-9.4, 4.1-9.0, 2.8-7.8, 3.5-7.2, 3.4-8.0, 3.8-7.9 and 3.8-7.2 respectively.

**Conclusion**: In most babies, there is a substantial surge in TSH and FT4 shortly after birth followed by a rapid decline over the subsequent 168 hours. The upper limits are manyfold higher than the values for adults. Use of method- and hour-based RIs in newborns allows for correct identification of neonates who are at risk of hypothyroidism. Due to current lack of analytical harmonisation, these RIs must necessarily be method-specific.

## P2-5-10 Clinical characteristics of ectopic thyroid in South-Indian children

### Saidalikutty Fouzeamol, Palany Raghupathy, Ahila Ayyavoo

#### Department of Pediatric Endocrinology, G. Kuppuswamy Naidu Memorial Hospital

**Introduction**: The prevalence of congenital hypothyroidism in India is 1:500 to 1:1221. Ectopic thyroid(ET) accounts for 49-61% of thyroid dysgenesis. Prevalence of ET in the population is 1 per 1,00,000 - 3,00,000. As there is minimal information on the spectrum of ET in South-Indian children, we analysed the clinical characteristics amongst children presenting at a tertiary care hospital.

**Setting**: Outpatient department of Pediatric Endocrinology, GKNM Hospital, Coimbatore, India.

**Methodology**: Retrospective record review of all patients diagnosed with ectopic thyroid between January 2005 & March 2016 has been done. Details extracted are clinical presentation, age at diagnosis, thyroid hormonal profile, visibility of the gland at ectopic position, nuclear imaging findings and management. Aetiological diagnosis is done by scintigraphy before initiation of thyroxine. A four week period of hormone withdrawal at 3 years of age is followed by radionuclide scan in children treated with thyroxine without etiological diagnosis in early infancy. Ultrasonography has not been used due to poor sensitivity and specificity in the diagnosis of ET as compared to scintigraphy.

**Results and discussion**: Amongst 52 children with ectopic thyroid, 27 were girls. Mean age at presentation is 3.3 years (range 2 days to 12.5 years). 11.5% are diagnosed after 10 years of age while 23% are identified in the neonatal period. Radionuclide scan revealed tracer uptake in the lingual region in 44 of 52 (85%) children and in the upper part of the neck in others (15%). Two children have dual ectopic thyroid gland. Rare combinations include lingual thyroid+generalized resistance to thyroid hormone and lingual thyroid+Turner syndrome. Predominant symptom at presentation is developmental delay in 14 of 52 children. Oyher symptoms include short stature, neck swelling and prolonged neonatal jaundice in 7, 6 and 8 children respectively. Physician’s examination revealed lingual thyroid as a posterior-tongue mass in two patients. The average age at diagnosis is 7.3 years for children who presented with short stature which is later than any other symptom of presentation. 24 of 52 children (46%) had TSH above 60 miu/L at diagnosis. 10 out of 14 (71%) children with developmental delay have an initial TSH above 60 miu/L. The neck masses and externally visible lingual thyroids regressed with oral thyroxine replacement.

**Conclusions**: Ectopic thyroid gland should be considered while evaluating any asymptomatic child with a posterior tongue mass or midline mass in the neck. Prompt thyroxine replacement prevents surgical excision.

## P2-5-11 Predictors of transient congenital hypothyroidism in children with eutopic thyroid gland

### Hae Sang Lee^1^, Hwal Rim Jeong^2^, Jong Seo Yoon^1^, Cheol Hwang So^1^

#### ^1^Pediatrics, Ajou University School of Medicine; ^2^Pediatrics, Masan Medical Center

**Background and aims**: Congenital hypothyroism (CH) is the most common cause of preventable mental retardation. Recently, detection of CH with eutopic thyroid gland has increased through neonatal screening program. In this study, we aimed to evaluate the predicting factors to distinguish between permanent and transient CH in patients with a eutopic thyroid gland.

**Methods**: We retrospectively reviewed 121 children who were diagnosed with CH and eutopic thyroid gland. All subjects were treated with L-thyroxine and underwent re-evaluation after 3 years old of age.

**Results**: Of the 121 CH patients, 24 (19.8%) children were evaluated as having permanent CH (PCH) and 97 (80.2%) children showed transient CH (TCH). Initial free T4 and TSH level were significantly higher in TCH than in PCH subjects. Also, the mean doses of L-thyroxine (ug/kg/day) at 24 months and 36 months were significantly lower in TCH than in PCH subjects with a eutopic thyroid gland during a 3-year treatment period. Based on the receiver operating characteristic (ROC) curve, the optimal cut point of L-thyroxine dose at 2 and 3 years were 3.2 and 2.7 ug/kg day in predicting TCH, which was associated with an 83.5% sensitivity and 69.6% specificity at 2 years and 92.8% sensitivity and 58.3% specificity at 3 years, respectively.

**Conclusion**: The L-thyroxine requirements during treatment period have a predictive role for differentiate TCH from PCH in the patients with eutopic thyroid gland.

## P2-5-12 Clinical Profile and Outcome of Children with Graves Disease Seen at the Out-Patient Department of a Tertiary Pediatric Hospital from 2003-2013

### Katrine Anne R De Lara^2^, Lorna R Abad^1^

#### ^1^Pediatric Endocrinology, Philippine Children's Medical Center, Section Head; ^2^Las Pinas General Hospital and Satellite Trauma Center, ISO Office

**Background**: Graves disease is one of the most common causes of thyrotoxicosis in children that is characterized by hyperthyroidism, diffuse goiter, ophthalmopathy and rarely dermopathy.

**Objectives**: This study presented the clinical profile of patients diagnosed with Graves disease seen at the out-patient department of a tertiary pediatric hospital from 2003-2013. It described the demographic data of the patients, the signs and symptoms at initial consult, the thyroid function tests at initial and final consult, the treatment options taken: medical with antithyroid drugs, surgical or radioactive iodine ablation and determined the remission rate of patients after medical therapy.

**Study Design**: Retrospective study of forty two patients with Graves disease diagnosed from 2003-2013. Data from patient charts were extracted from the out-patient department of a tertiary pediatric institution.

**Results**: Forty two patients with Graves disease from 2003-2013 were included, thirty five females and seven males. A positive family history for thyroid disease was seen in twenty six percent. Initial presentation included goiter, neck mass, weakness, increased sweating, and palpitations. Medical treatment with Methimazole was the initial treatment in twenty eight patients (67%). Only nine patients went into remission (21.42%).

**Conclusion**: Hyperthyroidism is more common in females. Signs and symptoms were comparable with local and international data. Remission rate of twenty one percent approximates that of published reports.

**Recommendations**: A large scale multicenter study should be done to compare remission rates of different institutions managing patients with Graves disease. A study on factors affecting remission is recommended.

**Keywords**: Graves disease, Methimazole, Propylthiouracil, Radioactive Iodine Ablation, Remission

## P2-5-13 Slipped capital femoral epiphysis (SCFE) associated with hypothyroidism: a case report of hypothyroidism accompanied by SCFE occurring before puberty and literature review

### Tomohiro Hori^1^, Saori Kadowaki^1^, Hideki Matsumoto^1^, Akifumi Nozawa^1^, Kaori Kanda^1^, Michio

### Ozeki^1^, Norio Kawamoto^1^, Yu Shirakami^2^, Kenji Orii^1^, Zenichiro Kato^1^, Toshiyuki Fukao^1^

#### ^1^Department of Pediatrics, Graduate School of Medicine, Gifu University; ^2^Department of Pediatrics, Gifu Prefectural Kibogaoka Medical and Support Center for Children

Slipped capital femoral epiphysis (SCFE) is a rare disease. However, it is a very important complication of hypothyroidism occurring in childhood.

The patient was an 8-year-old boy with a pain in the hip joint. Hip joint radiography and MRI scans diagnosed him with chronic bilateral SCFE. He was short stature and his growth had slowed down for 4 years from around age 4. Hirsutism, dry skin and bradycardia were noted. Decreased thyroid function was observed (FT4 0.10 ng/dl, TSH 1,789 μ IU/ml) and thyroid autoantibodies were positive (TPOAb >600 IU/ ml, TgAb 1,720 IU/ml). Thyroid ultrasonography showed marked atrophy of the thyroid. Thus, a diagnosis of atrophic thyroiditis was made. For the treatment of SCFE, the patient was admitted and kept at rest with traction of the lower limbs. Atrophic thyroiditis was treated with oral levothyroxine therapy beginning at a low dose followed by gradual dose increase. The FT4 level was improved within the normal range about 2 months later. Surgery was performed for bilateral SCFE.

We review the literature reporting the patients with SCFE associated with hypothyroidism. SCFE associated with hypothyroidism is rare; 41 cases were found from 24 reports according to our search of literature published from 1980 to present. We analyzed the clinical features in 42 cases comprising these reported cases and our case. Our present study that compared general SCFE and SCFE associated with hypothyroidism revealed the following characteristic features of the latter: 1) the age at onset is mostly early teens, the same as in cases of general SCFE, but the onset is occasionally seen before or after puberty and in adulthood, 2) non-obese patients are relatively frequent, and 3) short stature is frequent. In particular, the occurrence of SCFE in patients aged 16 years or older, who normally have closed epiphysis, cannot be explained by the mechanisms of general SCFE and suggests the presence of hypothyroidism. Moreover, as in our present case, the presence of hypothyroidism should also be suspected in cases of SCFE occurring in prepubertal patients aged 8 years or younger. Close examination of thyroid function should be conducted aggressively in patients with SCFE who are not in their early teens, are non-obese, or have short stature or decreased growth rate.

**Consent for publication:** The authors declare that written informed consent was obtained for publication.

## P2-5-14 Functional status of thyroid gland in hospitalized children with down's syndrome

### Rabi Biswas^1^, Abu Syed Munsi^2^, Manzoor Hussain^2^, Rafiqul Islam^1^, A.S.M. Nawshad Uddin Ahmed^1^

#### ^1^Dhaka Shishu (Children) Hospital, Shere Bangla Nagar, Dhaka, Bangladesh, Department of Paediatric Endocrinology and Metabolic Disorders; ^2^Department of Paediatric Cardiology, Dhaka Shishu (Children) Hospital, Shere Bangla Nagar

**Background**: Over one-hundred and thirty years ago a link between Down’s syndrome and thyroid disease had been proposed. The additive effects of this co-morbid condition leads to further amplification of the clinical problems in these children with Down’s syndrome. While several international studies have shown association of thyroid dysfunction with Down’s syndrome, there is paucity of data available from Bangladesh.

**Objective**: The purpose of this study was to know the prevalence of thyroid dysfunction in Down’s syndrome children in our setting and to correlate the features of Down’s syndrome with those of thyroid dysfunction.

**Methods**: A cross-sectional study was carried out with 40 patients of Down’s syndrome admitted at Dhaka Shishu Hospital from January 2014 to March 2015. Diagnosis of Down’s syndrome was considered clinically with or without support of karyotyping. Thyroid function was assessed by serum FT4 and TSH.

**Results**: All 40 patients enrolled were below 5 years and most of them (72.5%) were within 12 months of age. Twenty-one cases were male and 19 female. Hypothyroidism was found in 6cases (15.0%), of which 4 (10%) had compensated hypothyroidism. Hyperthyroidism was not observed in any of the cases. There was no significant sex difference.

**Conclusion**: Hypothyroidism in children with Down’s syndrome is prevalent in our setting. It thus seems necessary to screen all Down’s syndrome children for thyroid dysfunction.

**Key words**: Down’s syndrome, hypothyroidism.

## P2-5-15 Hashimoto's thyroiditis manifested by exposure to iodinated contrast media: a case report

### Miyuki Kitamura, Shuichi Yatsuga, Junko Nishioka, Takako Sasaki, Yasutoshi Koga

#### Department of Pediatrics and Child Health, Kurume University School of Medicine

**Introduction**: Although Hashimoto’s thyroiditis is the most common cause of acquired hypothyroidism, many patients are asymptomatically euthyroid without medications. Iodinated contrast media ( ICM ) is often used as a contrast agent in imaging studies. Exposure to transient excess iodine does not usually induce hypothyroidism, because it is generally tolerated well. We report a patient with asymptomatic Hashimoto’s thyroiditis, who developed symptomatically hypothyroidism after exposure to ICM.

**Case report**: Seventeen-year-old girl was diagnosed as having pulmonary arteriovenous fistulas at 8 years old. She has received many cardiac catheterization procedures at the age of 8 to 14 years old, using ICM, and has been no symptoms until the age of 17 years. Since she showed low oxygen saturation at 17 years old, she received cardiac catheterization and coil embolization again. General fatigue and headache were immediately appeared at the same day of last cardiac catheterization. After discharge, she complained severe general fatigue and headache, and finally she could not walk alone. She showed 39 bpm in heart, and 67/37 mmHg in blood pressure. Free T4 (fT4) level was 0.59 ng/dL, and TSH level was 1.39 μIU/mL. Anti-thyroglobulin antibody (TgAb), 122.3U/mL (>28U/mL) and anti-thyroid peroxidase antibody (TPOAb), 20.9U/mL (>16U/mL) were both positive. She was diagnosed with Hashimoto’s thyroiditis. To rule out brain abscess and/or tumor, she received enhanced CT with ICM and had negative findings. She was referred to our hospital for treatment of Hashimoto’s thyroiditis. She was continued for goitor, hair loss and general fatigue. In our hospital, fT4 level was 0.89 ng/dL (lower limit of the normal range), TSH level was 0.87 μIU/mL. TgAb and TPOAb were continuously positive. FT4 level was still slightly low, then, we treated low dose of levothyroxine for the therapeutic purpose of hypothyroidism. Her symptoms were improved after the levothyroxine treatment. To date, no symptoms of hypothyroidism exist with low dose of levothyroxine treatment.

**Discussion**: Production of thyroid hormone depends on adequate iodine intake. Wolff-Chaikoff effect is known that ultra-excessive iodine reduces production of thyroid . Individuals with underlying thyroid disease sometime develop hyper- or hypothyroidism after excess iodine exposure. She may be oligosymptomatic carrier of Hashimoto’s thyroiditis around 17 years old, however her thyroid status changed to be symptomatic hypothyroidism after exposure to ICM. Mechanism of iodine-induced thyroid dysfunction is uncertain.

**Conclusions**: Individuals who have an antibodies associated with Hashimoto’s thyroiditis may be at risk for hypothyroidism after exposure to ICM.

**Consent for publication:** The authors declare that written informed consent was obtained for publication.

## P2-6-1 Study on polymorphisms of estrogen receptor-βgene RasI and AluI in the girls with idiopathic central precocious puberty

### Guoqing Dong, Mingzhu Li

#### Department of Pediatrics, Shenzhen Maternal and Child Healthcare Hospital affiliated to the Southern Medical University

**Objective**: To explore the Polymorphisms of estrogen receptor-β(ERβ) gene RasI and AluI sites in the girls with idiopathic central precocious puberty(ICPP).

**Methods:** F RasI and AluI sites in ERβ gene were detected by direct DNA sequencing in 100 girls with ICPP and 100 healthy girls, And the frequency distribution of genotypes and alleles of RasI and AluI were statistically analyzed between the ICPP group and the control.

**Results**: (1) There was statistically significant difference in the genotype(RR, Rr, rr) frequency of RasI site between ICPP group and control group iχ2 = 5.960, *P* =0.045 ), And the risk of ICPP in girls with allele R was 1.579 fold of that with allele r i95%CI F1.047 `2.382, *P* <0.05 ). (2) There was no statistically significant difference in the genotype(AA, Aa, aa) frequency of AluI site between ICPP group and the control iχ2 =2.889, *P* =0.236), the risk of ICPP in girls with allele A was 1.589 fold of that with allele a i95%CI F0.916 `2.755 CP >0.05 ).

**Conclusions**: The polymorphisms of RasI site but AluI site in ERβgene may be related to ICPP, And Allele R may be a risk factor of ICPP for girls.

## P2-6-2 Final height outcome of girls with idiopathic central precocious puberty treated with gonadotropin releasing hormone agonist: A longitudinal retrospective study from northeastern Thailand

### Pattara Wiromrat, Ouyporn Panamonta

#### Division of Endocrinology, Department of Pediatrics, Faculty of Medicine, Khon Kaen University

**Background**: Idiopathic central precocious puberty (ICPP) is a common endocrine disorder. Gonadotropin releasing hormone agonist (GnRHa) is the standard treatment to preserve adult height and menstrual cessation. However, the height outcomes varied across studies.

**Objective**: To evaluate the final adult height of Thai girls with ICPP who received GnRHa treatment.

**Methods**: In this longitudinal retrospective study, medical records of 45 girls who were diagnosed as ICPP and treated with GnRHa during 2000-2016 were reviewed. All girls received a fixed 3.75 mg dose of GnRHa subcutaneously every 4 weeks. Predicted adult height (PAH) was calculated according to Bayley and Pinneau method from initial bone age (BA). Their heights, weights and Tanner stages were followed every 3 months until they reached final adult height (FAH).

**Results**: Mean age of thelarche was 6.9 ± 0.6 years. Twenty-seven girls presented with menstrual bleeding at mean age of 8.4 ± 0.8 years. GnRHa was started at 8.5 ± 0.8 years with mean duration of 3.3 ± 1 years. Thirty-one and fourteen patients received leuprolide acetate and triptorelin depot respectively. Leuprolide was switched to triptorelin in two patients due to pubertal progression. Mean FAH was 157.4 ± 5.7cm which significantly increased from PAH (153.4 ± 6.8 cm, p<0.01) and target height (TH) (155.3 ± 4.4 cm, p<0.01). Subgroup analysis showed the PAH in girls who presented with and without menstruation were not different at baseline (153.3 ± 6.8 vs 153.3 ± 7.1 cm, p=0.9) although girls with menstruation had more advanced BA (12.5 ± 0.6 vs 11 ± 0.6 years, p<0.01). But after treatment, girls presented with menstruation had significantly decreased FAH compared with those who had not (156 ± 5.7 vs 160 ± 4.9 cm, p=0.01). However, FAH was increased significantly from PAH in girls presented with menstruation.

**Conclusion**: GnRHa treatment at the usual dose of 3.75 mg subcutaneously every 4 weeks could effectively improve final adult height in Thai girls with ICPP. Modest benefit could also be observed even in girls presented with menstruation.

## P2-6-3 A novel MKRN3 mutation in sporadic central precocious puberty: a first case in a Japanese girl

### Junko Nishioka^1^, Hirohito Shima^2^, Maki Fukami^2^, Shuichi Yatsuga^1^, Takako Sasaki^1^, Kikumi Ushijima^1,2^, Miyuki Kitamura^1^, Yasutoshi Koga^1^

#### ^1^Department of Pediatrics and Child Health, Kurume University School of Medicine; ^2^Department of Molecular Endocrinology, National Research Institute for Child Health and Development

## P2-6-4 Structural and functional cardiac alterations in ovarian NGF overexpressing mice

### Maria C Garcia Rudaz^1^, Jenny E Wilson^2^, Ekaterina Salimova^5^, Greg Dissen^3^, Sergio R Ojeda^3^, Chrishan S Samuel^4^, Nadia Rosenthal^5^, Michael A Cowley^2^, Pablo J Enriori^2^

#### ^1^Department of Pediatrics, Australian National University, Women, Youth & Children Hospital; ^2^Department of Physiology, Monash University; ^3^Department of Neurosciences, Oregon Health and Science University; ^4^Department of Pharmacology, Monash University; ^5^Monash University, Australian Regenerative Medicine Institute

PCOS, the most common female endocrine disorder of unknown etiology is characterized by reproductive abnormalities and associated metabolic conditions. PCOS has also been associated with an increase in atherosclerotic disease and endothelial dysfunction and an increased prevalence of several cardiovascular risk factors including hypertension. We have shown that transgenic overexpression of NGF, a marker of sympathetic hyperactivity, directed to the ovary by the mouse 17 α-hydroxylase promoter (17NF mice), resulted in ovarian abnormalities and associated metabolic perturbations including insulin resistance and hyperinsulinemia similar to those seen in women with PCOS. 17NF mice also display an increase in body weight and abnormal body fat distribution. Importantly, overproduction of NGF leads to systemic sympathetic hyperactivity in these mice. Here, we sought to determine whether 17NF mice exhibit associated cardiovascular alterations. We evaluated left ventricular (LV) structure and function through the use of echocardiography. Several structural and functional cardiac alterations were observed in 17NF mice compared to WT mice including decreased LV mass to body weight ratio as a result of a thinner LV posterior wall and shorter longitudinal heart axis. Additionally, likely due to LV wall thinning, LV area and volume was enlarged in 17NF mice. Moreover, despite increased left ventricular volume, stroke volume was similar in both groups of mice suggesting compromised cardiac function. In support of this, ejection fraction was also found to be slightly decreased in 17NF mice. Furthermore, decreased systolic-diastolic changes in the area of the left ventricle and LV posterior wall dimensions in 17NF mice suggest compromised LV contractility. To determine whether cardiac remodeling observed in 17NF mice was associated with alterations in myocardial collagen content, LV interstitial myocardial collagen fraction was assessed by analysis of picrosirus red stained LV sections. Interstitial myocardial collagen fraction was found to be approximately 30% lower in 17NF mice compared to WT mice at 10 weeks of age and 20 weeks of age. We also measured blood pressure by tail cuff recording in body weight-matched mice. A significant increase in Systolic BP (p<0.05) was detected across the 10-week time period, in 17NF mice compared to WT mice and at the time points 17 and 17.5 weeks of age. Altogether, these results suggest an overexpression of NGF in the ovary is sufficient to cause reproductive, metabolic and cardiovascular alterations of PCOS and are in support of the hypothesis that sympathetic hyperactivity may play a pathological role in the development and/or progression of PCOS.

## P2-6-5 Gonadotropin-inhibitory hormone (GnIH) is a novel mediator between the hypothalamic-pituitary-gonadal (HPG) and hypothalamic-pituitary-thyroid (HPT) axes

### Mika Kiyoara^1^, You Lee Son^2^, Ichiro Miyata^1^, Kazuyoshi Tsutsui^2^

#### ^1^Department of Pediatrics, The Jikei University School of Medicine; ^2^Department of Biology, Waseda University, and Center for Medical Life Science of Waseda University

**Background**: Thyroid disorders affect the timing of puberty, such as precocious puberty or delayed puberty. Although the cross-talk between the hypothalamic-pituitary-gonadal (HPG) and hypothalamic-pituitary-thyroid (HPT) axes has been suggested in the reproductive regulation by thyroid hormones, the mechanisms involved in the abnormal puberty by thyroid disorders have not been fully elucidated. Gonadotropin- inhibitory hormone (GnIH) is a hypothalamic neuropeptide inhibiting gonadotropin secretion by decreasing the activity of gonadotropin- releasing hormone (GnRH) neurons as well as directly acting on gonadotropes, indicating that GnIH is a key factor of the HPG axis. Therefore, we investigated the possible role of GnIH as a mediator between the HPG and HPT axes involved in the regulation of puberty onset by thyroid status.

**Methods**: First, we investigated the effect of thyroid status on puberty onset and GnIH expression. Hypothyroidism and hyperthyroidism were induced in female mice at postnatal days 20 by 2-weeks administration of propylthiouracil (PTU) and thyroxine (T4), respectively. Second, we examined the effect of thyroid hormone (triiodothyronine, T3) on GnIH mRNA expression in hypothalamic explants. Finally, we investigated the expression of thyroid hormone receptors (TRs) in GnIH neurons by immunohistochemistry combined with in situ hybridization.

**Results**: Female mice treated with PTU were induced to become hypothyroidism evidenced by the lower T3 and elevated TSH levels, and loss of weight. Hypothyroidism mice showed delayed pubertal onset and increased GnIH mRNA expression. These results imply that increased GnIH expression may inhibit the HPG axis, thus puberty onset is delayed. In contrast, T4-treated hyperthyroidism mice, which determined by elevated T3 and lower TSH levels, and gain of weight, showed no significant difference in the puberty onset. Although elevated T3 levels did not affect puberty onset in hyperthyroidism mice, GnIH expression was reduced by less than 50%, indicating that thyroid status could regulate GnIH expression. Supporting our hypothesis, T3 treatment decreased GnIH mRNA levels in hypothalamic explants. Furthermore, we showed that GnIH neurons express both TR α and TR β , indicating that thyroid hormone could directly act on GnIH neurons via TRs.

**Conclusions**: We demonstrated that thyroid status alters GnIH mRNA expression in vivo and in vitro , and GnIH neurons express TRs, indicating that the effect of thyroid hormone on GnIH expression is mediated by TRs. Therefore, we suggest that GnIH is concerned in the regulation of reproduction by thyroid hormone by mediating cross-talk between the HPG and HPT axes.

## P2-6-6 rFSH monotherapy prior to hCG-rFSH combination therapy is an effective new treatment to achieving future fertility in adolescent patients with congenital male hypogonadotropic hypogonadism

### Naoko Sato^1,2^, Akiko Hosokawa^3^, Sachiko Kitanaka^1^, Atsuko Yoshizawa^2^, Masahiro Noda^2^, Toshiaki Tanaka^2^

#### ^1^Department of Pediatrics, Tokyo University; ^2^Tanaka Growth Clinic; ^3^Hikari Clinic

**Background:** Congenital male hypogonadotropic hypogonadism (CMHH), a disorder associated with infertility, is treated with hCG-rFSH combination therapy (hCG-rFSH) to achieve fertility. However, approximately 60% of CMHH could not achieve sufficient spermatogenesis with the current hCG-rFSH (Sato et al.). Dywer et al. reported on the effectiveness of rFSH mono-therapy followed by hCG-rFSH to improve spermatogenesis in adult patients with CMHH with small testis. But there is no report of this treatment in adolescent patients with CMHH (ADCMHH).

**Objective:** With the aim of improving spermatogenesis of ADCMHH, new methods are to be proposed and effectiveness for spermatogenesis is discussed.

Proposal for new treatment: After rFSH (75IU) for two months, hCG-rFSH is administered in appropriate therapeutic dose according to age.

**Case 1**: 18-year-old male: He was diagnosed as Kallmann syndrome (KS) because of micropenis and hyposmia in infancy and identified with KAL1 mutation. From the age of 12 years, low dose testosterone was administered. At the age of 16, treatment was changed to rFSH mono-therapy for 4 months. From the age of 17 to the present, hCG-rFSH has been continued. After two months of switching to rFSH-hCG, testosterone reached adult level and testicular volume was increased to double. Sperm count was confirmed by semen test at the age of 18.

**Case 2**: 21-year-old male: At the age of 18, He was diagnosed as KS because of cryptorchidism, delayed puberty and hyposmia. rFSH mono- therapy was administered daily for 60 days, then switched to hCG-rFSH. Testis volume was less than 1 ml when treatment began and increased to 5 ml in two years. Testosterone reached adult level. Nocturnal emission was confirmed.

**Conclusion**: rFSH mono-therapy prior to hCG-rFSH was used to treat ADCMHH with small testis. Enlargement of testis and spermatogenesis were recognized. This result suggests the effectiveness of this new method to achieve future fertility in ADCMHH.

**Consent for publication:** The authors declare that written informed consent was obtained for publication.

## P2-6-7 The various phenotypes of ten CHARGE syndrome cases with identified pathogenic CHD7 mutations

### Kohei Aoyama^1^, Haruo Mizuno^1^, Tatsushi Tanaka^1^, Atsushi Suzuki^1^, Mie Inaba^2^, Seiji Mizuno^2^, Shinji Saitoh^1^

#### ^1^Department of Pediatrics and Neonatology, Nagoya City University Graduate School of Medical Sciences; ^2^Department of Pediatrics, Aichi Prefectural Colony Central Hospital

**Background/Aims**: CHARGE syndrome is an autosomal dominant genetic condition characterized by various features such as coloboma, heart malformation, atresia of the choanae, retardation of growth or development, genital anomalies, and ear malformation. In 2004, CHD7 was identified as the gene responsible for CHARGE syndrome. Although several diagnostic criteria and genotype-phenotype studies have been reported for CHARGE syndrome, the complex phenotypic spectrum associated with pathogenic CHD7 mutations has not been well understood. The aim of this study was to gain a greater understanding of various phenotypes of CHARGE syndrome identified by pathogenic CHD7 mutation.

**Methods**: We enrolled 17 cases with clinically suspected CHARGE syndrome diagnoses. All coding exons of CHD7 of all the cases were sequenced using an Ion PGM system with the Ion Ampliseq Custom Panel for hypogonadotropic hypogonadism (including CHD7 ). We identified ten cases with pathogenic CHD7 mutations and extracted phenotypes from medical records or clinical information collected from the primary pediatrician. In cases harboring a previously reported mutation, we compared our case with the published clinical information.

**Results**: We identified five nonsense, three frame-shift, and one missense mutation in ten CHARGE syndrome cases. The same nonsense mutation was identified in two unrelated cases. Of the nine CHD7 mutations, six were novel. At the time of the study, the mean patient age was 5.8 years (1 month to 12.6 years) and nine of the ten patients were male. Seven male patients had cryptorchidism and/or micropenis but two male patients did not have either phenotype as an endocrinological finding. A male patient harboring a CHD7 nonsense mutation, who had double outlet right ventricle (DORV) and esophageal atresia, died at the age of 1 year 9 months owing to sudden cardiorespiratory insufficiency of unknown cause. Another infant with the same CHD7 mutation as the deceased had no congenital heart disease and had several different phenotypes. The other cases displayed some phenotypic differences from those previously reported in cases with the same CHD7 mutation. We could not find a clear phenotype trend by type of mutation.

**Conclusion**: This study shows that there is a range of phenotypes within the diagnosis of CHARGE syndrome, even in individuals with the same CHD7 mutation. Our results indicate that the type and position of CHD7 mutation alone are not sufficient to determine CHARGE phenotype. Other elements, such as environmental factors during the fetal period, might modify the CHARGE syndrome phenotype.

## P2-6-8 Physical assessment and reference growth curves for children with 46, XY disorders of sex development

### Di Wu^1^, Chunxiu Gong^1^, Hui Chen^2^

#### ^1^Department of Endocrinology, Genetics and Metabolism, Beijing Children's Hospital, Capital Medical University; ^2^Capital Medical University, School of Biomedical Engineering

**Objective**: In addition to the abnormalities pertaining to sexual development, impaired growth and development is another important issue to be considered in disorders of sex development (DSD) patients. This study is to profile the growth and development of children with 46, XY DSD.

**Methods**: Cases of 46, XY DSD aged between 0-16 years were sourced from the hospital registration database. Height differences were compared between DSD and normal boys. Growth curves for DSD were obtained by LMS method.

**Results**: 571 cases of non-CAH 46, XY DSD were included in the study. The HtSDS of study subjects were -0.031 ± 1.202. 304 cases showed good response with HCG standard test, with HtSDS of 0.082 ± 1.212. 123 cases showed a poor response, with HtSDS of -0.240 ± 1.090. The HtSDS of poor response with HCG test group was generally lower than that of normal boys (P=0.017). In children aged 12 years or older in both groups, HtSDS were significantly lower than normal boys. HtSDS of both groups were lower than average height of normal boy among multiple age groups since early infancy. A growth lag in study subjects was particularly discernible in the 12-16 years age group, which tended to be more pronounced in the poor response group.

**Conclusion**: The growth retardation of children with non-CAH, 46, XY DSD was observed in early childhood and puberty. The level of retardation related with androgens, which suggests that the male hormone has an effect on the physical development of these children.

## P2-6-9 WAGR syndrome due to 46,XY,del(11)(p11.12p14.3) in a patient born to a mother with 46,XX,ins(13;11) (q21.2;p11.12p14.3)

### Shinichi Nakashima^1^, Satoshi Okada^2^, Fumiko Kato^1^, Kaori Yamoto^1^, Tsutomu Ogata^1^

#### ^1^Department of Pediatrics, Hamamatsu University School of Medichine; ^2^Department of Pediatrics, Hiroshima University Graduate School of Biomedical & Health Sciences

**Background**: WAGR (W ilms’ tumor, a niridia, g enitourinary anomalies, and mental r etardation) syndrome is rare genetic disorder caused by contiguous gene deletion at 11p, including WT1 involved in the development of Wilms’ tumor and urogenital abnormality and PAX6 relevant to the development of arniridia and mental retardation. Here, we report a patient with WAGR syndrome and 46,XY,del(11)(p11.12p14.3) who was born to a mother with 46,XX,ins(13;11)(q21.2;p11.12p14.3).

**Case report**: A 5-month-old Japanese phenotypic female presented with ambiguous genitalia, microphthalmia and cataract. Ultrasound examination showed uterus-like structure and failed to delineate gonads. MRI revealed hypoplasia of the corpus callosum and cerebellum. Cytogenetic analysis showed a 46,XY,del(11)(p11.12p14.3) karyotype in the patient and a 46,XX,ins(13;11)(q21.2;p11.12p14.3) karyotype in the phenotypically normal mother. Family history showed multiple abortions by the maternal grandmother and a single abortion by a maternal elder brother.

**aCGH analysis**: An approximately 27.2 Mb deletion on 11p including WT1 and PAX6 was detected in the patient, and no apparent deletion or duplication was found in the mother.

**Discussion**: The results indicates that the patient had WAGR phenotype because of the contiguous gene deletion at 11p. Notably, the interstitial deletion at 11p of this girl was caused by the balanced insertion of the mother, and the family history suggests that the insertion was derived from the grandmother and inherited to the maternal elder brother as well. Thus, careful genetic counseling is recommended in this family, with regard to the risk of the development of WAGR syndrome in the offsprings.

**Consent for publication:** The authors declare that written informed consent was obtained for publication.

## P2-6-10 Histological analyses of placenta identified a high incidence of placental dysfunction in IUGR-patients with hypospadias

### Yasuko Shoji^1^, Keiko Matsuoka^2^, Masanobu Kawai^1,3^, Shinsuke Onuma^1^, Mikiko Koizumi^1^, Mariko Nakacho^1^, Hiroyuki Yamada^1^, Yuri Etani^1^, Futoshi Matsui^4^, Kohji Yazawa^4^, Fumi Matsumoto^4^, Makoto Takeuchi^2^, Shinobu Ida^1^

#### ^1^Department of Pediatric Gastroenterology, Nutrition and Endocrinology, Osaka Medical Center And Research Institute for Maternal And Child Health; ^2^Department of Pathology, Osaka Medical Center And Research Institute for Maternal And Child Health; ^3^Department of Bone and Mineral Research, Osaka Medical Center And Research Institute for Maternal And Child Health; ^4^Department of Urology, Osaka Medical Center And Research Institute for Maternal And Child Health

**Object**: Hypospadias is a common congenital malformation in males and often observed in infants with intrauterine growth restriction (IUGR). Because placental insufficiency has been implicated in the pathogenesis of IUGR, we investigated the association between placental histological findings and clinical presentations in patients with hypospadias.

**Subject and method**: Thirty six boys born between January 2011 and September 2015 were retrospectively analyzed based on their medical records.

**Results**: Hypospadias are classified into three types based on the location of urethral meatus; glandular/graular and coronal/subcoronal type (type 1), distal (penile) type (type 2), proximal (peroscrotal, scrotal, perineal) type (type 3), and 17 patients (47.2%) were categorized into type 1, 4 patients (11.1%) in type 2 and 15 patients (41.7%) in type 3. Thirteen out of 28 IUGR patients were classified into type 3 hypospadias, whereas 2 out of 8 non-IUGR patients had type 3 hypospadias, suggesting that the percentage of severe hypospadias was greater in IUGR patients compared to non-IUGR patients. Hypospadias was observed more frequently in preterm-IUGR patients (94.1%) than term-IUGR patients (63.4%). Nine patients (25%) exhibited genitourinary complications including micropenis in 2, cryptorchidism in 5 and bifid scrotum in 2. Histological examinations revealed placental abnormalities such as low placental weight, ischemic changes, calcifications, villitis and degenerative changes in most of IUGR patients.

**Discussion**: Hypospadias is caused by the abnormal fusion of urethral folds during early fetal development, by the time of approximately 16-week of gestation. Placenta plays important roles in the development of external genitalia by stimulating the synthesis of fetal gonadal hormones through the production of placental hCG; therefore, the impaired placental function in IUGR patients may be responsible for the insufficient development of external genitalia and cause hypospadias.

**Conclusion**: Severe hypospadias are more frequently observed in IUGR patients, especially in case of preterm birth.

## P2-6-11 The effect of letrozole on the reproductive function and linear growth in the early and middle period of puberty boy

### Juan Lin, Mei Hua Ma

#### Department of Pediatrics, The Third Affiliated Hospital of Sun Yat-Sen University

**Objective**: To provide clinical data for the effect of letrozole on the reproductive function and linear growth in the early and middle period of puberty boy.

**Method**: 43 early and middle pubertal boy with seriously damaged predict adult height, treated with letrozole 1.5 mg/m2/d Po( ≯ 2.5mg/d) were enrolled as treatment group. 48 cases of healthy puberty boy(include boy with idiopathic short stature) were enrolled as control. According to letrozole treatment time, divide into short time group, mid-long time group, long time group, the control group as the match.

**Results**: After letrozole treatment, testicular volume and Genital stage of treatment group with short time and mid-long time group were significantly increased compared with the corresponding control group. The testicular volume and T in control group had a good positive correlation, as well in treatment group (r = 0.803, P < 0.001 and r = 0.835, P < 0.001).The testosterone levels in each group of treatment group were significantly higher than accordingly control group, as well as serum'sH, LH, LH/FSH level. While E2 level with long time group of treatment group significantly reduced than the control group. 17 cases of control group, 13 cases of treatment group had serum AMH, INHB level tested before and after intervention. The control group of serum AMH level had decreasing trend in puberty, blood testosterone values elevated gradually, and serum AMH and T show a significantly negative correlation (r = -0.466, p=0.059). While treatment group of serum AMH and T show significant positive correlation (r=0.586.p=0.035), differ from normal AMH change trend. Serum INHB level in two groups after intervention all had different level rise, and INHB in control group increase more than the treatment group, but there was no statistically significant difference. Children in long time group of treatment group had more evident bone age inhibition than control group.

**Conclusion**: The testicular volume change and testosterone levels changes had high consistency, suggesting it may clinical preliminary judge serum testosterone levels according to the size of testis. Letrozole treatment can obviously promote the secondary sex characters development in adolescent boys. The AMH change trend with letrozole treatment group differ from normal trend, prompt that it may have inhibitory effect on testis maturity. Letrozole treatment group with its serum INHB level had a little different with control group, it cannot deny that testis sertoli cells function had unaffected completely, further study needed. Children with long-term letrozole treatment had bone age delayed.

## P2-6-12 Tanner-stage specific paediatric ranges for gonadotropins

### Philip Bergman^3,5^, S McNeil^1^, P Kosanam^1^, Kay Weng Choy^4^, James Doery^2,4^, Justin Brown^3,5^

#### ^1^Monash University, Monash School of Medicine; ^2^Department of Medicine, Monash University; ^3^Department of Paediatrics, Monash University; ^4^Department of Biochemistry, Monash Medical Centre; ^5^Department of Paediatric Endocrinology & Diabetes, Monash Children's Hospital

**Aims**: Disorders of puberty are diagnosed and monitored by measurement of hormones of the hypothalamic-pituitary-gonadal (HPG) axis. As growth and development can influence normal circulating concentrations of these hormones, gender- and Tanner stage-specific gonadotropin ranges are more meaningful than age-specific values. In particular, given the pulsatile nature of gonadotropin release, are baseline luteinising hormone (LH) and follicle-stimulating hormone (FSH) levels using a sensitive assay able to distinguish between prepuberty and the onset of puberty?

**Methods**: LH and'sH results (measured on Beckman Coulter UniCel DxI 800 analyser) betweeen March 2011 and October 2015 were extracted from Monash Pathology laboratory information system. Patients were included where data on Tanner staging were available. Patients with primary gonadal failure and on treatments influencing the HPG axis were excluded. The remaining data were used to calculate gender- and Tanner stage-specific LH and'sH ranges (mean +/- 2SD).

**Results**: Of 724 records of paired LH and'sH measurements Tanner staging for 185 was available. See table below for gender- and Tanner stage-specific LH and'sH ranges (mean +/- 2SD).

**Conclusion**: Results from a cohort of healthy children and adolescents are ideal for calculation of paediatric reference intervals for gonadotropins. Nevertheless, our data from hospital outpatients (after exclusion of any intervention that might alter HPG axis) still provided a useful picture of gonadotropin levels across Tanner stages. More data points are required to establish reference ranges for routine clinical use. There is significant overlap between gonadotropin results between Tanner Stages I and II suggesting that diagnosing the onset of puberty is difficult using baseline measurements, particularly in females, suggesting the need for gonadotropin-releasing hormone-stimulated gonadotropins to assess maturity of the HPG axis.

## P2-6-13 The efficacy of intramuscular testosterone enanthate therapy for micropenis or microphalus is different among hypogonadotropic hypogonadism, hypergonadotropic hypogonadism, and other etiologies

### Tomohiro Ishii, Hironori Shibata, Goro Sasaki, Satoshi Narumi, Sato Takeshi, Mie Hayashi, Naoko Amano, Naoaki Hori, Mikako Inokuchi, Seiji Sato, Tsutomu Ogata, Nobutake Matsuo, Tomonobu Hasegawa

#### Department of Pediatrics, Keio University School of Medicine

**Background**: Micropenis or microphalus is defined as penile length (PL)-SDS less than -2.5 with the absence or presence of anatomical defects including hypospadias, respectively. We previously reported that intramuscular testosterone enanthate (TE) therapy effectively increased PL in boys with micropenis or microphalus. However, the effect of TE has never been compared among hypogonadotropic hypogonadism, hypergonadotropic hypogonadism, and idiopathic forms.

**Methods**: We retrospectively investigated the effect of TE in 60 Japanese boys (age, median 2.4 years, range 0.3 to 6.8) with micropenis (N=43) or microphalus (N=17) at Department of Pediatrics, Keio University Hospital. The inclusion criteria are as follows: (1) age AR and SRD5A2 mutation (Group 3). Increment of PL-SDS at the first injection of TE was primarily assessed between micropenis and microphalus or among Groups 1, 2, and 3 by non-parametric analyses.

**Results**: All the boys showed significant improvement of PL-SDS with up to four times TE injections (before TE, median -3.9, range -13.6 to -2.6; after TE, median -1.8, range -5.3 to 0.6; p <0.0001) without severe adverse events. Increment of PL-SDS at the first injection of TE was significantly correlated inversely with age (r =-0.45 and p =0.0003), not significantly correlated with PL-SDS before TE (r =-0.20 and p =0.13), not significantly different in boys between micropenis (median 1.3, range 0.0 to 8.1) and microphalus (median 1.3, range -0.1 to 4.3) (p =0.89), and significantly greater in group 1 (median 3.1, range 0.8 to 8.1) than in group 2 (median 0.9, range -0.1 to 5.9) or in group 3 (median 1.3, range 0.0 to 4.9) (p =0.026; p p

**Discussion**: The response of PL to TE therapy was significantly greater in boys with hypogonadotropic hypogonadism than those with other etiologies. These results suggest that patients with hypogonadotropic hypogonadism can relatively preserve PL potential, consistent with the previous findings by Bin-Abbas B, et al. that an adult PL in hypogonadotropic hypogonadism reached within 2 SD of the mean by replacement therapy at puberty.

## P2-6-14 The effect of leuprolide acetate treatment on luteinizing hormone suppression in children with central precocious puberty: a meta-analysis

### Ratna Dewi Artati, Aman Bhakti Pulungan, Kuntjoro Harimurti

#### Division of Endocrinology and Metabolism, Department of Child Health

**Background**: Central precocious puberty (CPP) is a characteristic development of sexual puberty as a consequence of premature activity of hypothalamic hypophyse gonadal (HHP) axis before 8 years old for girls or 9 years old for boys. Several studies have showed different results in Leuprolide Acetate (LA) therapy for CPP in terms of administration doses and time of treatment on suppression of gonadotropine secretion. Objective: To determine effects of different administration of LA therapy, monthly doses and every three month, on suppression of LH secretion in CPP patients.

**Method**: Meta-analyses of systematic review on available literature from Cochrane library, MEDLINE, EBSCO, PROQUEST and other registered reference about therapy to suppress LH secretion in CPP patients. Three researches independently conducted reviews on abstract and full- texts for inclusion criterion and data extraction, respectively.

**Result**: There are two studies fulfill inclusion criterion and included in the meta-analyses. Meta-analyses showed that LH suppression varies with different administration doses and time of LA. These studies compare LA therapy using 11,25 mg/3 month with control 7,5 mg/month, 22,5 mg/3 month with control 7,5 mg/month, and 22,5 mg/3 month with control 11,25 mg/3 month doses.

**Conclusion**: LA therapy 7,5 mg/month gives greater LH suppression compared with 11,25 mg/3 month and 22,5 mg/3 month; while LA therapy 22,5 mg/3 month provides greater suppression compared with 11,25 mg/3 month. There are no differences in growth velocity with different doses of LA therapy.

Key words: Central precocious puberty, LH suppression, leuprolide acetate

## P2-6-15 Relationship between the plasma level of Kisspeptin and Premature Thelarche in female infants

### Yu Yang, Renlong Zhang, Qiao Shi, Li Yang, Liling Xie

#### Department of Endocrine Genetics and Metabolism, Children's Hospital of Jiangxi Province

**Objective**: This study aimed to investigate the relationship between the plasma level of Kisspeptin and premature thelarche (PT) in female infants and risk factors associated with PT in female infants.

**Methods**: Forty-two female infants with an onset of PT under 2 years of age were selected from Children’s Hospital of Jiangxi Province during Sep 2013 to Mar 2016.Out of the 42 PT patients,18 cases were observed complete regression( CR group) of PT symptom, 11 were persistent or reproduction(PR group),13 were developed ICPP(ICPP group).Thirty-five healthy age-matched female infants were served as controls(control group).Plasma level of Kisspeptin was examined by enzyme linked immunosorbent assay(ELISA). Risk factors associated with PT were analyzed using multivariable logistic regression analysis.

**Results**: 1.In the ICPP and PR group, kisspeptin levels were significantly higher compared to control group (1.52 ± 0.14 ng/ml vs 0.45± 0.15ng/ml, P<0.05; 0.73 ± 0.12ng/ml vs 0.45 ± 0.15ng/ml, P<0.05).2.There were no significantly difference between CR and control group 3. There were association between the prevalence of PT and inharmonious relationship in parents, PT family history, mother’s menarche age, pollution of living environment, exposure to light at night.

**Conclusion**: These results suggest that kisspeptin maybe closely related with PT in female infants which will develop to ICPP and the kisspeptin levels could be a potential predict marker for these patients. Inharmonious relationship in parents, PT family history, mother’s menarche age, pollution of living environment, exposure to light at night were the risk factors to PT.

## P2-6-16 The diversity of etiologies and clinical course of gynecomastia

### Chong Kun Cheon, Yoo-Mi K, Su-Young Kim

#### Division of Endocrinology and Metabolism/Department of Pediatrics, Pusan National University Children's Hospital

**Objective**: Gynecomastia is defined as a benign enlargement of the male mammary glands due to an imbalance of free estrogen and androgen activity, resulting in palpable subareolar tissue. This condition is frequently benign in origin but may have a serious underlying etiology. In this study we sought to investigate various etiology and clinical course in patients with gynecomastia.

**Methods**: During 2009-2014, a total of 73 patients were diagnosed with gynecomastia. Clinical courses and the causes of gynecomastia were reviewed retrospectively.

**Results**: The number of patients showing gynecomastia based on onset-age was 15(20.5%) between 5 and 9 years of age, 39(53.3%) between 10 and 14 years of age, and 19(26%) between 15 and 18 years of age. The number of patients with gynecomastia based on age at visit was 10(13.8%) between 5 and 9 years of age, 31(42.4%) between 10 and 14 years of age, and 32(43.8%) between 15 and 18 years of age. The etiology of gynecomastia includes pubertal gynecomastia (75.3%), simple obesity (12.3%), central precocious puberty (2.7%), Cushing’ s syndrome (20.7%), Klinefelter syndrome (2.7%), idiopathic hyperprolactinemia (2.7%), subclinical hypothyroidism (2.7%), aromatase excess syndrome (2.7%), antiepileptic drug (2.7%), and ADHD drug (2.7%). Among the 55 patients with pubertal gynecomastia, 69% were only observed, 19% were treated with tamoxifen, and 12% underwent surgical procedure. Mean (SD) age of treated patients with tamoxifen was 14.6 ± 0.7 years with gynecomastia duration of 21.7 ± 4.8 months. Mean reduction in breast nodule diameter was 1.45 ± 0.69cm (p<0.0001) after treatment of 3.2 ± 0.9 months with tamoxifen. No side effects were reported in the treated subjects. Mean (SD) age of treated patients with surgical procedure was 17.4 ± 0.5 years with gynecomastia duration of 47 ± 7.3 months in addition to satisfactory results.

**Conclusions**: Considering the diversity of possible etiologies a careful history taking and physical examination or laboratory testing is imperative and treatment of the underlying cause is warranted after gynecomastia has been diagnosed.

## P2-6-17 Roles of metformin in the expression of aromatase mRNA and protein expression in insulin resistant 3T3-L1 preadipocytes

### Shanshan Zhang

#### Department of Pediatrics, General Hospital, Tianjin Medical University

**Objective**: To investigate the aromatase expression in the presence of insulin resistance, or the conditions with metformin interference. Methods: Insulin resistant 3T3-L1 preadipocytes were induced by glucose (25 mmol/L) and insulin (10-6mol/L). Cells were divided into three groups, including (i) blank control, (ii) preadipocytes with insulin resistance, and (iii) preadipocytes with insulin resistance subject to metformin with a concentration of 1.0mmol/L, 0.1 mmol/L and 0.01 mmol/L, respectively. Determination of aromatase (Cyp19a1) mRNA and protein expression was performed using Real-time PCR and Western blot analysis, respectively.

**Results**: After high glucose and insulin induction, the glucose consumption showed remarkable decrease compared with the baseline level (12.39 ± 2.14mmol/L vs. 5.74 ± 2.00mmol/L, P<0.05), while the glucose consumption ratio also showed obvious decrease compared with the baseline level (68.87 ± 11.91% vs. 31.91 ± 11.12%, P<0.05). Compared with the insulin-resistant cells, cells subject to low dose of metformin (10-5mol/L) induced no statistical difference, however, when cells were subject to metformin of 10-3mol/L or 10-4mol/L, remarkable decrease was noticed in the expression of Cyp19a1 compared with the insulin-resistant adipocytes. Compared with the mature adipocytes, the expression of aromatase in the insulin-resistant cells showed remarkable up-regulation. However, after metformin interference with a concentration of 10-3mol/L and 10-4mol/L, the expression of aromatase was obviously down-regulated. Compared with the metformin group, the expression of p-ERK showed obvious decrease in the cells subject to metformin and PD98059. The p-ERK/t-ERK showed remarkable decrease after PD98059 interference. Furthermore, the expression of Cyp19a1 gene expression after PD98059 showed remarkable increase compared with the cells subject to metformin.

**Conclusions**: Metformin could down-regulate the expression of Cyp19a1 mRNA and protein expression significantly, and the MEK/ERK signaling pathway involved in such process.

## P2-6-18 Peripheral precocity due to hcg secreting pineal tumor: a rare cause

### Sk H Rahaman

#### Department of Endocrinology, All India Institute of Medical Sciences

A boy of three and half year old brought to our department with complains of progressive phallic enlargement, noticed by the mother at the age of two and half year. Family members also noticed change of voice and appearance of pubic hair for 6 months. He had accelerated growth and aggressive behaviour. However, there was no facial hair. The child didn’t have headache, vomiting, visual impairment or seizure. Neither there was bony pain, fracture or deformity. He was born out of non-consanguineous marriage, full term. Mother’s pregnancy was uncomplicated. There was no birth asphyxia. His developmental milestones were normal. There was no significant medical history in the past. Family history was non-contributory.

He was alert, hyperactive, normotensive without having goiter, facial asymmetry or bony deformity. There was no hyperpigmentation. Anthropometrics revealed height 107cm, weight 22 kg (both >97th centiles, according to Indian chart), MPH 163cm (5th centile, according to Indian chart). Testicular volume bilateral 6ml, SPL 8cm and pubic hair Tanner stage 3. There was no hepato-splenomegally. Rest of the general and systemic examination revealed no abnormality. Investigations revealed normal hemogram, kidney and liver function. Bone age was 5.5 years. He had elevated testosterone (509.62 ng/dl) and suppressed gonadotropins (LH<0.21mIU/ml,'sH<0.66mIU/ml). hCG stimulation test didn’t increase LH levels. A diagnosis of peripheral precocity was made. On further evaluation, we found normal thyroid function, 17 hydroxy-progesterone and DHEAS. However serum beta-hCG level (31.49 mIU/L, normal level: To search for the source of beta-hCG, CECT chest and abdomen was done and was normal. MR sella and brain revealed a subtle abnormality in pineal gland but without any definite lesion. USG scrotum revealed no testicular mass. FDG PET scan and 68 Gallium DOTANOC PET CT scan also didn’t contribute. In view of non-localisation, CSF analysis was done. CSF beta-hCG level (49.21 mIU/L) came higher than peripheral blood and alpha feto protein level was normal with normal CSF cytology. To characterise the lesion of pineal gland, thin section MR brain was done which picked a 8.0 x 6.0 x 5.0 mm cystic pineal gland with blood fluid level within. A diagnosis of peripheral precocious puberty caused by beta-hCG secreting pineal tumor was made. After discussion with the parents and medical oncology opinion, cisplatin based chemotherapy was planned.

**Consent for publication:** The author declares that written informed consent was obtained for publication.

## P2-6-19 Challeges in the evaluation of primary amenorrhea

### Shaila S Bhattacharyya, Shobi Anandi

#### Division of Paediatric Endocrinology / Department of Pediatrics, Manipal Hospital

Evaluation of primary amenorrhea is a clinical challenge wherein one often comes across atypical clinical scenarios. We report 5 cases of primary amenorrhea to highlight the importance of a careful and individualized evaluation to arrive at a diagnosis.

Case 1 is a 16-year-old patient of primary amenorrhea with presence of secondary sexual characteristics compatible with her chronological age. On examination, a blind vaginal pouch was observed. Her pelvic ultrasound and magnetic resonance imaging revealed hypoplastic uterus without endometrial lining with atretic upper vagina. Bilateral ovaries were normal for age with numerous follicles. Hormonal profile was normal and karyotype was 46XX, thus leading to the diagnosis of Mayer-Rokitansky-Kuster-Hauser syndrome.

Case 2 is a 14 year old patient of primary amenorrhea and absent secondary sexual characteristics. Evaluation revealed female external genitalia, 46 XY karyotype, hypergonadotrophic hypogonadism, absent gonadal tissue and absent Mullerian structures. Laparoscopic examination is planned. The most probable diagnosis in this case is rare embryonic testicular regression or partial gonadal dysgenesis.

Case 3 is a 15 year old patient of primary amenorrhea and absent secondary sexual characteristics. Examination was suggestive of female external genitalia, 46 XX karyotype, hypergonadotrophic hypogonadism, hypoplastic uterus with bilateral small ovaries devoid of follicles. A diagnosis of primary ovarian insufficiency has been considered in this case.

Case 4 presented to us at 14 years of age with primary amenorrhea and absent secondary sexual characteristics. Ambiguous genitals were detected at birth; evaluation revealed 46 XY karyotype with hypoplastic mullerian structures and right sided gonad. At 7 months of age, she was subjected to clitoral resection and gonadectomy which revealed testicular tissue with ovarian stroma. Examination was suggestive of female external genitalia with clitoral scarring and a blind vaginal pouch, hypergonadotrophic hypogonadism, absent gonadal tissue and absent Mullerian structures. A diagnosis of partial gonadal dysgenesis versus ovotesticular DSD was considered.

Case 5 is a 15 year old patient of primary amenorrhea and absent secondary sexual characteristics. Examination was suggestive of female external genitalia, blind vaginal pouch, 46 XY/45XO karyotype, hypergonadotrophic hypogonadism, hypoplastic uterus with bilateral small ovaries. Laparoscopic examination was suggestive of rudimentary Mullerian structures and dysplastic gonads which were excised. She was diagnosed as case of Mixed Gonadal dysgenesis.

The above cases demonstrate the atypical presentations and varied constellation of clinical and laboratory features which can pose a diagnostic dilemma to the treating physician unless a systematic strategy for evaluation is employed.

**Consent for publication:** The authors declare that written informed consent was obtained for publication.

## P2-6-20 Hyperleptinemia in obese and non-obese children with early puberty

### Jang KyungMi, Moon JungEun, Cho EunMi, Choi ByungHo, Ko CheolWoo

#### Division of Endocrinology/ Department of Pediatrics, Kyungpook National University Children's Hospital

**Background**: Leptin is mainly produced by adipocytes. In animal and human, it is a potnet anorectic and increases in obesity. Some reported that precocious puberty is prevelent in children with obesity.

**Objective and hypotheses**: This study was done to see the changes of blood leptin levels in both obese and non-obese children with early puberty or precocious puberty.

**Method**: Study patients consist of 325 children with early puberty or precocious puberty showing abnormally high blood leptin levels (>7.8 ng/mL) who visited our institute for GnRH stimulation tests between Jan 2014 and Feb 2015. Their medical records were reviewed retrospectively. And their clinical and laboratory data was analyzed.

**Results**: They are 273 girls and 52 boys. Their mean ages are 9.2 ± 1.7 yrs. 254 out of 325 (78%) were non-obese, and 71 (22%) were obese. Hyperleptinemia was more frequent in non-obese group than obese group. Blood leptin levels (ng/mL) was significantly higher in obese group compared to non-obese group, 17.0 ± 7.8 vs 12.6 ± 4.9, respectively (p<0.001). In obese group, boy’s blood leptin levels (ng/ mL) are significantly higher than girl’s, 23.2 ± 11.2 vs 16.1 ± 6.8, respectively (p<0.05). Blood leptin levels does not show any significant difference between GnRH (+) (peak LH >5 mIU/mL) and GnRH (-) groups. In GnRH (+) group, their age was significantly older compared to the age of GnRH(-) group in both girls and boys (p<0.05). Interestingly, obese GnRH (+) girl’s age was younger than non-obese GnRH (+) girls (p<0.05), this would be said that obesity may associates with earlier pubertal onset in GnRH (+) girls with hyperleptinemia.

**Conclusion**: hyperleptinemia was more frequently found in non-obese sexually precocious children than expected. Further study regarding the mechanism of hyperleptinemia and its clinical significance in non-obese children with early puberty or precocious puberty is necessary.

## P2-6-21 Mood disorder in one of adolescent monozygotic twins with late-onset combined methylmalonic aciduria and homocystinemia due to MMACHC gene defect

### Xiaohong Chen, Lifang Feng, Dan Sun, Hui Yao

#### Wuhan Women and Children Medical care center, Endocrinology and Metabolism

This study reported a school boy, one of the monozygotic twins, with late-onset combined methylmalonic aciduria and homocystinemia (Cblc type) characterized as mood disorder. The previously happy boy presented with feeling sad, anxious, irritable, and lack of interest at the age of 12 years and 5 months . The patient's diagnosis was delayed until a metabolic workup revealed hyper-ratio of plasma propionylcarnitine ^acetylcarnitine (0.37 vs. normal range 0.04 to 0.25), hyperhomocysteinemia(>65umol/L vs. normal range 5-13.9umol^L), low methionine(5.525umol ^L vs. normal range 8 - 35umol ^L), and methylmalonic aciduria(197.88 mmol/mol creatinine vs normal range 0.2 to 3.6 mmol/mmol creatinine) . MRI showed cerebral atrophy. Molecular testing showed compound heterozygosity for mutations (c.482G>A and c.609G>A) in the MMACHC gene. After a month’s treatment with hydroxocobalamin, L-carnitine, betaine, and folate, progressive improvement has been observed. Another of the twins had no symptoms, and the MRI was normal, but similar changes were found in his metabolic workup, and the same mutations were found in his MMACHC gene, so we provided treatment for him too. Based on this case, we recommend that all teenagers with psychiatric disorders should be considered in making a differential diagnosis of inborn errors of metabolism.

**Consent for publication:** The authors declare that written informed consent was obtained for publication.

## P2-6-22 Body Mass Index and Body Fat Composition are Both Related to Central Precocious Puberty in Chinese Girls

### Ke Huang, Jun-fen Fu, Guan-Ping Dong

#### Division of Endocrinology, Children's Hospital of Zhejiang University, School of Medicine

**Aims**: To evaluate the fat mass and body composition by dual-energy x-ray absorptiometry (DEXA) in Chinese precocious puberty girls, and to examine the association between body fat composition, body mass index (BMI) and pubertal mutation in girls.

**Method**: CPP girls were diagnosed according to their clinical characters and the results of LHRH stimulating test. Total body fat mass and percent body fat (BF) was assessed with DEXA.

**Results**: Altogether, 26 central precocious puberty (CPP) girls and 28 age-matched prepubertal girls (age, 7 to 9 years) were enrolled in this study. The %BF of the CPP girls was significantly higher than control group (30.53 VS 28.33, p<0.05); while the BMI of the two groups was 16.84 and 15.78, respectively (p<0.05). In the CPP girls, the peak LH level in the LHRH stimulating test was negative correlated with the %BF and BMI.

**Conclusion**: Higher BMI and %BF were found in CPP girls. The use of DEXA has been shown to be a reliable and accurate measurement of fat mass. Because of its low cost and rapid administration, BMI is still a useful measurement for screening obesity and puberty.

## P2-6-23 Two Girls With Amenorrhoea Due to Müllerian Agenesis

### Kwong-tat Chan, Yuen-yu Lam

#### Department of Paediatrics, Kwong Wah Hospital

**Introduction**: Primary amenorrhoea is defined as either absence of menarche by age 14 if the girl has no secondary sexual characteristics development, or by age 16 in the presence of normal growth and secondary sexual characteristics. Secondary amenorrhoea is defined as the absence of menses for three months in ladies with previous normal menstruation and for nine months in those with previous oligomenorrhoea. Numerous conditions could lead to amenorrhoea. We would like to describe the clinical features and investigation findings of two girls who had amenorrhoea due to müllerian agenesis.

**Case 1**: She was born with low-birth-weight (1.91kg). She had bilateral inguinal hernia at 2 months old with herniotomy done. Karyotype revealed 46XX. Ultrasound at 5-year-old could not identify uterus and ovaries. Vagina was probably present. She defaulted follow-up since 6 years old. She was referred back to our hospital at age of 10 due to short stature. She had mild thoracic scoliosis. She had gradual pubertal development but did not have menarche. Investigations at 14 years old showed normal serum LH,'sH and oestradiol levels. Repeat of ultrasound pelvis failed to identify normal ovaries and uterus. MRI pelvis demonstrated normal ovaries but could not identify normal uterus and vagina. Impression was uterine agenesis

**Case 2:** She presented at age 13 because of obesity. She had breast development at 10 years old. She reported to have menarche at 13 years old, but she had no more menstruation afterward. She had progressive pubertal development thereafter. Medroxyprogesterone acetate was prescribed but no withdrawal bleeding occurred. Investigations showed normal serum LH,'sH and oestradiol levels. The uterus and ovaries were not visualised in ultrasound pelvis. MRI pelvis revealed normal ovaries but uterus was not seen. Vagina was probably rudimentary. The diagnosis was also müllerian agenesis.

**Discussion**: Müllerian agenesis, also known as Mayer-Rokitansky-Kuster-Hauser (MRKH) syndrome, is reported to be the second commonest cause of primary amenorrhoea. Majority of cases are sporadic. The estimated incidence is 1 in 4000-5000 liveborn females. About 15% of girls with primary amenorrhoea are caused by MRKH syndrome. It could be associated with other anomalies involving renal system, skeletal system and hearing. Paediatricians should consider this entity in the differential diagnoses of primary amenorrhoea, as counselling on fertility problem is needed. These girls should be referred to gynaecologist for creation of neovagina when they start to be sexually active.

## P2-6-24 Precocious Puberty Secondary to Juvenile Granulosa Cell Tumor - A Case Report

### Maria Luisa C Malabuyoc, Sioksoan C Cua, Dianne Marie R Delid

#### Philippine General Hospital, Section of Pediatric Endocrinology and Metabolism

Precocious puberty is characterized by early pubertal changes before the age of 8 years in girls and 9 years in boys. The etiologies for precocious puberty in young girls include central, peripheral, and sometimes idiopathic causes. In girls with peripheral precocious puberty, a common etiology is the presence of an ovarian tumor. This is a case of a 4-year old girl who developed prolonged vaginal bleeding, breast budding and abdominal enlargement. Diagnostic evaluation revealed elevated estradiol levels and presence of a left adnexal mass. Surgical excision of the tumor led to cessation of the vaginal bleeding and normalization of estradiol levels. Pathology reported a juvenile granulosa tumor of the ovary.

**Consent for publication:** The authors declare that written informed consent was obtained for publication.

**Fig. 1 (abstract P2-6-24).**
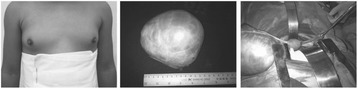
See text for description

## P2-6-25 Case Report: Sex Chromosome Disorder of Sexual Development: 45,X/46,XY Mosaicism

### Maria Luisa C Malabuyoc, Sylvia C Estrada

#### Philippine General Hospital, Section of Pediatric Endocrinology and Metabolism

A 45,X/46,XY mosaicism is associated with a broad spectrum of phenotypes ranging from apparently normal male development to individuals with incomplete sexual differentiation and accompanying clinical signs of Turner syndrome in both males and females [1]. This type of disorder of sexual development is associated with a numerical sex chromosome abnormality leading to abnormal gonadal development [2]. Among individuals with a 45,X/46,XY karyotype, sexual ambiguity is the most common presentation, accounting for approximately 60% [1]. This case report of an 11-year old male who presented with ambiguous genitalia at birth, documents the medical and genetic complexity of children with DSDs and the need for a multi-disciplinary team approach. The patient was born, preterm, first of twins, and was noted to have hypospadias with chordee and with non-palpable gonads. The twin brother exhibited a normal male genitalia. On physical examination, he has a short stature and examination of the external genitalia showed presence of a phallus, hypospadias with chordee, perineal body with a urethral orifice and hyperpigmented empty labio-scrotal folds, pubic hair tanner stage 2 and genitalia tanner stage 2. Diagnostic work-up revealed presence of mullerian structures (uterus, fallopian tubes, and cervix) and non-functioning gonads. Chromosomal analysis revealed 45,X/46,XY mosaicism. Total laparoscopic hysterectomy, bilateral gonadectomy and first stage repair of hypospadias were the surgical management of choice after the decision was made to continuously rear the patient as a male and to prevent tumor development. Growth hormone and testosterone were advised to the family as the most appropriate hormone treatment for the patient.

**Fig. 1 (abstract P2-6-25).**
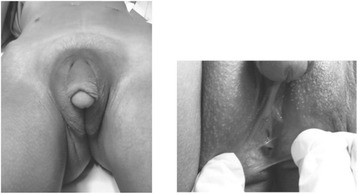
See text for description

**Consent for publication:** The authors declare that written informed consent was obtained for publication.

## P2-7-1 Three Chinese Patients from Two Kindreds with Aldosterone Synthase Deficiency: Clinical Characteristic with Mutation Analysis Report

### Melati Wijaya, Huamei Ma, Jun Zhang, Minlian Du, Yanhong Li, Qiuli Chen, Hongshan Chen, Song Guo

#### Division of Endocrinology and Metabolism/Department of Pediatrics, The First Affiliated Hospital of Sun Yat Sen University

**Introduction**: Aldosterone Synthase deficiency(ASD) is a rare autosomal recessive disease caused by CYP11B2 gene mutation, usually presenting with severe salt-wasting in infancy or stress-induced hyperkalaemia and postural hypotension in adulthood. ASD is unable to be detected by Neonatal screening of 17-hydroxyprogesterone, hence patients would not be diagnosed until they suffer from salt-wasting crisis.

**Objective**: to summarize the clinical features of ASD.

**Method**: By describing clinical features and outcomes.

**Result**: Two brothers (M1, M2) visited our unit for the first time at 2m and 2.5y respectively; the other patient is a 5month-old girl (F). All of three patients had repeated vomiting with poor feeding at newborn period and failure to thrive. They all visited local hospitals and were found to have hyperkalaemia, hyponatremia and metabolic acidosis. Treatment of underlying condition was started at that time, followed by giving sodium supplementary 2.0-4.5g/d. “F” has a healthy sister;and a brother who died 10 days after birth, was also suspected to be affected. All of them have normal external genitalia. “M1” and “F” laboratory test showed: hyperkalaemia [K+ 7.64~7.69mmol/l], hyponatremia [Na+ 123~132mmol/l], patient F had metabolic acidosis. Plasma ACTH, Cortisol, 17-hydroxyprogesterone, Androstenedione were normal. Plasma renin was highly elevated, while plasma aldosterone was in normal. Sequencing of CYP11B2 gene showed that “M1” and “M2” both carried same heterogenous pathogenic mutation: c.1121G>A (p.Arg374Gln) and c.1486delC p.(Leu496fs) , inherited from their parent. “F” homozygous for c.1303G>A p.(Gly435Ser) inherited from her mother who has generalized weakness with unknown newborn condition. “M2” had been given only sodium supplementation treatment till 6 month old. He presented normal growth and blood electrolyte till his last follow up at 4.33 years old. “M1” and “F” received fludrocortisone therapy at dosages of 0.1 mg Qd and 0.1 mg Bid, with sodium supplementation 2-3g/d and 3-6g/d respectively. “M1” mother only gave him fludrocortisone at 0.1g Qd and discontinued sodium therapy by herself when M1 started to receive his food supplements at 6 month old. He regularly followed up till his last visit at 1 years old, showed normal blood electrolyte and growth. “F” last visit was at 12m, still needed NaCl 3-6g/d supplementation and fludrocortisone therapy 0.1 g Bid. Her blood electrolyte was normal but her growth is slower than others her age.

**Conclusion**: We reported 3 Chinese patients from two kindred that have newborn salt-wasting clinical features. Adequate fludrocortisone therapy and sodium treatment brings good prognosis for patients.

**Consent for publication:** The authors declare that written informed consent was obtained for publication.

## P2-7-2 Unusual Presentation of Pediatric Pheochromocytoma and Paraganglioma

### Rapeepun Chai-udom^1^, Hataichanok Bansiddhi Kongmanas^1^, Apirak Santingamkun^3^, Thiti Snabboon^2^, Chucheep Sahakitrungruang^3^, Taninee Sahakitrungruang^1^

#### ^1^Division of Endocrinology and Metabolism / Department of Pediatrics, Faculty of Medicine, Chulalongkorn University; ^2^Division of Endocrinology and Metabolism / Department of Internal Medicine, Faculty of Medicine, Chulalongkorn University2; ^3^Department of Surgery, Faculty of Medicine, Chulalongkorn University

**Background**: Pheochromocytoma (PHEO) and paraganglioma (PGL) are rare catecholamine producing neuroendocrine tumors in children. Hypertension with headaches, sweating and palpitation are the common presenting symptoms of PHEO/PGL. Measurement of plasma free metanephrines or urinary fractionated metanephrines is the gold standard for diagnosis of PHEO/PGL. Generally, the tumor can be located by computed tomography (CT) scan, magnetic resonance imaging (MRI) or metaiodo-benzylguanidine (MIBG) scintigraphy.

**Case presentations**: We reported 3 cases of PHEO/PGL who had unusual presentation or trouble localizing the lesion. They were managed at the pediatric endocrine unit of King Chulalongkorn Memorial Hospital between 2011 and 2015. Table 1 summarized their presentation, management and genetic testing in brief.

**Cases 1**: A 14-year-old Thai boy presented with hypertensive crisis, congestive heart failure and chronic intestinal pseudo-obstruction. Case 2 was an 8-year-old Thai boy who presented with headaches, sweating, palpitation and hypertension. His plasma free metanephrines and urinary fractionated metanephrines were in normal range, but plasma Chromogranin A was high. Case 3 was a 10-year-old Thai boy presented with headache. He had high level of plasma normetanephrine and chromogranin A levels. Although there was no detectable lesion from CT scan of chest and abdomen in case 3, but 18F-fluorodihydroxy-phenylalanine (18-F-FDOPA) could detect the multiple lesions in pelvic cavity and anterolateral to urinary bladder, size up to 3.4 x 2.4 x 4.3cm. All of them underwent exploratory laparotomy with successful tumor removal and pathological examination revealed composite pheochromocytoma for case 1 and paraganglioma for case 2 and 3.

**Conclusion**: We report here the youngest case of pheochromocytoma manifested with chronic intestinal pseudo obstruction, a very rare but potentially life-threatening complication. Measurement of plasma chromogranin A can improve diagnostic accuracy in patients with normal levels of plasma or urinary fractionated metanephrines. The functional imaging modality such as 18-F useful to localize the lesion.

**Consent for publication:** The authors declare that written informed consent was obtained for publication

**Table 1 (abstract P2-7-2).**
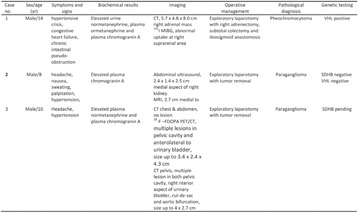
Summary of Cases

## P2-7-3 Addison disease in 16 Vietnamese patients with X-linked adrenoleukodystrophy

### Dung Chi Vu^1^, Ha Thu Nguyen^1^, Khanh Ngoc Nguyen^1^, Thao Phuong Bui^1^, Ngoc Thi BIch Can^1^, Hoan Thi Nguyen^1^, Nobuyuki Shimozawa^2^, Vu Anh Hoang^3^, Dat Phu Nguyen^1^

#### ^1^Department of Endocrinology, Metabolism and Genetics, Vietnam National Children's Hospital; ^2^Department of Pediatrics, Gifu University School of Medicine; ^3^University of Medicine and Pharmacy - Ho Chi Minh City, Center for Molecular Biomedicine

**Background**: X-linked adrenoleukodystrophy (X-ALD) is caused by a defect in the gene ABCD1.This disease characterized by progressive neurologic dysfunction, occasionally associated with adrenal insufficiency.

**Objective**: To describe the clinical, laboratory, cerebral MRI characteristics in Addison patients who have mutations of ABCD1.

**Method**: This is case series study. Clinical features, biochemical finding and cerebral MRI lesions of 16 cases from 14 unrelated families were studied.

**Results**: In this study, we had 12 cerebral adrenoleukodystrophy (CALD) cases, 3 Addison only (AO) cases and one adrenomyeloneuropathy (AMN) case. Approximately 50% of the CALD patients had adrenal insufficiency preceded the onset of neurological symptoms. In 5 cases, adrenal insufficiency symptoms were overlooked, the patients were only admitted to hospital when they had progressive neurological symptoms such as: hemiparesis or seizures. Endocrine inviestigations (9/16 cases) showed: low serum cortisol levels, and increased plasma ACTH. All of AO patients were followed up by cerebral MRI every 6 months. No patients had abnormal lesions in MRI. By sequencing of ABCD1 gene, 13 different mutations were identified, including 2 novel mutations [c.1668G>C (p.Q556H); c.292_296delTCGGC (p.S98Rfs*95)]. The reported mutation c.1628C>T (p.Pro543Leu) was identified in two cases (siblings: the elder was AO; the younger was CALD). The reported mutation c.1552 C>T (p.Arg518Tryp) was identified in two cases (siblings: the elder was AMN; the younger had slow progressive of CALD). Among the patients diagnosed with AO, none had acute adrenal crisis and no death was observed. All AO patients had normal plasma ACTH and good physical development after receiving hormone replacement therapy. By contrast, 5 deaths were observed among 12 CALD cases, and one of these deaths was due to acute adrenal crisis. Most of them had vegetative state for several years before death.

**Conclusions**: It is critical to perfom mutation analysis for X-ALD in boys diagnosed with unexplained adrenal insufficiency. If the test reveals an elevation in VLCFA consistent with X-ALD, additional assessments including a brain MRI and neurological and neuropsychological evaluations should be performed to examine cerebral involvement.

## P2-7-4 Growth analysis in 40 Japanese patients with 21OHD- influence of glucocorticoid dosage, genotype and phenotype on growth outcome

### Akari N Utsunomiya^1^, Kazuhiko Jinno^2^, Satoshi Okada^1^, Reiko Kagawa^1^, Sonoko Sakata^1^, Keiichi Hara^3^, Sinichiro Miyagawa^1^, Masao Kobayashi^1^

#### ^1^Department of Pediatrics, Hiroshima University Hispital; ^2^Department of Pediatrics, Hiroshima Prefectural Hospital; ^3^Department of Pediatrics, National Hospital Organization, Kure Medical Hopital

**Background**: An Endocrine Society Clinical Practice Guideline of steroid 21-hydroxylase deficiency (21OHD) recommend the dose of hydrocortisone (HC) should be less than 20 mg/m2/day during first year and less than 15 mg/m2/day during childhood. Height loss in patients with 21OHD is mentioned to be associated with the dose of HC in childhood, genotype, and compliance. However, it has not been elucidated which factor closely associates with growth.

**Objective**: To evaluate the influence of HC and 5 α fludrocorticosterone (F) dosage and genotype on growth pattern and outcome. METHODS: We analyzed 40 patients with 21OHD (17 had completed growth, 3 had been predicted final height, 20 still growing). Final height (FH) /predicted FH (pFH) and loss of height potential related to target height (TH) were calculated. The patients were divided into 4 groups depending on the dose of HC and evaluated height outcome (Group1: HC >20 mg/m2/day in first year, >15 mg/m2/day in 5-10 years old, Group2: HC 2/day in first year, >15 mg/m2/day in 5-10 years old, Group3: HC >20 mg/m2/day in first year, 2/day in 5-10 years old, Group4: HC< 20 mg/m2/day in first year, 2/day in 5-10 years old). Mean FH SD score (SDS) and pFH SDS were analyzed in accordance to genotype and dosage of HC and F. As for 20 patients before final height, we analyzed their growth pattern including bone mass age at 3 and 5 years old in each treatment groups and genotype.

**Results**: Mean (FH SDS and pFH SDS) was -1.1+/-0.83 SD. Two from 20 patients with final height were short stature (<-2SD). Mean (FH-TH) was -0.72+/-0.92 SD. Goup2 exhibited higher FH SDS, pFH SDS and smaller height losses. Patients harboring genotype associate with null enzymatic activity required higher dose of HC and F and exhibited smaller body height during early childhood. On the other hand, patients with I172N allele were treated with lower dose of HC and F than the others, but showed acceleration of bone age.

**Conclusions**: HC substitution in 21OHD patients should be kept at the lowest efficient level, if possible 2/day during the first year. However, bone age progression associate with too small dosage of HC should be prevented with the optimization of HC, especially under 10 years old. Genotype played only a minor role in growth outcome, but provided a supportive information about growth pattern.

## P2-7-5 The incidence and risk factors of adrenal crisis in Japanese patients with pediatric-onset adrenal insufficiency in a single institution

### Yosuke Ichihashi^1^, Tomohiro Ishii^1^, Hironori Shibata^1^, Naoko Amano^1^, Mikako Inokuchi^1^, Takayuki Abe^2^, Tomonobu Hasegawa^1^

#### ^1^Department of Pediatrics, Keio University Hospial; ^2^Department of Hygieiology and Public Health, Keio University

**Background**: European studies estimated the incidence of adrenal crisis (AC) among pediatric- and adult-onset patients with adrenal insufficiency (AI) as 3.6-8.3 per 100 person-years (PY), and determined risk factors for AC including younger age, female sex, coexistence of central diabetes insipidus (DI), primary AI with concomitant non-endocrine disease, and past history of AC. However, neither its incidence nor risk factors among Japanese patients with AI were published in the literature.

**Objectives**: To elucidate incidence and risk factors of AC among Japanese patients with pediatric-onset AI.

**Methods**: We retrospectively reviewed the medical records of patients who had been treated with oral glucocorticoid at Department of Pediatrics, Keio University Hospital, Tokyo, Japan, between January, 2012 and December, 2015. We selected patients with AI which was diagnosed biochemically or genetically such as congenital adrenal hyperplasia, adrenal hypoplasia congenita, or congenital or acquired combined anterior pituitary hormone deficiency. We excluded patients who started glucocorticoid replacement at the age of 18 and older or who had been followed less than 6 months. In this study, AC was defined as acute impairment of general health requiring intravenous glucocorticoid administration without any other causes. We collected data of age, sex, anthropometric data, duration of treatment, reported number of infections (fever, diarrhea, or emesis), and coexistence of central DI. The incidence of AC, death, severe neurologic sequelae was calculated as total number of each event divided by a total follow-up time. Risk factors for AC were assessed by multiple regression analysis with age, sex, types of AIs (primary or central), and reported number of infections.

**Results**: Eighty-three patients with a total follow-up time of 306.5 PY were included (33 males and 50 females; 51 with primary AI and 32 with central AI). The median age of the patients was 25.0 years (range 2-49). The incidence of AC was 7.1 per 100 PY [95% confidence interval (CI) 4.2-10.0]. Death was not observed, and severe neurologic sequela was 0.3 per 100 PY (95%CI 0.0-1.0). The reported number of infections was the only significant risk factor for developing AC (*p*<0.0001).

**Conclusion**: The incidence of AC among Japanese patients with pediatric-onset AI is similar to that in the previous European studies. The reported number of infection, which was not taken into account in the previous studies, is a risk factor developing AC in our cohort, irrespective of age, sex, type of AI, or coexistence of central DI.

## P2-7-6 Mortality Problems of Congenital Adrenal Hyperplasia in Central Java-Indonesia: 12 years experiences

### Agustini Utari^1,2^, Mahayu D Ariani^1^, Annastasia Ediati^3^, Achmad Z Juniarto^1^, Sultana MH Faradz^1^

#### ^1^Center for Biomedical Research (CEBIOR), Faculty of Medicine, Diponegoro University; ^2^Division of Endocrinology, Department of Pediatric, Faculty of Medicine, Diponegoro University; ^3^Faculty of Psychology, Diponegoro University

**Introduction**: Congenital Adrenal Hyperplasia (CAH) is an autosomal recessive inheritance due to the defect in the pathway that converts cholesterol into cortisol leading to decreased production of cortisol. Patients with CAH need life long treatment of glucocorticoid and sometimes mineralocorticoid. Early recognition and treatment of CAH can prevent adrenal crises. However, in our cohort of the classic type of CAH, the late-identified patients with CAH are more prevalent than early-identified patients who are different with countries that already have CAH newborn screening. Moreover, fatal adrenal crises are seen in clinical practice. Reports on mortality in patients with CAH in Indonesia are lacking. The aims of this present study were to report and identify the mortality problems of CAH in Indonesia.

**Methods**: A retrospective study was done in the Center for Biomedical Research (CEBIOR) Semarang, Central Java. Data related to mortality was derived from research database from 2004-2016.

**Result**: Of 78 patients with CAH (74 females and four males) in our cohort database, ten patients (12, 8%) were reported died due to adrenal crises. Among these ten patients had died of adrenal crises, six patients with CAH have already confirmed with hormonal and mutation analysis and four patients confirmed base on clinical and hormonal analysis. The mean age of diagnosis was 8.4 months (ranged from 1 month to 5.5 years). The average age of death was 22.3 month (ranged from 2 months to 6 years).

Among 78 patients, only 5% are 46 XY CAH, which may indicate that adrenal crises in male newborn might be under-diagnosed or unidentified. Nine siblings of seven families had a history of siblings who died because of a probable salt wasting condition before diagnosis confirmation. Thus the mortality rate of CAH in Indonesia is probably higher than this rate.

**Conclusion**: There was a high mortality rate of CAH in Central Java, Indonesia due adrenal crises. The high mortality rate can be anticipated or reduced if the Hydrocortisone and Fludrocortisone were available and affordable for patients with CAH. Findings from this study are strongly recommend for promoting early diagnosis and treatment as well as improving compliance of young patients with CAH. In addition, awareness of the medical community in the rural area and endorsement to the policy maker for availability of medication are essential to decrease the mortality rate of patients with CAH in Indonesia.

## P2-7-7 Clinical presentation and outcome of pheochromocytoma in children and adolescents

### Yoon-Myung Kim, Eungu Kang, Jin-Ho Choi, Han-Wook Yoo

#### Department of Pediatrics, Asan Medical Center Children's Hospital, University of Ulsan College of Medicine

**Objective**: Pheochromocytoma is catecholamine-producing adrenal tumors arising from neural crest-derived cells and characterized by triads of paroxysmal headache, palpitations, and diaphoresis. Pheochromocytoma may be associated with underlying hereditary conditions, such as von Hippel-Lindau disease, multiple endocrine neoplasia 2A and 2B, neurofibromatosis or tuberous sclerosis. This study investigated clinical presentation and outcome of pheochromocytoma in children and adolescents.

**Methods**: Seven patients (6 males and 1 female) were included. Clinical and radiological features, laboratory findings, perioperative management were reviewed retrospectively.

**Results**: The age at diagnosis was 11.4 ± 1.4years and follow-up duration was 5.9 ± 7.5 years (1 months – 17.5 years). All patients manifested with headache and hypertension (systolic and diastolic blood pressure 174.9 ± 14.1 and 123.7 ± 17.0 mmHg, respectively). Another presenting symptoms include decreased visual acuity (n=4), hyperhidrosis (n=3), weight loss (n=2), and seizure (n=1). Initial height- and weight-SDS were -0.1 ± 0.9 and -1.0 ± 0.6, respectively. A diagnosis was made by elevated 24 hour urine total metanephrine (5.8 ± 3.7 mg/day) in 6 patients and by elevated 24 hour urine norepinephrine (2968.7 ± 3295.3 μ g/day). Computerized tomography demonstrated tumors in adrenal gland; 5 in right side, 1 in left side, and 1 in bilateral. Hypertensive retinopathy (*n*=4) and left ventricular hypertrophy (n=4) were observed as complications of hypertension. VHL gene mutations were identified in 2 patients; one patient had previously diagnosed retinal hemangioma and the other developed contralateral pheochromocytoma after 12 years later. The other 5 patients did not reveal the features suggesting genetic syndromes. Phenoxybenzamine (n=4) was the most frequently used antihypertensive medication, and the duration of preoperative blood control was 20.6 ± 7.7 days. All patients had hypertensive episodes during the surgery, which were controlled by intravenous nitroprusside. One patient experienced postoperative hypotension and required intravenous phenylephrine. During follow up no tumor recurrence was observed.

**Conclusions**: Most patients initially presented symptoms associated with uncontrolled hypertension. Because pheochromocytoma could be the initial presentation of tumor-predisposing syndrome, regular monitoring is necessary after tumor resection.

## P2-7-8 Three pediatric patients with pheochromocytoma and paraganglioma

### Kenichi Yamamoto^1^, Satoshi Takakuwa^1^, Takeshi Usui^2,3^, Kei Miyata^1^, Shinji Takeyari^1^, Hirohumi Nakayama^1^, Keiko Yamamoto^1^, Makato Fujiwara^1^, Taichi Kitaoka^1^, Takuo Kubota^1^, Shigetoyo Kogaki^1^, Keiichi Oozono^1^

#### ^1^Department of Pediatrics, Osaka University Graduate School of Medicine; ^2^National Hospital Organization Kyoto Medical Center, Clinical Research Institute; ^3^Shizuoka General Hospital, Department of Clinical Genetics

**Background**: Pheochromocytoma and paraganglioma (PHEO/PGL) are neuroendocrine tumors that arise from adrenal medulla, and sympathetic and parasympathetic paraganglia due to various causes. Pediatric patients with PHEO/PGL are not common but more highly associated with genetic mutations than adults. We report three pediatric patients with PHEO/PGL due to various causes.

**Clinical Cases:** Patient 1 had abdominal pain after external injury at the age of 11. Abdominal computed tomography (CT) showed retroperitoneal masses. He had headache and hypertension. Urine normetanephrine levels elevated, and abdominal magnetic resonance imaging (MRI) revealed multiple abdominal PGLs. The PGLs were excised after the preoperative preparation ofα-blocker and saline infusion. We detected a germline mutation in the SDHD gene. Patient 2 had marfanoid habitus, chronic diarrhea and mucosal neuromas of the tongue at the age of 13. She was diagnosed as multiple endocrine neoplasia type 2B (MEN2B) due to a germline mutation in the RET gene. She also suffered from medullary thyroid carcinoma. Urine metanephrine levels elevated, and 123I-metaiodobenzylguanidine scintigraphy demonstrated bilateral PHEOs. Bilateral adrenalectomy after the preoperative preparation was performed before thyroidectomy. She had replacement therapy with hydrocortisone, fludrocortisone and thyroxine. Patient 3 had tricuspid atresia (cyanotic congenital heart disease: CCHD) followed by the Fontan surgery. She had experienced mild hypoxia since birth. She did not have any specific symptoms related to PHEO/PGL. During cardiac catheterization, her blood pressure increased markedly, and anα-blocker was administered. Urine normetanephrine levels elevated, and abdominal CT demonstrated a tumor in the right adrenal gland. Surgical treatment was performed, and PHEO was pathologically confirmed. We did not detect any germline mutations in the RET, VHL, SDHB, SDHD, TMEM127 and, MAX genes. Every patient had no family history of PHEO/PGL.

**Conclusion**: Approximately 70% of pediatric patients with PHEO/PGL have a germline mutation. Clinical features suspecting hereditary causes include a positive family history, syndromic features such as neurofibromatos type 1, MEN2 and von Hippel-Lindau (VHL) syndrome, and multifocal, bilateral, or metastatic conditions. In our study, the germline mutations were proven in the patients with multiple PGLs and MEN2B. Although the patient with PHEO accompanied by CCHD had no germline mutation, several studies report that hypoxia can increase a risk of PHEO/PGL. We conclude that genetic tests should be performed in pediatric patients with PHEO/PGL with a positive family history, syndromic features, and multifocal, bilateral, or metastatic condition and that continuous hypoxia can be a risk of PHEO/PGL.

**Consent for publication:** The authors declare that written informed consent was obtained for publication.

## P2-7-9 Evaluation of factors associated with elevated newborn 17 hydroxyprogesterone levels

### Shaila S Bhattacharyya, Shobi Anandi

#### Division of Paediatric Endocrinology, Department of Pediatrics, Manipal Hospital

**Introduction**: Measurement of 17 hydroxyprogesterone (17 OHP) in dried blood spots has been widely used as a newborn screening tool for congenital adrenal hyperplasia. Various maternal and neonatal factors can result in falsely high values of 17OHP.

**Aim**: To determine the effect of gender, mode of delivery, gestational age, birth weight, maternal and neonatal factors on the newborn 17OHP values.

**Methods**: Retrospective data related to newborn 17 OHP values and clinical characteristics were collected for the period between October 2013 and October 2015. The blood samples from total 2009 newborns were spotted on to filter paper between 48-72 hours of age and the 17 OHP concentration was measured using competitive Enzyme Immuno Assay. A single model including the following covariates was evaluated using analysis of variance (ANOVA): mode of delivery(normal/ caesarean section), gender, gestational age(term, preterm- 34 weeks), birth weight( 2.5 kg, maternal factors like gestational diabetes mellitus, hypertension, polyhydraminos, olighydraminos, antenatal steroid use and neonatal factors like fetal distress, respiratory distress syndrome, early onset sepsis, necrotizing enterocolitis and neonatal seizures. It was followed up with a post-hoc Tukey HSD test to determine the levels of statistical significance.

**Results**: The mean 17OHP value in the study group was 5.15 ± 2.97 ng/ml. There was no significant difference in 17 OHP values among males and females. The levels were significantly greater in preterm babies(7.31 ± 5.07 ng/ml, p<0.0001) and babies born by Caesarean section ( 5.32 ± 3.14 ng/ml, p<0.0001). Among the preterm babies, the levels were higher in the

**Conclusion**: Factors like mode of delivery, gestational age, birth weight and neonatal morbidities should be taken into account while interpreting newborn 17 OHP values.

## P2-7-10 Cerebral involvement in 16 Vietnamese patients with X-linked adrenoleukodystrophy

### Dung Chi Vu^1^, Ha Thu Nguyen^1^, Khanh Ngoc Nguyen^1^, Thao Phuong Bui^1^, Ngoc Thi Bich Can^1^, Hoan Thi Nguyen^1^, Nobuyuki Shimozawa^2^, Vu Anh Hoang^3^, Dat Phu Nguyen^1^

#### ^1^Department of Endocrinology, Metabolism and Genetics, Vietnam National Children's Hospital; ^2^Department of Pediatrics, Gifu University School of Medicine; ^3^University of Medicine and Pharmacy - Ho Chi Minh City, Center for Molecular Biomedicine

**Background**: X-linked adrenoleukodystrophy (X-ALD) is caused by a defect in the gene ABCD1 , which maps to Xq28 and codes for a peroxisomal membrane protein that is a member of the ATP-binding cassette transporter superfamily. This disease characterized by progressive neurologic dysfunction, occasionally associated with adrenal insufficiency.

**Objective**: To describe cerebral involvement in affected Vietnamese patients who have mutations identified in ABCD1 .

**Method**: This is case series study. Clinical features, biochemical finding and cerebral MRI lesions of 16 cases from 14 unrelated families were studied.

**Results**: We had 12 cereral adrenoleukodystrophy (CALD) cases and 1 adrenomyeloneuropathy (AMN) case. The patients admitted to hospital because of cerebral symptoms such as inattention, visual disturbances, seizure. Most of the CALD patients had late symptoms including cognitive impairment (12/12), extrapyramidal signs, aphasia (6/12), blind (3/12). Six of 12 patients had an IQ of < 70. Neuroimaging studies (cerebral MRI) showed classical patterns in all patients with neurological symptom. Only 4 patients had Loes score < 8 points. By sequencing the ABCD1 gene, 13 different mutations were identified, including 2 unreported mutations [c.1668G>C (p.Q556H); c.292_296delTCGGC (p.S98Rfs*95)]. The reported mutation c.1628C>T (p.Pro543Leu) was identified in two cases (siblings: the elder was AO; the younger was CALD). The reported mutation c.1552 C>T (p.Arg518Tryp) was identified in two cases (siblings: the elder was AMN; the younger had slow progressive of CALD). All of the CALD had an extremely rapid procession. Five of the 12 patients died during the following time, of whom one died from acute adrenal crisis, and the four patients had had vegetative state for several years before death.

**Conclusion**: Pediatricians, endocrinologists, neurologists and psychiatrists may encounter X-ALD in clinical practice. Recognition of X-ALD is highly important, since treatments, such as allogeneic HCT in the early stage of CCALD and endocrine replacement therapy for adrenocortical insufficiency, is available in some cases.

## P2-7-11 A rare combination: classic lipoid congenital adrenal hyperplasia and Turner syndrome

### Mahoko Furujo^1^, Toshihide Kubo^1^, Noriyuki Katsumata^2^, Yozo Ichiba^3^

#### ^1^Department of Pediatrics, Okayama Medical Center, National Hospital Organization; ^2^Department of Molecular Endocrinology, National Center for Child Health and Development; ^3^Department of Health, Okayama Institute of Health Foundation

**Introduction**: Lipoid congenital adrenal hyperplasia (lipoid CAH) is a rare autosomal recessive disorder that severely inhibits the synthesis of all adrenal and gonadal steroids. Lipoid CAH is ususally caused by mutations in the gene for steroidogenic acute regulatory protein ( StAR) , which is required for the movement of cholesterol from the outer to the inner mitochondrial membranes to synthesize pregnenolone. Turner syndrome is a condition involving total or partial absence of one X chromosome in all or part of the body’s cells, reduced final height, absence of female sex hormones. We report 33-year-old female with lipoid CAH due to StAR mutation and Turner syndrome concomitantly.

**Case**: A one-day-old patient, the second child of nonconsanguineous Japanese parents, was born at 42 weeks of gestation with a weight of 2.5 kg. The patient admitted due to adrenal salt wasting crisis, and hyperpigmention. She had the normal female external genitalia and no adrenal hemorrhage on ultrasonography.

**Results**: Her karyotype was reported as 46, X,idic(X)(p11.2)[20]/ 45, X[10] , based on which a diagnosis of mosaic Turner syndrome. In the StAR gene analysis, a homozygote or heterozygote Q258X mutation was detected. She was short stature, delayed puberty and osteopenia. Her treatment was hormonal replacement therapy with physiological doses of glucocorticoids and mineralocorticoids and with estrogens.

**Conclusions**: The pathogenesis of lipoid CAH with Turner syndrome has not been previously reported. However, an awareness of rare combination is important because of another future reports.

**Consent for publication:** The authors declare that written informed consent was obtained for publication.

## P2-7-12 A case of adult 11β-Hydroxylase deficiency associated with brain bleeding

### Yasusada Kawada^1,2^, Mami Eguchi^2^, Motohide Gotoh^2^, Shunsuke Araki^2^, Kazuyasu Kubo^2^, Rinko Kawagoe^2^, Yukiyo Yamamoto^2^

#### ^1^Department of Pediatrics, Kyushu Rousai Hospital; ^2^Department of Pediatrics, University of Occupational and Environmental Health

**Introduction**: 11β-Hydroxylase deficiency(11β-OHD) is hypertensive form of Congenital Adrenal Hyperplasia(CAH). This form of CAH is rare and represents about 5% of the number of 21-hydroxylase deficiency. Here we experienced the brain bleeding case with 11β-OHD.

**Case**: The patient was 31-year-old male with CAH; 11β-OHD. He and his younger sister were diagnosed as 11β-OHD at Hamamatsu medical school. Steroid replacement therapies were started with hydrocortisone. They transferred to Kitakyushu-city at age 8 and 7. At 16 year-old, his medicine was changed to dexamethasone(0.75mg/day). His blood pressure were almost 150-130/ 90 mmHg. Recent data before bleeding showed as follows; ACTH 202.8, PRA 0.9ng/ ml/ hr, Aldosterone 124 pg/ml, Na 141, K 4.1, Cl 104, BG 72. His career is care-helper. He worked hard and night duty was 5 times at 1-month.

**Brain Bleeding**: He had apraxia and conscious disturbance(E3, V4, M6). He and his mother visited our emergency clinic. His blood pressure was 153/ 106mmHg. Brain CT revealed brain bleeding at left frontal lobe with ventricular perforation. Brain MRI didnot present any vascular malformation. Conservative therapy(antihypertensive drug and antiepileptic drug) and steroid replacement were performed. His laboratory data were high ACTH(227.6 pg/ml) and DOC(1.10 ng/ml) levels. Na 138 CK 4.0, Cl 102, BG 74 were within normal range. He recovered well in final.

**Conclusion**: Brain bleeding happened in CAH; 11 β-OHD. Stress and poor compliance might lead to brain bleeding in this case. 11 β-OHD patients should check their blood pressure and keep normo-pressure with steroid supplement.

**Consent for publication:** The authors declare that written informed consent was obtained for publication.

## P2-7-13 A Japanese infant with secondary pseudohypoaldosteronism caused by urinary tract infection

### Masayo Yamazaki^1^, Toshihiro Tajima^1^, Koji Yokoyama^1^, Makiko Oguma^1,2^, Mizuki Kobayashi^1^, Takanori Yamagata^1^

#### ^1^Department of Pediatrics, Jichi Medical University; ^2^Department of Pediatrics, Japan Community Health care Organization Utsunomiya Hospital

## P2-7-14 Role of social media group for congenital adrenal hyperplasia patients in Indonesia

### Fia A Mutiksa^1^, Syeda T Noor^1^, I wayan B Suryawan^2^, Aman B Pulungan^2^

#### ^1^Faculty of medicine, University of Indonesia; ^2^Indonesian pediatric society; ^3^Indonesian pediatric society, Head of endocrinology

**Background**: Congenital adrenal hyperplasia (CAH) is a rare disease in the world, and also in Indonesia As an autosomal recessive disoder, it leads to enzyme deficiency involving hormones i.e cortisol, aldosteron, or both. Some problems of CAH management occure in Indonesia, especially unavailability of hydrocortisone. Parents or guardians of CAH patients utilize media social for daily communication.

**Objective**: To explore the role of social media group for solving CAH problems in Indonesia.

**Method**: This research used qualitative approach that was conducted on June 2016 by interviewing parents or guardians of CAH patients, KAHAKI (the CAH club in Indonesia), and Indonesian pediatric endocrinologists. The technique of data analysis was content analysis performing data analysis from the interview in verbatim form.

**Results**: There were 348 cases found but not all of the patients’ data were completely filled. Most cases were obtained in Jakarta (38,8%), Central Java (21,8%) and East Java (17,8%). All the informants confirmed that social media group was useful for CAH patients. Because of the limited medication, this group became a helpful media to inform the availability of hydrocortisone in Indonesia. Parents/guardians of CAH patients could also ask directly to pediatric endocrinologist in the group if something happened to their child. The group also build the connection between CAH patients and endocrinologists in various cities in Indonesia to share their problems and solve them together.

**Conclusions**: Social media group was useful for CAH patients, especially to inform availability of hydrocortisone in Indonesia.

**Keywords**: congenital adrenal hyperplasia, Indonesia, social media group

## P2-7-15 Adrenal crisis with systemic inflammatory response syndrome (SIRS). Report of 2 case series

### Toshihiko Takiura, Tomoko Ota, Atsushi Ogawa, Tetsushi Ogawa, Junko Ito

#### Department of Pediatrics, Toranomon hospital

**Introduction**: Adrenal crisis (AC) is a life-threatening complication of adrenal insufficiency (AI) and often induced by mild infection. In this status, stress dose of hydrocortisone (HC) should be administered immediately to save lives. We experienced two cases of AC with systemic inflammatory response syndrome (SIRS). Sepsis is defined as infection-induced SIRS and its first-line therapies are fluid resuscitation, vasopressors and antibiotics. It is difficult to differentiate AC from sepsis and it means a delay of administering HC for AI patient. We report two severe AC cases recovered from SIRS by the immediate administration of HC.

**Case 1:** 21 years old female. She has had panhypopituritarism, central diabetes insipidus (CDI) and hypothalamus disorder because of post-surgery for recurrent craniopharyngioma. She began to feel fatigue 5 days before admission and had fever 2 days before. But she didn’t increase the dose of HC for sick days. On arrival, she became unconscious and had developed shock status. The stress dose of HC was administered immediately. She was managed with respirator and vasopressors in ICU. Her symptoms and blood test on admission indicated SIRS and multiple organ failure (MOF) (SOFA score: 16 points). Her circulation was stabilized at 5 hours later. She could leave ICU at 9 days after admission, but mild cerebellar ataxia derived from hypoxic encephalopathy remained.

**Case 2:** 17 years old male. He also has had panhypopituritarism and CDI because of craniopharyngioma. He began to feel fatigue 2 days before admission. On the next day, his fatigue became more severe and had fever at night. He didn’t take any steroids because of low medication compliance. He became shock status in the morning on the day of admission. The stress dose of HC was administered immediately. His symptoms and blood test on admission indicated SIRS and MOF (SOFA score: 12 points). He needed vasopressor, but we could stop it at the next day. He had stayed in ICU for 4 days, but no complication remained.

**Conclusion**: Both cases indicate that immediate administration of HC is critical to save lives of AI patients, because it is difficult to distinguish AC from sepsis.

**Consent for publication**: The authors declare that written informed consent was obtained for publication.

## P2-7-16 A Case Report of Adrenocortical Adenoma in a Young Girl

### Huyen Thi Bich Tran

#### Children Hospital 1, Endocrinology

Adrenocortical tumors are rare childhood neoplasms. More than 95% are functional and present with virilization, Cushing’s syndrome, hypertension, or hyperestrogenism. We present a exceptionally rare case of a patient with androgen- and cortisol-co-secreting adrenal adenoma.

A 4-year-old girl was referred to us for appearance of symptoms of virilization: moustache, pubic hair, and gradual enlargement of clitoris for 1 years. Her voice gardually deepened and changed to male pattern. On systemic examination, her weight was 26kg, height was 112cm, BMI 17.46. Pulse was 100 beats/min, blood pressure was 100/60mmHg. She had no Cushingoid features. The laboratory showed that she had elavated 24 hour urine free cortisol level: 362.7 μg/dl (21-143 μg/dl) and serum testosteron: 286.5 ng/dl (14-76 ng/dl). The serum ACTH was low: 1.58 pg/ml (7.2-63.3 pg/ml). Levels of DHEAS were normal: 0.914 μg/ml (0.5-3.5μg/ml). Levels of 17OH(P), LH,'sH, Estrogen were normal range. The bone-age was 11 years.

Abdominal CT scan showed a left adrenal mass. It was decided to manage her with surgery to remove the tumor. Subsequently adrenalectomy was performed and histopathology study revealed a 5.5x 5 x3.5 adrenal adenoma. Follow-up of the patient showed that signs of virilization were suppressed. Serum testosterone levels dropped to normal after surgery, and remained normal. Two to six months after adrenalectomy, she was noticed to have significant symptoms of adrenal insufficiency and gradual enlargement of breast (Tanner B2). Laboratory tests showed: AM cortisol levels and ACTH levels were low on several occasions. The tests of diagnosis for precocious puberty (PP) were performed. After confirming the diagnosis adrenal insufficiency and PP the patient was given 5mg of hydrocortisone a day and 3.75mg of Diphereline a month.

In summary, a diagnosis of adrenal cortical adenoma requires surgical removal as early as possible to prevent the untoward effects of virilization or corticosteroid excess. Although the girl in the present study seems to have been cured, long-term follow-up is warranted.

**Consent for publication:** The author declare that written informed consent was obtained for publication.

## P2-8-1 A case with acrodysostosis and familial exudative vitreoretinopathy having p.R368X mutation in PRKAR1A gene

### Noboru Uchida^1,2^, Reiko Horikawa^1^

#### ^1^Division of Endocrinology and Metabolism, National Center for Child Health and Development; ^2^Department of Pediatrics, Saiseikai Utsunomiya Hospital

## P2-8-2 Research of bone mineral density for Chongqing children and adolescents aged 6 to 18 years

### Feng – Xiong

#### Chongqing Association of Physicians, Branch of Endocrine Metabolism Practitioner

**Objective**: To investigate the bone mineral density (BMD) for Chongqing children and adolescents from 6 to 18 years old; to evaluate the relationships between BMD vs age, sex, weight, height and puberty stage; furthermore to establish the reference database of BMD in children.

**Methods**: 1334 healthy and normal children aged 6 to 18 years (687 girls C647 boys) in Chongqing were sampled with stratified cluster sampling. The investigation items included the measurement of height, weight, breast development (girls) and testicular volume by orchidometer (boys), which evaluated using the criteria of Tanner. DXA scans were performed using Hologic bone densitometers (QDR 4500 Discovery Wi), the posterior-anterior spine and total hip were the selected sites. SPSS 17.0 of statistical software was used for data analysis.

**Results**: 1) The BMD increased progressively with age. In girls, the BMD grew obviously from 11 to 14 years, fastest from 12 to 13 years, slowly after 15. In boys, what was said above were respective from 12 to 16 years, from 15 to 16 year and after 16. 2) Regions of BMD grew gradually with the increase of pubertal stage. The BMD increased significantly during U stage to V stage, whice slowed down at Xstage. 3) Regions of BMD had no sexual difference aged 6 to 9 years, which were higher aged 12 to 13 years and lower aged 16 to 18 years in girls. 4) The BMD were correlated with height and weight in most age groups. 5) The 2nd, 10th, 25th, 50th, 75th, 90th, 98th smoothed percentiles curves of BMD-for-age and -2, -1, 0, +1, +2 Z-scores curves of BMD-for-Tanner stage for girls and boys were made out respectively.

**Conclusion**: We establish the normative curves of BMD-for-age and BMD-for-Tanner stage for Chongqing children and adolescents aged 6 to 18 years. Application of the curves will promote child discovering early growth disorder, and will be useful to diagnosis of diseases and assessment of therapeutic effects.

## P2-8-3 Importance of recognition of 3-M syndrome with CUL-7 mutation and response to growth hormone therapy

### Tadashi Moriwake^1^, Junko Kaneko^2^, Daisuke Miyahara^1^, Natsuko Futagawa^1^, Yusuke Higuchi^3^, Kosei Hasegawa^3^

#### ^1^Department of Pediatrics, National Hospital Organization Iwakuni Clinical Center; ^2^Kaneko Pediatric Clinic; ^3^Department of Pediatrics, Okayama University Hospital

**Introduction**: 3-M syndrome is rare disorder with both prenatal and postnatal severe growth retardation. Three genes involved in this disorder were reported, which were CUL-7, OBSL1, and CCDC genes. This disorder is considered as important cause of SGA dwarfism who do not show catch- up growth.

**Case 1**: 15years old female was referred for the investigation of the cause of severe growth retardation. No consanguinity. Short stature in brother (CASE2). She was born at 39w gestation. Her birth weight and height were 1672g and 41cm, respectively. Psychomotor development was normal during infancy. Growth retardation was prominent and no catch-up growth was observed. She was diagnosed as growth hormone (GH) deficiency and had been treated with GH for 2 years. GH therapy was discontinued because of judgement as insufficient growth promoting effect (SDS -5.98 to -4.65). Spontaneous menarche was observed at age 11. Micrognathia and long philtrum were obvious. Radiologically, educed diameter of vertebral body and slender tubular bones. Compound heterozygous mutations of CUL7 gene which were p.Gly569Lys's 4X and p.Arg1604Phe's 34X were detected.

**Case 2**: 11y old male, younger brother of case 1. He was born at 39w gestation with 45cm and 1798g of height and weight, respectively.

Psychomotor development was normal. No catch up growth and no pubertal change was observed. His height and weight were 111.4cm (-4.7SD) and 23.3kg(-1.65SD). Characteristic face with long philtrum and micrognathia was pointed out. Endocriological test revealed pubertal response of LH with undetectable level of testosterone and estradiol, and attenuated growth hormone response to pharmacological stimuli. Radiologically, reduced diameter of vertebral body and slender tubular bones. Bone age was 11y4m. Compound heterozygous mutations of CUL7 gene which were p.Gly569Lys's 4X and p.Arg1604Phe's 34X were detected. Early response against GH substitution revealed elevated IGF-I and PINP. On the other hand, responses of bone alkaline phosphatase(BAP) or telopeptide of N-terminal type I collagen (NTx) was not apparent, which suggested that abonormal signal transduction of IRS-1 function might affect bone turn over.

**Discussion**: The patient may have no mature protein of Cullin-7, and lack of Cullin-7 may disturb proper IRS-1 signal transduction. Bone turn over is not stimulated dramatically by GH. However, Mild height SD change during GH therapy in case1 apparent even this low turnover state.

**Conclusion**: In case of severe growth retardation, 3Msyndrome is should be considered. If available, GH can be a candidate for the treatment for the growth.

**Consent for publication:** The authors declare that written informed consent was obtained for publication.

## P2-8-4 A case of osteogenesis imperfecta caused by PPIB mutation (First Japanese case)

### Junko Kanno^1^, Sayaka Kawashima^1^, Chisumi Sogi^1^, Miki Kamimura^1^, Tetsuya Niihori^3^, Yoko Aoki^3^, Shigeo Kure^1^, Ikuma Fujiwara^2^

#### ^1^Department of Pediatrics, Tohoku University school of Medicine; ^2^Department of Pediatric Endocrinology and Environmental Medicine, Tohoku University school of Medicine; ^3^Department of Medical Genetics, Tohoku University school of Medicine

**Background**: Osteogenesis imperfecta (OI) is a heritable disorder of connective tissue characterized by increased bone fragility, low bone mass and bone deformity. The majority of patients with OI have a heterozygous mutation in either COL1A1 or COL1A2 , the genes that encode type 1 collagen. Recently, mutations in the PPIB encoding cyclophilin B were identified as causes of recessive OI. This protein forms a complex with P3H1 and CRTAP that 3-hydroxylates a proline residue on the α 1(I) chain (Pro986) and has cis/trans isomerase (PPIase) activity essential for proper collagen folding.

**Case presentation**: The patient was born at 39 wk of gestation after an uncomplicated pregnancy. She was the first child of non- consanguineous Japanese parents. She had a femur fracture seven days after birth. She had suffered femur fracture five times until the referral to our hospital at 6 years of age. At that time, her height was 108cm, and she did not show any clear blue sclerae or tooth hypoplasia. She could not walk alone without help. Lumbar bone mineral density (BMD) by DXA was extremely low (0.246 g/cm2, -5.81SD). With a diagnosis of OI type W, pamidronate treatment was started. Her BMD gradually increased, and she had not experienced any fractures afterwards. Now at 9 years of age, her height is 116.3 cm, and her BMD has been improved to 0.497 g/cm2 (-1.4SD). By whole exome sequencing, she was identified to have a homozygous mutation (c.377A>G, p.E126G) in the PPIB and her parents were heterozygous carriers. OI caused by the absence of cyclophilin B is much milder compared to the severe or lethal OI caused by a deficiency of P3HI or CRTAP. Our patient’s clinical phenotype was similar to those of patients described in the previous reports. The parents of the case were not related, but they were from the same community.

**Conclusions**: We identified a homozygous PPIB mutation in an OI patient. To our knowledge, this is the first Japanese family with recessive OI caused by PPIB mutation.

**Consent for publication:** The authors declare that written informed consent was obtained for publication.

## P2-8-5 A droplet digital PCR (ddPCR) can be a new diagnostic method for detection of mosaic mutations of GNAS in patients with McCune-Albright syndrome

### Satoshi Watanabe^1,2^, Sumito Dateki^1^, Akiko Nakatomi^3^, Kei Izumi^3^, Hiroyuki Moriuchi^1^, Kohichiro Yoshiura^2^

#### ^1^Department of Pediatrics, Nagasaki University Graduate School of Biomedical Sciences; ^2^Department of Human Genetics, Nagasaki University Graduate School of Biomedical Sciences; ^3^Isahaya Ryoiku Center

**Introduction**: McCune-Albright syndrome (MAS) is caused by a somatic mosaic mutation of GNAS . It was often difficult to detect the GNAS mutation using DNA from peripheral blood leukocyte because of the low rate mosaicism. Recent droplet digital PCR (ddPCR) technology have made it possible to detect low rate mosaic mutations using TaqMan probe of a target sequence. When FAM-labeled probe is designed for mutant and HEX-labeled probe is designed for wild, the mutant alleles will be distinguished from wild type alleles by their specific probe amplitude. We here present the trial to detect the GNAS mutations of in MAS using ddPCR.

**Materials and Methods**: Five MAS patients were participated in our study. DNA was obtained from peripheral blood leukocyte. Droplets were generated using Droplet Generator and the DNA was distributed to 20,000 droplets. The droplets were amplified and finally each droplets were measured fluorescence intensity one by one by Droplet Reader. The droplets including a mutant allele or a wild allele indicate strong amplitude of FAM or HEX respectively, and the mutant allele rate is calculated dividing the number of FAM-positive droplets (the copies of mutant allele) by the total amount of FAM-positive and HEX-positive droplets (= the total copies of DNA).

**Results**: The R201H mutation of a patient was detected to 9.4% of the mutant allele frequency by ddPCR. The detection limit of ddPCR was calculated to 0.2% by diluting the patient’s DNA with a control’s DNA. Three of the other four patient’s mutations were able to be detected when pre-PCR with PNA inhibiting wild type allele amplification was performed before the ddPCR. The detection limit of ddPCR after the nested PCR with PNA was calculated to 0.005%.

**Conclusion**: ddPCR is an extremely sensitive detection method and needs only about ten dollars per one reaction and several hours to detect the mutations. We here presented that detection limit of ddPCR in GNAS analysis is 0.005%.

## P2-8-6 Hypocalcemic Cardiomyopathy: A Rare Presentation of Hereditary Vitamin D-Resistant Rickets

### Pontipa Engkakul, Wiraporn Yodvisitsak, Ratthapon Wongwandee

#### Department of Pediatrics, Faculty of Medicine, Thammasat University

Hypocalcemia is a rare cause of dilated cardiomyopathy (DCM). Hypocalcemic DCM is usually reversible, however the prognosis depends on the severity and time of diagnosis. The most common cause of infantile hypocalcemic DCM is vitamin D deficiency. To our knowledge, hypocalcemic DCM due to hereditary vitamin D-resistant rickets has never been reported. We report a 5-month-old girl who presented with generalized tonic clonic seizure secondary to hypocalcemia. She had typical features associated with rickets including frontal bossing, rachitic rosary and widening of wrists and ankles. In addition, she had anterior mitral valve prolapse, mitral regurgitation and congestive heart failure secondary to DCM. Her investigations were compatible with hereditary vitamin D-resistant rickets. She responded well to high dose calcium and calcitriol supplementation. She had rapid improvement of left ventricular function after the treatment of hypocalcemia. Therefore, she was diagnosed as having DCM secondary to hypocalcemia. Despite complete recovery of left ventricular function, she still has mild anterior mitral valve prolapse and mitral regurgitation after 1-year of follow-up. In spite of the fact that DCM is usually idiopathic, hypocalcemia should be considered as one of the reversible etiologies.

**Consent for publication:** The authors declare that written informed consent was obtained for publication.

## P2-8-7 Relationship between plasma levels of vitamin D and metabolic syndrome-related components in Japanese obese children and adolescents

### Motohide Goto, Yukiyo Yamamoto, Masahiro Ishii, Mami Eguchi, Reiko Saito, Shunsuke Araki, Kazuyasu Kubo, Rinko Kawagoe, Yasusada Kawada, Koichi Kusuhara

#### Department of Pediatrics, University of Occupational and Environmental Health

Low plasma levels of vitamin D (25(OH)D) have been associated with the development of metabolic syndrome (MS) in adult. However, there was no report about the potential relationship of 25(OH)D with various MS-related components in childhood obesity and MS. We investigated the relationship between plasma levels of 25(OH)D and anthropometries or various MS-related components in Japanese obese children and adolescents. Seventy-four obese Japanese children and adolescents (37 boys and 37 girls) were enrolled, ages 4.4 to 16.9 years old. The relationship between plasma levels of 25(OH)D and anthropometries (BMI, BMI z-score, waist circumference), MS-related components (systolic / diastolic blood pressure, TG, HDL-C, AST, ALT, FBS, Insulin, UA, apo A1, apo A2, apo B, HOMA- IR, HOMA- β) were evaluated. We defined vitamin D levels equal to or less than 10 ng/mL as severe deficiency, 11- 19 ng/mL as mild deficiency and 20- 29 ng/mL as insufficient. Vitamin D levels equal to or more than 30 ng/mL were considered sufficient. Among 74 obese children, 25 were diagnosed as MS. Plasma levels of 25(OH)D were significantly decreased in the MS group, compared with the non-MS group (23.1 ± 2.05 ng/mL vs . 28.1 ± 1.28 ng/mL, p <0.05). The mean of body weight, POW, waist circumference, BMI, BMI z-score, AST, ALT, FPG, UA, HOMA-IR and HOMA- β were significantly higher in the lowest serum 25(OH)D group. The proportion of MS was significantly greater than the proportion of non-MS in the severe vitamin D deficiency group. By univariate linear regression analysis, plasma levels of 25(OH)D were inversely correlated with body weight, POW, BMI, waist circumference, systolic / diastolic blood pressure, HOMA-IR and HOMA-β. HDL-C was positively correlated with plasma levels of 25(OH)D. Plasma levels of 25(OH)D, which is decreased in Japanese children and adolescents with MS, are associated with anthropometries and MS-related components. Our results showed a trend similar to adults or adolescents, suggesting that vitamin D may play important roles in the development of MS even in earlier aged children. Vitamin D deficiency is a risk factor of MS in children and adolescents, and the screening of serum vitamin D levels are required in obese children and adolescents.

## P2-8-8 New enhancers were found involved in Shox gene expression

### Huiying Mao

#### Children's Hospital of ChongQing Medical University, Endocrinology and Metabolism of Pediatrics

**Objective**: To analyze the specific locations and quantities of conserved non-coding elements iCNEs of short stature homeobox containing gene (Shox), and to explore the romote-control mechanisms of CNEs for Shox gene.

**Methods**: Screened all CNEs of Shox gene by the method of comparative genomics, then plasmid containing different CNEs intervention in human embryonic kidney (HEK293T) cells, detecting Shox expression by quantitative Real-time PCR.

**Results**: The control group which transfected empty plasmid of excluding CNEs, Shox gene expression were no statistical significance compared with the blank load transfection group(p =0.9487) and in groups of CNE-4(p =0.6986) ACNE2(p =0.9352) ACNE3(p =0.9997) ACNE4(p =1) ACNE5(p =0.429) ACNE6(p =0.1066) ACNE7(p =0.4284) ACNE8(p =0.8373) ACNE12(p =1) ACNE13(p =0.1052) ACNE14(p =0.8651) and CNE15(p =0.9295)(P>0.05), while the differences of Shox expression in groups of CNE-5i(p <0.0001) ACNE-3(p <0.0001) ACNE- 2(p <0.0001) ACNE9(p <0.0001) ACNE10(p <0.0001) and CNE11(p <0.0001) was highly significant (P<0.05).

**Conclusion**: We have found two new CNEs(CNE10 ACNE11);2.CNE-5, CNE-3, CNE-2, CNE9, CNE10, CNE11 has being involved in the regulation of Enhancement of Shox expression in HEK293T cells.

**Key words**: Conserved non-coding elements, Shox gene, gene expression Cregulatory mechanism

## P2-8-9 The targeted next-generation sequencing revealed novel CUL7 mutations in a short stature patient born for small gestational age

### Tomozumi Takatani, Tadashi Shiohama, Rieko Takatani, Naoki Shimojo

#### Department of Pediatrics, Chiba University, Graduate School of Medicine

The mother and father of a patient are not consanguineous. He was born by vacuum extraction at 41weeks gestation without asphyxia. His birth weight, length and head circumstance were 2246g (-3.47SD), 47cm (-1.85SD) and 33.5cm (-0.34SD), respectively. He showed hypocalcemia at 7days after delivery due to transient neonatal hypoparathyroidism. The administration of alfacalcidol and calcium lactate normalized his calcium level. Calcium lactate administration and alfacalcidol administration were ceased at the age of 15 and 48 days, respectively. The three-year follow-up revealed normal cognitive development and no catch-up growth. He showed proportionated short stature without specific findings in radiographic examinations. A blood test revealed normal IGF-1 level. Growth hormone therapy for short stature born in small for gestational started at the age of 3 years when his height was 78.2 cm (-4.64 SD). His growth velocity showed mild increase in response to growth hormone. At the age of 5 years, growth hormone stimulation test by insulin showed normal growth hormone secretion. We performed a genetic analysis using an Illumina TruSight One sequencing panel on a MiSeq platform according to the manufacturer’s instructions (Illumina, San Diego, CA, USA). The sequencing reads were analyzed with Basespace VariantStudio ver. 2.2 (Illumina). Mutations were validated by standard sanger sequencing. We found novel compound heterozygous mutations (p.Trp68X and p.Gly1536Asp) in CUL7, which is known as a causative gene of 3M syndrome (OMIM#273750), a rare autosomal recessive primordial growth disorder characterized by intrauterine and postnatal growth disorder. His mother has a heterozygous mutation (p.Gly1536Asp). Targeted next-generation sequencing provided the definitive molecular diagnosis of 3M syndrome that is sufficient information for both disease prognosis and appropriate genetic counseling. Our case supports the usefulness of the clinical application of next-generation sequencing for undiagnosed growth disorders.

**Consent for publication:** The authors declare that written informed consent was obtained for publication.

## P2-8-10 An effective case of enzyme replacement therapy for perinatal hypophosphatasia with a novel mutation in ALPL gene

### Maki Oyachi^1^, Daisuke Harada^1^, Yuki Hanioka^1^, Natsuko Sakamoto^1^, Kaoru Ueyama^1^, Kawai Kondo^1^, Kanako Kishimoto^1^, Masafumi Izui^1^, Yuiko Nagamatsu^1^, Hiroko Kashiwagi^1^, Miho Yamamuro^1^, Yoshihito Ishiura^1^, Makoto Tamura^2^, Shin Kikuchi^2^, Tomoyuki Akiyama^3^

#### ^1^Department of Pediatrics, Japan Community Health care Organization (JCHO) Osaka Hospital; ^2^Department of Pediatrics and Neonatology, Takatsuki General Hospital; ^3^Department of Child Neurology, Okayama University Graduate School of Medicine and Dentistry; ^4^Department of Bone and Mineral Research, Osaka Medical Center and Research Institute for Maternal and Child Health

## P2-8-11 The radiological features in a patient of achondroplasia with p.Ser348Cys mutation in the FGFR3

### Daisuke Miyahara^1^, Tadashi Moriwake^1^, Rie Fukuhara^2^, Yusuke Higuchi^3^, Kosei Hasegawa^3^

#### ^1^Department of Pediatrics, National Hospital Organization Iwakuni Clinical Center; ^2^Department of Neonatology, Prefectural Hiroshima Hospital; ^3^Department of Pediatrics, Okayama University Hospital

**Aim and Method**: Achondroplasia (ACH, MIM#100800) is the most common cause of short-limb dwarfism. This disorder is caused by gain- of-function mutation of the fibroblast growth factor receptor-3 (FGFR3) gene on chromosome 4p16.3. More than 98% of patients with ACH have an identical glycine-to-arginine substitution at residue 380 of FGFR3 (G380R). We have been reported a female infant with p.Ser348Cys mutation in the FGFR3 which was not reported before. The patient with p.Ser348Cys mutation demonstrated typical rhizomelic phenotype at 9 month old. In this study, to estimate that the phenotype of the patient with p.Ser348Cys mutation was compatible with ACH, we analyzed the radiological features in the patients with p.Ser348Cys mutation in comparison with 10 infant ACH cases who had typical G380R mutations in FGFR3. Reported radiological features of ACH include caudal narrowing of the interpedicular distance, squared iliac wings, rhizomelic shortening of the limbs. We selected four radiological index, which included the ratio of the interpedicular distances at the first (L1) and the fourth (L4) lumbar vertebrae (L1/L4 ratio), the ratio of the interteardrop distance(I) to the pelvic width(P) (I/P), the ratio of the radial/humeral length (R/H), and the ratio of the tibial/femoral length (T/F).

**Results**: In the patient of p.Ser348Cys, L1/L4 ratio were average G380R range (1.0 and 1.1 in patient at 1 and 9 month, respectively. G380R:1.10+-0.12, mean+-SD). I/P ratio were low (0.34) at 1 month and higher (0.39) at 9 month (G380R 0.4+-0.018) , R/H ratio remained average G380R range (0.89, G380R:0.87+-0.058), T/F ratio showed average G380R range at 1month, but lower value at 9 month (0.83,0.74 at 1 and 9 month, G380R 0.84+-0.049).

**Discussion and Conclusion**: The most diagnostic radiologic criteria of ACH was considered as L1/L4 ratio. Including L1/L4 ratio, the patient with p.Ser348Cys demonstrated radiologically typical G380R data at one month. Lower T/F ratio at 9 month may reflect the sufficient growth of femur in this patient. In conclusion, our patient with p.Ser348Cys FGFR3 demonstrated typical ACH phenotype, however the growth of each bone should be carefully monitored.

## P2-8-12 Hypocalcemic seizure in infants with TBX1 gene mutation: importance of searching for thymic hypoplasia

### Kosei Hasegawa^1^, Hiroyuki Tanaka^2^, Yousuke Higuchi^1^, Hirokazu Tsukahara^3^

#### ^1^Department of Pediatrics, Okayama University Hospital; ^2^Department of Pediatrics, Saiseikai General Hospital; ^3^Department of Pediatrics, Okayama University Graduate School of Medicine, Dentistry and Pharmaceutical Sciences

22q11.2 deletion syndrome is characterized by characteristic facial appearance, hypoparathyroidism, thymic hypoplasia, congenital heart disease and so on. 22q11.2 legion included TBX1 and other 30 genes, and TBX1 is thought to be the central gene that causes 22q11.2 deletion syndrome. (Case) 2 months old boy is referred to child neurological department because of repetitive seizure attack. He was born in vaginal delivery and was fed by breast milk. Physiological examination showed no abnormalities except for polydactyly of right fifth finger of foot. Laboratory examination revealed severe hypocalcemia (4.7mg/dl), hyperphosphataemia(12.2mg/dl) and normal parathyroid hormone level (42pg/ml).25(OH) vitamin D level was 15 ng/ml(37.5nmol/L). Continuous calcium infusion and oral active vitamin D corrected hypocalcemia and hyperphosphataemia. Radiological examination revealed thymic hypoplasia that reminded us 22q11.2 deletion syndrome in combination of hypocalcemia, while deletion of 22q11.2 was not detected by choromosomal analysis and FISH analysis. Genetic analysis identified 11 base duplication (c.968-977dupAACCCCGTGGC) in exon 8 of TBX1. This mutation causes early termination in three isoforms of TBX1 mRNA and is considered to reduce expression of TBX1 mRNA. Appropriate amount of TBX1 expression is essential for hypoparathyroid gland and this mutation might causes hypoparathyroidism. His development and growth is normal at 7 years old. (Conclusion) In case of infantile hypocalcemia without typical physical symptom of 22q11.2 deletion, it is important to search thymic hypoplasia to detect TBX1 gene mutation.

**Consent for publication**: The authors declare that written informed consent was obtained for publication.

## P2-8-13 Comparison between serum vitamin D levels in precocious pubertal girls and normal girls

### Joon Woo Baek, Yeon Joung Oh, Min Jae Kang, Young Suk Shim, Il Tae Hwang, Seung Yang

#### Department of Pediatrics, Hallym University Sacred Heart Hospital

**Background**: Vitamin D deficiency has been associated with chronic diseases, such as diabetes mellitus, obesity and autoimmune disease. However, There are only a few studies about the correlation between Vitamin D levels and precocious puberty in girls.

**Objective**: In the previous study, vitamin D levels may be associated with precocious puberty. we also aimed to re-evaluate the relationship between serum 25-hydroxyvitamin D (25(OH)D) and precocious puberty in girls.

**Method**: A total of 155 girls with central precocious puberty (CPP) and 45 control girls were enrolled. Anthropometric measurement and serum level of 25(OH)D were estimated for all subjects.

**Results**: Mean 25(OH)D level of CPP group was 17.3 ± 5.6ng/mL, which was lower than the control group(19.0 ± 5.3ng/mL). there was significant difference in the mean serum 25OHD concentration between the precocious puberty group and the control group (P =0.042). After 25(OH)D levels be classified by month(season), Significant difference in the mean serum 25(OH)D concentration between the two groups was only winter (Dec~Feb). 113 of the 155 girls with CPP (72.9%) had 25(OH)D deficiency (defined as serum 25(OH)D 30 ng/mL). the prevalence of 25(OH)D was significantly higer odds ratio (OR, 2.35; 95% CI, 1.18-4.66, P =0.017) among CPP group than controls.

**Conclusion**: Our results showed that vitamin D level was significant association with precocious puberty. We also recommend further studies are required to identify the correlation vitamin D levels and precocious puberty.

## P2-8-14 A case of Hecht syndrome having bilateral hypoplastic mandibular condyles and shallow mandibular fossas

### Chieko Kusano^1^, Tomoyuki Yaguchi^1^, Naoaki Hori^1^, Yoshitake Sato^1^, Kazumi Izawa^2^, Rika Kosaki^3^, Kenjiro Kosaki^4^, Gen Nishimura^5^, Tomonobu Hasegawa^6^

#### ^1^Department of Pediatrics, Ota Memorial Hospital; ^2^Department of Oral and Maxillofacial Surgery, Ota Memorial Hospital; ^3^Division of Medical Genetics, National Center for Child Health and Development; ^4^Keio University School of Medicine, Center for Medical Genetics; ^5^Division of Radiology, Tokyo Metropolitan Children's Medical Center; ^6^Department of Pediatrics, Keio University School of Medicine

Hecht syndrome (OMIM#158300) also known as Trismus-Pseudocamptodactyly syndrome (TPS) is a rare autosomal dominant distal arthrogryposis clinically characterized by trismus, pseudocamptodactyly, feet deformities and short stature. Bilateral hyperplasia of coronoid processes was reported to cause trismus. Veugelers et al. (2004) identified MYH8 heterozygous mutation (c.2021G A; p.Arg674Gln) in TPS families. To date, all 8 families with TPS had Arg679Gln, who were molecularly studied and described.

A 10-year-old Japanese boy visited us because of short stature and abnormal multiple muscles and tendons. He received surgical fixation for bilateral vertical taluses at 4 years. Family history was unremarkable. His physical examination revealed short stature (-2.8SD), bilateral ptosis, abnormal multiple muscles and tendons; limited opening of mouth (trismus), limited dorsiflexion of neck, pseudocamptodactyly (partial flexion of the 3-5th fingers on dorsiflexed right hand) and camptodactyly of left 4-5th fingers, bilateral pes planovalgus, bilateral limited planter flexion of both ankle joints and bilateral downturning toes. According to these phenotypes, we clinically diagnosed him as Hecht syndrome. Endocrinological survey was normal. Bone X-rays showed multiple camptdactyly and feet deformities. 3-D CT scan showed bilateral hypoplastic mandibular condyles and bilateral flat mandibular fossas without hyperplasia of mandibular coronoid process. Molecular analysis identified heterozygous MYH8 sequence variation (c.2404G A; p.Val802lle). According to PolyPhen-2 analysis, Val802lle is predicted to be benign with a score of 0.000. Val802Ile is reported in the healthy people with MAF score T=0.000008/1.

We experienced a Japanese boy diagnosed as clinically definite Hecht syndrome. His CT scan showed bilateral hypoplastic mandibular condyles and shallow mandibular fossas, which were never reported. We propose that trismus in Hecht syndrome can be caused by the hypoplastic mandibular condyle and shallow mandibular fossa. Pathogenicity of Val802Ile remains to be determined in this case.

**Consent for publication:** The authors declare that written informed consent was obtained for publication.

## P2-8-15 First Korean Case of Infantile Hypophosphatasia with Novel Mutation in ALPL Gene

### Eun Kyung Cho^1^, Aram Yang^1^, Jinsup Kim^1^, Eu Gene Park^1^, Young Bae Sohn^3^, Su Jin Kim^4^, Sung Won Park^5^, Chang-Seok Ki^2^, Sung Yoon Cho^1^, Dong-Kyu Jin^1^

#### ^1^Department of Pediatrics, Samsung Medical Center; ^2^Department of Laboratory Medicine & Genetics, Samsung Medical Center; ^3^Department of Medical Genetics, Ajou University School of Medicine; ^4^Department of Pediatrics, Myungji Hospital, Seonam University College of Medicine; ^5^Department of Pediatrics, Jeil Hospital, Dankook University College of Medicine

Hypophosphatasia is a rare hereditary disorder characterized by defective bone and teeth mineralization and the deficiency of tissue non- specific alkaline phosphatase (TNAP) activity. We describe the clinical and biochemical findings as well as the molecular analysis of a Korean boy with infantile hypophosphatasia. A 1-month-old boy visited the clinic because of poor feeding, frequent vomiting, hypotonia, and failure to thrive from birth. Total calcium, phosphorous, alkaline phosphatase (ALP), parathyroid hormone (PTH), and 25-hydroxyvitamin D were 18.5 mg/dL (Reference range, RR, 8.5–10.5), 3.4 mg/dL (RR, 3.5–5.5), 7 IU/L (RR, 150–240), 0.9 pg/mL (RR, 12–95), and 32.7 ng/mL (RR, 10–60), respectively. Intravenous hydration with normal saline was started, and dietary calcium intake was restricted. Skeletal X-rays showed a markedly increased distance of the anterior fontanelle, impaired mineralization, and rachitic changes in the metaphyses. Based on clinical, biochemical, and radiological findings, hypophosphatasia seemed to be the most probable underlying etiology. By Sanger sequencing of the ALPL gene, we identified two heterozygous variants, including a missense (c.334G>A; p.Gly112Ser) and a nonsense (c.1039C>T; p.Gln347*) variant. The father and mother of the patient were confirmed to be heterozygous carriers of each variant. The c.334G>A (p.Gly112Ser) variant had previously been reported in a patient with lethal type hypophosphatasia, while the nonsense c.1039C>T (p.Gln347*) variant was novel. We concluded that the prompt and accurate diagnosis of the disorder, together with appropriate interventions including genetic counselling, is essential for avoiding preventable morbidity and premature mortality.

## P2-8-16 A novel mutation in COL2A1 gene in patient with Stickler syndrome type 1: a case report

### Yousuke Higuchi^1,2^, Kosei Hasegawa^2^, Miho Yamashita^2,3^, Hiroyuki Tanaka^4^, Hirokazu Tsukahara^1^

#### ^1^Department of Pediatrics, Okayama University Graduate School of Medicine, Dentistry and Pharmaceutical Sciences; ^2^Department of Pediatrics, Okayama University Hospital; ^3^Faculty of Human Life Sciences, Notre Dame Seishin University; ^4^Department of Pediatrics, Okayama Saiseikai General Hospital

**This abstract is not included as the full text has been published as**

Higuchi Y, Hasegawa K, Yamashita M, Tanaka H, Tsukahara H. A novel mutation in COL2A1 gene in patient with Stickler syndrome type 1: a case report, 2017, 11(236) doi 10.1186/s13256-017-1396-y https://jmedicalcasereports.biomedcentral.com/articles/10.1186/s13256-017-1396-y.

## P2-8-17 Correlation between Frequency of Bone Fracture and Health-related Quality of Life (HRQOL) in Children with Osteogenesis Imperfecta

### Trivanie S Elona^1^, Mentari Mentari^1^, Aman B Pulungan^1^,^2,3^

#### ^1^Faculty of Medicine, Universitas Indonesia; ^2^Department of Child Health, Cipto Mangunkusumo Hospital, Division of Endocrinology; ^3^Indonesian Pediatric Society

**Background**: Osteogenesis imperfecta (OI) is a genetic disorder of connetive tissue by mutations of COL1A1 and COL1A2 with frequent fractures as the main symptom because of reduced bone density. Case detection rate and treatment management of OI have become more favorable in Indonesia since Forum Osteogenesis Imperfecta (FOSTEO) was established in 2013. This study aims to assess the correlation between frequency of fracture after therapy and HRQOL in children with OI in Indonesia since there were no previous similar studies in Indonesia.

**Methods**: A cross-sectional study was conducted with all OI patients in Indonesian OI children registry and members of FOSTEO by using Pediatric Quality of Life InventoryTM (PedsQLTM) 4.0 Generic Core Scale. Other data were also collected i.e latest medication, and frequency of fracture. The questionnaire was filled by parents for younger age using online form, and parents who did not join WhatsApp group of FOSTEO were called and interviewed by phone.

**Results**: Among 126 patients in the registry data, 49 (38.8%) filled the questionnaire, 48 (38.69%) inactive numbers, 12 (9.52%) unanswered phone calls, 8 (6.35%) reported dead, 9 (7.14%) were not FOSTEO members. From 44 patients, only 41 filled the questionnaire with more than 50% items were completed, found that 51.2% girls, and 39% age 2-4 years old. Latest medications included pamidronate (14.6%) and zolendronic acid (85.4%). PedsQLTM showed HRQOL of children age 2-4, 5-7, 8-12, and 13-18 years old (mean score 69.57, 67.08, 58.83, 53.99 respectively). Statistically, frequency of fracture (median 1, min-max 0-10) had very weak correlation with HRQOL (mean 65.48 ± SD 14.71) by using Spearman test (r=0.251 and P= 0.188).

**Conclusion**: Frequency of bone fracture had very weak correlation with HRQOL of children with OI. Require more time to call all of the patients. Indonesian OI children registry needs to be updated.

**Keywords**: children, osteogenesis imperfecta, quality of life, frequency of bone fracture

## P2-8-18 Hypocalcemia due to vitamin D deficiency in an adolescent Japanese boy

### Naoki Miyahara^1^, Rei Nishimura^1^, Yuki Kawashima^1^, Masanobu Fujimoto^1^, Keiichi Hanaki^2^, Susumu Kanzaki^1^

#### ^1^Division of Pediatrics and Perinatology, Faculty of Medicine, Tottori University; ^2^Department of Women's & Children's Family Nursing, Faculty of Medicine, Tottori University

Introduction: Vitamin D deficiency (VDD) causes rickets, osteomalacia or hypocalcemia. Recently, reports of the rickets or hypocalcemia with VDD in infants has been increased in Japan, because of insufficient intake of vitamin D or calcium and decrease of exposure to sunlight. However, hypocalcemia resulting from VDD are extremely rare in adolescents. We report a case of VDD in a 14-year-old boy with no underlying diseases.

**Case report:** A 14-year-old Japanese boy was brought to our hospital during winter season because of tetany. He had heavily trained “judo” in the sport hall for 4 hours every day for 2 years, and had lived in insufficient sunlight exposure. His height was 165.8cm (-0.03SD), weight 55.0kg (-0.11SD) and BMI 20.0. He presented with both Chvostek’s sign and Trousseau’s sign, but had no bowleg or genu valgum. His laboratory studies showed decreased serum calcium level (5.4 mg/dL). His serum level of phosphate and alkaline phosphatase were 5.0 mg/dL and 1,941 U/L, respectively. His serum intact parathyroid hormone level showed increased (148 pg/mL), and serum level of 25-hydroxyvitamin D was extremely low (7 ng/mL). A radiograph of the wrists did not show any changes. The nutritionist in our hospital revealed that his intake of vitamin D and calcium were insufficient, and his intake of phosphate was increased. According to these findings, we diagnosed him as having hypocalcemia due to primary VDD. He was treated with calcium lactate (0.05 mg/kg/day) and alfacalcidol (2 μ g/day). By dietary counseling, nutritional balance of his diet was improved. His serum calcium normalized one week after treatment and his serum 25-hydroxyvitamin D level was also improved 1 month after treatment. He has never showed tetany and hypocalcemia after treatment. Discussion: We speculate that his lifestyle and dietary habit caused the VDD. Furthermore, the increased vitamin D requirement at puberty might worsen his VDD. We should consider VDD as the cause of hypocalcemia even in adolescent.

**Consent for publication:** The authors declare that written informed consent was obtained for publication.

## P2-8-19 Vitamin D deficiency mimicking Pseudohypoparathyroidism in a baby with symptomatic hypocalcaemia

### Pik Fung Wong, Yuen Yu Lam, Kwong Tat Chan

#### Department of Paediatrics, Kwong Wah Hospital

**Introduction**: Vitamin D deficiency is characterized biochemically by reduced serum 25-hydroxyvitaminD (25OH) level, hypocalcaemia, hypophosphatemia, increased alkaline phosphatase (ALP) and parathyroid hormone (PTH).

Pseudohypoparathyrodism (PHP) is a rare inherited disorder with end-organ resistance to PTH featuring hypocalcaemia, hyperphosphatemia, high PTH and decreased urinary phosphate excretion. They represented two important diagnoses of hypocalcaemia with distinctive features. We report a young infant with transient but significant vitamin D deficiency presented as symptomatic hypocalcaemia with seizures. The biochemical features mimicked PHP, and responded to treatment with vitamin D and calcium.

**Case report**: KL was a full term male baby with good past health, was feeding well on breast milk and formula with good weight gain. He was admitted on day 29 of life for repeated seizure attacks for 2 days. He had no clinical features of rickets nor dysmorphism. Low ionized calcium 0.72mmol/L, hyperphosphatemia 3.7 mmol/L, raised ALP 353 IU/L and PTH 8.4 pmol/L were found. Magnesium level was borderline low 0.64 mmol/L. The renal threshold phosphate concentration (TmPi/GFR) was high (>2mmol/L, ref 1.02-2mmol/L), and no hypercalciuria was found. Blood gas and albumin were normal. X-ray showed no ricketic changes. Seizure was treated with phenobarbitone and subsided after hypocalcaemia resolved. Intravenous calcium gluconate was given, followed by continuous calcium infusion and then oral calcium. Calcium level was normalized with no more seizure after 2 days.

Mother's blood for calcium and PTH were normal, vitamin D level was not checked. Mother’s blood test during 2nd trimester showed lowish phosphate (0.8mmol/L) and calcium (2.08 mmol/L). Patient’s total 25OH vitamin D, 25OH D2 and 25OH D3 were all low (<10nmol/L) indicating severe deficiency. Oral alphacalcidol was added with maximum dose of 0.25 mcg /kg/day. Calcium supplement was stopped on day 37 of admission. He was discharged after 6 weeks with alfacalcidol 0.25mcg/kg/day, which was gradually weaned off at age of 4 months. He was on formula milk after discharge. No mutation was found in GNAS gene for Albright Osteodystrophy. His calcium, PTH and vitamin D level were normal till last blood test at 27 months old.

**Discussion**: Vitamin D deficiency can mimic pseudohypoparathyroidism in the biochemical picture possibly due to end-organ resistance to PTH. It is possible that our patient was exposed to a low vitamin D level in utero (suggested by the lowish phosphate and calcium level of mother) and postnatally. As breast milk has low vitamin D level, vitamin D deficiency can be exacerbated.

## P2-8-20 A case with suspected primary hypoparathyroidism who showed atypically elevated PTH level

### Hiroko Yagi^1,2^, Kentaro Miyai^2^, Yukihiro Hasegawa^1,2^

#### ^1^Division of Genetic Research, Tokyo Metropolitan Children's Medical Center; ^2^Division of Endcrinoloy and Metabolism, Tokyo Metropolitan Children's Medical Center

**Background**: Parathyroid hormone (PTH) level is one of the important clinical markers to differentiate between primary hypoparathyroidism and pseudohypoparathyroidism (PHP).

Objective: To report a clinical course and molecular analysis of a hypoparathyroidism patient who presented hypocalcemia with elevated PTH level, atypical for primary hypoparathyroidism.

**Case**: A 13-year-old boy was referred to our hospital due to transient unconsciousness with hypocalcemia. He had been diagnosed as mild mental retardation when he was a junior high school student. His facial appearance was unremarkable. Blood test showed Ca < 5 mg/dl, P 7.7 mg/dl, ALP 1526 U/L, intact PTH (iPTH) 164 pg/ml, Mg 1.9 mg/dl, urine test showed Ca/Cr < 0.01 mg/mg. Radiography of the left hand showed neither brachydactyly nor rickets. Ultrasonography revealed no malformation of heart or kidney. Head computed tomographic scanning showed bilateral calcification of the basal ganglia. He was successfully treated by administration of intravenous calcium gluconate and oral alfacarcidol. After his serum calcium level was normalized (iPTH level was 24 pg/ml), urinary cAMP elevated in response to the PTH injection. Neither of the copy number alteration nor the methylation alteration was detected at the GNAS locus.

**Discussion**: Urinary cAMP response to the PTH injection and the subsequent molecular analysis at the GNAS locus was not suggestive of PHP1b. In primary hypoparathyroidism, 22q11.2 deletion syndrome presents broad severity. However other syndromes and gene mutations that cause primary hypoparathyroidism have rarely been reported to show hypocalcemia with elevated PTH levels. Molecular analysis for primary hypoparathyroidism needs to be done in our case.

**Conclusion**: Our case suggests that PTH level may not be useful to differentiate between primary hypoparathyroidism and PHP.

**Consent for publication:** The authors declare that written informed consent was obtained for publication.

